# Supplementary material for: Palladium-catalyzed asymmetric carbene coupling en route to inherently chiral heptagon-containing polyarenes
Source: Nat Commun. 2024 Apr 18;15:3353. doi: 10.1038/s41467-024-47731-5 (PMC11026441; doi:10.1038/s41467-024-47731-5)
Supplement: Supplementary file 1 — Supplementary Information [file 41467_2024_47731_MOESM1_ESM.pdf]

Supplementary Information

Palladium-catalyzed asymmetric carbene coupling en route to inherently chiral heptagon-containing polyarenes

Huan Zhang[1†], Chuan-Jun Lu[1†], Gao-Hui Cai[1], Long-Long Xi[1], Jia Feng[1] & Ren-Rong Liu[1]*

[1]College of Chemistry and Chemical Engineering, Qingdao University, NingXia Road 308#, Qingdao 266071, China

*Corresponding author: E-mail: renrongliu@qdu.edu.cn

Table of Contents

1	General information	S1
2	Synthesis of substrates **1**	S1
3	Characterization and NMR spectra of products **I** and **1**	S5
4	Optimization of Reaction Conditions	S24
5	Synthesis of substrates **3**	S26
6	Characterization and NMR spectra of products **3**	S27
7	HPLC analysis of **3**	S122
8	Synthetic transformations	S164
9	Gram scale reaction	S174
10	Crystallographic data for **3w**	S175
11	Rotational barriers	S192
12	HRMS of all substrates and products	S200
13	Supplementary References	S222

1. General Information

Unless stated otherwise, all reagents were purchased from commercial sources and used without further purification. Solvents were dried and distilled before use by standard procedures. Reactions were monitored by thin layer chromatography (TLC) using silica gel plates. Flash column chromatography was performed over silica gel (200-300 mesh). NMR spectra were recorded on a Bruker Avance operating at for **^{1}H NMR** at 400 MHz (600 MHz), **^{13}C NMR** at 100 MHz (150 MHz), **^{19}F NMR** at 376 MHz (565 MHz), and chemical shifts (δ) are reported in ppm relative to those of residual solvent signals: $CDCl_3$ (**^{1}H NMR** δ 7.26, **^{13}C NMR** δ 77.00), $(CD_3)_2SO$ (**^{1}H NMR** δ 2.50, **^{13}C NMR** δ 39.60). All coupling constants (*J*) are reported in Hz. The following abbreviations were used to describe peak splitting patterns when appropriate: s = singlet, d = doublet, t = triplet, q = quartet, m = multiplet, br = broad. HRMS were recorded on Waters Xevo G2-XS QT of mass spectrometer. The enantiomeric excesses of the products were determined by HPLC analysis on Shimadzu LC-20AT, using Chiralpak AD-H (4.6 mm Φ × 250 mmL), IA-H (4.6 mm Φ × 250 mmL), OJ-H (4.6 mm Φ × 250 mmL), OX-H (4.6 mm Φ × 250 mmL), AS-H (4.6 mm Φ × 250 mmL) columns purchased from Daicel Chemical Industries.

2. Synthesis of *N*-arylsulfonylhydrazones 1a-1i

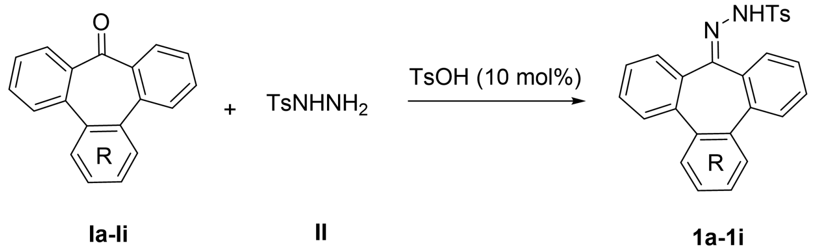

(1) Synthesis of 7-Membered cyclic ketones I

7-Membered cyclic ketones **Ii** is commercially available, **Ia**,[1] **Ig**[2] and **Ih**[3] were known compounds and were synthesized following the reported procedures.[1-3] **If** was synthesized following the reported procedure.[4] Cyclic ketones **Ib-Ie** used in this study were prepared using reductive Heck and the subsequent dehydration reaction.

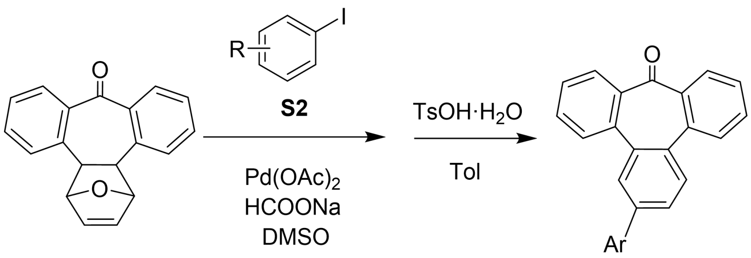

SI I

Procedure A: Under N_2 atmosphere, to a mixture of **S1** (5 mmol),[1] **S2** (7.5 mmol), $Pd(OAc)_2$ (10.0 mol%), HCOONa (10 mmol) in DMSO (40 mL) was stirred at 100 °C using oil bath for 14 h. After the mixture was cooled to room temperature, H_2O (10 mL) was added. The mixture was diluted with EtOAc (5 mL) and extracted with EtOAc (5 mL × 3). The combined organic layers were washed with brine (10 mL), dried (Na_2SO_4), and concentrated.

The above mixture (3 mmol), $TsOH·H_2O$ (6 mmol) was stirred in toluene (30 mL) at 120 °C using oil bath. After completion of the reaction which was indicated by TLC, the reaction mixture was concentrated under reduced pressure. The residue was purified by silica gel column chromatography to obtain **I**.

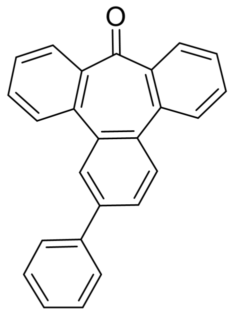

2-Phenyl-9H-tribenzo[a,c,e][7]annulen-9-one (Ib) was synthesized by following Procedure A. The crude material was purified by column chromatography (SiO_2, petroleum ether: EtOAc = 20:1) to provide **Ib** as a white solid (0.57 g, 68% yield).

^{1}H NMR (400 MHz, $CDCl_3$) δ 7.88 (s, 1H), 7.79 (dd, J = 12.8, 7.8 Hz, 2H), 7.75 – 7.66 (m, 6H), 7.61 (tt, J = 7.8, 1.4 Hz, 2H), 7.49 (td, J = 7.1, 6.5, 3.9 Hz, 4H), 7.43 – 7.39 (m, 1H).

^{13}C NMR (150 MHz, $CDCl_3$) δ 198.7, 143.0, 142.9, 141.1, 140.1, 137.1, 137.0, 136.8, 135.6, 131.7, 131.5, 129.8, 129.2, 129.0, 128.9, 128.2, 128.1, 127.8, 127.1, 127.0, 126.7, 126.6.

HRMS: (ESI) m/z: $[M+H]^+$ Calcd for $C_{25}H_{17}O$ 333.1274; Found 333.1271.

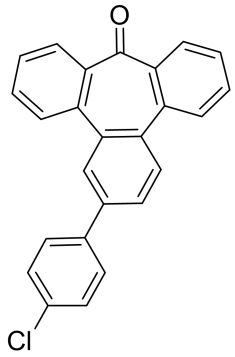

2-(4-Chlorophenyl)-9H-tribenzo[a,c,e][7]annulen-9-one (Ic) was synthesized by following Procedure A. The crude material was purified by column chromatography (SiO_2, petroleum ether: EtOAc = 20:1) to provide **Ic** as a white solid (0.62 g, 70% yield).

^{1}H NMR (400 MHz, $CDCl_3$) δ 7.94 – 7.74 (m, 3H), 7.73 – 7.68 (m, 3H), 7.66 (dd, *J* = 8.2, 2.0 Hz, 1H), 7.60 (t, *J* = 8.0 Hz, 4H), 7.52 – 7.43 (m, 4H).

^{13}C NMR (150 MHz, $CDCl_3$) δ 198.6, 143.0, 142.9, 139.8, 138.5, 137.1, 136.9, 136.6, 135.9, 133.9, 131.8, 131.5, 129.6, 129.1, 129.1, 129.0, 128.3, 128.3, 128.2, 126.7, 126.7, 126.6.

HRMS: (ESI) m/z: $[M+H]^+$ Calcd for $C_{25}H_{16}OCl$ 367.0884; Found 367.0891.

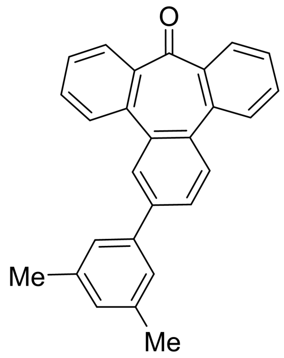

2-(3,5-Dimethylphenyl)-9H-tribenzo[a,c,e][7]annulen-9-one (Id) was synthesized by following Procedure A. The crude material was purified by column chromatography (SiO_2, petroleum ether: EtOAc = 20:1) to provide **Id** as a yellow solid (0.61 g, 69% yield).

^{1}H NMR (400 MHz, $CDCl_3$) δ 7.86 (d, *J* = 1.4 Hz, 1H), 7.80 (ddd, *J* = 17.8, 7.9, 1.2 Hz, 2H), 7.72 (td, *J* = 4.1, 1.5 Hz, 4H), 7.62 (qd, *J* = 7.4, 1.5 Hz, 2H), 7.52 – 7.46 (m, 2H), 7.31 (s, 2H), 7.06 (s, 1H), 2.43 (s, 6H).

^{13}C NMR (150 MHz, $CDCl_3$) δ 198.7, 143.0, 142.9, 141.3, 140.0, 138.4, 137.2, 136.9, 135.4, 131.5, 131.5, 129.8, 129.4, 129.2, 129.0, 128.1, 128.0, 127.0, 126.6, 126.6, 125.0, 21.4.

HRMS: (ESI) m/z: $[M+H]^+$ Calcd for $C_{27}H_{21}O$ 361.1587 ; Found 361.1591 .

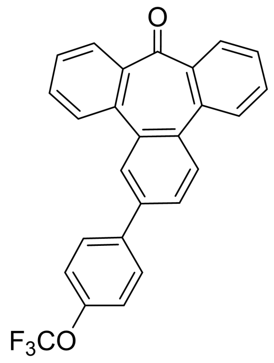

2-(4-(Trifluoromethoxy)phenyl)-9H-tribenzo[a,c,e][7]annulen-9-one (Ie) was synthesized by following Procedure A. The crude material was purified by column chromatography (SiO_2, petroleum ether: EtOAc = 20:1) to provide **Ie** as a yellow solid (0.75 g, 60% yield).

^{1}H NMR (400 MHz, $CDCl_3$) δ 7.74 – 7.57 (m, 9H), 7.52 (t, J = 7.6 Hz, 2H), 7.44 – 7.38 (m, 2H), 7.25 (d, J = 8.2 Hz, 2H).

^{13}C NMR (100 MHz, $CDCl_3$) δ 198.5, 148.9, 143.0, 143.0, 139.6, 138.8, 137.1, 136.9, 136.6, 135.9, 131.8, 131.5, 129.8, 129.1, 129.0, 128.5, 128.3, 128.2, 126.8, 126.7, 126.6, 121.8, 121.3, 119.2.

HRMS: (ESI) m/z: $[M+Na]^+$ Calcd for $C_{26}H_{15}F_3O_2Na$ 439.0916; Found 439.0917.

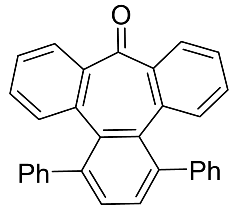

1,4-Diphenyl-9H-tribenzo[a,c,e][7]annulen-9-one (If) was synthesized by known literature.[3]

^{1}H NMR (400 MHz, $CDCl_3$) δ 7.59 – 7.49 (m, 4H), 7.19 (q, J = 47.9, 28.5 Hz, 11H), 6.95 (dt, J = 15.1, 7.7 Hz, 5H).

^{13}C NMR (100 MHz, $CDCl_3$) δ 199.7, 145.7, 142.3, 142.1, 135.5, 134.2, 133.6, 131.2, 129.6, 128.9, 128.0, 127.5, 126.4, 124.7.

HRMS: (ESI) m/z: $[M+H]^+$ Calcd for $C_{31}H_{21}O$ 409.1587; Found 409.1593.

(2) Synthesis of *N*-arylsulfonylhydrazones 1a-1i (Procedure B)

To a mixture of **I**[1] (1 mmol), **II** (1.2 mmol), TsOH (10 mol%) in MeOH (4 mL) was stirred at 70 °C overnight. After completion of the reaction (monitored by TLC), the crude material was purified by column chromatography to provided **1**.

3. Characterization and NMR spectra of products I and 1

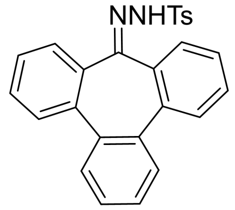

4-Methyl-N'-(9H-tribenzo[a,c,e][7]annulen-9-ylidene)benzenesulfonohydrazide (1a) was synthesized by following Procedure B. The crude material was purified by column chromatography (SiO_2, petroleum ether: EtOAc = 5:1) to provide **1a** (327 mg, 77% yield) as a white solid.

^{1}H NMR (400 MHz, $CDCl_3$) δ 7.93 (s, 1H), 7.67 (dd, J = 7.8, 1.3 Hz, 1H), 7.58 (s, 1H), 7.56 (s, 1H), 7.55 – 7.51 (m, 2H), 7.49 (d, J = 1.4 Hz, 1H), 7.47 (s, 1H), 7.45 – 7.43 (m, 2H), 7.43 – 7.35 (m, 4H), 7.23 (dd, J = 7.6, 1.5 Hz, 1H), 7.07 (d, J = 8.1 Hz, 2H), 2.34 (s, 3H).

^{13}C NMR (100 MHz, $CDCl_3$) δ 155.0, 143.7, 140.4, 137.7, 137.4, 137.0, 136.4, 134.9, 133.9, 130.9, 130.8, 130.4, 130.0, 129.4, 129.3, 128.9, 128.3, 128.0, 128.0, 127.7, 127.4, 126.0, 124.9, 21.5.

HRMS: (ESI) m/z: $[M+Na]^+$ Calcd for $C_{26}H_{20}N_2O_2SNa$ 447.1138; Found 447.1138.

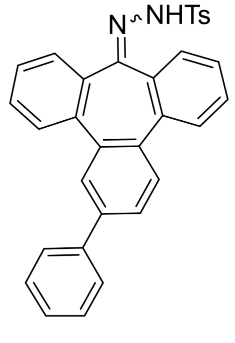

4-Methyl-N'-(2-phenyl-9H-tribenzo[a,c,e][7]annulen-9-ylidene)benzenesulfono-hydrazide (1b) was synthesized by following Procedure B. The crude material was purified by column chromatography (SiO_2, petroleum ether: EtOAc = 5:1) to provide **1b** as a white solid (375 mg, 75% yield).

^{1}H NMR (400 MHz, $CDCl_3$) δ 8.00 (d, *J* = 12.2 Hz, 1H), 7.74 – 7.63 (m, 5H), 7.59 (d, *J* = 7.9 Hz, 1H), 7.57 (d, *J* = 1.6 Hz, 1H), 7.55 (s, 1H), 7.53 – 7.48 (m, 2H), 7.46 (dd, *J* = 4.6, 2.7 Hz, 2H), 7.44 (d, *J* = 1.7 Hz, 1H), 7.43 – 7.34 (m, 4H), 7.23 (d, *J* = 7.9 Hz, 1H), 6.99 (dd, *J* = 8.1, 6.1 Hz, 2H), 2.12 (d, *J* = 10.0 Hz, 3H).

^{13}C NMR (150 MHz, $CDCl_3$) δ 155.0, 154.9, 143.7, 143.7, 140.7, 140.3, 140.3, 140.2, 139.9, 139.8, 137.7, 137.6, 137.2, 137.0, 136.7, 136.6, 136.2, 135.3, 134.8, 134.8, 133.9, 133.8, 131.3, 130.7, 130.5, 130.5, 130.4, 129.4, 129.2, 129.2, 128.9, 128.9, 128.8, 128.6, 128.5, 128.3, 128.3, 128.1, 128.0, 127.7, 127.3, 126.9, 126.9, 126.5, 126.2, 126.0, 126.0, 124.9, 124.9, 21.3, 21.2.

HRMS: (ESI) m/z: $[M+Na]^+$ Calcd for $C_{32}H_{24}N_2O_2SNa$ 523.1451; Found 523.1453.

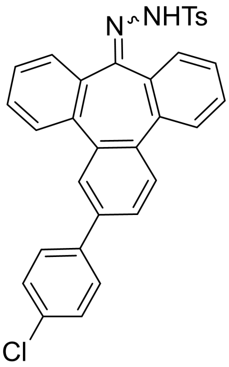

N'-(2-(4-chlorophenyl)-9H-tribenzo[a,c,e][7]annulen-9-ylidene)-4-methylben zenesulfonohydrazide (1c) was synthesized by following Procedure B. The crude material was purified by column chromatography (SiO_2, petroleum ether: EtOAc = 5:1) to provide **1c** as a white solid (401 mg, 75% yield).

^{1}H NMR (400 MHz, $CDCl_3$) δ 7.94 (d, *J* = 12.4 Hz, 1H), 7.74 – 7.66 (m, 2H), 7.60 (ddd, *J* = 12.5, 5.8, 2.6 Hz, 7H), 7.52 (d, *J* = 1.7 Hz, 1H), 7.49 – 7.46 (m, 2H), 7.46 – 7.39 (m, 4H), 7.25 (d, *J* = 4.5 Hz, 1H), 7.05 (dd, *J* = 8.0, 5.9 Hz, 2H), 2.20 (d, *J* = 8.6 Hz, 3H).

^{13}C NMR (100 MHz, $CDCl_3$) δ 154.7, 154.7, 143.8, 143.8, 140.4, 140.4, 139.6, 139.1, 138.5, 138.4, 138.0, 137.5, 137.2, 137.0, 136.9, 136.7, 136.5, 135.7, 135.0, 135.0, 134.0, 133.9, 131.5, 130.7, 130.7, 130.6, 130.5, 129.4, 129.4, 129.3, 129.3, 129.2,

129.1, 128.8, 128.7, 128.5, 128.4, 128.2, 128.2, 127.4, 126.4, 126.2, 126.1, 126.1, 125.0, 125.0, 21.4.

HRMS: (ESI) m/z: [M+Na]$^+$ Calcd for $C_{32}H_{23}ClN_2O_2SNa$ 557.1061; Found 557.1069.

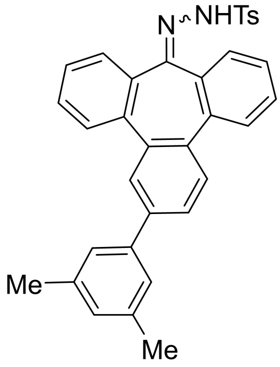

N'-(2-(3,5-dimethylphenyl)-9H-tribenzo[a,c,e][7]annulen-9-ylidene)-4-methyl benzenesulfonohydrazide (1d) was synthesized by following Procedure B. The crude material was purified by column chromatography (SiO_2, petroleum ether: EtOAc = 5:1) to provide **1d** as a white solid (370 mg, 70% yield).

^{1}H NMR (400 MHz, $CDCl_3$) δ 7.97 (d, *J* = 11.5 Hz, 1H), 7.76 – 7.69 (m, 2H), 7.65 (dd, *J* = 4.5, 1.8 Hz, 1H), 7.63 – 7.51 (m, 5H), 7.48 – 7.38 (m, 4H), 7.32 – 7.27 (m, 3H), 7.08 (d, *J* = 4.7 Hz, 1H), 7.04 (d, *J* = 3.2 Hz, 1H), 7.02 (d, *J* = 3.2 Hz, 1H), 2.44 (d, *J* = 5.1 Hz, 6H), 2.18 (d, *J* = 10.8 Hz, 3H).

^{13}C NMR (150 MHz, $CDCl_3$) δ 155.1, 155.0, 143.7, 141.1, 140.7, 140.4, 140.3, 140.0, 139.9, 138.5, 138.4, 137.7, 137.7, 137.4, 137.1, 136.7, 136.7, 136.1, 135.1, 134.9, 134.9, 133.9, 133.8, 131.2, 130.8, 130.6, 130.4, 130.4, 129.5, 129.4, 129.3, 128.9, 128.7, 128.6, 128.3, 128.3, 128.1, 128.0, 127.3, 126.6, 126.4, 126.1, 126.0, 125.0, 124.9, 124.9, 21.4, 21.4.

HRMS: (ESI) m/z: [M+Na]$^+$ Calcd for $C_{34}H_{28}N_2O_2SNa$ 551.1764; Found 551.1765.

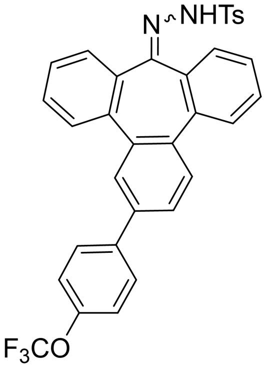

4-Methyl-N'-(2-(4-(trifluoromethoxy)phenyl)-9H-tribenzo[a,c,e][7]annulen-9-ylidene)benzenesulfonohydrazide (1e) was synthesized by following Procedure B. The crude material was purified by column chromatography (SiO_2, petroleum ether: EtOAc = 5:1) to provide **1e** as a yellow solid (397 mg, 68% yield).

^{1}H NMR (400 MHz, $CDCl_3$) δ 7.90 (d, J = 6.5 Hz, 1H), 7.59 – 7.55 (m, 2H), 7.54 – 7.42 (m, 7H), 7.35 (s, 1H), 7.33 – 7.26 (m, 4H), 7.21 (dd, J = 8.3, 5.2 Hz, 2H), 7.14 – 7.11 (m, 1H), 6.92 (t, J = 7.1 Hz, 2H), 2.05 (d, J = 8.6 Hz, 3H).

^{13}C NMR (150 MHz, $CDCl_3$) δ 155.1, 155.0, 143.7, 141.1, 140.7, 140.4, 140.3, 140.0, 139.9, 138.5, 138.4, 137.7, 137.7, 137.4, 137.1, 136.7, 136.7, 136.1, 135.1, 134.9, 134.9, 133.9, 133.8, 131.2, 130.8, 130.6, 130.4, 130.4, 129.5, 129.4, 129.3, 128.9, 128.7, 128.6, 128.3, 128.3, 128.1, 128.0, 127.3, 126.6, 126.4, 126.1, 126.0, 125.0, 124.9, 124.9, 118.8 (q, J = 257.3 Hz), 21.4, 21.4.

^{19}F NMR (565 MHz, $CDCl_3$) δ -57.64 (d, J = 11.0 Hz).

HRMS: (ESI) m/z: $[M+Na]^+$ Calcd for $C_{33}H_{23}F_3N_2O_3SNa$ 607.1274; Found 607.1276.

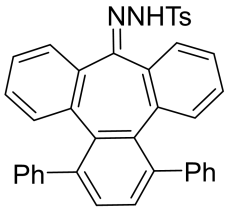

N'-(1,4-diphenyl-9H-tribenzo[a,c,e][7]annulen-9-ylidene)-4-methylbenzenesulfono hydrazide (1f) was synthesized by following Procedure B. The crude material was purified by column chromatography (SiO_2, petroleum ether: EtOAc = 5:1) to provide **1f** as a white solid (346 mg, 60% yield).

^{1}H NMR (400 MHz, $CDCl_3$) δ 8.12 (s, 1H), 7.64 (d, J = 8.0 Hz, 2H), 7.47 (d, J = 7.9 Hz, 1H), 7.39 (d, J = 7.8 Hz, 1H), 7.35 (d, J = 7.6 Hz, 1H), 7.31 – 7.06 (m, 11H), 6.84 (d, J = 8.9 Hz, 5H), 6.76 (s, 3H), 2.02 (s, 3H).

^{13}C NMR (100 MHz, $CDCl_3$) δ 156.5, 143.7, 142.3, 142.1, 141.9, 141.9, 141.1, 136.6, 136.1, 135.1, 134.8, 134.4, 134.2, 134.1, 133.1, 130.4, 129.7, 129.5, 129.4, 128.3, 128.1, 127.9, 127.6, 127.3, 127.2, 127.1, 126.5, 126.4, 124.8, 124.1, 21.3.

HRMS: (ESI) m/z: $[M+Na]^+$ Calcd for $C_{38}H_{28}N_2O_2SNa$ 599.1764; Found 599.1773.

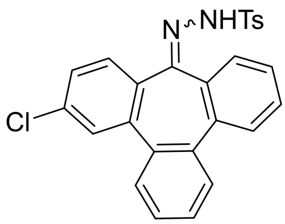

N'-(6-chloro-9H-tribenzo[a,c,e][7]annulen-9-ylidene)-4-methylbenzenesulfonohydrazide (1g) was synthesized by following Procedure B. The crude material was purified by column chromatography (SiO_2, petroleum ether: EtOAc = 5:1) to provide **1g** as a white solid (321 mg, 70% yield).

^{1}H NMR (400 MHz, $CDCl_3$) δ 7.92 (d, *J* = 36.1 Hz, 1H), 7.64 (d, *J* = 2.0 Hz, 1H), 7.57 (d, *J* = 6.1 Hz, 2H), 7.54 – 7.49 (m, 2H), 7.49 – 7.39 (m, 5H), 7.35 (s, 2H), 7.21 (dd, *J* = 18.5, 7.9 Hz, 1H), 7.07 (d, *J* = 7.9 Hz, 2H), 2.33 (s, 3H).

^{13}C NMR (100 MHz, $CDCl_3$) δ 153.7, 153.7, 143.8, 143.7, 140.1, 139.5, 138.8, 138.6, 137.4, 137.4, 136.7, 136.5, 136.2, 136.0, 135.1, 135.1, 134.7, 133.6, 132.2, 130.9, 130.8, 130.7, 130.6, 130.5, 130.2, 129.9, 129.4, 129.3, 128.9, 128.7, 128.5, 128.4, 128.2, 128.1, 127.9, 127.8, 127.5, 127.4, 127.4, 126.4, 126.1, 124.9, 21.5.

HRMS: (ESI) m/z: $[M+Na]^+$ Calcd for $C_{26}H_{19}ClN_2O_2SNa$ 481.0748; Found 481.0749.

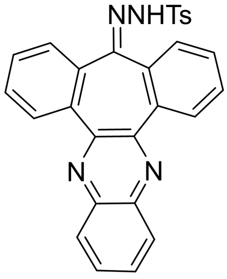

N'-(10H-dibenzo[3,4:6,7]cyclohepta[1,2-b]quinoxalin-10-ylidene)-4-methylbenzenesulfonohydrazide (1h) was synthesized by following Procedure B. The crude material was purified by column chromatography (SiO_2, petroleum ether: EtOAc = 5:1) to provide **1h** as a yellow solid (324 mg, 68% yield).

^{1}H NMR (400 MHz, $CDCl_3$) δ 8.20 – 8.04 (m, 4H), 7.92 (d, *J* = 8.4 Hz, 1H), 7.72 (t, *J* = 7.6 Hz, 1H), 7.64 (d, *J* = 6.3 Hz, 2H), 7.61 – 7.47 (m, 5H), 7.39 (d, *J* = 8.0 Hz, 2H), 6.72 (d, *J* = 8.0 Hz, 2H), 1.87 (s, 3H).

^{13}C NMR (150 MHz, $CDCl_3$) δ 153.2, 150.4, 149.6, 143.5, 141.3, 140.8, 140.5, 135.4, 135.0, 134.4, 134.0, 132.3, 130.6, 130.4, 130.3, 130.3, 129.5, 129.5, 129.1, 129.0, 128.8, 127.4, 126.8, 126.2, 125.7, 20.9.

HRMS: (ESI) m/z: $[M+Na]^+$ Calcd for $C_{28}H_{20}N_4O_2SNa$ 499.1199; Found 499.1204.

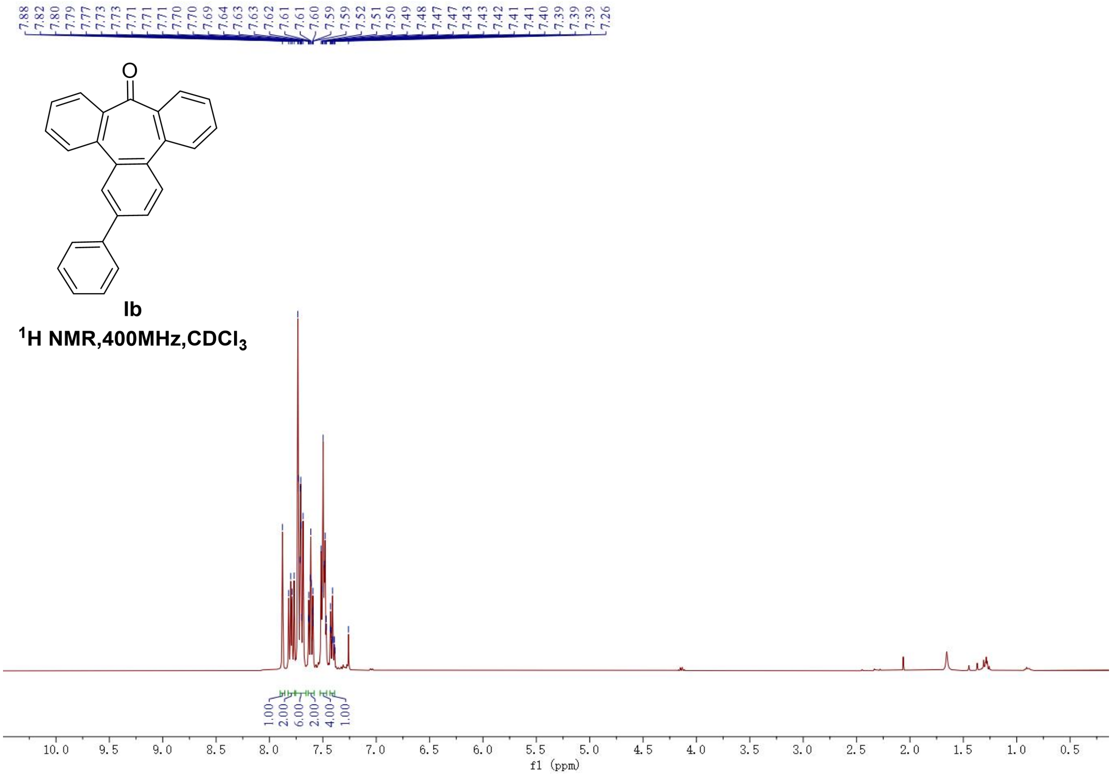
7.88 7.82 7.80 7.79 7.77 7.73 7.73 7.71 7.71 7.71 7.70 7.70 7.69 7.64 7.63 7.63 7.62 7.61 7.61 7.60 7.59 7.59 7.52 7.51 7.50 7.49 7.48 7.47 7.47 7.43 7.43 7.42 7.41 7.40 7.39 7.39 7.39 7.26
O
Ib
1H NMR,400MHz,CDCl3
1.00 2.00 6.00 2.00 4.00 1.00
10.0 9.5 9.0 8.5 8.0 7.5 7.0 6.5 6.0 5.5 5.0 4.5 4.0 3.5 3.0 2.5 2.0 1.5 1.0 0.5
f1 (ppm)

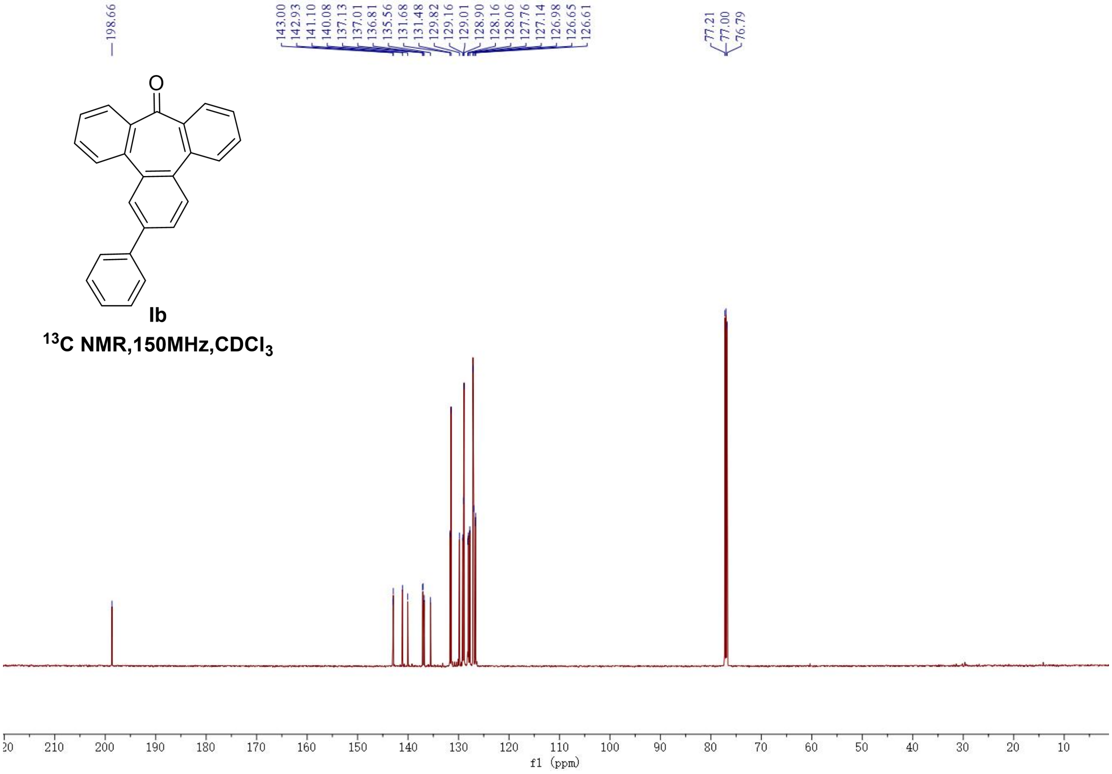
198.66
143.00 142.93 141.10 140.08 137.13 137.01 136.81 135.56 131.68 131.48 129.82 129.16 129.01 128.90 128.16 128.06 127.76 127.14 126.98 126.65 126.61
77.21 77.00 76.79
O
Ib
13C NMR,150MHz,CDCl3
20 210 200 190 180 170 160 150 140 130 120 110 100 90 80 70 60 50 40 30 20 10
f1 (ppm)

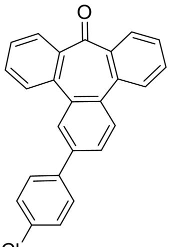

Ic

^{1}H NMR,400MHz,$CDCl_3$

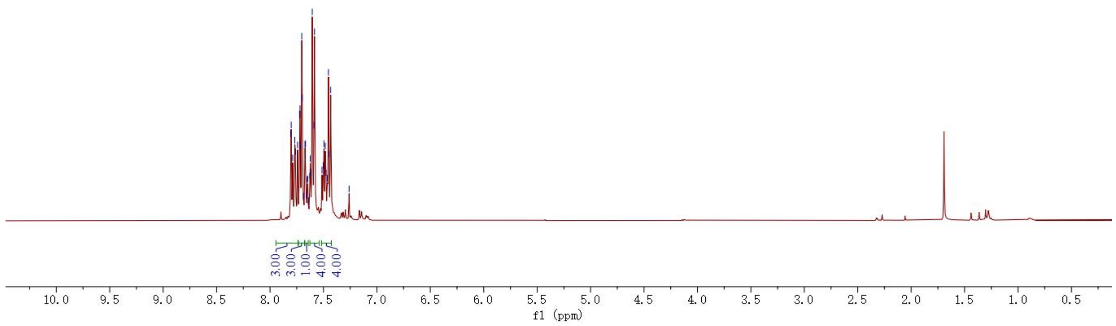

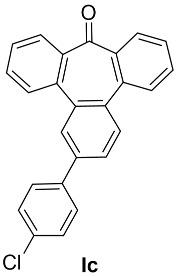

Ic

^{13}C NMR,150MHz,$CDCl_3$

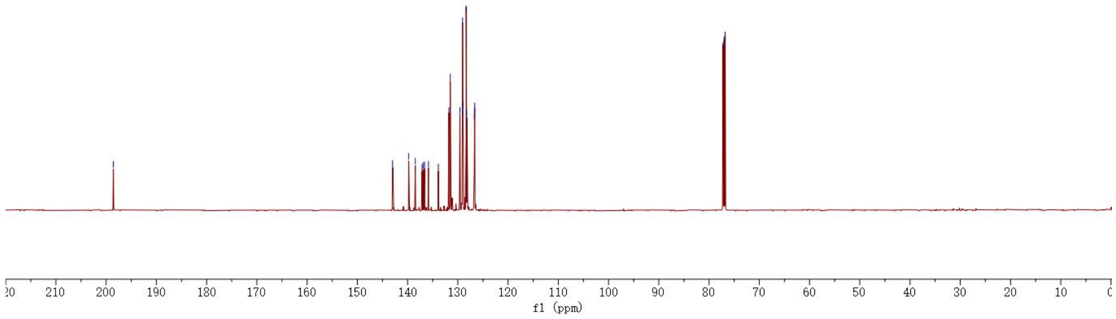

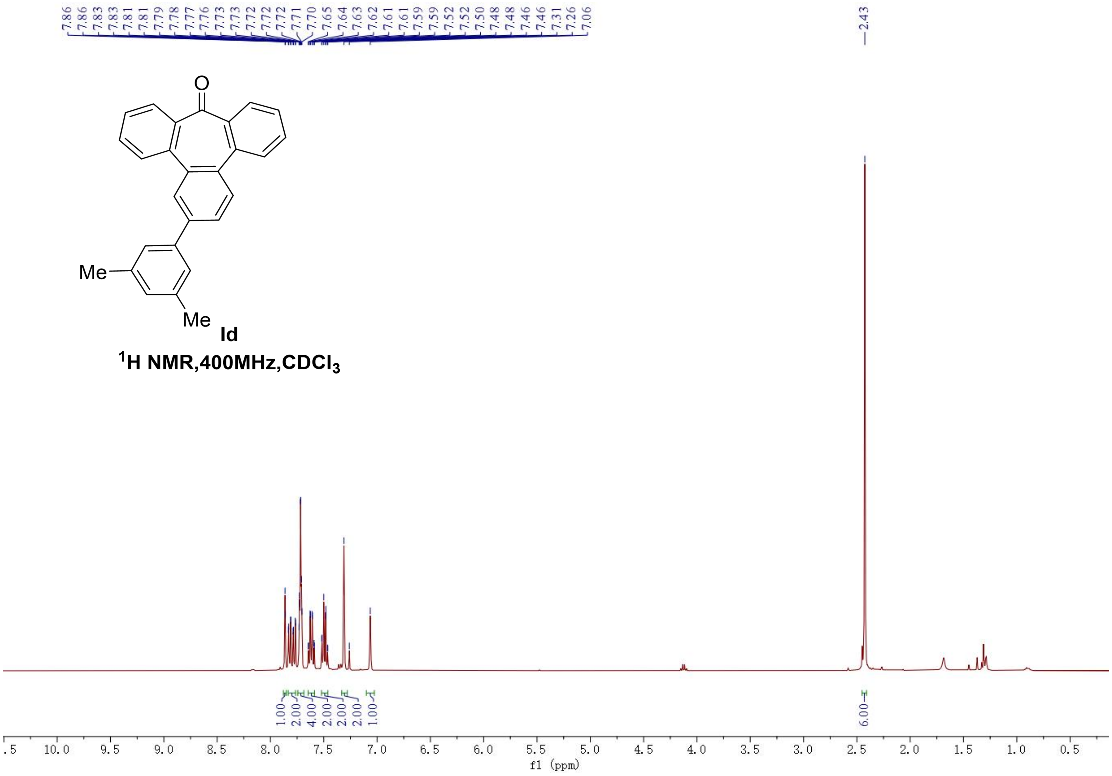
7.86 7.86 7.83 7.83 7.81 7.81 7.79 7.78 7.77 7.76 7.73 7.73 7.72 7.72 7.72 7.71 7.70 7.65 7.64 7.63 7.62 7.61 7.61 7.59 7.59 7.52 7.52 7.50 7.48 7.48 7.46 7.46 7.31 7.26 7.06
2.43
O
Me
Me
Id
^{1}H NMR,400MHz,CDCl$_3$
1.00 2.00 4.00 2.00 2.00 2.00 1.00
6.00
f1 (ppm)

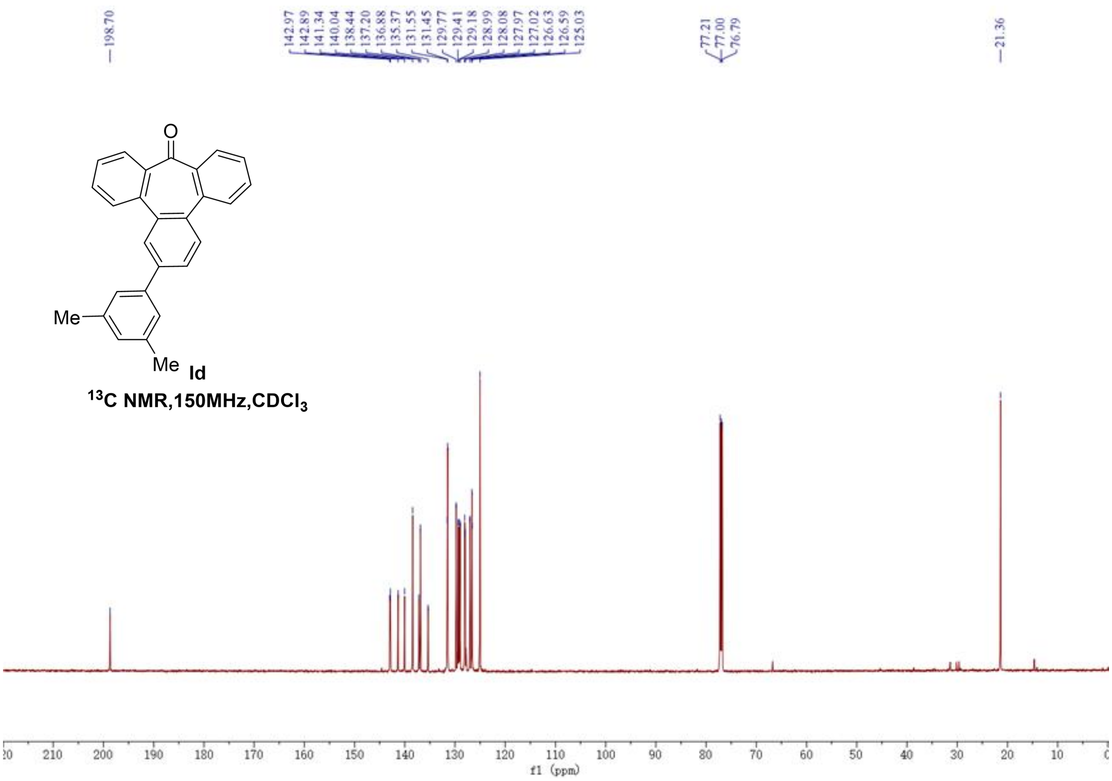
198.70
142.97 142.89 141.34 140.04 138.44 137.20 136.88 135.37 131.55 131.45 129.77 129.41 129.18 128.99 128.08 127.97 127.02 126.63 126.59 125.03
77.21 77.00 76.79
21.36
O
Me
Me
Id
^{13}C NMR,150MHz,CDCl$_3$
f1 (ppm)

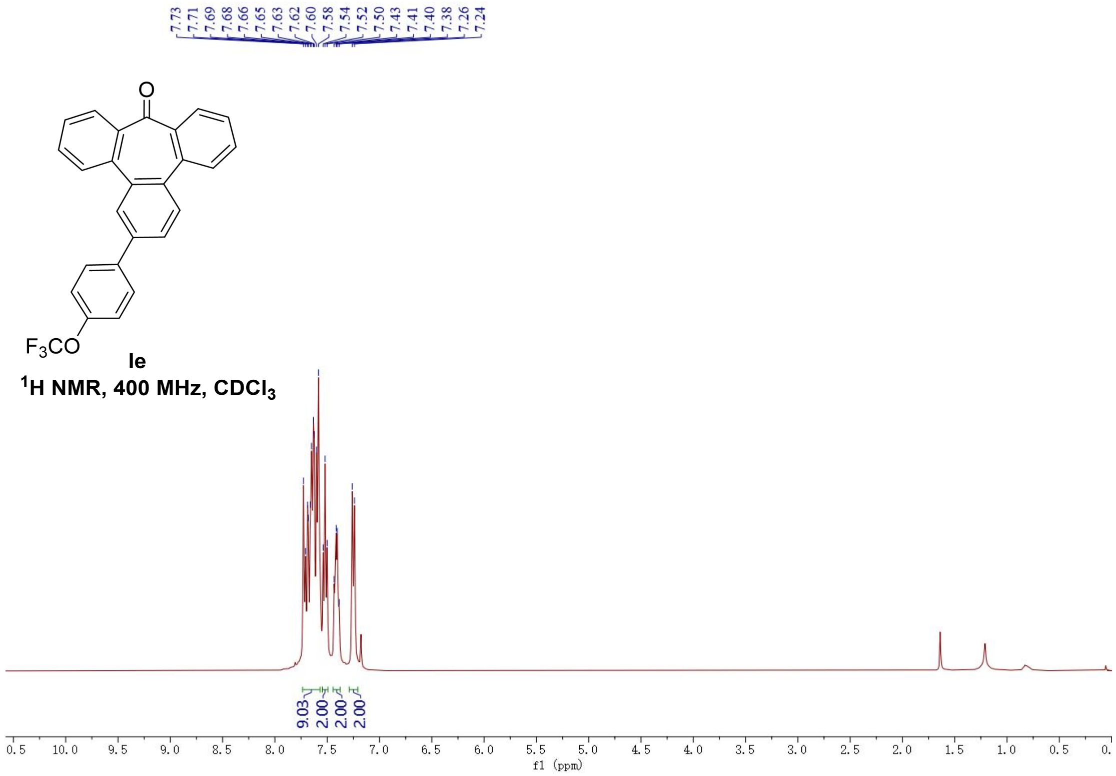

Ie

^{1}H NMR, 400 MHz, CDCl$_3$

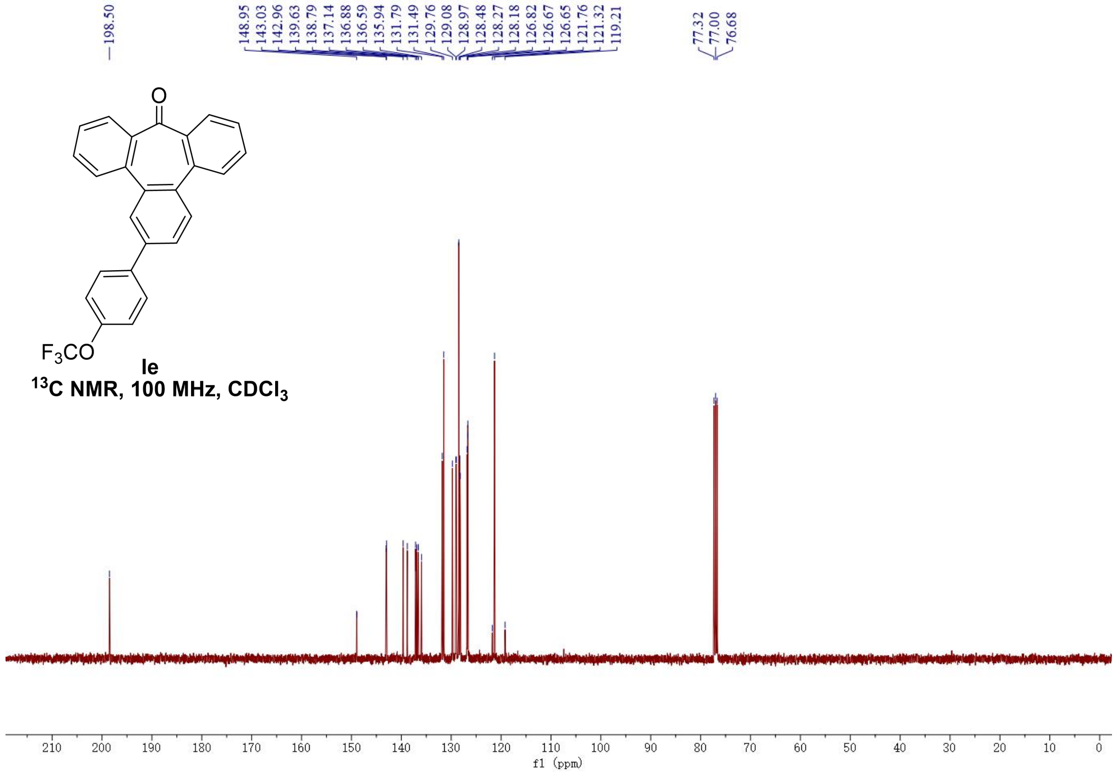

Ie

^{13}C NMR, 100 MHz, CDCl$_3$

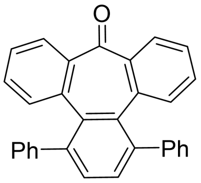

If
^{1}H NMR, 400 MHz, CDCl$_3$

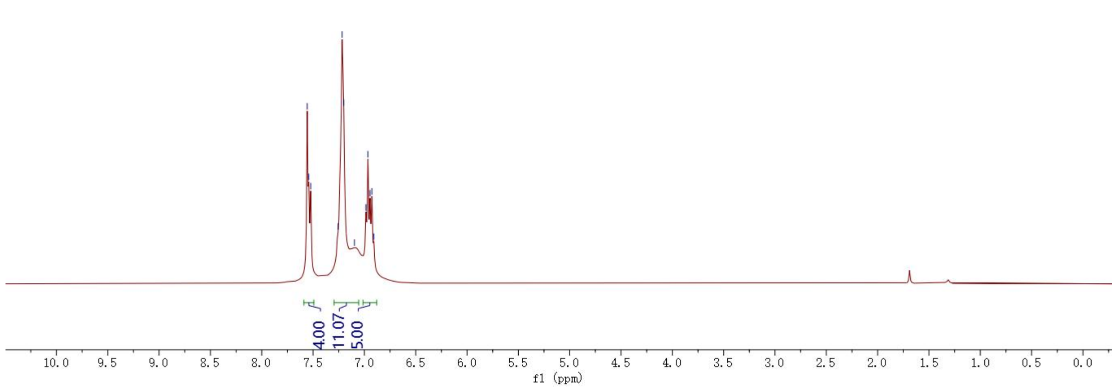

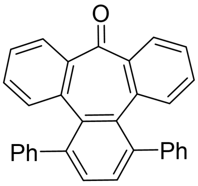

If
^{13}C NMR, 100 MHz, CDCl$_3$

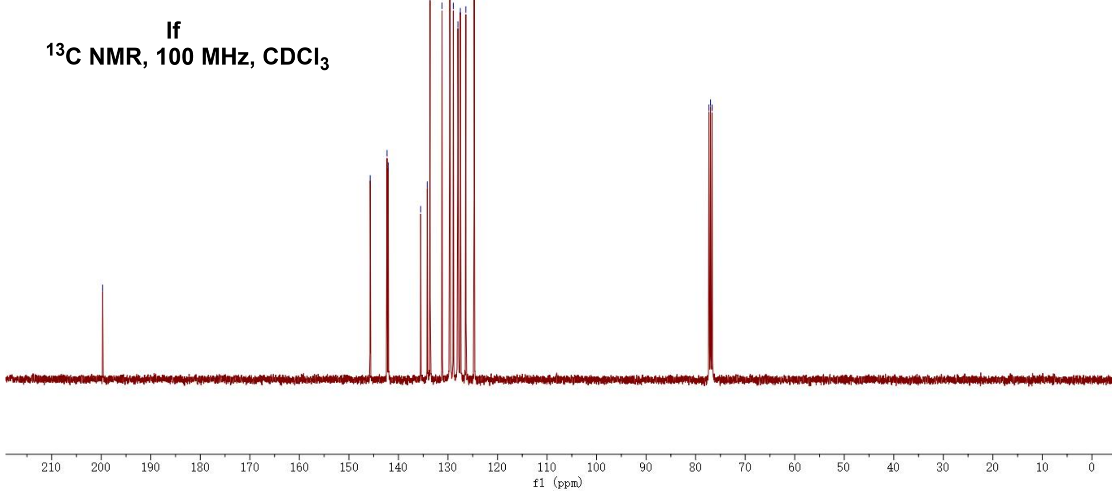

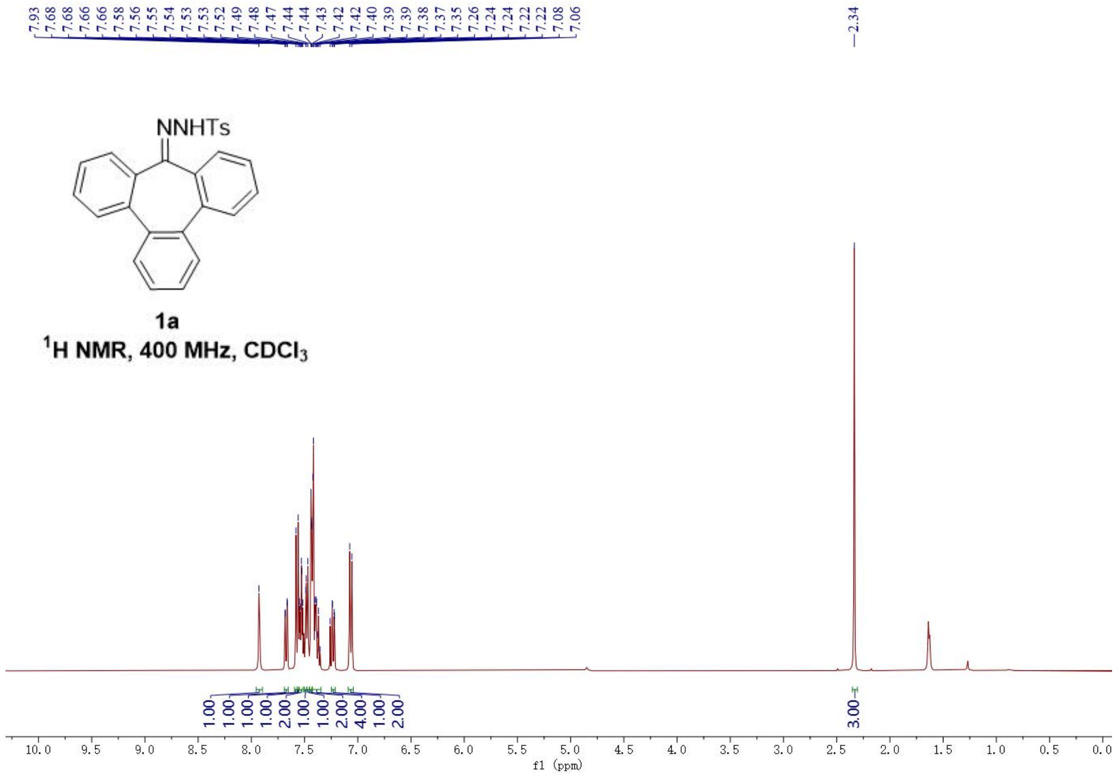
7.93
7.68
7.68
7.66
7.66
7.58
7.56
7.55
7.54
7.53
7.53
7.52
7.49
7.48
7.47
7.44
7.44
7.43
7.42
7.42
7.40
7.39
7.39
7.38
7.37
7.35
7.26
7.24
7.24
7.22
7.22
7.08
7.06
2.34
NNHTs
1a
^{1}H NMR, 400 MHz, CDCl$_3$
1.00
1.00
1.00
1.00
2.00
1.00
1.00
1.00
2.00
4.00
1.00
2.00
3.00
10.0 9.5 9.0 8.5 8.0 7.5 7.0 6.5 6.0 5.5 5.0 4.5 4.0 3.5 3.0 2.5 2.0 1.5 1.0 0.5 0.0
f1 (ppm)

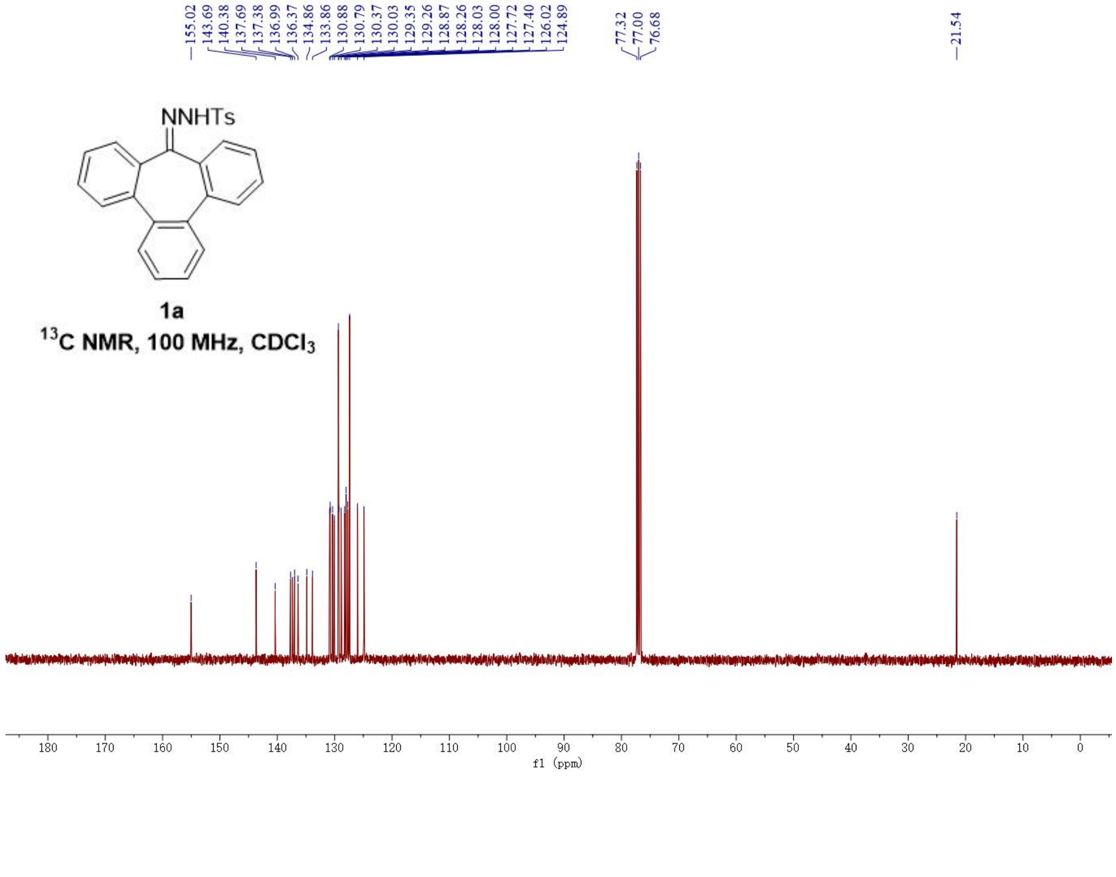
155.02
143.69
140.38
137.69
137.38
136.99
136.37
134.86
133.86
130.88
130.79
130.37
130.03
129.35
129.26
128.87
128.26
128.03
128.00
127.72
127.40
126.02
124.89
77.32
77.00
76.68
21.54
NNHTs
1a
^{13}C NMR, 100 MHz, CDCl$_3$
180 170 160 150 140 130 120 110 100 90 80 70 60 50 40 30 20 10 0
f1 (ppm)

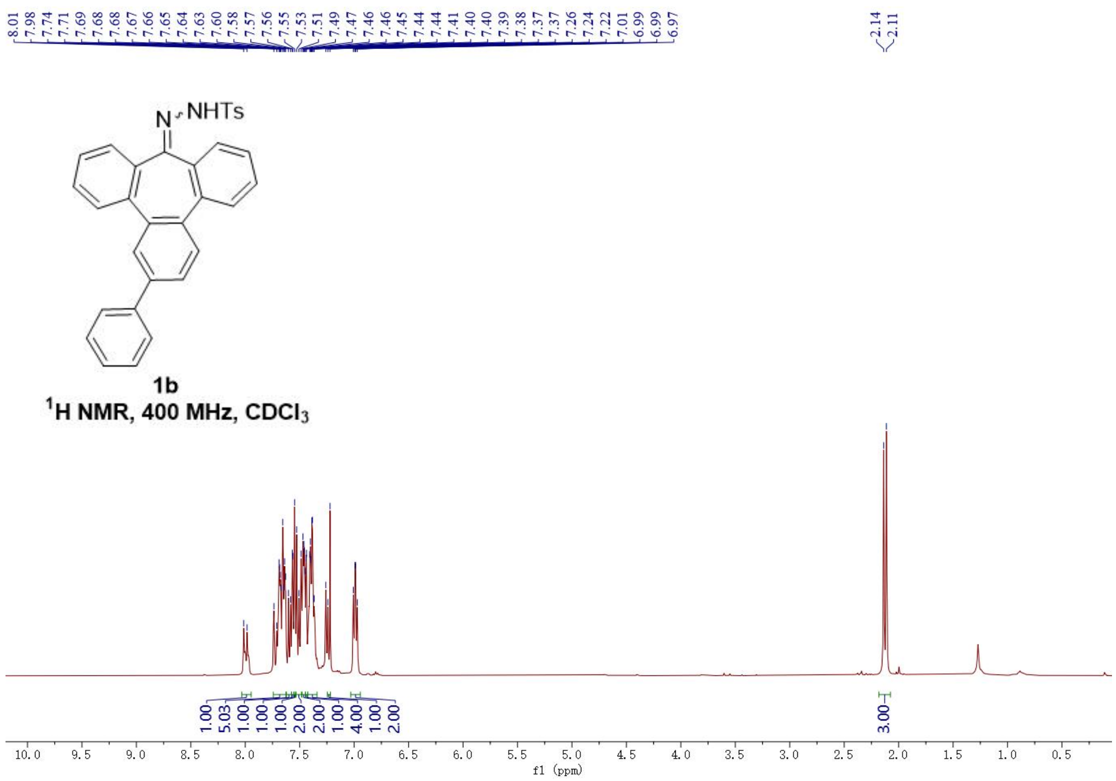
8.01 7.98 7.74 7.71 7.69 7.68 7.67 7.66 7.65 7.64 7.63 7.60 7.58 7.57 7.56 7.55 7.53 7.51 7.49 7.47 7.46 7.45 7.44 7.44 7.41 7.40 7.39 7.38 7.37 7.37 7.26 7.24 7.22 7.01 6.99 6.97
2.14 2.11
N NHTs
1b
1H NMR, 400 MHz, CDCl3
1.00 5.03 1.00 1.00 2.00 2.00 1.00 4.00 1.00 2.00
3.00
10.0 9.5 9.0 8.5 8.0 7.5 7.0 6.5 6.0 5.5 5.0 4.5 4.0 3.5 3.0 2.5 2.0 1.5 1.0 0.5
f1 (ppm)

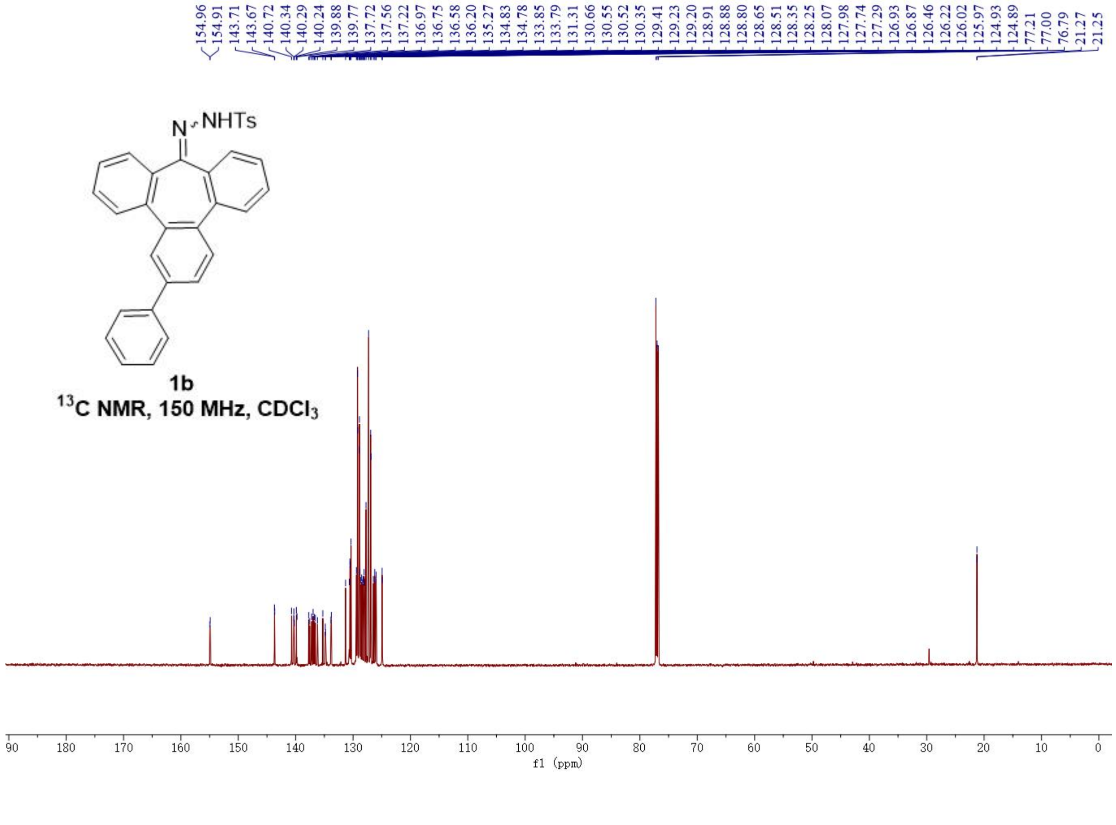
154.96 154.91 143.67 140.72 140.34 140.29 140.24 139.88 139.77 137.72 137.56 137.22 136.97 136.75 136.58 136.20 135.27 134.83 134.78 133.85 133.79 131.31 130.66 130.55 130.52 130.35 129.41 129.23 129.20 128.91 128.88 128.80 128.65 128.51 128.35 128.25 128.07 127.98 127.74 127.29 126.93 126.87 126.46 126.22 126.02 125.97 124.93 124.89 77.21 77.00 76.79 21.27 21.25
N NHTs
1b
13C NMR, 150 MHz, CDCl3
90 180 170 160 150 140 130 120 110 100 90 80 70 60 50 40 30 20 10 0
f1 (ppm)

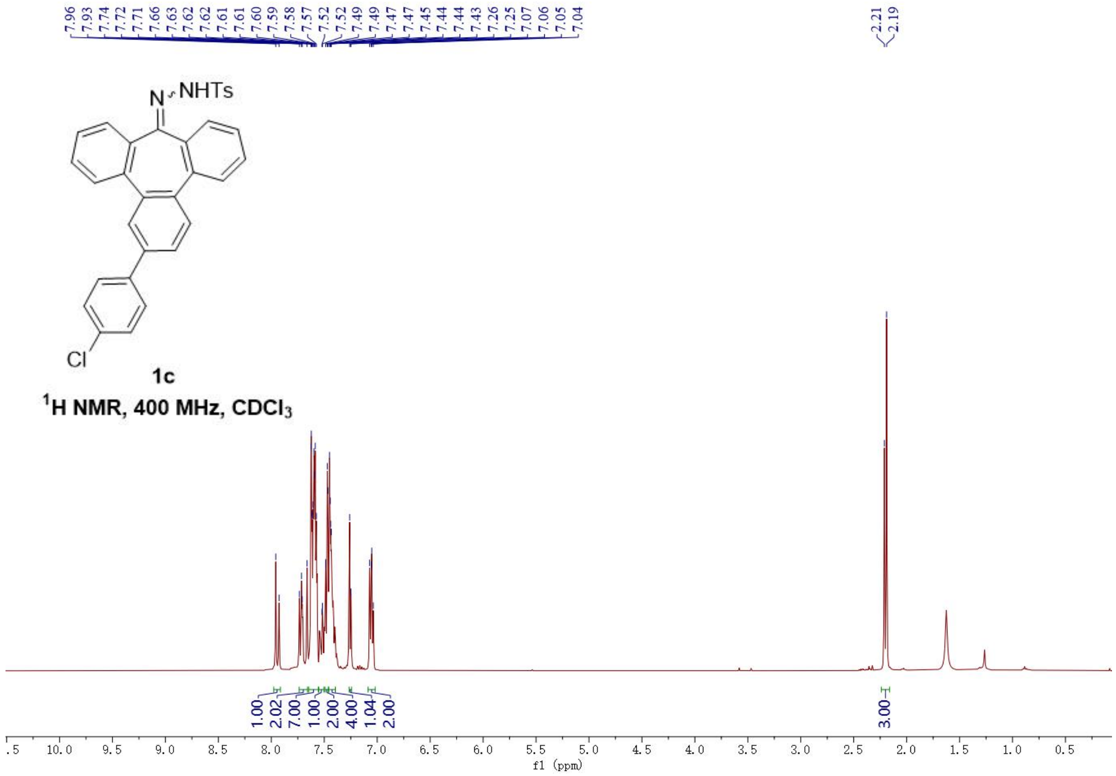
7.96 7.93 7.74 7.72 7.71 7.66 7.63 7.62 7.62 7.61 7.61 7.60 7.59 7.58 7.57 7.52 7.52 7.49 7.49 7.47 7.45 7.44 7.44 7.43 7.26 7.25 7.07 7.06 7.05 7.04
2.21 2.19
N NHTs
Cl
1c
^{1}H NMR, 400 MHz, $CDCl_3$
1.00 2.02 7.00 1.00 2.00 4.00 1.04 2.00
3.00
f1 (ppm)

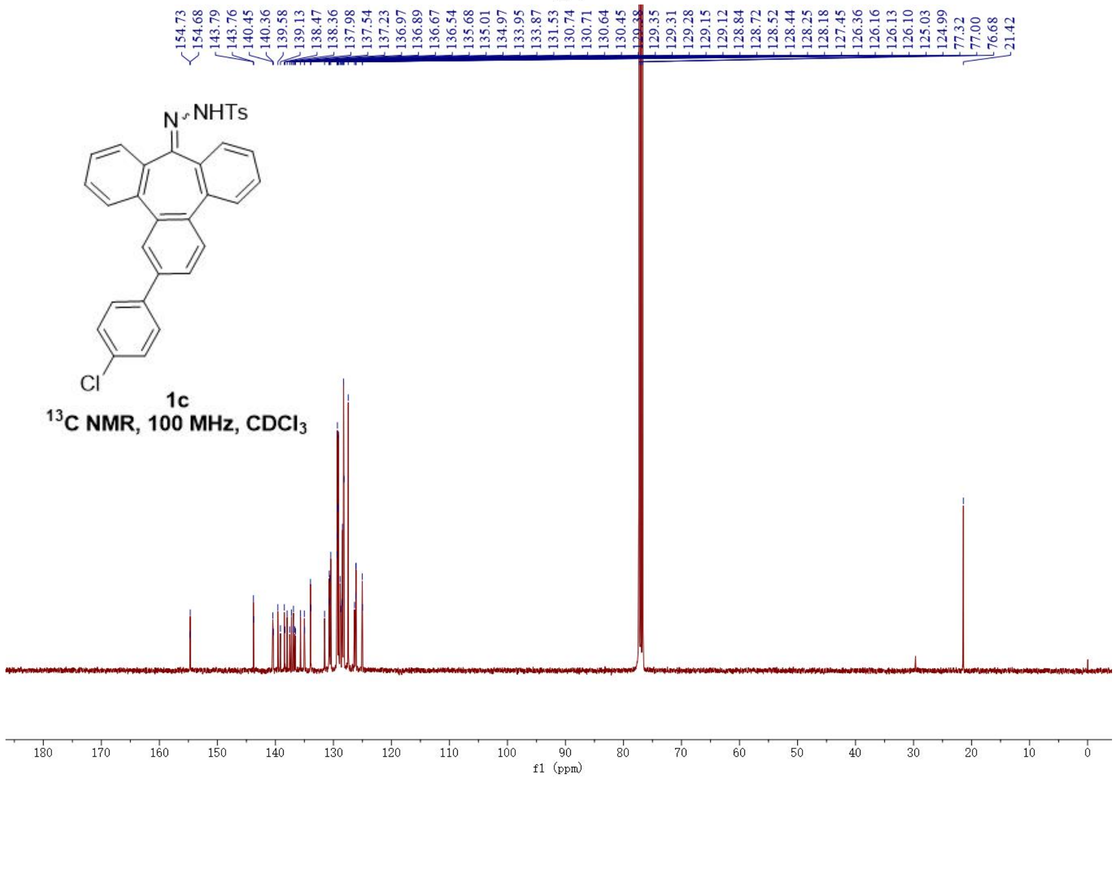
154.73 154.68 143.79 143.76 140.45 140.36 139.58 139.13 138.47 138.36 137.98 137.54 137.23 136.97 136.89 136.67 136.54 135.68 135.01 134.97 133.95 133.87 131.53 130.74 130.71 130.64 130.45 129.38 129.35 129.31 129.28 129.15 129.12 128.84 128.72 128.52 128.44 128.25 128.18 127.45 126.36 126.16 126.13 126.10 125.03 124.99 77.32 77.00 76.68 21.42
N NHTs
Cl
1c
^{13}C NMR, 100 MHz, $CDCl_3$
f1 (ppm)

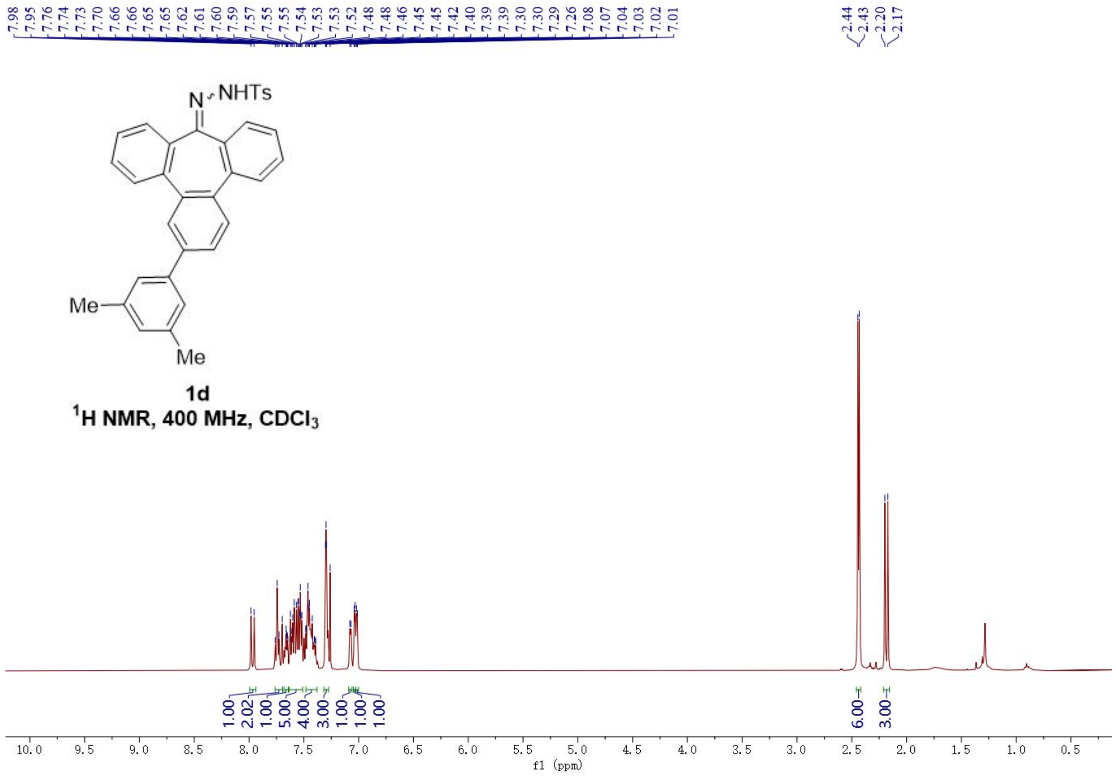
7.98 7.95 7.76 7.74 7.73 7.70 7.66 7.66 7.65 7.65 7.62 7.61 7.60 7.59 7.57 7.55 7.55 7.54 7.53 7.53 7.52 7.48 7.46 7.45 7.42 7.40 7.39 7.39 7.30 7.30 7.29 7.26 7.08 7.07 7.04 7.03 7.02 7.01
2.44 2.43 2.20 2.17
N NHTs
Me
Me
1d
^{1}H NMR, 400 MHz, CDCl$_3$
1.00 2.02 1.00 5.00 4.00 3.00 1.00 1.00 1.00
6.00 3.00
f1 (ppm)

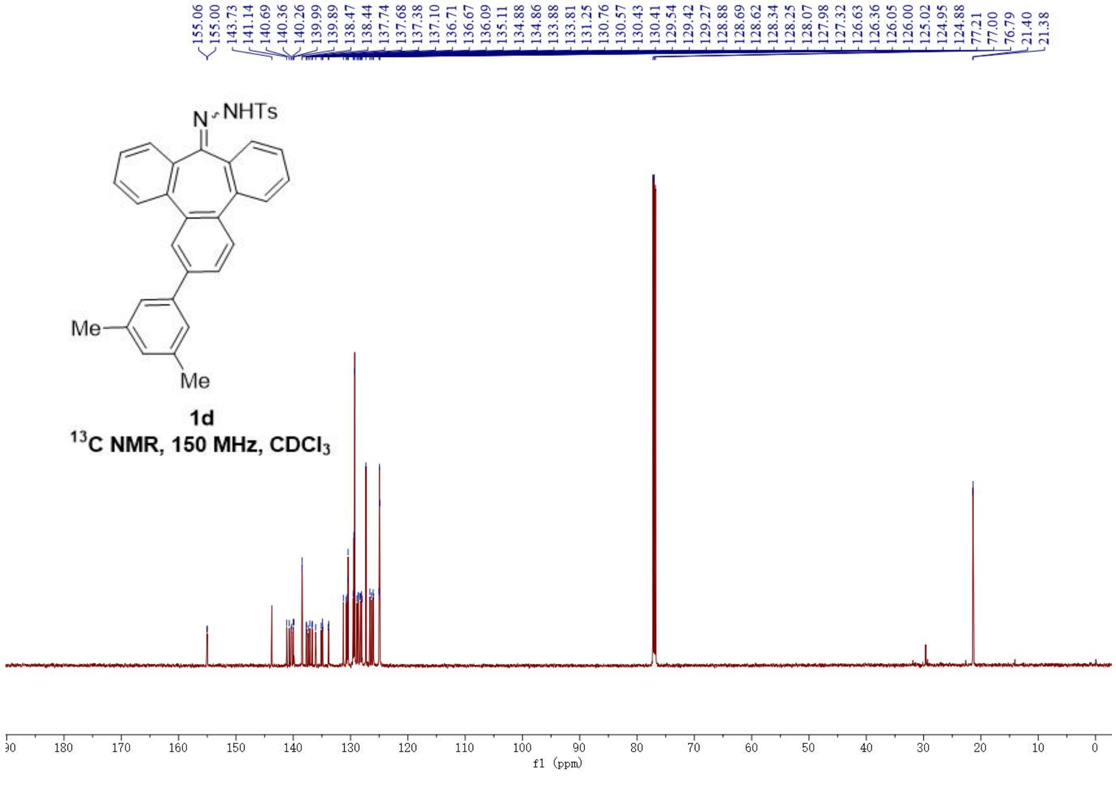
155.06 155.00 143.73 141.14 140.69 140.36 140.26 139.99 139.89 138.47 138.44 137.74 137.68 137.38 137.10 136.71 136.67 136.09 135.11 134.88 134.86 133.88 133.81 131.25 130.76 130.57 130.43 130.41 129.54 129.42 129.27 128.88 128.69 128.62 128.34 128.25 128.07 127.98 127.32 126.63 126.36 126.05 126.00 125.02 124.95 124.88 77.21 77.00 76.79 21.40 21.38
N NHTs
Me
Me
1d
^{13}C NMR, 150 MHz, CDCl$_3$
f1 (ppm)

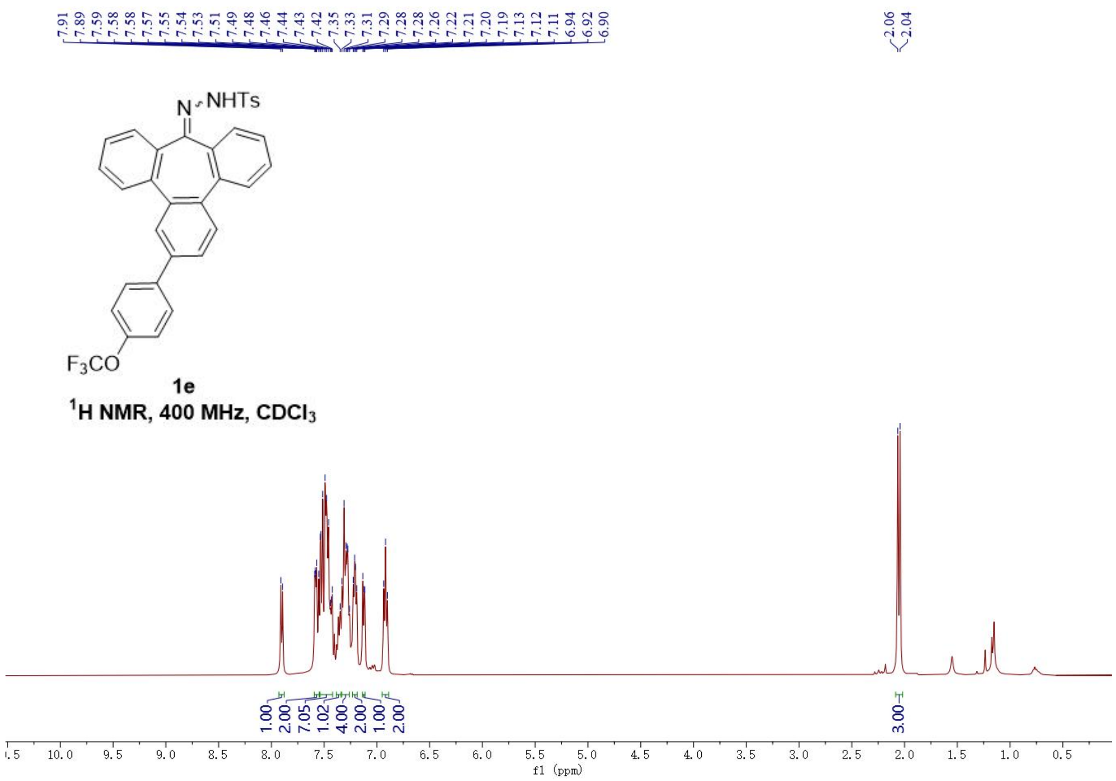
7.91 7.89 7.59 7.58 7.58 7.57 7.55 7.54 7.53 7.51 7.49 7.48 7.46 7.44 7.43 7.42 7.35 7.33 7.31 7.29 7.28 7.26 7.22 7.21 7.20 7.19 7.13 7.12 7.11 6.94 6.92 6.90
2.06 2.04
N-NHTs
F_3CO
1e
^{1}H NMR, 400 MHz, $CDCl_3$
1.00 2.00 7.05 1.02 4.00 2.00 1.00 2.00
3.00
f1 (ppm)

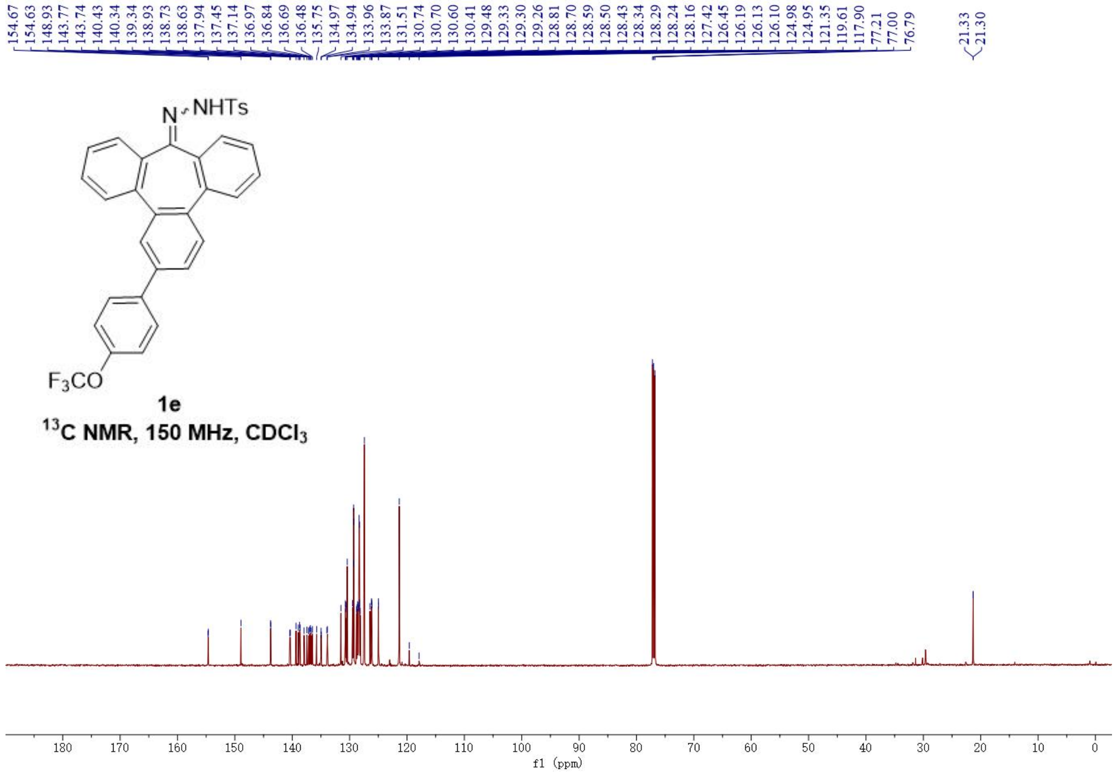
154.67 154.63 148.93 143.77 143.74 140.43 140.34 139.34 138.93 138.73 138.63 137.94 137.45 137.14 136.97 136.84 136.69 136.48 135.75 134.97 134.94 133.96 133.87 131.51 130.74 130.70 130.60 130.41 129.48 129.33 129.30 129.26 128.81 128.70 128.59 128.50 128.43 128.34 128.29 128.24 128.16 127.42 126.45 126.19 126.13 126.10 124.98 124.95 121.35 119.61 117.90 77.21 77.00 76.79
21.33 21.30
N-NHTs
F_3CO
1e
^{13}C NMR, 150 MHz, $CDCl_3$
f1 (ppm)

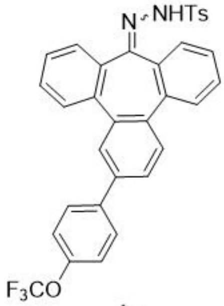

1e

^{19}F NMR, 565 MHz, CDCl$_3$

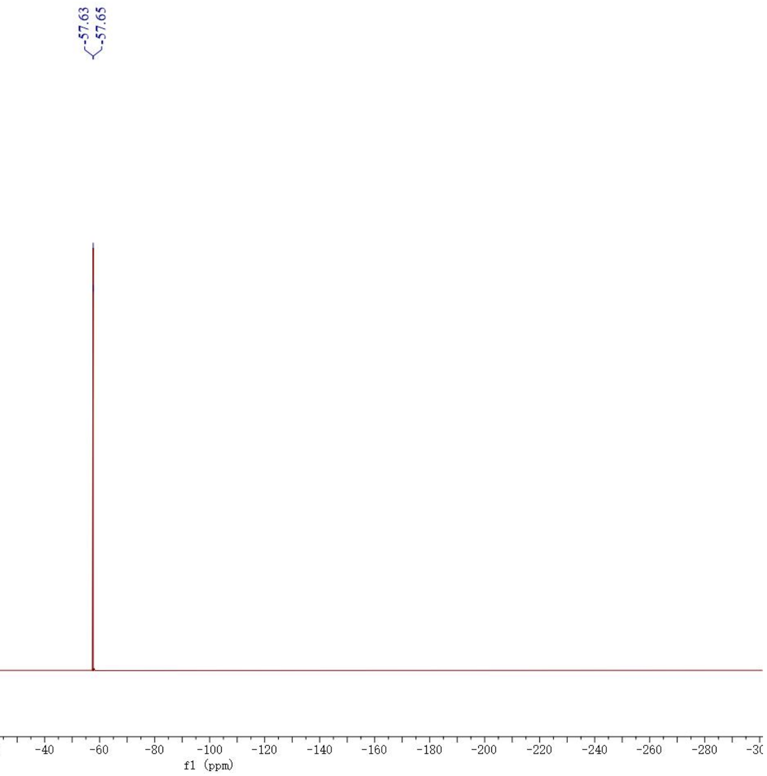

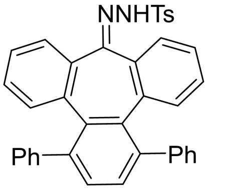

1f

^{1}H NMR, 400 MHz, CDCl$_3$

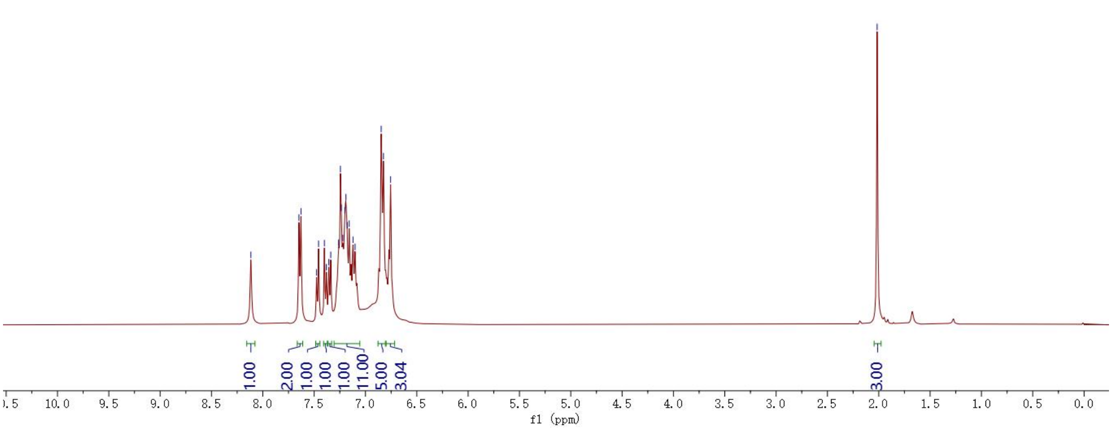

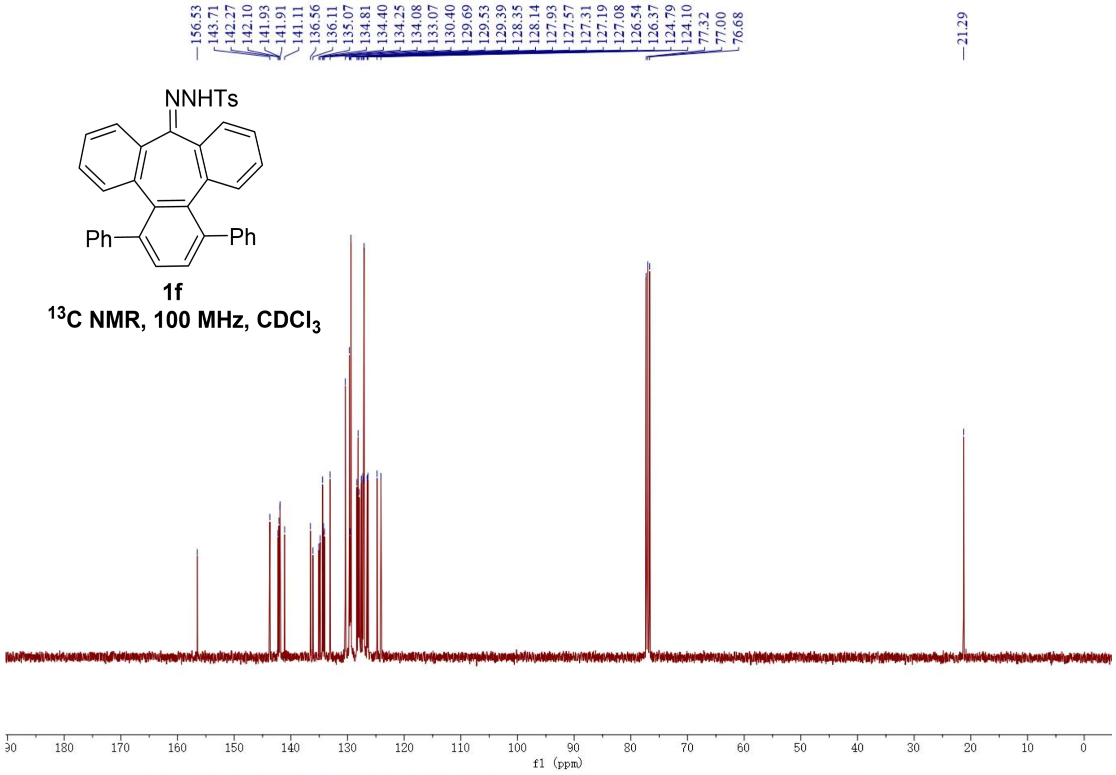
156.53
143.71
142.27
142.10
141.93
141.91
141.11
136.56
136.11
135.07
134.81
134.40
134.25
134.08
133.07
130.40
129.69
129.53
129.39
128.35
128.14
127.93
127.57
127.31
127.19
127.08
126.54
126.37
124.79
124.10
77.32
77.00
76.68
21.29
NNHTs
Ph
Ph
1f
13C NMR, 100 MHz, CDCl3
f1 (ppm)

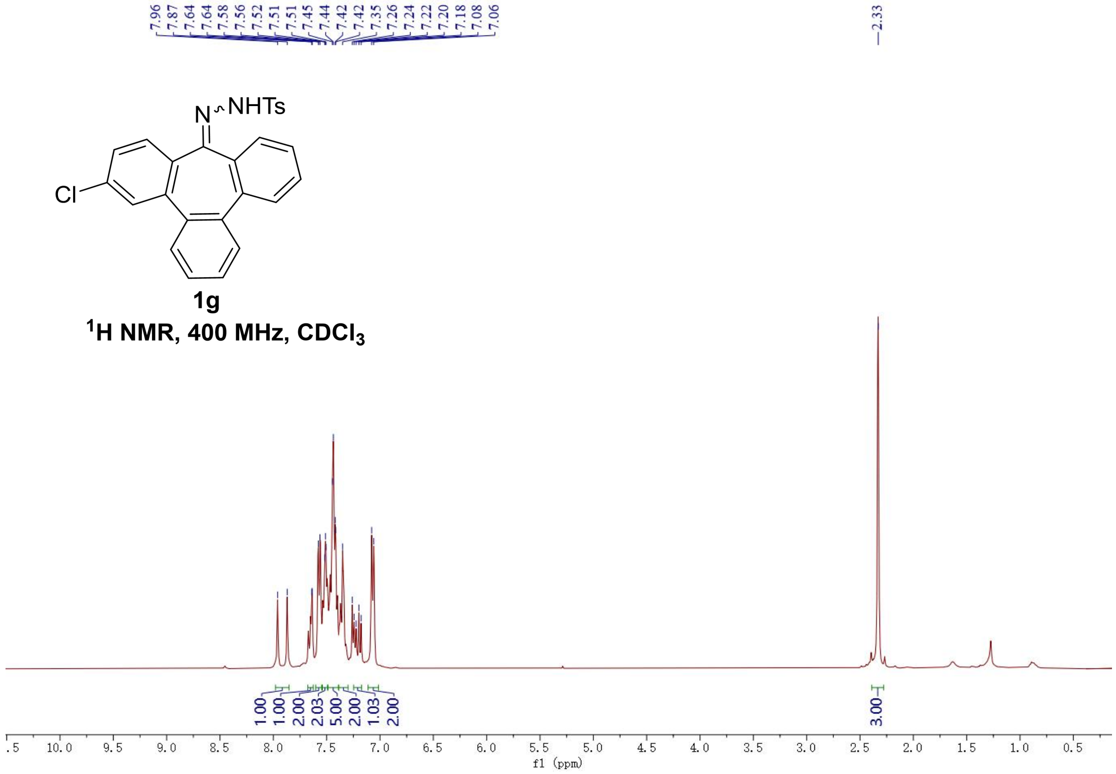
7.96
7.87
7.64
7.64
7.58
7.56
7.52
7.51
7.51
7.45
7.44
7.42
7.42
7.35
7.26
7.24
7.22
7.20
7.18
7.08
7.06
2.33
N
NHTs
Cl
1g
1H NMR, 400 MHz, CDCl3
1.00
1.00
2.00
2.03
5.00
2.00
1.03
2.00
3.00
f1 (ppm)

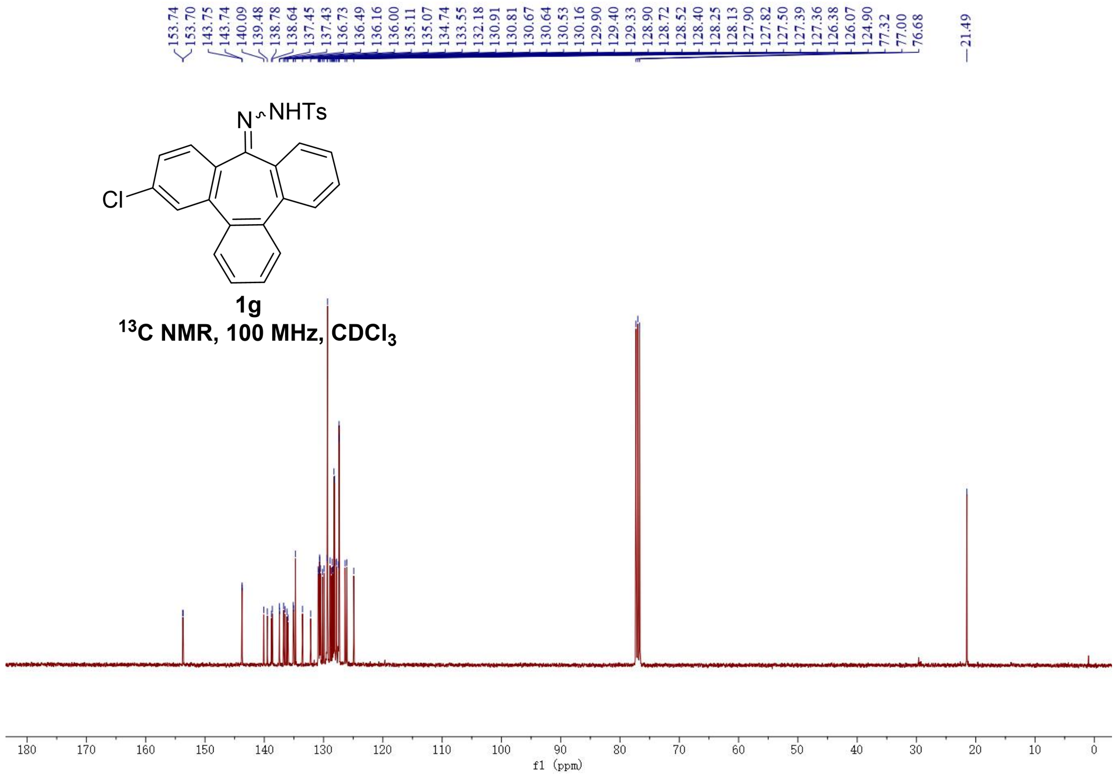
153.74
153.70
143.75
143.74
140.09
139.48
138.78
138.64
137.45
137.43
136.73
136.49
136.16
136.00
135.11
135.07
134.74
133.55
132.18
130.91
130.81
130.67
130.64
130.53
130.16
129.90
129.40
129.33
128.90
128.72
128.52
128.40
128.25
128.13
127.90
127.82
127.50
127.39
127.36
126.38
126.07
124.90
77.32
77.00
76.68
21.49
NHTs
N
Cl
1g
13C NMR, 100 MHz, CDCl3
f1 (ppm)

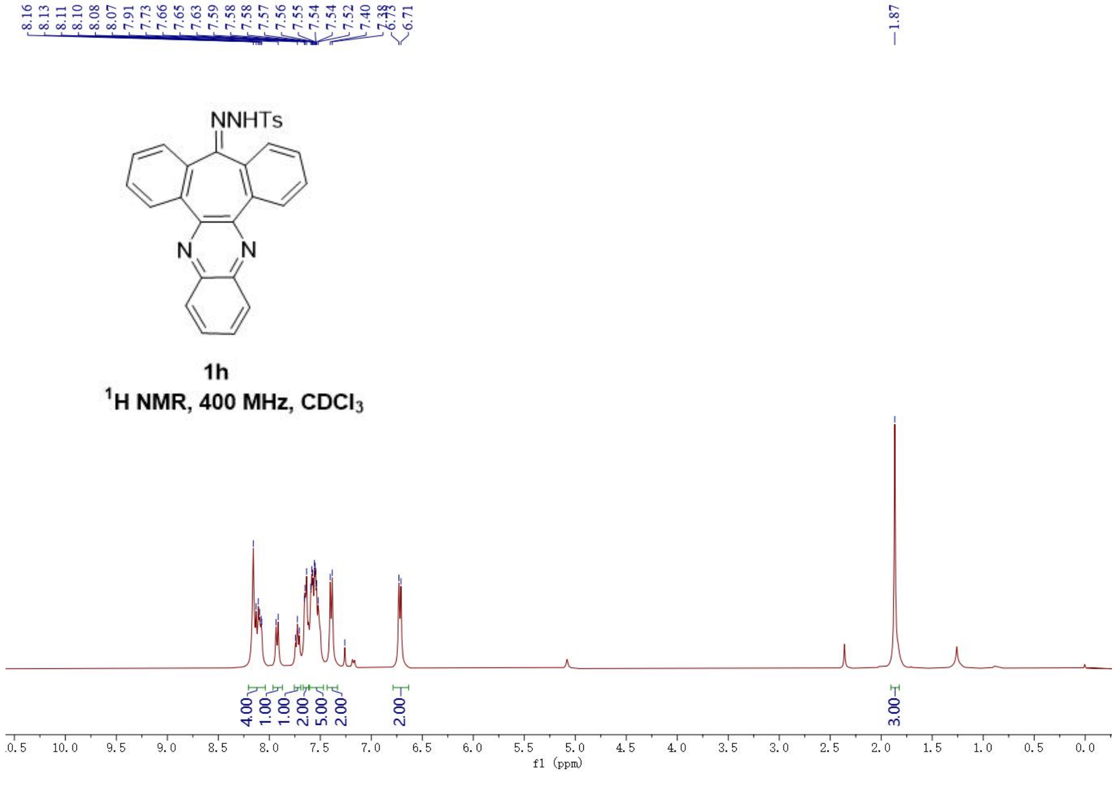
8.16
8.13
8.11
8.10
8.08
8.07
7.91
7.73
7.66
7.65
7.63
7.59
7.58
7.57
7.56
7.55
7.54
7.54
7.52
7.40
7.38
6.73
6.71
1.87
NNHTs
N
N
1h
1H NMR, 400 MHz, CDCl3
4.00
1.00
1.00
2.00
5.00
2.00
2.00
3.00
f1 (ppm)

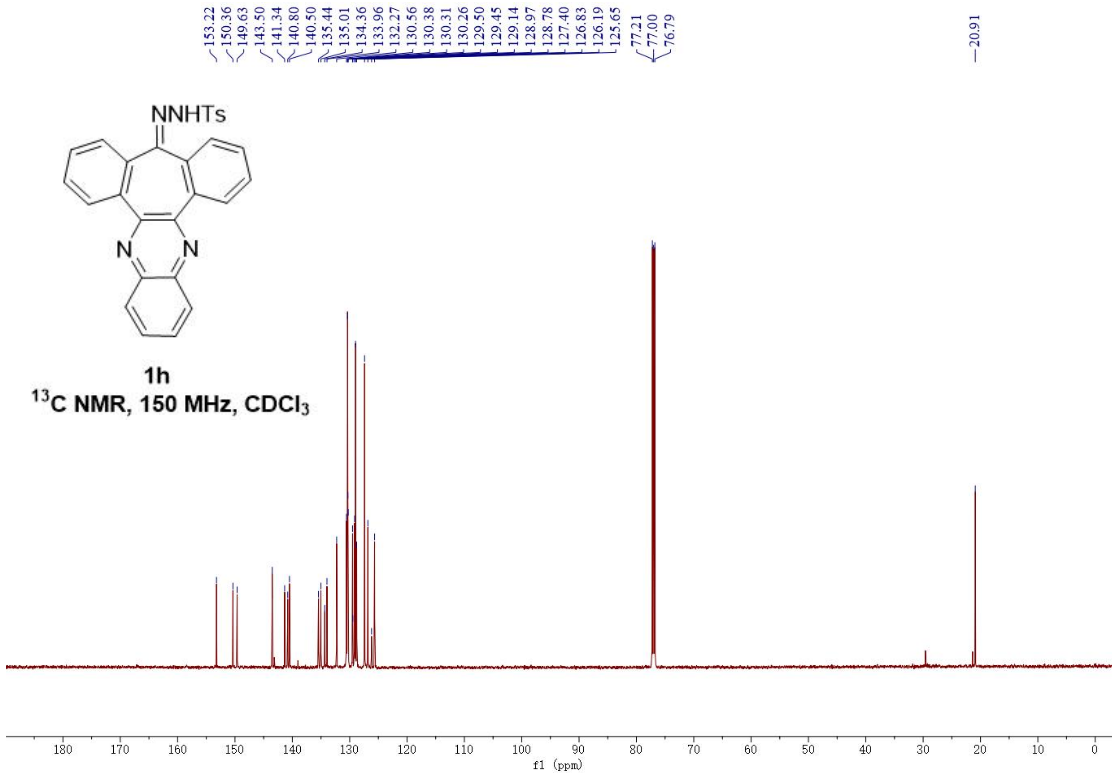
153.22
150.36
149.63
143.50
141.34
140.80
140.50
135.44
135.01
134.36
133.96
132.27
130.56
130.38
130.31
130.26
129.50
129.45
129.14
128.97
128.78
127.40
126.83
126.19
125.65
77.21
77.00
76.79
20.91
NNHTs
N
N
1h
^{13}C NMR, 150 MHz, $CDCl_3$
180 170 160 150 140 130 120 110 100 90 80 70 60 50 40 30 20 10 0
f1 (ppm)

4. Optimization of Reaction Conditions

Supplementary Table 1. Optimization of the catalyst and ligand[a]

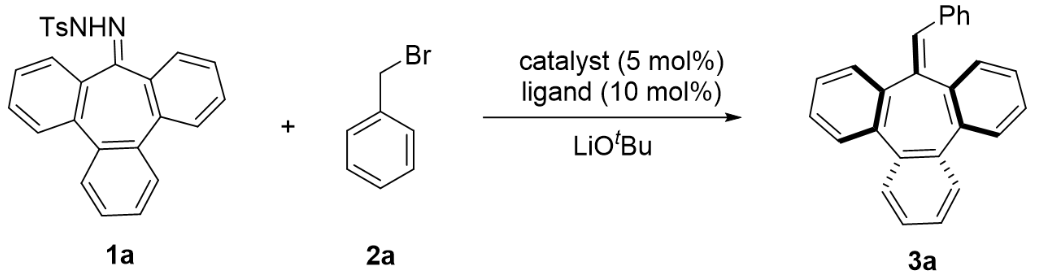

1a **2a** **3a**

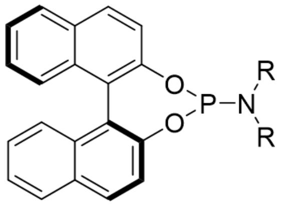

R = Me, **L1**
R = ^{i}Pr, **L2**

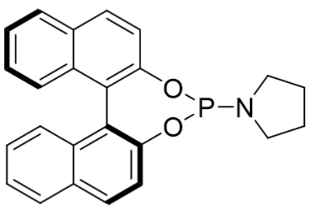

L3

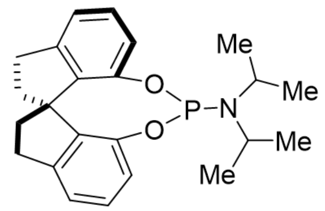

L4

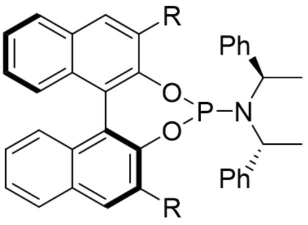

R = H, (S, R, R)-**L5**
R = Me, (S, R, R)-**L6**

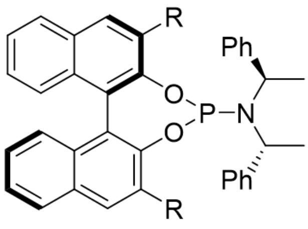

R = H, (R, R, R)-**L7**
R = Me, (R, R, R)-**L8**

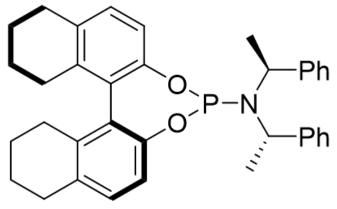

(S, S, S)-**L9**

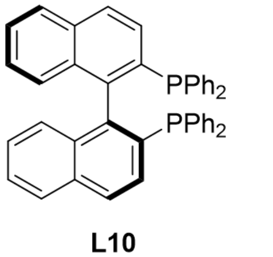

L10

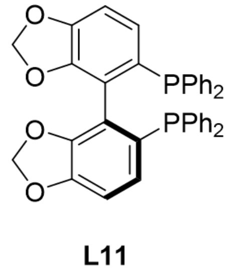

L11

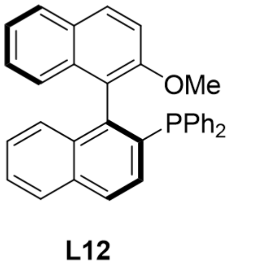

L12

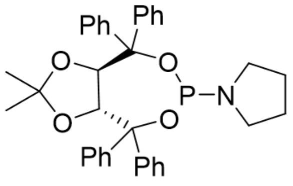

L13

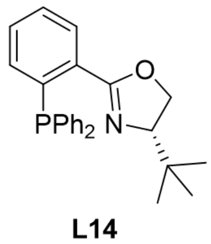

L14

entry	catalyst	ligand	yield (%)[b]	ee (%)[c]
1	$Pd(OAc)_2$	**L1**	42	24
2	$Pd_2(dba)_3$	**L1**	54	28
3	$[Pd(\eta\text{-}C_3H_5)Cl]_2$	**L1**	40	14
4	$Pd(PPh_3)_4$	**L1**	55	12
5	$Pd(dmdba)_2$	**L1**	50	22
6	$PdCl_2(CH_3CN)_2$	**L1**	44	10
7	$Pd(dba)_2$	**L1**	50	24
8	$Pd_2(dba)_3$	**L2**	66	67
9	$Pd_2(dba)_3$	**L3**	57	5
10	$Pd_2(dba)_3$	**L4**	55	62
11	$Pd_2(dba)_3$	**L5**	78	77
12	$Pd_2(dba)_3$	**L6**	55	7
13	$Pd_2(dba)_3$	**L7**	85	89
14	$Pd_2(dba)_3$	**L8**	52	21
15	$Pd_2(dba)_3$	**L9**	78	89
16	$Pd_2(dba)_3$	**L10**	trace	-
17	$Pd_2(dba)_3$	**L11**	60	3
18	$Pd_2(dba)_3$	**L12**	65	0
19	$Pd_2(dba)_3$	**L13**	45	11
20	$Pd_2(dba)_3$	**L14**	trace	-

[a]Reaction conditions: **1a** (0.1 mmol), **2a** (0.12 mmol), palladium (5.0 mol%), ligand (10.0 mol%), and the solvent (1.0 mL) in a sealed vial at 60 °C for 20 h. [b]Isolated yield. [c]Determined by chiral HPLC.

Supplementary Table 2. Optimization of the solvent and base[a]

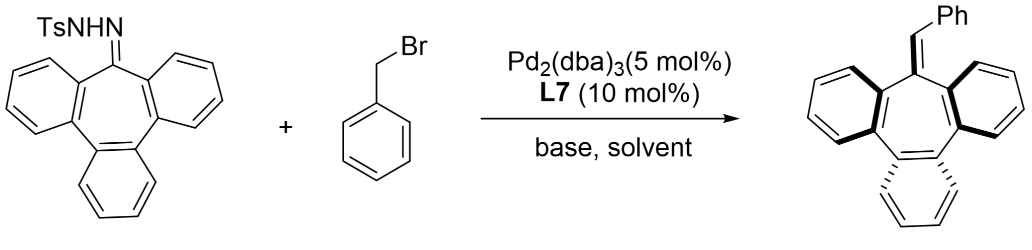

1a **2a** **3a**

entry	base	solvent	yield (%)[b]	ee (%)[c]
1	LiO^{t}Bu	Et_2O	41	87
2	LiO^{t}Bu	THF	65	89
3	LiO^{t}Bu	toluene	40	87

4	K_3PO_4	dioxane	66	75
5	Cs_2CO_3	dioxane	68	55
6	K_2CO_3	dioxane	62	77
7	NaOH	dioxane	70	34
8	NaO^{t}Bu	dioxane	trace	-
9[d]	LiO^{t}Bu	dioxane	80	91
10[e]	LiO^{t}Bu	dioxane	68	90
11[f]	LiO^{t}Bu	dioxane	42	50

[a]Reaction conditions: **1a** (0.1 mmol), **2a** (0.12 mmol), palladium (5.0 mol%), ligand (10.0 mol%), and the solvent (1.0 mL) in a sealed vial at 60 °C for 20 h. [b]Isolated yield. [c]Determined by chiral HPLC.[d]The reaction was performed at 50 °C for 24 h. [e]$Pd_2(dba)_3$ (2.5 mol%), **L7** (5.0 mol%), reaction time: 48 h. [f]Benzyl chloride (0.12 mmol) was used instead of **2a**.

5. Synthesis of tribenzocycloheptene 3

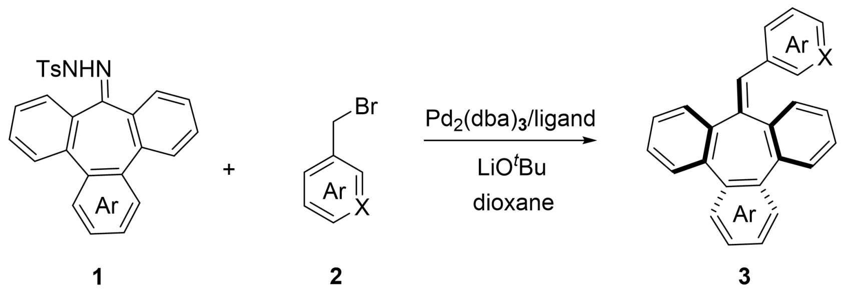

(1) Synthesis of racemic tribenzocycloheptene 3

Under nitrogen atmosphere, to a mixture of **1** (0.1 mmol, 1.0 equiv.), **2** (0.12 mmol, 1.2 equiv.), $Pd(PPh_3)_4$ (5 mol%), LiO^{t}Bu (3.0 equiv.) in a Schlenk tube was added dioxane (1.0 mL) at 70 °C (oil bath) for 20 h. After completion of the reaction, the solvent was removed under vacuum and the crude product was purified directly by column chromatography to afford the racemic product **3**.

(2) Synthesis of chiral tribenzocycloheptene (Procedure C):

Under nitrogen atmosphere, to a mixture of **1** (0.2 mmol, 1.0 equiv.), **2** (0.24 mmol, 1.2 equiv.), $Pd_2(dba)_3$ (5 mol%), ligand (**L7** or **L9**, 10 mol%), LiO^{t}Bu (3.0 equiv.) in a

Schlenk tube was added dioxane (2.0 mL). The resulting suspension was placed in an oil bath that had been preheated to 50 °C for 36 h. After completion of the reaction, the solvent was removed under vacuum and the crude product was purified with a chromatography column on silica gel to give **3** (petroleum ether/EtOAc).

6. Characterization and NMR spectra of products 3

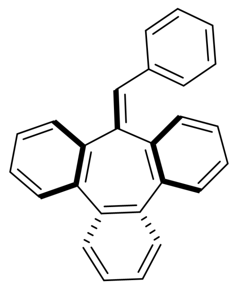

9-Benzylidene-9H-tribenzo[a,c,e][7]annulene (3a) was synthesized by following Procedure C. The crude material was purified by column chromatography (SiO_2, petroleum ether: EtOAc = 100:1) to provide **3a** as a white solid (52.8 mg, 80% yield).

^{1}H NMR (400 MHz, $CDCl_3$) δ 7.63 – 7.59 (m, 1H), 7.53 (dd, *J* = 7.8, 1.3 Hz, 1H), 7.50 – 7.44 (m, 2H), 7.38 – 7.31 (m, 3H), 7.26 (ddd, *J* = 7.9, 3.6, 1.5 Hz, 2H), 7.23 – 7.18 (m, 1H), 7.09 (d, *J* = 1.3 Hz, 1H), 7.05 – 7.02 (m, 1H), 6.99 (td, *J* = 5.7, 2.6 Hz, 3H), 6.93 (dd, *J* = 7.7, 2.0 Hz, 2H), 6.47 (s, 1H).

^{13}C NMR (100 MHz, $CDCl_3$) δ 146.4, 142.4, 142.3, 138.9, 138.8, 137.8, 137.4, 136.5, 130.4, 129.9, 129.6, 129.2, 129.0, 128.9, 128.0, 127.9, 127.6, 127.5, 127.5, 127.5, 127.3, 126.9, 126.0.

HRMS: (ESI) m/z: $[M+K]^+$ Calcd for $C_{26}H_{18}K$ 369.1040; Found 369.1048.

Optical $[\alpha]_{25}^{D}$ = -324.6 ° (c = 0.25, CH_2Cl_2, 91% ee)

HPLC (IA-H, ^{i}PrOH/n-hexane = 5/95, flow rate = 0.5 mL/min, l = 254 nm) t_R = 9.6 min (major), 10.5 min (minor).

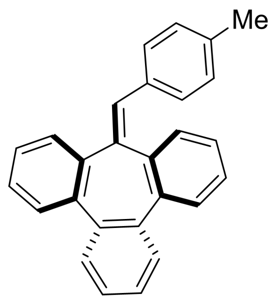

9-(4-Methylbenzylidene)-9H-tribenzo[a,c,e][7]annulene (3b) was synthesized by following Procedure C. The crude material was purified by column chromatography (SiO_2, petroleum ether) to provide **3b** as a white solid (48.2 mg, 70% yield).

^{1}H NMR (600 MHz, $CDCl_3$) δ 7.75 (dd, *J* = 7.4, 1.8 Hz, 1H), 7.69 – 7.65 (m, 1H), 7.63 – 7.59 (m, 2H), 7.51 – 7.49 (m, 1H), 7.47 (ddd, *J* = 6.8, 5.7, 1.8 Hz, 2H), 7.43 – 7.38 (m, 2H), 7.37 (dd, *J* = 1.5, 0.6 Hz, 1H), 7.23 (dd, *J* = 7.3, 1.2 Hz, 1H), 7.21 (dd, *J* = 1.5, 0.5 Hz, 1H), 6.98 (d, *J* = 8.4 Hz, 2H), 6.96 (d, *J* = 8.2 Hz, 2H), 6.58 (s, 1H), 2.27 (s, 3H).

^{13}C NMR (100 MHz, $CDCl_3$) δ 146.5, 142.5, 141.5, 139.0, 138.8, 137.8, 137.4, 136.7, 133.6, 130.4, 129.9, 129.6, 129.1, 128.9, 128.9, 128.7, 128.0, 127.9, 127.5, 127.5, 127.4, 127.3, 126.0, 21.1.

HRMS: (ESI) m/z: $[M+K]^+$ Calcd for $C_{27}H_{20}K$ 383.1197; Found 383.1200.

Optical $[\alpha]_{25}^{D}$ = -331.5 ° (c = 0.25, CH_2Cl_2, 91% ee)

HPLC (IA-H, ^{i}PrOH/n-hexane = 5/95, flow rate = 0.5 mL/min, l = 254 nm) t_R = 9.7 min (major), 11.7 min (minor).

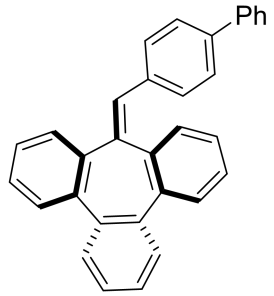

9-([1,1'-Biphenyl]-4-ylmethylene)-9H-tribenzo[a,c,e][7]annulene (3c) was synthesized by following Procedure C. The crude material was purified by column chromatography (SiO_2, petroleum ether) to provide **3c** as a white solid (55.3 mg, 68% yield).

^{1}H NMR (400 MHz, $CDCl_3$) δ 7.65 (dd, *J* = 7.2, 2.1 Hz, 1H), 7.59 – 7.55 (m, 1H), 7.53 – 7.48 (m, 2H), 7.43 (d, *J* = 1.6 Hz, 1H), 7.42 – 7.39 (m, 2H), 7.38 – 7.33 (m,

2H), 7.30 (d, J = 1.0 Hz, 1H), 7.28 (ddd, J = 8.2, 5.5, 2.2 Hz, 6H), 7.21 (d, J = 7.4 Hz, 1H), 7.15 – 7.12 (m, 2H), 7.03 (d, J = 8.3 Hz, 2H), 6.52 (s, 1H).

^{13}C NMR (100 MHz, $CDCl_3$) δ 146.4, 142.5, 142.4, 140.6, 139.5, 138.9, 138.8, 137.8, 137.4, 135.5, 130.4, 129.9, 129.7, 129.6, 128.9, 128.7, 128.6, 128.0, 128.0, 127.7, 127.5, 127.5, 127.4, 127.2, 126.8, 126.6, 126.0.

HRMS: (ESI) m/z: $[M+K]^+$ Calcd for $C_{32}H_{22}K$ 445.1353; Found 445.1357.

Optical $[\alpha]_{25}^{D}$ = -471.6 ° (c = 0.25, CH_2Cl_2, 88% ee)

HPLC (AD-H, ^{i}PrOH/n-hexane = 5/95, flow rate = 0.5 mL/min, l = 254 nm) t_R = 13.3 min (major), 21.3 min (minor).

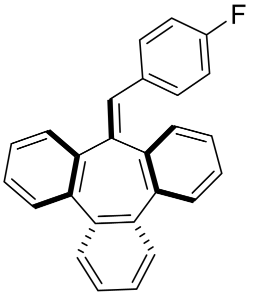

9-(4-Fluorobenzylidene)-9H-tribenzo[a,c,e][7]annulene (3d) was synthesized by following Procedure C. The crude material was purified by column chromatography (SiO_2, petroleum ether) to provide **3d** as a white solid (48.7 mg, 70% yield).

^{1}H NMR (400 MHz, $CDCl_3$) δ 7.65 (dd, J = 7.1, 2.1 Hz, 1H), 7.58 (dd, J = 7.8, 1.3 Hz, 1H), 7.55 – 7.49 (m, 2H), 7.39 (ddd, J = 6.9, 3.4, 2.0 Hz, 3H), 7.34 – 7.29 (m, 2H), 7.29 – 7.25 (m, 1H), 7.14 (td, J = 7.5, 1.3 Hz, 1H), 7.05 (dd, J = 7.7, 1.4 Hz, 1H), 6.93 (dd, J = 8.7, 5.6 Hz, 2H), 6.74 (t, J = 8.7 Hz, 2H), 6.47 (s, 1H).

^{13}C NMR (100 MHz, $CDCl_3$) δ 161.6 (d, J = 247.0 Hz), 146.2, 142.2 (d, J = 1.8 Hz), 142.0, 138.8 (d, J = 2.6 Hz), 137.9, 137.4, 132.6 (d, J = 3.4 Hz), 130.8, 130.7, 130.4, 129.9, 129.7, 128.9, 128.0 (d, J = 1.5 Hz), 127.8, 127.7, 127.6 (d, J = 1.5 Hz), 127.5, 127.2, 126.0, 115.0, 114.8.

^{19}F NMR (376 MHz, $CDCl_3$) δ -114.68.

HRMS: (ESI) m/z: $[M+K]^+$ Calcd for $C_{26}H_{17}FK$ 387.0946; Found 387.0952.

Optical $[\alpha]_{25}^{D}$ = -282.8 ° (c = 0.25, CH_2Cl_2, 96% ee)

HPLC (IA-H, ^{i}PrOH/n-hexane = 5/95, flow rate = 0.5 mL/min, l = 254 nm) t_R = 9.7 min (major), 10.3 min (minor).

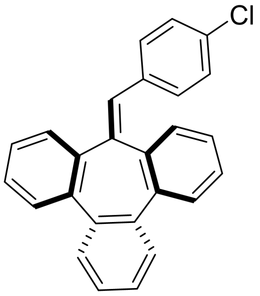

9-(4-Chlorobenzylidene)-9H-tribenzo[a,c,e][7]annulene (3e) was synthesized by following Procedure C. The crude material was purified by column chromatography (SiO_2, petroleum ether) to provide **3e** as a white solid (52.5 mg, 72% yield).

^{1}H NMR (400 MHz, $CDCl_3$) δ 7.63 – 7.58 (m, 1H), 7.52 (dd, J = 7.8, 1.2 Hz, 1H), 7.47 (td, J = 6.6, 2.4 Hz, 2H), 7.37 – 7.31 (m, 3H), 7.28 – 7.24 (m, 2H), 7.22 (dd, J = 7.6, 1.4 Hz, 1H), 7.09 (td, J = 7.5, 1.3 Hz, 1H), 7.00 – 6.93 (m, 3H), 6.83 (d, J = 8.6 Hz, 2H), 6.41 (s, 1H).

^{13}C NMR (100 MHz, $CDCl_3$) δ 146.0, 143.2, 141.9, 138.7, 138.7, 137.8, 137.3, 135.0, 132.5, 130.4, 130.4, 129.9, 129.7, 128.9, 128.1, 128.0, 128.0, 127.8, 127.7, 127.7, 127.6, 127.6, 127.2, 125.9.

HRMS: (ESI) m/z: $[M+K]^+$ Calcd for $C_{26}H_{17}ClK$ 403.0650; Found 403.0653.

Optical $[\alpha]_{25}^{D}$ = -345.8 ° (c = 0.25, CH_2Cl_2, 97% ee)

HPLC (IA-H, ^{i}PrOH/n-hexane = 5/95, flow rate = 0.5 mL/min, l = 254 nm) t_R = 10.3 min (major), 11.1 min (minor).

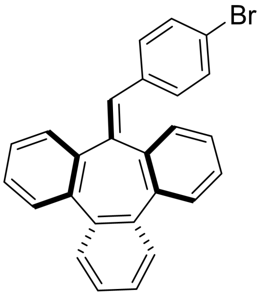

9-(4-Bromobenzylidene)-9H-tribenzo[a,c,e][7]annulene (3f) was synthesized by following Procedure C. The crude material was purified by column chromatography (SiO_2, petroleum ether) to provide **3f** as a white solid (50.7 mg, 62% yield).

^{1}H NMR (400 MHz, $CDCl_3$) δ 7.63 (dd, J = 7.1, 2.1 Hz, 1H), 7.56 (dd, J = 7.9, 1.3 Hz, 1H), 7.53 – 7.48 (m, 2H), 7.41 – 7.34 (m, 3H), 7.32 – 7.28 (m, 2H), 7.25 (dd, J = 7.7, 1.4 Hz, 1H), 7.16 (s, 1H), 7.14 (d, J = 2.2 Hz, 1H), 7.11 (dd, J = 7.5, 1.3 Hz, 1H), 7.01 (dd, J = 7.7, 1.4 Hz, 1H), 6.81 (d, J = 8.5 Hz, 2H), 6.42 (s, 1H).

^{13}**C NMR** (100 MHz, $CDCl_3$) δ 146.0, 143.3, 141.9, 138.7, 138.7, 137.7, 137.3, 135.4, 131.1, 130.7, 130.4, 129.9, 129.7, 128.9, 128.0, 128.0, 127.8, 127.8, 127.7, 127.6, 127.6, 127.1, 125.9, 120.7.

HRMS: (ESI) m/z: $[M+K]^+$ Calcd for $C_{26}H_{17}BrK$ 447.0145; Found 447.0154.

Optical $[\alpha]_{25}^{D}$ = -374.8 ° (c = 0.25, CH_2Cl_2, 93% ee)

HPLC (AD-H, ^{i}PrOH/n-hexane = 5/95, flow rate = 0.5 mL/min, l = 254 nm) t_R = 12.8 min (major), 14.5 min (minor).

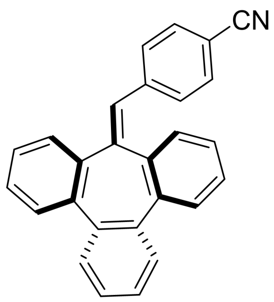

4-((9H-tribenzo[a,c,e][7]annulen-9-ylidene)methyl)benzonitrile (3g) was synthesized by following Procedure C. The crude material was purified by column chromatography (SiO_2, petroleum ether: EtOAc = 20:1) to provide **3g** as a white solid (52.6 mg, 74% yield).

^{1}**H NMR** (400 MHz, $CDCl_3$) δ 7.74 (dd, *J* = 7.2, 2.0 Hz, 1H), 7.67 (dd, *J* = 7.9, 1.2 Hz, 1H), 7.61 (dt, *J* = 5.5, 3.2 Hz, 2H), 7.51 – 7.44 (m, 3H), 7.43 – 7.36 (m, 5H), 7.22 (td, *J* = 7.5, 1.3 Hz, 1H), 7.10 (d, *J* = 8.4 Hz, 2H), 7.05 (dd, *J* = 7.7, 1.4 Hz, 1H), 6.58 (s, 1H).

^{13}**C NMR** (100 MHz, $CDCl_3$) δ 146.2, 145.4, 141.3, 138.4, 138.4, 137.6, 137.1, 131.7, 130.5, 129.9, 129.8, 129.6, 129.0, 128.2, 128.1, 128.1, 128.0, 127.8, 127.7, 127.3, 126.9, 125.7, 118.9, 110.0.

HRMS: (ESI) m/z: $[M+Na]^+$ Calcd for $C_{27}H_{17}NNa$ 378.1253; Found 378.1253.

Optical $[\alpha]_{25}^{D}$ = -390.2 ° (c = 0.25, CH_2Cl_2, 91% ee)

HPLC (AD-H, ^{i}PrOH/n-hexane = 10/90, flow rate = 0.5 mL/min, l = 254 nm) t_R = 16.2 min (major), 22.0 min (minor).

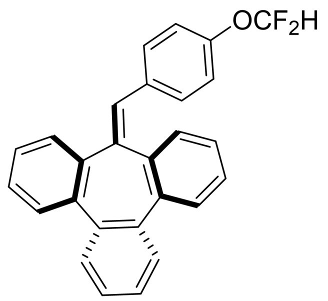

9-(4-(Difluoromethoxy)benzylidene)-9H-tribenzo[a,c,e][7]annulene (3h) was synthesized by following Procedure C. The crude material was purified by column chromatography (SiO_2, petroleum ether: EtOAc = 100:1) to provide **3h** as a white solid (53.1 mg, 67% yield).

^{1}H NMR (400 MHz, $CDCl_3$) δ 7.68 – 7.62 (m, 1H), 7.58 (dd, *J* = 7.8, 1.3 Hz, 1H), 7.55 – 7.49 (m, 2H), 7.39 (dd, *J* = 6.4, 2.9 Hz, 3H), 7.35 – 7.25 (m, 3H), 7.19 – 7.13 (m, 1H), 7.06 (dd, *J* = 7.7, 1.4 Hz, 1H), 6.96 (d, *J* = 8.7 Hz, 2H), 6.79 (d, *J* = 8.7 Hz, 2H), 6.54 – 6.15 (m, 2H).

^{13}C NMR (100 MHz, $CDCl_3$) δ 149.9, 146.1, 142.7, 142.0, 138.7 (d, *J* = 2.3 Hz), 137.8, 137.3, 133.8, 130.5, 130.4, 129.9, 129.7, 128.9, 128.0 (d, *J* = 1.8 Hz), 127.8, 127.7, 127.6, 127.6, 127.6, 127.2, 125.9, 118.8, 115.8, 115.8 (t, *J* = 259.4 Hz).

^{19}F NMR (565 MHz, $CDCl_3$) δ -80.49.

HRMS: (ESI) m/z: $[M+K]^+$ Calcd for $C_{27}H_{18}F_2OK$ 435.0957; Found 435.0966.

Optical $[\alpha]_{25}^{D}$ = -269.7 ° (c = 0.25, CH_2Cl_2, 93% ee)

HPLC (IA-H, ^{i}PrOH/n-hexane = 5/95, flow rate = 0.5 mL/min, l = 254 nm) t_R = 13.0 min (major), 14.1 min (minor).

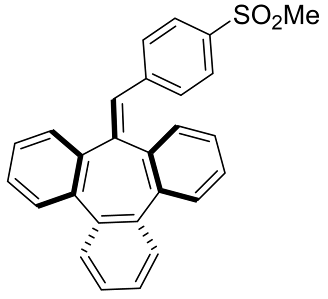

9-(4-(Methylsulfonyl)benzylidene)-9H-tribenzo[a,c,e][7]annulene (3i) was synthesized by following Procedure C. The crude material was purified by column chromatography (SiO_2, petroleum ether: EtOAc = 5:1) to provide **3i** as a white solid (58.7 mg, 72% yield).

^{1}H NMR (400 MHz, $CDCl_3$) δ 7.64 (dd, *J* = 7.2, 2.0 Hz, 1H), 7.57 (dd, *J* = 8.4, 2.2 Hz, 3H), 7.51 (dt, *J* = 6.2, 3.4 Hz, 2H), 7.37 (ddt, *J* = 6.7, 4.8, 2.3 Hz, 3H), 7.33 –

7.29 (m, 2H), 7.27 (dd, J = 7.7, 1.4 Hz, 1H), 7.10 (t, J = 7.7 Hz, 3H), 6.96 (dd, J = 7.7, 1.4 Hz, 1H), 6.51 (s, 1H), 2.86 (s, 3H).

^{13}C NMR (100 MHz, $CDCl_3$) δ 146.4, 145.5, 142.2, 141.3, 138.4, 138.4, 138.2, 137.6, 137.1, 130.5, 129.9, 129.8, 129.0, 128.2, 128.2, 128.1, 128.0, 127.8, 127.7, 127.1, 127.0, 126.9, 125.7, 44.4.

HRMS: (ESI) m/z: $[M+Na]^+$ Calcd for $C_{27}H_{20}O_2SNa$ 431.1076; Found 431.1080.

Optical $[\alpha]_{25}^{D}$ = -289.2 ° (c = 0.25, CH_2Cl_2, 92% ee)

HPLC (IA-H, ^{i}PrOH/n-hexane = 20/80, flow rate = 0.5 mL/min, l = 254 nm) t_R = 20.5 min (major), 25.9 min (minor).

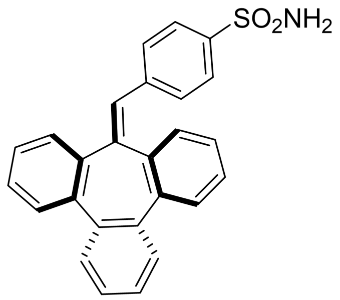

4-((9H-tribenzo[a,c,e][7]annulen-9-ylidene)methyl)benzenesulfonamide (3j) was synthesized by following Procedure C. The crude material was purified by column chromatography (SiO_2, petroleum ether: EtOAc = 2:1) to provide **3j** as a white solid (41.7 mg, 51% yield).

^{1}H NMR (400 MHz, $(CD_3)_2SO$) δ 7.79 (dd, J = 7.2, 2.0 Hz, 1H), 7.70 (dd, J = 7.8, 1.3 Hz, 1H), 7.65 – 7.60 (m, 2H), 7.59 (s, 1H), 7.57 (s, 1H), 7.55 – 7.49 (m, 3H), 7.49 – 7.44 (m, 2H), 7.43 (d, J = 1.3 Hz, 1H), 7.33 – 7.26 (m, 3H), 7.17 (d, J = 1.8 Hz, 1H), 7.15 (s, 1H), 7.06 (dd, J = 7.7, 1.4 Hz, 1H), 6.71 (s, 1H).

^{13}C NMR (150 MHz, $(CD_3)_2SO$) δ 145.4, 144.3, 142.4, 141.4, 139.6, 138.0, 138.0, 137.2, 136.6, 130.4, 130.0, 129.9, 129.2, 129.0, 128.4, 128.2, 128.1, 128.1, 127.6, 126.9, 125.9, 125.5.

HRMS: (ESI) m/z: $[M+Na]^+$ Calcd for $C_{26}H_{19}NO_2SNa$ 432.1029; Found 432.1033.

Optical $[\alpha]_{25}^{D}$ = -197.4 ° (c = 0.25, CH_2Cl_2, 87% ee)

HPLC (AD-H, ^{i}PrOH/n-hexane = 25/75, flow rate = 0.5 mL/min, l = 254 nm) t_R = 13.1 min (major), 25.3 min (minor).

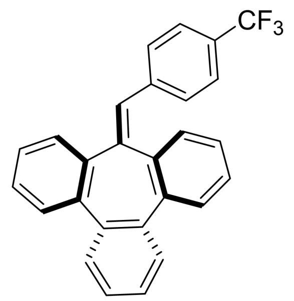

9-(4-(Trifluoromethyl)benzylidene)-9H-tribenzo[a,c,e][7]annulene (3k) was synthesized by following Procedure C. The crude material was purified by column chromatography (SiO_2, petroleum ether) to provide **3k** as a yellow solid (58.9 mg, 74% yield).

^{1}H NMR (400 MHz, $CDCl_3$) δ 7.64 (dd, *J* = 6.5, 2.3 Hz, 1H), 7.57 (d, *J* = 7.8 Hz, 1H), 7.51 (q, *J* = 5.0, 4.4 Hz, 2H), 7.38 (dd, *J* = 6.7, 3.1 Hz, 3H), 7.28 (ddd, *J* = 11.0, 8.0, 3.4 Hz, 5H), 7.16 – 7.11 (m, 1H), 7.04 (s, 1H), 7.03 – 6.97 (m, 2H), 6.51 (s, 1H).

^{13}C NMR (100 MHz, $CDCl_3$) δ 145.8, 145.0, 141.6, 140.1 (d, *J* = 1.5 Hz), 138.6 (d, *J* = 2.1 Hz), 137.7, 137.2, 130.5, 129.9, 129.7, 129.3, 129.0, 128.7, 128.4, 128.1, 128.1, 128.0, 127.9, 127.7, 127.7, 127.6, 127.0, 125.8, 124.9 (q, *J* = 3.9 Hz), 124.1 (q, *J* = 271.8 Hz).

^{19}F NMR (376 MHz, $CDCl_3$) δ -62.52 (d, *J* = 2.8 Hz).

HRMS: (ESI) m/z: $[M+H]^+$ Calcd for $C_{27}H_{18}F_3$ 399.1355; Found 399.1353.

Optical $[\alpha]_{25}^{D}$ = -303.0 ° (c = 0.25, CH_2Cl_2, 94% ee)

HPLC (AD-H, ^{i}PrOH/n-hexane = 5/95, flow rate = 0.5 mL/min, l = 254 nm) t_R = 9.7 min (major), 12.7 min (minor).

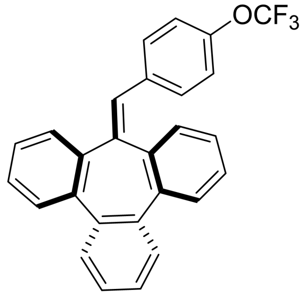

9-(4-(Trifluoromethoxy)benzylidene)-9H-tribenzo[a,c,e][7]annulene (3l) was synthesized by following Procedure C. The crude material was purified by column chromatography (SiO_2, petroleum ether) to provide **3l** as a white solid (68.8 mg, 83% yield).

^{1}H NMR (400 MHz, $CDCl_3$) δ 7.66 – 7.62 (m, 1H), 7.56 (d, J = 7.8 Hz, 1H), 7.50 (dt, J = 5.1, 2.8 Hz, 2H), 7.37 (td, J = 4.7, 1.9 Hz, 3H), 7.31 – 7.27 (m, 2H), 7.26 (s, 1H), 7.14 – 7.10 (m, 1H), 7.01 (dd, J = 7.8, 5.9 Hz, 2H), 6.88 (d, J = 7.8 Hz, 1H), 6.85 – 6.81 (m, 1H), 6.76 (s, 1H), 6.46 (s, 1H).

^{13}C NMR (100 MHz, $CDCl_3$) δ 148.9 (d, J = 2.2 Hz), 145.8, 144.3, 141.6, 138.7, 138.6 (d, J = 2.7 Hz), 137.7, 137.3, 130.4, 129.9, 129.7, 129.2, 128.9, 128.1, 128.0, 127.9, 127.8, 127.6, 127.5 (d, J = 3.3 Hz), 127.0, 125.8, 121.2, 120.3 (q, J = 257.1 Hz), 119.2.

^{19}F NMR (376 MHz, $CDCl_3$) δ -57.68 – -57.77 (m).

HRMS: (ESI) m/z: $[M+H]^+$ Calcd for $C_{27}H_{18}F_3O$ 415.1304; Found 415.1305.

Optical $[\alpha]_{25}^{D}$ = -277.9 ° (c = 0.25, CH_2Cl_2, 91% ee)

HPLC (AD-H, ^{i}PrOH/n-hexane = 5/95, flow rate = 0.5 mL/min, l = 254 nm) t_R = 8.2 min (major), 9.0 min (minor).

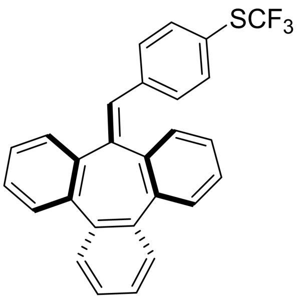

(4-((9H-tribenzo[a,c,e][7]annulen-9-ylidene)methyl)phenyl)(trifluoromethyl) sulfane (3m) was synthesized by following Procedure C. The crude material was purified by column chromatography (SiO_2, petroleum ether) to provide **3m** as a yellow solid (59.1 mg, 68% yield).

^{1}H NMR (400 MHz, $CDCl_3$) δ 7.62 (dd, J = 6.7, 2.2 Hz, 1H), 7.56 (d, J = 7.8 Hz, 1H), 7.49 (dt, J = 6.2, 3.5 Hz, 2H), 7.40 – 7.33 (m, 3H), 7.31 – 7.22 (m, 5H), 7.14 – 7.10 (m, 1H), 7.01 (d, J = 7.6 Hz, 1H), 6.98 (s, 1H), 6.96 (s, 1H), 6.46 (s, 1H).

^{13}C NMR (100 MHz, $CDCl_3$) δ 145.8, 144.9, 141.6, 139.2, 138.6 (d, J = 1.9 Hz), 137.6, 137.2, 135.8, 131.0, 130.4, 130.1, 129.9, 129.8, 129.0, 128.1, 128.0, 128.0, 127.8, 127.7, 127.6, 127.6, 127.0, 125.8, 122.6 (d, J = 266.7 Hz), 122.2 (d, J = 2.1 Hz).

^{19}F NMR (376 MHz, $CDCl_3$) δ -42.74 (d, J = 4.0 Hz).

HRMS: (ESI) m/z: $[M+H]^+$ Calcd for $C_{27}H_{18}F_3S$ 431.1076; Found 431.1088.

Optical $[\alpha]_{25}^{D}$ = -320.7 ° (c = 0.25, CH_2Cl_2, 94% ee)

HPLC (AD-H, ^{i}PrOH/n-hexane = 2/98, flow rate = 0.5 mL/min, l = 254 nm) t_R = 17.3 min (major), 14.8 min (minor).

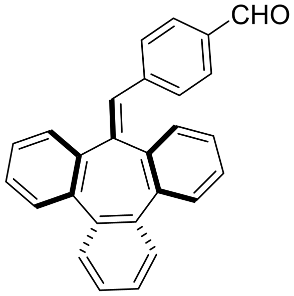

4-((9H-tribenzo[a,c,e][7]annulen-9-ylidene)methyl)benzaldehyde (3n) was synthesized by following Procedure C. The crude material was purified by column chromatography (SiO_2, petroleum ether: EtOAc = 20:1) to provide **3n** as a white solid (32.2 mg, 45% yield).

^{1}H NMR (400 MHz, $CDCl_3$) δ 9.78 (s, 1H), 7.66 (dd, J = 6.7, 2.2 Hz, 1H), 7.58 (d, J = 7.8 Hz, 1H), 7.56 – 7.48 (m, 4H), 7.39 (dd, J = 6.2, 3.8 Hz, 3H), 7.34 – 7.29 (m, 2H), 7.29 – 7.24 (m, 1H), 7.09 (t, J = 7.9 Hz, 3H), 6.99 (dd, J = 7.7, 1.4 Hz, 1H), 6.54 (s, 1H).

^{13}C NMR (100 MHz, $CDCl_3$) δ 191.6, 145.9, 145.7, 142.9, 141.6, 138.5, 138.5, 137.6, 137.1, 134.6, 130.4, 129.9, 129.8, 129.6, 129.4, 129.0, 128.1, 128.1, 128.0, 127.9, 127.7, 127.7, 127.0, 125.8.

HRMS: (ESI) m/z: $[M+Na]^+$ Calcd for $C_{27}H_{18}ONa$ 381.1250; Found 381.1254.

Optical $[\alpha]_{25}^{D}$ = -375.1 ° (c = 0.25, CH_2Cl_2, 83% ee)

HPLC (AD-H, ^{i}PrOH/n-hexane = 10/90, flow rate = 0.5 mL/min, l = 254 nm) t_R = 18.3 min (major), 27.4 min (minor).

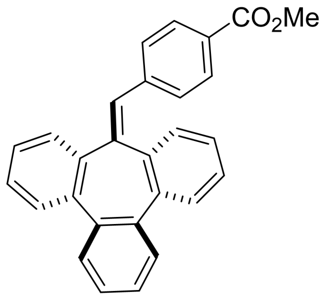

Methyl 4-((9H-tribenzo[a,c,e][7]annulen-9-ylidene)methyl)benzoate (3o) was synthesized by following Procedure C. The crude material was purified by column

chromatography (SiO_2, petroleum ether: EtOAc = 20:1) to provide **3o** as a white solid (50.4 mg, 65% yield).

^{1}H NMR (400 MHz, $CDCl_3$) δ 7.71 (d, *J* = 1.8 Hz, 1H), 7.69 (d, *J* = 1.8 Hz, 1H), 7.66 (dd, *J* = 7.3, 1.9 Hz, 1H), 7.57 (dd, *J* = 7.9, 1.2 Hz, 1H), 7.54 – 7.50 (m, 2H), 7.41 – 7.36 (m, 3H), 7.33 – 7.29 (m, 2H), 7.26 (dd, *J* = 7.7, 1.4 Hz, 1H), 7.10 (td, *J* = 7.5, 1.3 Hz, 1H), 7.02 – 6.97 (m, 3H), 6.53 (s, 1H), 3.76 (s, 3H).

^{13}C NMR (100 MHz, $CDCl_3$) δ 166.8, 145.8, 145.0, 141.8, 141.3, 138.6, 138.6, 137.7, 137.2, 130.4, 129.9, 129.7, 129.2, 129.1, 128.9, 128.2, 128.1, 128.0, 127.9, 127.8, 127.6, 127.6, 127.1, 125.8, 51.9.

HRMS: (ESI) m/z: $[M+Na]^+$ Calcd for $C_{28}H_{20}O_2Na$ 411.1356; Found 411.1360.

Optical $[\alpha]_{25}^{D}$ = +383.2° (c = 0.25, CH_2Cl_2, 92% ee)

HPLC (AD-H, ^{i}PrOH/n-hexane = 10/90, flow rate = 0.5 mL/min, l = 254 nm) t_R = 14.9 min (major), 12.5 min (minor).

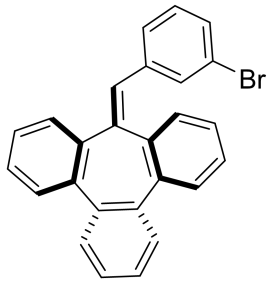

9-(3-Bromobenzylidene)-9H-tribenzo[a,c,e][7]annulene (3p) was synthesized by following Procedure C. The crude material was purified by column chromatography (SiO_2, petroleum ether) to provide **3p** as a white solid (82.8 mg, 68% yield).

^{1}H NMR (400 MHz, $CDCl_3$) δ 7.64 (dd, *J* = 7.3, 1.9 Hz, 1H), 7.56 (dd, *J* = 7.9, 1.3 Hz, 1H), 7.53 – 7.47 (m, 2H), 7.36 (ddt, *J* = 8.6, 5.9, 3.0 Hz, 3H), 7.31 – 7.27 (m, 2H), 7.26 (d, *J* = 1.5 Hz, 1H), 7.14 – 7.10 (m, 3H), 7.01 (dd, *J* = 7.7, 1.4 Hz, 1H), 6.83 (d, *J* = 7.5 Hz, 1H), 6.81 (d, *J* = 1.6 Hz, 1H), 6.41 (s, 1H).

^{13}C NMR (100 MHz, $CDCl_3$) δ 145.8, 144.1, 141.7, 138.7, 138.6, 138.6, 137.7, 137.3, 132.2, 130.4, 129.9, 129.7, 129.7, 129.3, 128.9, 128.0, 127.9, 127.7, 127.6, 127.6, 127.6, 127.5, 127.1, 125.9, 122.0.

HRMS: (ESI) m/z: $[M+K]^+$ Calcd for $C_{26}H_{17}BrK$ 447.0145; Found 447.0154.

Optical $[\alpha]_{25}^{D}$ = -274.1 ° (c = 0.25, CH_2Cl_2, 91% ee)

HPLC (AD-H, ^{i}PrOH/n-hexane = 5/95, flow rate = 0.5 mL/min, l = 254 nm) t_R = 10.8 min (major), 13.4 min (minor).

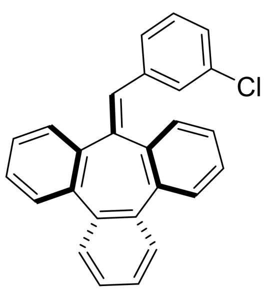

9-(3-Chlorobenzylidene)-9H-tribenzo[a,c,e][7]annulene (3q) was synthesized by following Procedure C. The crude material was purified by column chromatography (SiO_2, petroleum ether) to provide **3q** as a white solid (54.7 mg, 75% yield).

^{1}H NMR (400 MHz, $CDCl_3$) δ 7.66 – 7.62 (m, 1H), 7.56 (dd, *J* = 7.8, 1.3 Hz, 1H), 7.53 – 7.47 (m, 2H), 7.37 (ddt, *J* = 8.5, 6.1, 3.0 Hz, 3H), 7.32 – 7.28 (m, 2H), 7.26 (d, *J* = 1.5 Hz, 1H), 7.12 (td, *J* = 7.5, 1.3 Hz, 1H), 7.01 (dd, *J* = 7.7, 1.4 Hz, 1H), 6.96 (d, *J* = 1.4 Hz, 2H), 6.94 – 6.89 (m, 1H), 6.79 – 6.74 (m, 1H), 6.42 (s, 1H).

^{13}C NMR (100 MHz, $CDCl_3$) δ 145.9, 144.0, 141.7, 138.7, 138.6, 138.4, 137.7, 137.3, 133.7, 130.4, 130.0, 129.7, 129.2, 129.1, 128.9, 128.0, 127.9, 127.7, 127.6, 127.6, 127.2, 127.1, 126.8, 125.9.

HRMS: (ESI) m/z: $[M+H]^+$ Calcd for $C_{26}H_{18}Cl$ 365.1092; Found 365.1097.

Optical $[\alpha]_{25}^{D}$ = -294.9 ° (c = 0.25, CH_2Cl_2, 93% ee)

HPLC (IA-H, ^{i}PrOH/n-hexane = 5/95, flow rate = 0.5 mL/min, l = 254 nm) t_R = 9.7 min (major), 10.5 min (minor).

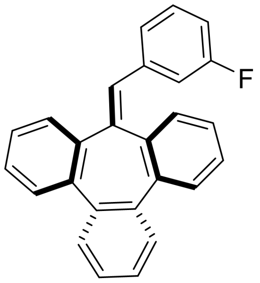

9-(3-Fluorobenzylidene)-9H-tribenzo[a,c,e][7]annulene (3r) was synthesized by following Procedure C. The crude material was purified by column chromatography (SiO_2, petroleum ether) to provide **3r** as a white solid (44.6 mg, 64% yield).

^{1}H NMR (400 MHz, $CDCl_3$) δ 7.64 (d, *J* = 7.0 Hz, 1H), 7.55 (dd, *J* = 7.8, 1.3 Hz, 1H), 7.52 – 7.47 (m, 2H), 7.36 (ddd, *J* = 6.6, 4.2, 2.0 Hz, 3H), 7.31 – 7.27 (m, 2H), 7.26 (d, *J* = 1.6 Hz, 1H), 7.15 – 7.10 (m, 1H), 7.04 (dd, *J* = 7.7, 1.4 Hz, 1H), 7.00 – 6.94 (m, 1H), 6.74 – 6.66 (m, 2H), 6.63 (d, *J* = 10.5 Hz, 1H), 6.45 (s, 1H).

^{13}**C NMR** (100 MHz, $CDCl_3$) δ 162.4 (d, J = 244.6 Hz), 145.9, 143.8, 141.7, 138.8, 138.7 (d, J = 2.3 Hz), 138.6, 137.6, 137.3, 130.4, 129.9, 129.7, 129.3 (d, J = 8.3 Hz), 128.9, 128.0 (d, J = 1.7 Hz), 127.9, 127.8 (d, J = 2.6 Hz), 127.7, 127.6 (d, J = 2.1 Hz), 127.1, 125.9, 125.1 (d, J = 2.9 Hz), 115.7, 115.5, 113.8, 113.6.

^{19}**F NMR** (565 MHz, $CDCl_3$) δ -113.53 (d, J = 8.6 Hz).

HRMS: (ESI) m/z: $[M+Na]^+$ Calcd for $C_{26}H_{17}FNa$ 371.1206; Found 371.1210.

Optical $[\alpha]_{25}^{D}$ = -239.4 ° (c = 0.25, CH_2Cl_2, 90% ee)

HPLC (AD-H, ^{i}PrOH/n-hexane = 5/95, flow rate = 0.5 mL/min, l = 254 nm) t_R = 11.4 min (major), 12.9 min (minor).

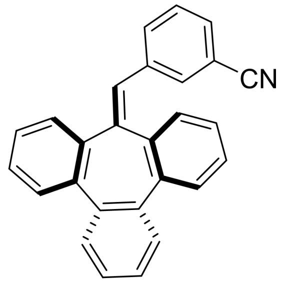

3-((9H-tribenzo[a,c,e][7]annulen-9-ylidene)methyl)benzonitrile (3s) was synthesized by following Procedure C. The crude material was purified by column chromatography (SiO_2, petroleum ether: EtOAc = 20:1) to provide **3s** as a white solid (49.7 mg, 70% yield).

^{1}**H NMR** (400 MHz, $CDCl_3$) δ 7.67 (dd, J = 7.1, 2.0 Hz, 1H), 7.58 (dd, J = 7.9, 1.2 Hz, 1H), 7.52 (dt, J = 9.0, 3.2 Hz, 2H), 7.43 – 7.34 (m, 3H), 7.33 – 7.30 (m, 2H), 7.29 – 7.23 (m, 2H), 7.21 (s, 1H), 7.14 – 7.07 (m, 3H), 6.95 (dd, J = 7.7, 1.4 Hz, 1H), 6.45 (s, 1H).

^{13}**C NMR** (100 MHz, $CDCl_3$) δ 145.4, 145.3, 141.1, 138.5, 138.4, 137.8, 137.7, 137.2, 133.3, 132.5, 130.4, 130.2, 130.0, 129.9, 129.0, 128.7, 128.2, 128.1, 128.1, 127.9, 127.7, 127.7, 126.8, 126.6, 125.7, 118.7, 112.1.

HRMS: (ESI) m/z: $[M+Na]^+$ Calcd for $C_{27}H_{17}NNa$ 378.1253; Found 378.1251.

Optical $[\alpha]_{25}^{D}$ = -309.7 ° (c = 0.25, CH_2Cl_2, 92% ee)

HPLC (IA-H, ^{i}PrOH/n-hexane = 10/90, flow rate = 0.5 mL/min, l = 254 nm) t_R = 14.7 min (major), 18.5 min (minor).

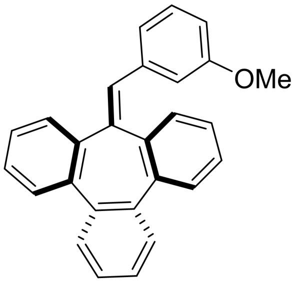

9-(3-Methoxybenzylidene)-9H-tribenzo[a,c,e][7]annulene (3t) was synthesized by following Procedure C. The crude material was purified by column chromatography (SiO_2, petroleum ether: EtOAc = 100:1) to provide **3t** as a white solid (58.4 mg, 81% yield).

^{1}H NMR (400 MHz, $CDCl_3$) δ 7.63 – 7.58 (m, 1H), 7.54 (dd, J = 7.9, 1.3 Hz, 1H), 7.52 – 7.46 (m, 2H), 7.40 – 7.32 (m, 3H), 7.30 – 7.24 (m, 2H), 7.23 (d, J = 1.7 Hz, 1H), 7.14 – 7.10 (m, 1H), 7.09 (d, J = 1.7 Hz, 1H), 6.94 (t, J = 7.9 Hz, 1H), 6.61 – 6.52 (m, 2H), 6.47 (d, J = 5.5 Hz, 2H), 3.42 (s, 3H).

^{13}C NMR (100 MHz, $CDCl_3$) δ 158.9, 146.3, 142.7, 142.4, 138.8, 138.8, 137.8, 137.3, 130.5, 129.8, 129.5, 128.9, 128.0, 127.9, 127.6, 127.5, 127.5, 127.4, 125.9, 122.0, 113.7, 113.4, 54.7.

HRMS: (ESI) m/z: $[M+H]^+$ Calcd for $C_{27}H_{21}O$ 361.1587; Found 361.1588.

Optical $[\alpha]_{25}^{D}$ = -267.8 ° (c = 0.25, CH_2Cl_2, 91% ee)

HPLC (IA-H, ^{i}PrOH/n-hexane = 5/95, flow rate = 0.5 mL/min, l = 254 nm) t_R = 10.4 min (major), 11.1 min (minor).

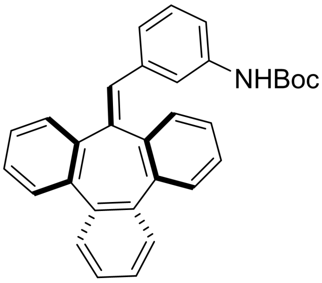

Tert-butyl (3-((9H-tribenzo[a,c,e][7]annulen-9-ylidene)methyl)phenyl)carbamate (3u) was synthesized by following Procedure C. The crude material was purified by column chromatography (SiO_2, petroleum ether: EtOAc = 10:1) to provide **3u** as a white solid (49.0 mg, 55% yield).

^{1}H NMR (400 MHz, $CDCl_3$) δ 7.74 (dd, J = 7.2, 1.9 Hz, 1H), 7.64 (dd, J = 7.8, 1.3 Hz, 1H), 7.62 – 7.56 (m, 2H), 7.50 – 7.43 (m, 3H), 7.42 – 7.36 (m, 2H), 7.34 (s, 1H),

7.20 (d, J = 1.3 Hz, 1H), 7.13 (dd, J = 7.7, 1.6 Hz, 3H), 6.99 (t, J = 7.9 Hz, 1H), 6.64 (d, J = 8.0 Hz, 1H), 6.56 (s, 1H), 6.30 (s, 1H), 1.51 (s, 9H).

^{13}C NMR (100 MHz, $CDCl_3$) δ 152.6, 146.2, 142.8, 142.2, 138.8, 138.7, 138.0, 137.7, 137.3, 130.4, 129.9, 129.6, 128.9, 128.9, 128.4, 128.0, 127.9, 127.6, 127.5, 127.5, 127.5, 127.4, 126.0, 123.9, 119.4, 117.0, 80.5, 28.3.

HRMS: (ESI) m/z: $[M+Na]^+$ Calcd for $C_{31}H_{27}NO_2Na$ 468.1934; Found 468.1932.

Optical $[\alpha]_{25}^{D}$ = -219.9 ° (c = 0.25, CH_2Cl_2, 85% ee)

HPLC (AD-H, ^{i}PrOH/n-hexane = 10/90, flow rate = 0.5 mL/min, l = 254 nm) t_R = 23.4 min (major), 29.1 min (minor).

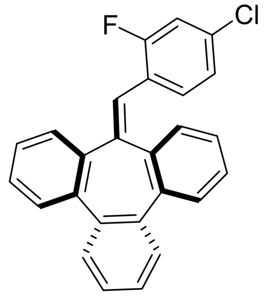

9-(4-Chloro-2-fluorobenzylidene)-9H-tribenzo[a,c,e][7]annulene (3v) was synthesized by following Procedure C. The crude material was purified by column chromatography (SiO_2, petroleum ether) to provide **3v** as a white oil (55.8 mg, 73% yield).

^{1}H NMR (400 MHz, $CDCl_3$) δ 7.66 – 7.62 (m, 1H), 7.58 – 7.50 (m, 3H), 7.43 – 7.36 (m, 3H), 7.35 – 7.29 (m, 2H), 7.26 (d, J = 1.4 Hz, 1H), 7.11 (td, J = 7.5, 1.3 Hz, 1H), 6.98 (dd, J = 7.7, 1.4 Hz, 1H), 6.93 (dd, J = 9.9, 2.0 Hz, 1H), 6.68 (dd, J = 8.5, 2.0 Hz, 1H), 6.63 (d, J = 8.0 Hz, 1H), 6.59 (d, J = 1.5 Hz, 1H).

^{13}C NMR (100 MHz, $CDCl_3$) δ 160.4 (d, J = 251.6 Hz), 145.6, 145.1, 141.7, 138.6 (d, J = 2.0 Hz), 137.8, 137.2, 133.2 (d, J = 10.3 Hz), 131.0 (d, J = 4.1 Hz), 130.5, 129.9, 129.6, 128.9, 128.1, 128.0, 127.9 (d, J = 3.1 Hz), 127.7, 127.6, 127.0, 126.2, 123.8 (d, J = 3.4 Hz), 123.1 (d, J = 13.2 Hz), 120.1 (d, J = 4.3 Hz), 116.1, 115.8.

^{19}F NMR (376 MHz, $CDCl_3$) δ -111.67.

HRMS: (ESI) m/z: $[M+K]^+$ Calcd for $C_{26}H_{16}ClFK$ 421.0556; Found 421.0552.

Optical $[\alpha]_{25}^{D}$ = -310.6 ° (c = 0.25, CH_2Cl_2, 85% ee)

HPLC (AD-H, ^{i}PrOH/n-hexane = 5/95, flow rate = 0.5 mL/min, l = 254 nm) t_R = 8.3 min (major), 9.3 min (minor).

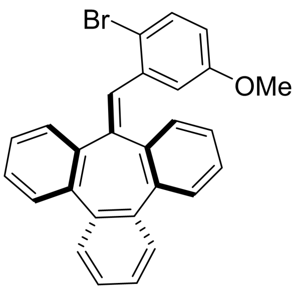

9-(2-Bromo-5-methoxybenzylidene)-9H-tribenzo[a,c,e][7]annulene (3w) was synthesized by following Procedure C. The crude material was purified by column chromatography (SiO_2, petroleum ether: CH_2Cl_2 = 20:1) to provide **3w** as a white solid (47.4 mg, 54% yield).

^{1}H NMR (400 MHz, $CDCl_3$) δ 7.61 (dd, J = 21.8, 6.4 Hz, 4H), 7.54 (d, J = 6.3 Hz, 1H), 7.44 (dd, J = 5.7, 3.5 Hz, 2H), 7.40 – 7.32 (m, 3H), 7.27 – 7.20 (m, 1H), 7.12 (t, J = 7.5 Hz, 1H), 6.98 (d, J = 7.6 Hz, 1H), 6.74 (s, 1H), 6.51 (dd, J = 8.8, 3.1 Hz, 1H), 6.22 (d, J = 3.0 Hz, 1H), 3.29 (s, 3H).

^{13}C NMR (100 MHz, $CDCl_3$) δ 157.9, 145.5, 144.4, 142.0, 138.8, 138.7, 138.0, 137.2, 137.2, 132.7, 130.7, 129.7, 129.2, 128.9, 128.8, 128.1, 128.0, 127.8, 127.7, 127.7, 127.6, 127.4, 126.4, 115.9, 115.4, 115.0, 54.8.

HRMS: (ESI) m/z: $[M+K]^+$ Calcd for $C_{27}H_{19}BrOK$ 477.0251; Found 477.0252.

Optical $[\alpha]_{25}^{D}$ = -67.7 ° (c = 0.25, CH_2Cl_2, 43% ee)

HPLC (OD-H, ^{i}PrOH/n-hexane = 5/95, flow rate = 0.5 mL/min, l = 254 nm) t_R = 10.7 min (major), 10.3 min (minor).

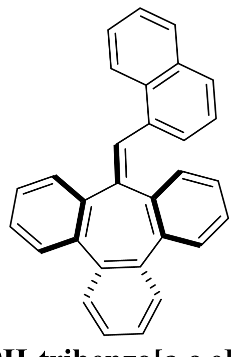

9-(Naphthalen-1-ylmethylene)-9H-tribenzo[a,c,e][7]annulene (3x) was synthesized by following Procedure C. The crude material was purified by column chromatography (SiO_2, petroleum ether: CH_2Cl_2 = 20:1) to provide **3x** as a white solid (42.6 mg, 56% yield).

¹H NMR (400 MHz, $CDCl_3$) δ 8.25 (d, *J* = 8.0 Hz, 1H), 7.91 (d, *J* = 7.8 Hz, 1H), 7.85 (d, *J* = 7.4 Hz, 1H), 7.80 – 7.71 (m, 4H), 7.68 (d, *J* = 7.9 Hz, 1H), 7.64 – 7.49 (m, 6H), 7.33 – 7.24 (m, 3H), 7.10 – 7.01 (m, 2H), 6.95 (d, *J* = 7.6 Hz, 1H).

¹³C NMR (100 MHz, $CDCl_3$) δ 146.0, 145.0, 142.5, 139.1, 138.8, 138.0, 137.5, 133.7, 133.4, 132.1, 130.5, 130.2, 129.2, 129.0, 128.5, 128.1, 127.8, 127.7, 127.6, 127.6, 127.4, 127.4, 127.3, 127.2, 126.6, 125.9, 125.6, 125.2, 124.5.

HRMS: (ESI) m/z: $[M+K]^+$ Calcd for $C_{30}H_{20}K$ 419.1197; Found 419.1199.

Optical $[\alpha]_{25}^{D}$ = -74.47 ° (c = 0.25, CH_2Cl_2, 30% ee)

HPLC (IA-H, ^{i}PrOH/n-hexane = 5/95, flow rate = 0.5 mL/min, l = 254 nm) t_R = 8.7 min (major), 10.3 min (minor).

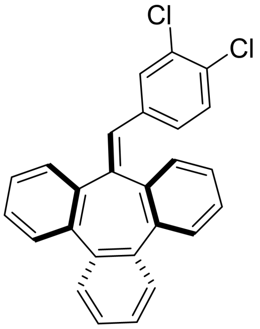

9-(3,4-Dichlorobenzylidene)-9H-tribenzo[a,c,e][7]annulene (3y) was synthesized by following Procedure C. The crude material was purified by column chromatography (SiO_2, petroleum ether) to provide **3y** as a white solid (53.5 mg, 67% yield).

¹H NMR (400 MHz, $CDCl_3$) δ 7.63 (dd, *J* = 7.3, 2.0 Hz, 1H), 7.55 (dd, *J* = 7.9, 1.2 Hz, 1H), 7.52 – 7.46 (m, 2H), 7.37 (ddd, *J* = 6.5, 3.9, 1.9 Hz, 2H), 7.32 (s, 1H), 7.30 – 7.22 (m, 3H), 7.15 – 7.11 (m, 1H), 7.05 – 7.00 (m, 2H), 6.99 – 6.95 (m, 1H), 6.68 (dd, *J* = 8.4, 2.1 Hz, 1H), 6.36 (s, 1H).

¹³C NMR (100 MHz, $CDCl_3$) δ 145.6, 144.6, 141.3, 138.5, 138.5, 137.7, 137.2, 136.6, 131.9, 130.9, 130.5, 130.5, 129.9, 129.8, 129.7, 129.0, 128.2, 128.1, 128.0, 127.8, 127.7, 127.7, 127.0, 126.5, 125.8.

HRMS: (ESI) m/z: $[M+Na]^+$ Calcd for $C_{26}H_{16}Cl_2Na$ 421.0521; Found 421.0519.

Optical $[\alpha]_{25}^{D}$ = -406.2 ° (c = 0.25, CH_2Cl_2, 93% ee)

HPLC (AD-H, ^{i}PrOH/n-hexane = 5/95, flow rate = 0.5 mL/min, l = 254 nm) t_R = 11.1 min (major), 13.0 min (minor).

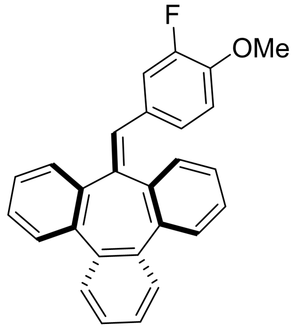

9-(3-Fluoro-4-methoxybenzylidene)-9H-tribenzo[a,c,e][7]annulene (3z) was synthesized by following Procedure C. The crude material was purified by column chromatography (SiO_2, petroleum ether: CH_2Cl_2 = 10:1) to provide **3z** as a white solid (40.8 mg, 54% yield).

^{1}H NMR (400 MHz, $CDCl_3$) δ 7.65 (dd, *J* = 7.2, 2.0 Hz, 1H), 7.57 (dd, *J* = 7.9, 1.3 Hz, 1H), 7.50 (dd, *J* = 6.4, 2.5 Hz, 2H), 7.38 (ddd, *J* = 7.8, 3.5, 1.8 Hz, 3H), 7.33 – 7.26 (m, 3H), 7.18 – 7.16 (m, 1H), 7.08 (dd, *J* = 7.7, 1.4 Hz, 1H), 6.73 – 6.61 (m, 3H), 6.39 (s, 1H), 3.73 (s, 3H).

^{13}C NMR (100 MHz, $CDCl_3$) δ 151.7 (d, *J* = 244.5 Hz), 146.6, 146.5, 146.3, 141.9, 141.8, 138.8 (d, *J* = 4.6 Hz), 137.8, 137.4, 130.4, 129.9, 129.9, 129.8, 128.9, 128.0 (d, *J* = 4.1 Hz), 127.8, 127.6, 127.5, 127.5, 127.2, 125.9, 125.4 (d, *J* = 3.1 Hz), 116.5, 116.3, 112.6, 56.1.

^{19}F NMR (565 MHz, $CDCl_3$) δ -135.11 – -136.16 (m).

HRMS: (ESI) m/z: $[M+K]^+$ Calcd for $C_{27}H_{19}FOK$ 417.1052; Found 417.1063.

Optical $[\alpha]_{25}^{D}$ = -301.7 ° (c = 0.25, CH_2Cl_2, 93% ee)

HPLC (AD-H, ^{i}PrOH/n-hexane = 5/95, flow rate = 0.5 mL/min, l = 254 nm) t_R = 15.8 min (major), 17.9 min (minor).

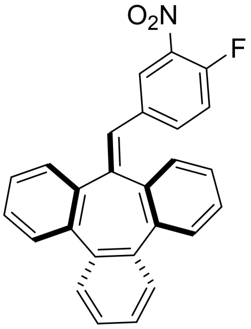

9-(4-Fluoro-3-nitrobenzylidene)-9H-tribenzo[a,c,e][7]annulene (3aa) was synthesized by following Procedure C. The crude material was purified by column

chromatography (SiO_2, petroleum ether: CH_2Cl_2 = 10:1) to provide **3aa** as a white solid (51.1 mg, 65% yield).

^{1}H NMR (400 MHz, $CDCl_3$) δ 7.79 – 7.73 (m, 2H), 7.70 (dd, *J* = 7.9, 1.2 Hz, 1H), 7.65 – 7.60 (m, 2H), 7.54 – 7.48 (m, 2H), 7.45 (s, 1H), 7.44 – 7.41 (m, 2H), 7.40 (d, *J* = 1.3 Hz, 1H), 7.27 – 7.24 (m, 1H), 7.23 – 7.18 (m, 1H), 7.06 (dd, *J* = 7.7, 1.4 Hz, 1H), 7.00 (dd, *J* = 10.6, 8.7 Hz, 1H), 6.55 (s, 1H).

^{13}C NMR (150 MHz, $CDCl_3$) δ 154.0 (d, *J* = 265.8 Hz), 145.7, 145.1, 140.8, 138.5, 138.3, 137.8, 137.1, 136.9 (d, *J* = 7.5 Hz), 135.7 (d, *J* = 8.2 Hz), 133.8 (d, *J* = 4.3 Hz), 130.5, 130.0 (d, *J* = 3.2 Hz), 129.0, 128.4, 128.3, 128.1, 128.1, 127.8, 127.8, 126.7, 126.4 (d, *J* = 2.9 Hz), 125.6, 125.3, 117.9, 117.8.

^{19}F NMR (565 MHz, $CDCl_3$) δ -119.50.

HRMS: (ESI) m/z: $[M+Na]^+$ Calcd for $C_{26}H_{16}FNO_2Na$ 416.1057; Found 416.1060.

Optical $[\alpha]_{25}^{D}$ = -231.6 ° (c = 0.25, CH_2Cl_2, 93% ee)

HPLC (AD-H, ^{i}PrOH/n-hexane = 5/95, flow rate = 0.5 mL/min, l = 254 nm) t_R = 21.4 min (major), 23.5 min (minor).

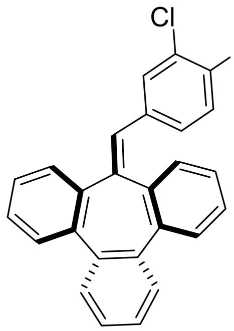

9-(3-Chloro-4-fluorobenzylidene)-9H-tribenzo[a,c,e][7]annulene (3ab) was synthesized by following Procedure C. The crude material was purified by column chromatography (SiO_2, petroleum ether) to provide **3ab** as a white solid (49.7 mg, 65% yield).

^{1}H NMR (600 MHz, $CDCl_3$) δ 7.75 (dd, *J* = 7.5, 1.7 Hz, 1H), 7.67 (dd, *J* = 7.9, 1.3 Hz, 1H), 7.63 – 7.59 (m, 2H), 7.49 (td, *J* = 7.2, 1.7 Hz, 2H), 7.44 (d, *J* = 0.5 Hz, 1H), 7.41 (d, *J* = 2.2 Hz, 1H), 7.40 – 7.39 (m, 1H), 7.37 (dd, *J* = 7.7, 1.4 Hz, 1H), 7.24 (td, *J* = 7.5, 1.3 Hz, 1H), 7.11 (d, *J* = 1.4 Hz, 1H), 7.10 (d, *J* = 1.1 Hz, 1H), 6.88 – 6.82 (m, 2H), 6.49 (s, 1H).

^{13}C NMR (100 MHz, $CDCl_3$) δ 156.8 (d, *J* = 249.5 Hz), 145.7, 143.7 (d, *J* = 1.8 Hz), 141.4, 138.6 (d, *J* = 3.2 Hz), 137.8, 137.3, 133.8 (d, *J* = 4.1 Hz), 131.2, 130.5, 129.9,

129.8, 129.0, 128.8 (d, J = 6.8 Hz), 128.1 (d, J = 3.7 Hz), 128.0, 127.8, 127.7 (d, J = 4.7 Hz), 127.0, 126.5, 125.8, 120.5, 120.3, 116.1, 115.9.

^{19}F NMR (376 MHz, $CDCl_3$) δ -117.27.

HRMS: (ESI) m/z: $[M+K]^+$ Calcd for $C_{26}H_{16}ClFK$ 421.0556; Found 421.0564.

Optical $[\alpha]_{25}^{D}$ = -284.0 ° (c = 0.25, CH_2Cl_2, 94% ee)

HPLC (AD-H, ^{i}PrOH/n-hexane = 5/95, flow rate = 0.5 mL/min, l = 254 nm) t_R = 10.2 min (major), 12.7 min (minor).

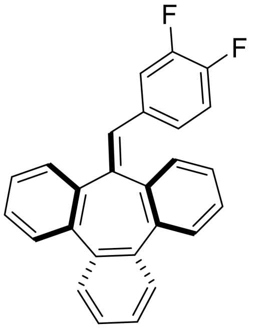

9-(3,4-Difluorobenzylidene)-9H-tribenzo[a,c,e][7]annulene (3ac) was synthesized by following Procedure C. The crude material was purified by column chromatography (SiO_2, petroleum ether) to provide **3ac** as a yellow solid (48.3 mg, 66% yield).

^{1}H NMR (400 MHz, $CDCl_3$) δ 7.65 (dd, J = 7.3, 1.9 Hz, 1H), 7.57 (dd, J = 7.8, 1.3 Hz, 1H), 7.54 – 7.48 (m, 2H), 7.42 – 7.34 (m, 3H), 7.33 – 7.25 (m, 3H), 7.18 – 7.13 (m, 1H), 7.02 (dd, J = 7.7, 1.5 Hz, 1H), 6.80 (dd, J = 10.2, 8.3 Hz, 1H), 6.76 – 6.64 (m, 2H), 6.39 (s, 1H).

^{13}C NMR (100 MHz, $CDCl_3$) δ 149.8 (dd, J = 246.8, 12.6 Hz), 149.2 (dd, J = 248.9, 12.8 Hz), 145.8, 143.6 (d, J = 1.9 Hz), 141.4, 138.6 (d, J = 2.1 Hz), 137.7, 137.3, 133.6 (dd, J = 6.2, 4.0 Hz), 130.4, 129.9 (d, J = 14.3 Hz), 129.0, 128.1, 128.0 (d, J = 4.5 Hz), 127.8, 127.6 (d, J = 4.8 Hz), 127.0, 126.8 (d, J = 1.9 Hz), 125.8, 125.5 (dd, J = 6.1, 3.4 Hz), 117.6, 117.5, 116.8, 116.6.

^{19}F NMR (565 MHz, $CDCl_3$) δ -138.10 (dt, J = 21.4, 10.3 Hz), -139.15 – -139.26 (m).

HRMS: (ESI) m/z: $[M+H]^+$ Calcd for $C_{26}H_{17}F_2$ 367.1293; Found 367.1304.

Optical $[\alpha]_{25}^{D}$ = -231.1 ° (c = 0.25, CH_2Cl_2, 93% ee)

HPLC (AD-H, ^{i}PrOH/n-hexane = 5/95, flow rate = 0.5 mL/min, l = 254 nm) t_R = 10.5 min (major), 11.6 min (minor).

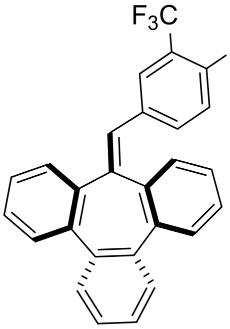

9-(4-Fluoro-3-(trifluoromethyl)benzylidene)-9H-tribenzo[a,c,e][7]annulene (3ad) was synthesized by following Procedure C. The crude material was purified by column chromatography (SiO_2, petroleum ether) to provide **3ad** as a white oil (51.6 mg, 62% yield).

^{1}H NMR (400 MHz, $CDCl_3$) δ 7.67 – 7.62 (m, 1H), 7.58 (dd, *J* = 7.9, 1.2 Hz, 1H), 7.54 – 7.49 (m, 2H), 7.42 – 7.37 (m, 2H), 7.35 (s, 1H), 7.33 – 7.29 (m, 2H), 7.27 (dd, *J* = 7.7, 1.4 Hz, 1H), 7.19 (dd, *J* = 7.0, 2.3 Hz, 1H), 7.13 (td, *J* = 7.5, 1.3 Hz, 1H), 7.05 – 7.00 (m, 1H), 6.96 (dd, *J* = 7.6, 1.4 Hz, 1H), 6.81 (t, *J* = 9.4 Hz, 1H), 6.44 (s, 1H).

^{13}C NMR (100 MHz, $CDCl_3$) δ 158.2 (d, *J* = 257.0 Hz), 145.5, 144.3, 141.3, 138.6, 138.5, 137.8, 137.3, 134.1 (d, *J* = 8.1 Hz), 133.0 (d, *J* = 3.9 Hz), 130.5, 129.9, 129.8, 129.0, 128.2, 128.1, 127.9 (d, *J* = 3.6 Hz), 127.7 (d, *J* = 6.8 Hz), 126.9, 126.3, 125.7, 122.4 (q, *J* = 271.8 Hz), 117.8 (dd, *J* = 32.7, 12.6 Hz), 116.5, 116.3.

^{19}F NMR (565 MHz, $CDCl_3$) δ -61.56 (d, *J* = 13.2 Hz), -116.55 (td, *J* = 15.1, 12.9, 5.9 Hz).

HRMS: (ESI) m/z: $[M+K]^+$ Calcd for $C_{27}H_{16}F_4K$ 455.0820; Found 455.0827.

Optical $[\alpha]_{25}^{D}$ = -233.8 ° (c = 0.25, CH_2Cl_2, 93% ee)

HPLC (AD-H, ^{i}PrOH/n-hexane = 5/95, flow rate = 0.5 mL/min, l = 254 nm) t_R = 8.9 min (major), 10.0 min (minor).

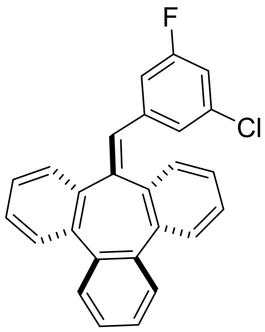

9-(3-Chloro-5-fluorobenzylidene)-9H-tribenzo[a,c,e][7]annulene (3ae) was synthesized by following Procedure C. The crude material was purified by column chromatography (SiO_2, petroleum ether) to provide **3ae** as a white solid (49.7 mg, 65% yield).

^{1}H NMR (400 MHz, $CDCl_3$) δ 7.65 (dd, *J* = 7.3, 1.9 Hz, 1H), 7.56 (dd, *J* = 7.8, 1.2 Hz, 1H), 7.50 (dt, *J* = 5.0, 2.9 Hz, 2H), 7.38 (td, *J* = 5.0, 2.4 Hz, 2H), 7.34 – 7.26 (m, 4H), 7.17 – 7.14 (m, 1H), 7.00 (dd, *J* = 7.6, 1.4 Hz, 1H), 6.77 – 6.69 (m, 2H), 6.47 (d, *J* = 10.0 Hz, 1H), 6.37 (s, 1H).

^{13}C NMR (100 MHz, $CDCl_3$) δ 162.2 (d, *J* = 248.2 Hz), 145.5, 145.4, 141.1, 139.8 (d, *J* = 8.9 Hz), 138.5 (d, *J* = 1.8 Hz), 137.5, 137.2, 134.4 (d, *J* = 11.2 Hz), 130.4, 130.0, 129.8, 129.0, 128.2 (d, *J* = 4.6 Hz), 128.1, 127.9, 127.7 (d, *J* = 1.7 Hz), 126.9, 126.5 (d, *J* = 2.6 Hz), 125.7, 125.3 (d, *J* = 3.0 Hz), 114.6, 114.4, 114.2, 113.9.

^{19}F NMR (376 MHz, $CDCl_3$) δ -113.67.

HRMS: (ESI) m/z: $[M+K]^+$ Calcd for $C_{26}H_{16}ClFK$ 421.0556; Found 421.0553.

Optical $[\alpha]_{25}^{D}$ = +268.7 ° (c = 0.25, CH_2Cl_2, 90% ee)

HPLC (OJ-H, ^{i}PrOH/n-hexane = 5/95, flow rate = 0.5 mL/min, l = 254 nm) t_R = 10.1 min (major), 12.7 min (minor).

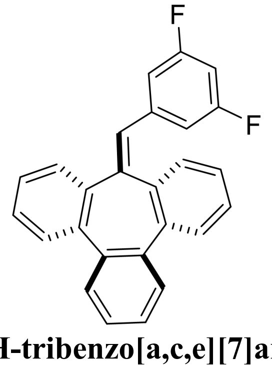

9-(3,5-Difluorobenzylidene)-9H-tribenzo[a,c,e][7]annulene (3af) was synthesized by following Procedure C. The crude material was purified by column chromatography (SiO_2, petroleum ether) to provide **3af** as a white solid (49.1 mg, 67% yield).

^{1}H NMR (400 MHz, $CDCl_3$) δ 7.65 (dd, *J* = 7.4, 1.9 Hz, 1H), 7.56 (dd, *J* = 7.8, 1.3 Hz, 1H), 7.53 – 7.48 (m, 2H), 7.41 – 7.33 (m, 3H), 7.32 – 7.25 (m, 3H), 7.17 – 7.13 (m, 1H), 7.02 (dd, *J* = 7.7, 1.4 Hz, 1H), 6.44 (d, *J* = 7.9 Hz, 3H), 6.39 (s, 1H).

^{13}C NMR (150 MHz, $CDCl_3$) δ 162.6 (d, *J* = 247.0 Hz), 162.5 (d, *J* = 247.1 Hz), 145.5, 145.2, 141.2, 139.8 (t, *J* = 9.7 Hz), 138.5 (d, *J* = 5.7 Hz), 137.5, 137.2, 130.4,

130.0, 129.8, 129.0, 128.2 (d, J = 4.7 Hz), 128.1, 127.9, 127.7 (d, J = 3.4 Hz), 126.9, 126.8 (d, J = 3.0 Hz), 125.7, 111.8 (dd, J = 20.5, 5.2 Hz), 102.4, 102.3, 102.1.

^{19}F NMR (565 MHz, $CDCl_3$) δ -110.53.

HRMS: (ESI) m/z: $[M+H]^+$ Calcd for $C_{26}H_{17}F_2$ 367.1293; Found 367.1291.

Optical $[\alpha]_{25}^{D}$ = +290.0 ° (c = 0.25, CH_2Cl_2, 90% ee)

HPLC (OJ-H, ^{i}PrOH/n-hexane = 5/95, flow rate = 0.5 mL/min, l = 254 nm) t_R = 10.2 min (major), 13.1 min (minor).

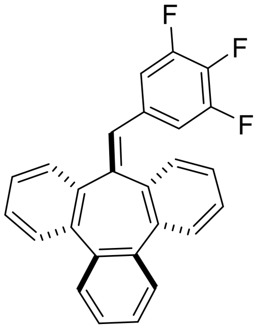

9-(3,4,5-Trifluorobenzylidene)-9H-tribenzo[a,c,e][7]annulene (3ag) was synthesized by following Procedure C. The crude material was purified by column chromatography (SiO_2, petroleum ether) to provide **3ag** as a white solid (47.6 mg, 62% yield).

^{1}H NMR (400 MHz, $CDCl_3$) δ 7.64 (dd, J = 7.3, 1.9 Hz, 1H), 7.56 (dd, J = 7.9, 1.2 Hz, 1H), 7.50 (dt, J = 5.6, 3.2 Hz, 2H), 7.41 – 7.35 (m, 2H), 7.33 – 7.26 (m, 4H), 7.17 – 7.13 (m, 1H), 6.99 (dd, J = 7.6, 1.4 Hz, 1H), 6.51 (dd, J = 8.9, 6.6 Hz, 2H), 6.31 (s, 1H).

^{13}C NMR (100 MHz, $CDCl_3$) δ 150.7 (dd, J = 248.3, 4.4 Hz),150.6 (dd, J = 248.6, 4.3 Hz), 145.4, 145.0, 140.9, 139.8, 138.4 (d, J = 6.8 Hz), 137.6, 137.2, 132.6 (td, J = 7.9, 4.6 Hz), 130.5, 129.9 (d, J = 7.0 Hz), 129.0, 128.3 (d, J = 5.8 Hz), 128.1, 128.0, 127.7 (d, J = 4.8 Hz), 126.8, 125.9 (d, J = 2.3 Hz), 125.7, 113.0 (d, J = 5.8 Hz), 112.9 (d, J = 5.8 Hz).

^{19}F NMR (376 MHz, $CDCl_3$) δ -135.22 (d, J = 20.7 Hz), -161.98 (t, J = 20.6 Hz).

HRMS: (ESI) m/z: $[M+K]^+$ Calcd for $C_{26}H_{15}F_3K$ 423.0757; Found 423.0754.

Optical $[\alpha]_{25}^{D}$ = +263.5 ° (c = 0.25, CH_2Cl_2, 90% ee)

HPLC (OJ-H, ^{i}PrOH/n-hexane = 5/95, flow rate = 0.5 mL/min, l = 254 nm) t_R = 10.5 min (major), 12.5 min (minor).

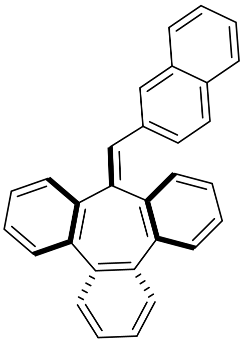

9-(Naphthalen-2-ylmethylene)-9H-tribenzo[a,c,e][7]annulene (3ah) was synthesized by following Procedure C. The crude material was purified by column chromatography (SiO_2, petroleum ether) to provide **3ah** as a white solid (49.4 mg, 65% yield).

^{1}H NMR (400 MHz, $CDCl_3$) δ 7.67 (dd, *J* = 7.5, 1.7 Hz, 1H), 7.59 – 7.56 (m, 2H), 7.53 (d, *J* = 1.5 Hz, 1H), 7.51 (d, *J* = 1.9 Hz, 1H), 7.49 (d, *J* = 1.8 Hz, 2H), 7.42 (d, *J* = 7.1 Hz, 2H), 7.39 – 7.37 (m, 1H), 7.37 – 7.35 (m, 1H), 7.34 (dd, *J* = 6.5, 1.6 Hz, 1H), 7.29 (d, *J* = 2.2 Hz, 1H), 7.29 – 7.27 (m, 1H), 7.26 (d, *J* = 2.0 Hz, 1H), 7.25 – 7.22 (m, 1H), 7.06 (dd, *J* = 6.8, 1.5 Hz, 2H), 6.97 (dd, *J* = 8.6, 1.8 Hz, 1H), 6.66 (s, 1H).

^{13}C NMR (100 MHz, $CDCl_3$) δ 146.4, 142.8, 142.3, 138.9, 138.8, 137.9, 137.4, 134.2, 133.2, 132.3, 130.4, 130.0, 129.6, 129.2, 128.9, 128.7, 128.0, 127.9, 127.9, 127.7, 127.6, 127.5, 127.5, 127.1, 126.8, 126.1, 125.9, 125.8.

HRMS: (ESI) m/z: $[M+K]^+$ Calcd for $C_{30}H_{20}K$ 419.1197; Found 419.1204.

Optical $[\alpha]_{25}^{D}$ = -433.9 ° (c = 0.25, CH_2Cl_2, 90% ee)

HPLC (AD-H, ^{i}PrOH/n-hexane = 5/95, flow rate = 0.5 mL/min, l = 254 nm) t_R = 14.8 min (major), 22.2 min (minor).

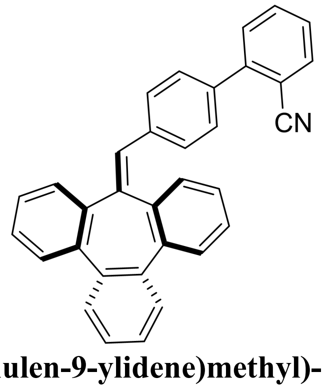

4'-((9H-tribenzo[a,c,e][7]annulen-9-ylidene)methyl)-[1,1'-biphenyl]-2-carbonitrile (3ai) was synthesized by following Procedure C. The crude material was purified

by column chromatography (SiO_2, petroleum ether: EtOAc = 20:1) to provide **3ai** as a white solid (70.7 mg, 82% yield).

^{1}H NMR (400 MHz, $CDCl_3$) δ 7.64 (dd, *J* = 7.3, 2.0 Hz, 1H), 7.60 – 7.55 (m, 2H), 7.52 – 7.47 (m, 2H), 7.42 (dd, *J* = 7.7, 1.4 Hz, 1H), 7.40 – 7.33 (m, 3H), 7.30 (d, *J* = 1.2 Hz, 1H), 7.28 (d, *J* = 1.9 Hz, 2H), 7.26 (d, *J* = 1.4 Hz, 1H), 7.24 (d, *J* = 1.5 Hz, 1H), 7.23 (s, 1H), 7.21 (s, 1H), 7.15 – 7.12 (m, 2H), 7.07 (s, 1H), 7.05 (s, 1H), 6.51 (s, 1H).

^{13}C NMR (150 MHz, $CDCl_3$) δ 146.2, 144.9, 143.5, 142.0, 138.7, 138.6, 137.6, 137.3, 136.9, 136.3, 133.7, 132.7, 130.4, 129.9, 129.8, 129.7, 129.4, 128.9, 128.3, 128.2, 128.0, 128.0, 127.8, 127.6, 127.6, 127.3, 127.2, 125.9, 118.7, 110.8.

HRMS: (ESI) m/z: $[M+Na]^+$ Calcd for $C_{33}H_{21}NNa$ 454.1566; Found 454.1572.

Optical $[\alpha]_{25}^{D}$ = -339.0 ° (c = 0.25, CH_2Cl_2, 94% ee)

HPLC (AD-H, ^{i}PrOH/n-hexane = 10/90, flow rate = 0.5 mL/min, l = 254 nm) t_R = 34.9 min (major), 39.3 min (minor).

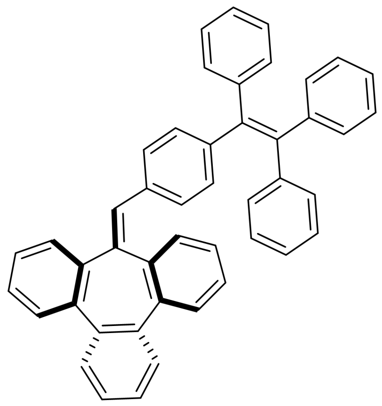

9-(4-(1,2,2-Triphenylvinyl)benzylidene)-9H-tribenzo[a,c,e][7]annulene (3aj) was synthesized by following Procedure C. The crude material was purified by column chromatography (SiO_2, petroleum ether: CH_2Cl_2 = 20:1) to provide **3aj** as a yellow solid (93.5 mg, 80% yield).

^{1}H NMR (400 MHz, $CDCl_3$) δ 7.58 – 7.54 (m, 1H), 7.50 – 7.44 (m, 3H), 7.35 – 7.30 (m, 3H), 7.25 (dd, *J* = 6.5, 3.0 Hz, 2H), 7.19 (d, *J* = 1.6 Hz, 1H), 7.09 – 7.02 (m, 2H), 6.99 (d, *J* = 2.9 Hz, 3H), 6.95 (t, *J* = 2.4 Hz, 6H), 6.92 – 6.86 (m, 6H), 6.72 – 6.67 (m, 4H), 6.39 (s, 1H).

^{13}C NMR (100 MHz, $CDCl_3$) δ 146.5, 143.7, 143.6, 143.6, 142.4, 142.3, 142.1, 140.9, 140.6, 138.9, 138.7, 137.7, 137.3, 134.6, 131.3, 131.3, 131.3, 130.9, 130.3, 129.9,

129.6, 129.0, 128.9, 128.5, 127.9, 127.8, 127.6, 127.6, 127.5, 127.5, 127.4, 127.4, 126.4, 126.4, 126.3, 126.0.

HRMS: (ESI) m/z: $[M+K]^+$ Calcd for $C_{46}H_{32}K$ 623.2136; Found 623.2134.

Optical $[\alpha]_{25}^{D}$ = -323.3 ° (c = 0.25, CH_2Cl_2, 88% ee)

HPLC (AD-H, ^{i}PrOH/n-hexane = 5/95, flow rate = 0.5 mL/min, l = 254 nm) t_R = 9.0 min (major), 12.8 min (minor).

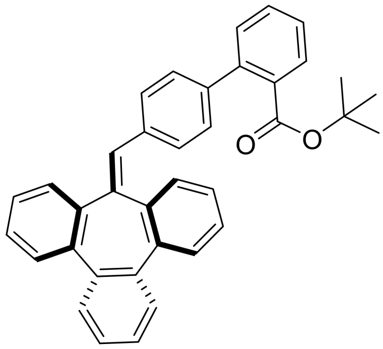

Tert-butyl 4'-((9H-tribenzo[a,c,e][7]annulen-9-ylidene)methyl)-[1,1'-biphenyl]-2-carboxylate (3ak) was synthesized by following Procedure C. The crude material was purified by column chromatography (SiO_2, petroleum ether: EtOAc = 20:1) to provide **3ak** as a white solid (64.8 mg, 64% yield).

^{1}H NMR (400 MHz, $CDCl_3$) δ 7.64 (dd, *J* = 7.8, 1.4 Hz, 2H), 7.56 (d, *J* = 7.8 Hz, 1H), 7.50 (dd, *J* = 6.6, 2.4 Hz, 2H), 7.41 (dd, *J* = 7.2, 1.8 Hz, 1H), 7.38 – 7.34 (m, 2H), 7.34 – 7.27 (m, 3H), 7.27 – 7.23 (m, 2H), 7.15 (s, 1H), 7.12 – 7.08 (m, 2H), 6.99 (s, 4H), 6.53 (s, 1H), 1.16 (s, 9H).

^{13}C NMR (100 MHz, $CDCl_3$) δ 168.1, 146.4, 142.4, 142.3, 141.5, 140.4, 138.9, 138.8, 137.8, 137.4, 135.2, 132.8, 130.5, 130.4, 130.3, 129.9, 129.7, 129.6, 128.9, 128.8, 128.7, 128.2, 128.0, 128.0, 127.6, 127.5, 127.5, 127.5, 127.3, 127.0, 126.0, 81.3, 27.6.

HRMS: (ESI) m/z: $[M+Na]^+$ Calcd for $C_{37}H_{30}O_2Na$ 529.2138; Found 529.2137.

Optical $[\alpha]_{25}^{D}$ = -294.3 ° (c = 0.25, CH_2Cl_2, 90% ee)

HPLC (AD-H, ^{i}PrOH/n-hexane = 10/90, flow rate = 0.5 mL/min, l = 254 nm) t_R = 10.2 min (major), 13.2 min (minor).

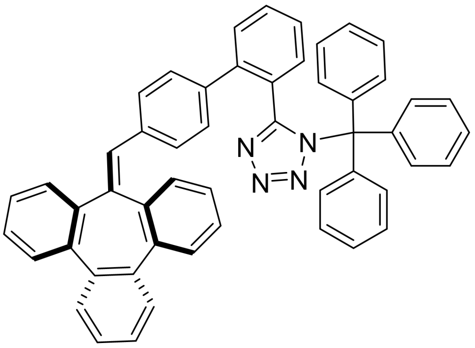

5-(4'-((9H-tribenzo[a,c,e][7]annulen-9-ylidene)methyl)-[1,1'-biphenyl]-2-yl)-1-trityl-1H-tetrazole (3al) was synthesized by following Procedure C. The crude material was purified by column chromatography (SiO_2, petroleum ether: EtOAc = 20:1) to provide **3al** as a yellow oil (74.5 mg, 52% yield).

^{1}H NMR (400 MHz, $CDCl_3$) δ 7.78 (dd, J = 7.4, 1.7 Hz, 1H), 7.65 – 7.61 (m, 1H), 7.60 – 7.55 (m, 2H), 7.52 (d, J = 1.9 Hz, 1H), 7.39 (dd, J = 6.8, 3.3 Hz, 3H), 7.35 – 7.32 (m, 2H), 7.25 – 7.23 (m, 2H), 7.22 (d, J = 2.5 Hz, 7H), 7.09 – 7.04 (m, 8H), 6.79 (s, 6H), 6.77 (s, 2H), 6.40 (s, 1H).

^{13}C NMR (100 MHz, $CDCl_3$) δ 164.0, 146.8, 146.6, 142.2, 142.2, 141.9, 141.1, 139.6, 138.9, 138.8, 137.6, 137.4, 134.9, 130.4, 130.4, 130.2, 130.1, 129.9, 129.8, 129.7, 128.9, 128.8, 128.8, 128.1, 128.0, 128.0, 127.9, 127.7, 127.5, 127.3, 127.2, 126.3, 126.0, 82.8.

HRMS: (ESI) m/z: $[M+Na]^+$ Calcd for $C_{52}H_{36}N_4Na$ 739.2832; Found 739.2845.

Optical $[\alpha]_{25}^{D}$ = -108.1 ° (c = 0.25, CH_2Cl_2, 90% ee)

HPLC (AD-H, ^{i}PrOH/n-hexane = 10/90, flow rate = 0.5 mL/min, l = 254 nm) t_R = 28.7 min (major), 19.8 min (minor).

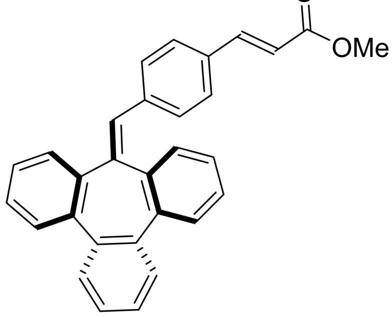

Methyl (E)-3-(4-((9H-tribenzo[a,c,e][7]annulen-9-ylidene)methyl)phenyl)acrylate (3am) was synthesized by following Procedure C. The crude material was purified by column chromatography (SiO_2, petroleum ether: EtOAc = 20:1) to provide **3am** as a white solid (37.3 mg, 45% yield).

^{1}H NMR (400 MHz, $CDCl_3$) δ 7.65 (dd, *J* = 7.2, 2.0 Hz, 1H), 7.58 (dd, *J* = 7.8, 1.2 Hz, 1H), 7.53 – 7.46 (m, 3H), 7.42 – 7.35 (m, 3H), 7.32 – 7.26 (m, 3H), 7.20 (s, 1H), 7.18 (s, 1H), 7.16 – 7.12 (m, 1H), 7.05 (dd, *J* = 7.6, 1.4 Hz, 1H), 6.98 (s, 1H), 6.96 (s, 1H), 6.49 (s, 1H), 6.25 (d, *J* = 16.0 Hz, 1H), 3.69 (s, 3H).

^{13}C NMR (150 MHz, $CDCl_3$) δ 167.5, 146.1, 144.4, 144.0, 142.0, 138.7, 138.7, 138.6, 137.7, 137.2, 132.8, 130.4, 129.9, 129.7, 129.6, 128.9, 128.3, 128.0, 128.0, 127.9, 127.8, 127.7, 127.6, 127.6, 127.2, 125.9, 117.2, 51.7.

HRMS: (ESI) m/z: $[M+Na]^+$ Calcd for $C_{30}H_{22}O_2Na$ 437.1512; Found 437.1516.

Optical $[\alpha]_{25}^{D}$ = -350.9 ° (c = 0.25, CH_2Cl_2, 93% ee)

HPLC (AD-H, ^{i}PrOH/n-hexane = 10/90, flow rate = 0.5 mL/min, l = 254 nm) t_R = 18.1 min (major), 26.9 min (minor).

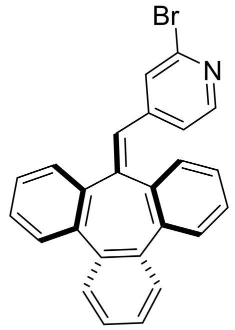

4-((9H-tribenzo[a,c,e][7]annulen-9-ylidene)methyl)-2-bromopyridine (3an) was synthesized by following Procedure C. The crude material was purified by column chromatography (SiO_2, petroleum ether: CH_2Cl_2 = 3:1) to provide **3an** as a yellow oil (52.5 mg, 64% yield).

^{1}H NMR (400 MHz, $CDCl_3$) δ 7.92 (d, *J* = 5.2 Hz, 1H), 7.62 (dd, *J* = 7.3, 1.9 Hz, 1H), 7.56 (dd, *J* = 7.9, 1.2 Hz, 1H), 7.51 – 7.46 (m, 2H), 7.37 (td, *J* = 4.7, 2.2 Hz, 2H), 7.31 – 7.26 (m, 4H), 7.14 – 7.11 (m, 1H), 7.02 (d, *J* = 1.4 Hz, 1H), 6.93 (dd, *J* = 7.6, 1.4 Hz, 1H), 6.63 (dd, *J* = 5.2, 1.5 Hz, 1H), 6.30 (s, 1H).

^{13}C NMR (100 MHz, $CDCl_3$) δ 149.5, 148.8, 147.0, 144.9, 142.2, 140.6, 138.2, 138.1, 137.3, 136.9, 130.5, 129.9, 129.8, 129.0, 128.5, 128.2, 128.2, 128.1, 127.8, 127.8, 127.8, 126.6, 125.5, 125.0, 122.3.

HRMS: (ESI) m/z: $[M+H]^+$ Calcd for $C_{25}H_{17}BrN$ 410.0539; Found 410.0546.

Optical $[\alpha]_{25}^{D}$ = -241.8 ° (c = 0.25, CH_2Cl_2, 90% ee)

HPLC (AD-H, ^{i}PrOH/n-hexane = 10/90, flow rate = 0.5 mL/min, l = 254 nm) t_R = 12.9 min (major), 14.4 min (minor).

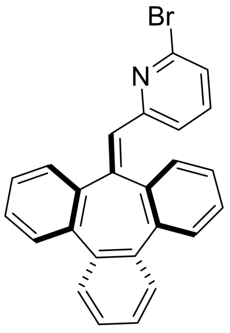

2-((9H-tribenzo[a,c,e][7]annulen-9-ylidene)methyl)-6-bromopyridine (3ao) was synthesized by following Procedure C. The crude material was purified by column chromatography (SiO_2, petroleum ether: CH_2Cl_2 = 5:1) to provide **3ao** as a white solid (50.8 mg, 62% yield).

^{1}H NMR (400 MHz, $CDCl_3$) δ 7.66 – 7.62 (m, 1H), 7.58 (dd, *J* = 7.8, 1.2 Hz, 1H), 7.55 – 7.49 (m, 2H), 7.46 – 7.43 (m, 1H), 7.41 – 7.36 (m, 2H), 7.32 – 7.29 (m, 2H), 7.27 (dd, *J* = 7.6, 1.5 Hz, 1H), 7.12 (td, *J* = 7.5, 1.2 Hz, 1H), 7.08 (dd, *J* = 7.8, 1.0 Hz, 1H), 7.05 – 7.00 (m, 2H), 6.64 (s, 1H), 6.57 (dd, *J* = 7.6, 1.0 Hz, 1H).

^{13}C NMR (100 MHz, $CDCl_3$) δ 157.1, 147.3, 145.1, 141.2, 141.1, 138.5, 138.2, 137.6, 137.5, 136.8, 130.6, 129.8, 129.7, 128.9, 128.6, 128.1, 128.1, 128.0, 127.8, 127.6, 127.1, 125.8, 125.5, 122.6.

HRMS: (ESI) m/z: $[M+H]^+$ Calcd for $C_{25}H_{17}BrN$ 410.0539; Found 410.0542.

Optical $[\alpha]_{25}^{D}$ = -284.5 ° (c = 0.25, CH_2Cl_2, 90% ee)

HPLC (AD-H, ^{i}PrOH/n-hexane = 5/95, flow rate = 0.5 mL/min, l = 254 nm) t_R = 17.7 min (major), 19.8 min (minor).

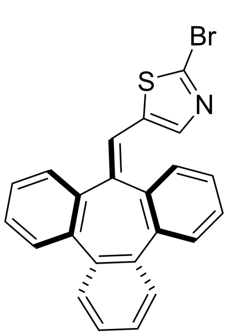

5-((9H-tribenzo[a,c,e][7]annulen-9-ylidene)methyl)-2-bromothiazole (3ap) was synthesized by following Procedure C. The crude material was purified by column chromatography (SiO_2, petroleum ether: CH_2Cl_2 = 5:1) to provide **3ap** as a white solid (49.9 mg, 60% yield).

^{1}H NMR (400 MHz, $CDCl_3$) δ 7.62 – 7.58 (m, 2H), 7.52 – 7.46 (m, 2H), 7.39 (d, J = 1.7 Hz, 1H), 7.37 – 7.34 (m, 2H), 7.31 (d, J = 2.0 Hz, 2H), 7.30 – 7.24 (m, 4H), 6.55 (s, 1H).

^{13}C NMR (100 MHz, $CDCl_3$) δ 145.0, 143.9, 143.0, 140.1, 138.5, 138.3, 138.0, 137.6, 137.2, 136.1, 130.6, 130.2, 129.8, 129.1, 129.0, 128.3, 128.0, 128.0, 127.8, 127.7, 127.3, 125.2, 118.4.

HRMS: (ESI) m/z: $[M+H]^+$ Calcd for $C_{23}H_{15}BrNS$ 416.0103; Found 416.0107.

Optical $[\alpha]_{25}^{D}$ = -150.8 ° (c = 0.25, CH_2Cl_2, 86% ee)

HPLC (OX-H, ^{i}PrOH/n-hexane = 5/95, flow rate = 0.5 mL/min, l = 254 nm) t_R = 22.9 min (major), 17.9 min (minor).

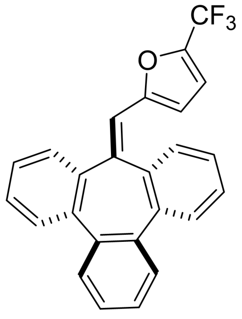

2-((9H-tribenzo[a,c,e][7]annulen-9-ylidene)methyl)-5-(trifluoromethyl)furan (3aq) was synthesized by following Procedure C. The crude material was purified by column chromatography (SiO_2, petroleum ether) to provide **3aq** as a white oil (52.0 mg, 67% yield).

^{1}H NMR (400 MHz, $CDCl_3$) δ 7.69 (td, J = 6.9, 2.8 Hz, 2H), 7.63 – 7.58 (m, 2H), 7.49 – 7.43 (m, 3H), 7.43 (s, 1H), 7.40 (dd, J = 5.8, 3.1 Hz, 3H), 7.38 – 7.33 (m, 1H), 6.57 (dd, J = 3.4, 1.6 Hz, 1H), 6.46 (s, 1H), 5.88 (d, J = 3.6 Hz, 1H).

^{13}C NMR (150 MHz, $CDCl_3$) δ 154.3, 145.1, 144.3, 141.1, 140.0 (q, J = 42.5 Hz), 138.3, 138.2, 137.2, 137.0, 130.5, 130.0, 129.8, 129.0, 128.3, 128.1 (d, J = 3.8 Hz), 127.8, 127.7, 127.6, 127.0, 125.6, 119.1 (q, J = 266.6 Hz), 116.5, 112.8 (d, J = 3.0 Hz), 108.7.

^{19}F NMR (565 MHz, $CDCl_3$) δ -63.84.

HRMS: (ESI) m/z: $[M+H]^+$ Calcd for $C_{25}H_{16}F_3O$ 389.1148; Found 389.1155.

Optical $[\alpha]_{25}^{D}$ = +8.9 ° (c = 0.25, CH_2Cl_2, 88% ee)

HPLC (AD-H, ^{i}PrOH/n-hexane = 5/95, flow rate = 0.5 mL/min, l = 254 nm) t_R = 9.7 min (major), 9.0 min (minor).

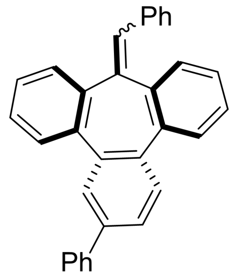

9-Benzylidene-2-phenyl-9H-tribenzo[a,c,e][7]annulene (3ar) was synthesized by following Procedure C. The crude material was purified by column chromatography (SiO_2, petroleum ether) to provide **3ar** as a white solid (55.3 mg, 68% yield).

^{1}H NMR (400 MHz, $CDCl_3$) δ 7.81 (d, J = 52.6 Hz, 1H), 7.66 (d, J = 2.0 Hz, 1H), 7.64 (d, J = 3.1 Hz, 1H), 7.62 (d, J = 1.7 Hz, 1H), 7.60 (d, J = 1.3 Hz, 2H), 7.57 – 7.52 (m, 1H), 7.41 (d, J = 7.2 Hz, 2H), 7.38 (s, 1H), 7.31 (dd, J = 6.5, 2.4 Hz, 3H), 7.29 – 7.27 (m, 1H), 7.14 (d, J = 1.2 Hz, 1H), 7.10 – 7.08 (m, 1H), 7.05 – 6.97 (m, 5H), 6.53 (d, J = 3.2 Hz, 1H).

^{13}C NMR (150 MHz, $CDCl_3$) δ 146.3, 146.3, 142.4, 142.3, 142.2, 140.7, 140.6, 140.3, 140.3, 139.2, 139.1, 137.9, 137.8, 137.8, 137.5, 137.4, 137.1, 136.4, 130.9, 130.4, 129.7, 129.5, 129.2, 129.2, 129.2, 128.9, 128.8, 128.8, 128.8, 128.7, 128.1, 128.1, 128.1, 128.0, 127.9, 127.7, 127.6, 127.5, 127.4, 127.2, 127.2, 126.9, 126.2, 126.1, 126.1.

HRMS: (ESI) m/z: $[M+K]^+$ Calcd for $C_{32}H_{22}K$ 445.1353; Found 445.1354.

Optical $[\alpha]_{25}^{D}$ = -260.3 ° (c = 0.25, CH_2Cl_2, 91% ee (major), 89% ee (minor), d.r.= 1.4:1)

HPLC (AD-H, ^{i}PrOH/n-hexane = 5/95, flow rate = 0.5 mL/min, l = 254 nm) t_R = 19.8 min (major), 15.0 min (minor).

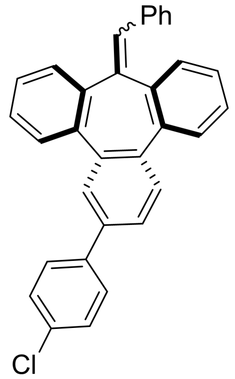

9-Benzylidene-2-(4-chlorophenyl)-9H-tribenzo[a,c,e][7]annulene (3as) was synthesized by following Procedure C. The crude material was purified by column chromatography (SiO_2, petroleum ether) to provide **3as** as a white solid (61.7 mg, 70% yield).

^{1}H NMR (400 MHz, $CDCl_3$) δ 7.93 – 7.80 (m, 1H), 7.75 – 7.70 (m, 1H), 7.65 (dd, J = 16.2, 8.5 Hz, 5H), 7.53 – 7.50 (m, 1H), 7.48 – 7.40 (m, 4H), 7.38 (s, 1H), 7.26 – 7.17 (m, 2H), 7.16 – 7.10 (m, 3H), 7.09 (dd, J = 4.2, 2.2 Hz, 2H), 6.63 (d, J = 2.6 Hz, 1H).

^{13}C NMR (100 MHz, $CDCl_3$) δ 146.3, 142.3, 142.2, 139.3, 139.1, 139.0, 138.1, 137.6, 137.2, 136.9, 136.4, 133.6, 131.0, 130.5, 129.6, 129.5, 129.3, 129.2, 129.0, 128.8, 128.4, 128.4, 128.4, 128.2, 128.2, 128.0, 127.7, 127.6, 127.5, 127.0, 126.2, 126.0.

HRMS: (ESI) m/z: $[M+K]^+$ Calcd for $C_{32}H_{21}ClK$ 479.0963; Found 479.0968.

Optical $[\alpha]_{25}^{D}$ = -247.5 ° (c = 0.25, CH_2Cl_2, 90% ee (minor), 92% ee (major), d.r.= 1.2:1)

HPLC (AD-H, ^{i}PrOH/n-hexane = 5/95, flow rate = 0.5 mL/min, l = 254 nm) t_R = 18.2 min (major), 19.1 min (minor).

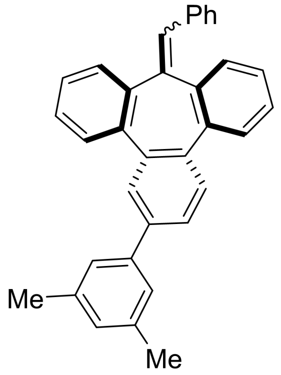

9-Benzylidene-2-(3,5-dimethylphenyl)-9H-tribenzo[a,c,e][7]annulene (3at) was synthesized by following Procedure C. The crude material was purified by column chromatography (SiO_2, petroleum ether) to provide **3at** as a white solid (66.9 mg, 77% yield).

^{1}H NMR (400 MHz, $CDCl_3$) δ 7.98 – 7.84 (m, 1H), 7.77 (dd, J = 7.8, 1.2 Hz, 1H), 7.72 – 7.61 (m, 3H), 7.55 – 7.51 (m, 1H), 7.45 – 7.37 (m, 4H), 7.34 (d, J = 1.6 Hz, 1H), 7.25 (d, J = 7.3 Hz, 1H), 7.22 (d, J = 1.9 Hz, 1H), 7.17 – 7.09 (m, 5H), 7.07 (s, 1H), 6.65 (d, J = 3.3 Hz, 1H), 2.44 (d, J = 8.3 Hz, 6H).

^{13}C NMR (100 MHz, $CDCl_3$) δ 146.3, 146.3, 142.4, 142.4, 142.3, 142.2, 140.6, 140.6, 140.6, 140.5, 139.1, 139.0, 138.3, 138.3, 137.9, 137.7, 137.6, 137.5, 137.5, 137.1,

136.5, 130.8, 130.3, 129.7, 129.5, 129.2, 129.2, 129.2, 129.1, 129.1, 128.9, 128.8, 128.6, 128.1, 128.0, 128.0, 127.9, 127.6, 127.6, 127.4, 126.9, 126.3, 126.1, 126.1, 125.1, 125.1, 21.4, 21.4.

HRMS: (ESI) m/z: $[M+K]^+$ Calcd for $C_{34}H_{26}K$ 473.1666; Found 473.1667.

Optical $[\alpha]_{25}^{D}$ = -243.4 ° (c = 0.25, CH_2Cl_2, 91% ee (major), 89% ee (minor), d.r.= 1.5:1)

HPLC (AD-H, ^{i}PrOH/n-hexane = 5/95, flow rate = 0.5 mL/min, l = 254 nm) t_R = 14.3 min (major), 13.2 min (minor).

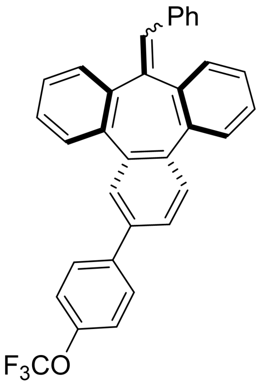

9-Benzylidene-2-(4-(trifluoromethoxy)phenyl)-9H-tribenzo[a,c,e][7]annulene (3au) was synthesized by following Procedure C. The crude material was purified by column chromatography (SiO_2, petroleum ether) to provide **3au** as a white solid (62.7 mg, 64% yield).

^{1}H NMR (400 MHz, $CDCl_3$) δ 7.88 – 7.76 (m, 1H), 7.72 – 7.62 (m, 4H), 7.59 (ddd, *J* = 10.1, 6.0, 2.8 Hz, 2H), 7.49 – 7.45 (m, 1H), 7.40 – 7.31 (m, 3H), 7.29 (d, *J* = 8.3 Hz, 2H), 7.19 (s, 1H), 7.16 (d, *J* = 1.6 Hz, 1H), 7.10 – 7.05 (m, 3H), 7.04 (d, *J* = 2.4 Hz, 2H), 6.58 (d, *J* = 3.4 Hz, 1H).

^{13}C NMR (150 MHz, $CDCl_3$) δ 148.8, 148.7, 146.4, 146.3, 142.3, 142.3, 142.2, 139.4, 139.3, 138.9, 138.9, 138.3, 138.2, 137.6, 137.3, 137.2, 136.9, 136.4, 131.1, 130.6, 129.6, 129.5, 129.3, 129.2, 129.2, 128.9, 128.8, 128.6, 128.6, 128.5, 128.3, 128.2, 128.2, 128.2, 128.0, 127.7, 127.6, 127.5, 127.0, 126.2, 126.2, 126.1, 121.3, 121.3, 120.5 (q, *J* = 257.3 Hz) .

^{19}F NMR (565 MHz, $CDCl_3$) δ -57.57.

HRMS: (ESI) m/z: $[M+K]^+$ Calcd for $C_{33}H_{21}F_3OK$ 529.1176; Found 529.1180.

Optical $[\alpha]_{25}^{D}$ = -213.9 ° (c = 0.25, CH_2Cl_2, 84% ee (major), 84% ee (minor), d.r.= 1.4:1)

HPLC (AD-H, ^{i}PrOH/n-hexane = 5/95, flow rate = 0.5 mL/min, l = 254 nm) t_R = 13.2 min (major), 15.2 min (minor).

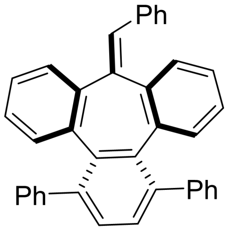

9-Benzylidene-1,4-diphenyl-9H-tribenzo[a,c,e][7]annulene (3av) was synthesized by following Procedure C. The crude material was purified by column chromatography (SiO_2, petroleum ether: CH_2Cl_2 = 20:1) to provide **3av** as a white solid (54.1 mg, 56% yield).

^{1}H NMR (400 MHz, $CDCl_3$) δ 7.46 – 7.34 (m, 4H), 7.27 (d, *J* = 7.5 Hz, 1H), 7.18 (d, *J* = 7.4 Hz, 5H), 7.12 (d, *J* = 6.8 Hz, 7H), 6.97 (dt, *J* = 37.2, 7.7 Hz, 4H), 6.85 (d, *J* = 8.0 Hz, 1H), 6.72 (d, *J* = 3.6 Hz, 2H), 6.65 (dt, *J* = 14.5, 7.6 Hz, 2H).

^{13}C NMR (100 MHz, $CDCl_3$) δ 148.9, 144.8, 143.1, 143.0, 142.9, 141.5, 141.4, 137.8, 137.8, 136.5, 134.9, 134.6, 133.8, 133.3, 130.3, 130.2, 130.1, 129.8, 129.2, 128.1, 127.8, 127.7, 127.3, 127.3, 127.3, 126.4, 126.3, 126.3, 126.1, 125.6, 125.5, 125.0.

HRMS: (ESI) m/z: $[M+K]^+$ Calcd for $C_{38}H_{26}K$ 521.1666; Found 521.1675.

Optical $[\alpha]_{25}^{D}$ = -47.0 ° (c = 0.25, CH_2Cl_2, 85% ee)

HPLC (OD-H, ^{i}PrOH/n-hexane = 5/95, flow rate = 0.5 mL/min, l = 254 nm) t_R = 10.0 min (major), 8.9 min (minor).

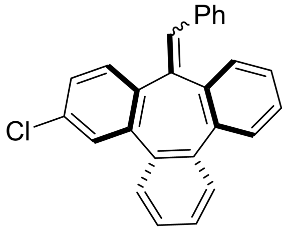

9-Benzylidene-6-chloro-9H-tribenzo[a,c,e][7]annulene (3aw) was synthesized by following Procedure C. The crude material was purified by column chromatography (SiO_2, petroleum ether) to provide **3aw** as a white solid (49.6 mg, 68% yield).

^{1}H NMR (400 MHz, $CDCl_3$) δ 7.65 (d, *J* = 7.5 Hz, 1H), 7.58 – 7.54 (m, 1H), 7.54 – 7.47 (m, 2H), 7.44 – 7.26 (m, 5H), 7.14 (d, *J* = 4.0 Hz, 1H), 7.09 – 7.00 (m, 4H), 6.97 (q, *J* = 3.1 Hz, 2H), 6.52 (d, *J* = 17.1 Hz, 1H).

^{13}**C NMR** (100 MHz, $CDCl_3$) δ 145.9, 144.7, 142.0, 141.2, 140.8, 139.6, 139.1, 139.0, 138.9, 137.6, 137.5, 137.2, 136.1, 136.1, 133.2, 133.2, 130.5, 130.2, 130.0, 129.8, 129.7, 129.7, 129.5, 129.2, 129.2, 129.0, 128.8, 128.7, 128.2, 128.1, 128.0, 128.0, 128.0, 127.8, 127.8, 127.7, 127.7, 127.4, 127.3, 127.1, 127.1, 126.1.

HRMS: (ESI) m/z: $[M+K]^+$ Calcd for $C_{26}H_{17}ClK$ 403.0650; Found 403.0649.

Optical $[\alpha]_{25}^{D}$ = -203.8 ° (c = 0.25, CH_2Cl_2, 88% ee (major), 82% ee (minor), d.r.= 1.4:1)

HPLC (AD-H, ^{i}PrOH/n-hexane = 5/95, flow rate = 0.5 mL/min, l = 254 nm) t_R = 12.2 min (major), 13.8 min (minor).

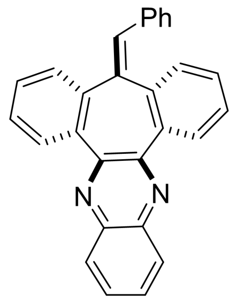

10-Benzylidene-10H-dibenzo[3,4:6,7]cyclohepta[1,2-b]quinoxaline (3ax) was synthesized by following Procedure C. The crude material was purified by column chromatography (SiO_2, petroleum ether: CH_2Cl_2 = 3:1) to provide **3ax** as a yellow solid (44.3 mg, 58% yield).

^{1}**H NMR** (400 MHz, $CDCl_3$) δ 8.31 – 8.23 (m, 3H), 8.22 – 8.18 (m, 1H), 7.86 – 7.79 (m, 2H), 7.59 (d, *J* = 2.2 Hz, 1H), 7.58 – 7.52 (m, 2H), 7.51 (s, 1H), 7.38 (t, *J* = 7.5 Hz, 1H), 7.28 (s, 1H), 7.10 (d, *J* = 8.1 Hz, 5H), 6.72 (s, 1H).

^{13}**C NMR** (100 MHz, $CDCl_3$) δ 152.1, 152.0, 146.5, 142.4, 141.5, 141.4, 140.6, 135.9, 135.8, 135.5, 131.6, 131.2, 130.5, 130.2, 130.1, 130.0, 130.0, 129.3, 129.3, 129.2, 128.1, 128.0, 127.9, 127.9, 127.3, 126.4.

HRMS: (ESI) m/z: $[M+H]^+$ Calcd for $C_{28}H_{19}N_2$ 383.1543; Found 383.1551.

Optical $[\alpha]_{25}^{D}$ = +239.0 ° (c = 0.25, CH_2Cl_2, 80% ee)

HPLC (IA-H, ^{i}PrOH/n-hexane = 5/95, flow rate = 0.5 mL/min, l = 254 nm) t_R = 11.9 min (major), 11.2 min (minor).

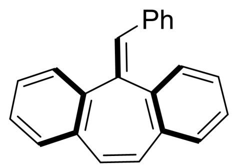

5-Benzylidene-5H-dibenzo[a,d][7]annulene (3ay) was synthesized by following Procedure C. The crude material was purified by column chromatography (SiO_2, petroleum ether: CH_2Cl_2 = 3:1) to provide **3ay** as a white solid (30.8 mg, 55% yield).

^{1}H NMR (400 MHz, $CDCl_3$) δ 7.41 (d, J = 7.6 Hz, 1H), 7.31 – 7.25 (m, 2H), 7.18 (d, J = 4.0 Hz, 2H), 7.14 (dd, J = 7.6, 1.6 Hz, 1H), 7.05 (s, 1H), 7.03 – 7.01 (m, 1H), 7.00 – 6.96 (m, 3H), 6.86 (d, J = 12.0 Hz, 1H), 6.83 – 6.77 (m, 3H), 6.39 (s, 1H).

HRMS: (ESI) m/z: $[M+H]^+$ Calcd for $C_{22}H_{17}$ 281.1325; Found 281.1327.

Optical $[\alpha]_{25}^{D}$ = 0 ° (c = 0.25, CH_2Cl_2, 0% ee)

HPLC (OJ-H, ^{i}PrOH/n-hexane = 30/70, flow rate = 0.5 mL/min, l = 254 nm) t_R = 29.1 min (major), 65.0 min (minor).

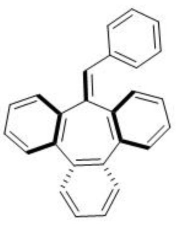

3a

^{1}H NMR, 400 MHz, CDCl$_3$

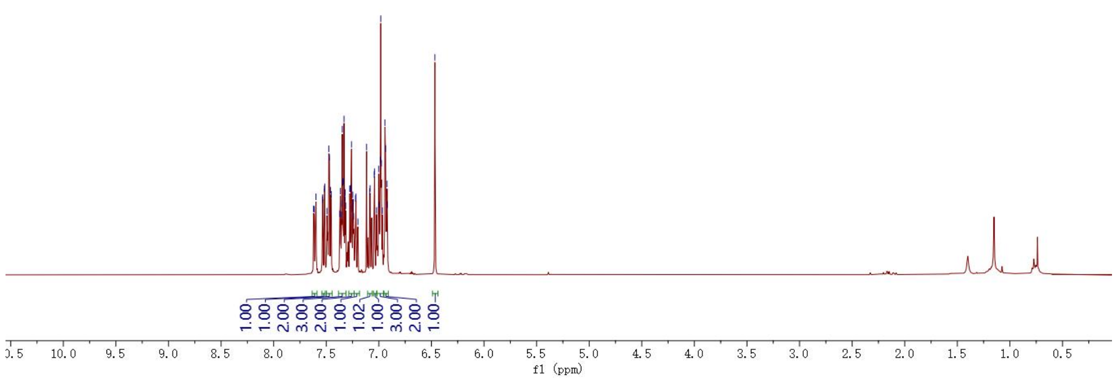

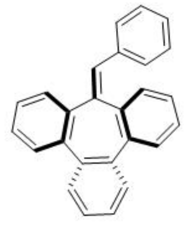

3a

^{13}C NMR, 100 MHz, CDCl$_3$

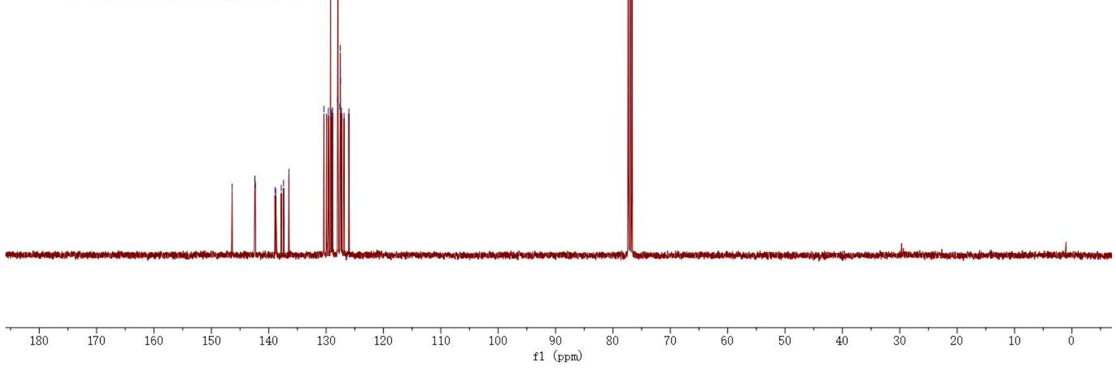

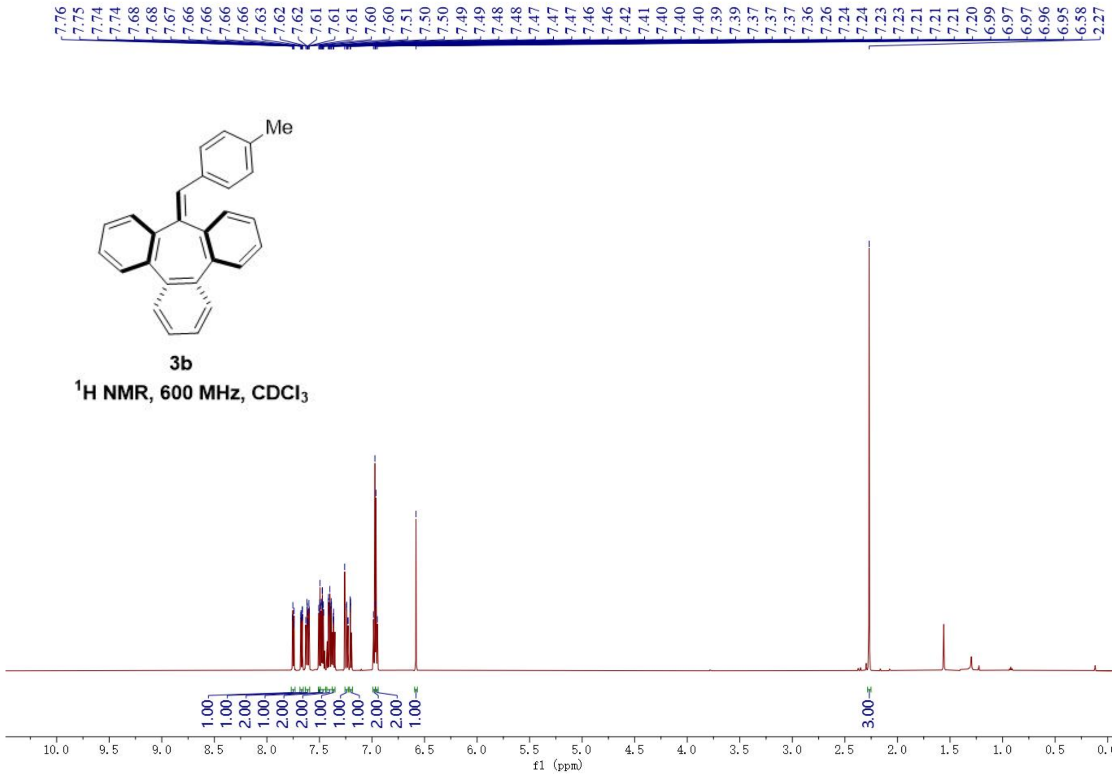
7.76 7.75 7.74 7.68 7.68 7.67 7.66 7.66 7.66 7.63 7.62 7.61 7.61 7.61 7.60 7.60 7.51 7.50 7.49 7.49 7.48 7.48 7.47 7.47 7.47 7.46 7.42 7.41 7.40 7.40 7.40 7.39 7.37 7.37 7.37 7.36 7.26 7.24 7.24 7.23 7.23 7.21 7.21 7.21 7.20 6.99 6.97 6.97 6.96 6.95 6.58 2.27
Me
3b
^{1}H NMR, 600 MHz, CDCl$_3$
1.00 1.00 2.00 1.00 2.00 2.00 1.00 1.00 1.00 2.00 2.00 1.00
3.00
f1 (ppm)

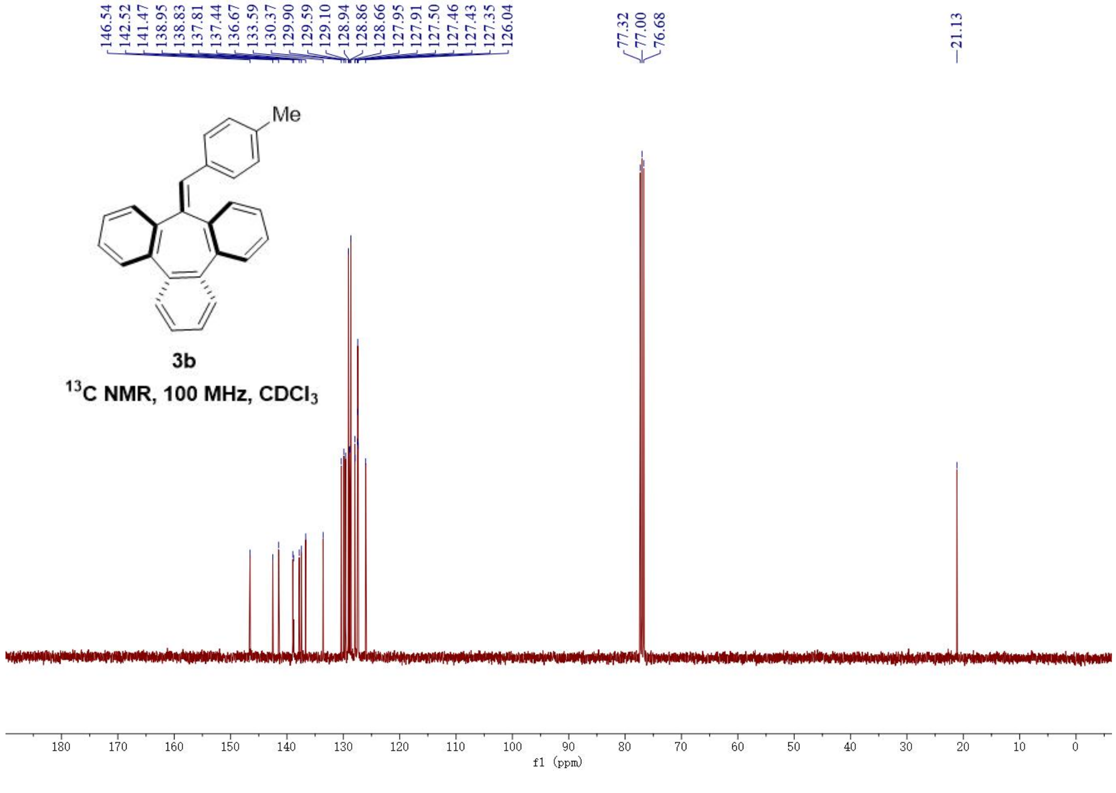
146.54 142.52 141.47 138.95 138.83 137.81 137.44 136.67 133.59 130.37 129.90 129.59 129.10 128.94 128.86 128.66 127.95 127.91 127.50 127.46 127.43 127.35 126.04
77.32 77.00 76.68
21.13
Me
3b
^{13}C NMR, 100 MHz, CDCl$_3$
f1 (ppm)

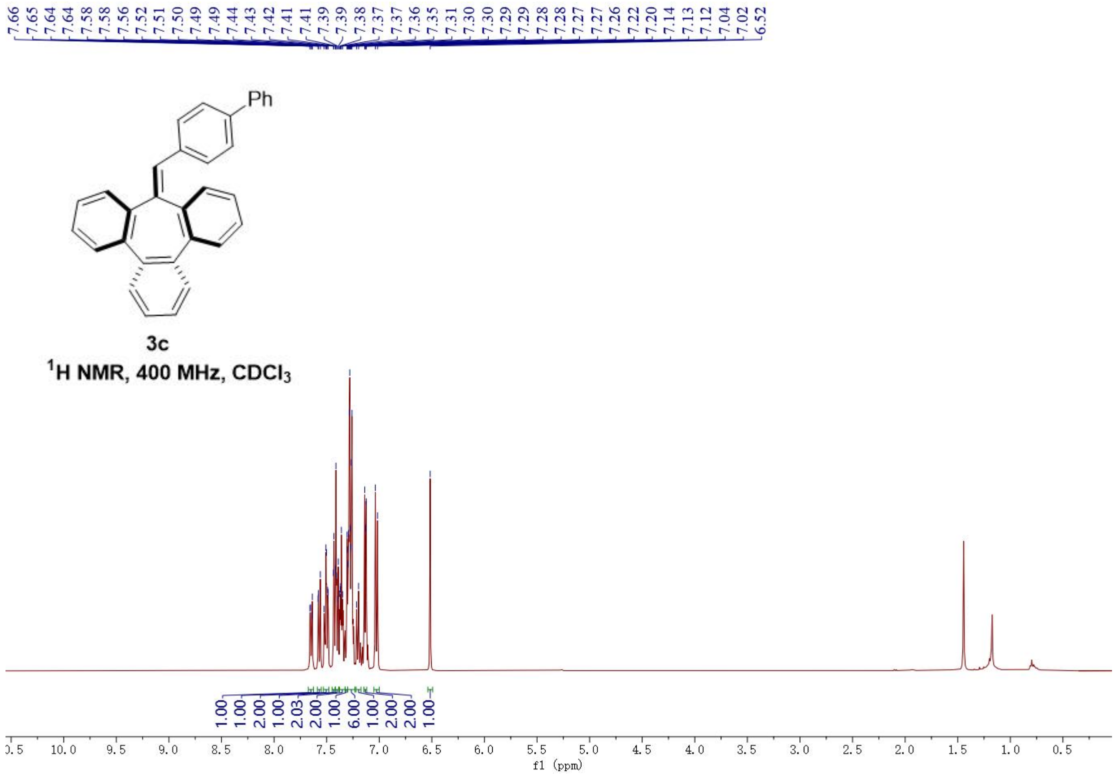
7.66 7.65 7.64 7.64 7.58 7.58 7.56 7.52 7.51 7.50 7.49 7.49 7.44 7.43 7.42 7.41 7.41 7.39 7.39 7.38 7.37 7.37 7.36 7.35 7.31 7.30 7.30 7.29 7.29 7.28 7.28 7.27 7.27 7.26 7.22 7.20 7.14 7.13 7.12 7.04 7.02 6.52
Ph
3c
1H NMR, 400 MHz, CDCl3
1.00 1.00 2.00 1.00 2.03 2.00 1.00 6.00 1.00 2.00 2.00 1.00
f1 (ppm)

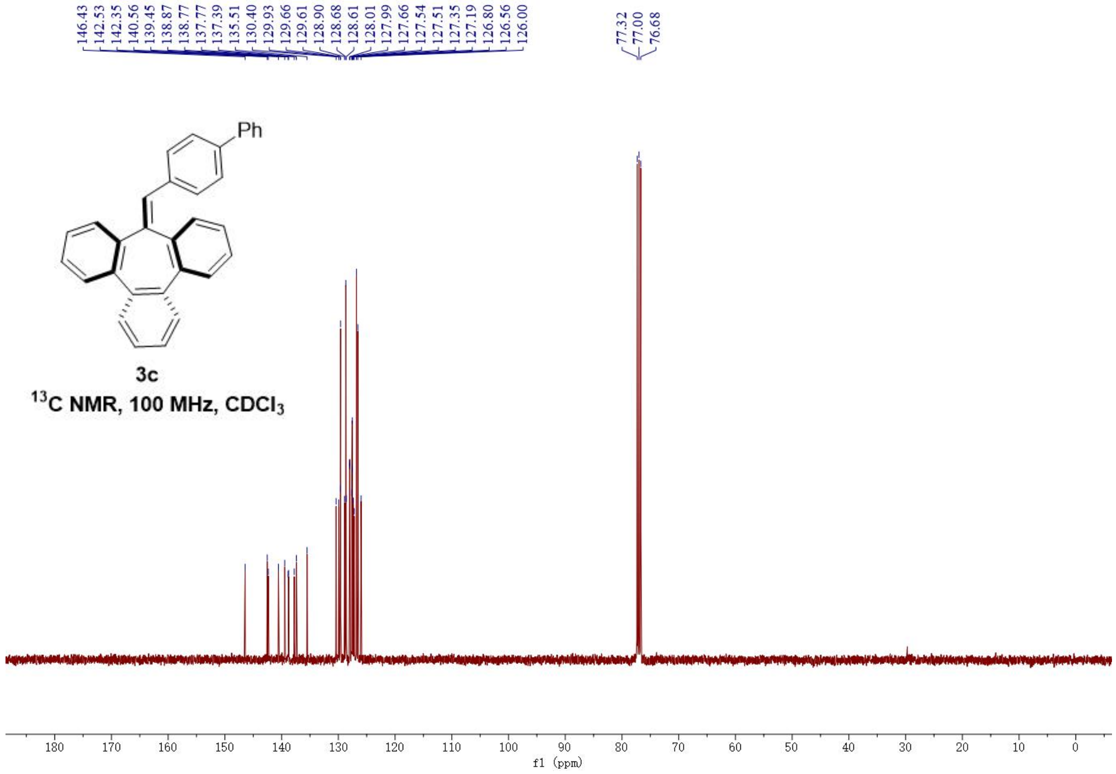
146.43 142.53 142.35 140.56 139.45 138.87 138.77 137.77 137.39 135.51 130.40 129.93 129.66 129.61 128.90 128.68 128.61 128.01 127.99 127.66 127.54 127.51 127.35 127.19 126.80 126.56 126.00
77.32 77.00 76.68
Ph
3c
13C NMR, 100 MHz, CDCl3
f1 (ppm)

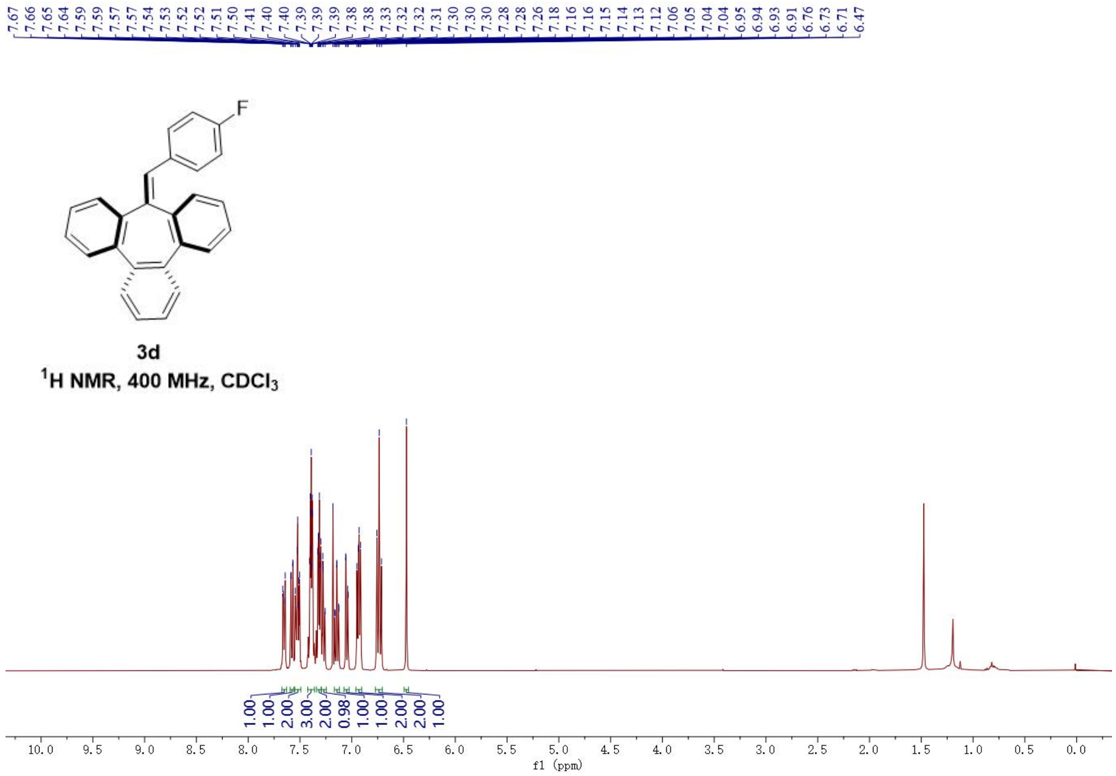
7.67 7.66 7.65 7.64 7.59 7.59 7.57 7.57 7.54 7.53 7.52 7.52 7.51 7.50 7.41 7.40 7.39 7.39 7.39 7.39 7.38 7.38 7.33 7.32 7.32 7.31 7.30 7.30 7.30 7.28 7.28 7.26 7.18 7.16 7.16 7.15 7.14 7.13 7.12 7.06 7.05 7.04 7.04 6.95 6.94 6.93 6.91 6.76 6.73 6.71 6.47
F
3d
^{1}H NMR, 400 MHz, CDCl$_3$
1.00 1.00 2.00 3.00 2.00 0.98 1.00 1.00 2.00 2.00 1.00
10.0 9.5 9.0 8.5 8.0 7.5 7.0 6.5 6.0 5.5 5.0 4.5 4.0 3.5 3.0 2.5 2.0 1.5 1.0 0.5 0.0
f1 (ppm)

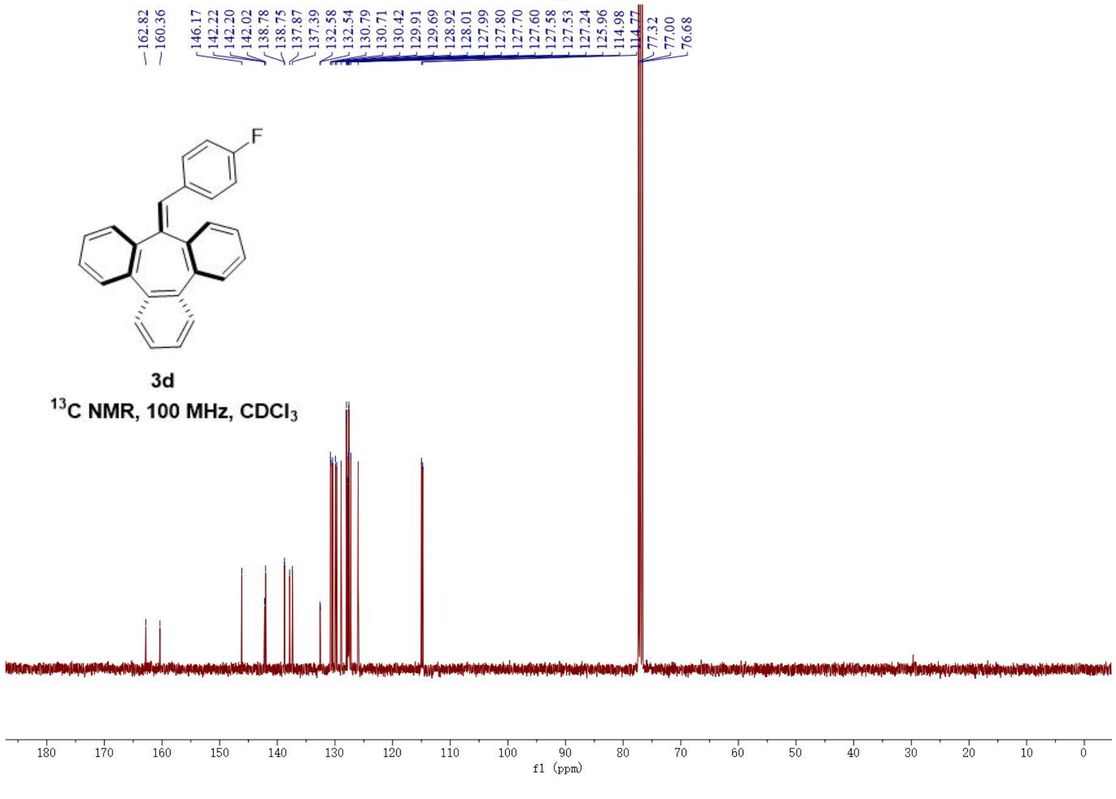
162.82 160.36 146.17 142.22 142.20 142.02 138.78 138.75 137.87 137.39 132.58 132.54 130.79 130.71 130.42 129.91 129.69 128.92 128.01 127.99 127.80 127.70 127.60 127.58 127.53 127.24 125.96 114.98 114.77 77.32 77.00 76.68
F
3d
^{13}C NMR, 100 MHz, CDCl$_3$
180 170 160 150 140 130 120 110 100 90 80 70 60 50 40 30 20 10 0
f1 (ppm)

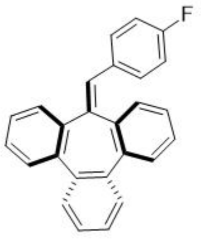

3d

^{19}F NMR, 376 MHz, $CDCl_3$

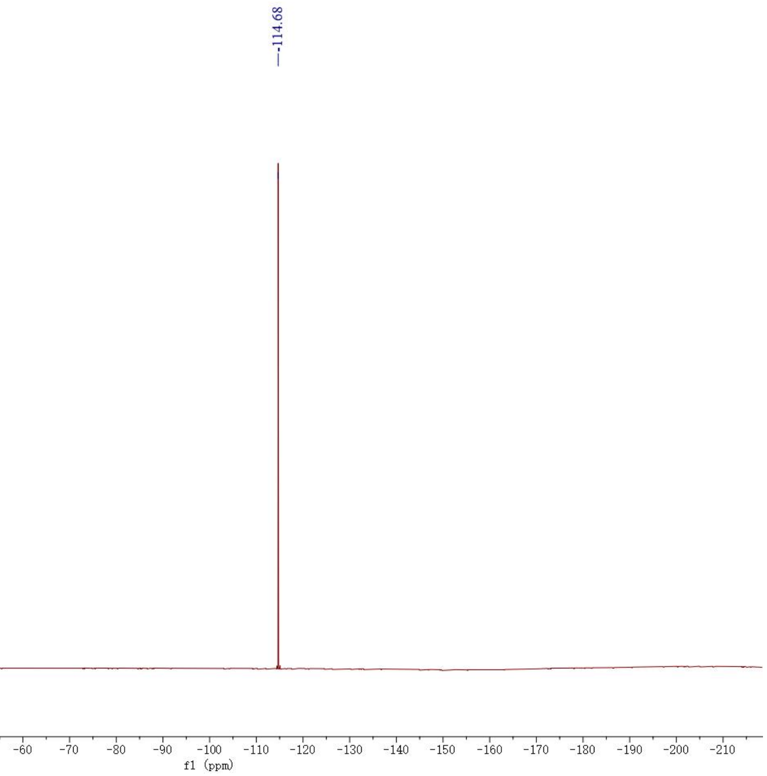

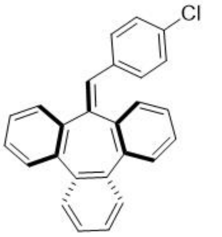

3e

^{1}H NMR, 400 MHz, $CDCl_3$

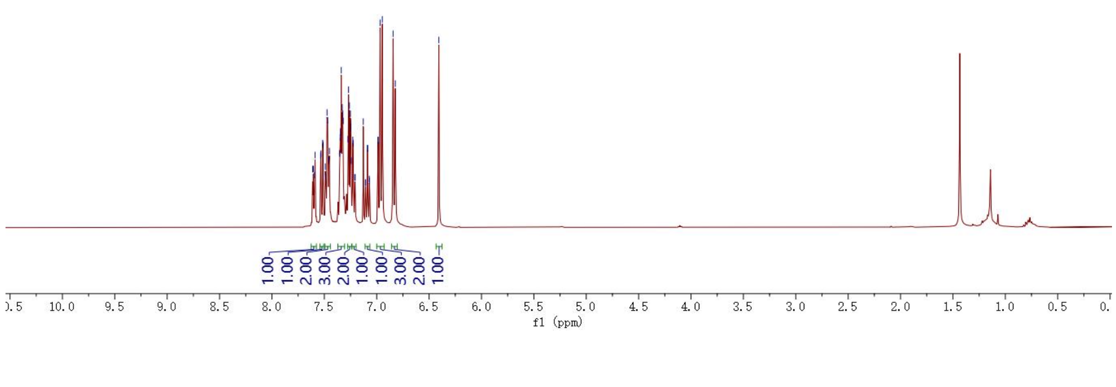

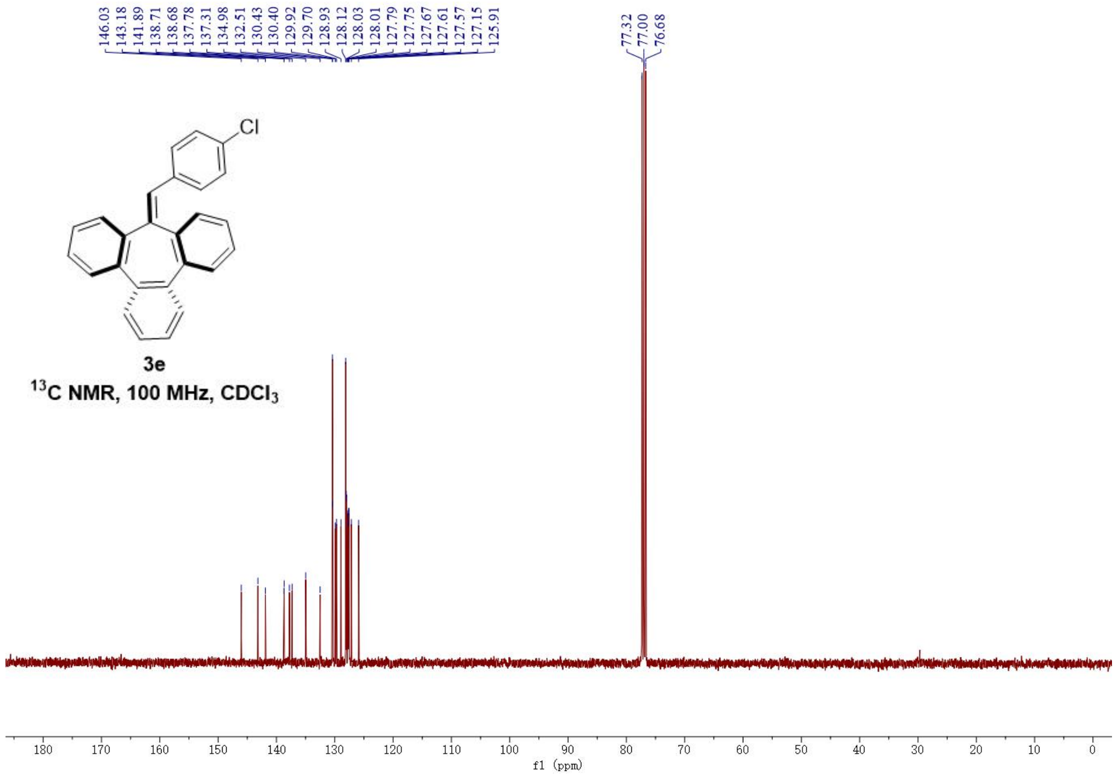
146.03
143.18
141.89
138.71
138.68
137.78
137.31
134.98
132.51
130.43
130.40
129.92
129.70
128.93
128.12
128.03
128.01
127.79
127.75
127.67
127.61
127.57
127.15
125.91
77.32
77.00
76.68
Cl
3e
13C NMR, 100 MHz, CDCl3
f1 (ppm)

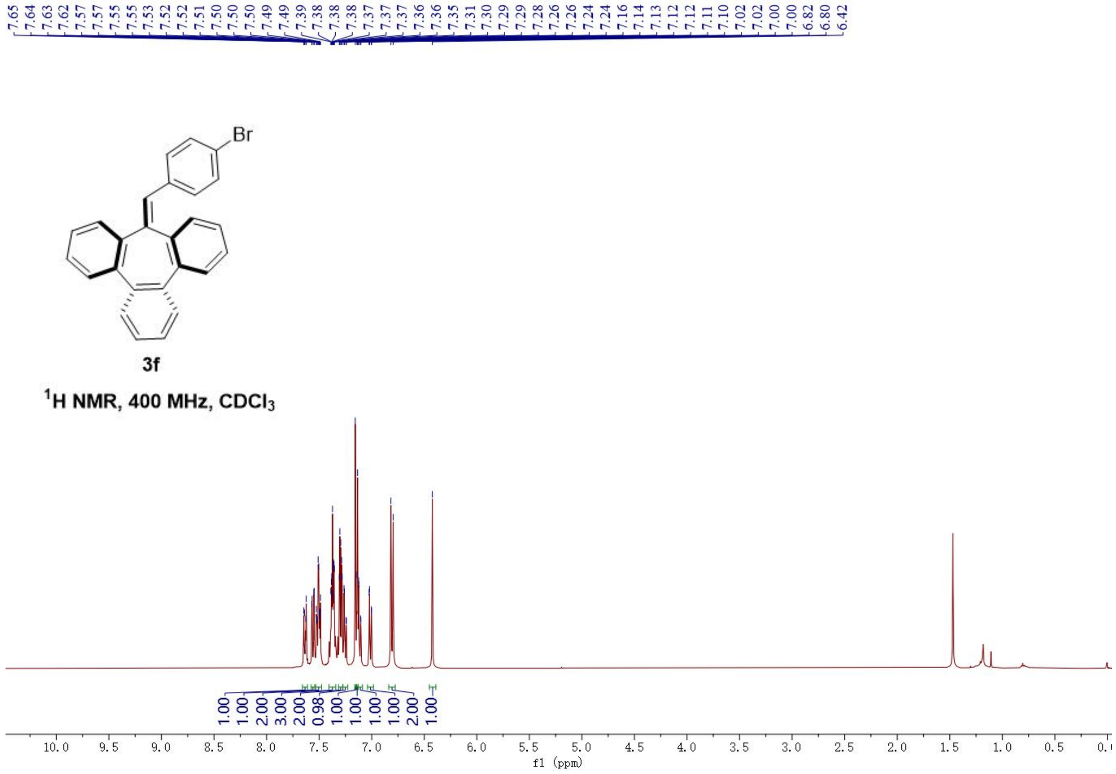
7.65
7.64
7.63
7.62
7.57
7.57
7.55
7.55
7.53
7.52
7.52
7.51
7.50
7.50
7.49
7.39
7.38
7.38
7.38
7.37
7.37
7.37
7.36
7.35
7.31
7.30
7.29
7.29
7.28
7.26
7.24
7.16
7.14
7.13
7.12
7.12
7.11
7.10
7.02
7.02
7.00
6.82
6.80
6.42
Br
3f
1H NMR, 400 MHz, CDCl3
1.00
1.00
2.00
3.00
2.00
0.98
1.00
1.00
1.00
2.00
1.00
f1 (ppm)

3f

^{13}C NMR, 100 MHz, CDCl$_3$

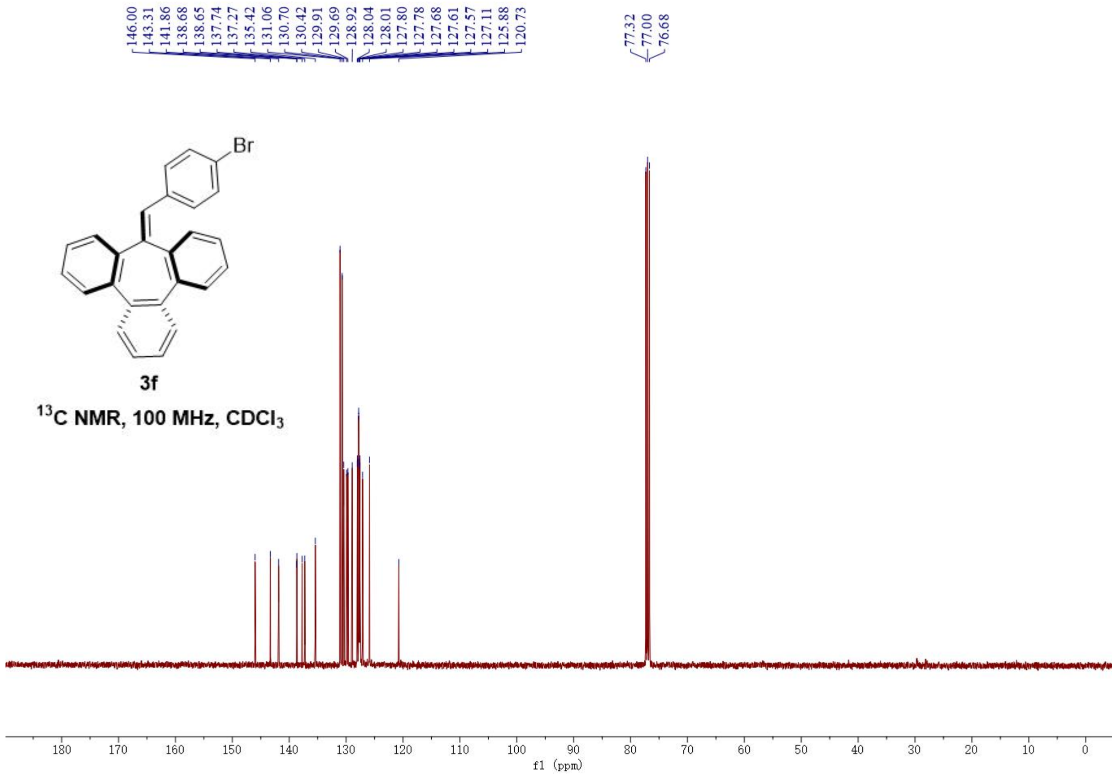

3g

^{1}H NMR, 400 MHz, CDCl$_3$

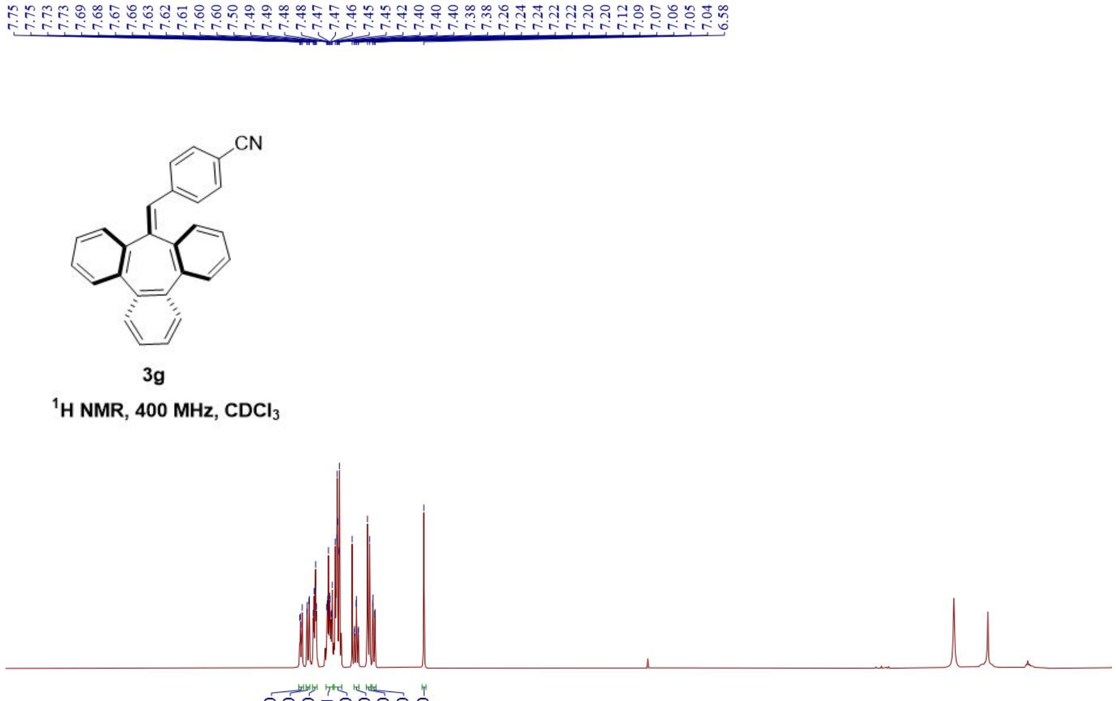

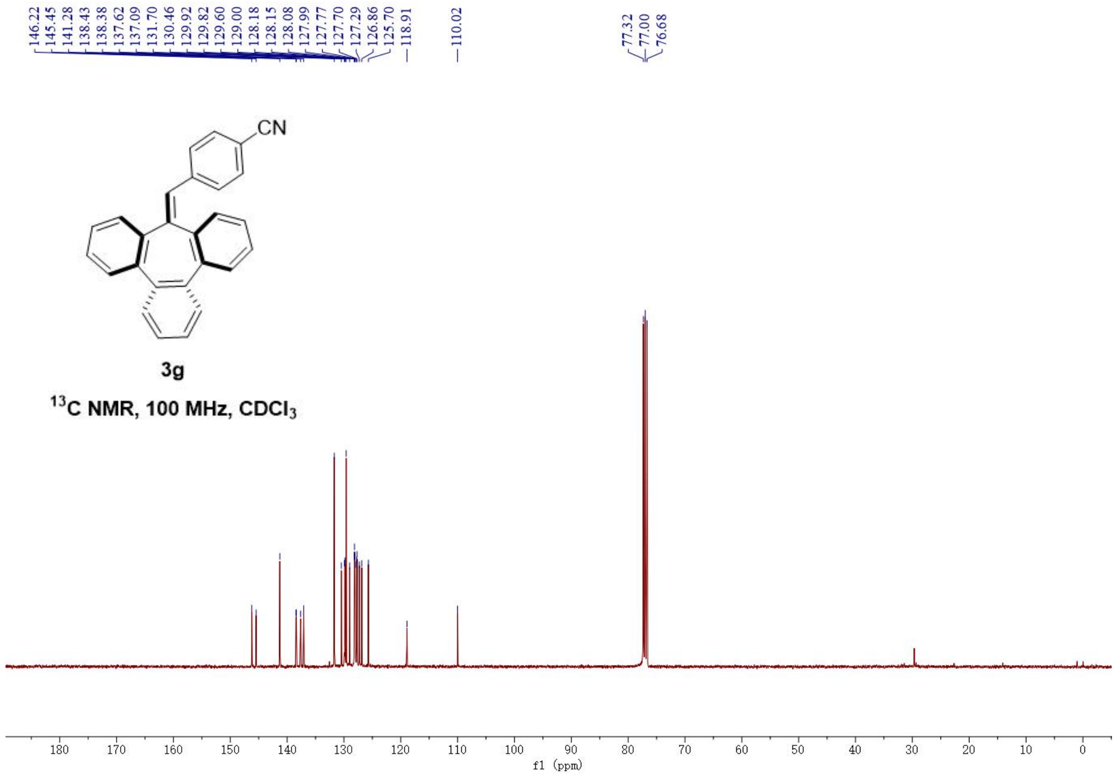
146.22
145.45
141.28
138.43
138.38
137.62
137.09
131.70
130.46
129.92
129.82
129.60
129.00
128.18
128.15
128.08
127.99
127.77
127.70
127.29
126.86
125.70
118.91
110.02
77.32
77.00
76.68
CN
3g
^{13}C NMR, 100 MHz, $CDCl_3$
180 170 160 150 140 130 120 110 100 90 80 70 60 50 40 30 20 10 0
f1 (ppm)

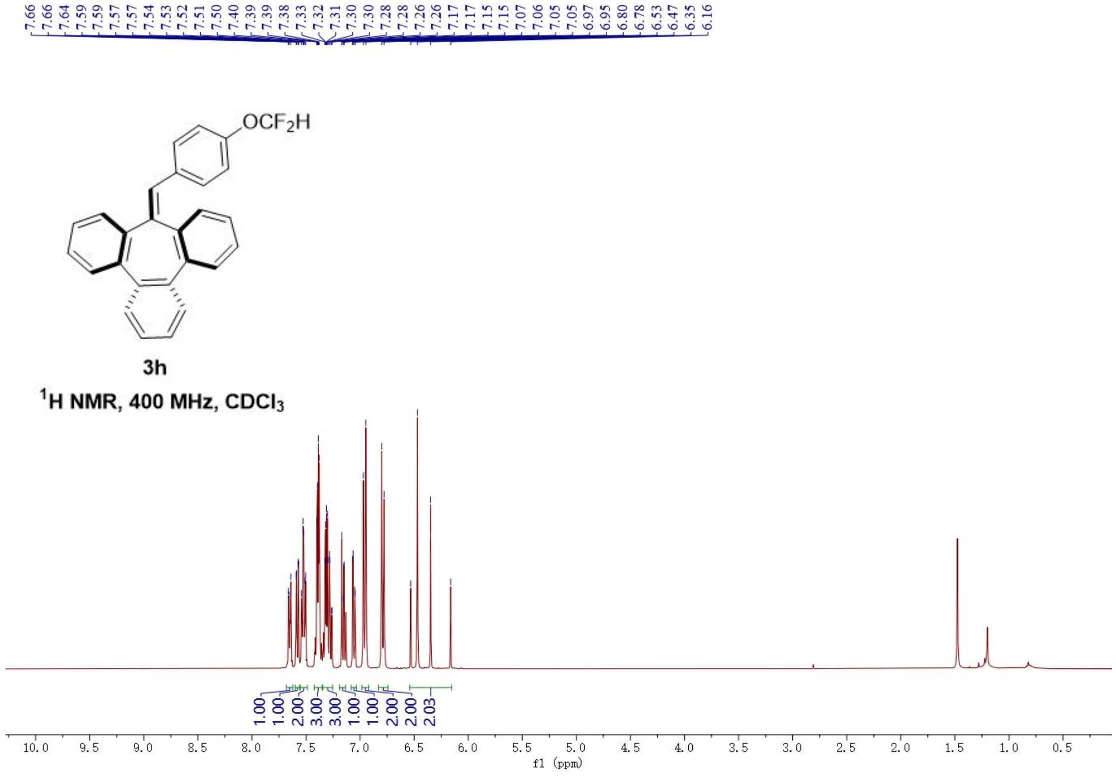
7.66
7.66
7.64
7.59
7.59
7.57
7.57
7.54
7.53
7.52
7.51
7.50
7.40
7.39
7.39
7.38
7.33
7.32
7.31
7.30
7.30
7.28
7.28
7.26
7.26
7.17
7.17
7.15
7.15
7.07
7.06
7.05
7.05
6.97
6.95
6.80
6.78
6.53
6.47
6.35
6.16
OCF_2H
3h
^{1}H NMR, 400 MHz, $CDCl_3$
1.00
1.00
2.00
3.00
3.00
1.00
1.00
2.00
2.00
2.03
10.0 9.5 9.0 8.5 8.0 7.5 7.0 6.5 6.0 5.5 5.0 4.5 4.0 3.5 3.0 2.5 2.0 1.5 1.0 0.5
f1 (ppm)

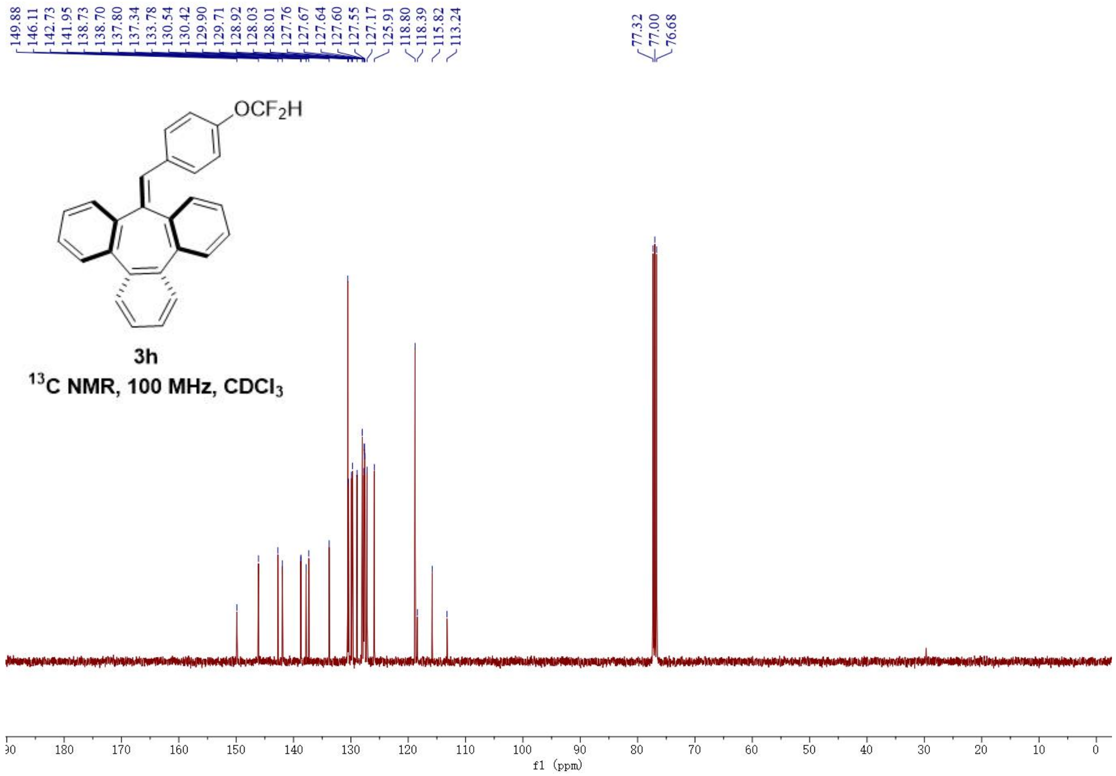
149.88
146.11
142.73
141.95
138.73
138.70
137.80
137.34
133.78
130.54
130.42
129.90
129.71
128.92
128.03
128.01
127.76
127.67
127.64
127.60
127.55
127.17
125.91
118.80
118.39
115.82
113.24
77.32
77.00
76.68
OCF2H
3h
13C NMR, 100 MHz, CDCl3
f1 (ppm)

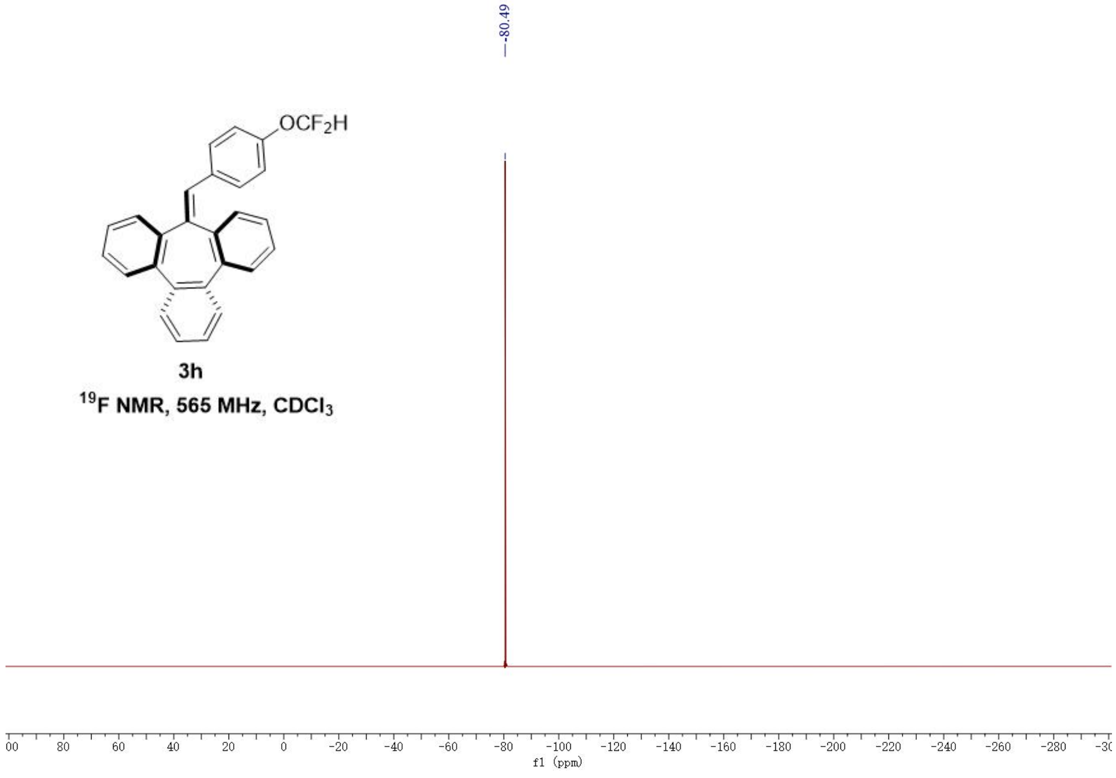
-80.49
OCF2H
3h
19F NMR, 565 MHz, CDCl3
f1 (ppm)

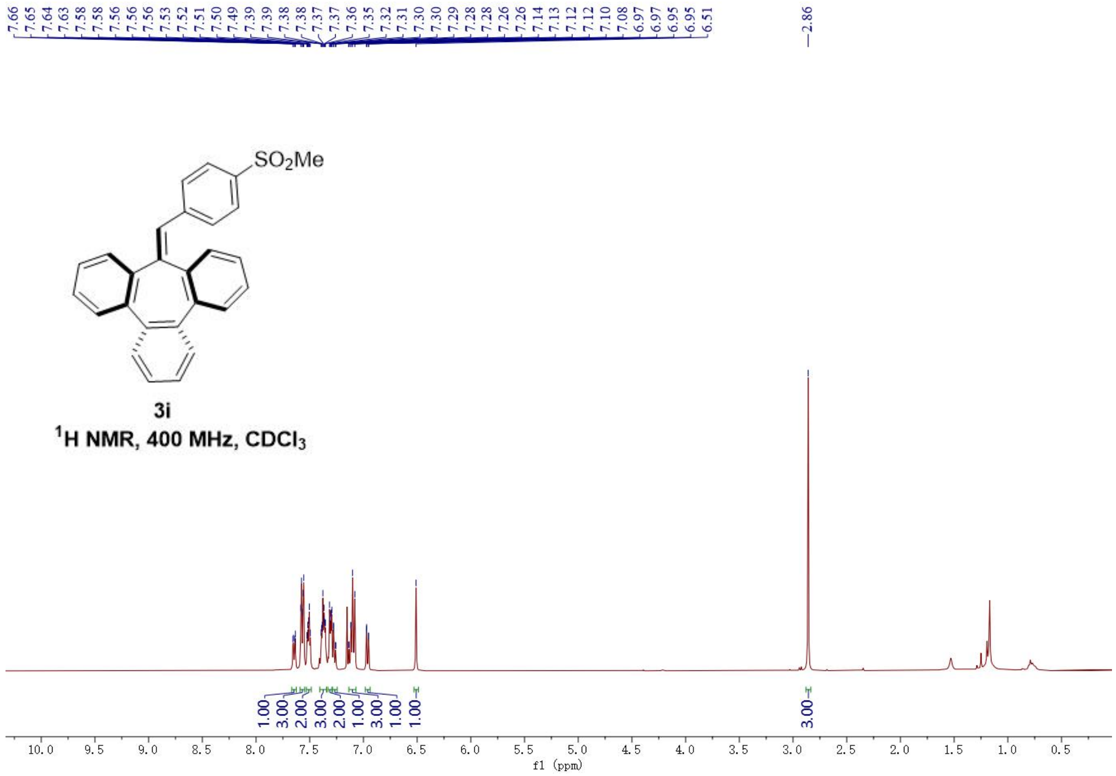
SO2Me
3i
1H NMR, 400 MHz, CDCl3
f1 (ppm)

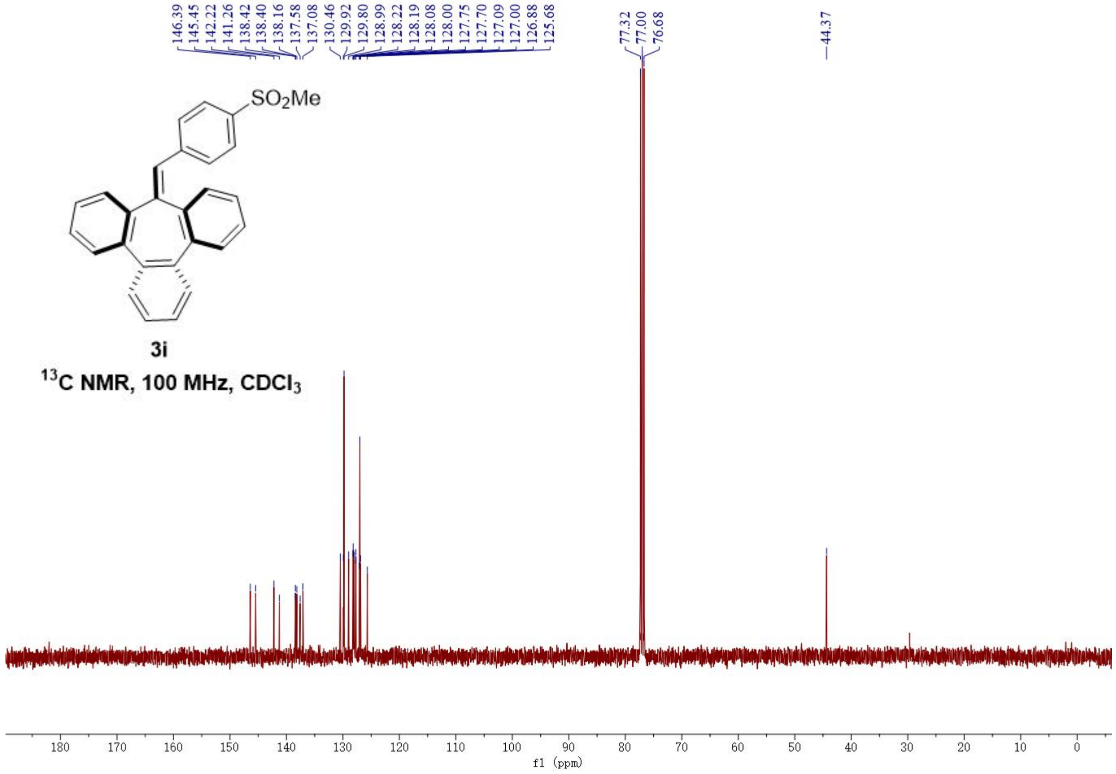
SO2Me
3i
13C NMR, 100 MHz, CDCl3
f1 (ppm)

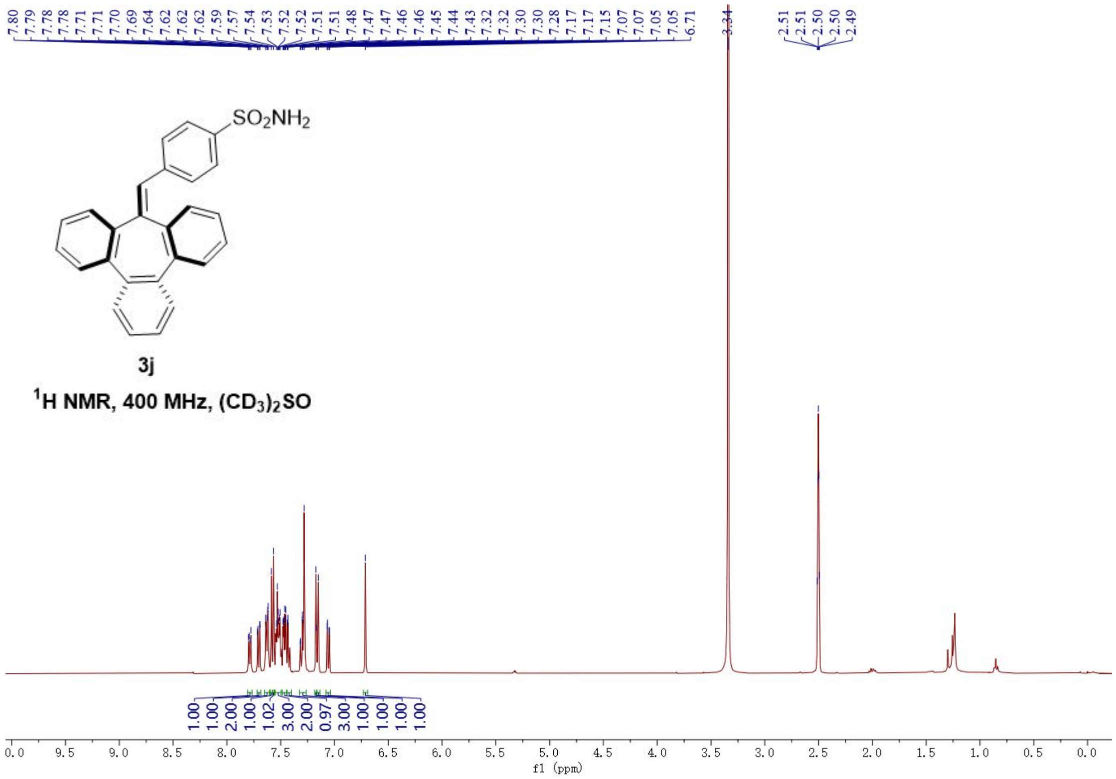
7.80 7.79 7.78 7.71 7.71 7.70 7.69 7.64 7.62 7.62 7.59 7.57 7.54 7.53 7.52 7.52 7.51 7.51 7.48 7.47 7.47 7.46 7.46 7.45 7.44 7.43 7.32 7.32 7.30 7.30 7.28 7.17 7.17 7.15 7.07 7.05 7.05 6.71
3.34
2.51 2.51 2.50 2.50 2.49
SO_2NH_2
3j
^{1}H NMR, 400 MHz, $(CD_3)_2SO$
1.00 1.00 2.00 1.00 1.02 3.00 2.00 0.97 3.00 1.00 1.00 1.00 1.00
f1 (ppm)

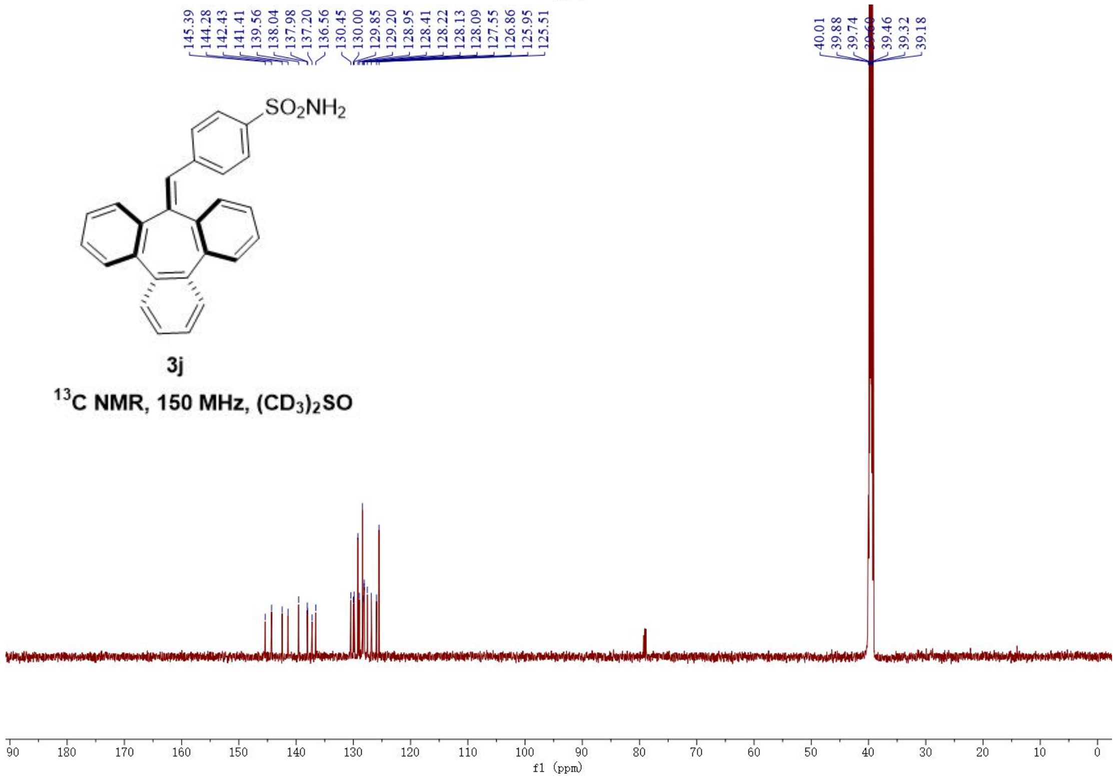
145.39 144.28 142.43 141.41 139.56 138.04 137.98 137.20 136.56 130.45 130.00 129.85 129.20 128.95 128.41 128.22 128.13 128.09 127.55 126.86 125.95 125.51
40.01 39.88 39.74 39.60 39.46 39.32 39.18
SO_2NH_2
3j
^{13}C NMR, 150 MHz, $(CD_3)_2SO$
f1 (ppm)

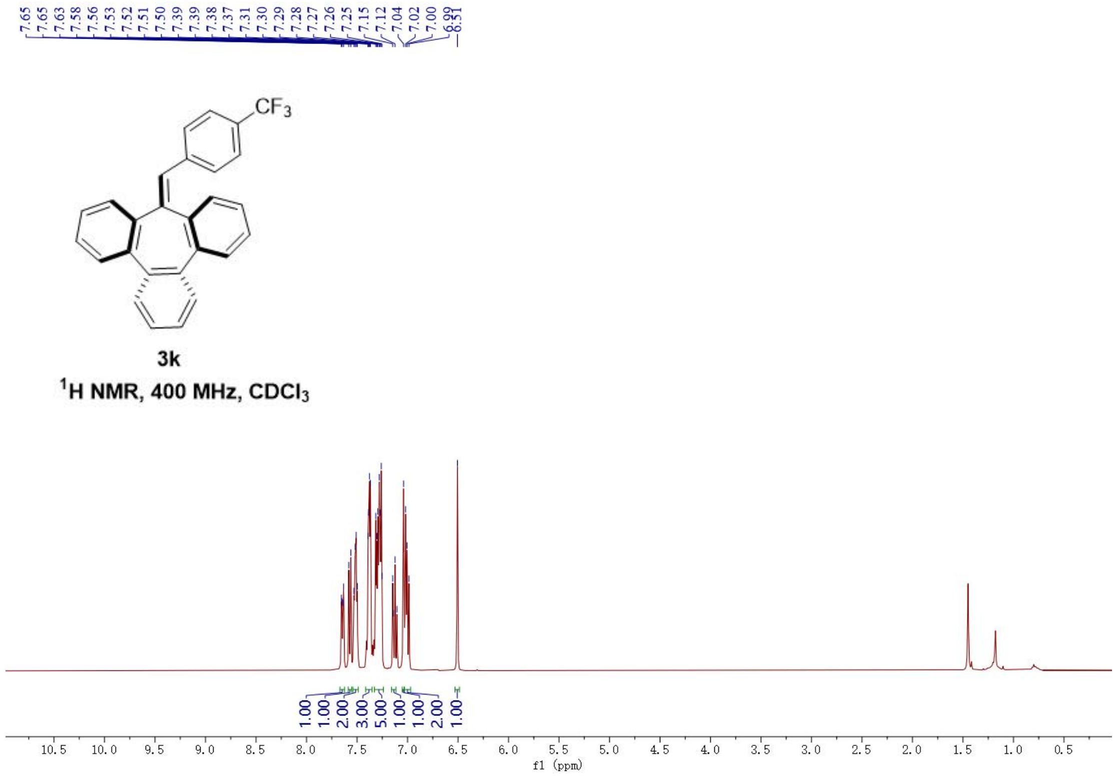
7.65 7.65 7.63 7.58 7.56 7.53 7.52 7.51 7.50 7.39 7.39 7.38 7.37 7.31 7.30 7.29 7.28 7.27 7.26 7.25 7.15 7.12 7.04 7.02 7.00 6.99 6.51
CF3
3k
1H NMR, 400 MHz, CDCl3
1.00 1.00 2.00 3.00 5.00 1.00 1.00 2.00 1.00
10.5 10.0 9.5 9.0 8.5 8.0 7.5 7.0 6.5 6.0 5.5 5.0 4.5 4.0 3.5 3.0 2.5 2.0 1.5 1.0 0.5
f1 (ppm)

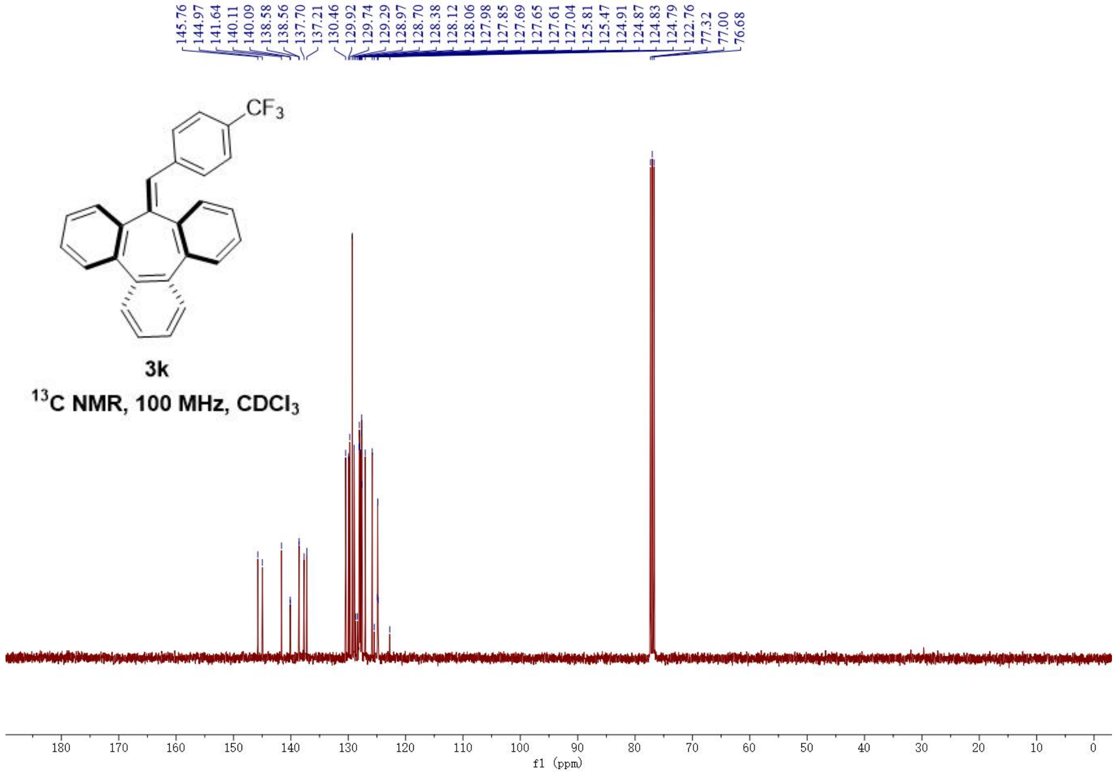
145.76 144.97 141.64 140.11 140.09 138.58 138.56 137.70 137.21 130.46 129.92 129.74 129.29 128.97 128.70 128.38 128.12 128.06 127.98 127.85 127.69 127.65 127.61 127.04 125.81 125.47 124.91 124.87 124.83 124.79 122.76 77.32 77.00 76.68
CF3
3k
13C NMR, 100 MHz, CDCl3
180 170 160 150 140 130 120 110 100 90 80 70 60 50 40 30 20 10 0
f1 (ppm)

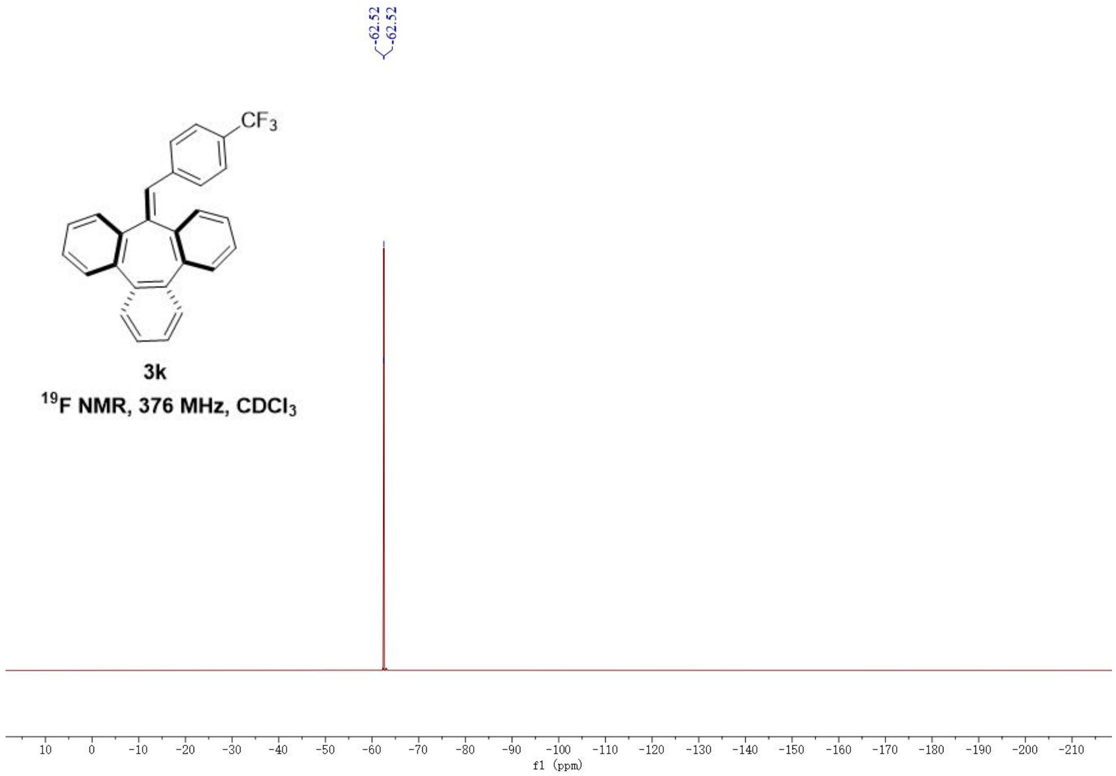
-62.52
-62.52
CF3
3k
19F NMR, 376 MHz, CDCl3
10 0 −10 −20 −30 −40 −50 −60 −70 −80 −90 −100 −110 −120 −130 −140 −150 −160 −170 −180 −190 −200 −210
f1 (ppm)

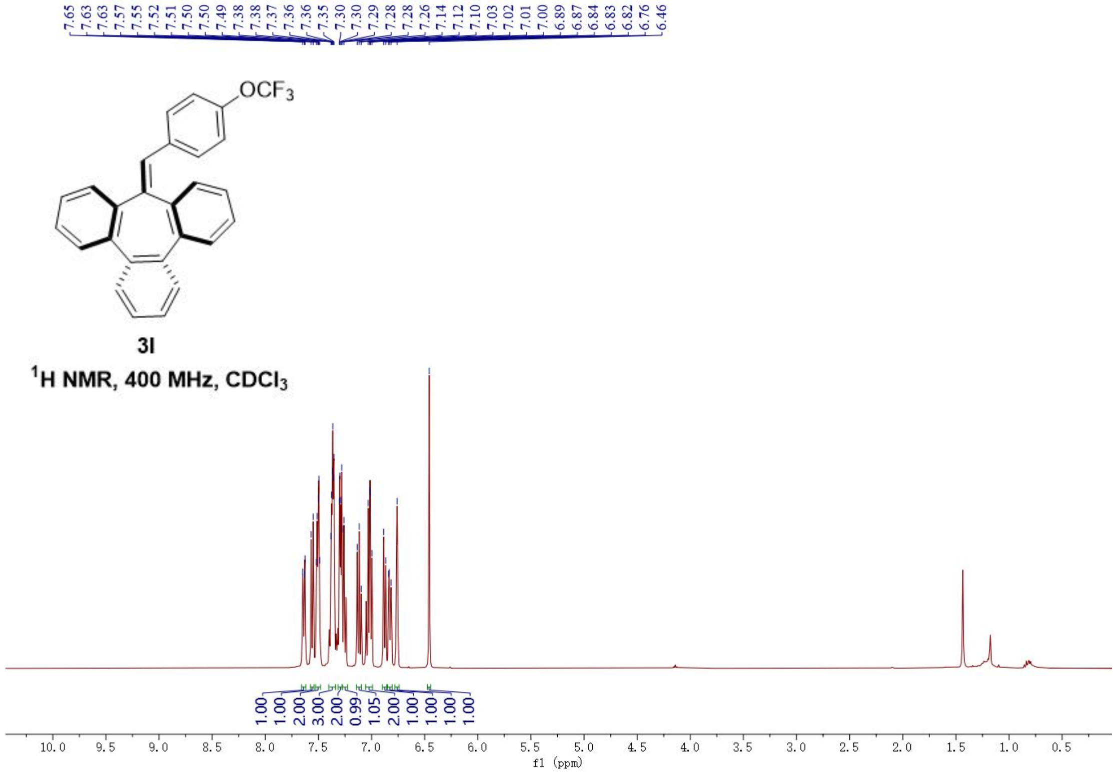
7.65 7.63 7.63 7.57 7.55 7.52 7.51 7.50 7.50 7.49 7.38 7.38 7.37 7.36 7.35 7.30 7.30 7.29 7.28 7.28 7.26 7.14 7.12 7.10 7.03 7.02 7.01 7.00 6.89 6.87 6.84 6.83 6.82 6.76 6.46
OCF3
3l
1H NMR, 400 MHz, CDCl3
1.00 1.00 2.00 3.00 2.00 0.99 1.05 2.00 1.00 1.00 1.00
10.0 9.5 9.0 8.5 8.0 7.5 7.0 6.5 6.0 5.5 5.0 4.5 4.0 3.5 3.0 2.5 2.0 1.5 1.0 0.5
f1 (ppm)

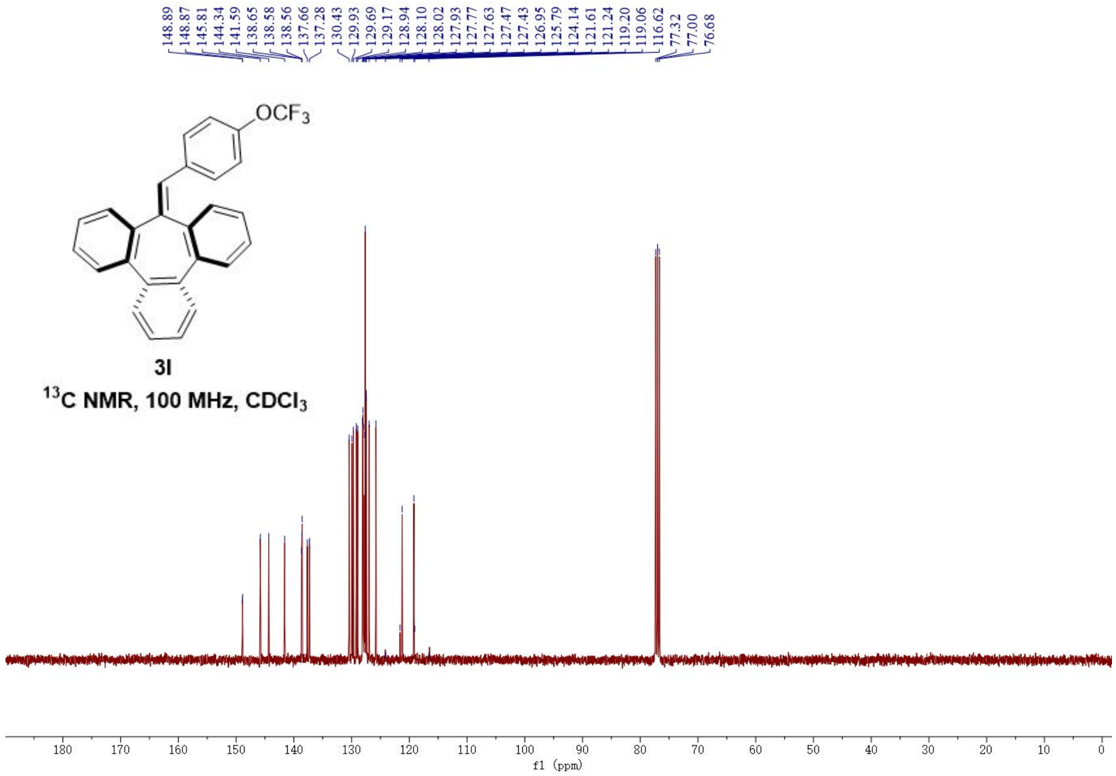
148.89
148.87
145.81
144.34
141.59
138.65
138.58
138.56
137.66
137.28
130.43
129.93
129.69
129.17
128.94
128.10
128.02
127.93
127.77
127.63
127.47
127.43
126.95
125.79
124.14
121.61
121.24
119.20
119.06
116.62
77.32
77.00
76.68
OCF$_3$
3l
^{13}C NMR, 100 MHz, CDCl$_3$
f1 (ppm)

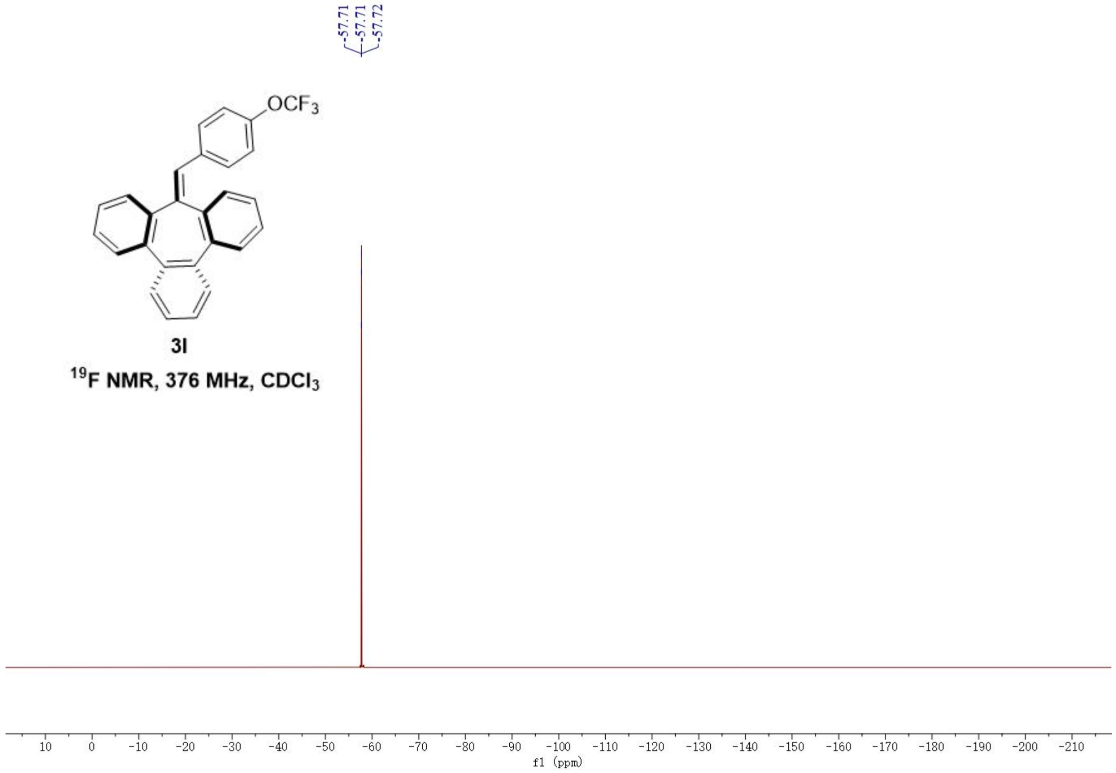
-57.71
-57.71
-57.72
OCF$_3$
3l
^{19}F NMR, 376 MHz, CDCl$_3$
f1 (ppm)

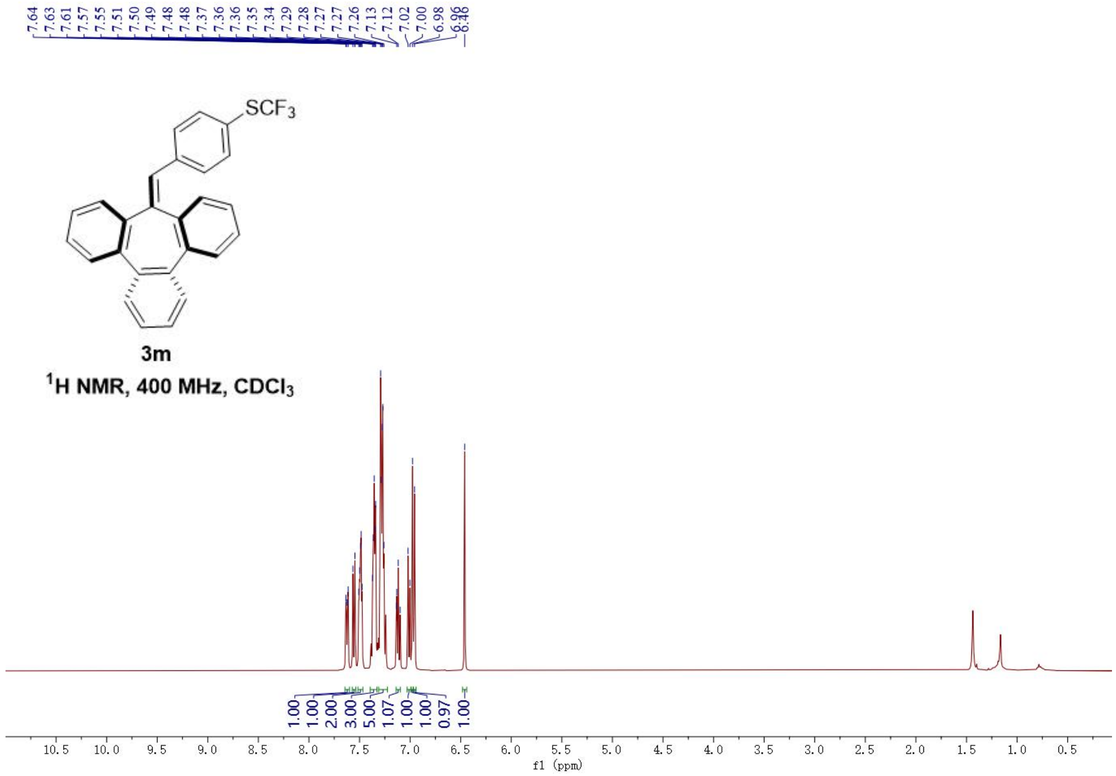

3m

^{1}H NMR, 400 MHz, $CDCl_3$

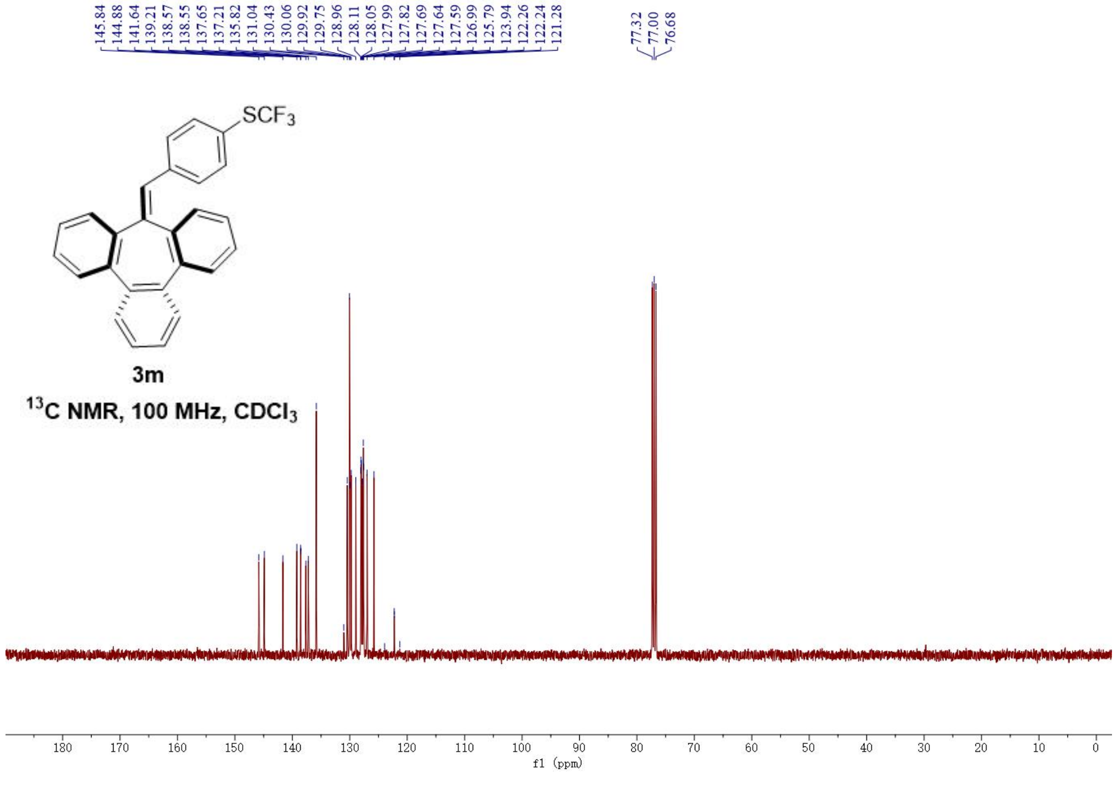

3m

^{13}C NMR, 100 MHz, $CDCl_3$

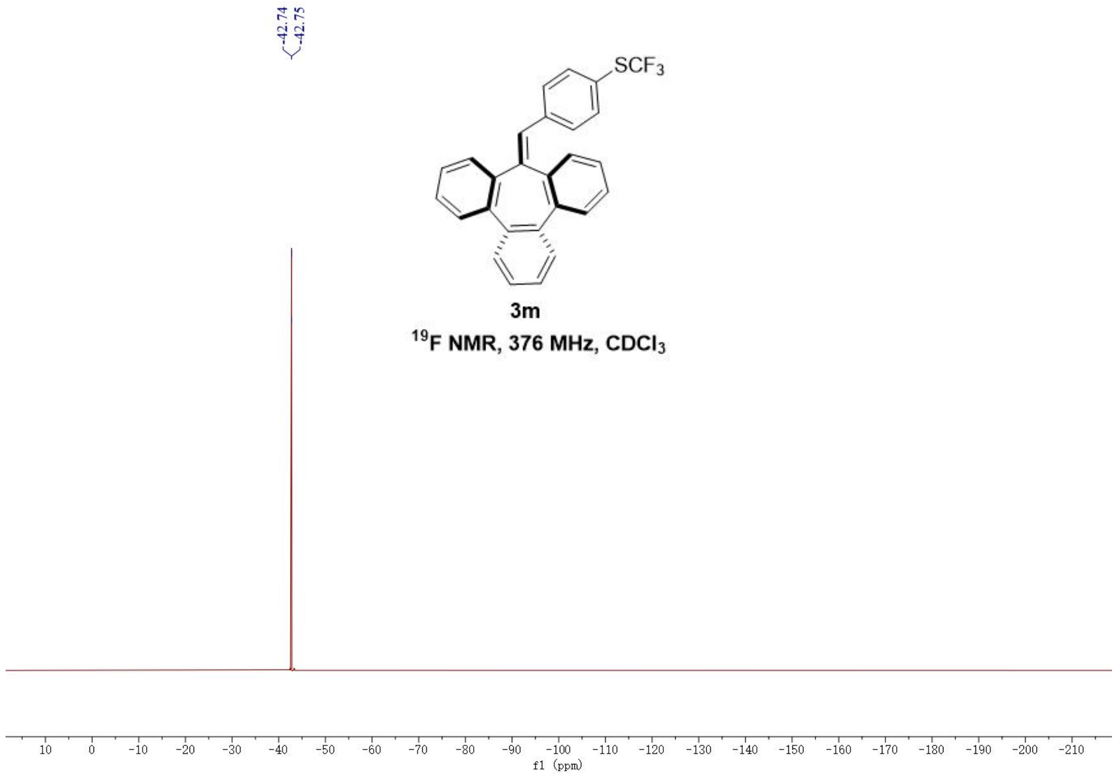
-42.74
-42.75
SCF3
3m
19F NMR, 376 MHz, CDCl3
10 0 −10 −20 −30 −40 −50 −60 −70 −80 −90 −100 −110 −120 −130 −140 −150 −160 −170 −180 −190 −200 −210
f1 (ppm)

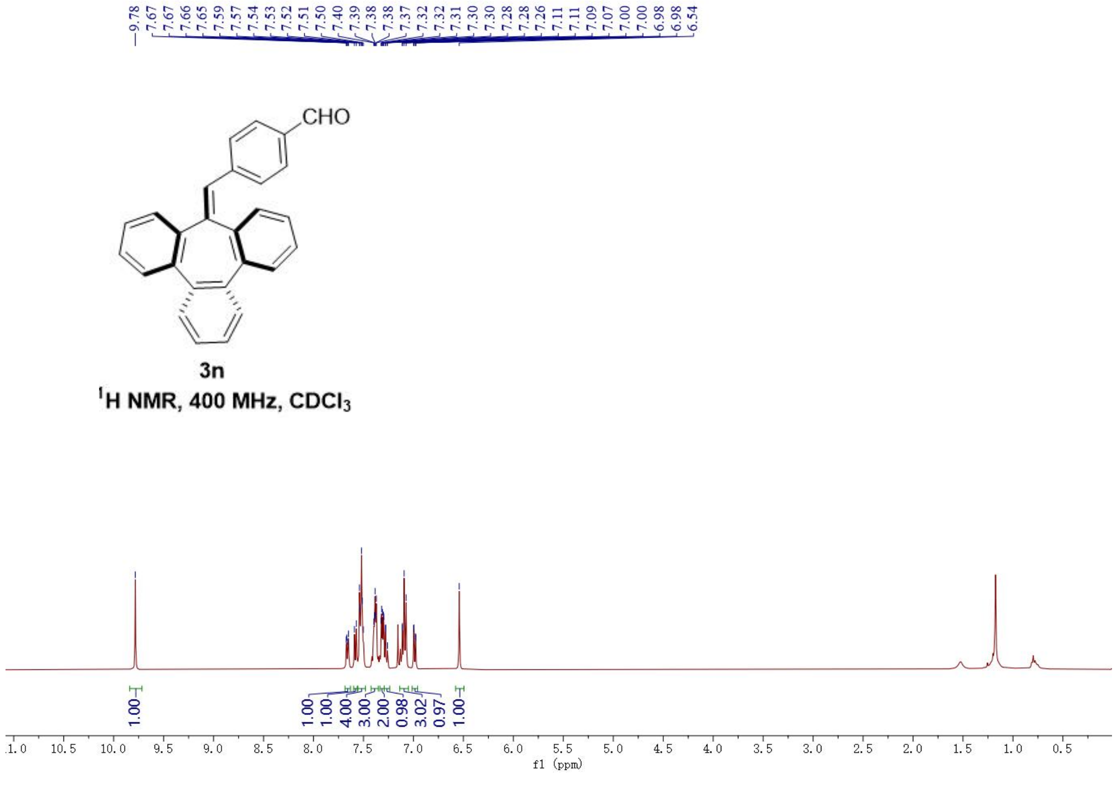
9.78 7.67 7.66 7.65 7.59 7.57 7.54 7.53 7.52 7.51 7.50 7.40 7.39 7.38 7.38 7.37 7.32 7.32 7.31 7.30 7.30 7.28 7.28 7.26 7.11 7.11 7.09 7.07 7.00 7.00 6.98 6.98 6.54
CHO
3n
1H NMR, 400 MHz, CDCl3
1.00 1.00 1.00 4.00 3.00 2.00 0.98 3.02 0.97 1.00
11.0 10.5 10.0 9.5 9.0 8.5 8.0 7.5 7.0 6.5 6.0 5.5 5.0 4.5 4.0 3.5 3.0 2.5 2.0 1.5 1.0 0.5
f1 (ppm)

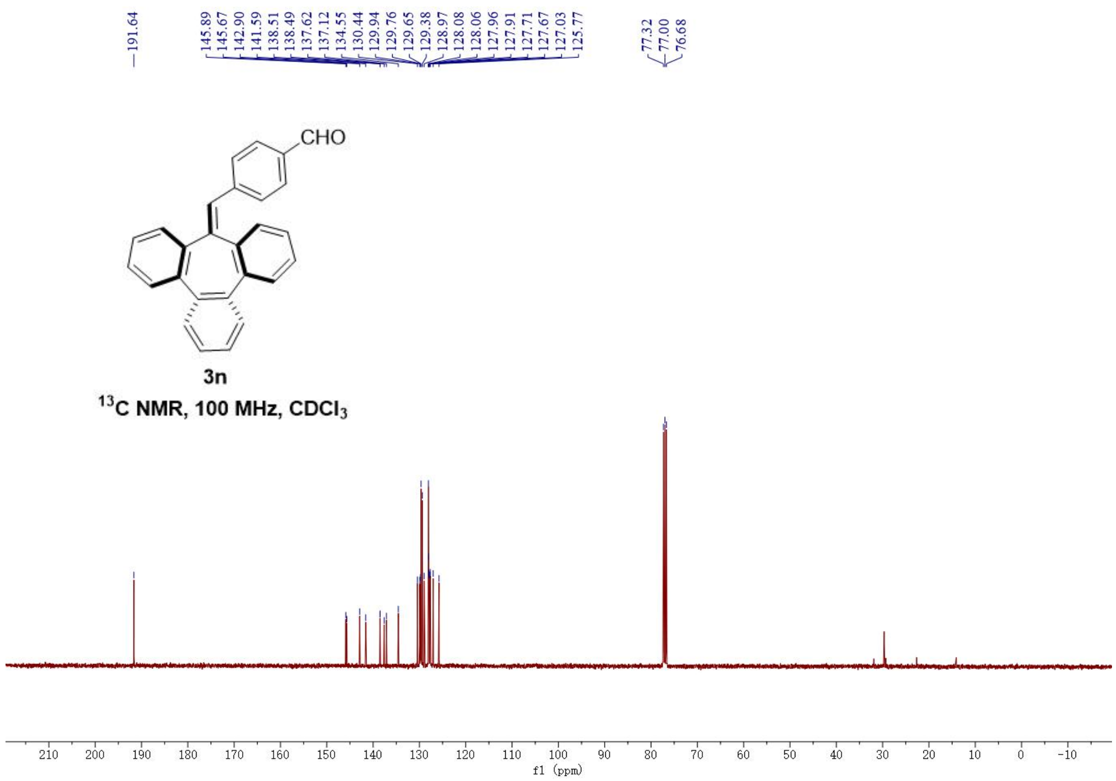

3n

^{13}C NMR, 100 MHz, $CDCl_3$

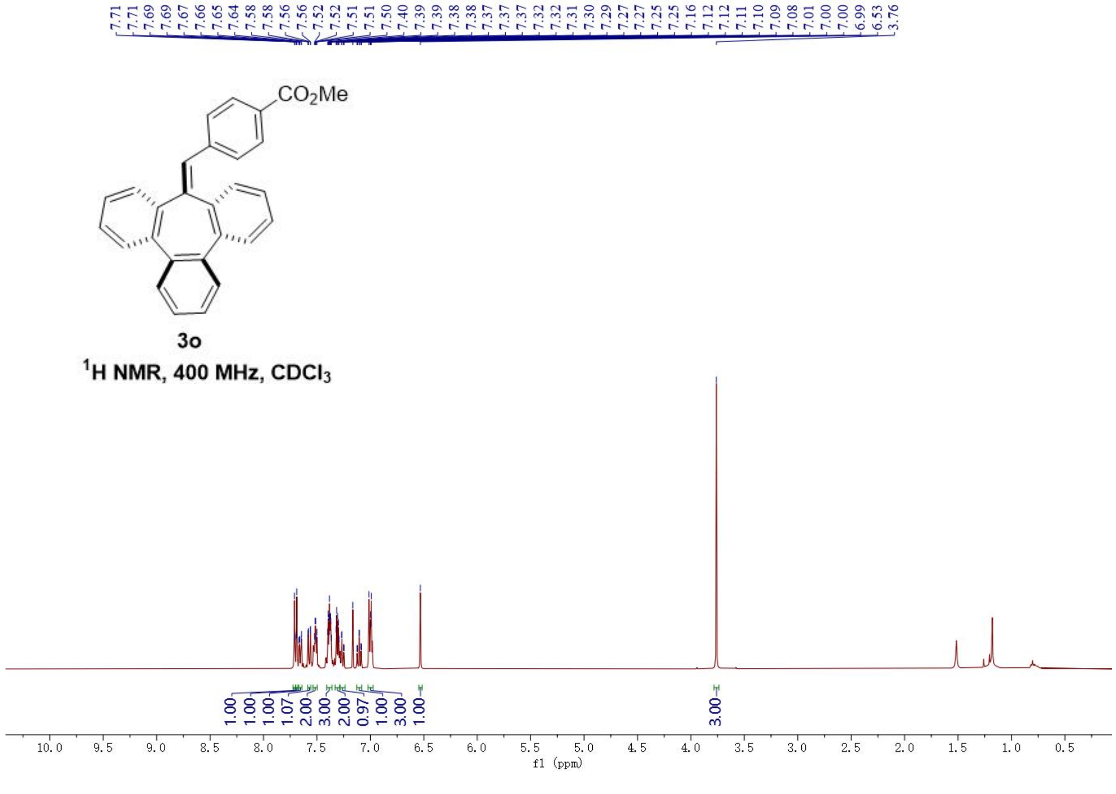

3o

^{1}H NMR, 400 MHz, $CDCl_3$

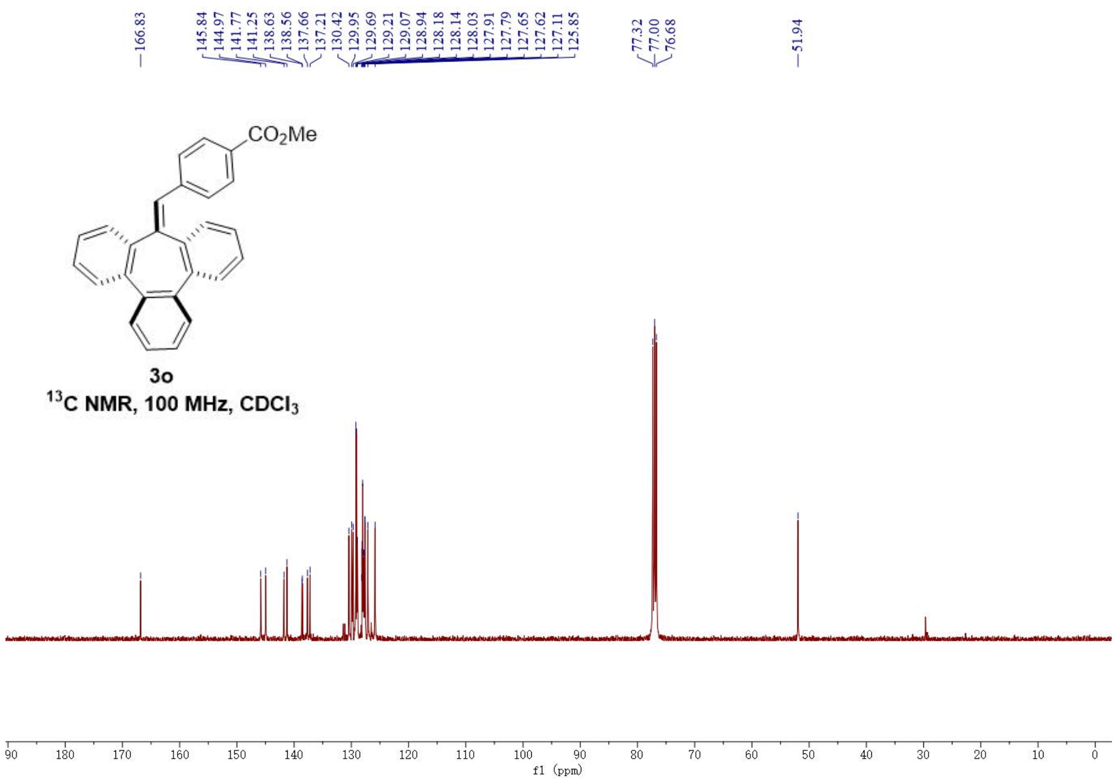

3o

^{13}C NMR, 100 MHz, CDCl$_3$

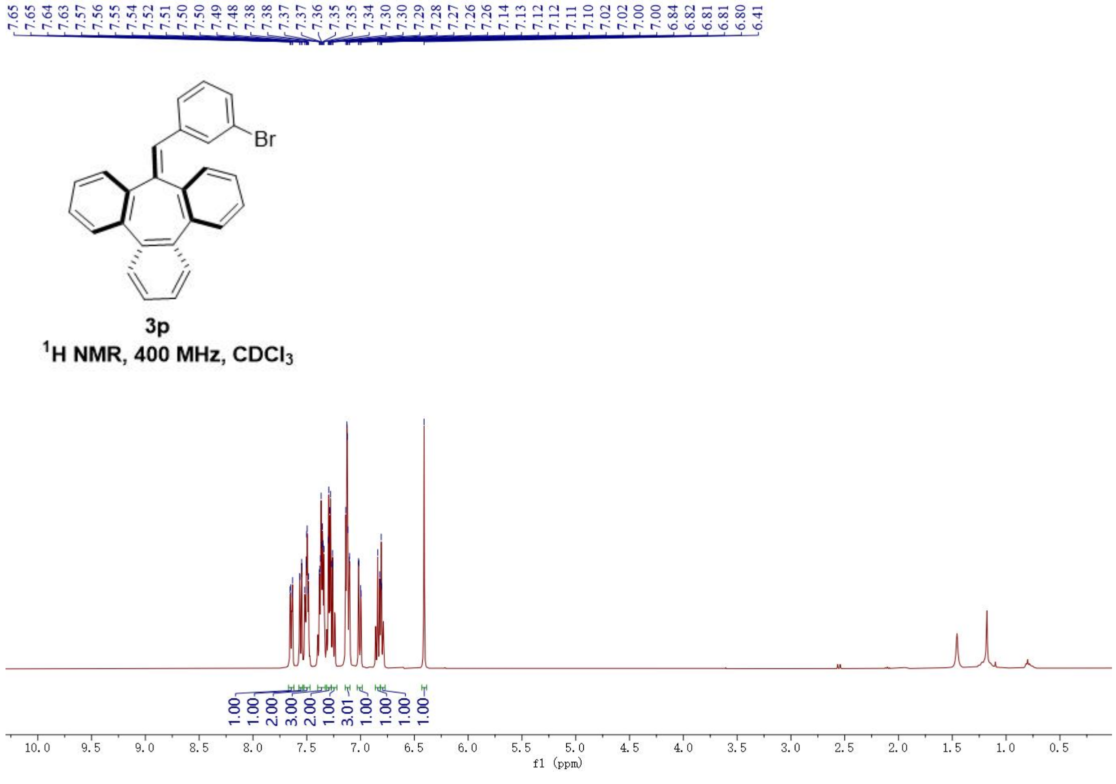

3p

^{1}H NMR, 400 MHz, CDCl$_3$

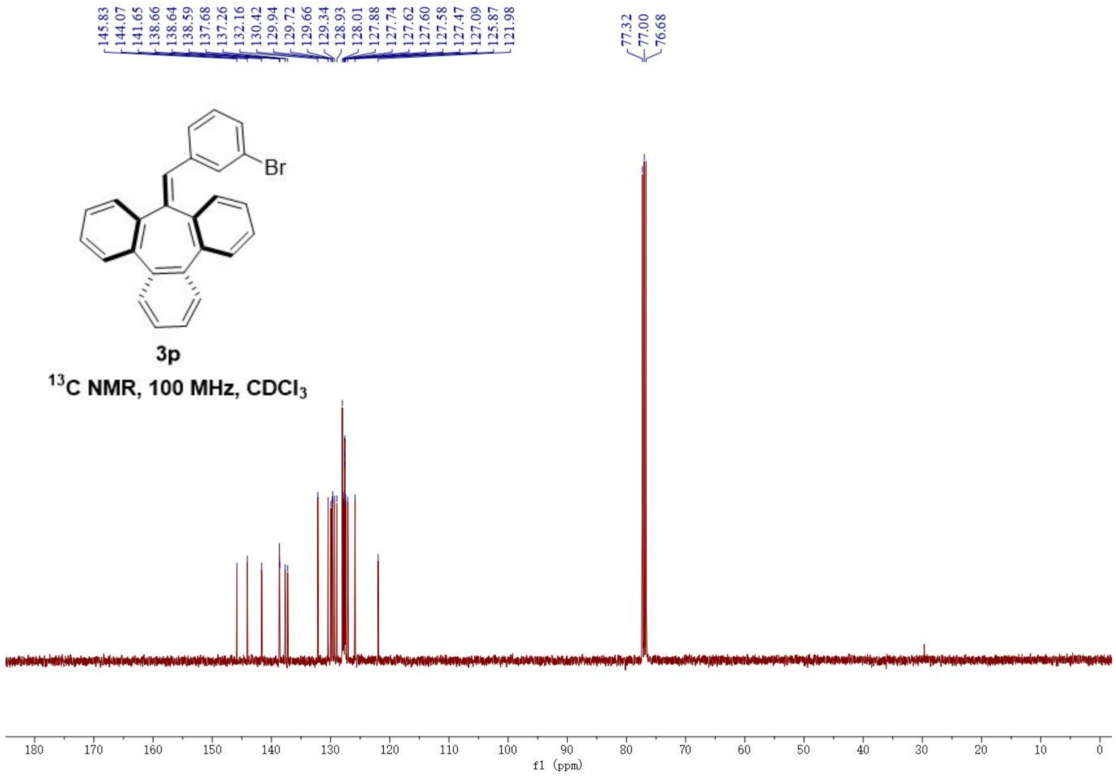
145.83 144.07 141.65 138.66 138.64 138.59 137.68 137.26 132.16 130.42 129.94 129.72 129.66 129.34 128.93 128.01 127.88 127.74 127.62 127.60 127.58 127.47 127.09 125.87 121.98
77.32 77.00 76.68
Br
3p
^{13}C NMR, 100 MHz, CDCl$_3$
180 170 160 150 140 130 120 110 100 90 80 70 60 50 40 30 20 10 0
f1 (ppm)

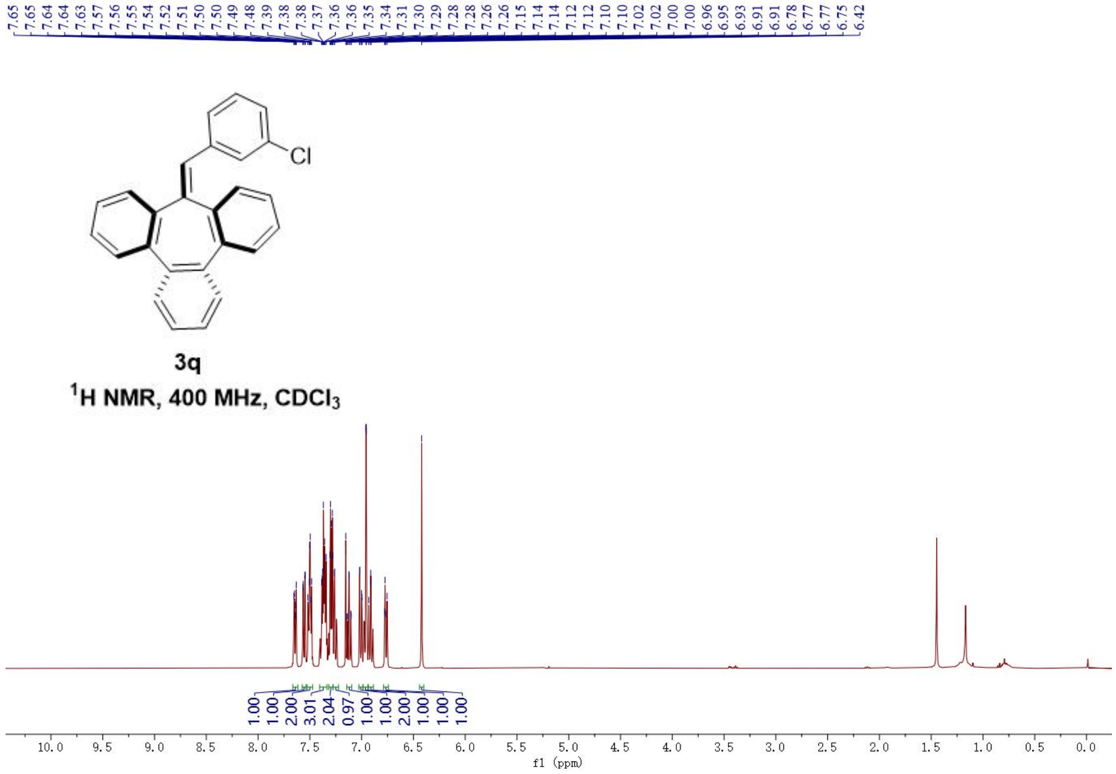
7.65 7.64 7.64 7.63 7.57 7.56 7.55 7.54 7.52 7.51 7.50 7.49 7.48 7.39 7.38 7.38 7.37 7.36 7.36 7.35 7.34 7.31 7.30 7.29 7.28 7.26 7.26 7.15 7.14 7.14 7.12 7.12 7.10 7.10 7.02 7.02 7.00 6.96 6.95 6.93 6.91 6.91 6.78 6.77 6.77 6.75 6.42
Cl
3q
^{1}H NMR, 400 MHz, CDCl$_3$
1.00 1.00 2.00 3.01 2.04 0.97 1.00 1.00 2.00 1.00 1.00
10.0 9.5 9.0 8.5 8.0 7.5 7.0 6.5 6.0 5.5 5.0 4.5 4.0 3.5 3.0 2.5 2.0 1.5 1.0 0.5 0.0
f1 (ppm)

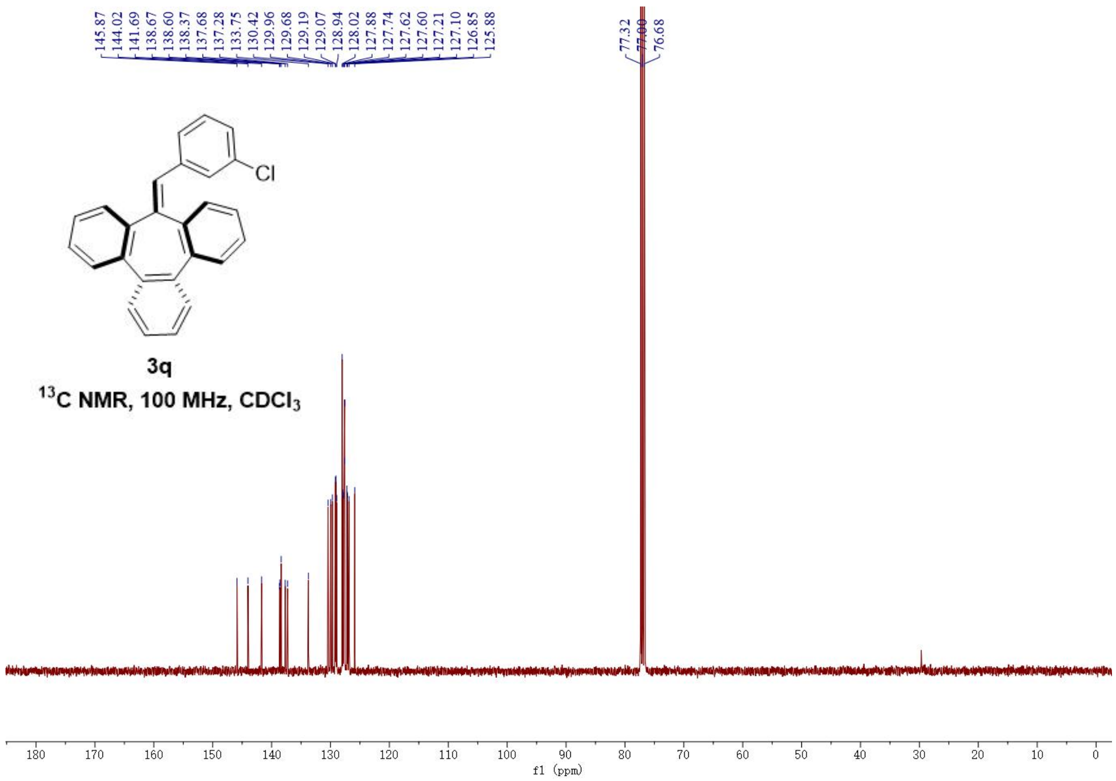
145.87
144.02
141.69
138.67
138.60
138.37
137.68
137.28
133.75
130.42
129.96
129.68
129.19
129.07
128.94
128.02
127.88
127.74
127.62
127.60
127.21
127.10
126.85
125.88
77.32
77.00
76.68
Cl
3q
^{13}C NMR, 100 MHz, CDCl$_3$
180 170 160 150 140 130 120 110 100 90 80 70 60 50 40 30 20 10 0
f1 (ppm)

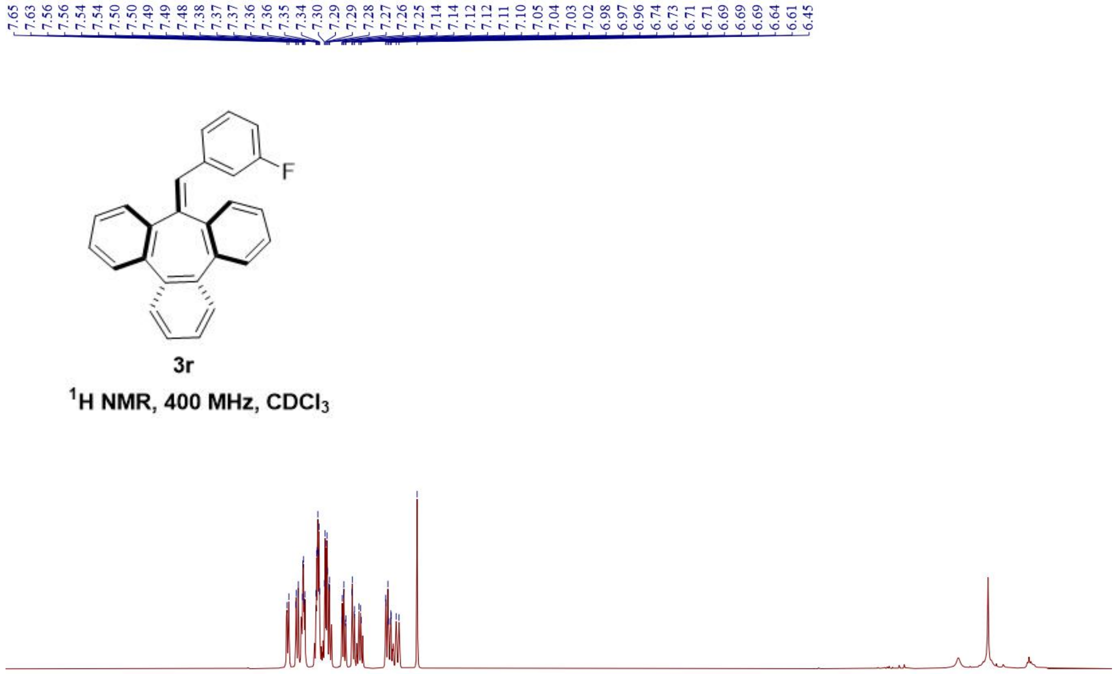
7.65
7.63
7.56
7.56
7.54
7.54
7.50
7.50
7.49
7.49
7.48
7.38
7.37
7.37
7.36
7.35
7.34
7.30
7.29
7.29
7.28
7.27
7.26
7.25
7.14
7.14
7.12
7.12
7.11
7.10
7.05
7.04
7.03
7.02
6.98
6.97
6.96
6.74
6.73
6.71
6.71
6.69
6.69
6.69
6.64
6.61
6.45
F
3r
^{1}H NMR, 400 MHz, CDCl$_3$
1.00
1.00
2.00
3.00
2.00
0.97
1.03
1.00
1.00
2.00
1.00
1.00
10.0 9.5 9.0 8.5 8.0 7.5 7.0 6.5 6.0 5.5 5.0 4.5 4.0 3.5 3.0 2.5 2.0 1.5 1.0 0.5
f1 (ppm)

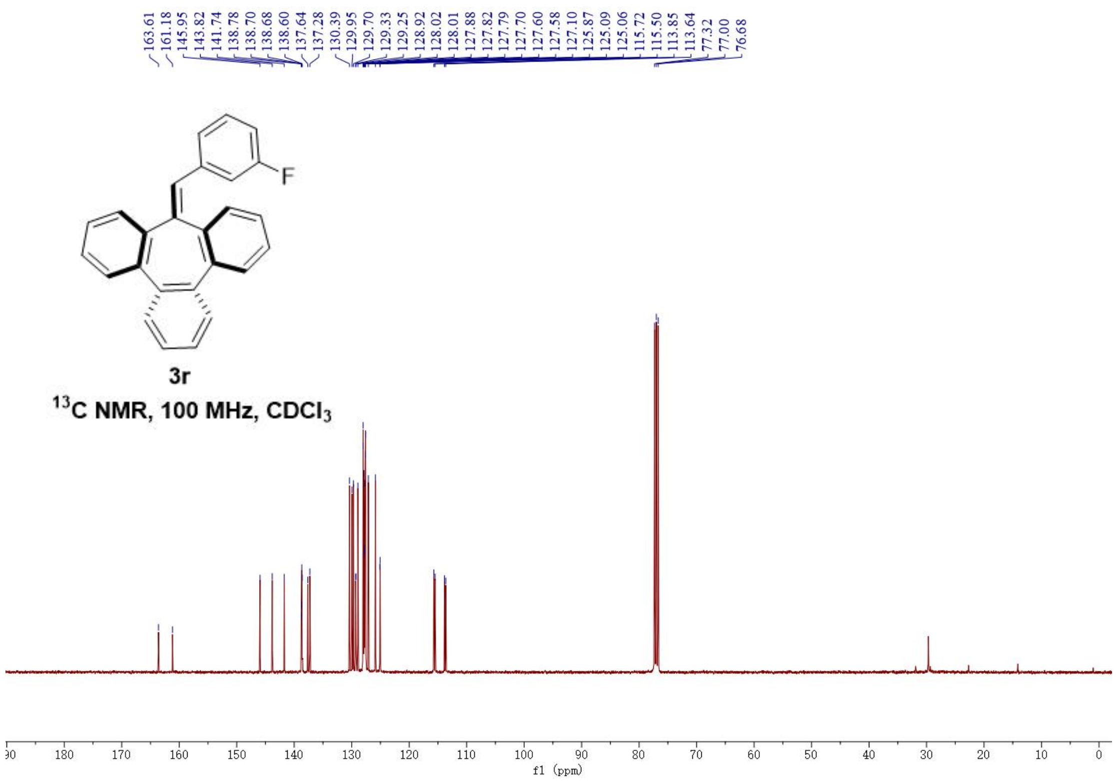
163.61
161.18
145.95
143.82
141.74
138.78
138.70
138.68
138.60
137.64
137.28
130.39
129.95
129.70
129.33
129.25
128.92
128.02
128.01
127.88
127.82
127.79
127.70
127.60
127.58
127.10
125.87
125.09
125.06
115.72
115.50
113.85
113.64
77.32
77.00
76.68
F
3r
^{13}C NMR, 100 MHz, CDCl$_3$
f1 (ppm)

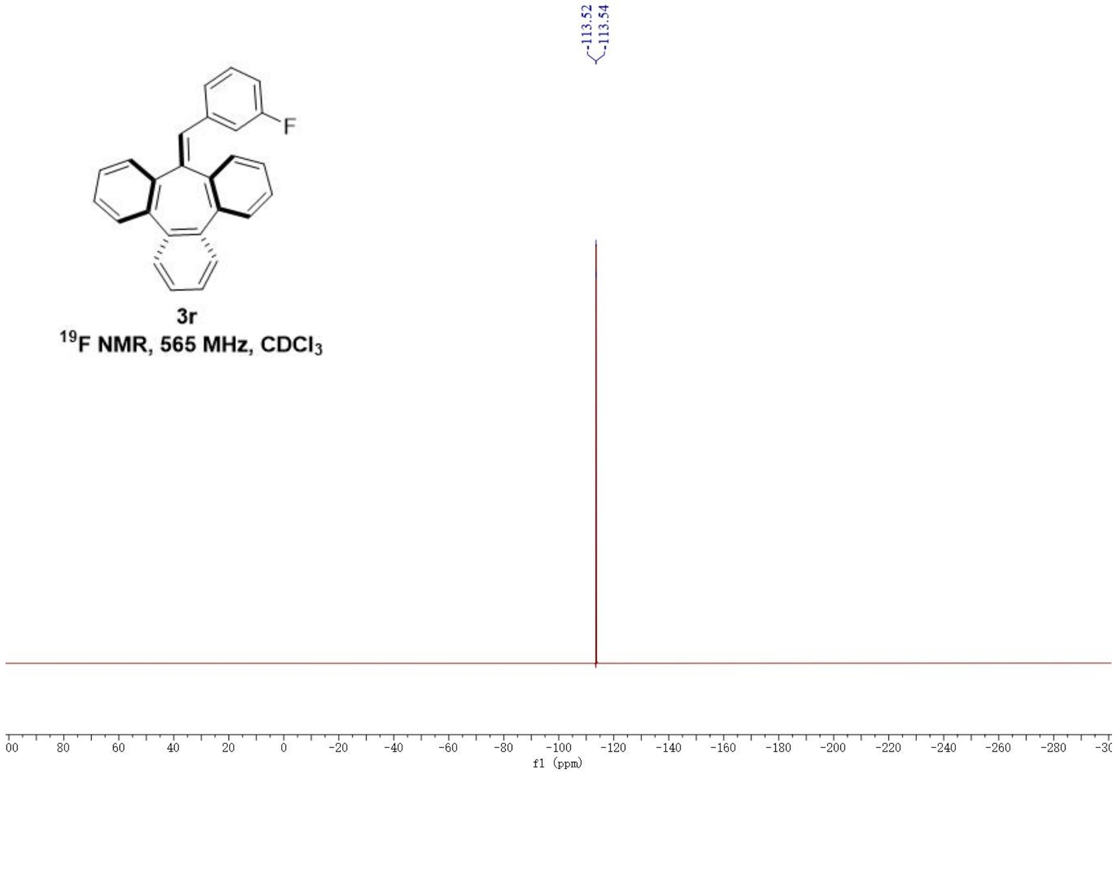
-113.52
-113.54
F
3r
^{19}F NMR, 565 MHz, CDCl$_3$
f1 (ppm)

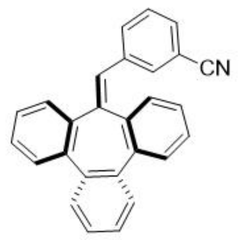

3s

^{1}H NMR, 400 MHz, $CDCl_3$

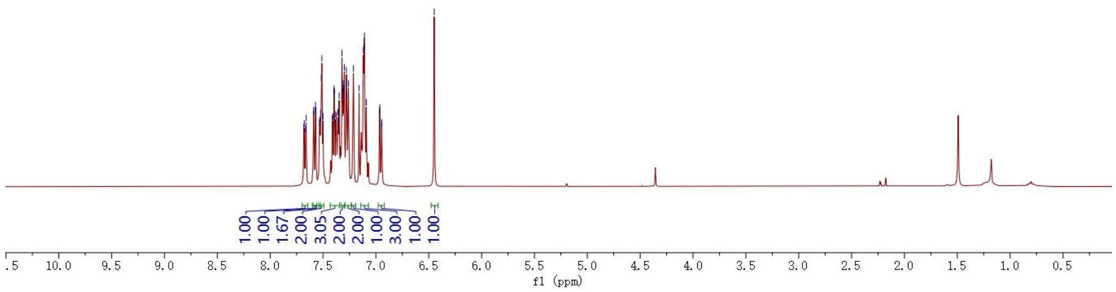

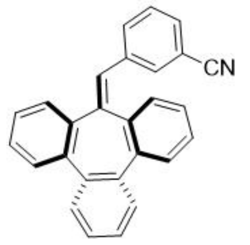

3s

^{13}C NMR, 100 MHz, $CDCl_3$

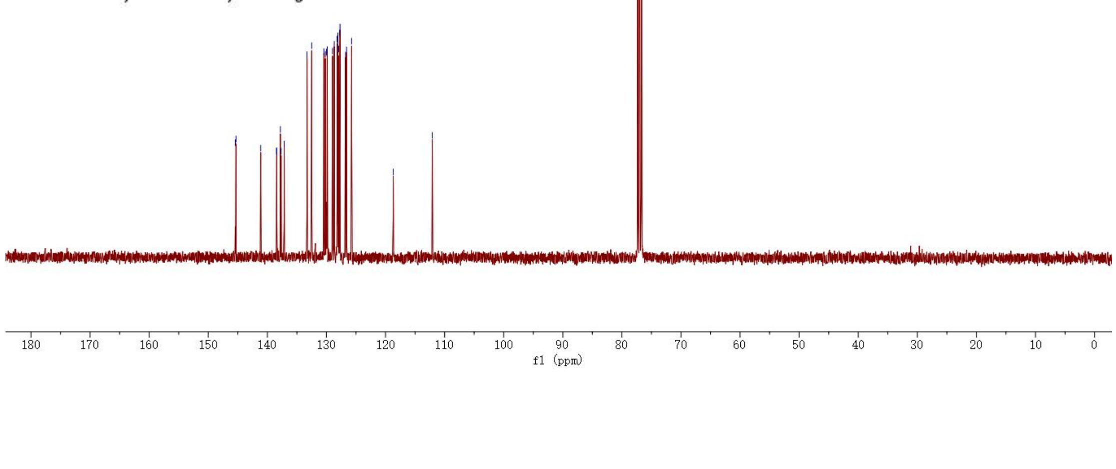

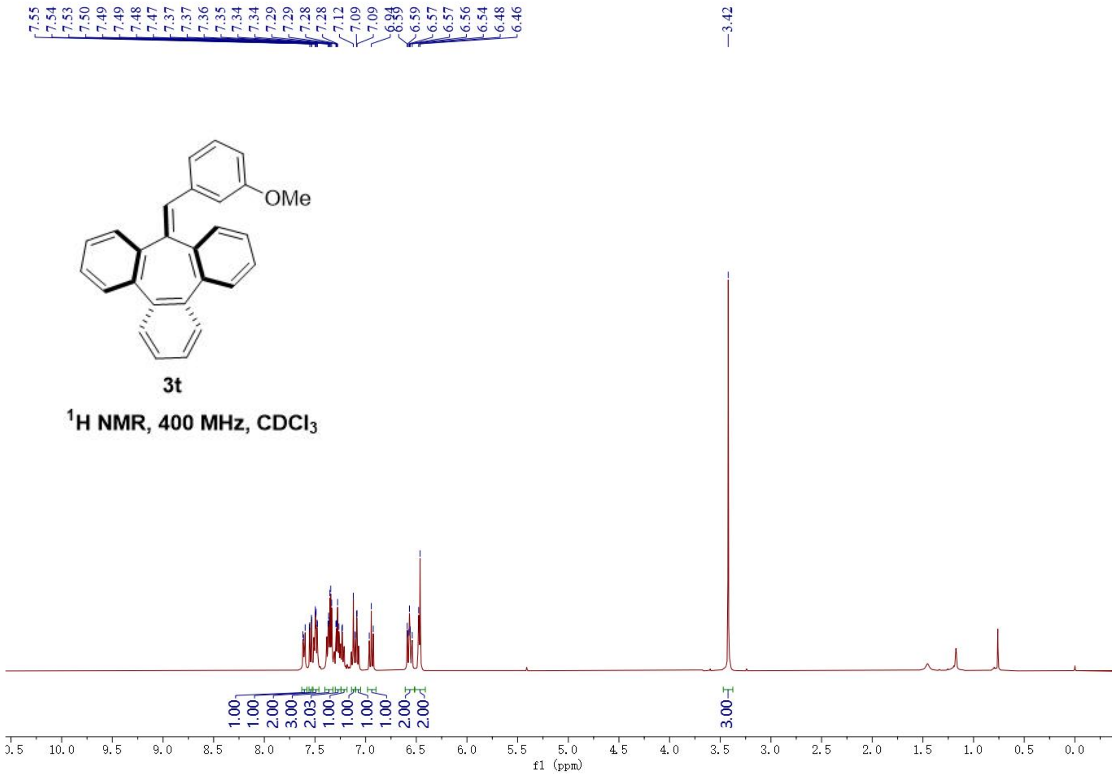
7.55 7.54 7.53 7.50 7.49 7.49 7.48 7.47 7.37 7.37 7.36 7.35 7.34 7.34 7.29 7.29 7.28 7.28 7.12 7.09 7.09 6.94 6.93 6.59 6.59 6.57 6.56 6.54 6.48 6.46
3.42
OMe
3t
^{1}H NMR, 400 MHz, CDCl$_3$
1.00 1.00 2.00 3.00 2.03 1.00 1.00 1.00 1.00 2.00 2.00
3.00
f1 (ppm)

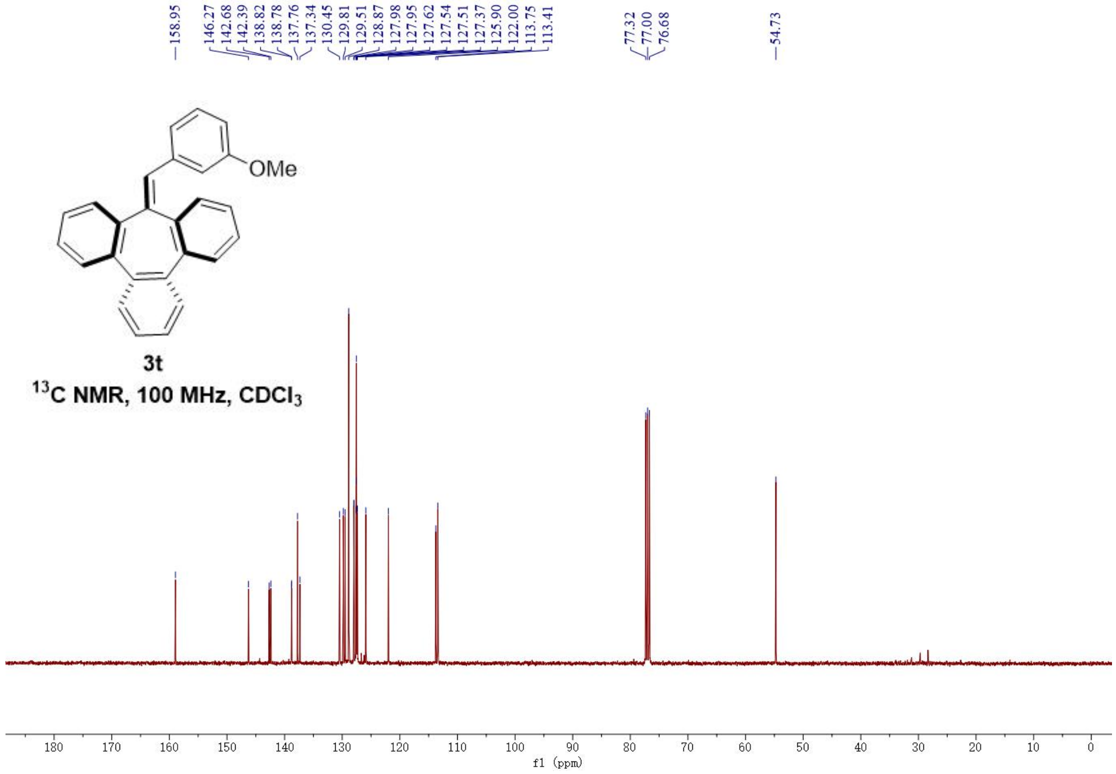
158.95
146.27 142.68 142.39 138.82 138.78 137.76 137.34 130.45 129.81 129.51 128.87 127.98 127.95 127.62 127.54 127.51 127.37 125.90 122.00 113.75 113.41
77.32 77.00 76.68
54.73
OMe
3t
^{13}C NMR, 100 MHz, CDCl$_3$
f1 (ppm)

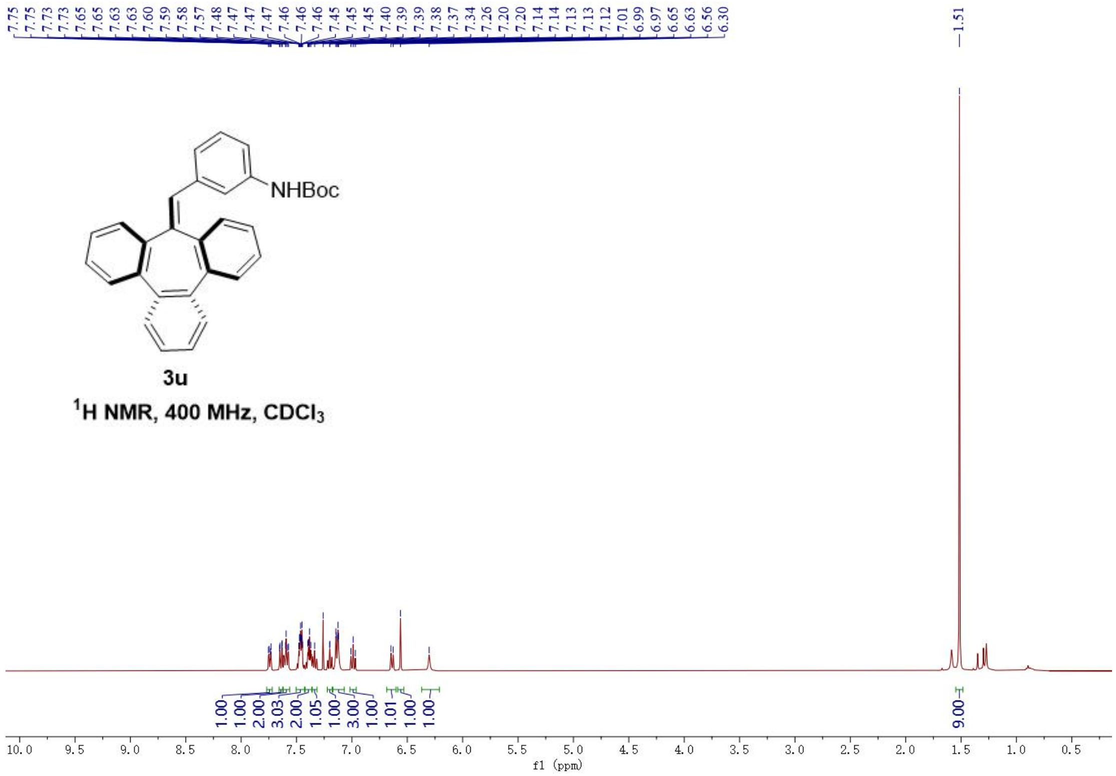
NHBoc
3u
1H NMR, 400 MHz, CDCl3

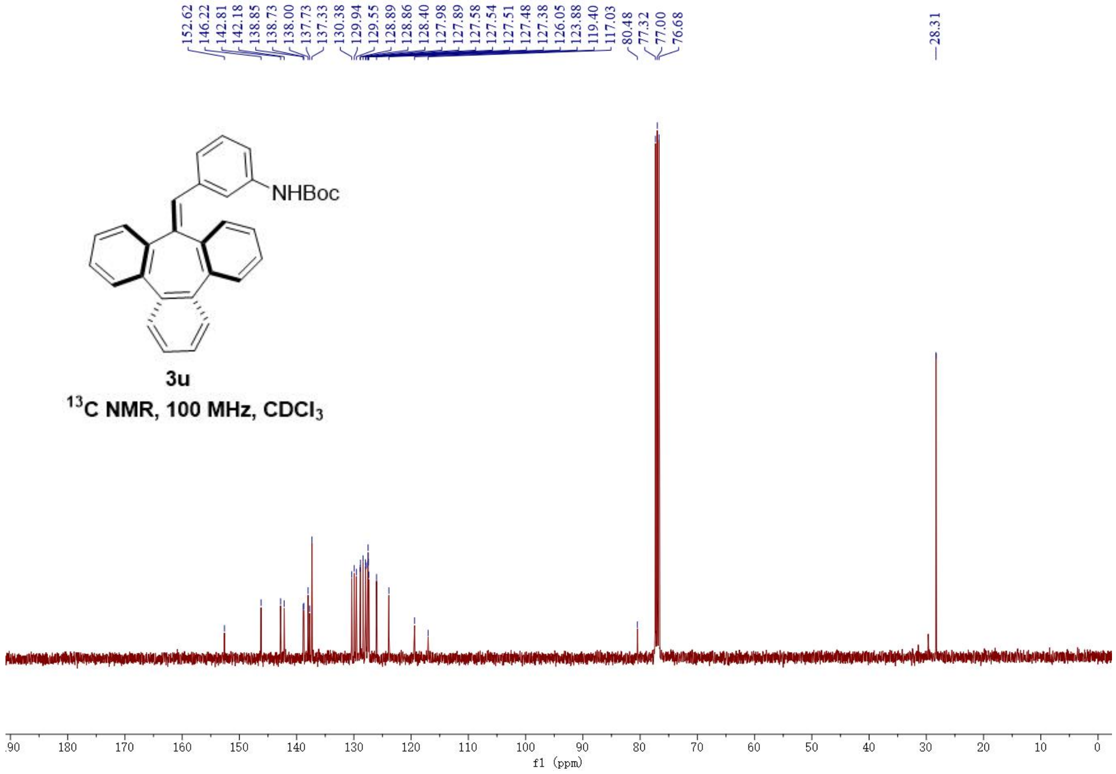
NHBoc
3u
13C NMR, 100 MHz, CDCl3

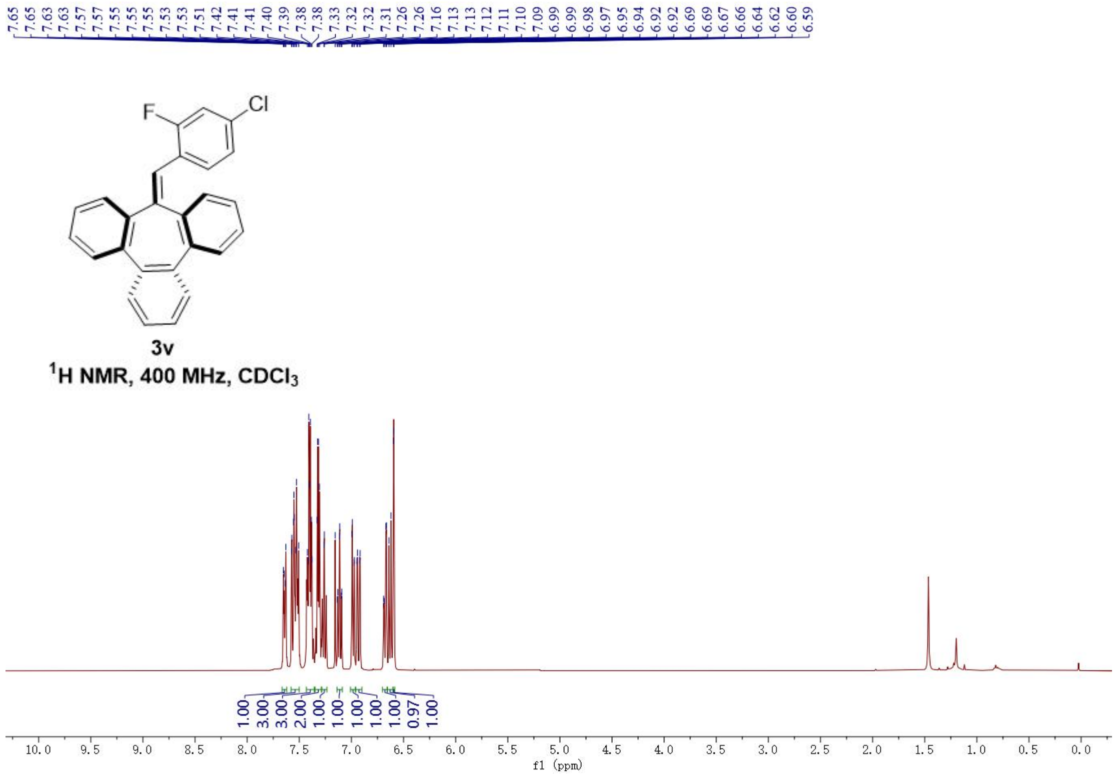
7.65 7.65 7.63 7.63 7.57 7.57 7.55 7.55 7.55 7.53 7.53 7.51 7.42 7.41 7.41 7.40 7.39 7.38 7.38 7.33 7.32 7.32 7.31 7.26 7.26 7.16 7.13 7.13 7.12 7.11 7.10 7.09 6.99 6.99 6.98 6.97 6.95 6.94 6.92 6.92 6.69 6.69 6.67 6.66 6.64 6.62 6.60 6.59
F
Cl
3v
1H NMR, 400 MHz, CDCl3
1.00 3.00 3.00 2.00 1.00 1.00 1.00 1.00 1.00 0.97 1.00
f1 (ppm)

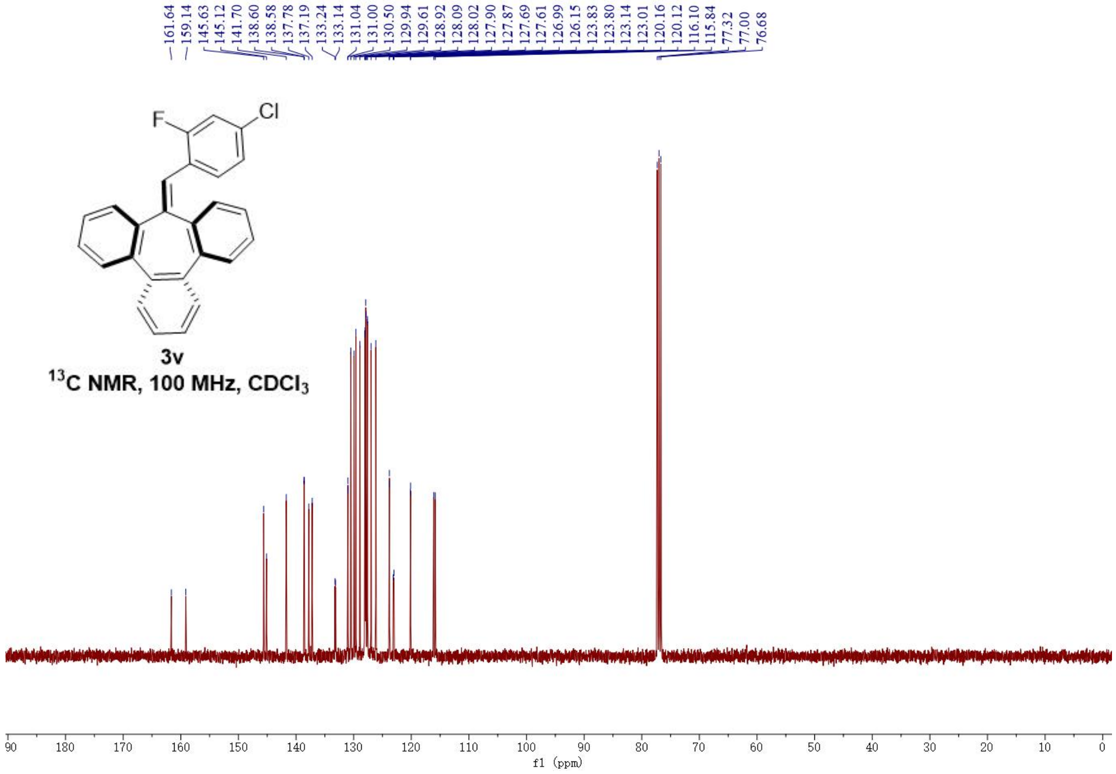
161.64 159.14 145.63 145.12 141.70 138.60 138.58 137.78 137.19 133.24 133.14 131.04 131.00 130.50 129.94 129.61 128.92 128.09 128.02 127.90 127.87 127.69 127.61 126.99 126.15 123.83 123.80 123.14 123.01 120.16 120.12 116.10 115.84 77.32 77.00 76.68
F
Cl
3v
13C NMR, 100 MHz, CDCl3
f1 (ppm)

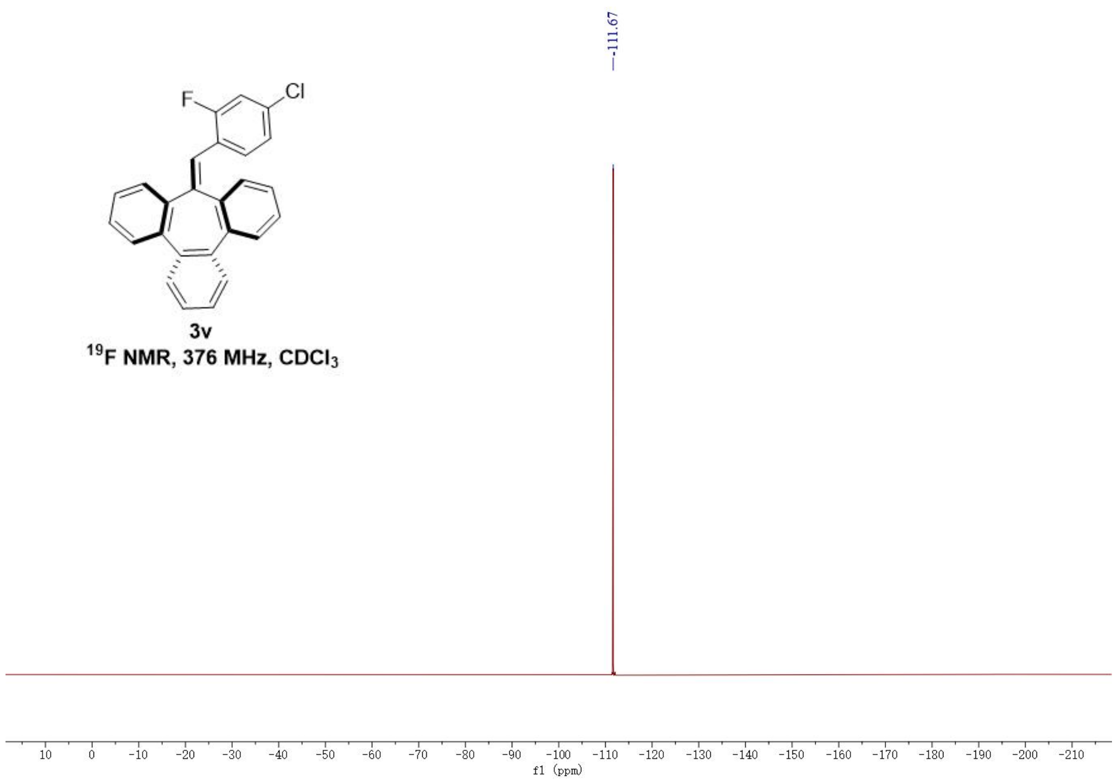

3v
^{19}F NMR, 376 MHz, $CDCl_3$

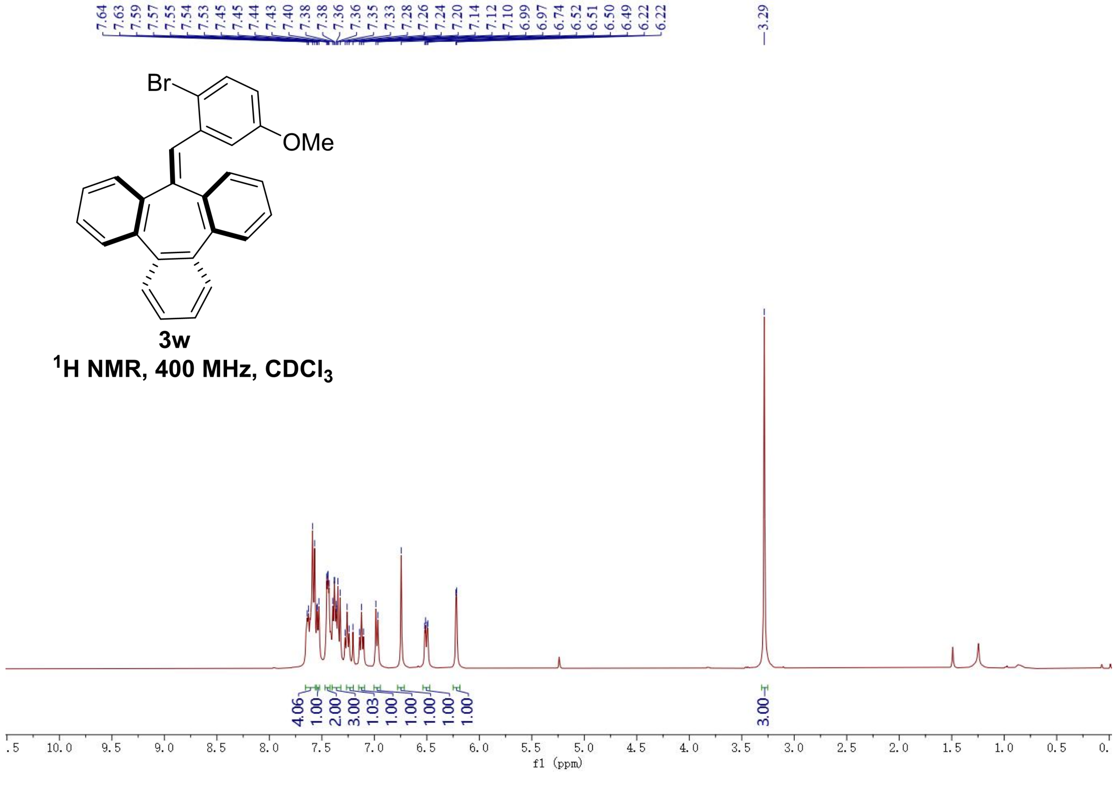

3w
^{1}H NMR, 400 MHz, $CDCl_3$

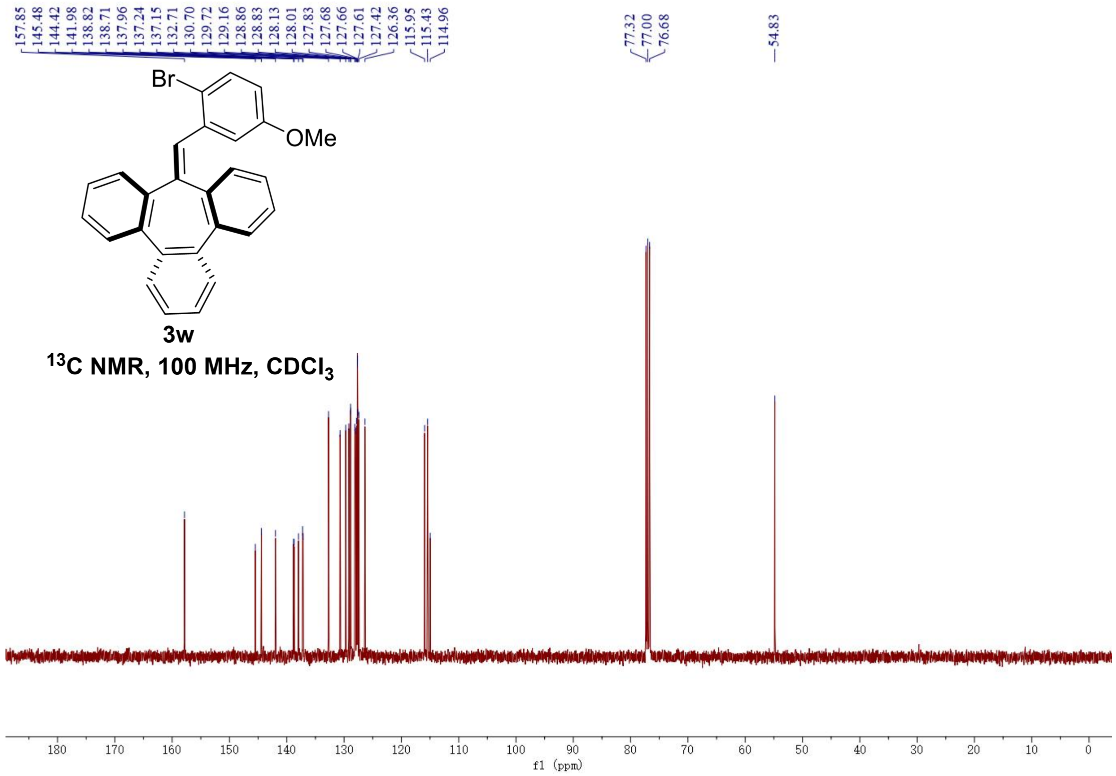
157.85
145.48
144.42
141.98
138.82
138.71
137.96
137.24
137.15
132.71
130.70
129.72
129.16
128.86
128.83
128.13
128.01
127.83
127.68
127.66
127.61
127.42
126.36
115.95
115.43
114.96
77.32
77.00
76.68
54.83
Br
OMe
3w
^{13}C NMR, 100 MHz, CDCl$_3$
f1 (ppm)

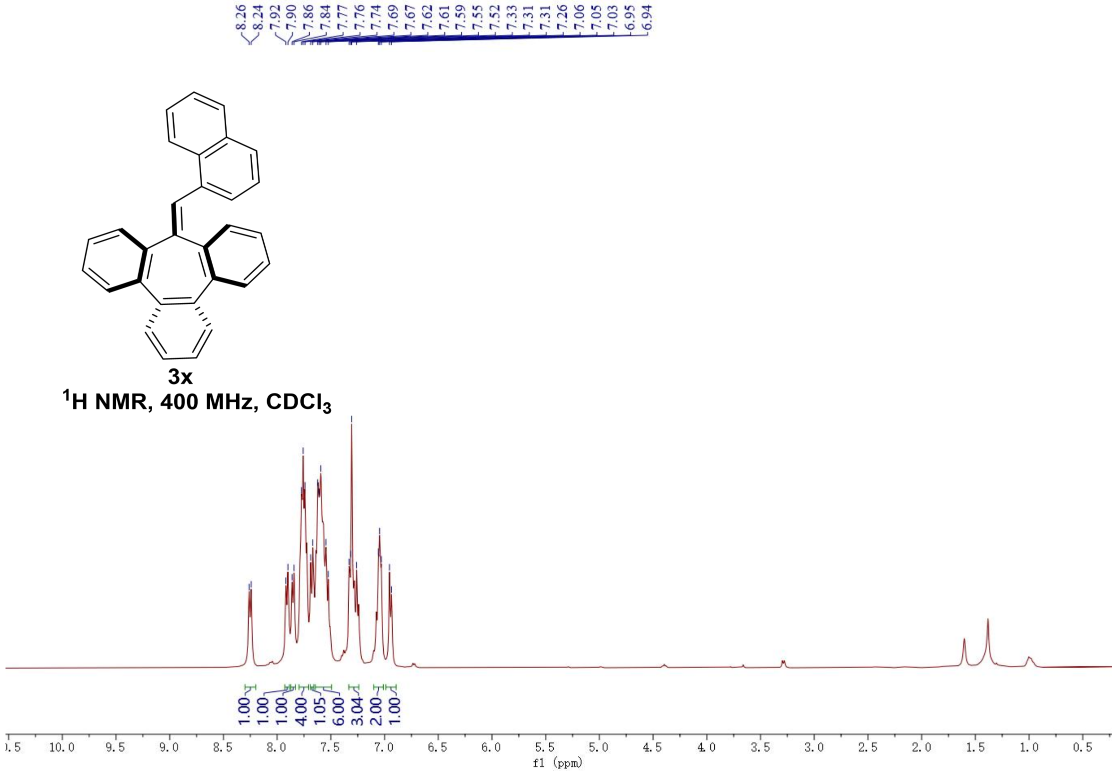
8.26
8.24
7.92
7.90
7.86
7.84
7.77
7.76
7.74
7.69
7.67
7.62
7.61
7.59
7.55
7.52
7.33
7.31
7.26
7.06
7.05
7.03
6.95
6.94
3x
^{1}H NMR, 400 MHz, CDCl$_3$
1.00
1.00
4.00
1.05
6.00
3.04
2.00
1.00
f1 (ppm)

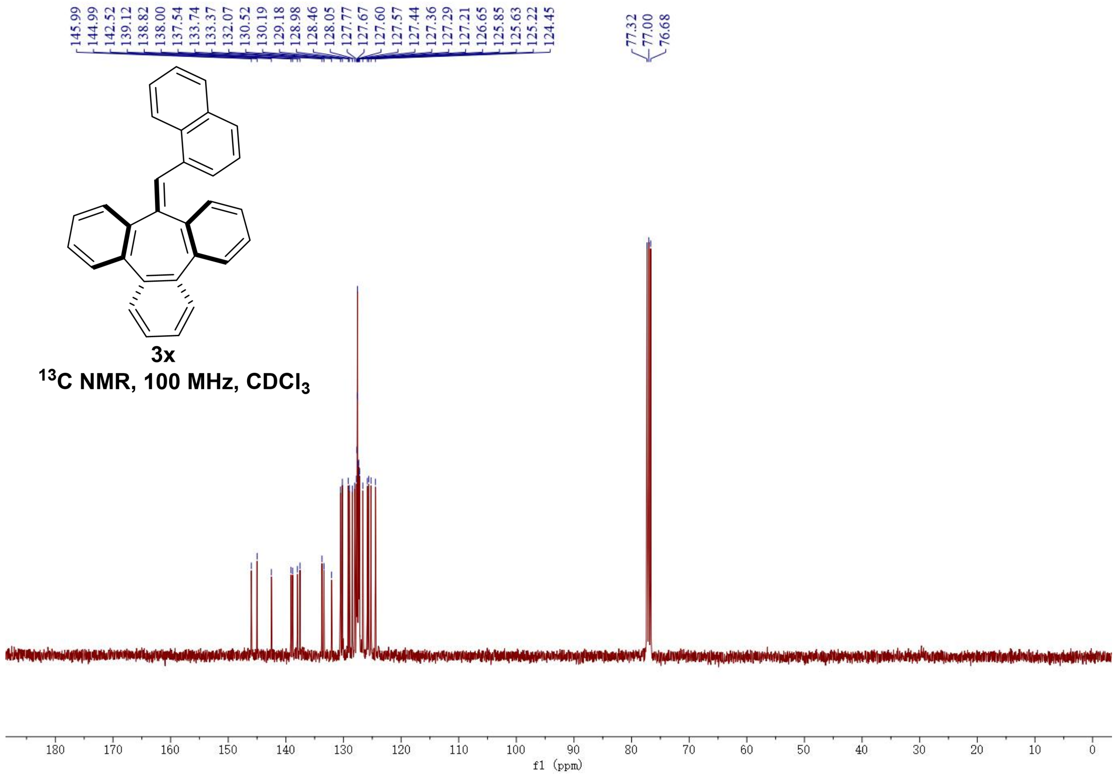
145.99
144.99
142.52
139.12
138.82
138.00
137.54
133.74
133.37
132.07
130.52
130.19
129.18
128.98
128.46
128.05
127.77
127.67
127.60
127.57
127.44
127.36
127.29
127.21
126.65
125.85
125.63
125.22
124.45
77.32
77.00
76.68
3x
^{13}C NMR, 100 MHz, CDCl$_3$
180
170
160
150
140
130
120
110
100
90
80
70
60
50
40
30
20
10
0
f1 (ppm)

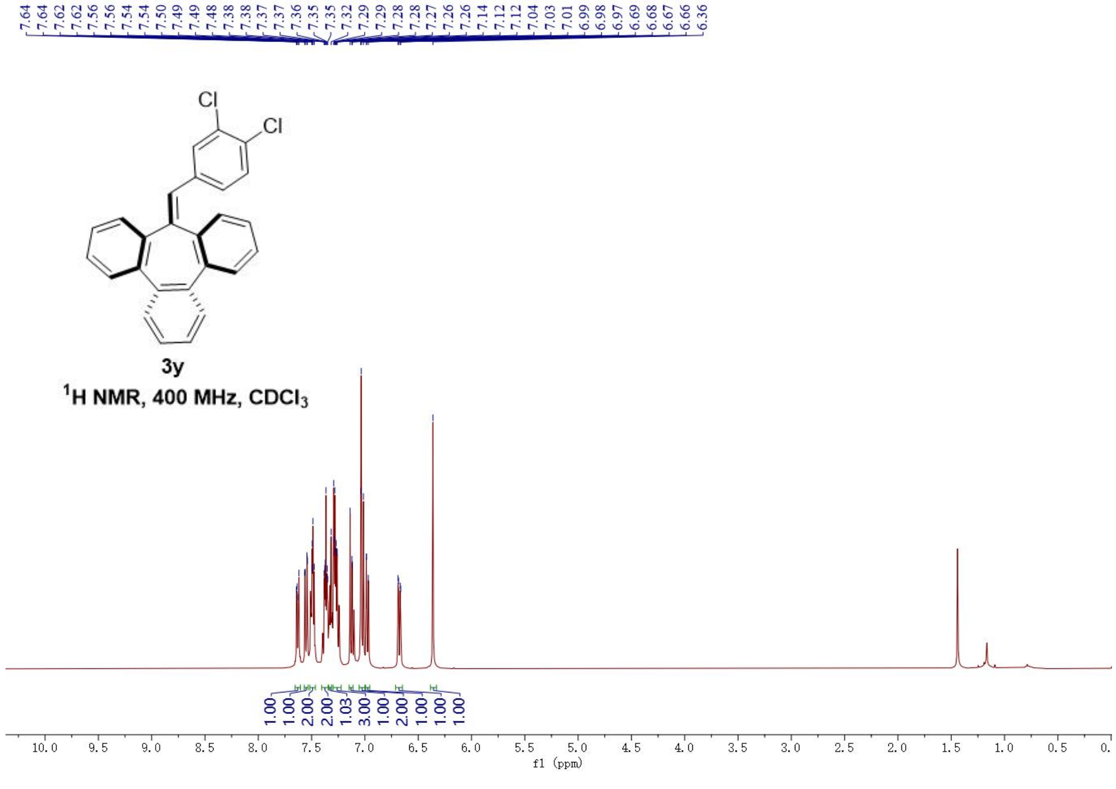
7.64
7.64
7.62
7.62
7.56
7.56
7.54
7.54
7.50
7.49
7.49
7.48
7.38
7.38
7.37
7.37
7.36
7.35
7.35
7.32
7.29
7.29
7.28
7.28
7.27
7.26
7.26
7.14
7.12
7.12
7.04
7.03
7.01
6.99
6.98
6.97
6.69
6.68
6.67
6.66
6.36
Cl
Cl
3y
^{1}H NMR, 400 MHz, CDCl$_3$
1.00
1.00
2.00
2.00
1.03
3.00
1.00
2.00
1.00
1.00
1.00
10.0
9.5
9.0
8.5
8.0
7.5
7.0
6.5
6.0
5.5
5.0
4.5
4.0
3.5
3.0
2.5
2.0
1.5
1.0
0.5
0.
f1 (ppm)

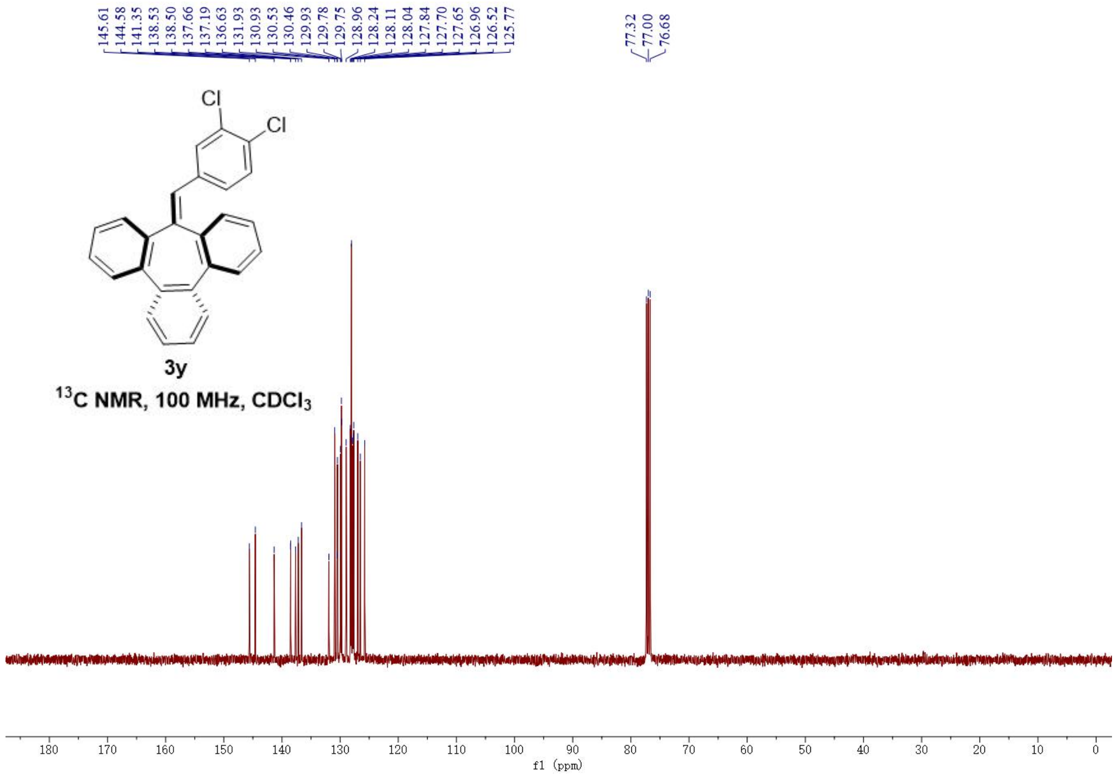

3y

^{13}C NMR, 100 MHz, $CDCl_3$

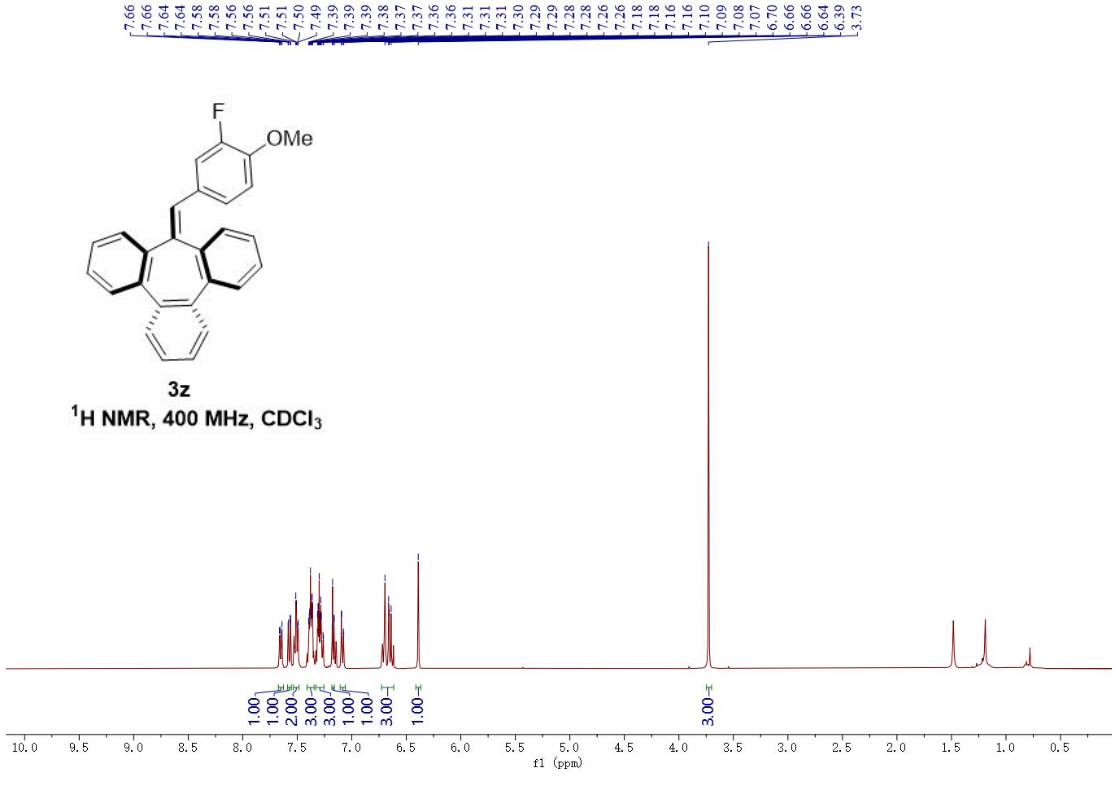

3z

^{1}H NMR, 400 MHz, $CDCl_3$

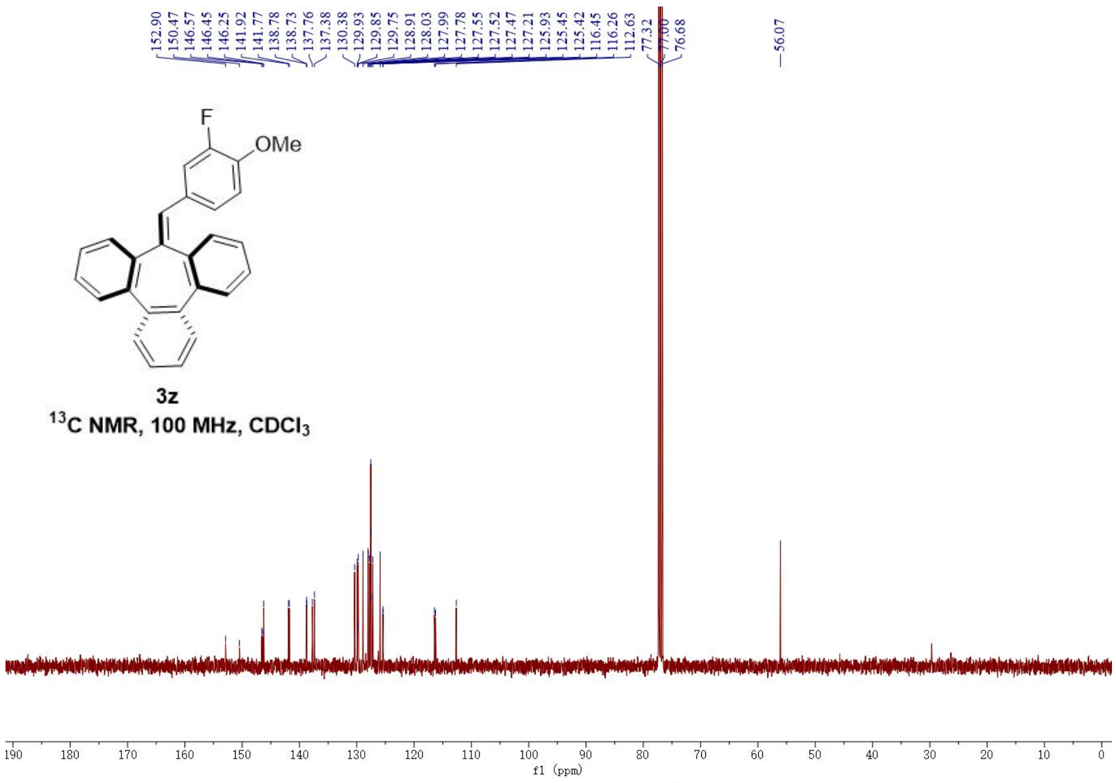

152.90
150.47
146.57
146.45
146.25
141.92
141.77
138.78
138.73
137.76
137.38
130.38
129.93
129.85
129.75
128.91
128.03
127.99
127.78
127.55
127.52
127.47
127.21
125.93
125.45
125.42
116.45
116.26
112.63
77.32
77.00
76.68
56.07
F
OMe
3z
^{13}C NMR, 100 MHz, CDCl$_3$
f1 (ppm)

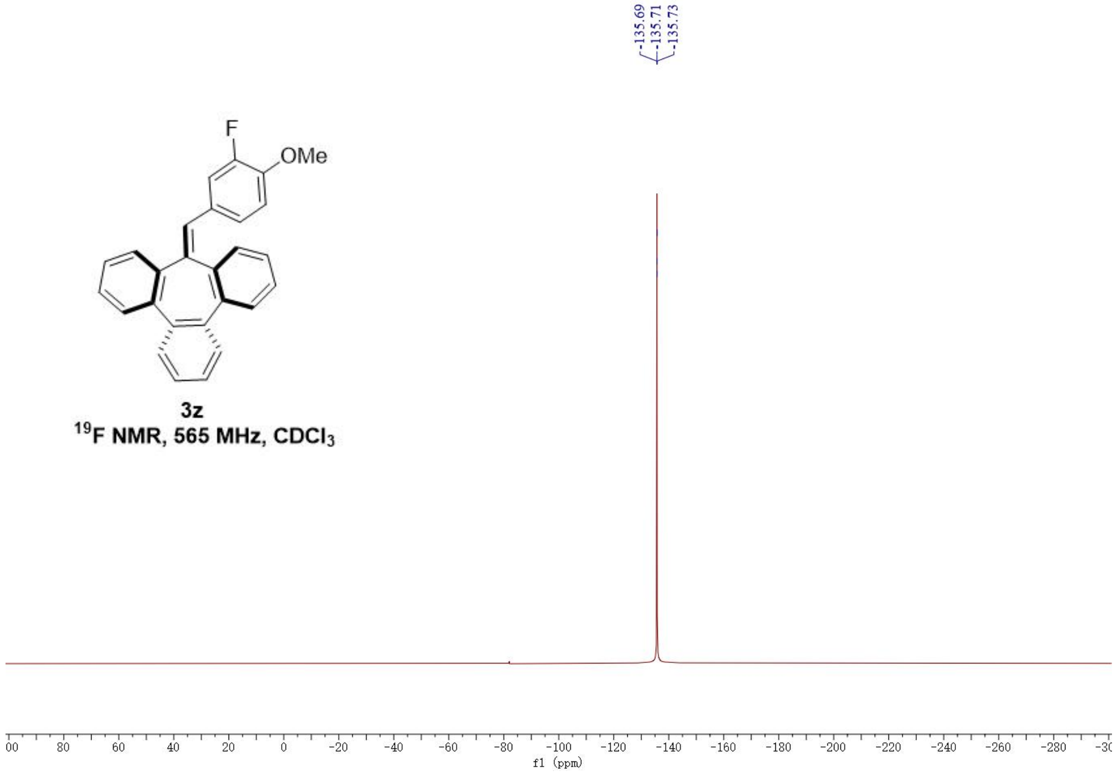

-135.69
-135.71
-135.73
F
OMe
3z
^{19}F NMR, 565 MHz, CDCl$_3$
f1 (ppm)

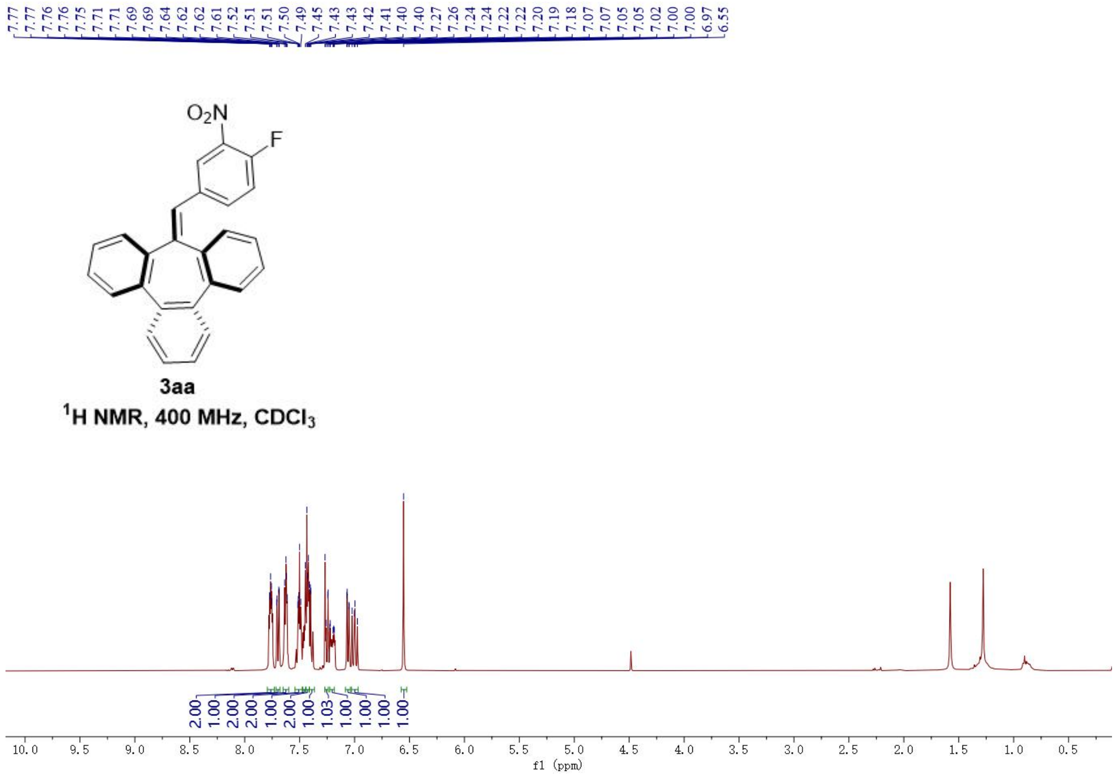

3aa

^{1}H NMR, 400 MHz, $CDCl_3$

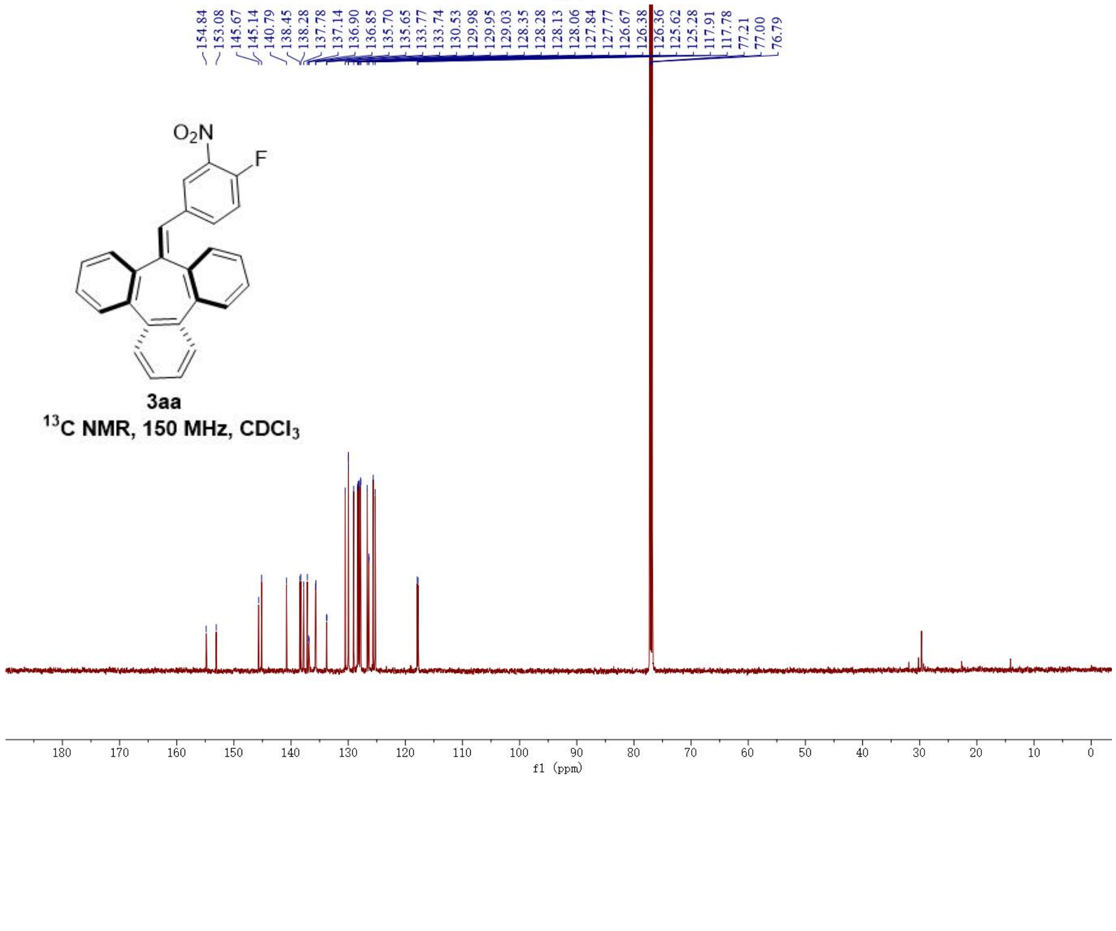

3aa

^{13}C NMR, 150 MHz, $CDCl_3$

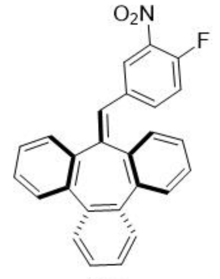

3aa

^{19}F NMR, 565 MHz, $CDCl_3$

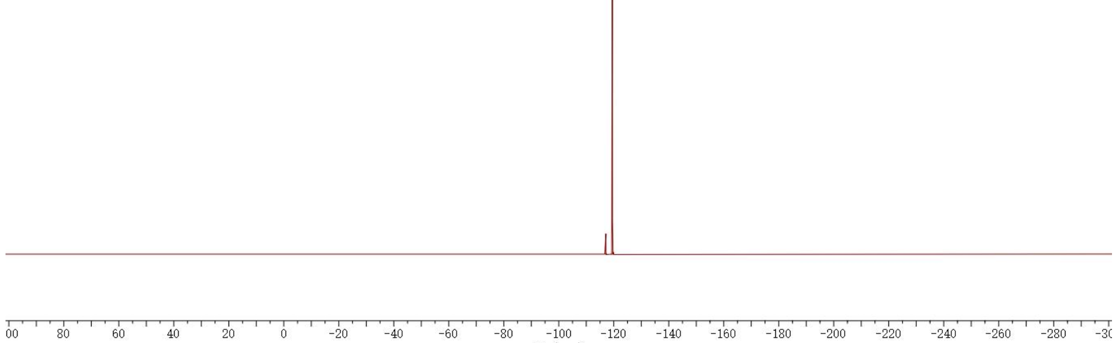

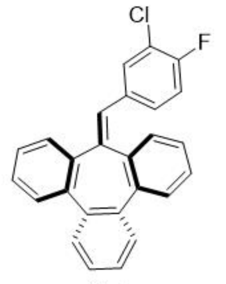

3ab

^{1}H NMR, 600 MHz, $CDCl_3$

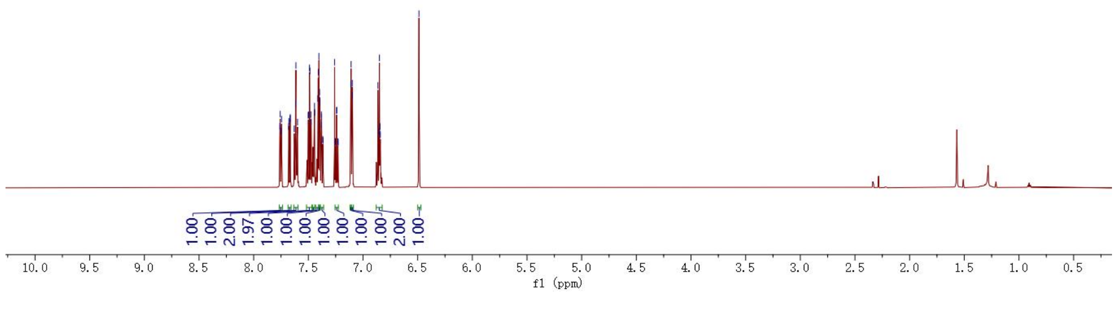

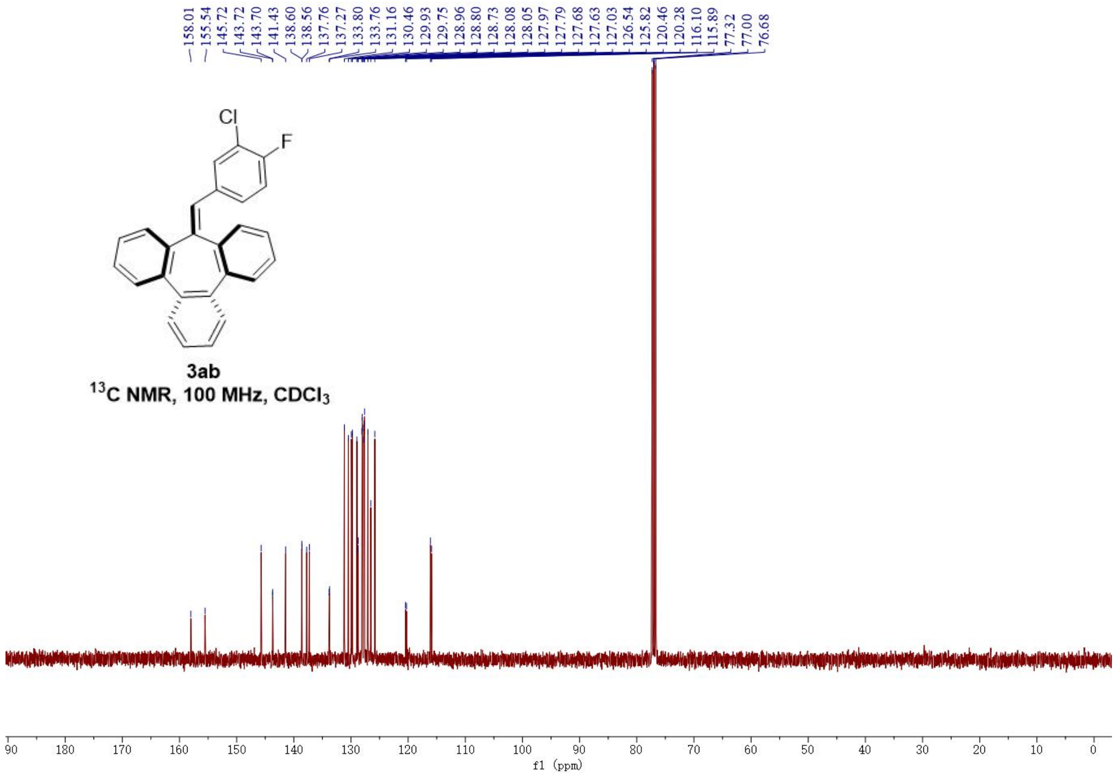

3ab

^{13}C NMR, 100 MHz, $CDCl_3$

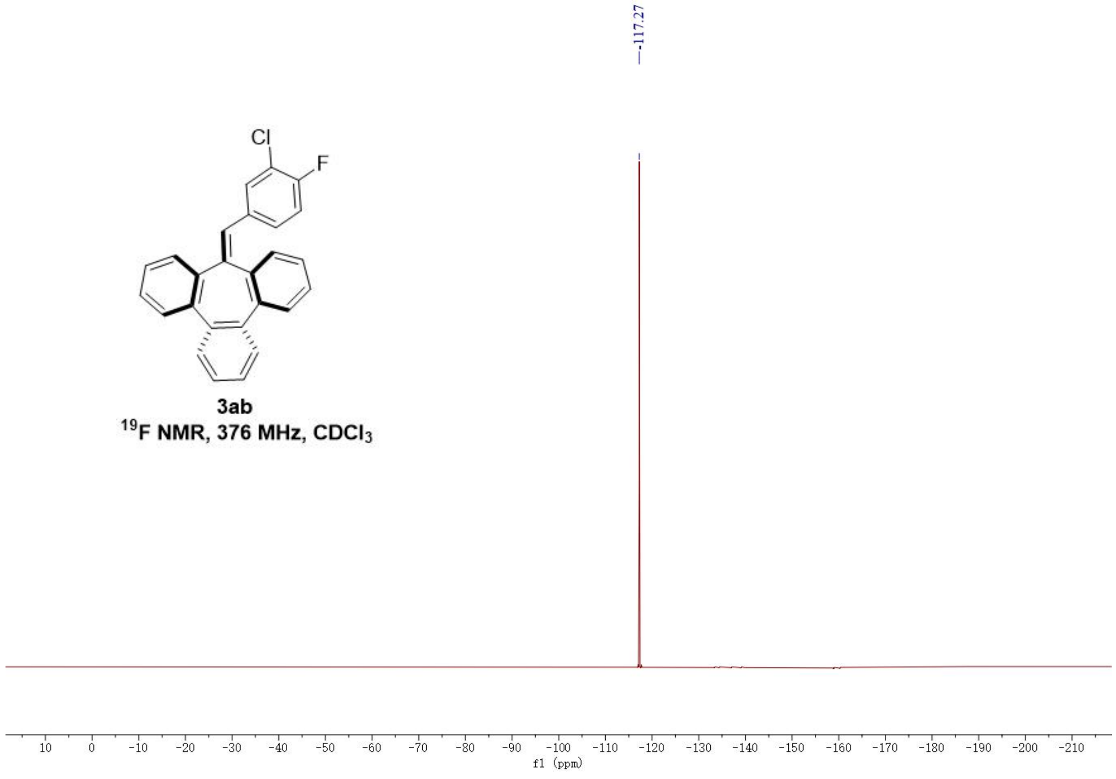

3ab

^{19}F NMR, 376 MHz, $CDCl_3$

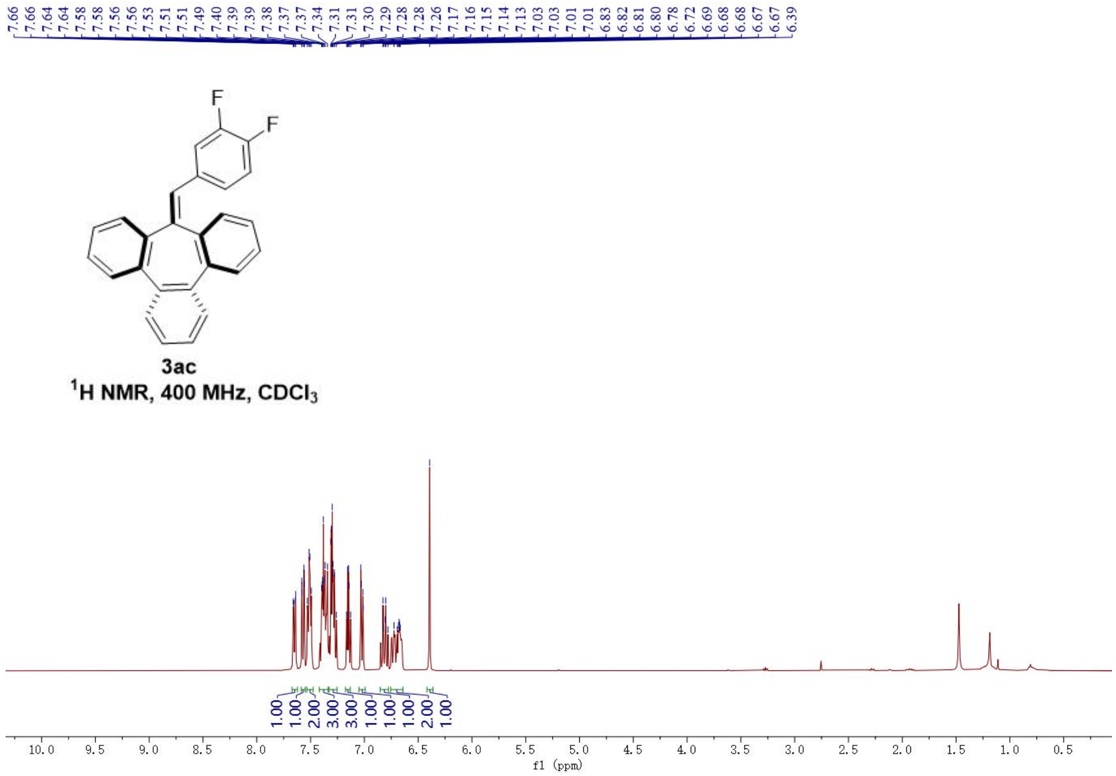

3ac
^{1}H NMR, 400 MHz, $CDCl_3$

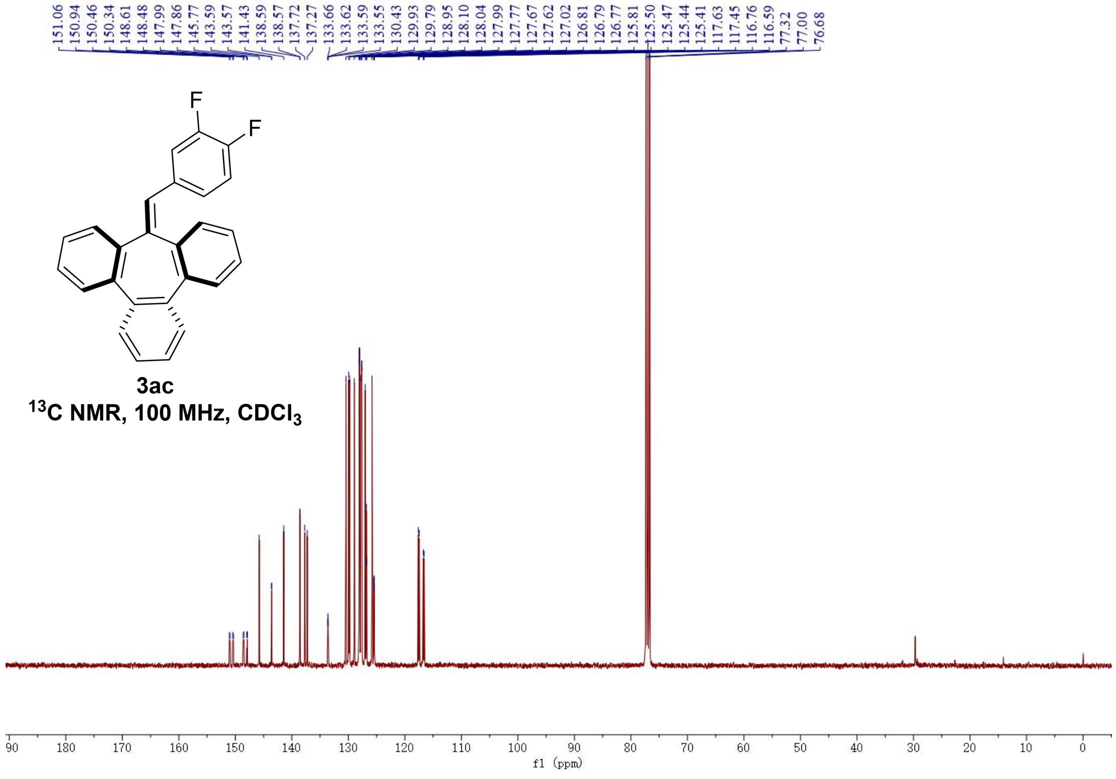

3ac
^{13}C NMR, 100 MHz, $CDCl_3$

3ac

^{19}F NMR, 565 MHz, $CDCl_3$

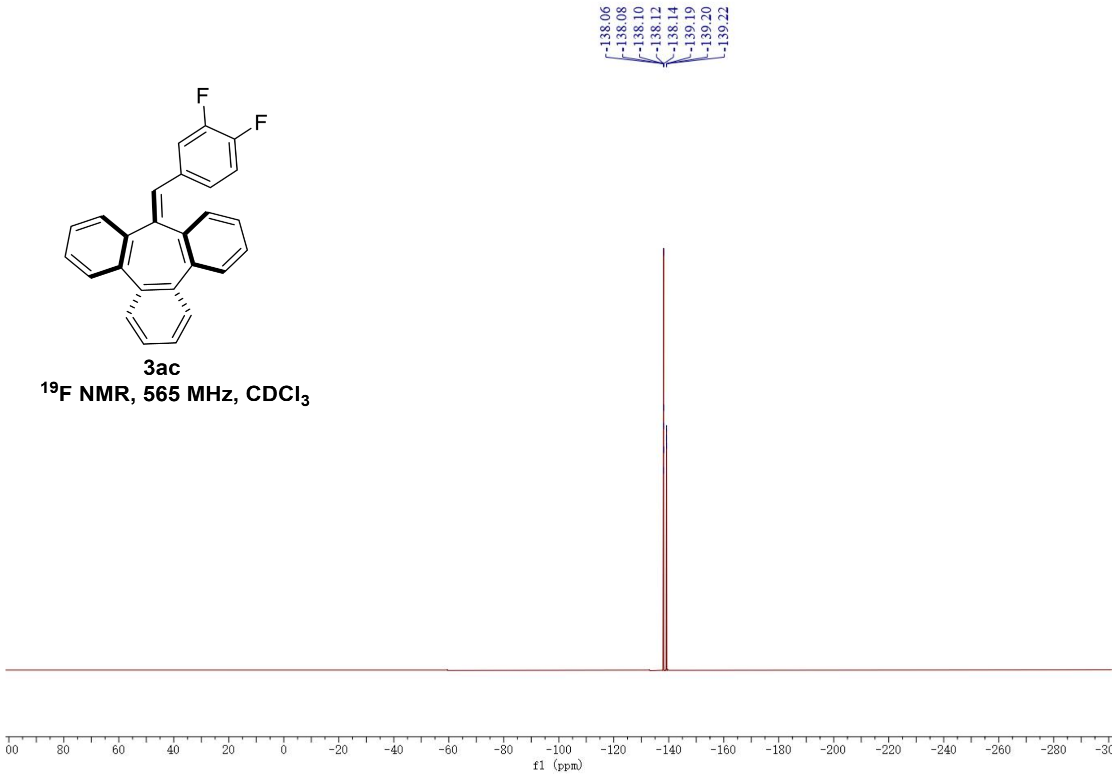

3ad

^{1}H NMR, 400 MHz, $CDCl_3$

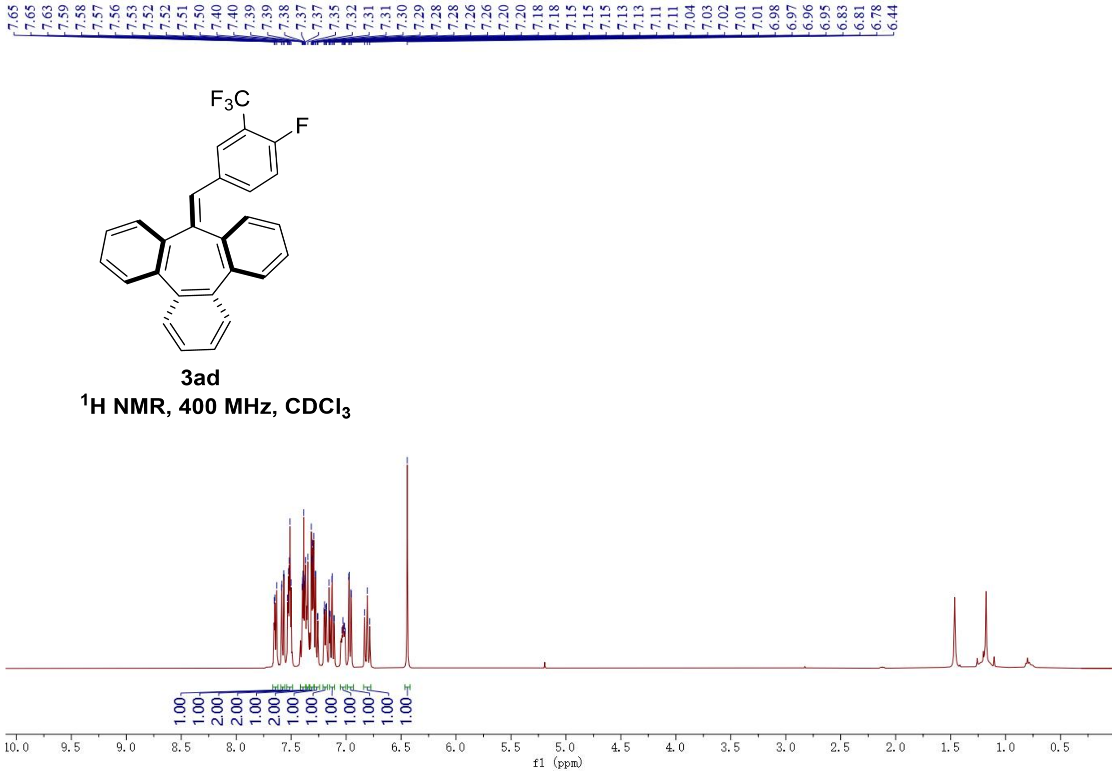

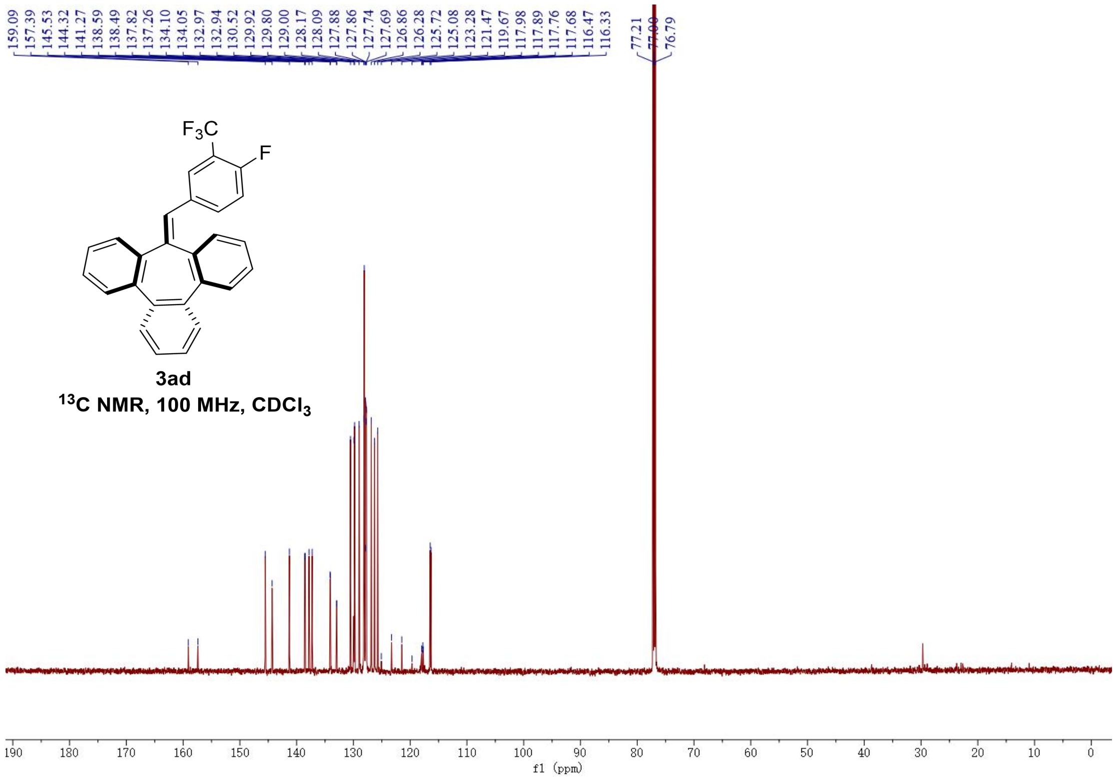
159.09
157.39
145.53
144.32
141.27
138.59
138.49
137.82
137.26
134.10
134.05
132.97
132.94
130.52
129.92
129.80
129.00
128.17
128.09
127.88
127.86
127.74
127.69
126.86
126.28
125.72
125.08
123.28
121.47
119.67
117.98
117.89
117.76
117.68
116.47
116.33
77.21
77.00
76.79
F_3C
F
3ad
^{13}C NMR, 100 MHz, $CDCl_3$
190 180 170 160 150 140 130 120 110 100 90 80 70 60 50 40 30 20 10 0
f1 (ppm)

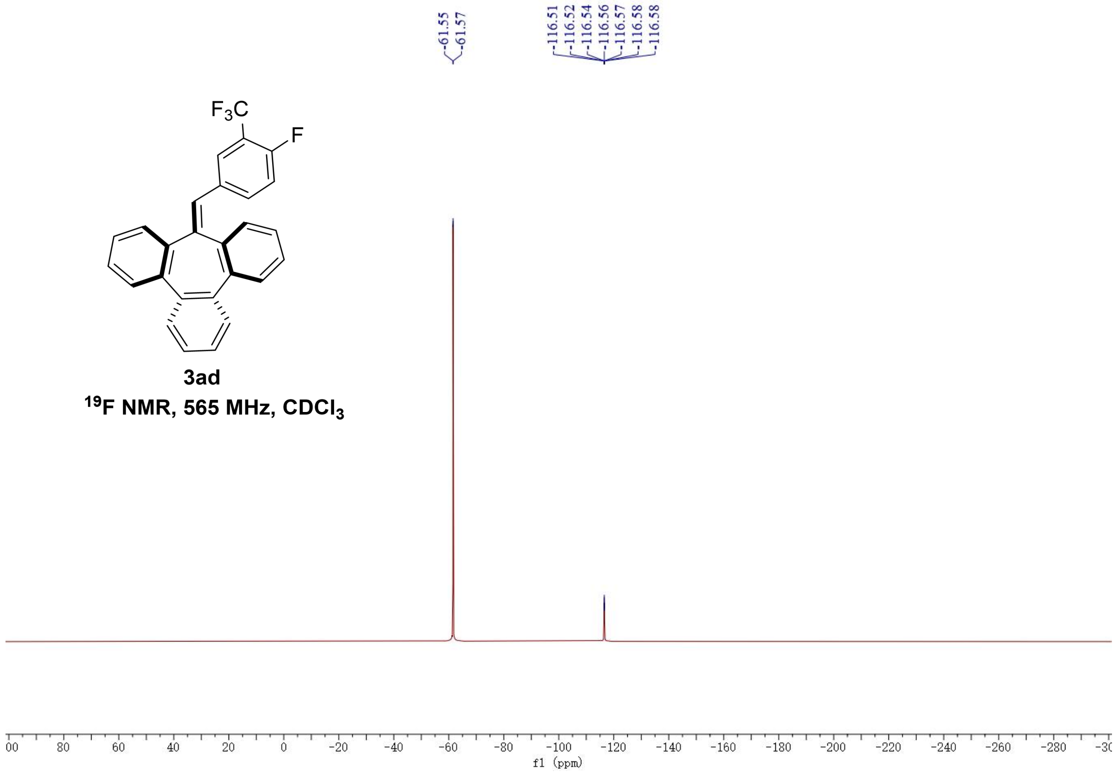
-61.55
-61.57
-116.51
-116.52
-116.54
-116.56
-116.57
-116.58
-116.58
F_3C
F
3ad
^{19}F NMR, 565 MHz, $CDCl_3$
00 80 60 40 20 0 -20 -40 -60 -80 -100 -120 -140 -160 -180 -200 -220 -240 -260 -280 -30
f1 (ppm)

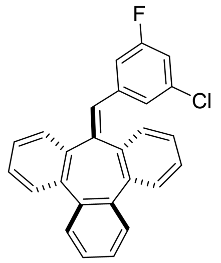

3ae

^{1}H NMR, 400 MHz, $CDCl_3$

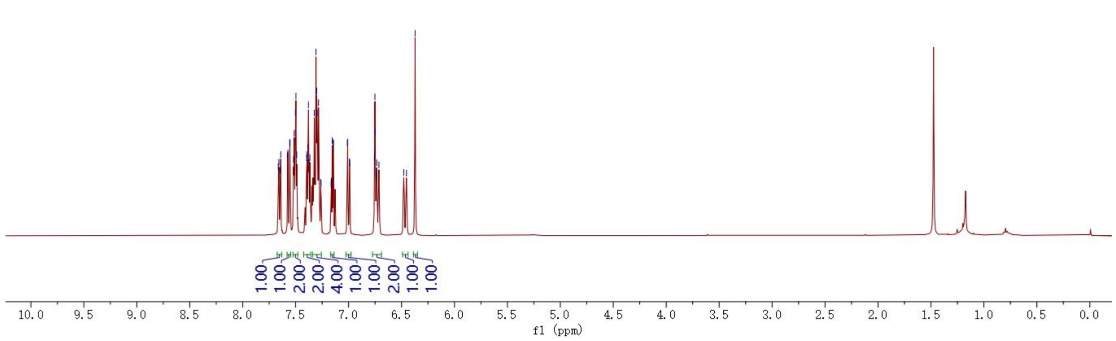

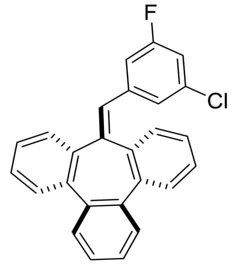

3ae

^{13}C NMR, 100 MHz, $CDCl_3$

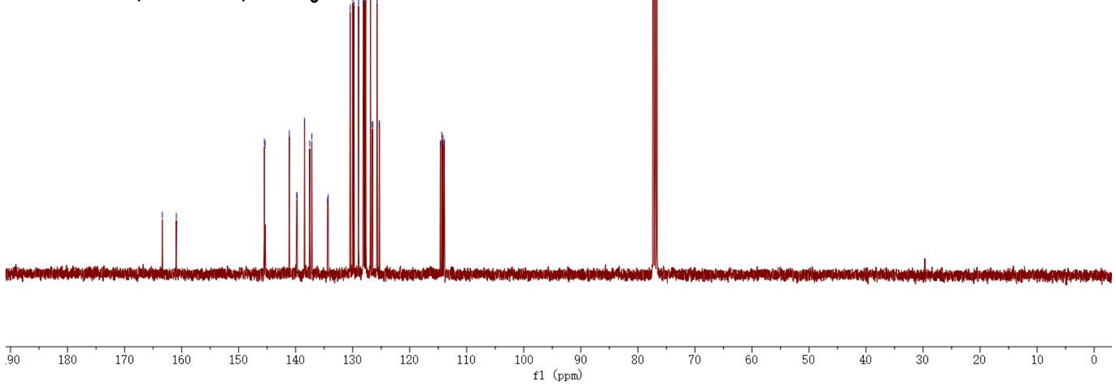

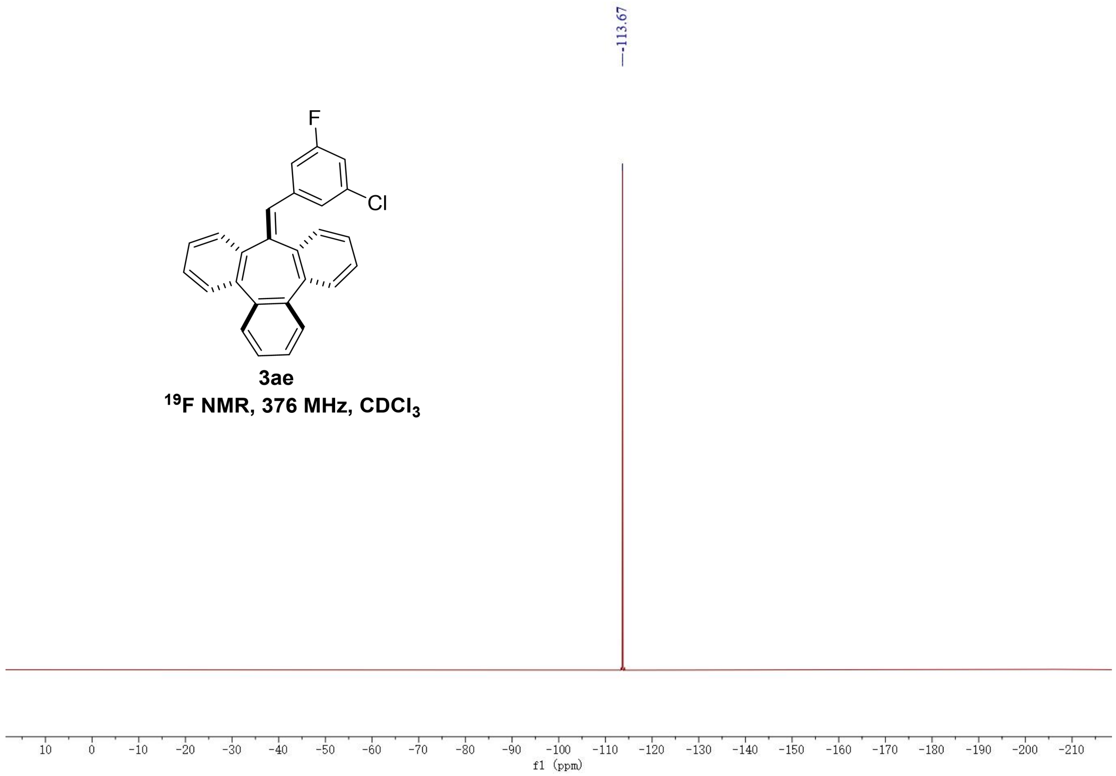

-113.67
F
Cl
3ae
^{19}F NMR, 376 MHz, CDCl$_3$
10 0 -10 -20 -30 -40 -50 -60 -70 -80 -90 -100 -110 -120 -130 -140 -150 -160 -170 -180 -190 -200 -210
f1 (ppm)

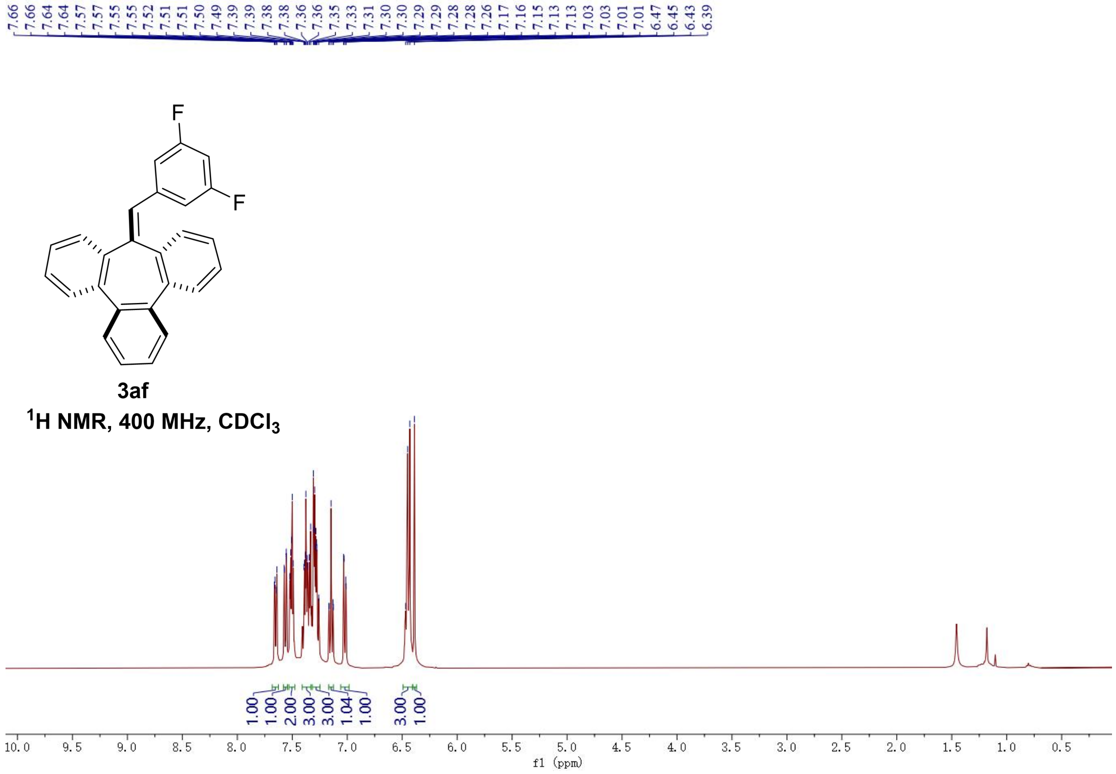

7.66 7.64 7.64 7.57 7.57 7.55 7.55 7.52 7.51 7.51 7.50 7.49 7.39 7.39 7.38 7.36 7.36 7.35 7.33 7.31 7.30 7.30 7.29 7.29 7.28 7.28 7.26 7.17 7.16 7.15 7.13 7.13 7.03 7.03 7.01 7.01 6.47 6.45 6.43 6.39
F
F
3af
^{1}H NMR, 400 MHz, CDCl$_3$
1.00 1.00 2.00 3.00 3.00 1.04 1.00 3.00 1.00
10.0 9.5 9.0 8.5 8.0 7.5 7.0 6.5 6.0 5.5 5.0 4.5 4.0 3.5 3.0 2.5 2.0 1.5 1.0 0.5
f1 (ppm)

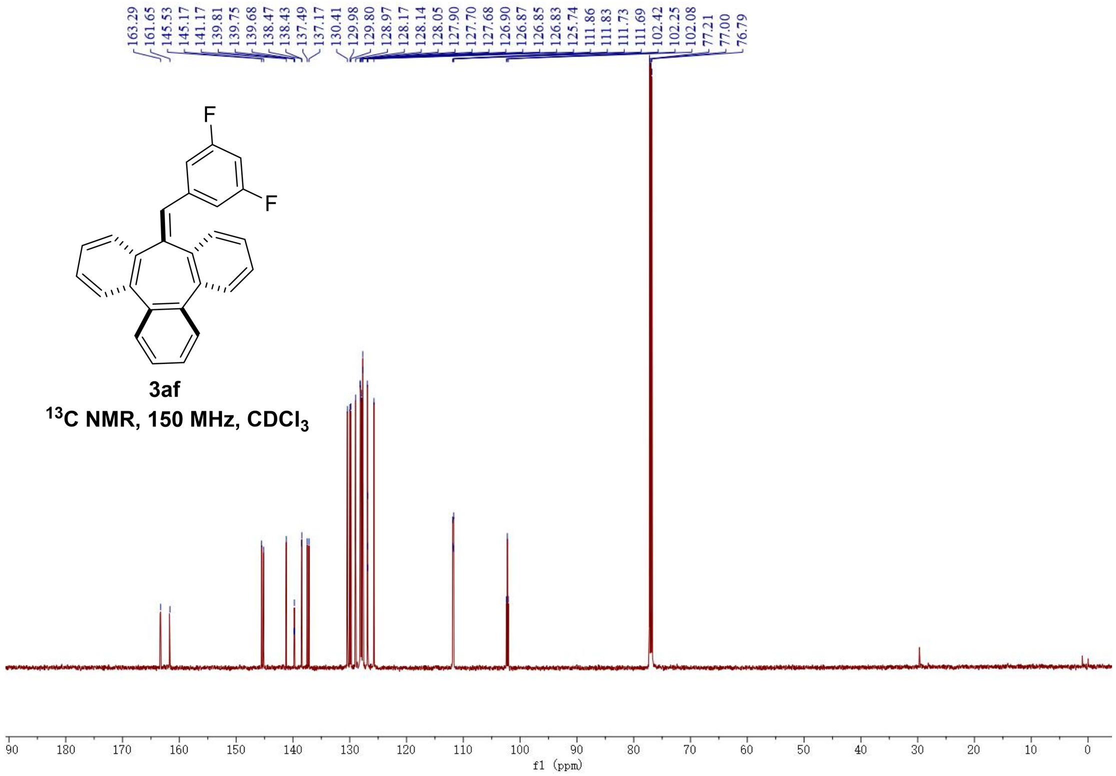
163.29
161.65
145.53
145.17
141.17
139.81
139.75
139.68
138.47
138.43
137.49
137.17
130.41
129.98
129.80
128.97
128.17
128.14
128.05
127.90
127.70
127.68
126.90
126.87
126.85
126.83
125.74
111.86
111.83
111.73
111.69
102.42
102.25
102.08
77.21
77.00
76.79
F
F
3af
^{13}C NMR, 150 MHz, CDCl$_3$
f1 (ppm)

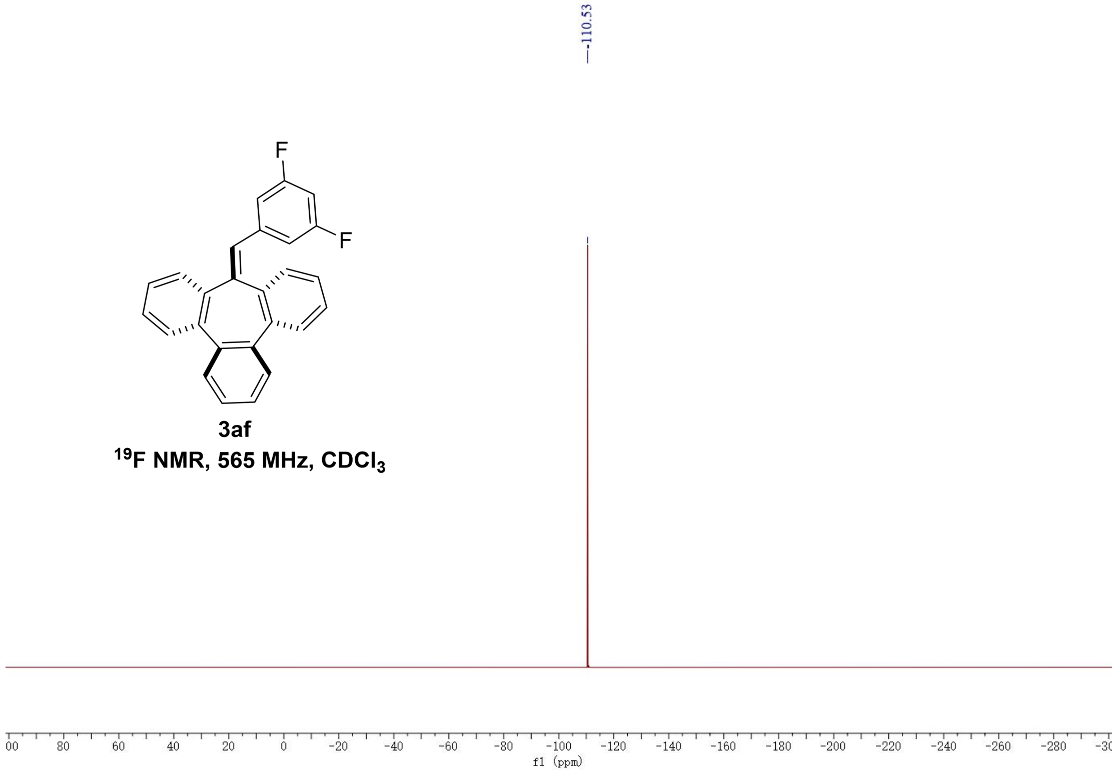
-110.53
F
F
3af
^{19}F NMR, 565 MHz, CDCl$_3$
f1 (ppm)

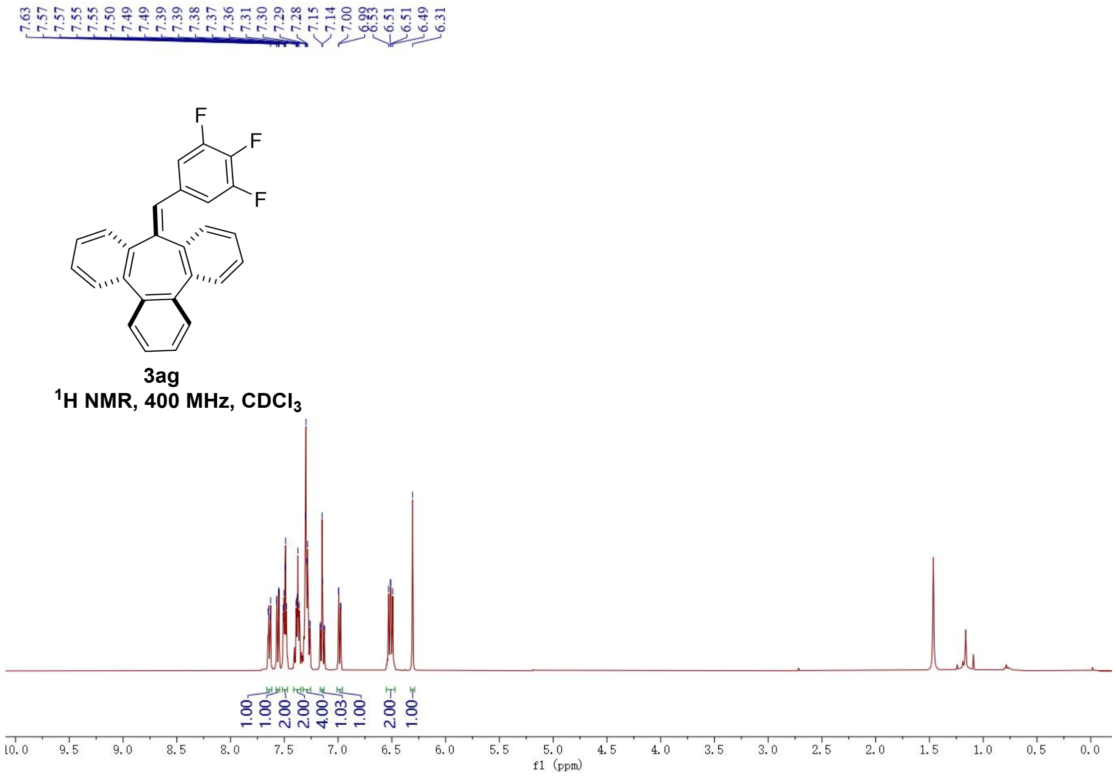
7.63 7.57 7.57 7.55 7.55 7.50 7.49 7.49 7.39 7.39 7.38 7.37 7.36 7.31 7.30 7.29 7.28 7.15 7.14 7.00 6.99 6.53 6.51 6.51 6.49 6.31
F
F
F
3ag
^{1}H NMR, 400 MHz, CDCl$_3$
1.00 1.00 2.00 2.00 4.00 1.03 1.00 2.00 1.00
10.0 9.5 9.0 8.5 8.0 7.5 7.0 6.5 6.0 5.5 5.0 4.5 4.0 3.5 3.0 2.5 2.0 1.5 1.0 0.5 0.0
f1 (ppm)

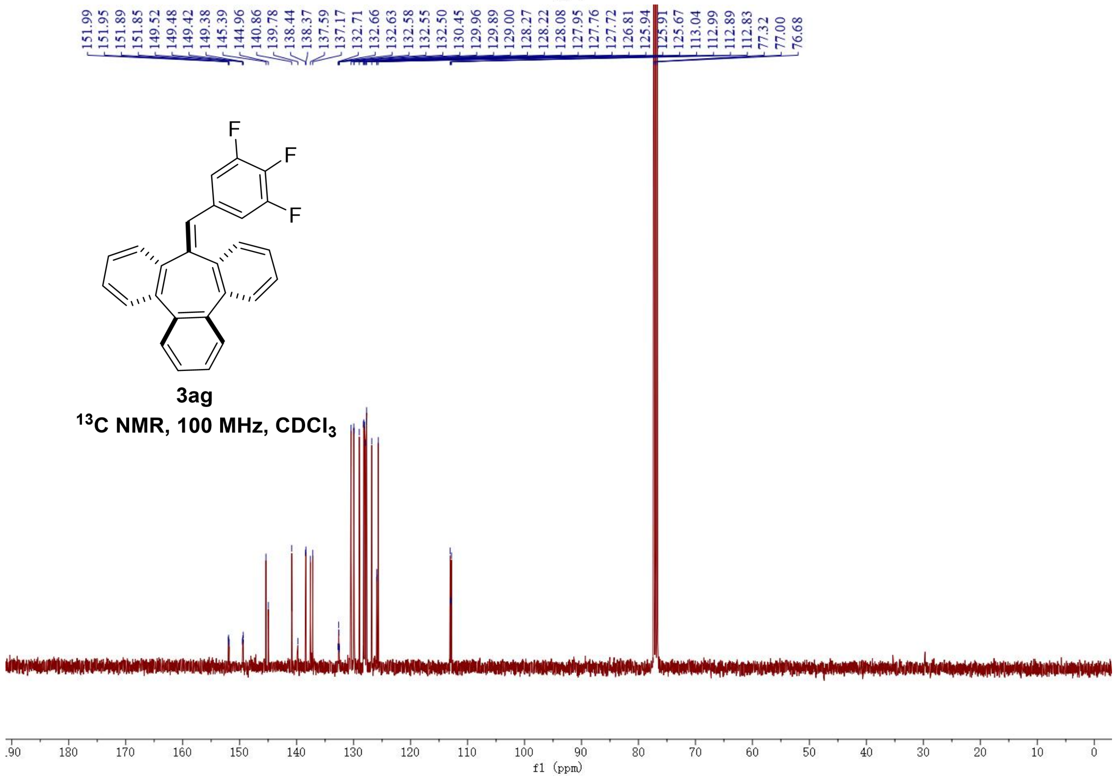
151.99 151.95 151.89 151.85 149.52 149.48 149.42 149.38 145.39 144.96 140.86 139.78 138.44 138.37 137.59 137.17 132.71 132.66 132.63 132.58 132.55 132.50 130.45 129.96 129.89 129.00 128.27 128.22 128.08 127.95 127.76 127.72 126.81 125.94 125.91 125.67 113.04 112.99 112.89 112.83 77.32 77.00 76.68
F
F
F
3ag
^{13}C NMR, 100 MHz, CDCl$_3$
190 180 170 160 150 140 130 120 110 100 90 80 70 60 50 40 30 20 10 0
f1 (ppm)

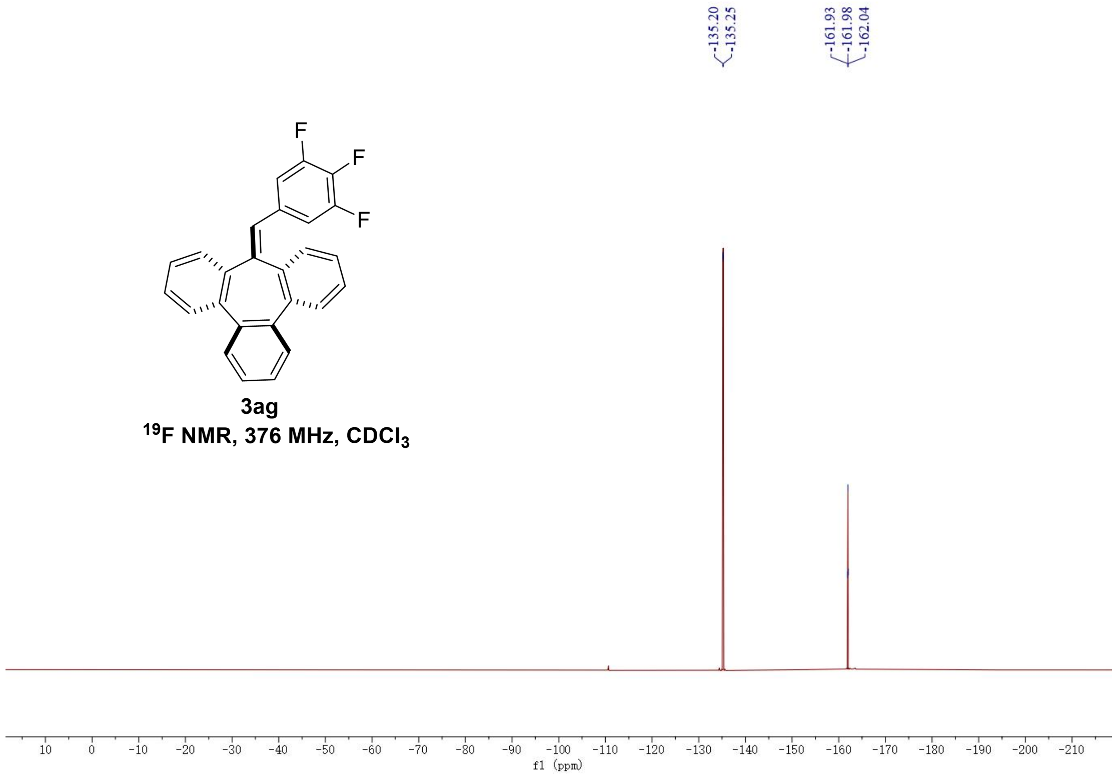

3ag
^{19}F NMR, 376 MHz, CDCl$_3$

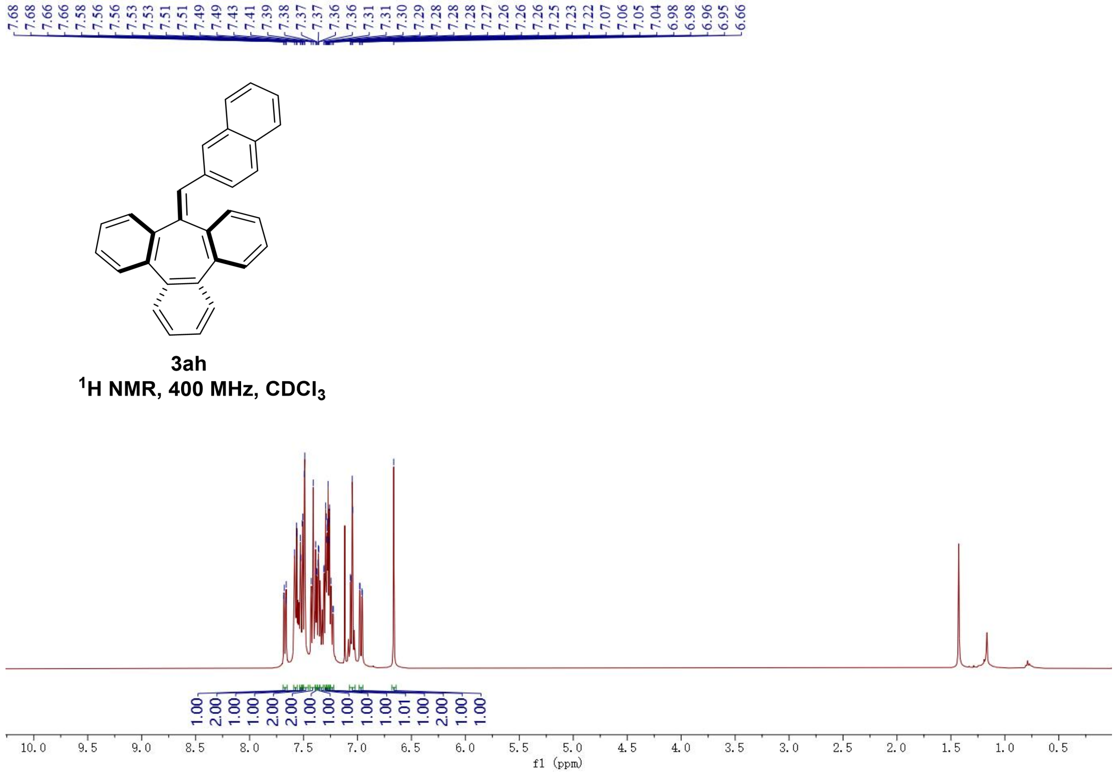

3ah
^{1}H NMR, 400 MHz, CDCl$_3$

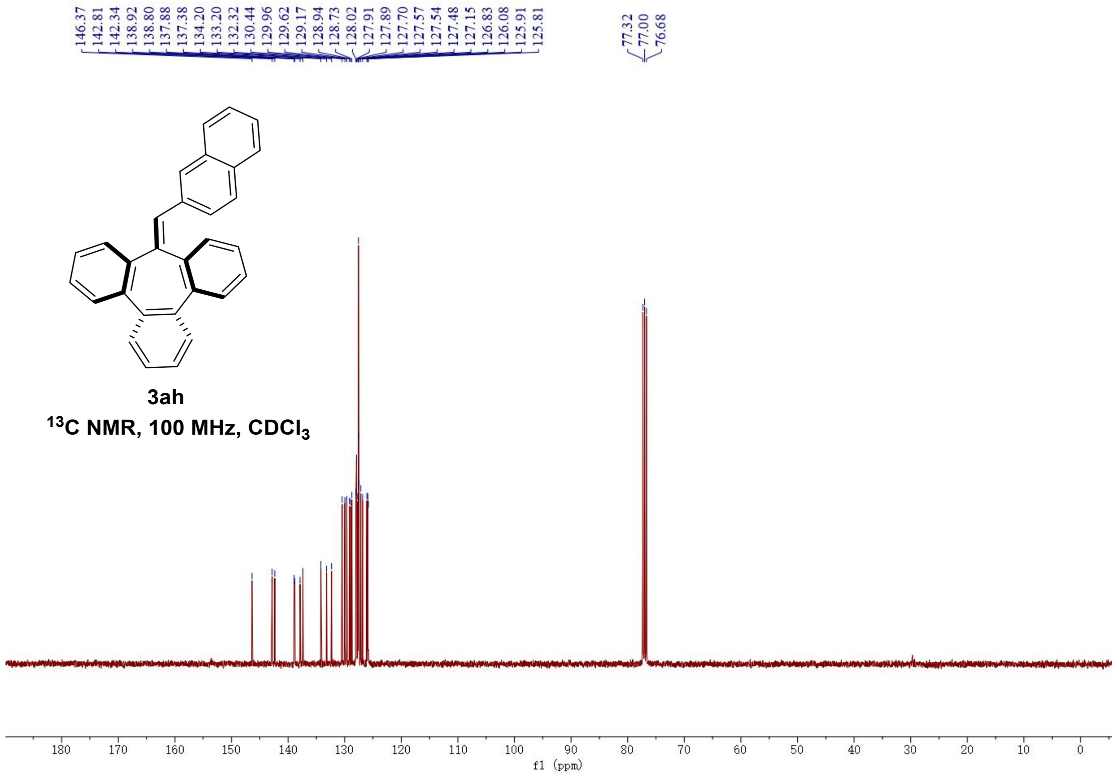
146.37
142.81
142.34
138.92
138.80
137.88
137.38
134.20
133.20
132.32
130.44
129.96
129.62
129.17
128.94
128.73
128.02
127.91
127.89
127.70
127.57
127.54
127.48
127.15
126.83
126.08
125.91
125.81
77.32
77.00
76.68
3ah
^{13}C NMR, 100 MHz, CDCl$_3$
f1 (ppm)

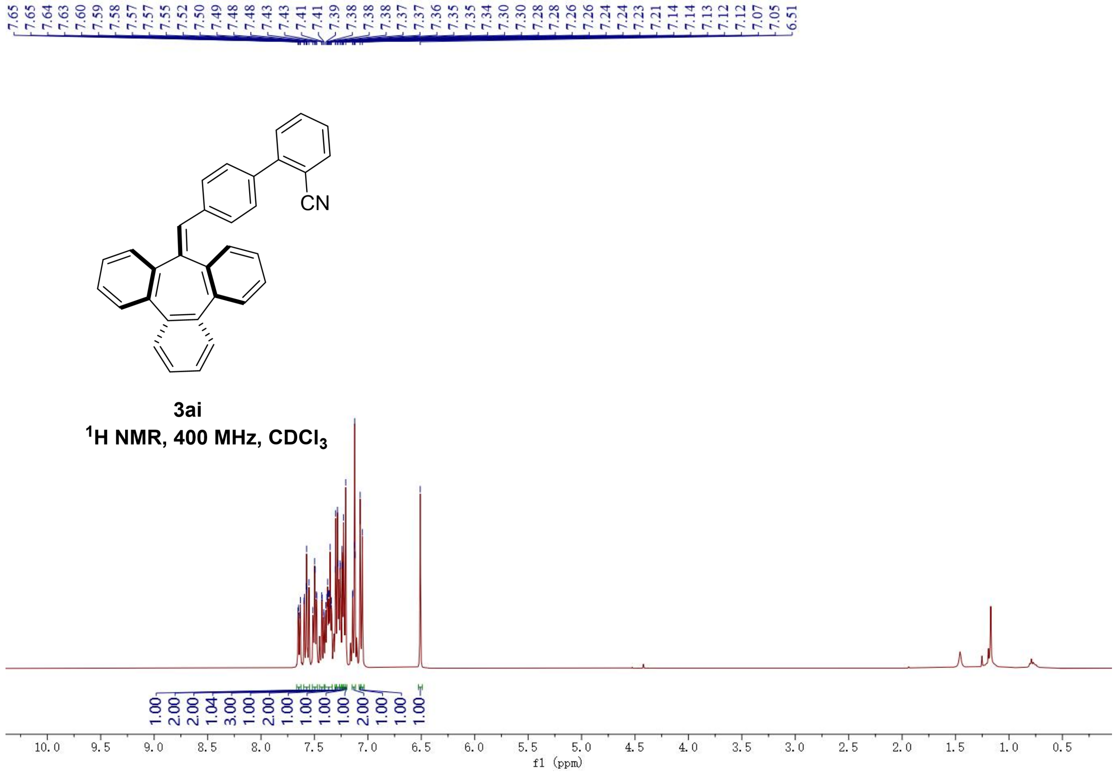
7.65
7.64
7.63
7.60
7.59
7.58
7.57
7.57
7.55
7.52
7.50
7.49
7.48
7.43
7.43
7.41
7.41
7.39
7.38
7.38
7.37
7.37
7.36
7.35
7.35
7.34
7.30
7.30
7.28
7.28
7.26
7.26
7.24
7.24
7.23
7.21
7.14
7.14
7.13
7.12
7.12
7.07
7.05
6.51
CN
3ai
^{1}H NMR, 400 MHz, CDCl$_3$
1.00
2.00
2.00
1.04
3.00
2.00
1.00
1.00
1.00
1.00
2.00
1.00
1.00
1.00
f1 (ppm)

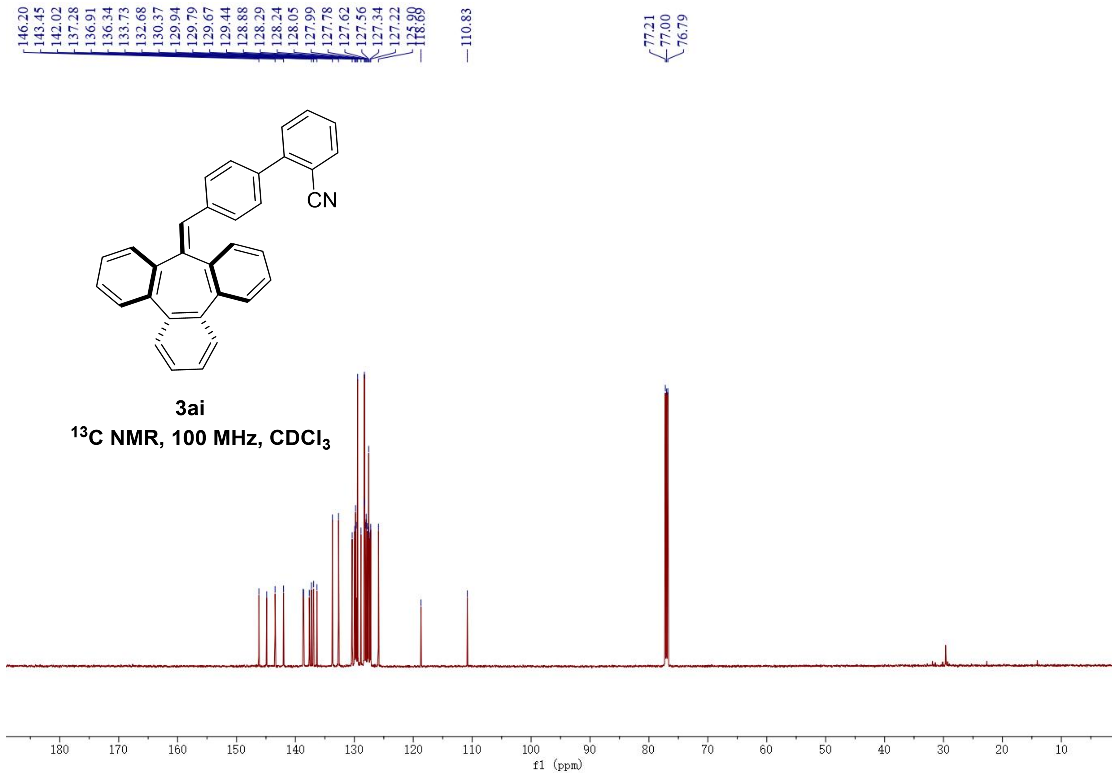
146.20
143.45
142.02
137.28
136.91
136.34
133.73
132.68
130.37
129.94
129.79
129.67
129.44
128.88
128.29
128.24
128.05
127.99
127.78
127.62
127.56
127.34
127.22
125.90
118.69
110.83
77.21
77.00
76.79
CN
3ai
^{13}C NMR, 100 MHz, CDCl$_3$
180 170 160 150 140 130 120 110 100 90 80 70 60 50 40 30 20 10
f1 (ppm)

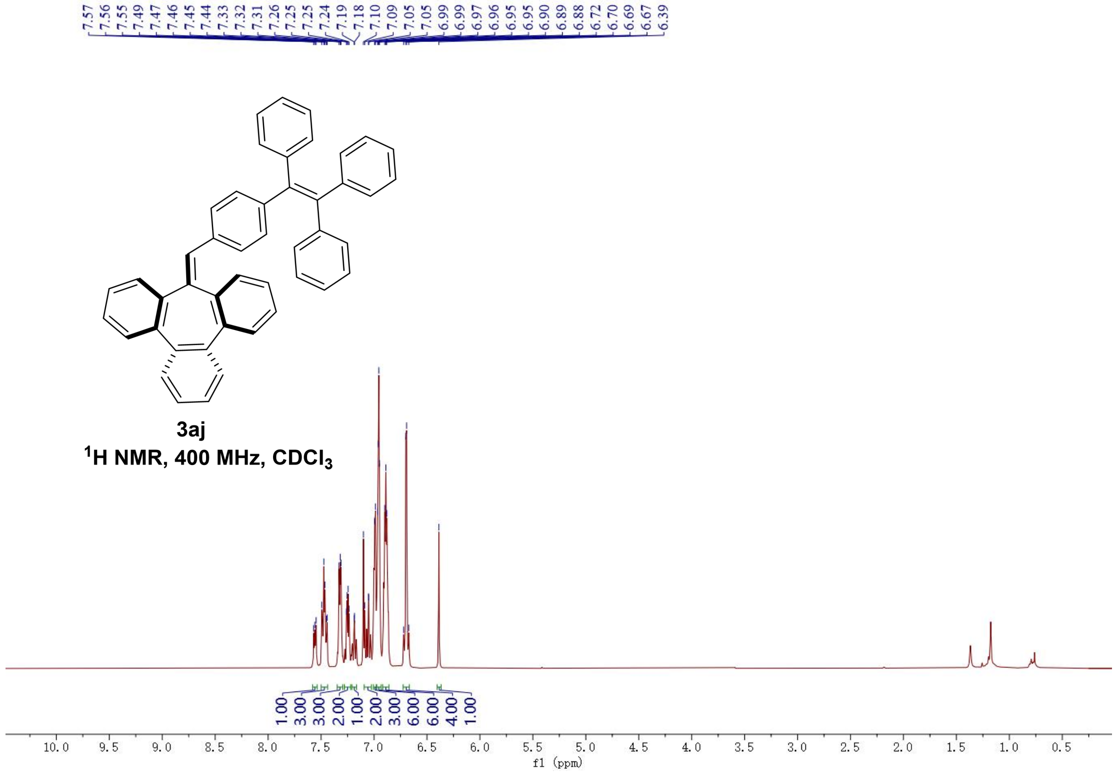
7.57
7.56
7.55
7.49
7.47
7.46
7.45
7.44
7.33
7.32
7.31
7.26
7.25
7.25
7.24
7.19
7.18
7.10
7.09
7.05
7.05
6.99
6.99
6.97
6.96
6.95
6.95
6.90
6.89
6.88
6.72
6.70
6.69
6.67
6.39
3aj
^{1}H NMR, 400 MHz, CDCl$_3$
1.00
3.00
3.00
2.00
1.00
2.00
3.00
6.00
6.00
4.00
1.00
10.0 9.5 9.0 8.5 8.0 7.5 7.0 6.5 6.0 5.5 5.0 4.5 4.0 3.5 3.0 2.5 2.0 1.5 1.0 0.5
f1 (ppm)

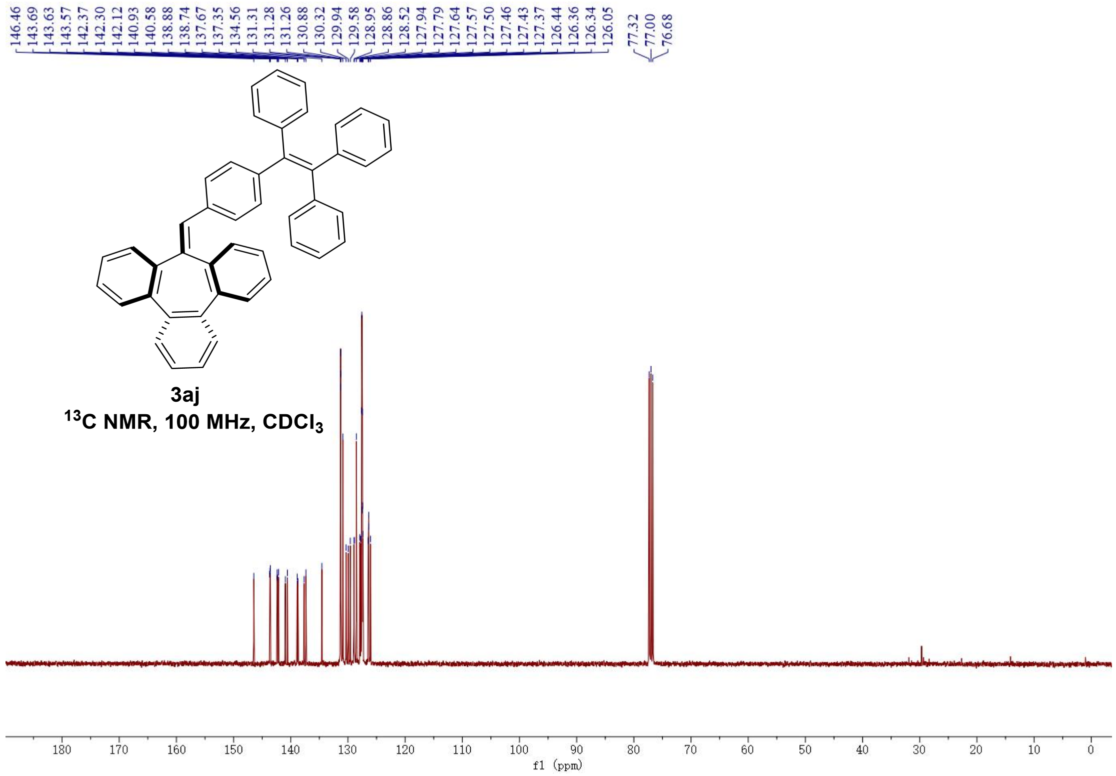
146.46
143.69
143.63
143.57
142.37
142.30
142.12
140.93
140.58
138.88
138.74
137.67
137.35
134.56
131.31
131.28
131.26
130.88
130.32
129.94
129.58
128.95
128.86
128.52
127.94
127.79
127.64
127.57
127.50
127.46
127.43
127.37
126.44
126.36
126.34
126.05
77.32
77.00
76.68
3aj
13C NMR, 100 MHz, CDCl3
f1 (ppm)

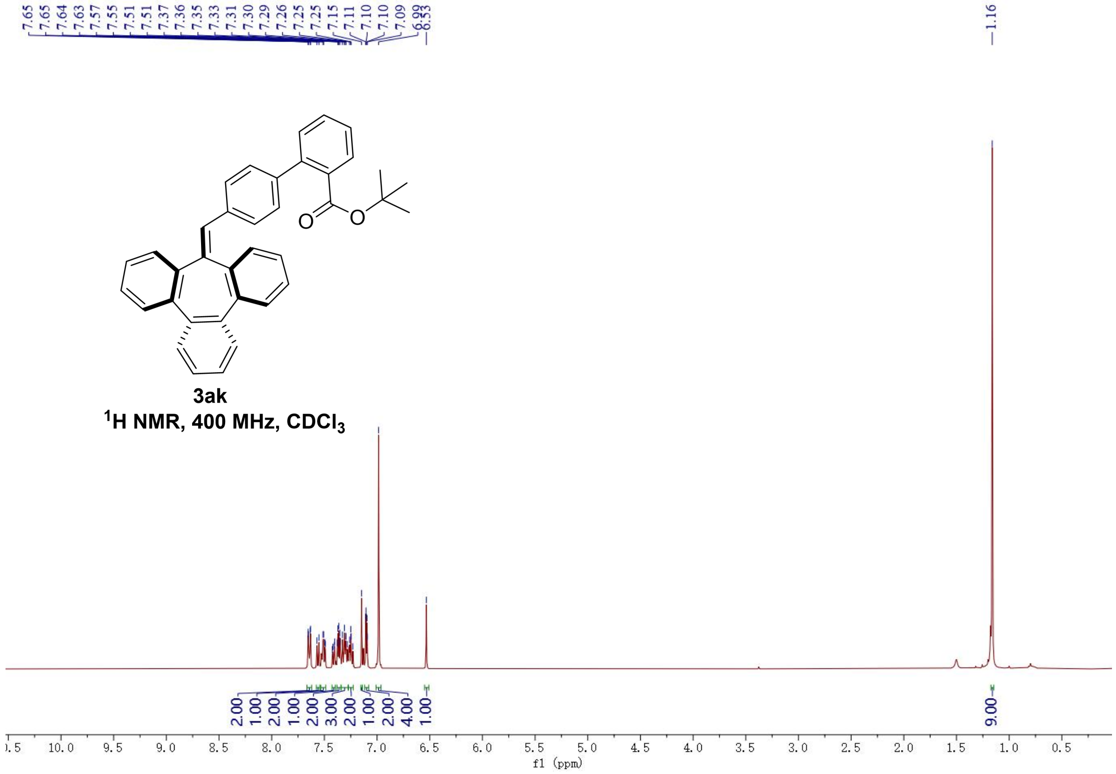
7.65
7.65
7.64
7.63
7.57
7.55
7.51
7.51
7.37
7.36
7.35
7.33
7.31
7.30
7.29
7.26
7.25
7.25
7.15
7.11
7.10
7.10
7.09
6.99
6.53
1.16
3ak
1H NMR, 400 MHz, CDCl3
2.00
1.00
2.00
1.00
2.00
3.00
2.00
1.00
2.00
4.00
1.00
9.00
f1 (ppm)

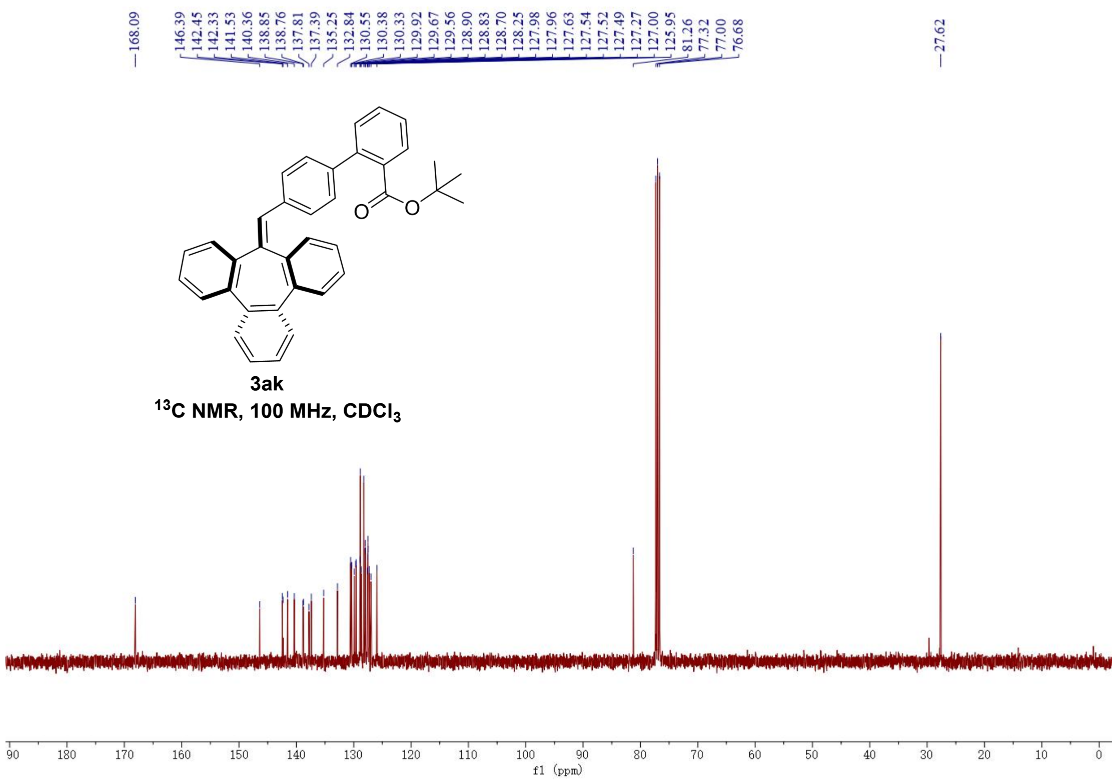

3ak

^{13}C NMR, 100 MHz, $CDCl_3$

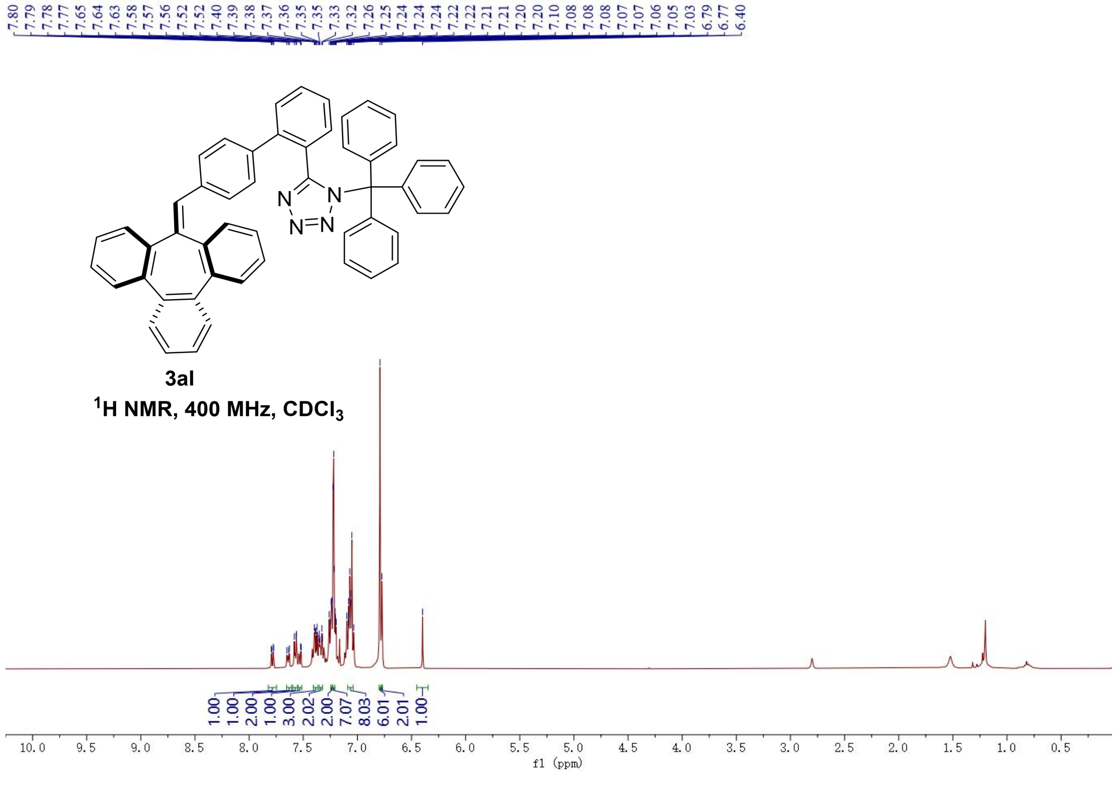

3al

^{1}H NMR, 400 MHz, $CDCl_3$

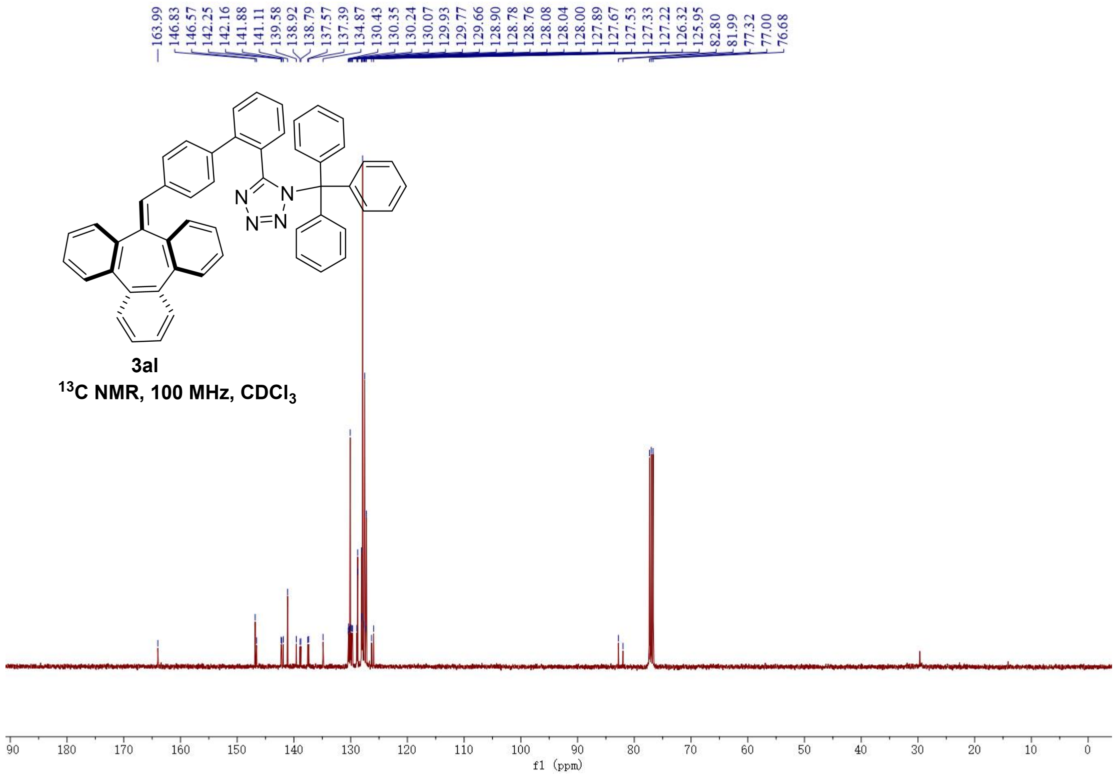
163.99
146.83
146.57
142.25
142.16
141.88
141.11
139.58
138.92
138.79
137.57
137.39
134.87
130.43
130.35
130.24
130.07
129.93
129.77
129.66
128.90
128.78
128.76
128.08
128.04
128.00
127.89
127.67
127.53
127.33
127.22
126.32
125.95
82.80
81.99
77.32
77.00
76.68
N
N
N
N
3al
^{13}C NMR, 100 MHz, CDCl$_3$
f1 (ppm)

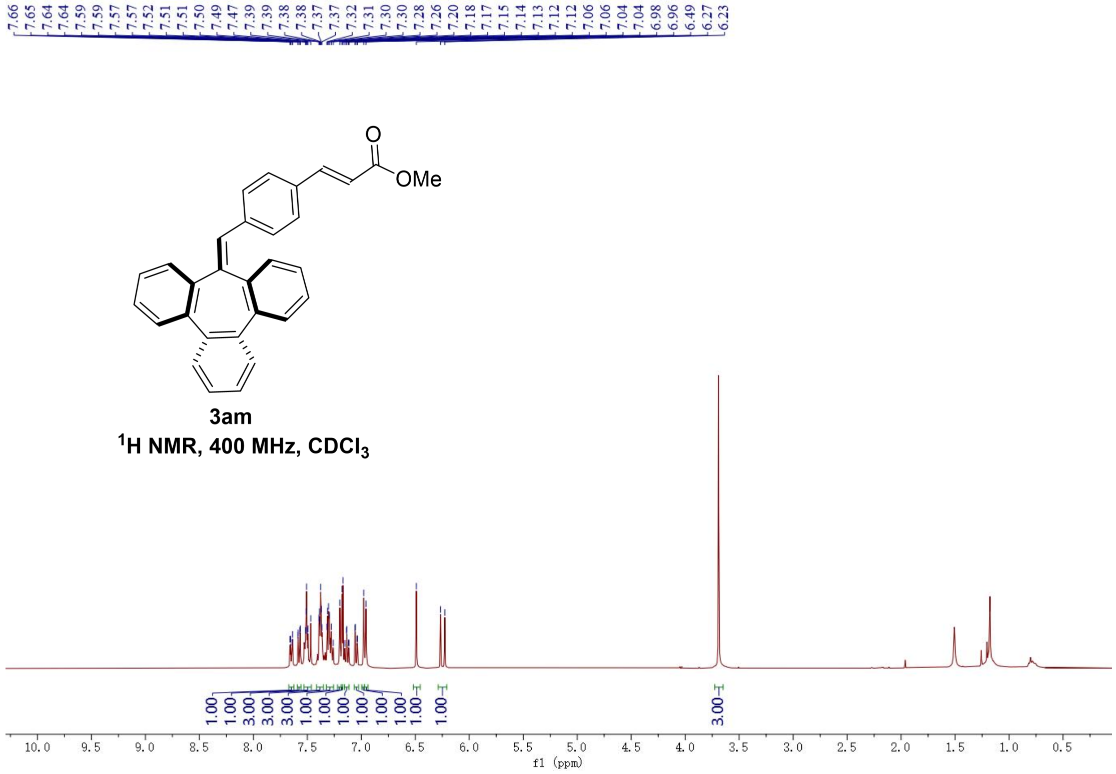
7.66
7.65
7.64
7.59
7.59
7.57
7.57
7.52
7.51
7.51
7.50
7.49
7.47
7.39
7.39
7.38
7.38
7.37
7.37
7.32
7.31
7.30
7.30
7.28
7.26
7.20
7.18
7.17
7.15
7.14
7.13
7.12
7.12
7.06
7.04
7.04
6.98
6.96
6.49
6.27
6.23
O
OMe
3am
^{1}H NMR, 400 MHz, CDCl$_3$
1.00
1.00
3.00
3.00
1.00
1.00
1.00
1.00
1.00
1.00
1.00
3.00
f1 (ppm)

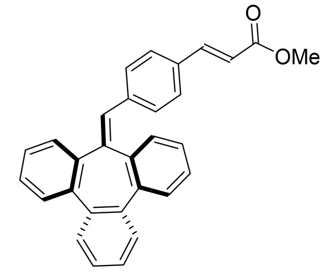

3am

^{13}C NMR, 150 MHz, $CDCl_3$

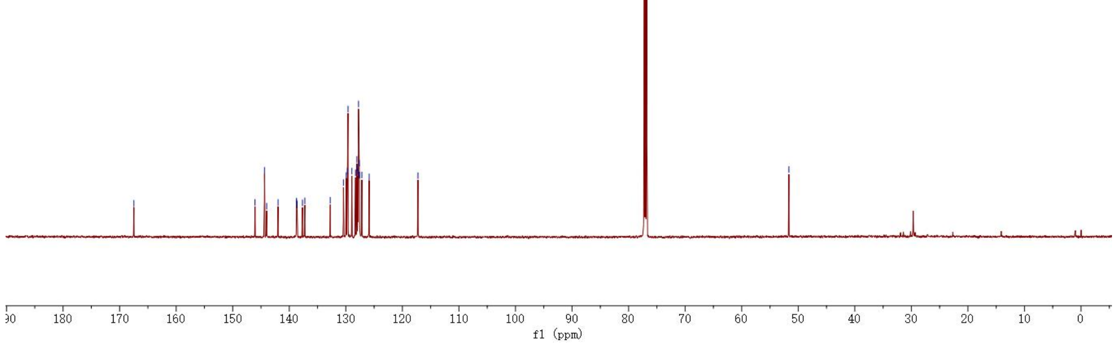

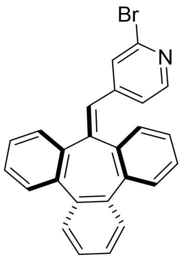

3an

^{1}H NMR, 400 MHz, $CDCl_3$

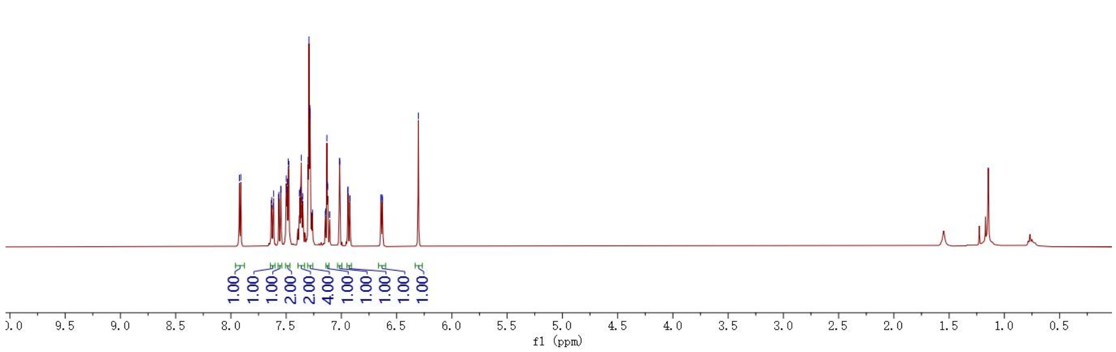

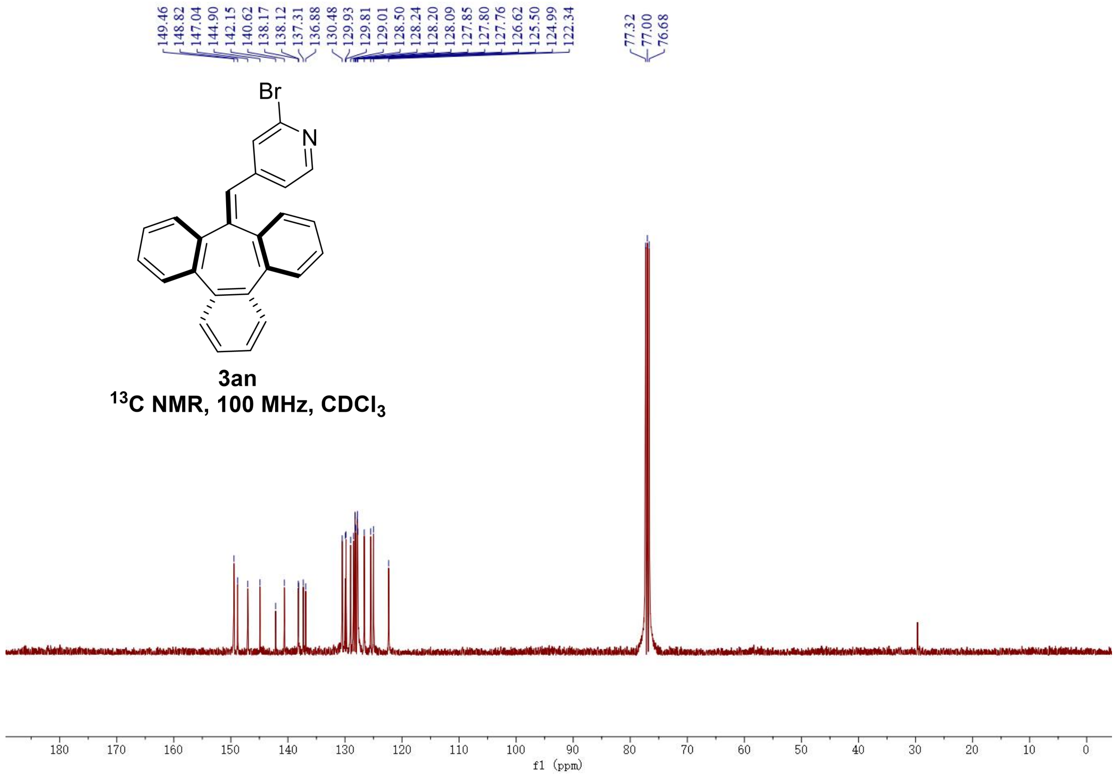

3an
^{13}C NMR, 100 MHz, $CDCl_3$

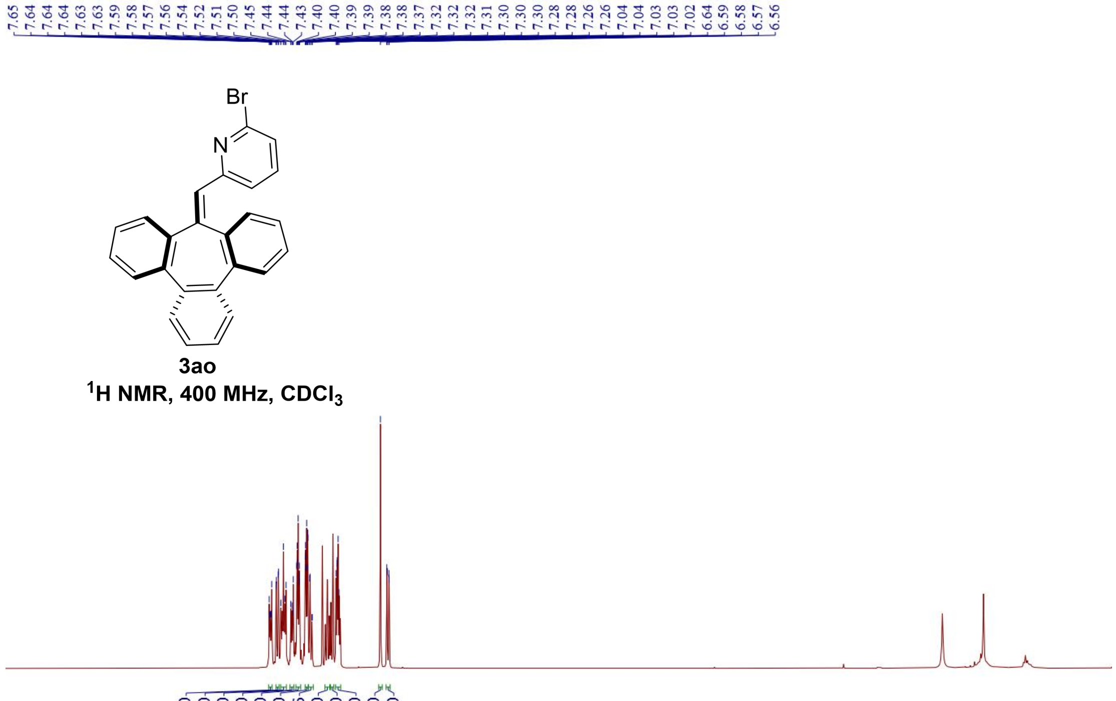

3ao
^{1}H NMR, 400 MHz, $CDCl_3$

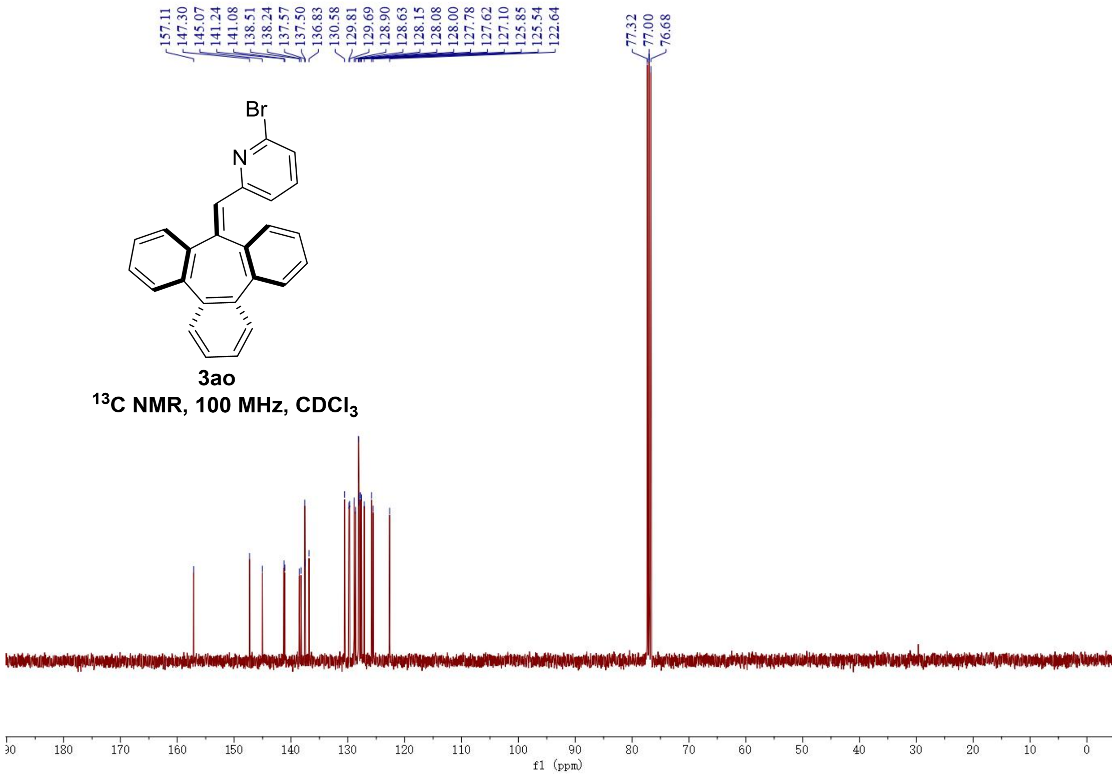
157.11
147.30
145.07
141.24
141.08
138.51
138.24
137.57
137.50
136.83
130.58
129.81
129.69
128.90
128.63
128.15
128.08
128.00
127.78
127.62
127.10
125.85
125.54
122.64
77.32
77.00
76.68
Br
N
3ao
13C NMR, 100 MHz, CDCl3
f1 (ppm)

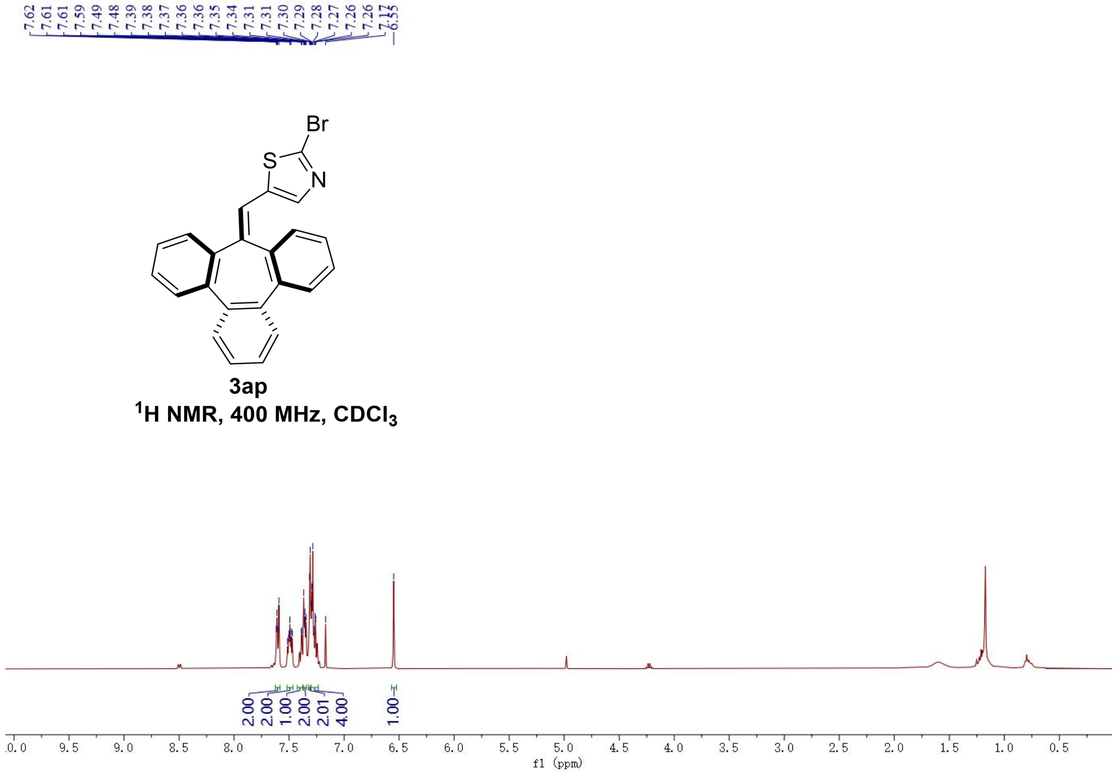
7.62
7.61
7.59
7.49
7.48
7.39
7.38
7.37
7.36
7.35
7.34
7.31
7.30
7.29
7.28
7.27
7.26
7.26
7.17
6.55
Br
S
N
3ap
1H NMR, 400 MHz, CDCl3
2.00
2.00
1.00
2.00
2.01
4.00
1.00
f1 (ppm)

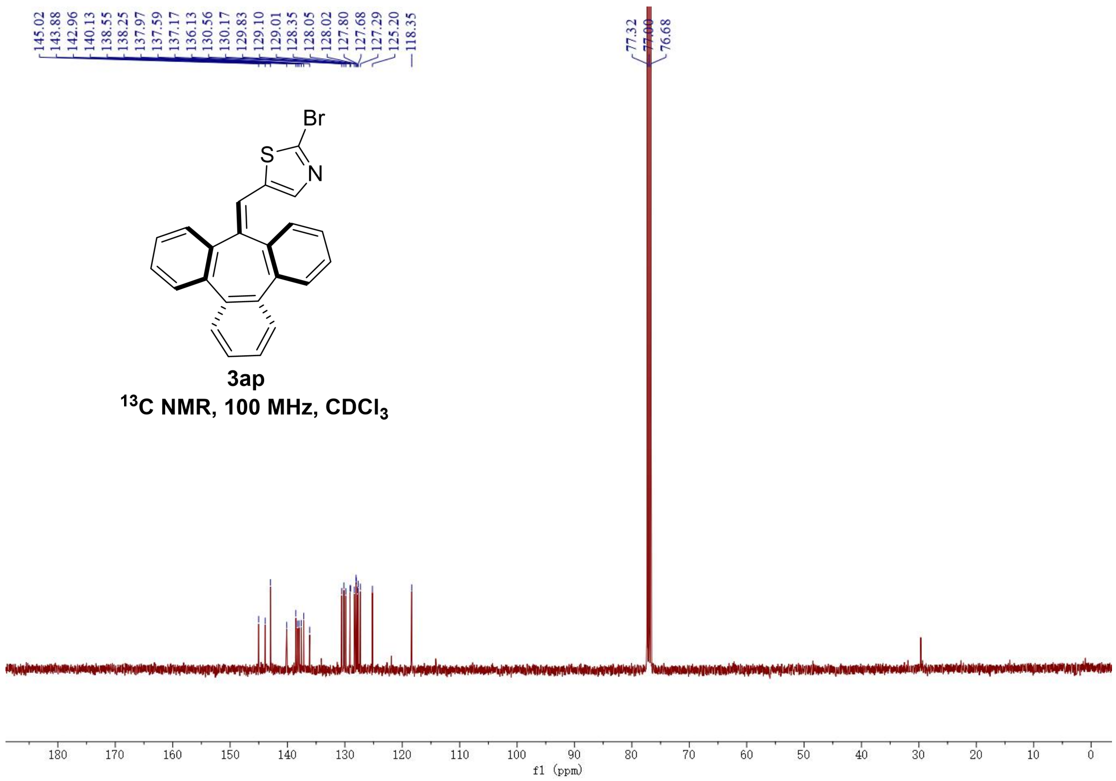

3ap
^{13}C NMR, 100 MHz, CDCl$_3$

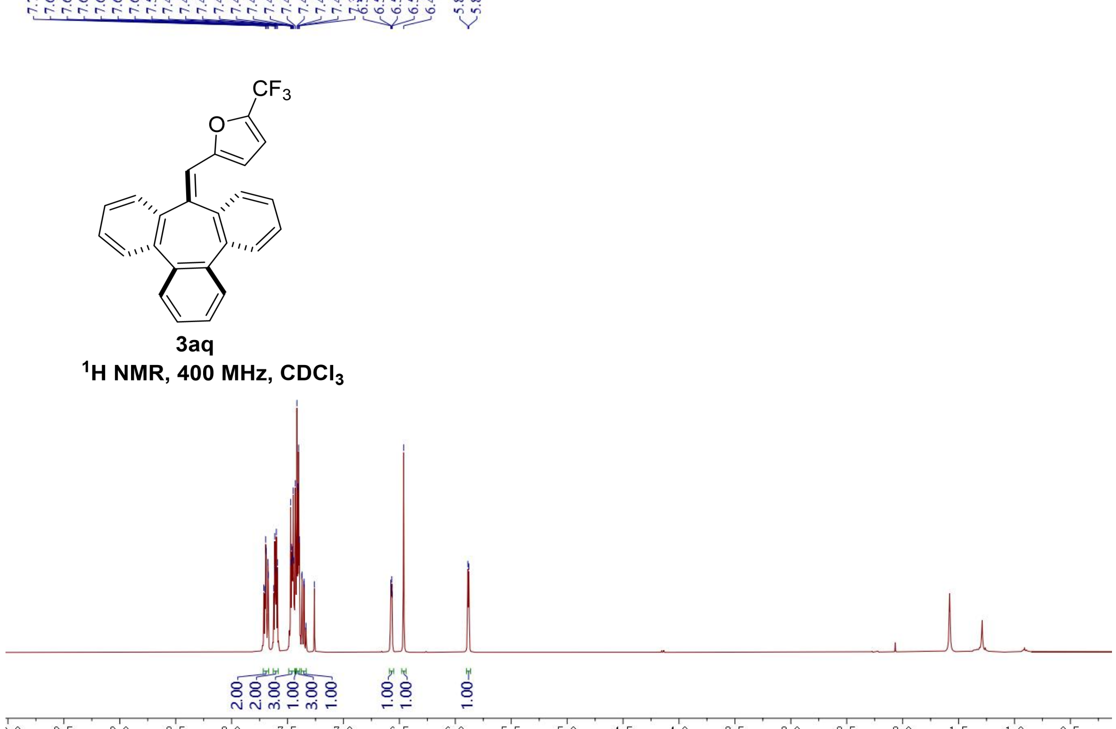

3aq
^{1}H NMR, 400 MHz, CDCl$_3$

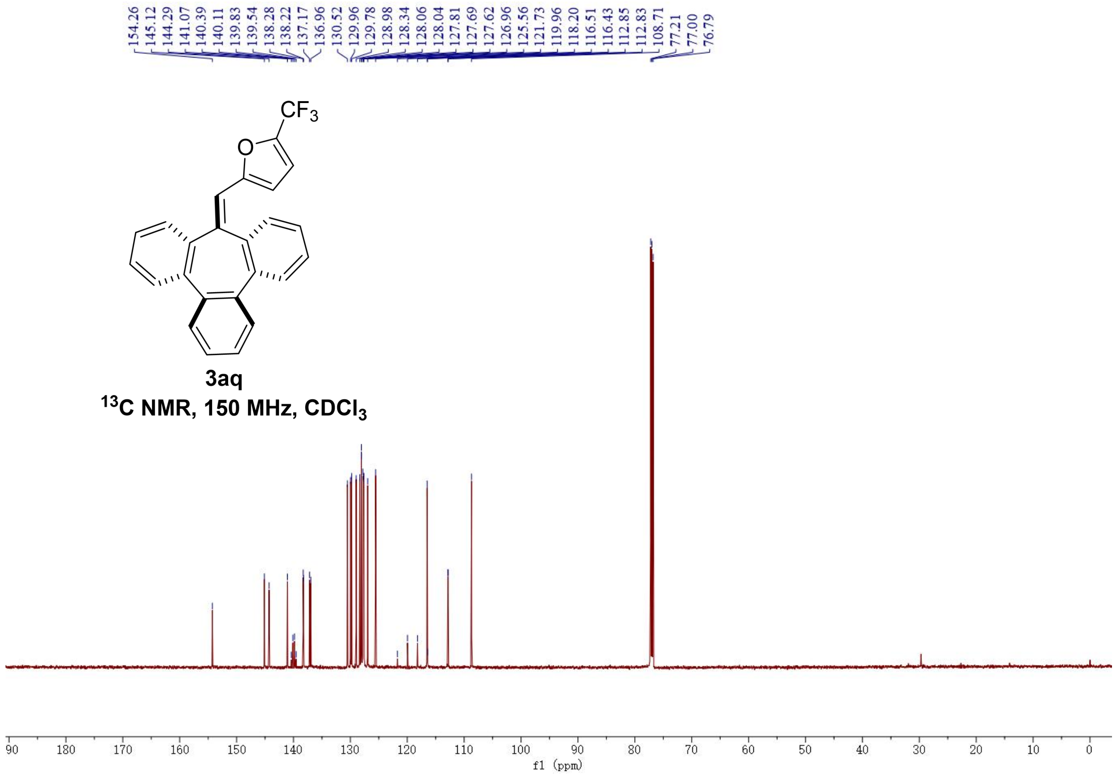

3aq

^{13}C NMR, 150 MHz, CDCl$_3$

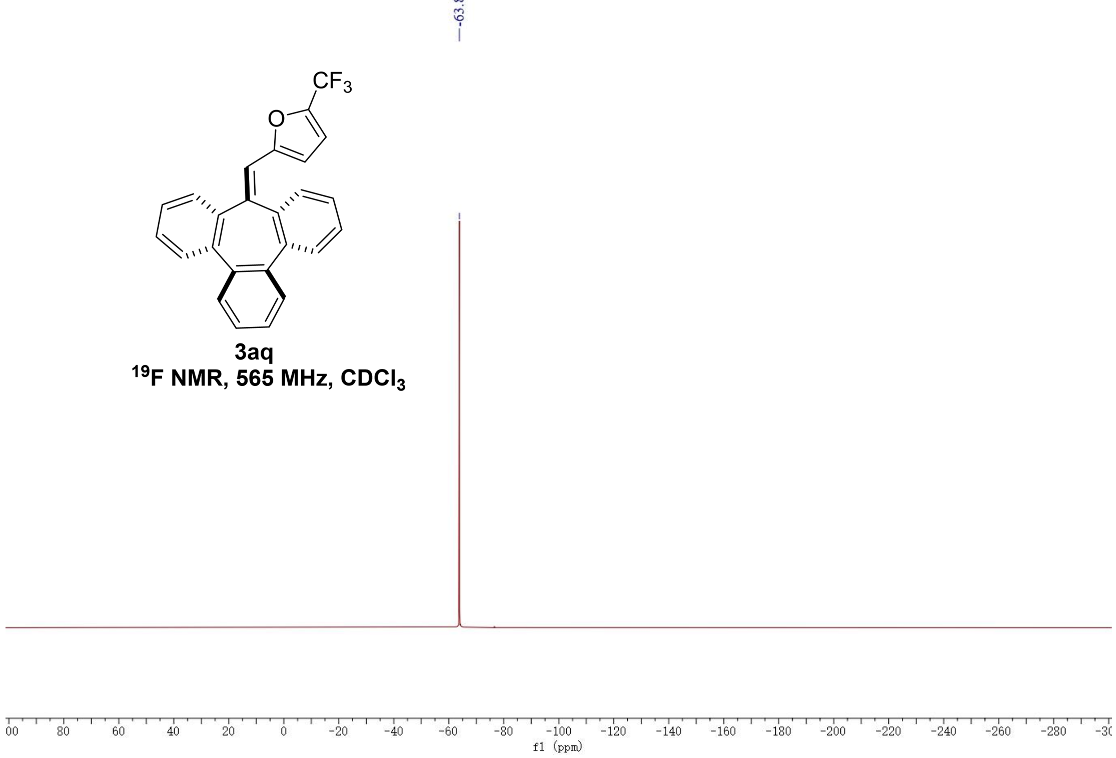

3aq

^{19}F NMR, 565 MHz, CDCl$_3$

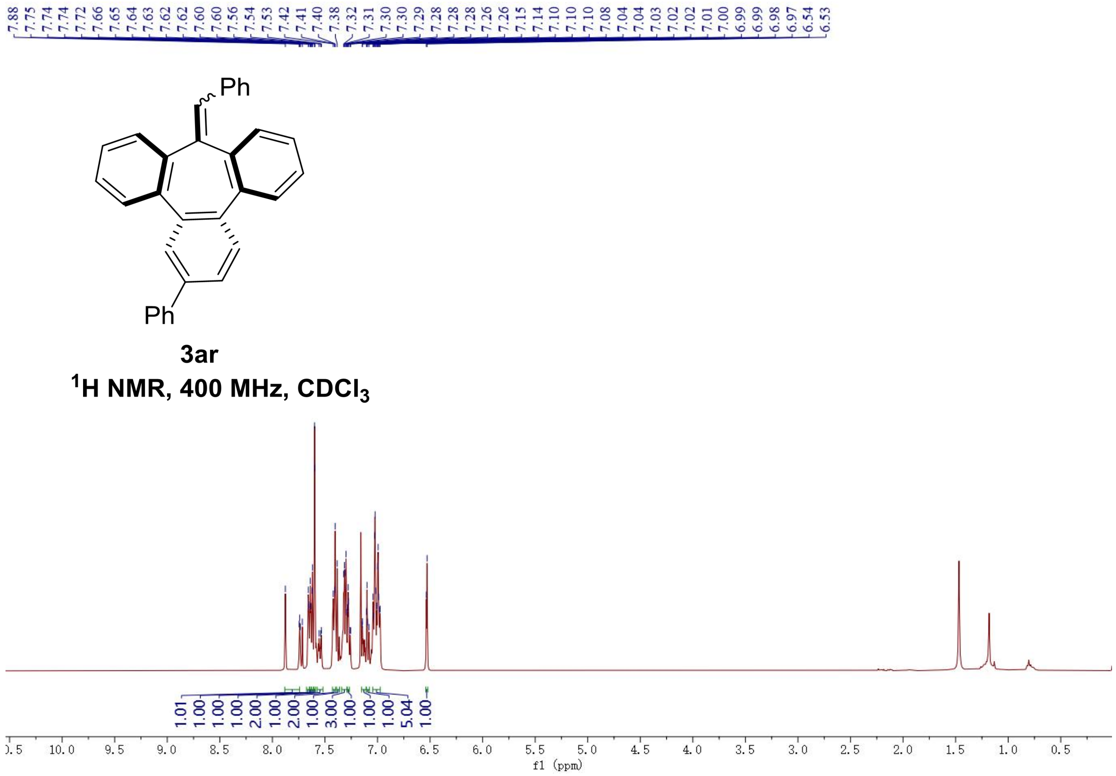
Ph
Ph
3ar
^{1}H NMR, 400 MHz, $CDCl_3$
f1 (ppm)

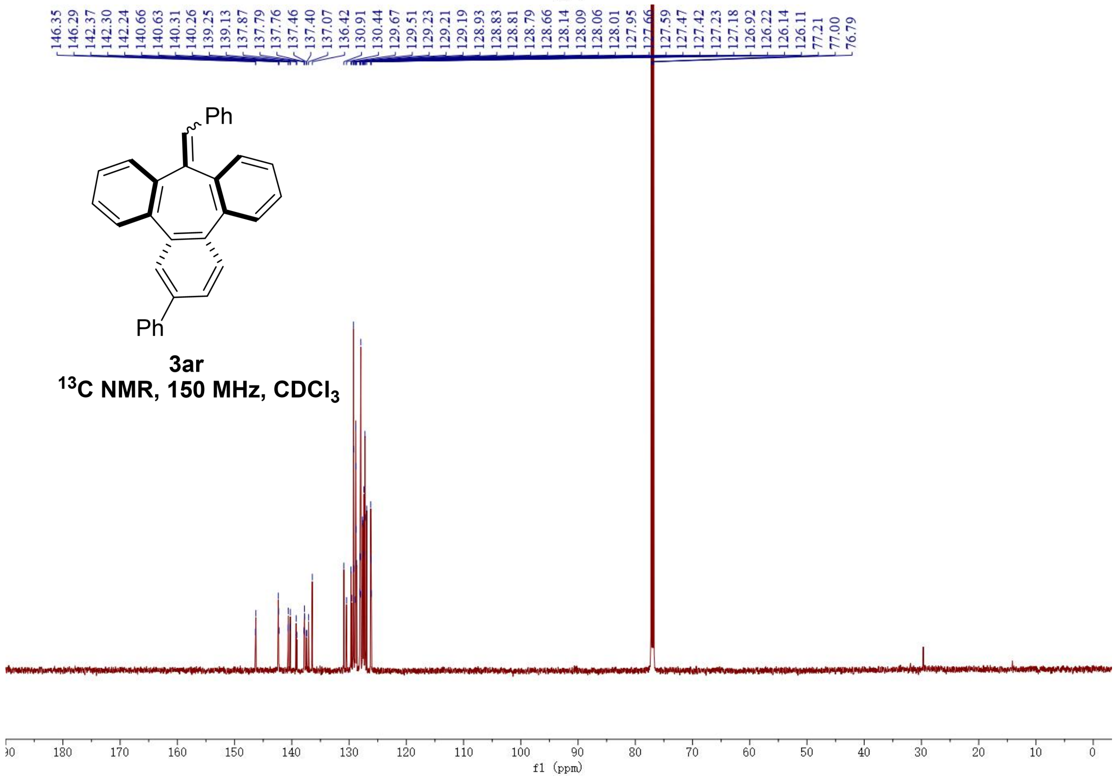
Ph
Ph
3ar
^{13}C NMR, 150 MHz, $CDCl_3$
f1 (ppm)

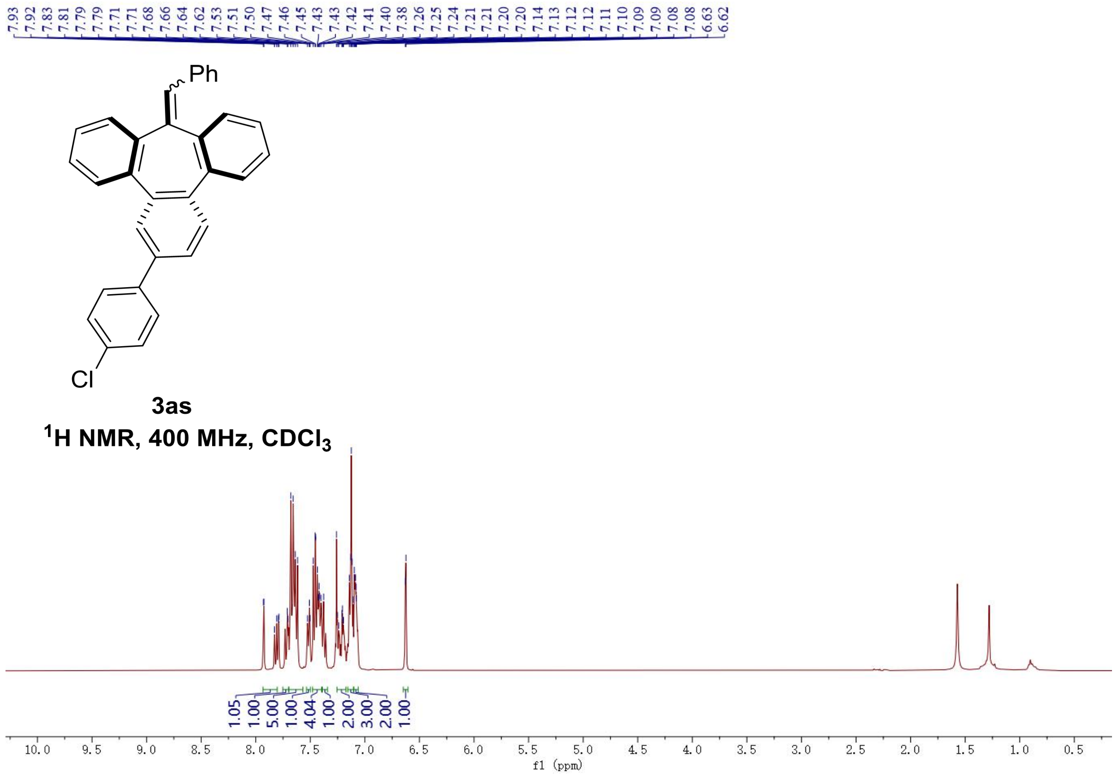
7.93 7.92 7.83 7.81 7.79 7.79 7.71 7.71 7.68 7.66 7.64 7.62 7.53 7.51 7.50 7.47 7.46 7.45 7.43 7.43 7.43 7.42 7.41 7.40 7.38 7.26 7.25 7.24 7.21 7.21 7.20 7.20 7.14 7.13 7.12 7.12 7.11 7.10 7.09 7.09 7.08 7.08 6.63 6.62
Ph
Cl
3as
^{1}H NMR, 400 MHz, $CDCl_3$
1.05 1.00 5.00 1.00 4.04 1.00 2.00 3.00 2.00 1.00
f1 (ppm)

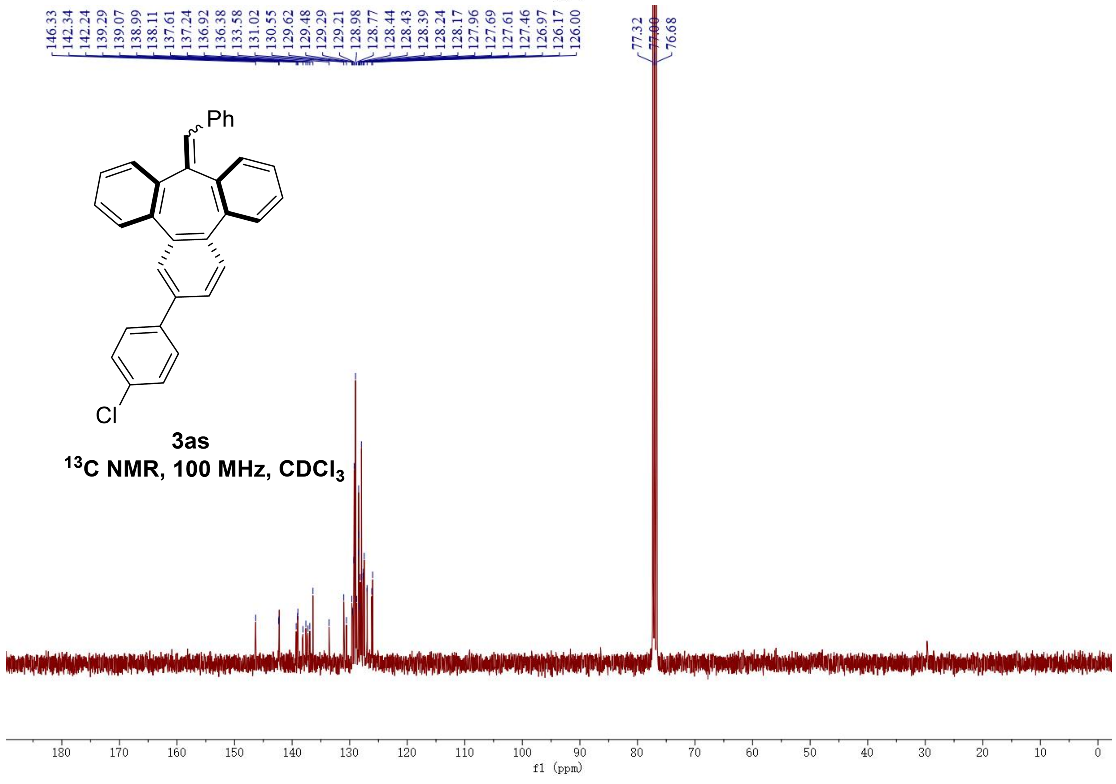
146.33 142.34 142.24 139.29 139.07 138.99 138.11 137.61 137.24 136.92 136.38 133.58 131.02 130.55 129.62 129.48 129.29 129.21 128.98 128.77 128.44 128.43 128.39 128.24 128.17 127.96 127.69 127.61 127.46 126.97 126.17 126.00
77.32 77.00 76.68
Ph
Cl
3as
^{13}C NMR, 100 MHz, $CDCl_3$
f1 (ppm)

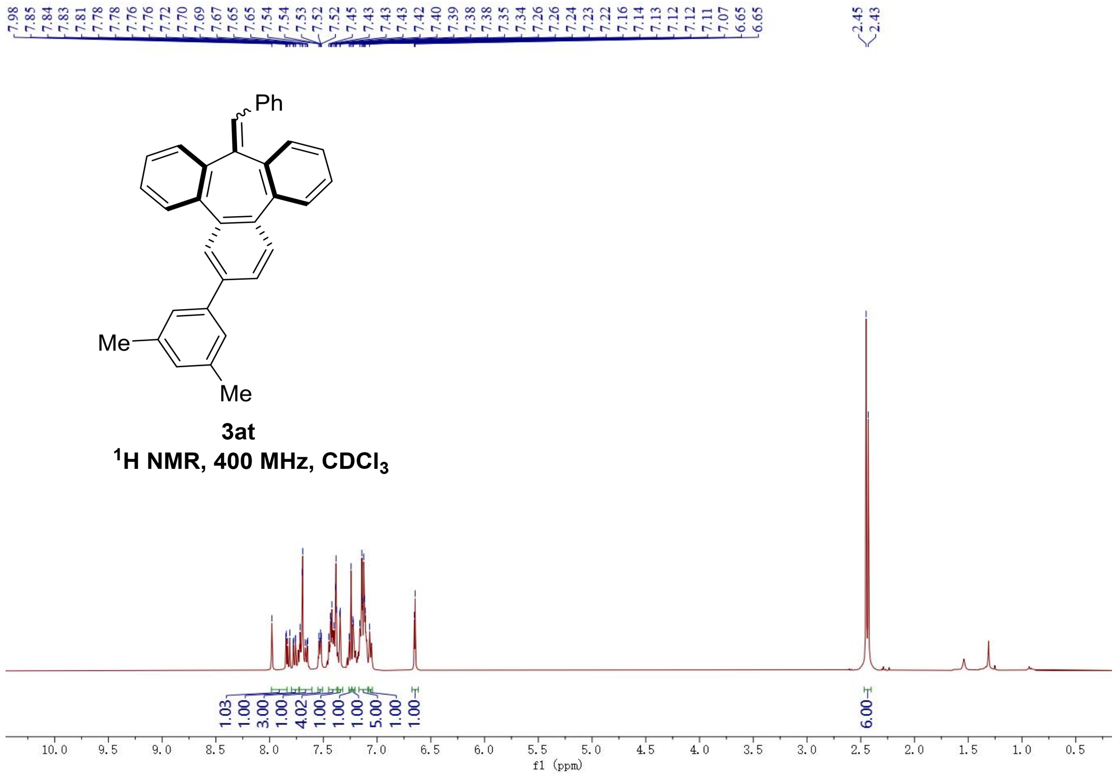
7.98 7.85 7.84 7.83 7.81 7.78 7.78 7.76 7.76 7.72 7.70 7.69 7.67 7.65 7.65 7.54 7.54 7.53 7.52 7.52 7.45 7.43 7.43 7.43 7.42 7.40 7.39 7.38 7.38 7.35 7.34 7.26 7.26 7.24 7.23 7.22 7.16 7.14 7.13 7.12 7.12 7.11 7.07 6.65 6.65
2.45 2.43
Ph
Me
Me
3at
^{1}H NMR, 400 MHz, CDCl$_3$
1.03 1.00 3.00 1.00 4.02 1.00 1.00 1.00 5.00 1.00 1.00
6.00
f1 (ppm)

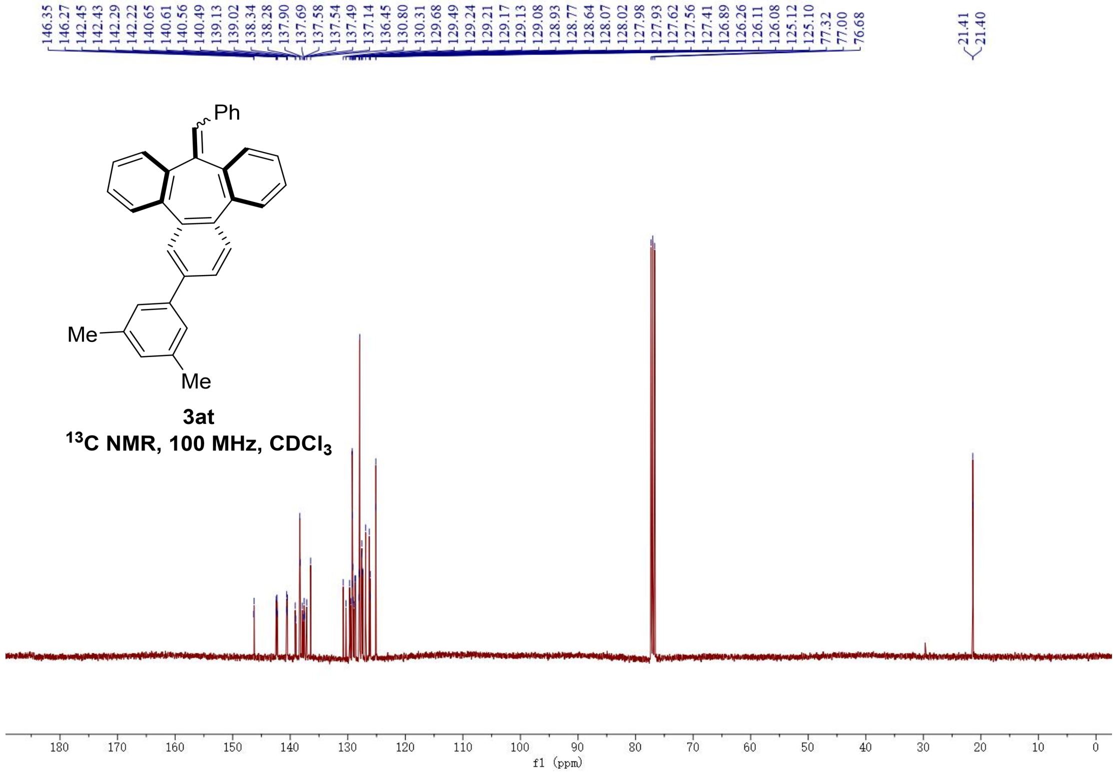
146.35 146.27 142.45 142.43 142.29 142.22 140.65 140.61 140.56 140.49 139.13 139.02 138.34 138.28 137.90 137.69 137.58 137.54 137.49 137.14 136.45 130.80 130.31 129.68 129.49 129.24 129.21 129.17 129.13 129.08 128.93 128.77 128.64 128.07 128.02 127.98 127.93 127.62 127.56 127.41 126.89 126.26 126.11 126.08 125.12 125.10 77.32 77.00 76.68
21.41 21.40
Ph
Me
Me
3at
^{13}C NMR, 100 MHz, CDCl$_3$
f1 (ppm)

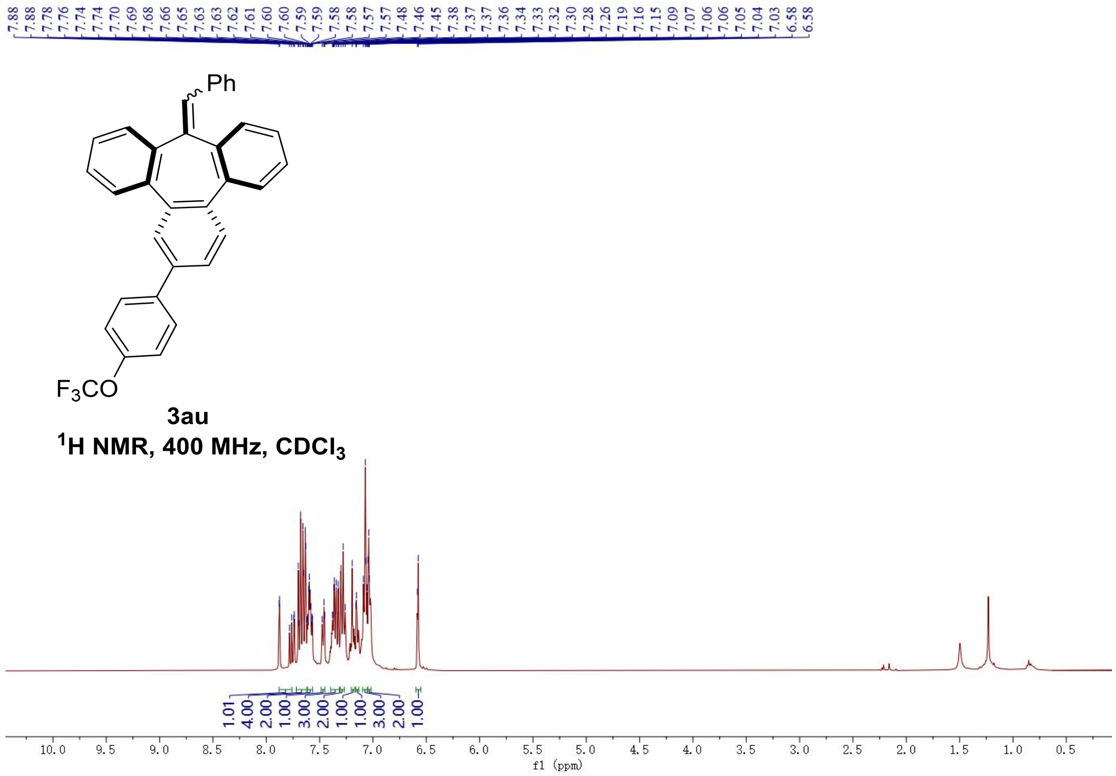
7.88
7.88
7.78
7.76
7.74
7.74
7.70
7.69
7.68
7.66
7.65
7.63
7.63
7.62
7.61
7.60
7.59
7.59
7.59
7.58
7.58
7.57
7.57
7.48
7.46
7.45
7.38
7.37
7.37
7.36
7.34
7.33
7.32
7.30
7.28
7.26
7.19
7.16
7.15
7.09
7.07
7.06
7.06
7.05
7.04
7.03
6.58
6.58
Ph
F_3CO
3au
^{1}H NMR, 400 MHz, $CDCl_3$
1.01
4.00
2.00
1.00
3.00
2.00
1.00
1.00
3.00
2.00
1.00
10.0 9.5 9.0 8.5 8.0 7.5 7.0 6.5 6.0 5.5 5.0 4.5 4.0 3.5 3.0 2.5 2.0 1.5 1.0 0.5
f1 (ppm)

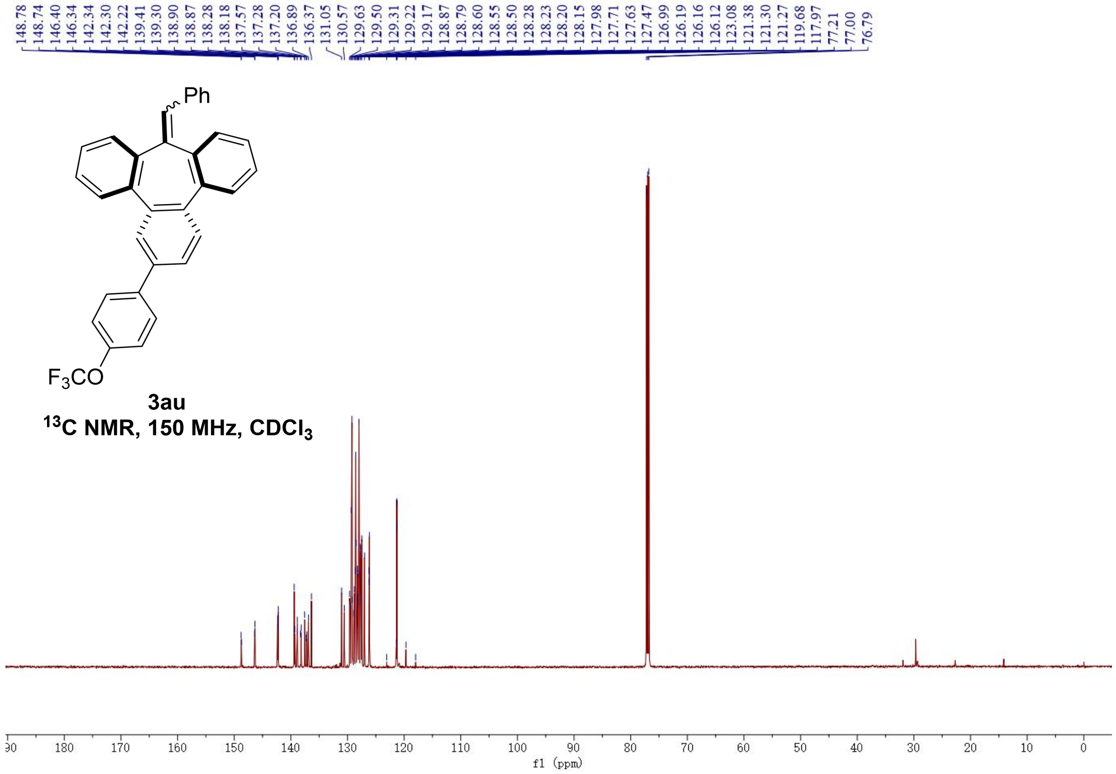
148.78
148.74
146.40
146.34
142.34
142.30
142.22
139.41
139.30
138.90
138.87
138.28
138.18
137.57
137.28
137.20
136.89
136.37
131.05
130.57
129.63
129.50
129.31
129.22
129.17
128.87
128.79
128.60
128.55
128.50
128.28
128.23
128.20
128.15
127.98
127.71
127.63
127.47
126.99
126.19
126.16
126.12
123.08
121.38
121.30
121.27
119.68
117.97
77.21
77.00
76.79
Ph
F_3CO
3au
^{13}C NMR, 150 MHz, $CDCl_3$
90 180 170 160 150 140 130 120 110 100 90 80 70 60 50 40 30 20 10 0
f1 (ppm)

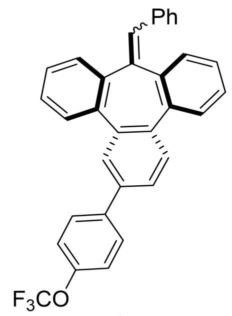

3au

^{19}F NMR, 565 MHz, $CDCl_3$

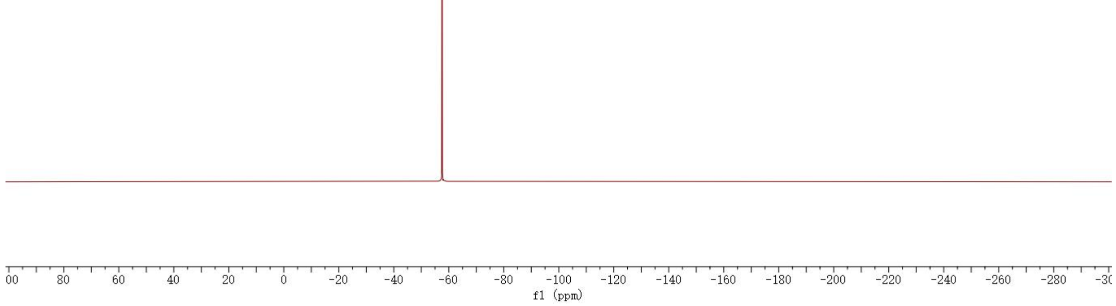

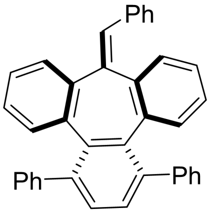

3av

^{1}H NMR, 400 MHz, $CDCl_3$

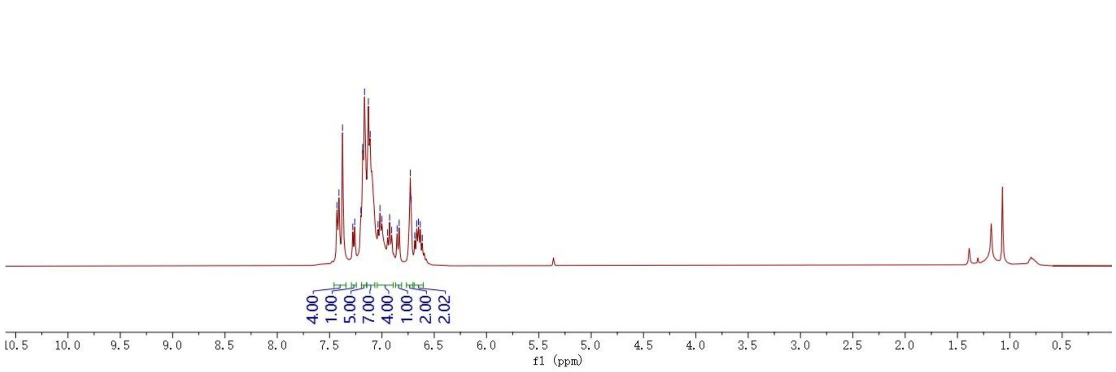

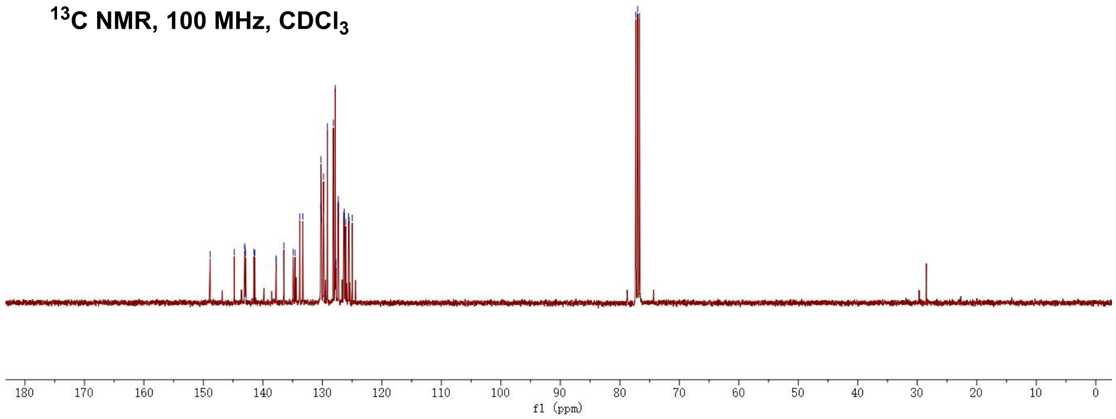

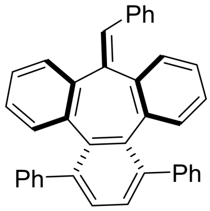

3av
^{13}C NMR, 100 MHz, $CDCl_3$

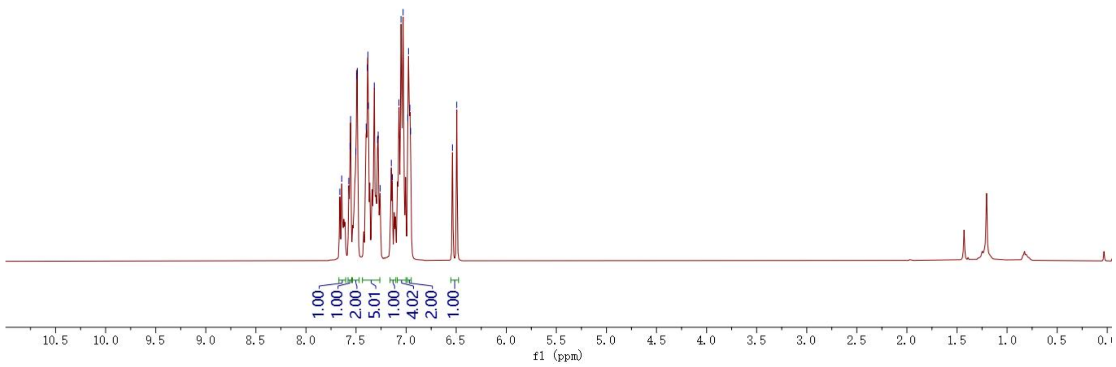

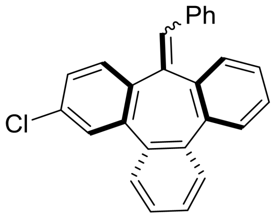

3aw
^{1}H NMR, 400 MHz, $CDCl_3$

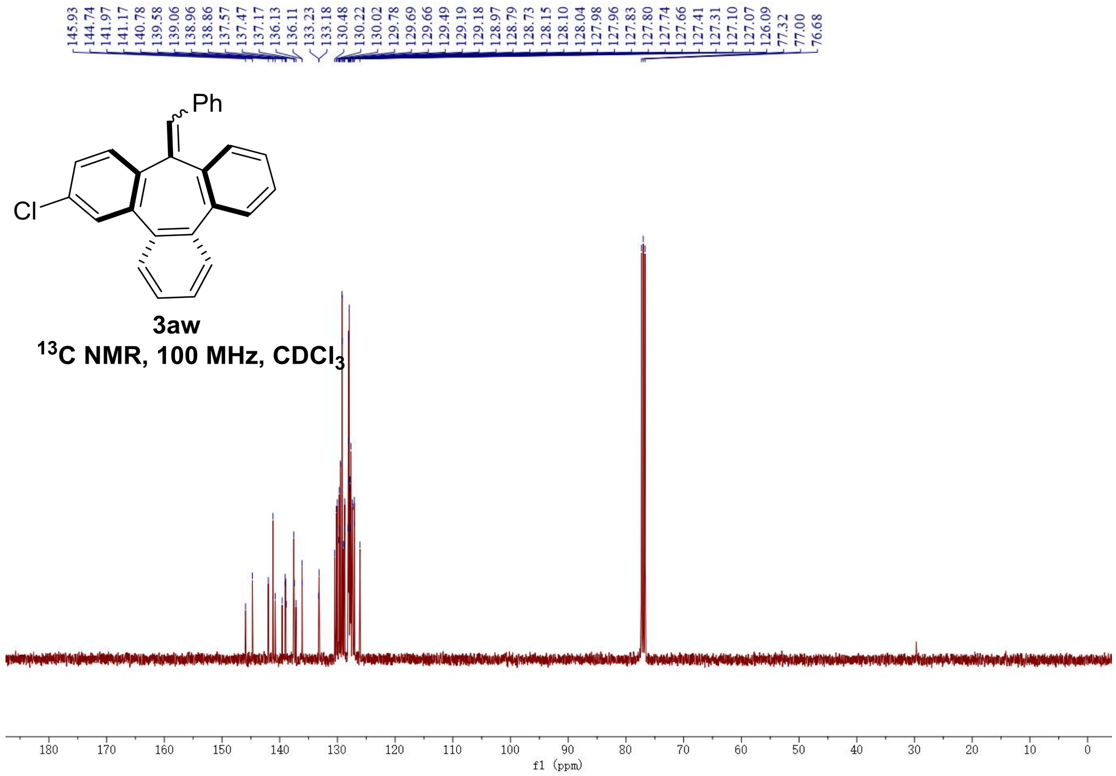

3aw
^{13}C NMR, 100 MHz, $CDCl_3$

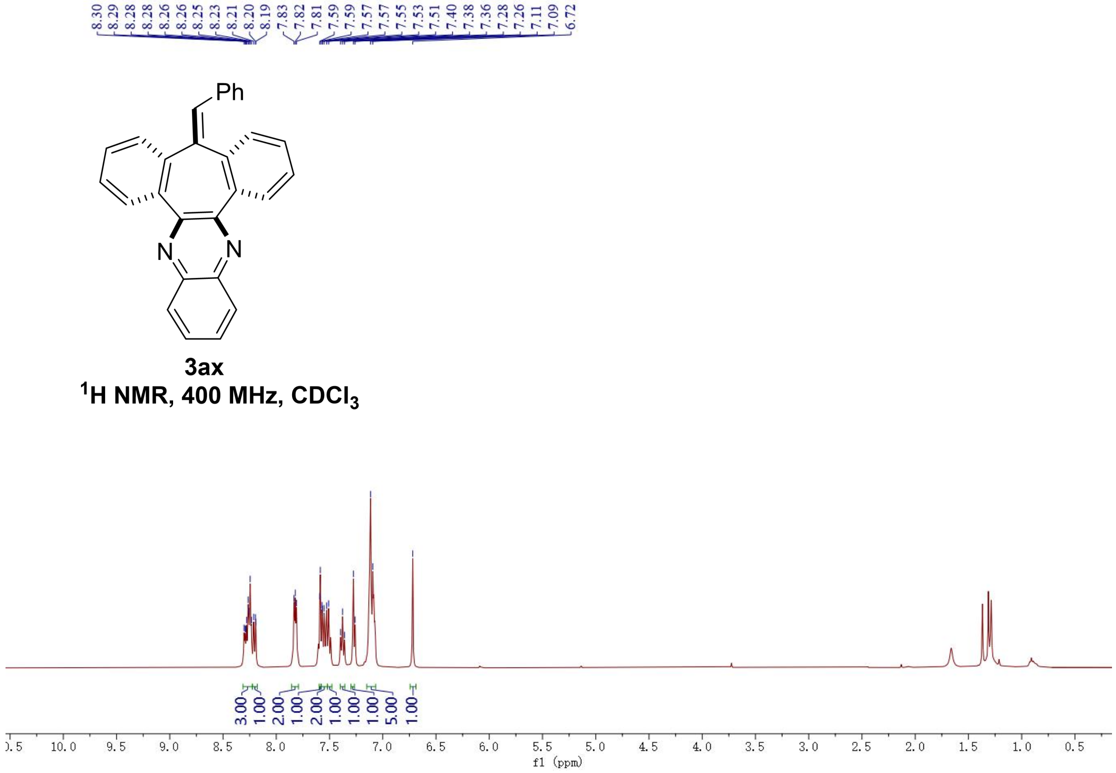

3ax
^{1}H NMR, 400 MHz, $CDCl_3$

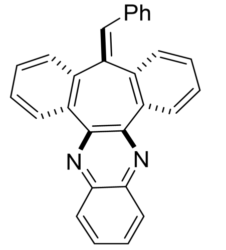

3ax
^{13}C NMR, 100 MHz, $CDCl_3$

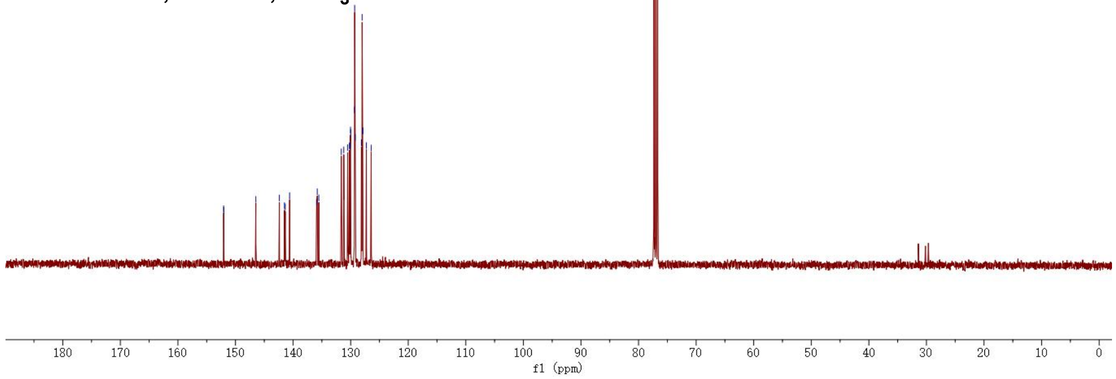

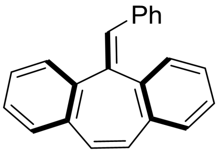

3ay
^{1}H NMR, 400 MHz, $CDCl_3$

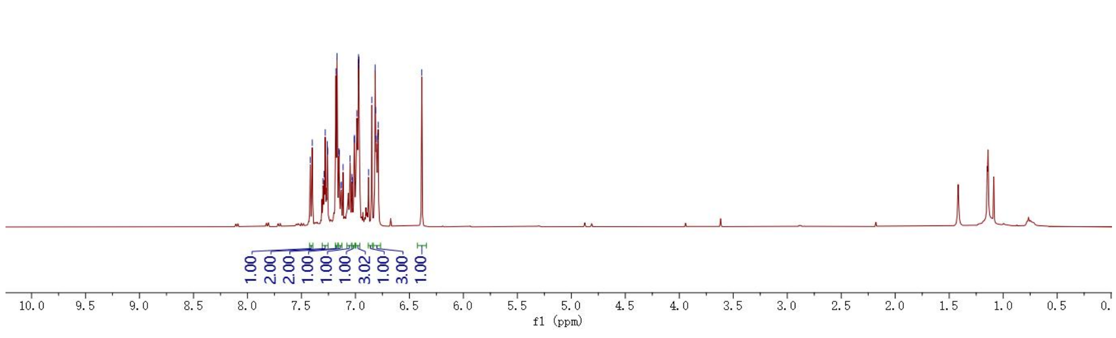

7. HPLC analysis of 3

HPLC conditions: Chiralpak IA-H, 5% ^{i}PrOH/Hx eluent, 0.5 mL/min, 254 nm

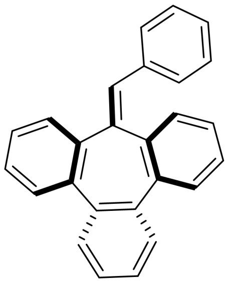

3a

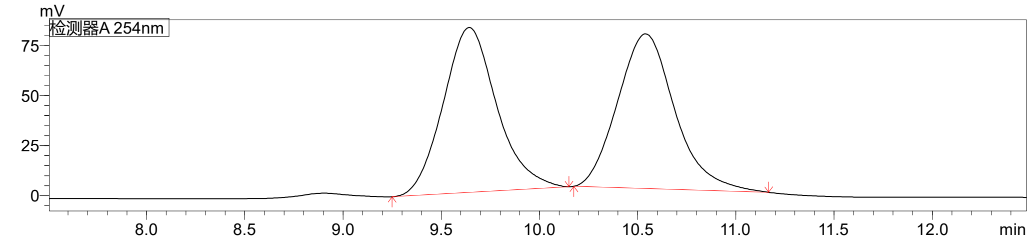

Peak#	Ret. Time	Height	Area%
1	9.642	82447	49.990
2	10.540	77330	50.010

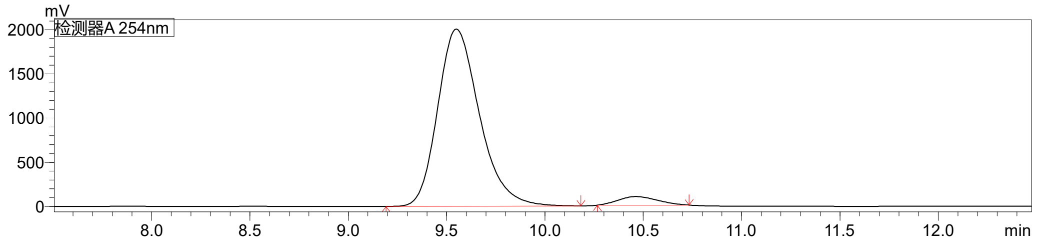

Peak#	Ret. Time	Height	Area%
1	9.550	2007365	95.649
2	10.465	97112	4.351

HPLC conditions: Chiralpak IA-H, 5% ^{i}PrOH/Hx eluent, 0.5 mL/min, 254 nm

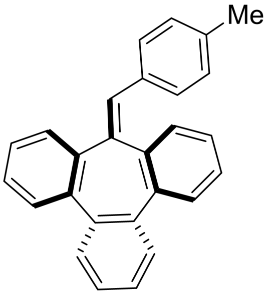

3b

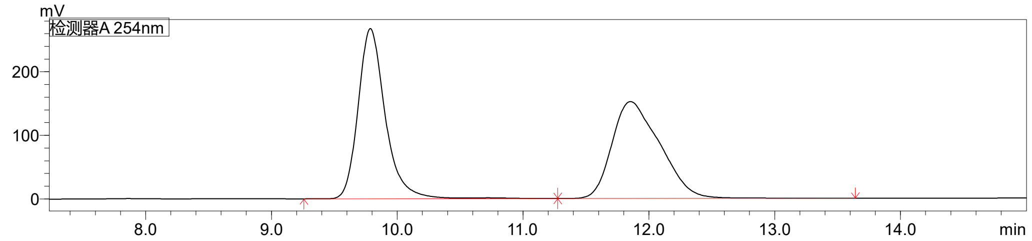

Peak#	Ret. Time	Height	Area%
1	9.786	268029	50.033
2	11.853	152656	49.967

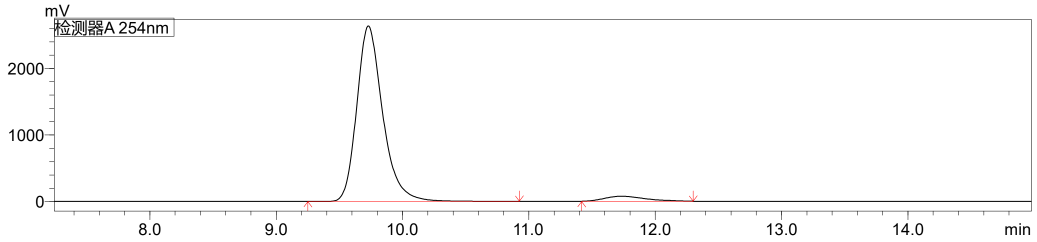

Peak#	Ret. Time	Height	Area%
1	9.729	2640159	95.675
2	11.734	75780	4.325

HPLC conditions: Chiralpak AD-H, 5% iPrOH/Hx eluent, 0.5 mL/min, 254 nm

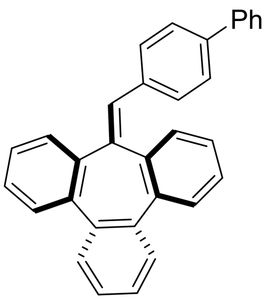

3c

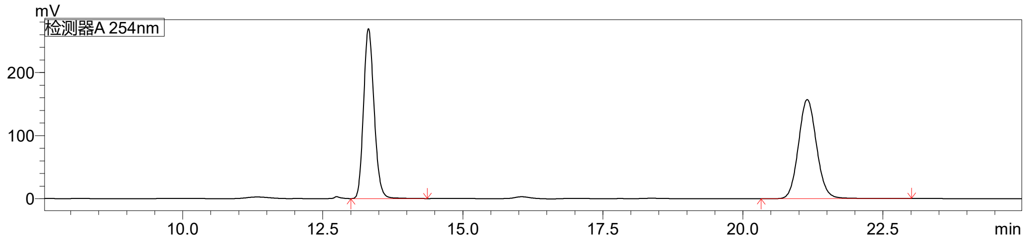

Peak#	Ret. Time	Height	Area%
1	13.314	269447	50.032
2	21.147	157173	49.968

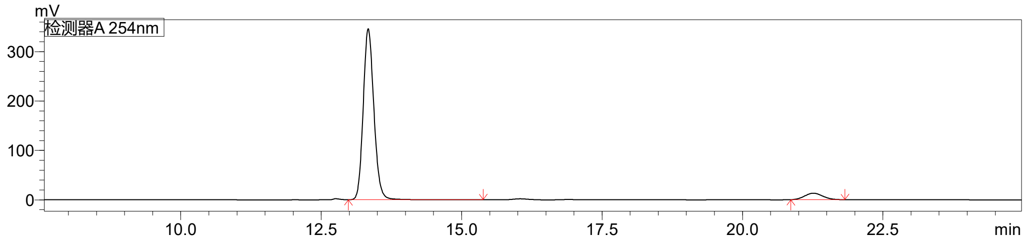

Peak#	Ret. Time	Height	Area%
1	13.336	346266	93.941
2	21.261	13176	6.059

HPLC conditions: Chiralpak IA-H, 5% iPrOH/Hx eluent, 0.5 mL/min, 254 nm

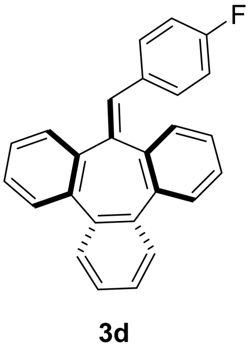

3d

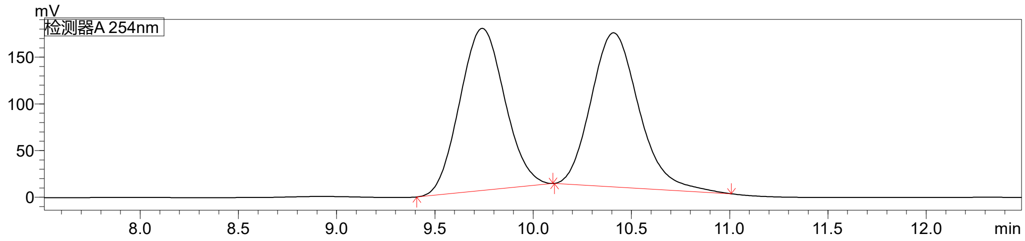

Peak#	Ret. Time	Height	Area%
1	9.741	173573	49.716
2	10.409	164960	50.284

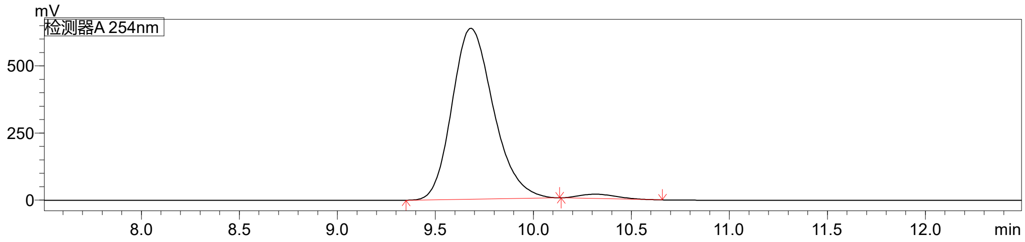

Peak#	Ret. Time	Height	Area%
1	9.681	637194	97.804
2	10.315	16138	2.196

HPLC conditions: Chiralpak IA-H, 5% iPrOH/Hx eluent, 0.5 mL/min, 254 nm

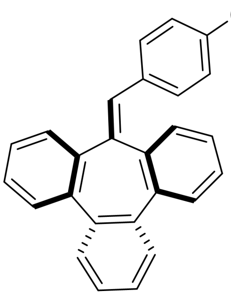

3e

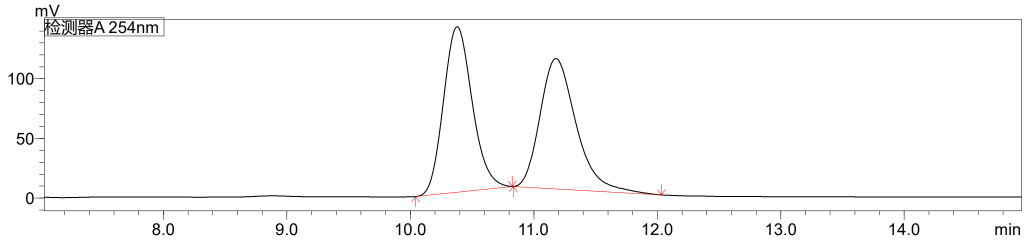

Peak#	Ret. Time	Height	Area%
1	10.379	138744	50.168

2	11.178	109336	49.832

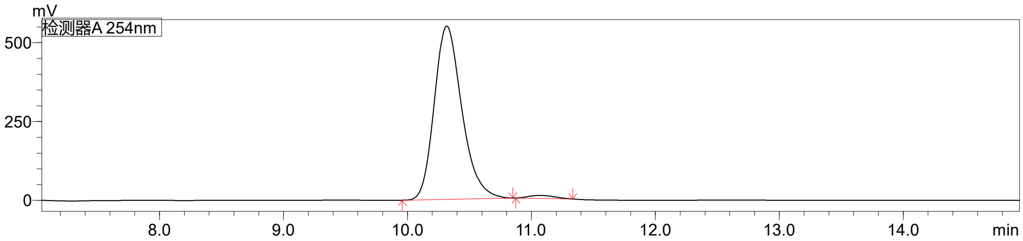

Peak#	Ret. Time	Height	Area%
1	10.316	550027	98.376
2	11.068	9677	1.624

HPLC conditions: Chiralpak AD-H, 5% ^{i}PrOH/Hx eluent, 0.5 mL/min, 254 nm

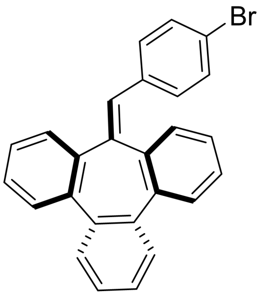

3f

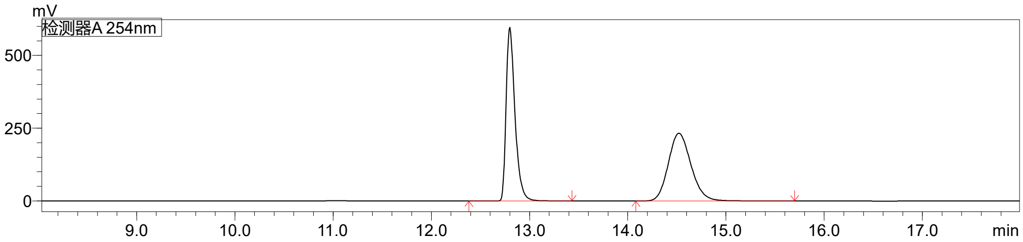

Peak#	Ret. Time	Height	Area%
1	12.798	596680	50.020
2	14.521	232826	49.980

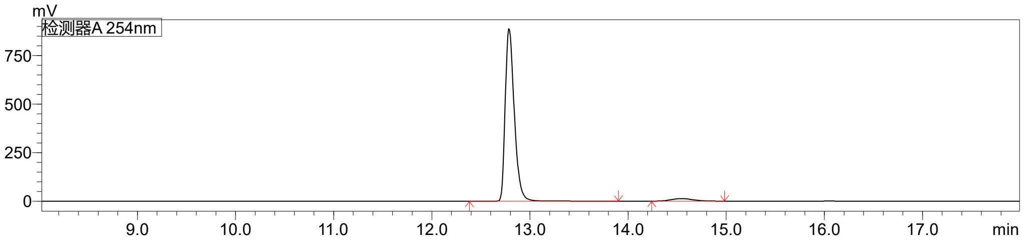

Peak#	Ret. Time	Height	Area%

1	12.787	888769	96.528
2	14.544	13175	3.472

HPLC conditions: Chiralpak AD-H, 10% iPrOH/Hx eluent, 0.5 mL/min, 254 nm

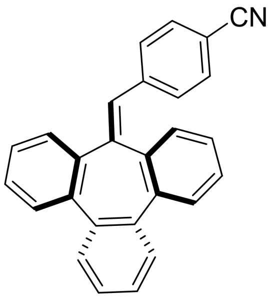

3g

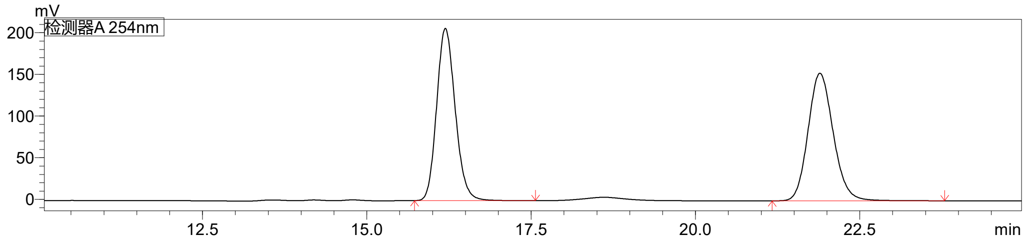

Peak#	Ret. Time	Height	Area%
1	16.194	206660	49.882
2	21.892	153031	50.118

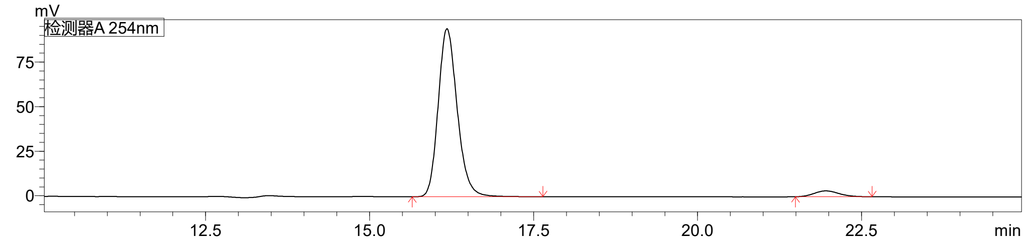

Peak#	Ret. Time	Height	Area%
1	16.179	94231	95.623
2	21.955	3289	4.377

HPLC conditions: Chiralpak IA-H, 5% iPrOH/Hx eluent, 0.5 mL/min, 254 nm

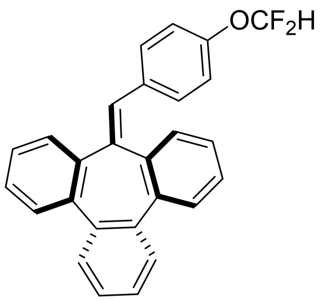

3h

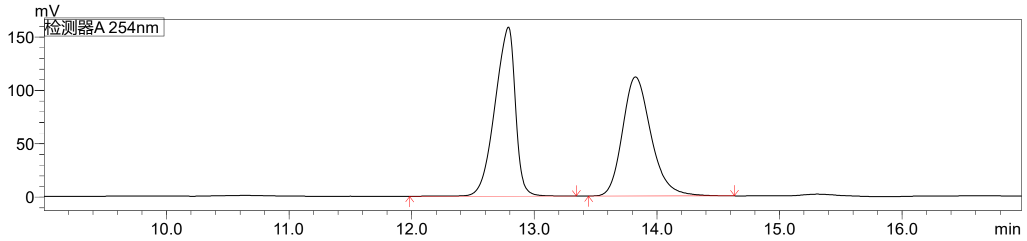

Peak#	Ret. Time	Height	Area%
1	12.788	158514	50.331
2	13.825	111730	49.669

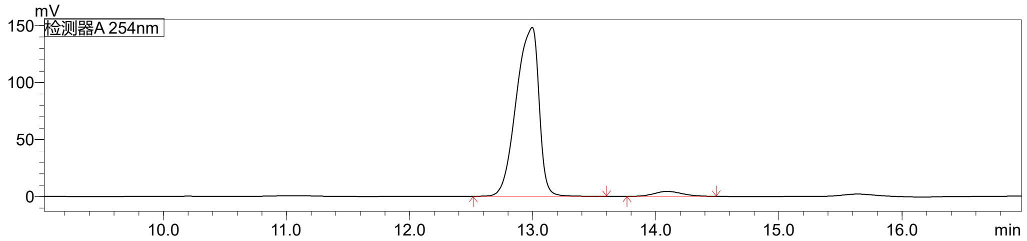

Peak#	Ret. Time	Height	Area%
1	12.996	148030	96.508
2	14.094	4321	3.492

HPLC conditions: Chiralpak IA-H, 20% iPrOH/Hx eluent, 0.5 mL/min, 254 nm

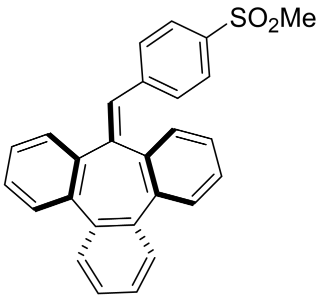

3i

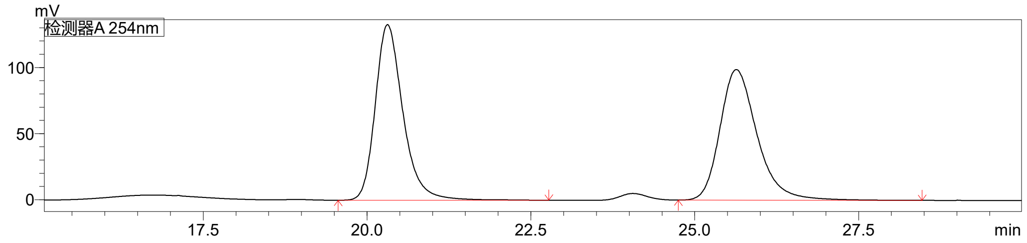

Peak#	Ret. Time	Height	Area%
1	20.312	132810	50.100
2	25.633	98811	49.900

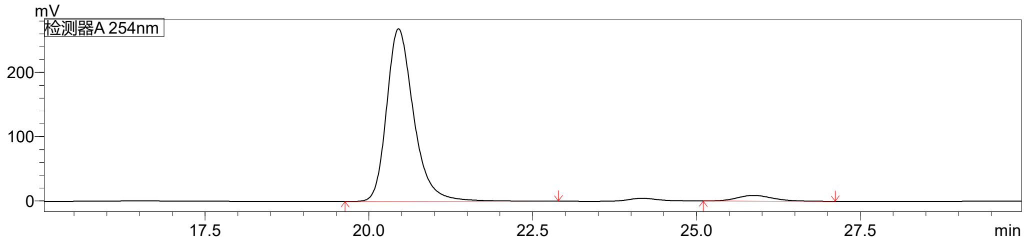

Peak#	Ret. Time	Height	Area%
1	20.451	268710	95.917
2	25.869	8809	4.083

HPLC conditions: Chiralpak AD-H, 25% iPrOH/Hx eluent, 0.5 mL/min, 254 nm

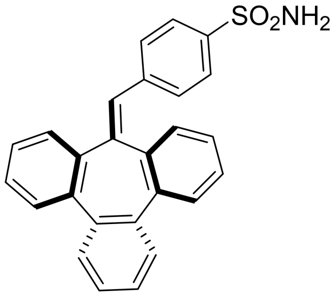

3j

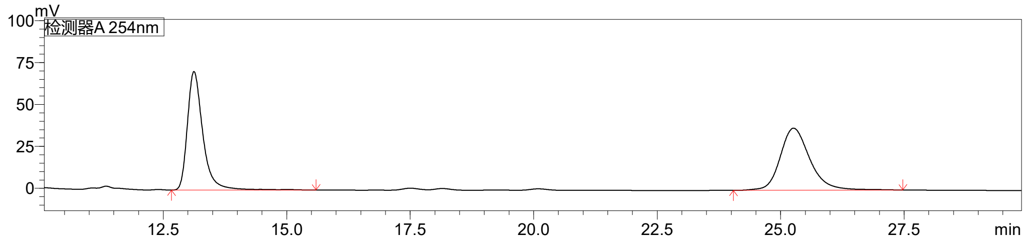

Peak#	Ret. Time	Height	Area%
1	13.121	70712	49.991

2	25.261	37037	50.009

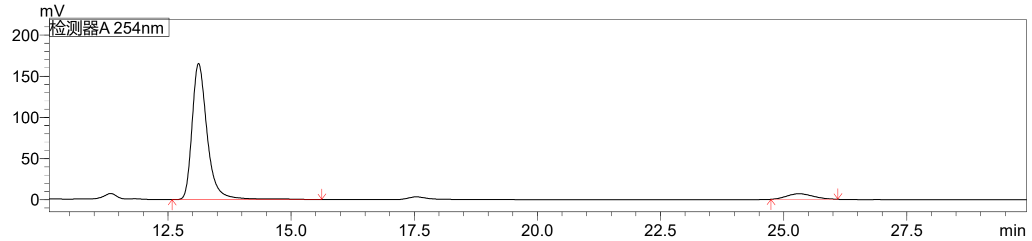

Peak#	Ret. Time	Height	Area%
1	13.120	165296	93.387
2	25.312	6718	6.613

HPLC conditions: Chiralpak AD-H, 5% iPrOH/Hx eluent, 0.5 mL/min, 254 nm

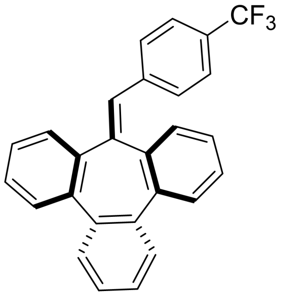

3k

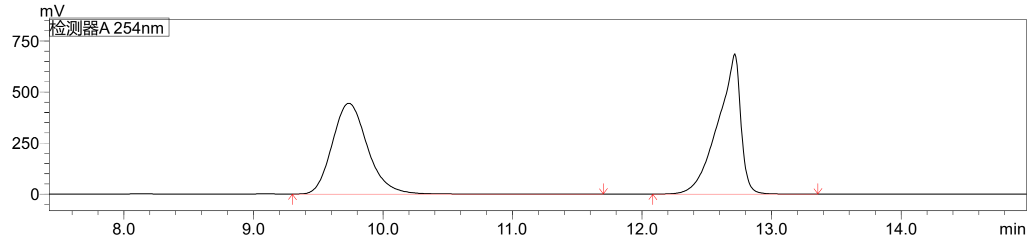

Peak#	Ret. Time	Height	Area%
1	9.736	444938	49.938
2	12.716	687122	50.062

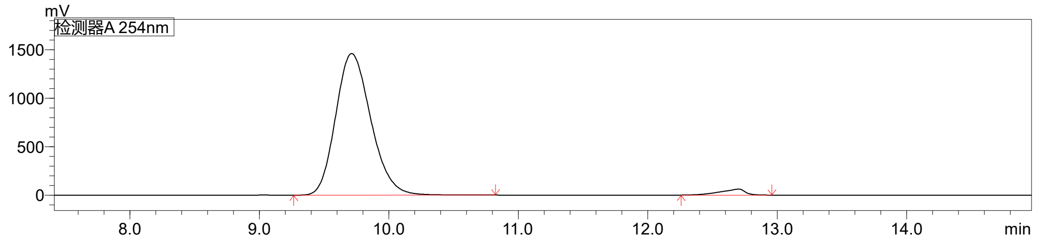

Peak#	Ret. Time	Height	Area%
1	9.713	1460083	97.038
2	12.701	63291	2.962

HPLC conditions: Chiralpak AD-H, 5% iPrOH/Hx eluent, 0.5 mL/min, 254 nm

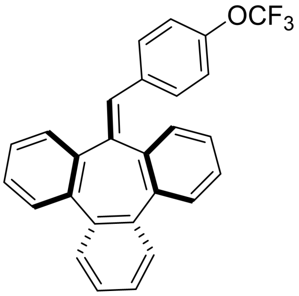

3l

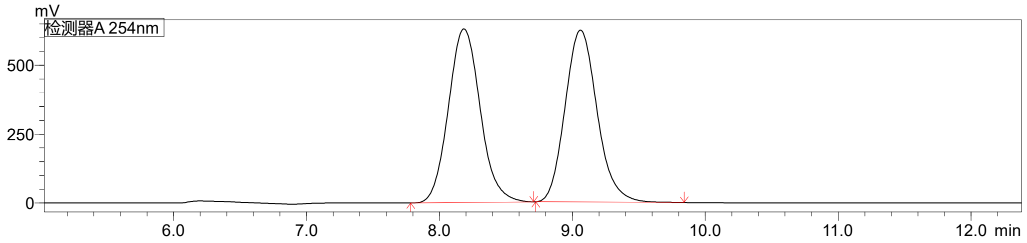

Peak#	Ret. Time	Height	Area%
1	8.184	630413	50.089
2	9.061	623875	49.911

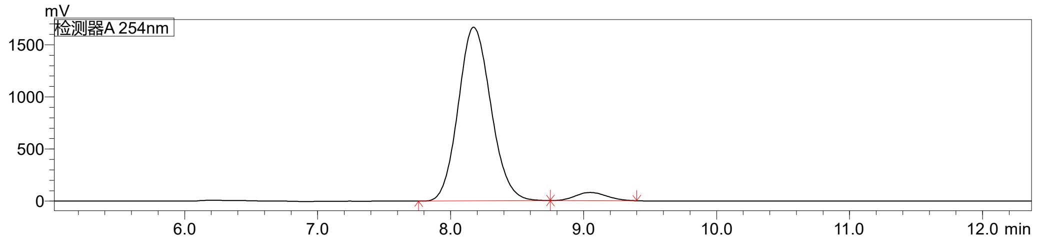

Peak#	Ret. Time	Height	Area%
1	8.173	1667584	95.651
2	9.049	78714	4.349

HPLC conditions: Chiralpak AD-H, 2% iPrOH/Hx eluent, 0.5 mL/min, 254 nm

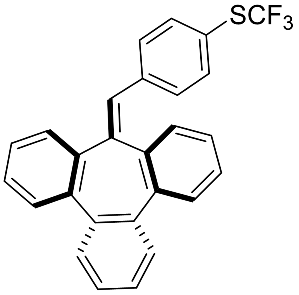

3m

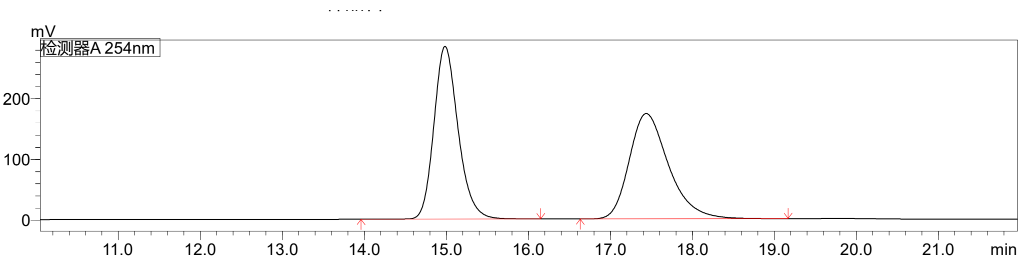

Peak#	Ret. Time	Height	Area%
1	14.983	284579	50.154
2	17.437	173380	49.846

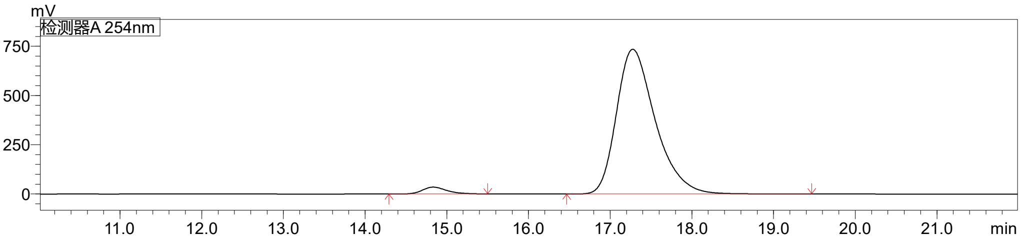

Peak#	Ret. Time	Height	Area%
1	14.834	34677	2.910
2	17.276	734506	97.090

HPLC conditions: Chiralpak AD-H, 10% iPrOH/Hx eluent, 0.5 mL/min, 254 nm

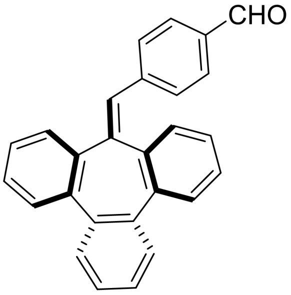

3n

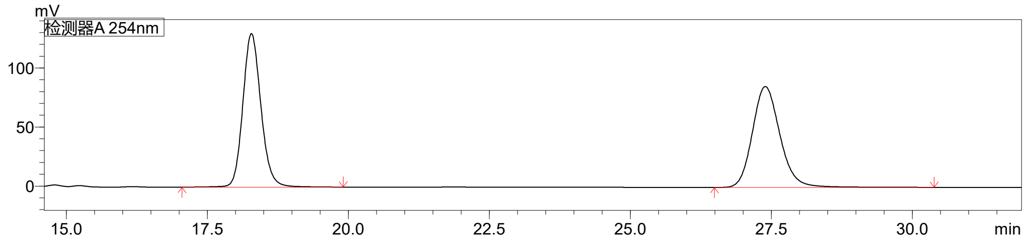

Peak#	Ret. Time	Height	Area%
1	18.281	130092	50.005
2	27.392	85491	49.995

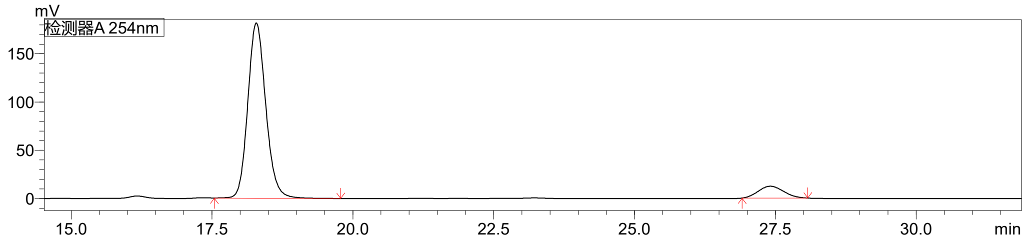

Peak#	Ret. Time	Height	Area%
1	18.285	181691	91.315
2	27.410	12299	8.685

HPLC conditions: Chiralpak AD-H, 10% iPrOH/Hx eluent, 0.5 mL/min, 254 nm

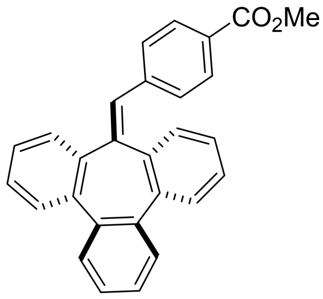

3o

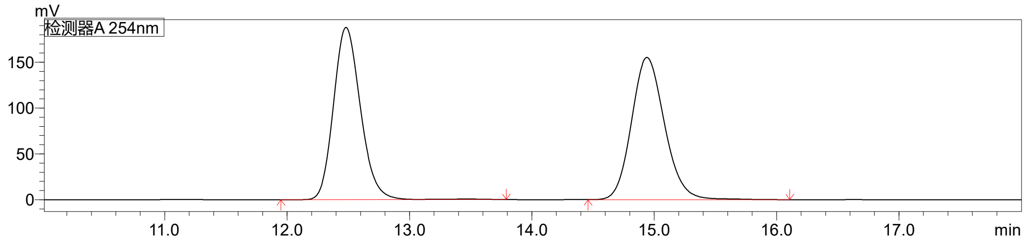

Peak#	Ret. Time	Height	Area%
1	12.481	188017	49.613

2	14.940	155143	50.387

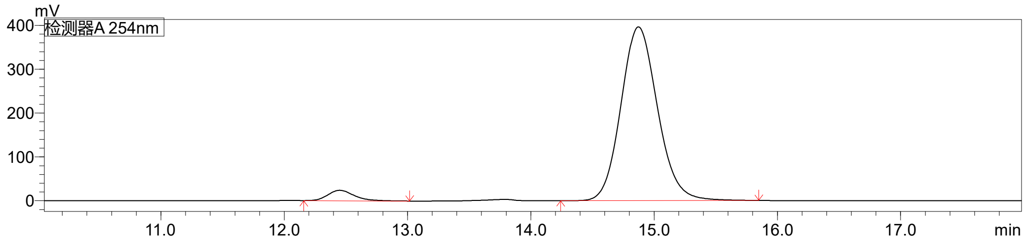

Peak#	Ret. Time	Height	Area%
1	12.451	23829	4.155
2	14.872	395847	95.845

HPLC conditions: Chiralpak AD-H, 5% iPrOH/Hx eluent, 0.5 mL/min, 254 nm

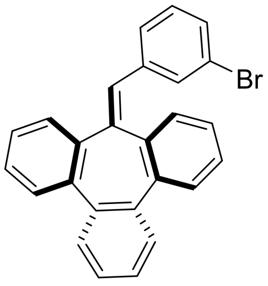

3p

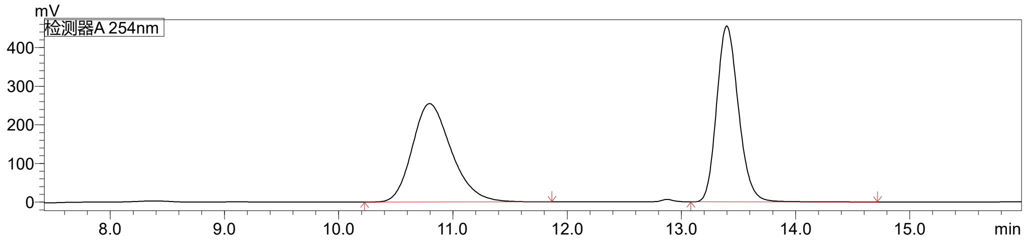

Peak#	Ret. Time	Height	Area%
1	10.795	254495	50.054
2	13.397	455171	49.946

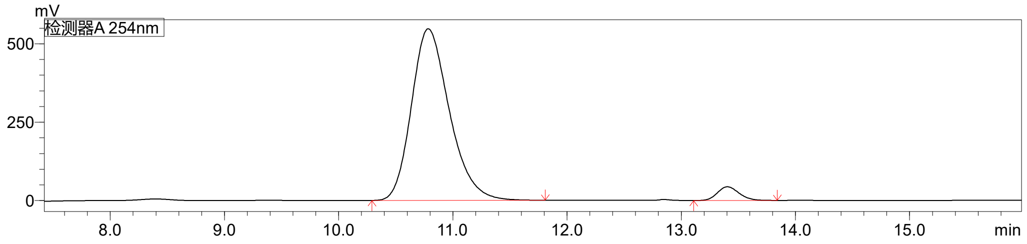

Peak#	Ret. Time	Height	Area%

1	10.784	547717	95.656
2	13.404	43723	4.344

HPLC conditions: Chiralpak IA-H, 5% iPrOH/Hx eluent, 0.5 mL/min, 254 nm

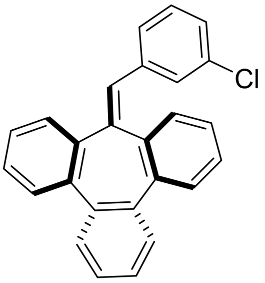

3q

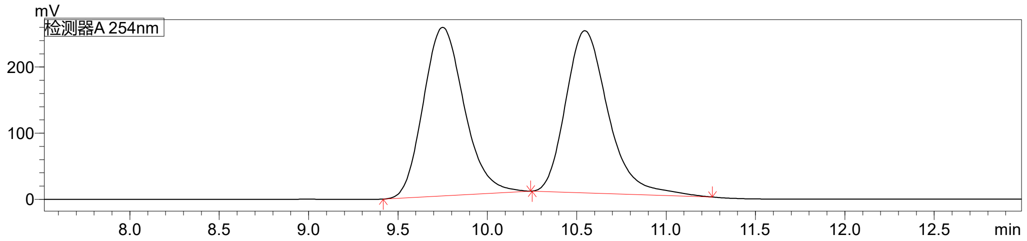

Peak#	Ret. Time	Height	Area%
1	9.750	254945	50.006
2	10.545	245124	49.994

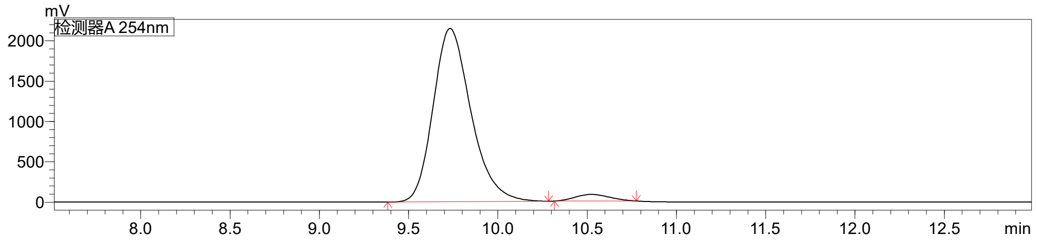

Peak#	Ret. Time	Height	Area%
1	9.732	2149216	96.634
2	10.523	82239	3.366

HPLC conditions: Chiralpak AD-H, 5% iPrOH/Hx eluent, 0.5 mL/min, 254 nm

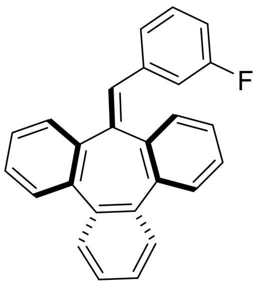

3r

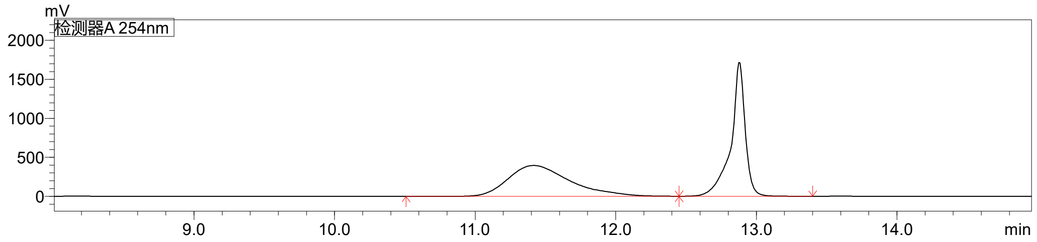

Peak#	Ret. Time	Height	Area%
1	11.415	395426	50.016
2	12.879	1715011	49.984

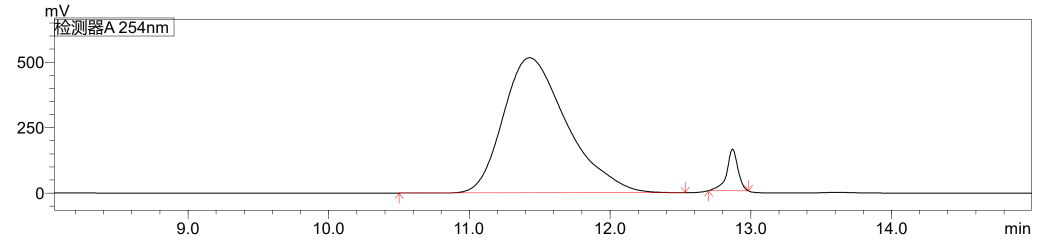

Peak#	Ret. Time	Height	Area%
1	11.428	516339	94.862
2	12.870	159549	5.138

HPLC conditions: Chiralpak IA-H, 10% iPrOH/Hx eluent, 0.5 mL/min, 254 nm

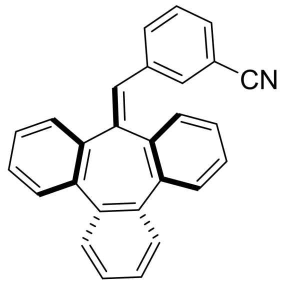

3s

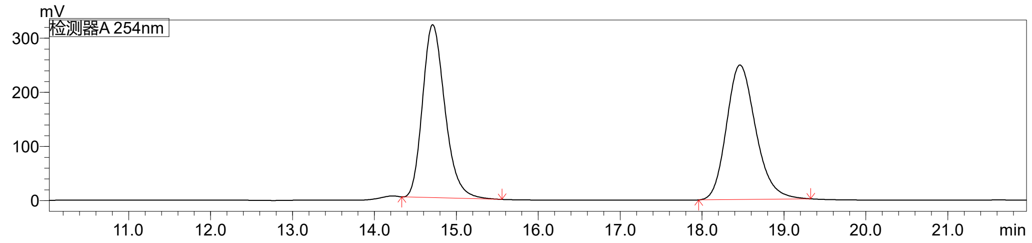

Peak#	Ret. Time	Height	Area%
1	14.710	319666	49.608
2	18.461	248563	50.392

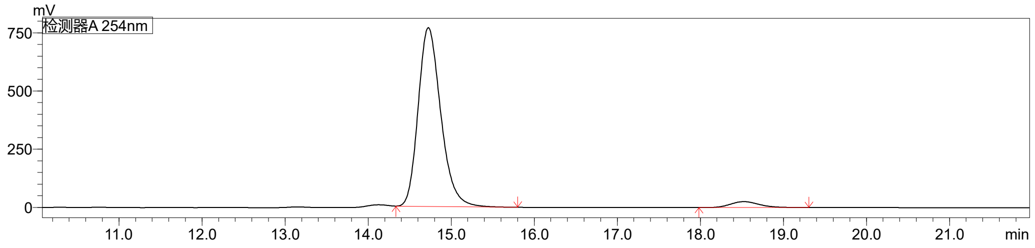

Peak#	Ret. Time	Height	Area%
1	14.724	768311	95.955
2	18.521	25514	4.045

HPLC conditions: Chiralpak IA-H, 5% iPrOH/Hx eluent, 0.5 mL/min, 254 nm

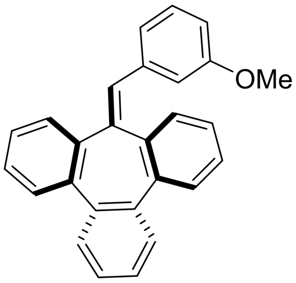

3t

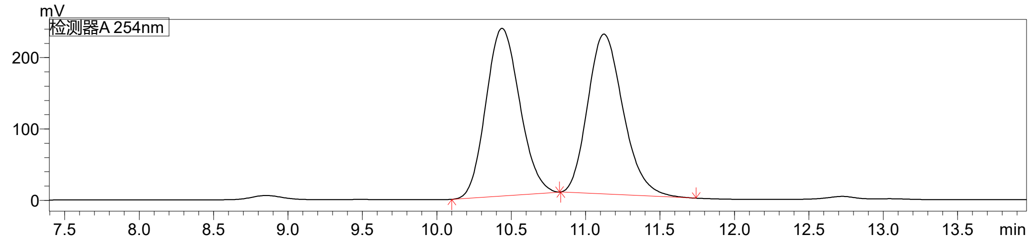

Peak#	Ret. Time	Height	Area%
1	10.438	234785	50.013

2	11.123	223990	49.987

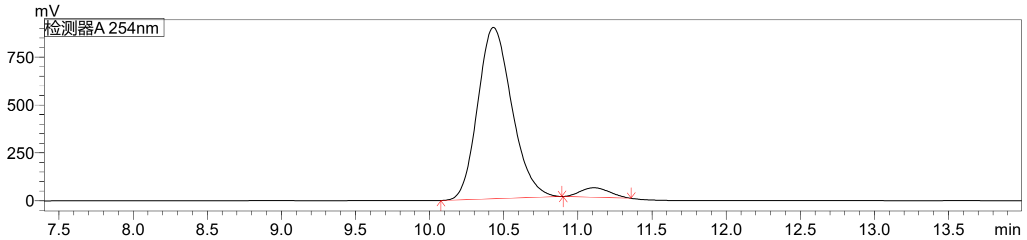

Peak#	Ret. Time	Height	Area%
1	10.430	895379	95.426
2	11.107	49827	4.574

HPLC conditions: Chiralpak AD-H, 10% iPrOH/Hx eluent, 0.5 mL/min, 254 nm

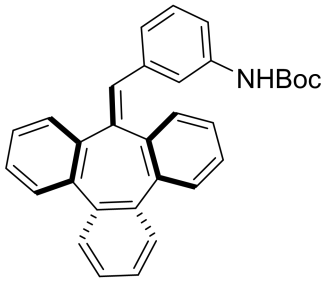

3u

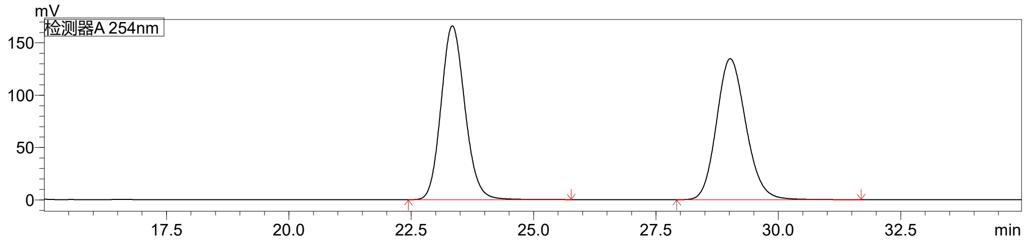

Peak#	Ret. Time	Height	Area%
1	23.339	166134	50.002
2	29.016	134711	49.998

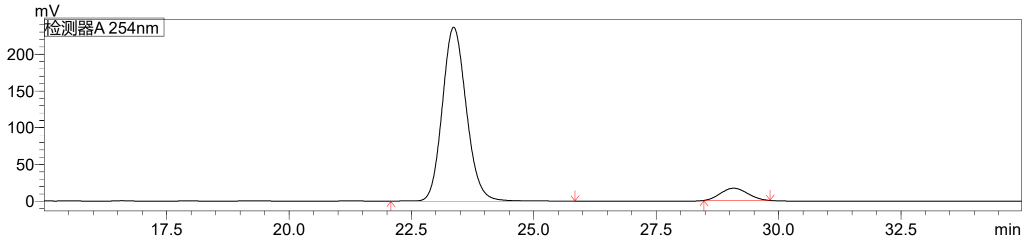

Peak#	Ret. Time	Height	Area%

1	23.363	236561	92.681
2	29.082	16669	7.319

HPLC conditions: Chiralpak AD-H, 5% iPrOH/Hx eluent, 0.5 mL/min, 254 nm

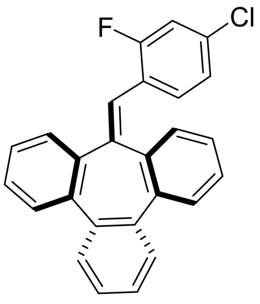

3v

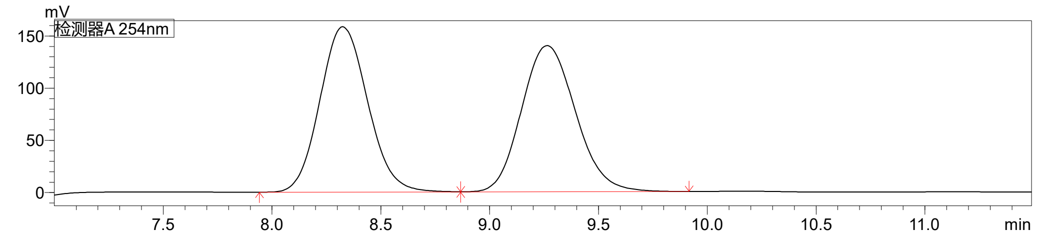

Peak#	Ret. Time	Height	Area%
1	8.324	158372	49.980
2	9.264	139985	50.020

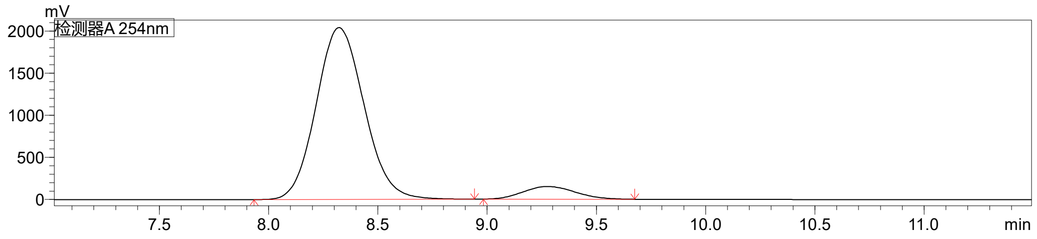

Peak#	Ret. Time	Height	Area%
1	8.322	2040144	92.563
2	9.277	150565	7.437

HPLC conditions: Chiralpak OD-H, 5% ^{i}PrOH/Hx eluent, 0.5 mL/min, 254 nm

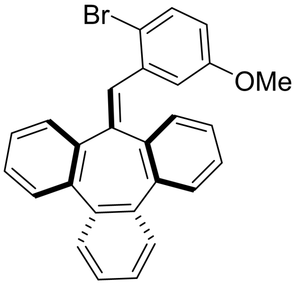

3w

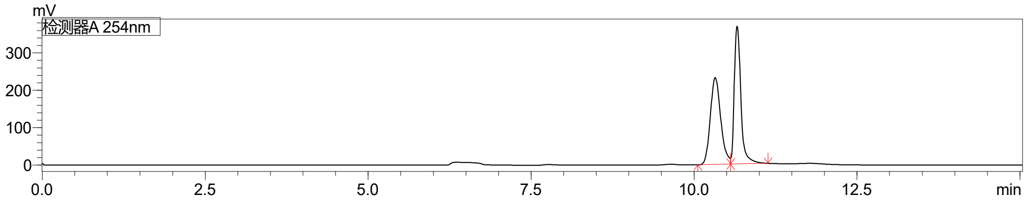

Peak#	Ret. Time	Height	Area%
1	10.324	232785	49.931
2	10.661	368395	50.069

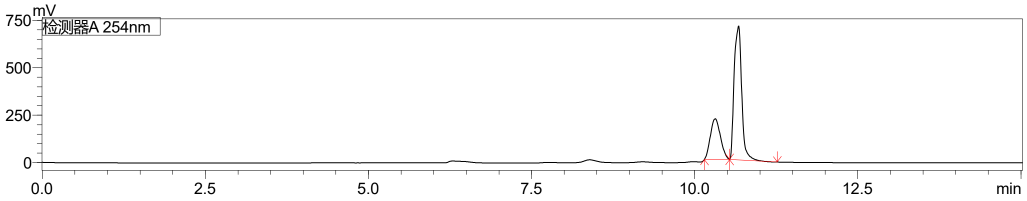

Peak#	Ret. Time	Height	Area%
1	10.313	214293	28.745
2	10.672	706349	71.255

HPLC conditions: Chiralpak IA-H, 5% ^{i}PrOH/Hx eluent, 0.5 mL/min, 254 nm

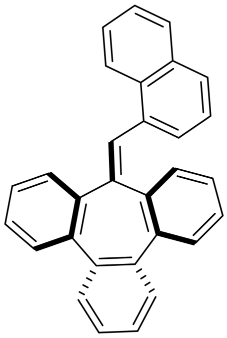

3x

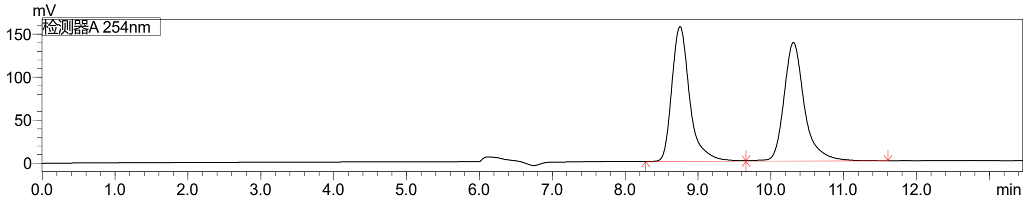

Peak#	Ret. Time	Height	Area%
1	8.753	156728	49.857
2	10.309	137882	50.143

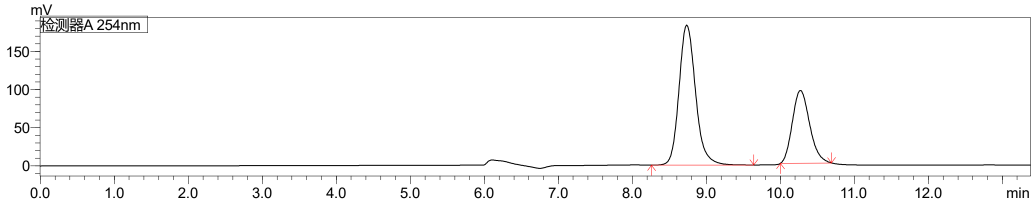

Peak#	Ret. Time	Height	Area%
1	8.734	183635	64.893
2	10.271	95322	35.107

HPLC conditions: Chiralpak AD-H, 5% iPrOH/Hx eluent, 0.5 mL/min, 254 nm

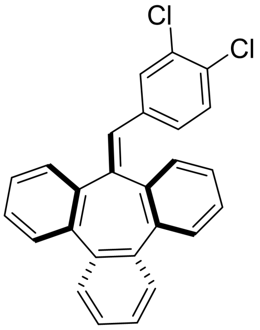

3y

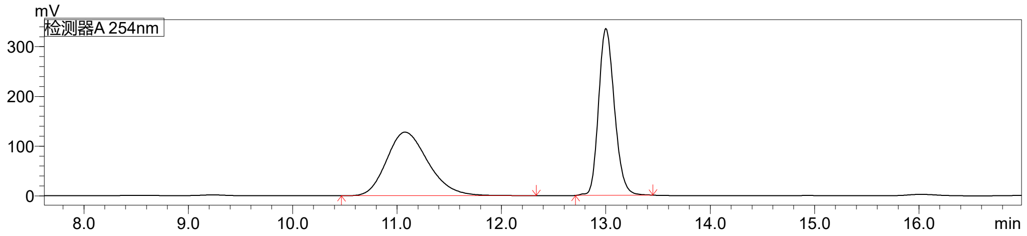

Peak#	Ret. Time	Height	Area%
1	11.077	127868	49.995
2	13.000	335688	50.005

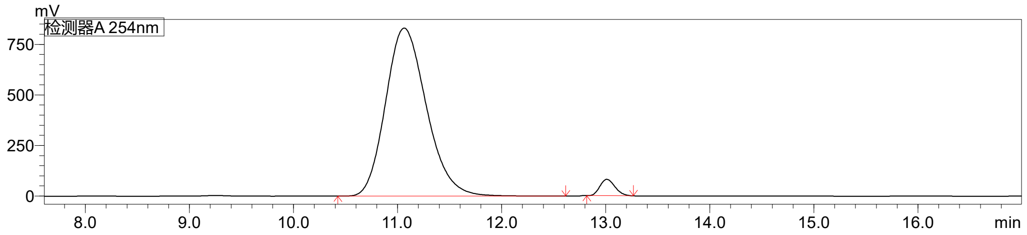

Peak#	Ret. Time	Height	Area%
1	11.063	830605	96.523
2	13.010	81172	3.477

HPLC conditions: Chiralpak AD-H, 5% iPrOH/Hx eluent, 0.5 mL/min, 254 nm

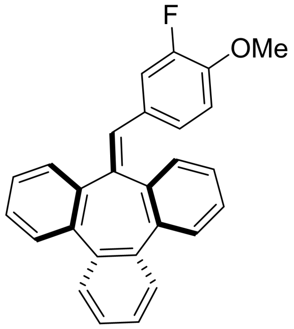

3z

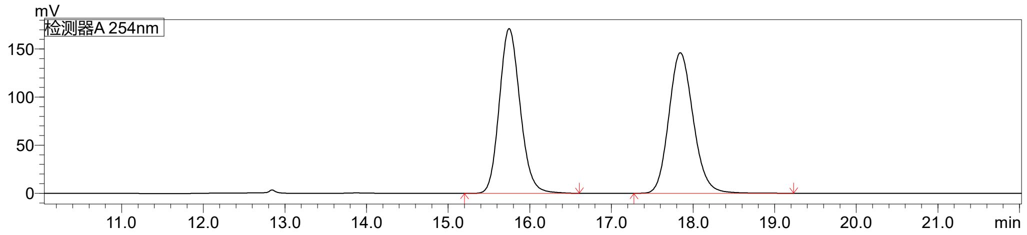

Peak#	Ret. Time	Height	Area%
1	15.747	171459	50.040
2	17.844	146199	49.960

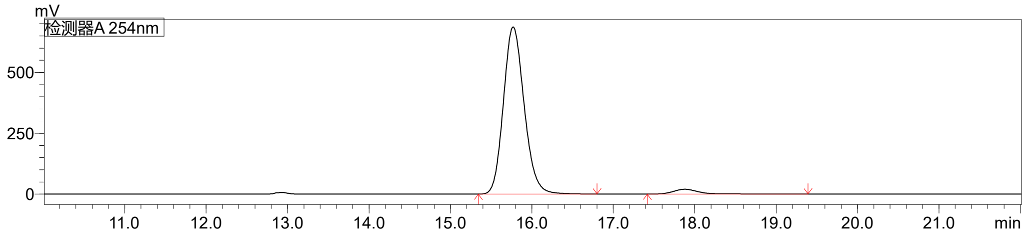

Peak#	Ret. Time	Height	Area%
1	15.770	686849	96.548

2	17.878	19805	3.452

HPLC conditions: Chiralpak AD-H, 5% iPrOH/Hx eluent, 0.5 mL/min, 254 nm

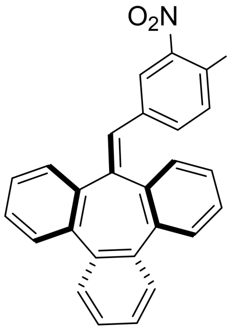

3aa

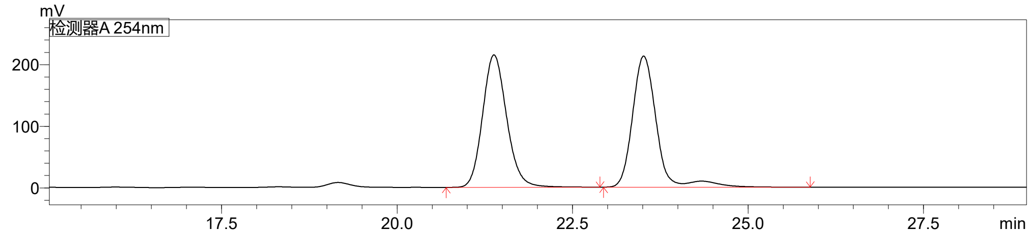

Peak#	Ret. Time	Height	Area%
1	21.380	215029	50.129
2	23.512	212890	49.871

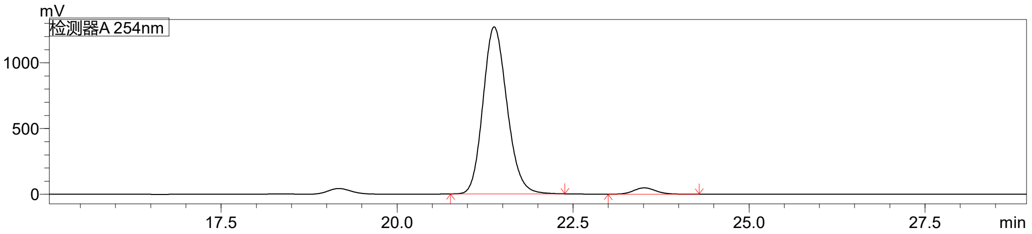

Peak#	Ret. Time	Height	Area%
1	21.378	1270732	96.594
2	23.510	48276	3.406

HPLC conditions: Chiralpak AD-H, 5% iPrOH/Hx eluent, 0.5 mL/min, 254 nm

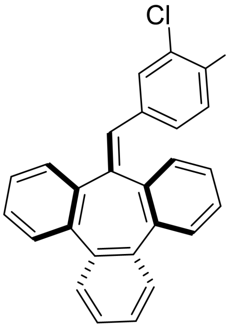

3ab

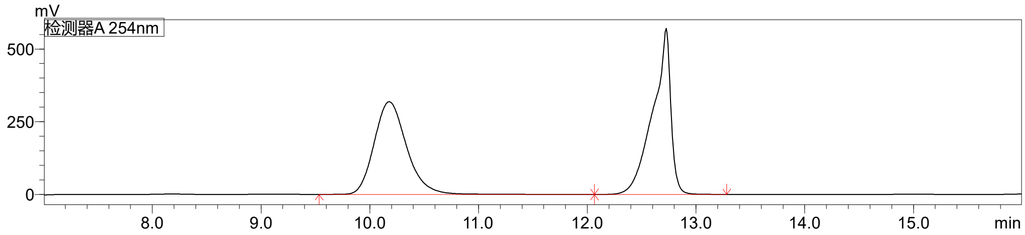

Peak#	Ret. Time	Height	Area%
1	10.178	318542	50.097
2	12.725	570811	49.903

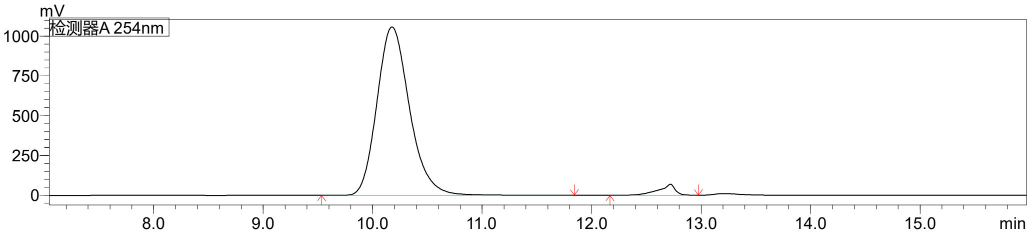

Peak#	Ret. Time	Height	Area%
1	10.176	1058318	96.724
2	12.719	69575	3.276

HPLC conditions: Chiralpak AD-H, 5% iPrOH/Hx eluent, 0.5 mL/min, 254 nm

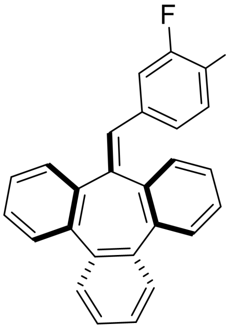

3ac

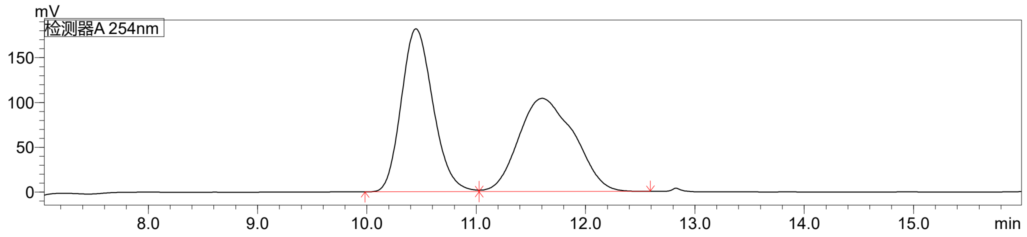

Peak#	Ret. Time	Height	Area%
1	10.449	182045	49.996
2	11.603	104081	50.004

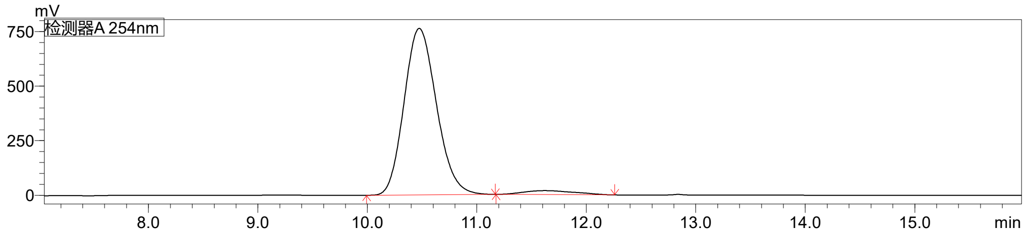

Peak#	Ret. Time	Height	Area%
1	10.473	763634	96.333
2	11.620	18294	3.667

HPLC conditions: Chiralpak AD-H, 5% iPrOH/Hx eluent, 0.5 mL/min, 254 nm

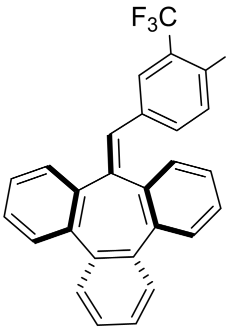

3ad

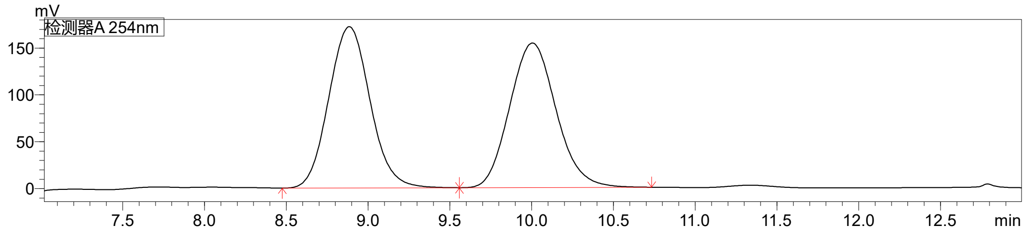

Peak#	Ret. Time	Height	Area%
1	8.885	172176	49.845

2	10.005	154211	50.155

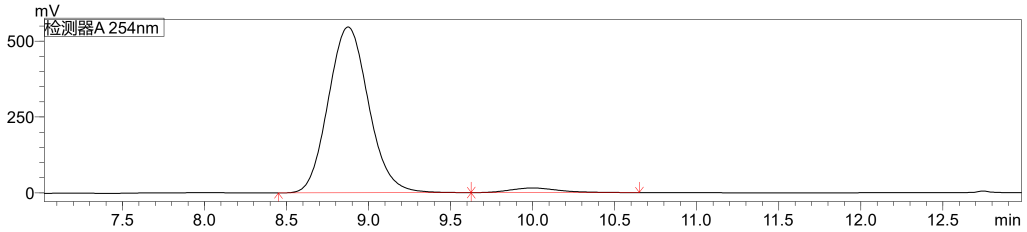

Peak#	Ret. Time	Height	Area%
1	8.875	547464	96.690
2	10.000	15450	3.310

HPLC conditions: Chiralpak OJ-H, 5% iPrOH/Hx eluent, 0.5 mL/min, 254 nm

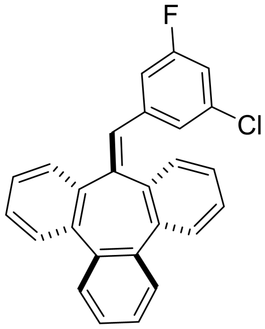

3ae

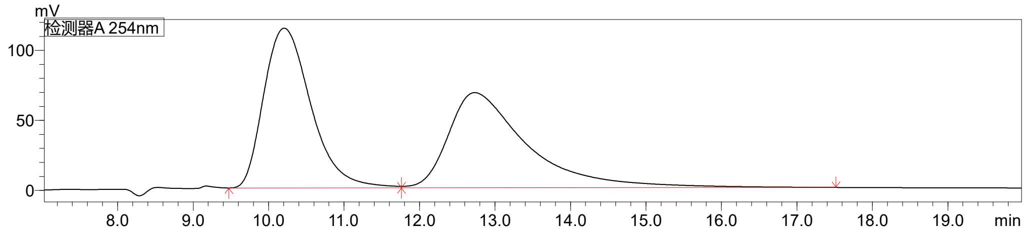

Peak#	Ret. Time	Height	Area%
1	10.206	114059	50.127
2	12.730	67940	49.873

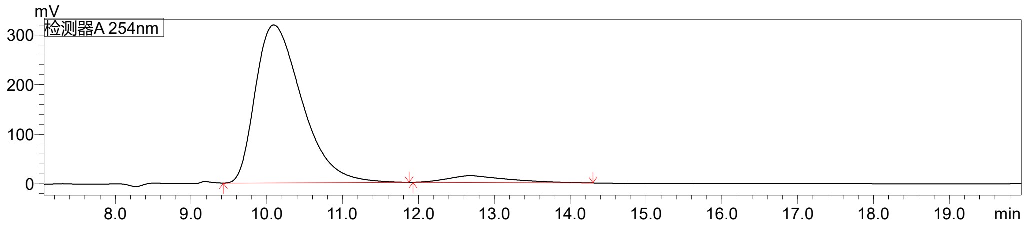

Peak#	Ret. Time	Height	Area%

1	10.090	318653	94.706
2	12.681	13843	5.294

HPLC conditions: Chiralpak OJ-H, 5% iPrOH/Hx eluent, 0.5 mL/min, 254 nm

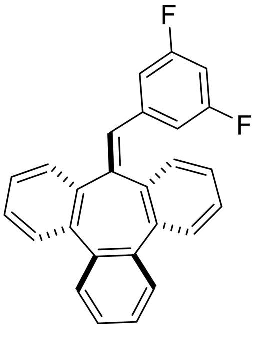

3af

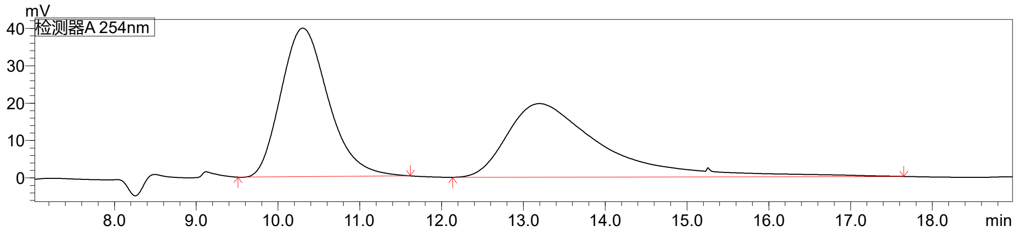

Peak#	Ret. Time	Height	Area%
1	10.302	39732	50.188
2	13.195	19659	49.812

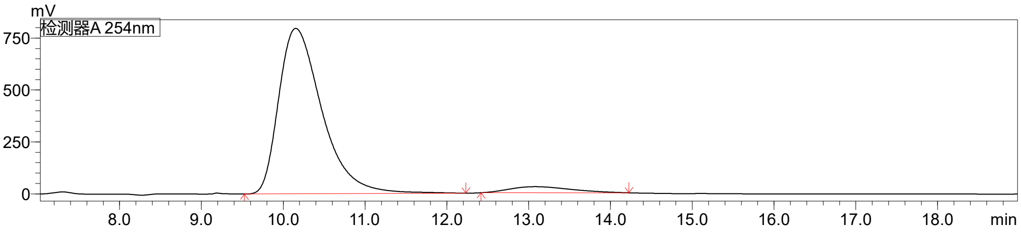

Peak#	Ret. Time	Height	Area%
1	10.153	797068	94.980
2	13.078	29451	5.020

HPLC conditions: Chiralpak OJ-H, 5% iPrOH/Hx eluent, 0.5 mL/min, 254 nm

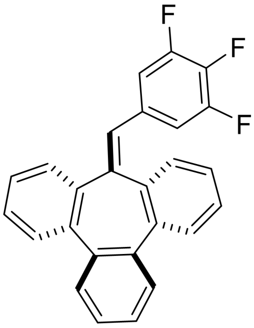

3ag

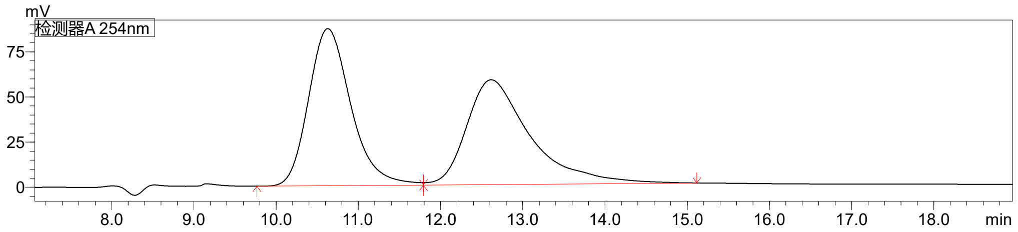

Peak#	Ret. Time	Height	Area%
1	10.628	87012	49.956
2	12.615	58044	50.044

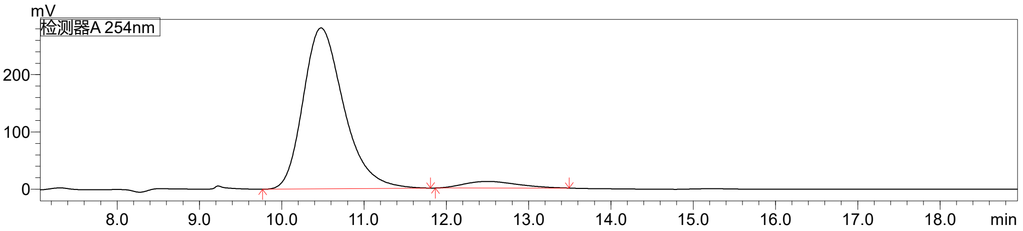

Peak#	Ret. Time	Height	Area%
1	10.477	281034	94.820
2	12.501	11563	5.180

HPLC conditions: Chiralpak AD-H, 5% iPrOH/Hx eluent, 0.5 mL/min, 254 nm

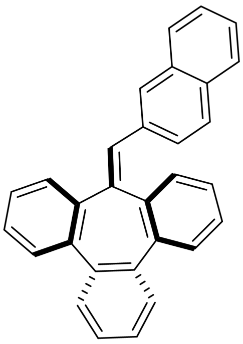

3ah

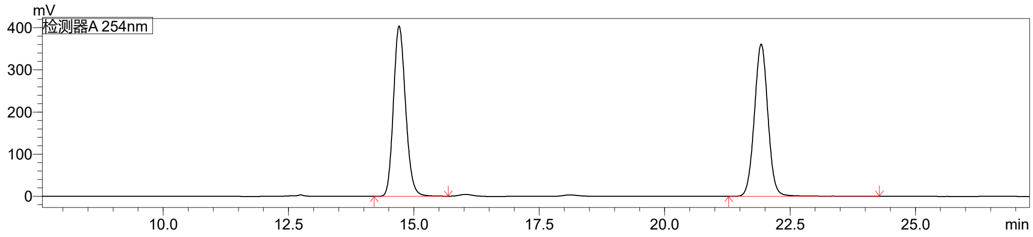

Peak#	Ret. Time	Height	Area%
1	14.705	404364	50.005
2	21.924	361527	49.995

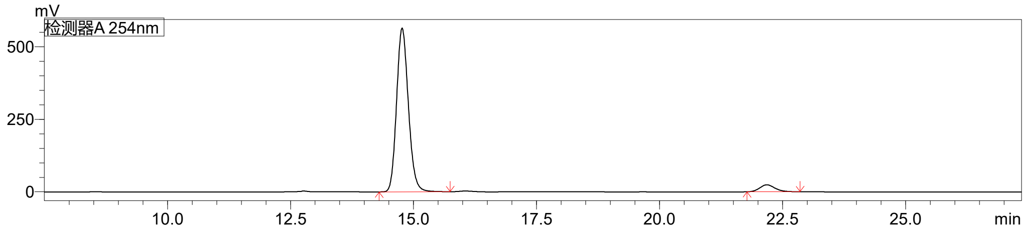

Peak#	Ret. Time	Height	Area%
1	14.767	564659	94.767
2	22.177	24073	5.233

HPLC conditions: Chiralpak AD-H, 10% iPrOH/Hx eluent, 0.5 mL/min, 254 nm

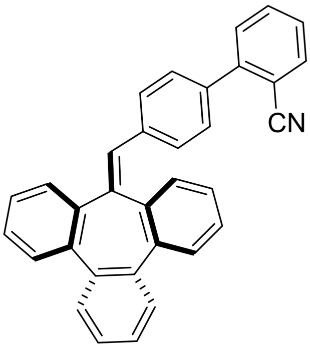

3ai

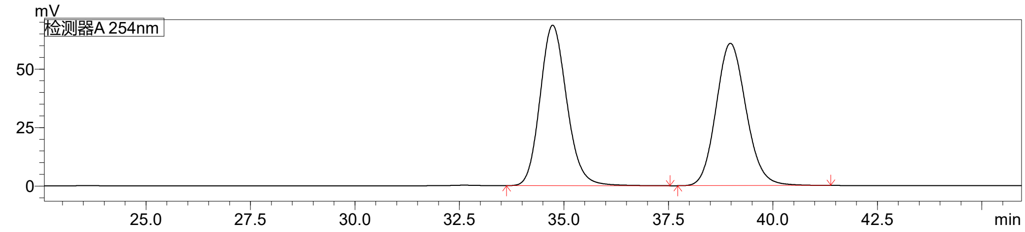

Peak#	Ret. Time	Height	Area%

1	34.730	68585	50.096
2	38.982	60824	49.904

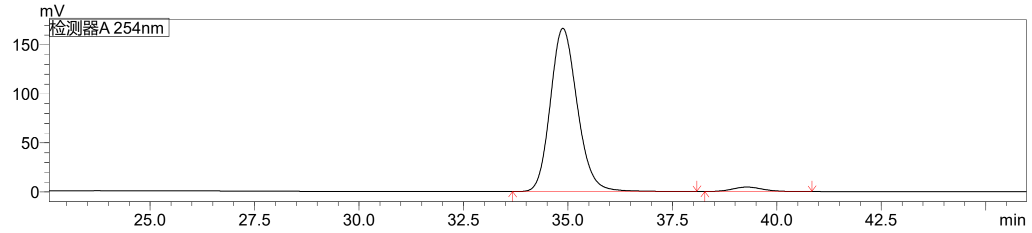

Peak#	Ret. Time	Height	Area%
1	34.877	166585	97.089
2	39.282	4515	2.911

HPLC conditions: Chiralpak AD-H, 5% iPrOH/Hx eluent, 0.5 mL/min, 254 nm

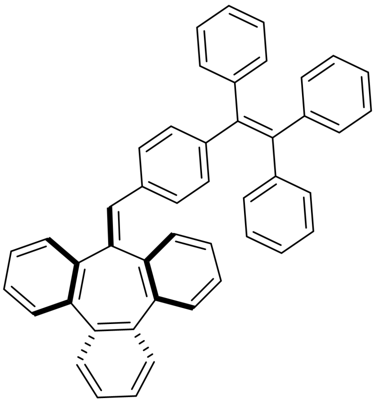

3aj

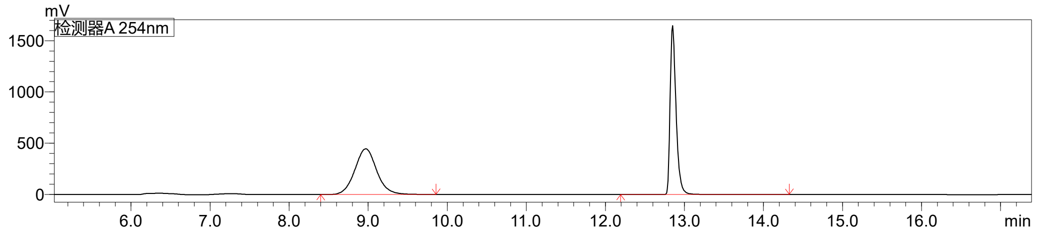

Peak#	Ret. Time	Height	Area%
1	8.968	446325	50.002
2	12.850	1648686	49.998

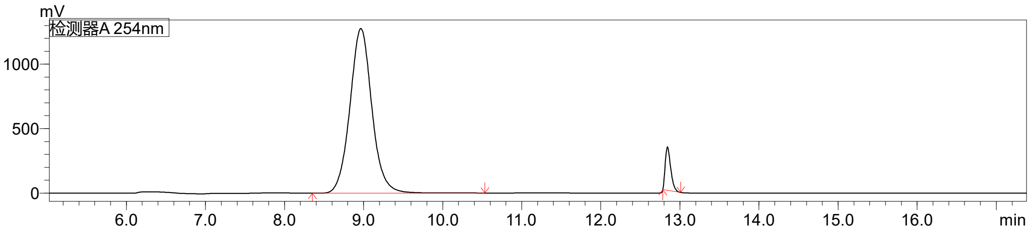

Peak#	Ret. Time	Height	Area%
1	8.965	1277160	93.858
2	12.842	337482	6.142

HPLC conditions: Chiralpak AD-H, 10% iPrOH/Hx eluent, 0.5 mL/min, 254 nm

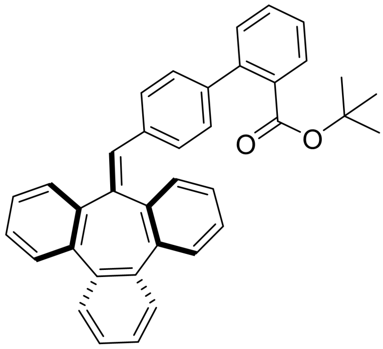

3ak

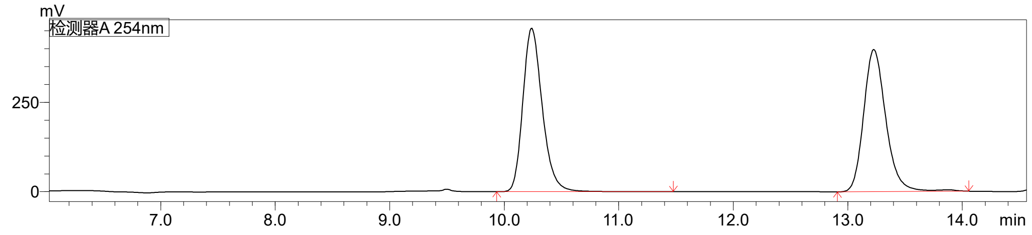

Peak#	Ret. Time	Height	Area%
1	10.239	457007	50.077
2	13.227	397506	49.923

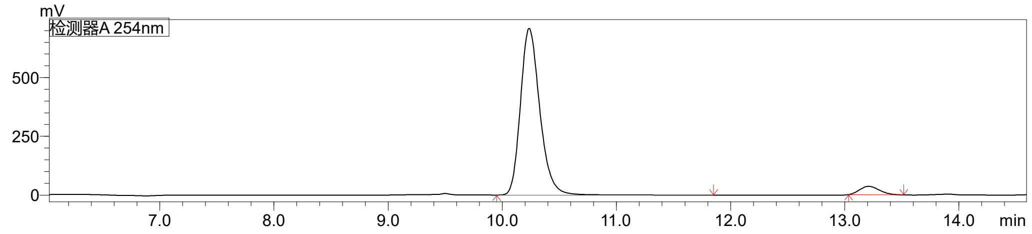

Peak#	Ret. Time	Height	Area%
1	10.235	709931	95.089

2	13.209	35678	4.911

HPLC conditions: Chiralpak AD-H, 10% iPrOH/Hx eluent, 0.5 mL/min, 254 nm

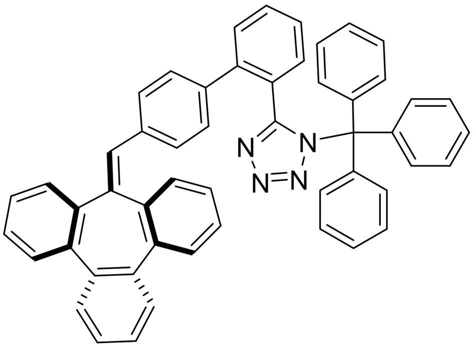

3al

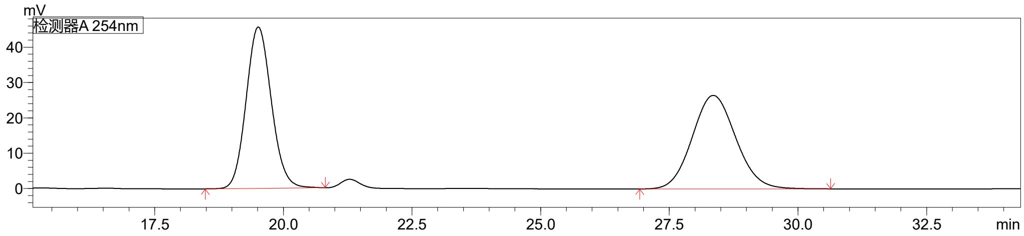

Peak#	Ret. Time	Height	Area%
1	19.512	45692	49.948
2	28.355	26453	50.052

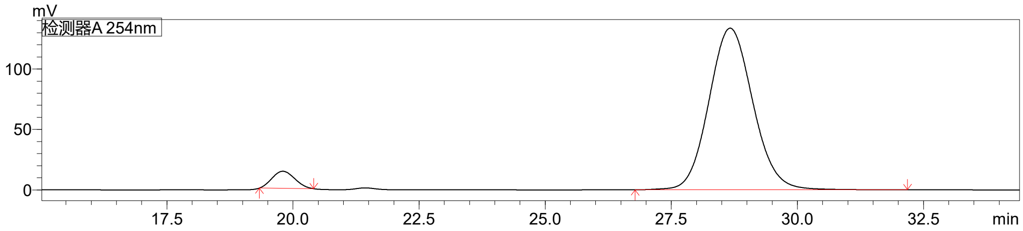

Peak#	Ret. Time	Height	Area%
1	19.797	14143	5.151
2	28.666	133749	94.849

HPLC conditions: Chiralpak AD-H, 10% iPrOH/Hx eluent, 0.5 mL/min, 254 nm

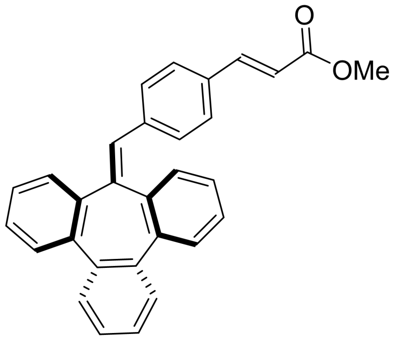

3am

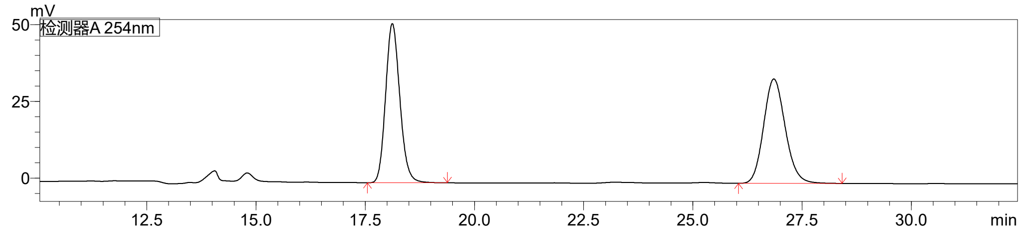

Peak#	Ret. Time	Height	Area%
1	18.120	51835	50.116
2	26.853	34008	49.884

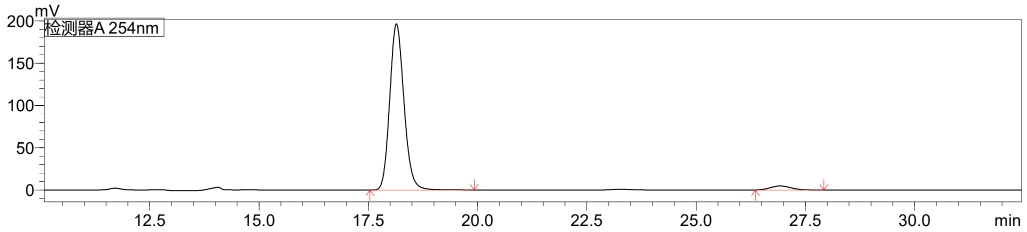

Peak#	Ret. Time	Height	Area%
1	18.143	196584	96.558
2	26.923	4784	3.442

HPLC conditions: Chiralpak AD-H, 10% iPrOH/Hx eluent, 0.5 mL/min, 254 nm

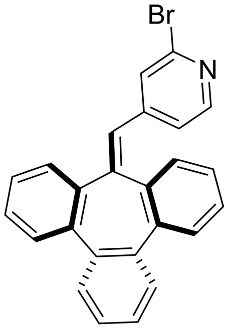

3an

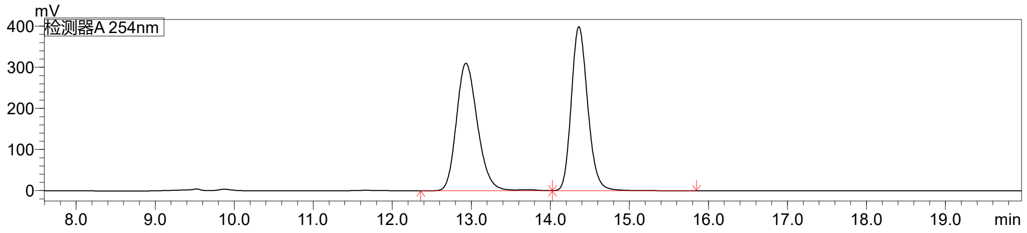

Peak#	Ret. Time	Height	Area%
1	12.932	310406	49.956
2	14.360	399218	50.044

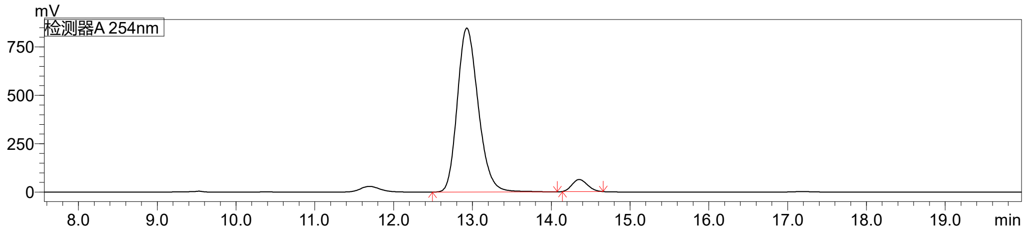

Peak#	Ret. Time	Height	Area%
1	12.928	847335	94.887
2	14.355	62248	5.113

HPLC conditions: Chiralpak AD-H, 5% iPrOH/Hx eluent, 0.5 mL/min, 254 nm

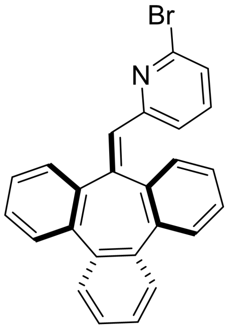

3ao

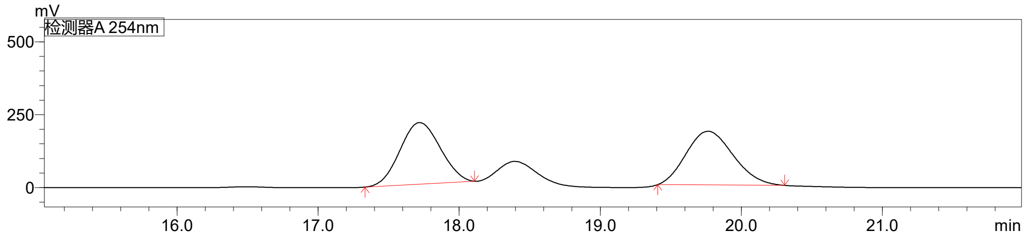

Peak#	Ret. Time	Height	Area%
1	17.720	211508	49.562

2	19.764	184091	50.438

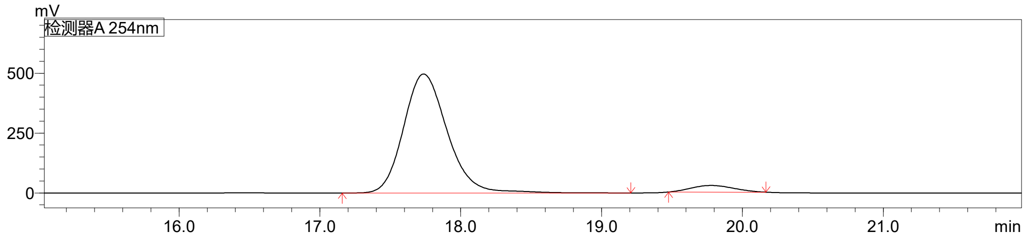

Peak#	Ret. Time	Height	Area%
1	17.736	496701	94.815
2	19.777	27723	5.185

HPLC conditions: Chiralpak OX-H, 5% iPrOH/Hx eluent, 0.5 mL/min, 254 nm

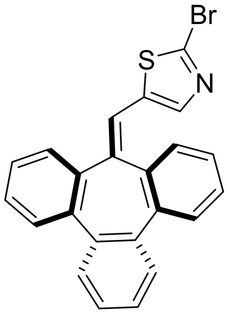

3ap

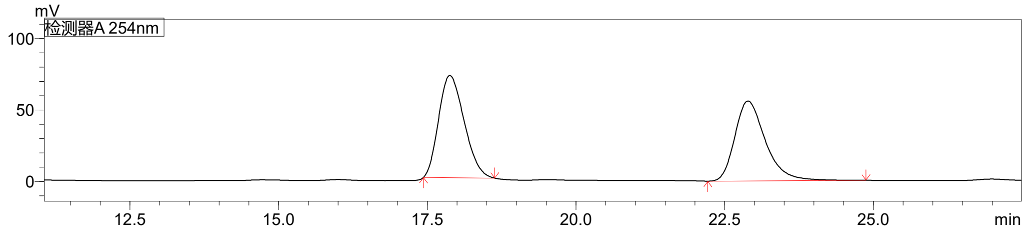

Peak#	Ret. Time	Height	Area%
1	17.878	71465	51.608
2	22.892	55919	48.392

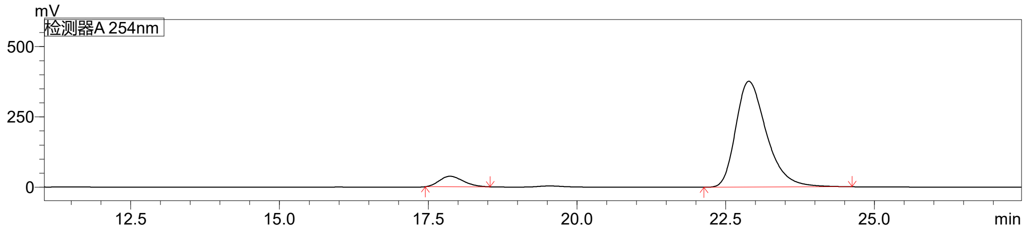

Peak#	Ret. Time	Height	Area%

1	17.867	37370	7.137
2	22.891	376860	92.863

HPLC conditions: Chiralpak AD-H, 5% iPrOH/Hx eluent, 0.5 mL/min, 254 nm

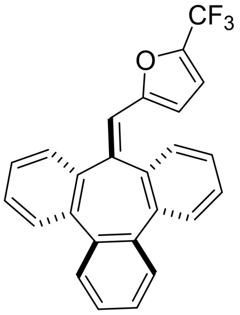

3aq

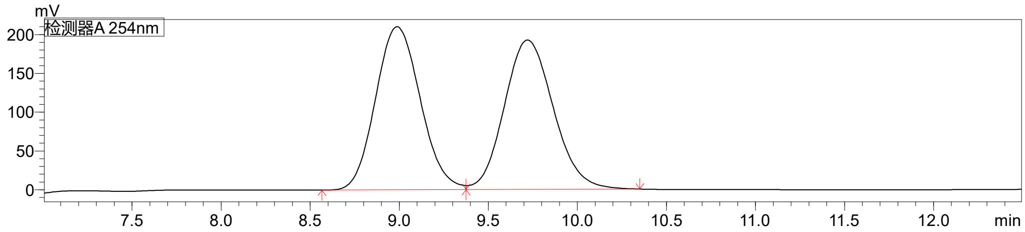

Peak#	Ret. Time	Height	Area%
1	8.988	210129	49.906
2	9.720	192516	50.094

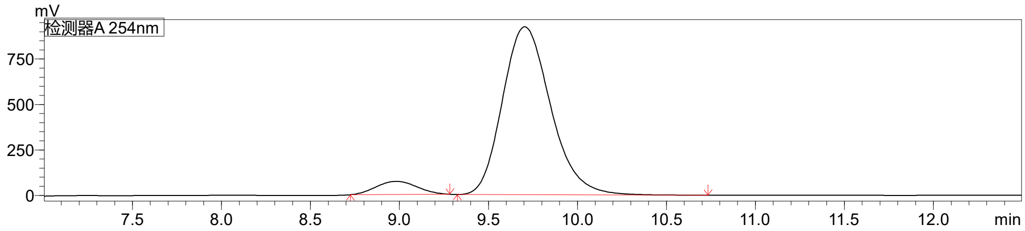

Peak#	Ret. Time	Height	Area%
1	8.980	72579	6.254
2	9.703	923561	93.746

HPLC conditions: Chiralpak AD-H, 5% iPrOH/Hx eluent, 0.5 mL/min, 254 nm

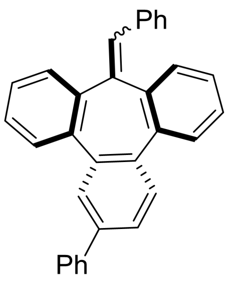

3ar

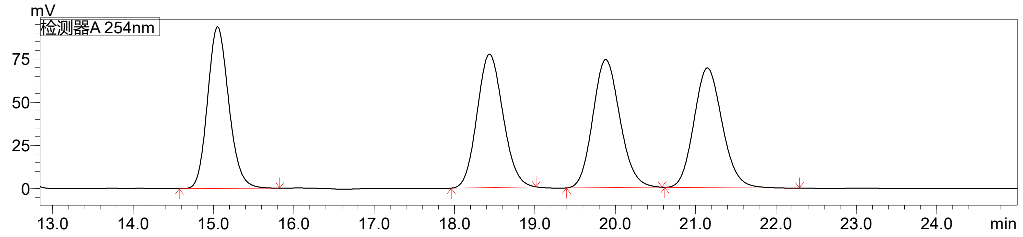

Peak#	Ret. Time	Height	Area%
1	15.051	93489	24.935
2	18.435	77190	25.184
3	19.882	74064	25.275
4	21.147	69149	24.606

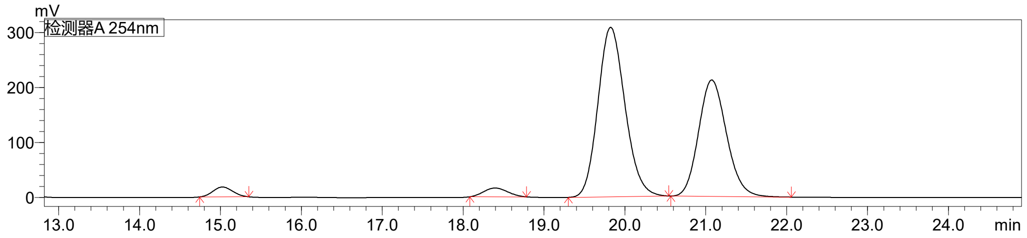

Peak#	Ret. Time	Height	Area%
1	15.024	17990	2.354
2	18.396	15986	2.526
3	19.823	308495	55.681
4	21.073	211970	39.439

HPLC conditions: Chiralpak AD-H, 5% iPrOH/Hx eluent, 0.5 mL/min, 254 nm

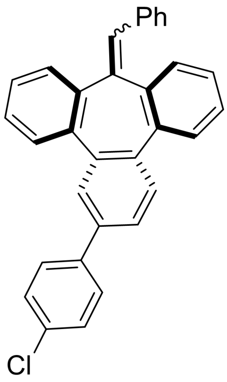

3as

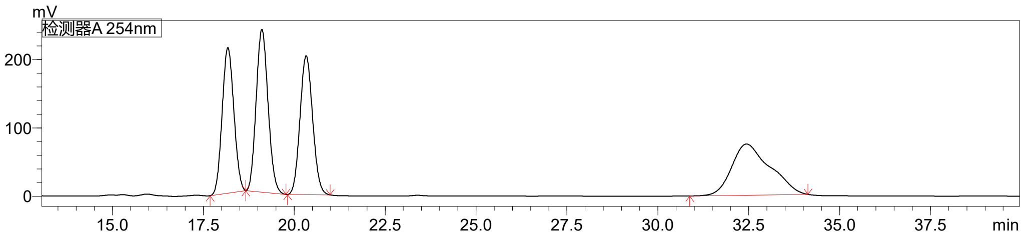

Peak#	Ret. Time	Height	Area%
1	18.173	213040	23.177
2	19.109	238398	26.392
3	20.326	203362	23.868
4	32.440	74878	26.562

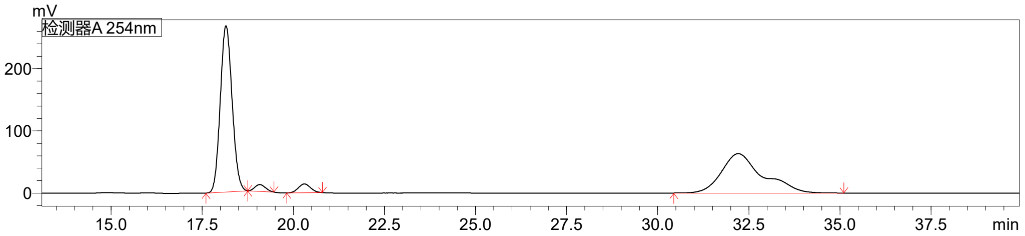

Peak#	Ret. Time	Height	Area%
1	18.152	267115	52.232
2	19.079	10899	1.901
3	20.303	14165	2.889
4	32.208	63267	42.978

HPLC conditions: Chiralpak AD-H, 5% iPrOH/Hx eluent, 0.5 mL/min, 254 nm

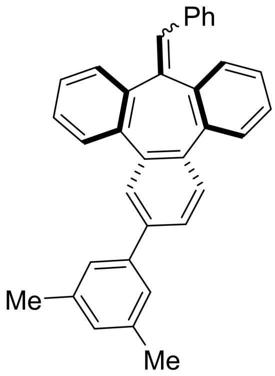

3at

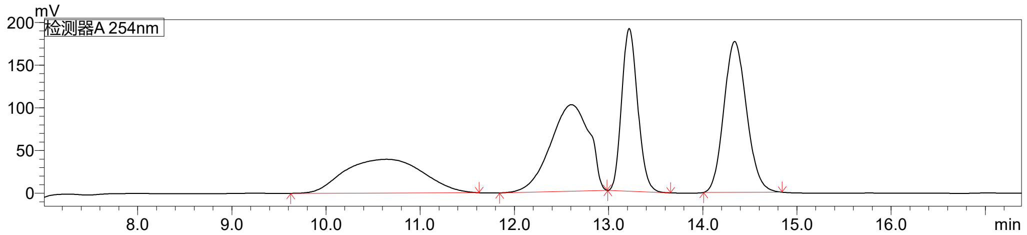

Peak#	Ret. Time	Height	Area%
1	10.642	39490	21.972
2	12.605	101285	27.879
3	13.218	190579	21.615
4	14.338	176567	28.534

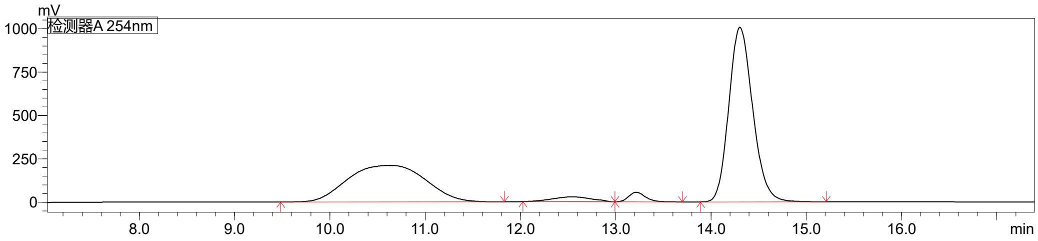

Peak#	Ret. Time	Height	Area%
1	10.630	210365	38.478
2	12.544	27643	2.561
3	13.216	55249	2.171
4	14.303	1005614	56.790

HPLC conditions: Chiralpak AD-H, 5% iPrOH/Hx eluent, 0.5 mL/min, 254 nm

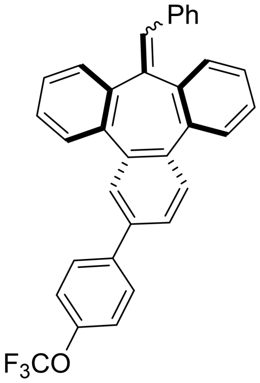

3au

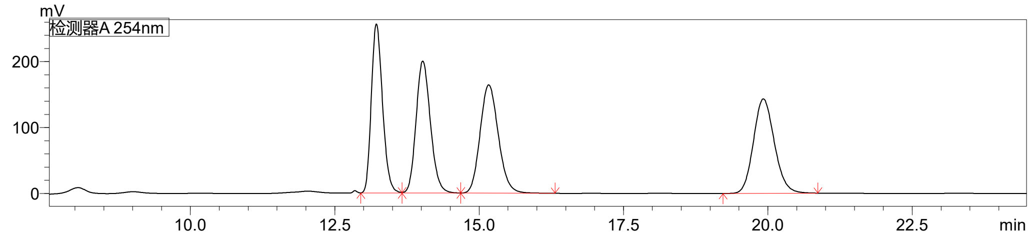

Peak#	Ret. Time	Height	Area%
1	13.220	256175	24.481
2	14.023	200252	24.490
3	15.165	164438	25.516
4	19.921	143261	25.514

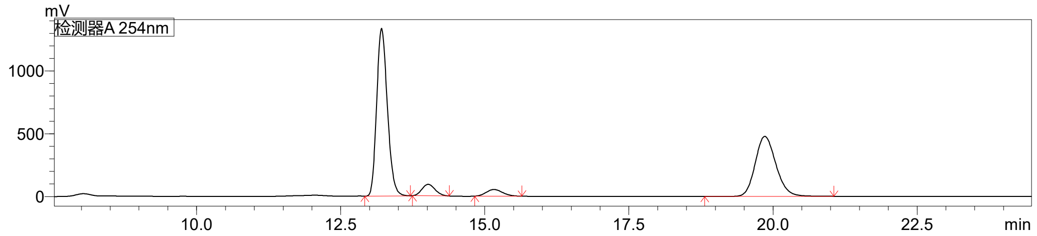

Peak#	Ret. Time	Height	Area%
1	13.208	1336340	54.438
2	14.015	92130	4.615
3	15.157	53458	3.343
4	19.857	477655	37.604

HPLC conditions: Chiralpak OD-H, 5% ^{i}PrOH/Hx eluent, 0.5 mL/min, 254 nm

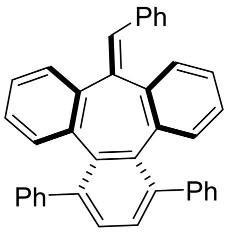

3av

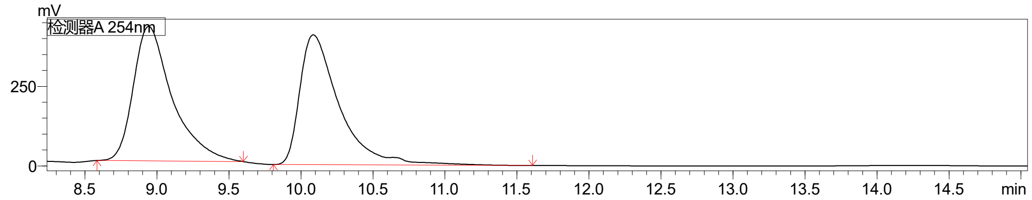

Peak#	Ret. Time	Height	Area%
1	8.942	422641	50.374
2	10.085	408433	49.626

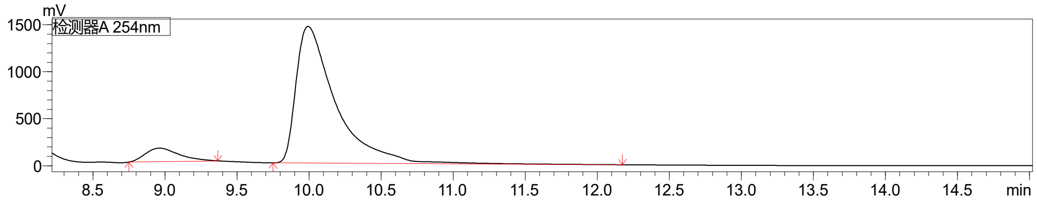

Peak#	Ret. Time	Height	Area%
1	8.960	143952	7.761
2	9.992	1455658	92.239

HPLC conditions: Chiralpak AD-H, 5% ^{i}PrOH/Hx eluent, 0.5 mL/min, 254 nm

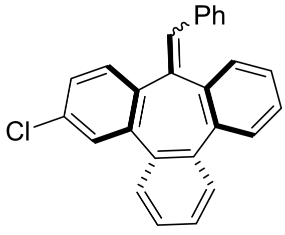

3aw

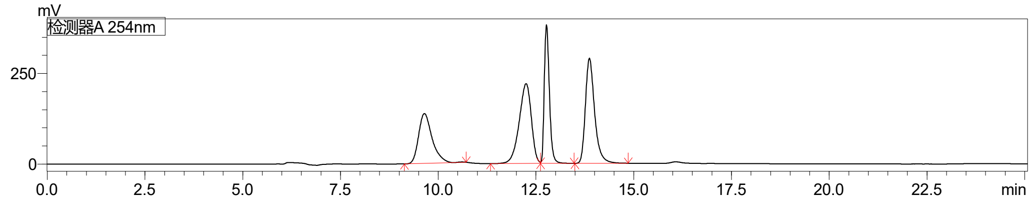

Peak#	Ret. Time	Height	Area%
1	9.646	137395	20.344
2	12.247	220331	29.205
3	12.769	382350	21.308
4	13.866	289916	29.143

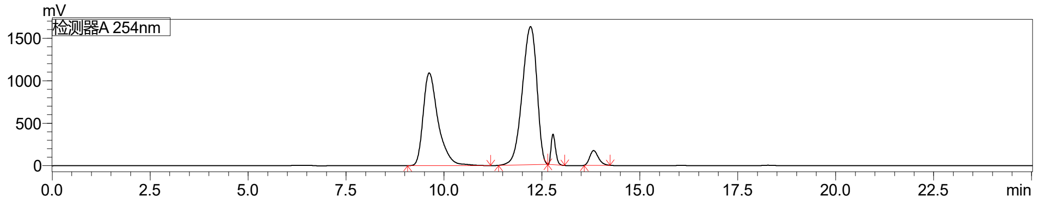

Peak#	Ret. Time	Height	Area%
1	9.622	1090263	37.909
2	12.209	1625441	54.856
3	12.782	359020	3.735
4	13.819	173631	3.500

HPLC conditions: Chiralpak IA-H, 5% iPrOH/Hx eluent, 0.5 mL/min, 254 nm

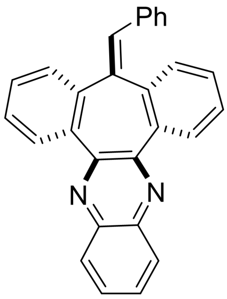

3ax

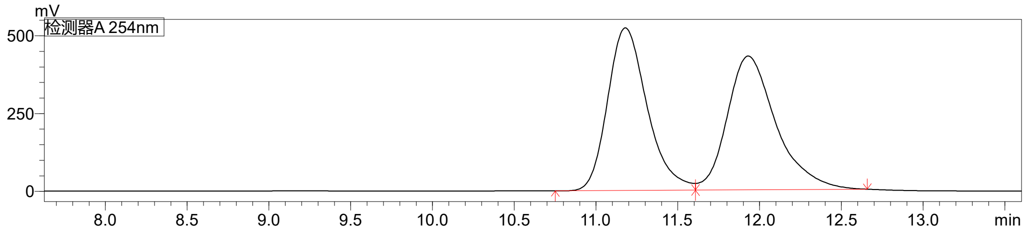

Peak#	Ret. Time	Height	Area%
1	11.179	522641	49.665
2	11.930	429947	50.335

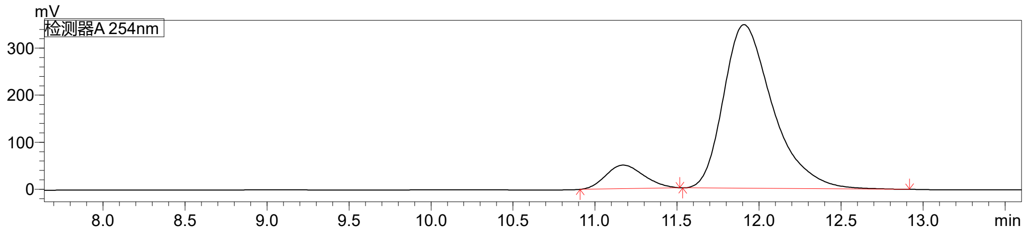

Peak#	Ret. Time	Height	Area%
1	11.171	50221	9.997
2	11.908	347953	90.003

HPLC conditions: Chiralpak OJ-H, 30% iPrOH/Hx eluent, 0.5 mL/min, 254 nm

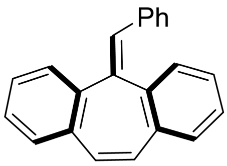

3ay

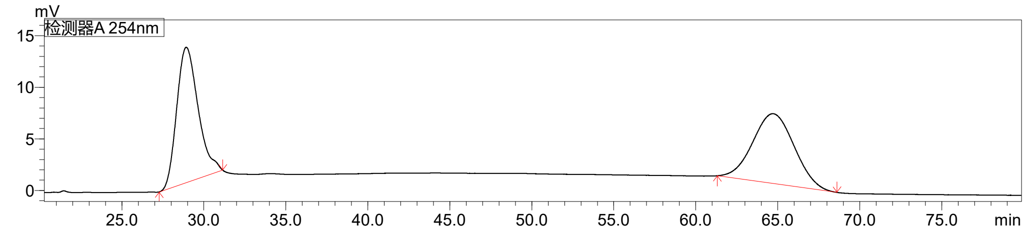

Peak#	Ret. Time	Height	Area%
1	28.924	13130	49.982
2	64.695	6769	50.018

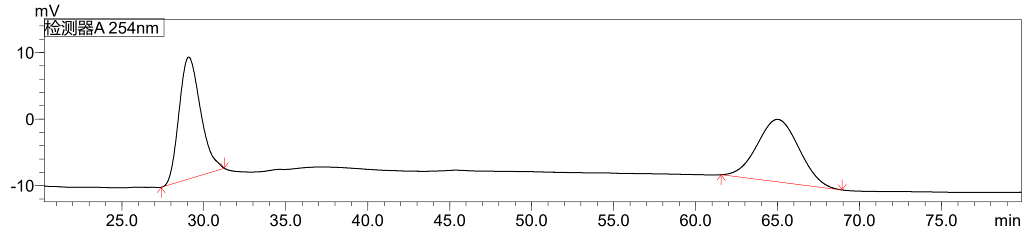

Peak#	Ret. Time	Height	Area%
1	29.076	18316	49.955
2	64.971	9379	50.045

8. Synthetic transformations

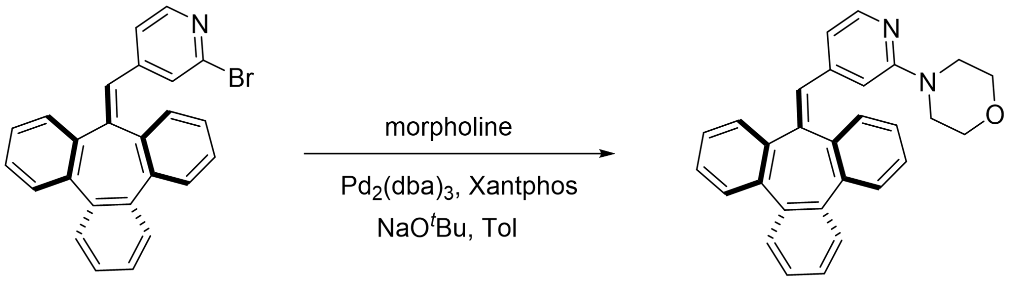

3al **4**

Under N_2 atmosphere, morpholine (0.2 mmol, 1.0 equiv.) and **3al** (0.26 mmol, 1.3 equiv.) were combined in the reaction flask. Dry toluene (2 mL) was added, followed by NaO^{t}Bu (3.0 mmol, 1.5 equiv.), $Pd_2(dba)_3$ (0.004 mmol, 0.02 equiv.) and Xantphos (0.012 mmol, 0.06 equiv.). The mixture was heated to 100 °C for 12 hours. After the reaction was completed, it was cooled down to room temperature and diluted with EtOAc (10 mL). It was then washed with water (5 mL). The organic solution was concentrated under reduced pressure. The crude product was purified by flash column chromatography (silica gel, petroleum ether: EtOAc = 5:1) to provided **4** (68.3 mg, 82%, 90% ee) as a white solid.

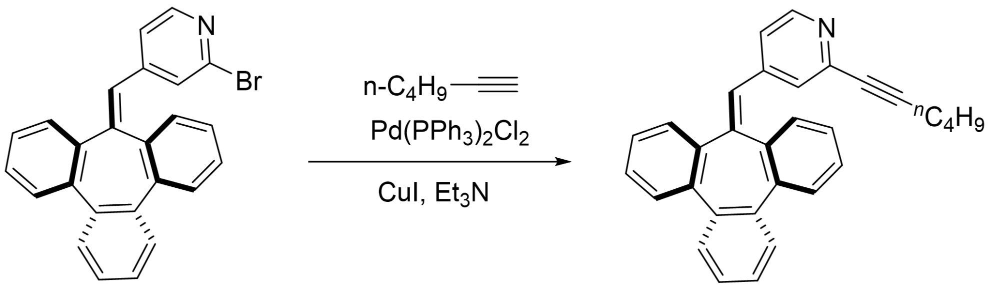

3al **5**

Under nitrogen atmosphere, **3al** (0.2 mmol, 1.0 equiv.), $Pd(PPh_3)_2Cl_2$ (0.05 equiv.) and CuI (0.1 equiv.) were dissolved in Et_3N (8 mL), then 1-hexyne (0.24 mmol, 1.2 equiv.) were added to the solution. The mixture is stirred at ambient temperature overnight. Then, water is added and the resulting mixture is extracted with diethyl ether. The combined organic extracts are washed with brine and dried ($MgSO_4$). The solvent is removed under reduced pressure and the residue is purified by chromatography on silica gel (petroleum ether: EtOAc = 10:1) to give the product **5** (69.9 mg, 85%, 90% ee) as a yellow oil.

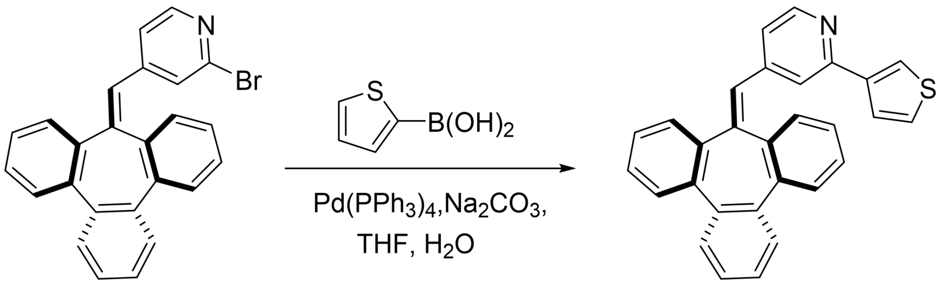

Under nitrogen atmosphere, a mixture of **3al** (0.2 mmol, 1.0 equiv.), thiophen-3-ylboronic acid (0.22 mmol, 1.1 equiv.), $Pd(PPh_3)_4$ (0.03 equiv.), Na_2CO_3 (5 equiv.) in dry THF (0.7 mL) and water (0.7 mL) was stirred at 60 °C using oil bath for 24 hours. After the reaction was completed, cool the mixture to room temperature, pour the mixture into water and extract the mixture with DCM. The combined organic layers were dried with anhydrous Na_2SO_4, filtered and concentrated. The residue was purified by a silica gel flash chromatography (petroleum ether: EtOAc = 10:1) to give the product **6** (59.5 mg, 72%, 90% ee) as a white solid.

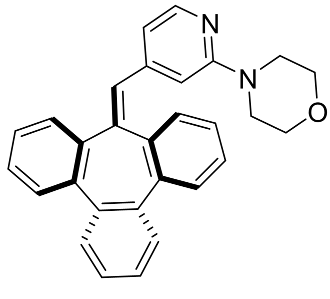

4

4-(4-((9H-tribenzo[a,c,e][7]annulen-9-ylidene)methyl)pyridin-2-yl)morpholine (4)

^{1}H NMR (400 MHz, $CDCl_3$) δ 7.98 (d, J = 5.2 Hz, 1H), 7.74 – 7.69 (m, 1H), 7.67 (dd, J = 7.8, 1.2 Hz, 1H), 7.64 – 7.58 (m, 2H), 7.51 – 7.44 (m, 3H), 7.42 – 7.38 (m, 2H), 7.36 (dd, J = 7.7, 1.4 Hz, 1H), 7.23 (dd, J = 7.5, 1.2 Hz, 1H), 7.13 (dd, J = 7.6, 1.3 Hz, 1H), 6.47 (s, 1H), 6.34 (d, J = 5.3 Hz, 1H), 6.28 (s, 1H), 3.74 (d, J = 2.0 Hz, 4H), 3.35 – 3.28 (m, 2H), 3.27 – 3.20 (m, 2H).

^{13}C NMR (150 MHz, $CDCl_3$) δ 159.4, 147.4, 146.4, 145.5, 145.5, 141.7, 138.4, 138.4, 137.4, 137.0, 130.5, 129.7, 129.4, 128.9, 128.0, 128.0, 127.9, 127.9, 127.7, 127.7, 127.3, 127.0, 125.6, 114.5, 107.4, 66.6, 53.4, 45.6.

HRMS: (ESI) m/z: $[M+H]^+$ Calcd for $C_{29}H_{25}N_2O$ 417.1961; Found 417.1969.

Optical $[\alpha]_{25}^{D}$ = +26.7 ° (c = 0.25, CH_2Cl_2, 90% ee)

HPLC (AS-H, ^{i}PrOH/n-hexane = 15/85, flow rate = 0.5 mL/min, l = 254 nm) t_R = 14.7 min (major), 32.3 min (minor).

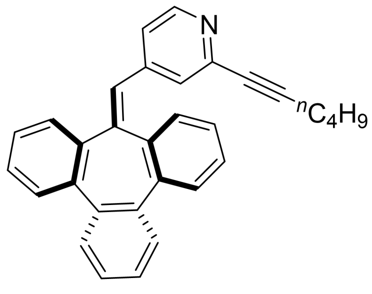

5

4-((9H-tribenzo[a,c,e][7]annulen-9-ylidene)methyl)-2-(hex-1-yn-1-yl)pyridine (5)

^{1}H NMR (400 MHz, $CDCl_3$) δ 8.22 (d, *J* = 5.0 Hz, 1H), 7.76 – 7.73 (m, 1H), 7.67 (dd, *J* = 7.9, 1.2 Hz, 1H), 7.63 – 7.59 (m, 2H), 7.51 – 7.46 (m, 2H), 7.43 – 7.36 (m, 4H), 7.25 – 7.20 (m, 1H), 7.10 – 7.04 (m, 2H), 6.69 (d, *J* = 5.3 Hz, 1H), 6.46 (s, 1H), 2.41 (s, 2H), 1.59 (d, *J* = 7.4 Hz, 2H), 1.48 (d, *J* = 7.3 Hz, 2H), 0.96 (t, *J* = 7.4 Hz, 3H).

^{13}C NMR (150 MHz, $CDCl_3$) δ 149.2, 147.6, 145.2, 144.3, 143.7, 140.9, 138.3, 137.3, 136.9, 130.4, 129.9, 129.7, 128.9, 128.2, 128.1, 128.0, 127.7, 126.9, 126.8, 126.0, 125.6, 121.8, 90.7, 80.3, 30.3, 21.9, 18.9, 13.6.

HRMS: (ESI) m/z: $[M+Na]^+$ Calcd for $C_{31}H_{25}NNa$ 434.1879; Found 434.1890.

Optical $[\alpha]_{25}^{D}$ = -232.3 ° (c = 0.25, CH_2Cl_2, 90% ee)

HPLC (IA-H, ^{i}PrOH/n-hexane = 10/90, flow rate = 0.5 mL/min, l = 254 nm) t_R = 12.0 min (major), 13.1 min (minor).

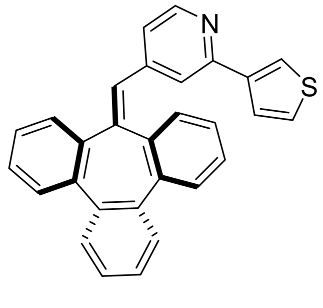

6

4-((9H-tribenzo[a,c,e][7]annulen-9-ylidene)methyl)-2-(thiophen-3-yl)pyridine (6)

^{1}H NMR (400 MHz, $CDCl_3$) δ 8.36 (s, 1H), 7.77 – 7.74 (m, 1H), 7.71 (d, *J* = 7.8 Hz, 1H), 7.65 – 7.59 (m, 3H), 7.47 (dd, *J* = 5.9, 3.1 Hz, 3H), 7.41 (dd, *J* = 5.6, 3.3 Hz, 3H),

7.33 (d, J = 13.5 Hz, 2H), 7.24 (s, 1H), 7.22 (s, 1H), 7.12 (d, J = 7.6 Hz, 1H), 6.79 (d, J = 5.3 Hz, 1H), 6.53 (s, 1H).

^{13}C NMR (150 MHz, $CDCl_3$) δ 153.0, 149.3, 147.4, 145.3, 144.8, 142.0, 141.4, 138.4, 138.3, 137.5, 137.0, 130.6, 129.9, 129.7, 129.0, 128.3, 128.2, 128.1, 128.1, 127.8, 127.8, 126.9, 126.5, 126.1, 126.0, 125.6, 123.3, 121.9, 120.3.

HRMS: (ESI) m/z: $[M+H]^+$ Calcd for $C_{29}H_{20}NS$ 414.1311; Found 414.1307.

Optical $[\alpha]_{25}^{D}$ = -361.7 ° (c = 0.25, CH_2Cl_2, 90% ee)

HPLC (AD-H, ^{i}PrOH/n-hexane = 10/90, flow rate = 0.5 mL/min, l = 254 nm) t_R = 15.2 min (major), 18.1 min (minor).

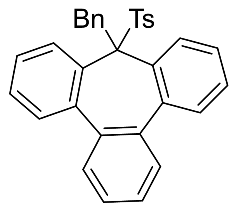

7

9-benzyl-9-tosyl-9H-tribenzo[a,c,e][7]annulene (7)

^{1}H NMR (400 MHz, $CDCl_3$) δ 7.7 (d, J = 8.3 Hz, 2H), 7.6 (dd, J = 5.8, 3.4 Hz, 1H), 7.6 – 7.5 (m, 2H), 7.5 – 7.4 (m, 3H), 7.4 – 7.4 (m, 2H), 7.4 – 7.3 (m, 2H), 7.3 (d, J = 1.5 Hz, 1H), 7.3 (s, 1H), 7.3 (s, 1H), 7.2 (dd, J = 7.7, 1.5 Hz, 1H), 7.1 (d, J = 7.4 Hz, 1H), 6.9 (t, J = 7.7 Hz, 2H), 6.8 (dd, J = 7.9, 1.5 Hz, 2H), 4.4 (d, J = 13.9 Hz, 1H), 3.7 (d, J = 14.0 Hz, 1H), 2.4 (s, 3H).

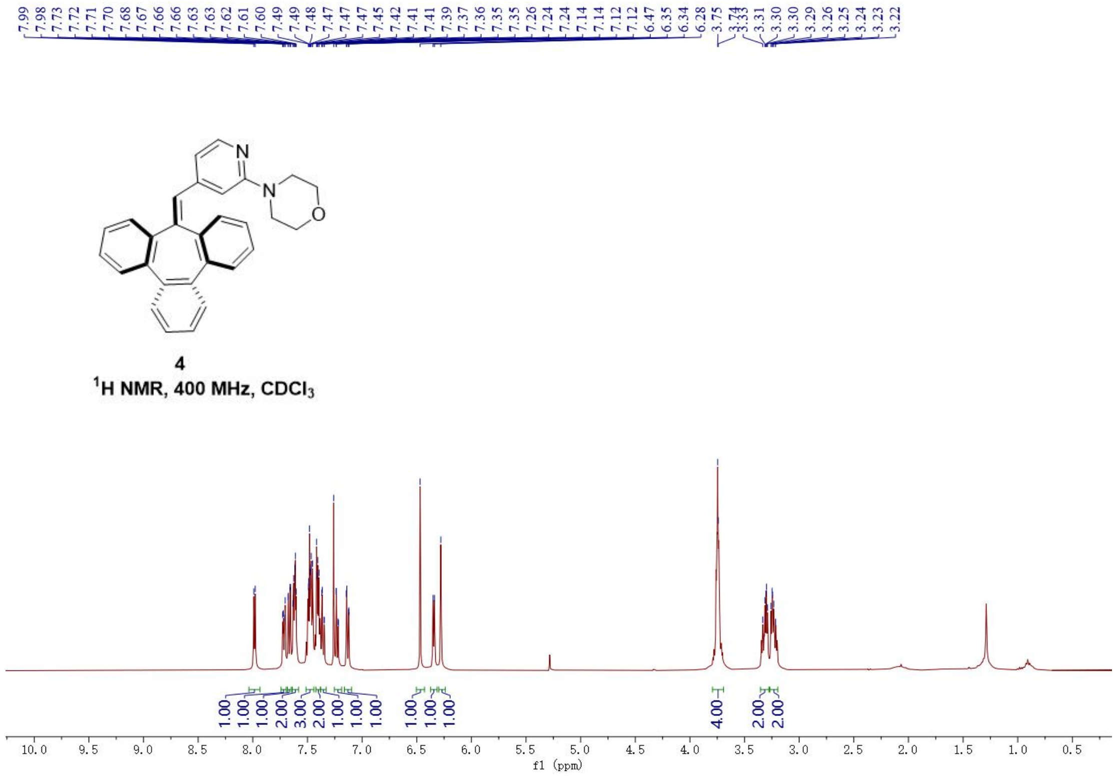
7.99 7.98 7.73 7.72 7.71 7.70 7.68 7.67 7.66 7.66 7.63 7.63 7.62 7.61 7.60 7.49 7.49 7.48 7.47 7.47 7.47 7.45 7.42 7.41 7.41 7.39 7.37 7.36 7.35 7.35 7.26 7.24 7.24 7.14 7.12 7.12 6.47 6.35 6.34 6.28 3.75 3.74 3.33 3.31 3.30 3.29 3.26 3.25 3.24 3.23 3.22
4
^{1}H NMR, 400 MHz, CDCl$_3$
1.00 1.00 1.00 2.00 3.00 2.00 1.00 1.00 1.00 1.00 1.00 1.00 4.00 2.00 2.00
f1 (ppm)

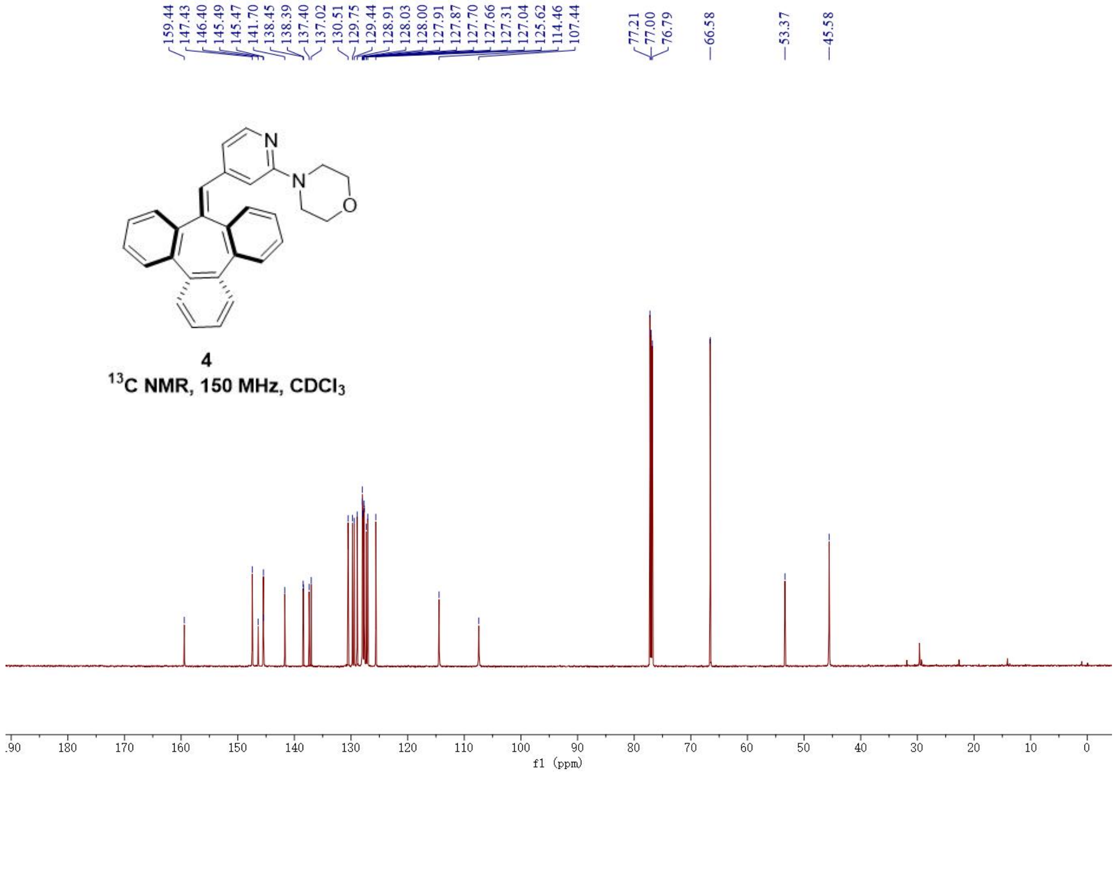
159.44 147.43 146.40 145.49 145.47 141.70 138.45 138.39 137.40 137.02 130.51 129.75 129.44 128.91 128.03 128.00 127.91 127.87 127.70 127.66 127.31 127.04 125.62 114.46 107.44 77.21 77.00 76.79 66.58 53.37 45.58
4
^{13}C NMR, 150 MHz, CDCl$_3$
f1 (ppm)

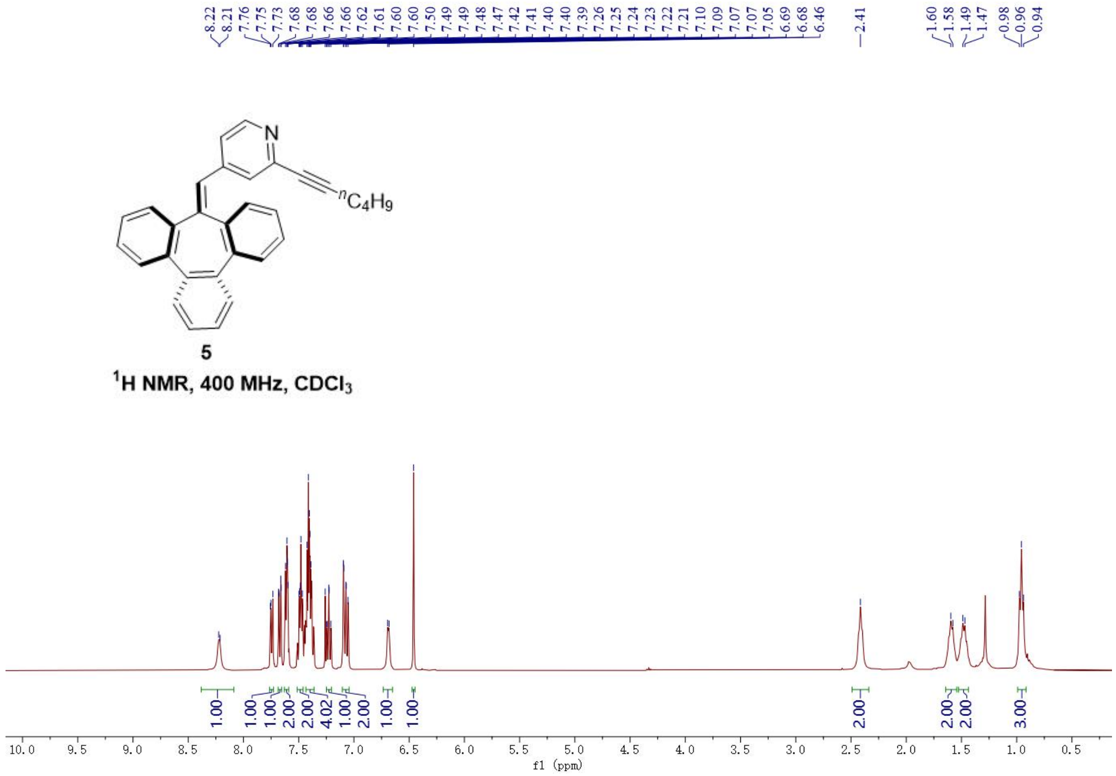
8.22
8.21
7.76
7.75
7.73
7.68
7.68
7.66
7.66
7.62
7.61
7.60
7.60
7.50
7.49
7.49
7.48
7.47
7.42
7.41
7.40
7.40
7.39
7.26
7.25
7.24
7.23
7.22
7.21
7.10
7.09
7.07
7.07
7.05
6.69
6.68
6.46
2.41
1.60
1.58
1.49
1.47
0.98
0.96
0.94
N
$^nC_4H_9$
5
^{1}H NMR, 400 MHz, $CDCl_3$
1.00
1.00
1.00
2.00
2.00
4.02
1.00
2.00
1.00
1.00
2.00
2.00
2.00
3.00
f1 (ppm)

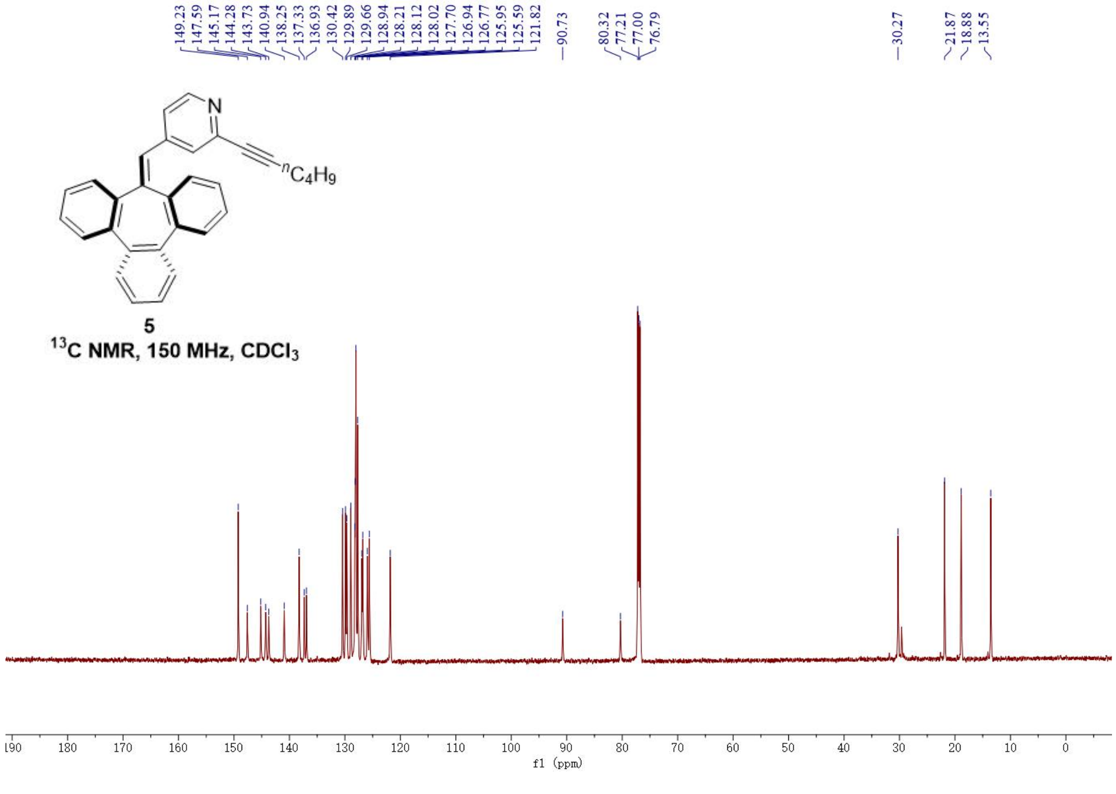
149.23
147.59
145.17
144.28
143.73
140.94
138.25
137.33
136.93
130.42
129.89
129.66
128.94
128.21
128.12
128.02
127.70
126.94
126.77
125.95
125.59
121.82
90.73
80.32
77.21
77.00
76.79
30.27
21.87
18.88
13.55
N
$^nC_4H_9$
5
^{13}C NMR, 150 MHz, $CDCl_3$
f1 (ppm)

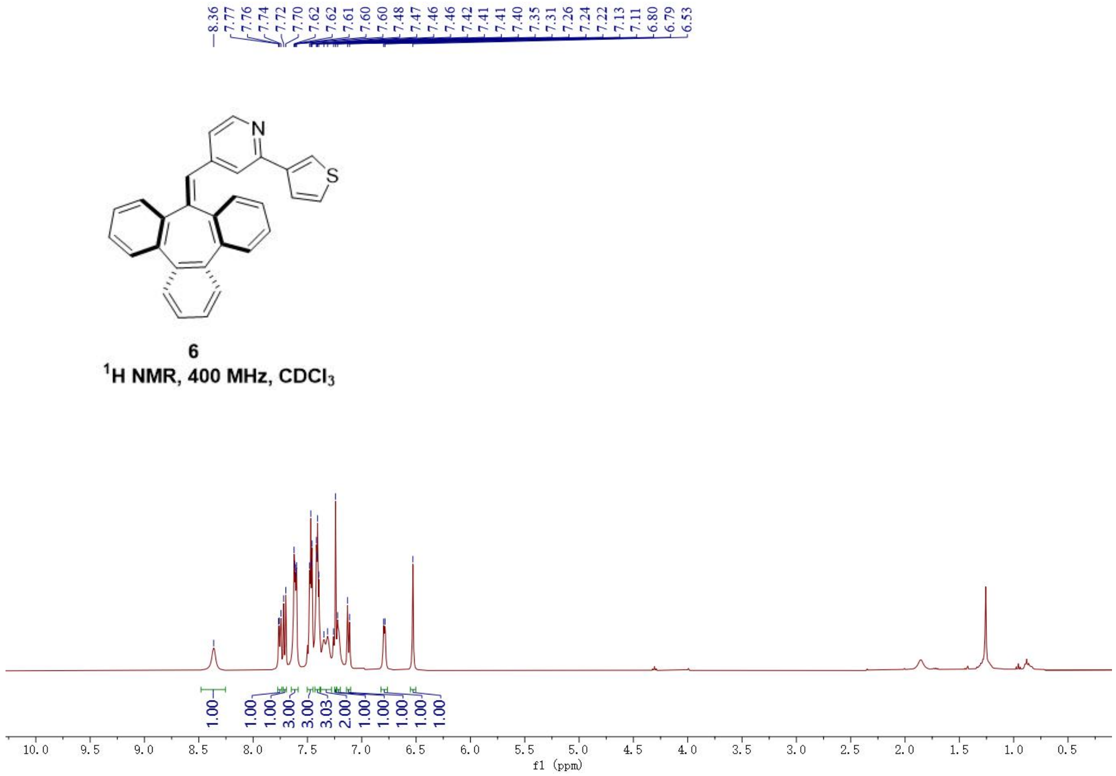

6
^{1}H NMR, 400 MHz, $CDCl_3$

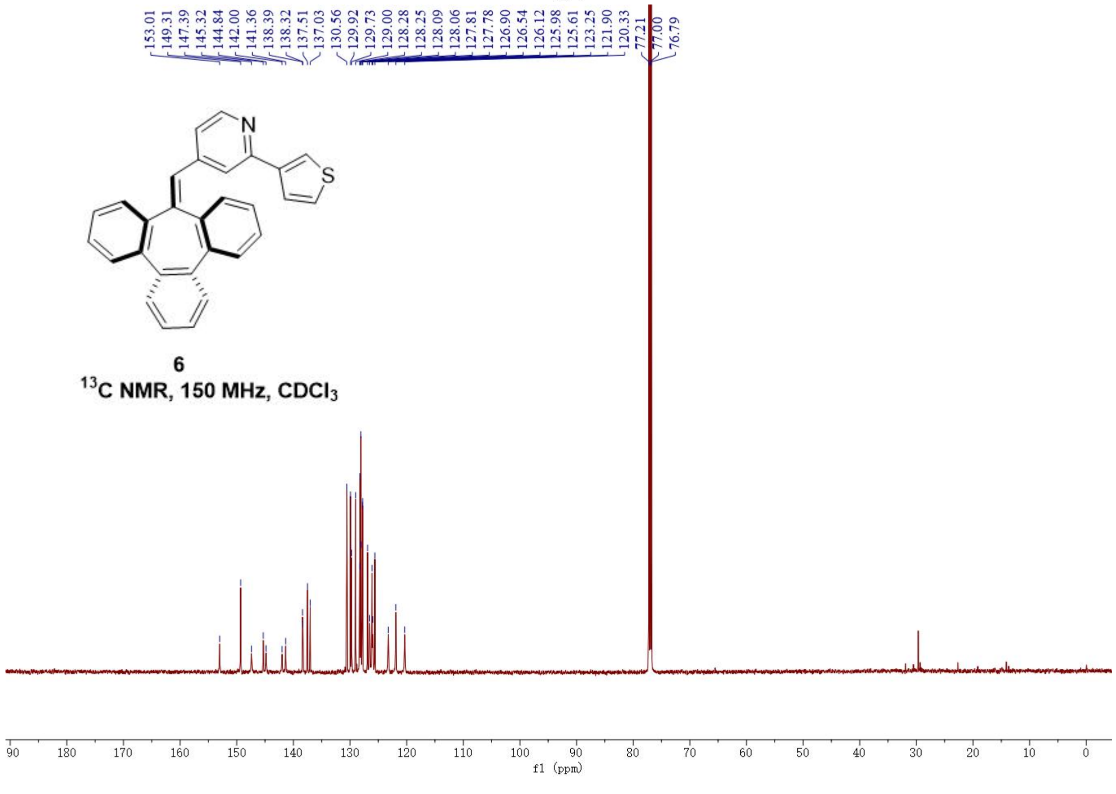

6
^{13}C NMR, 150 MHz, $CDCl_3$

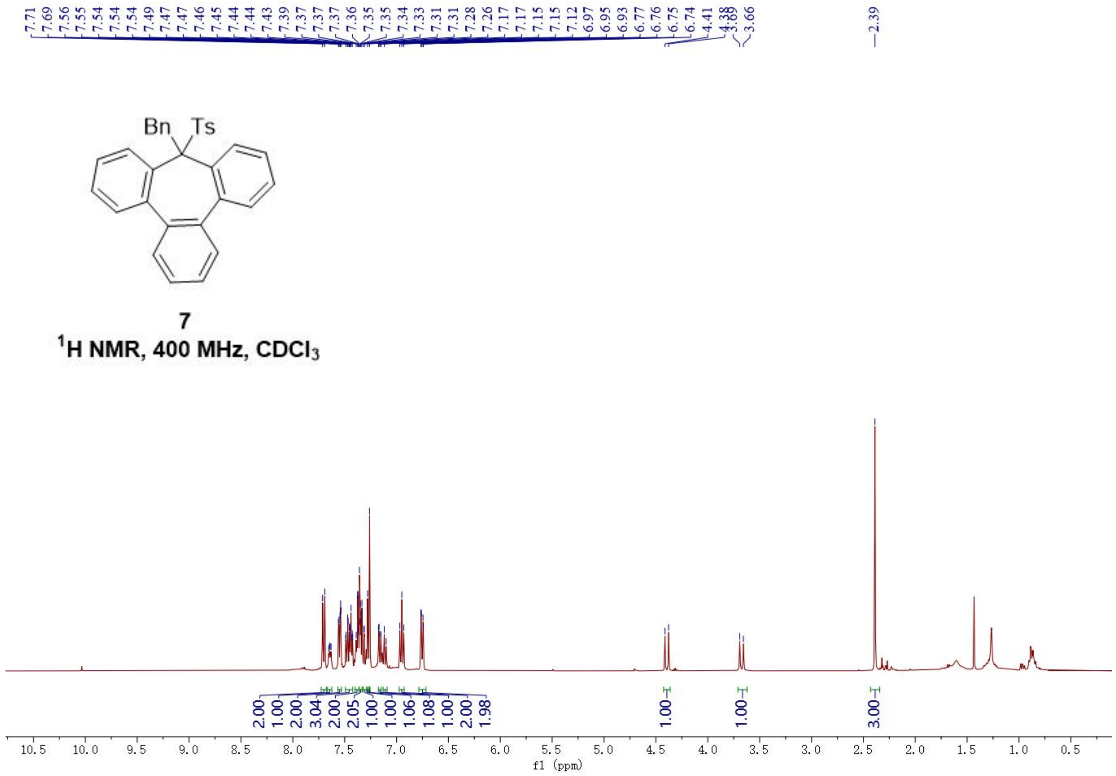

HPLC conditions: Chiralpak AS-H, 15% ^{i}PrOH/Hx eluent, 0.5 mL/min, 254 nm

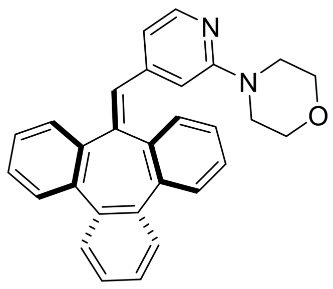

4

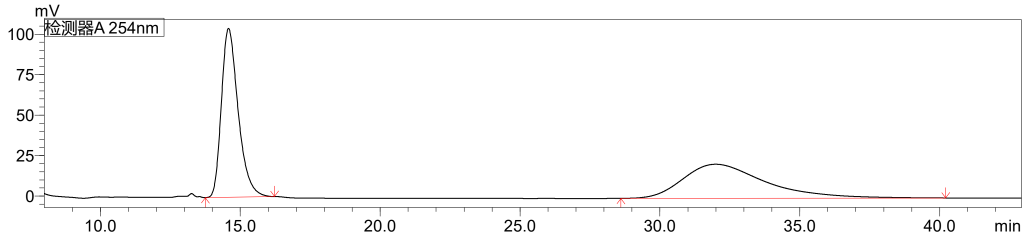

Peak#	Ret. Time	Height	Area%
1	14.579	104256	50.393
2	31.994	21018	49.607

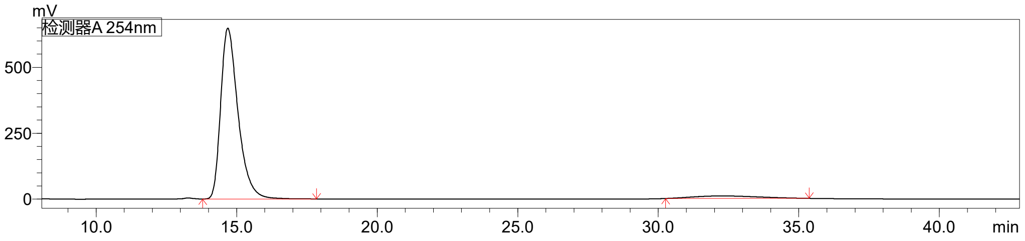

Peak#	Ret. Time	Height	Area%
1	14.687	647982	94.703
2	32.268	9524	5.297

HPLC conditions: Chiralpak IA-H, 10% iPrOH/Hx eluent, 0.5 mL/min, 254 nm

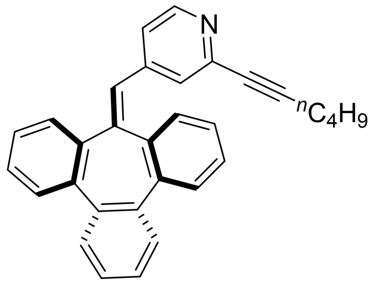

5

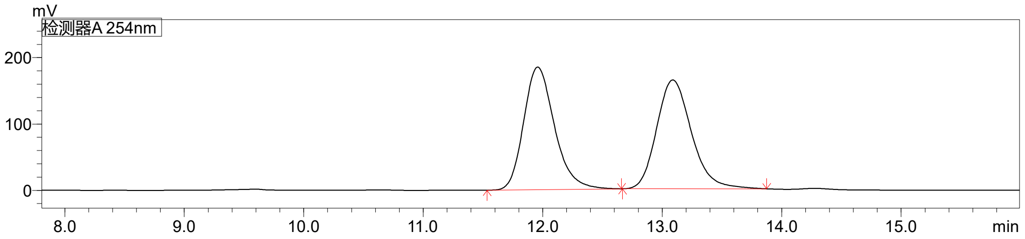

Peak#	Ret. Time	Height	Area%
1	11.958	184929	49.934
2	13.088	164050	50.066

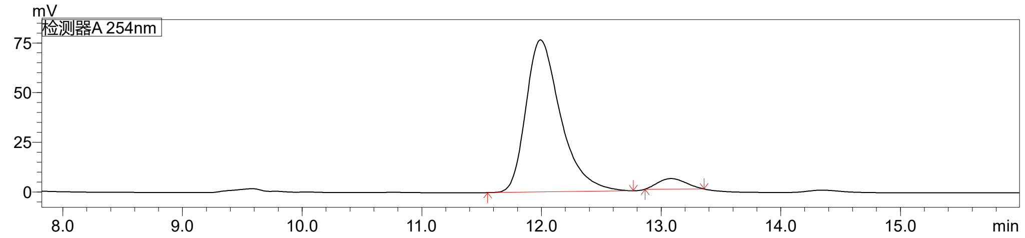

Peak#	Ret. Time	Height	Area%
1	11.991	76520	94.782

2	13.081	5282	5.218

HPLC conditions: Chiralpak AD-H, 10% iPrOH/Hx eluent, 0.5 mL/min, 254 nm

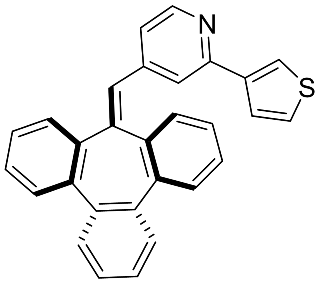

6

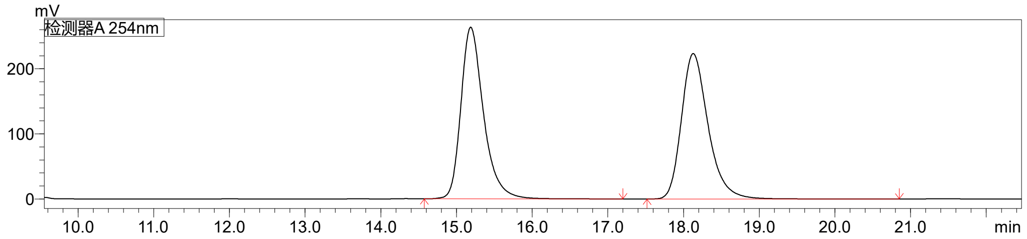

Peak#	Ret. Time	Height	Area%
1	15.187	263636	50.080
2	18.127	223320	49.920

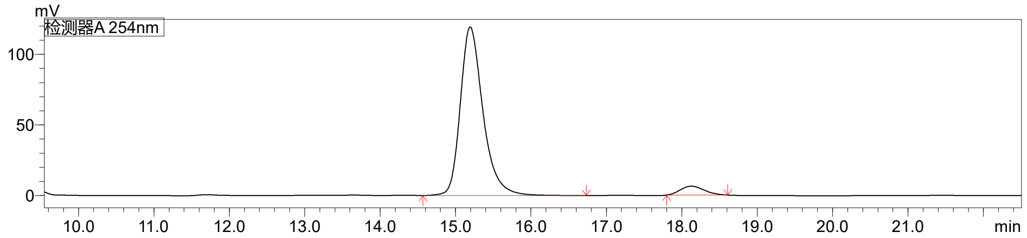

Peak#	Ret. Time	Height	Area%
1	15.194	119295	94.741
2	18.124	6307	5.259

9. Gram scale reaction

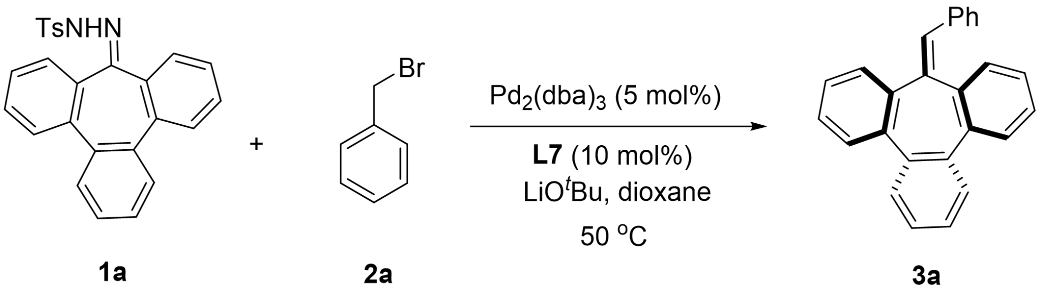

Add reactants **1a** (0.849 g, 2 mmol) and **2a** (0.29 mL, 2.4 mmol), $Pd_2(dba)_3$ (91.6 mg, 0.1 mmol), **L7** (0.108 g, 0.2 mmol), LiO^tBu (0.480 g, 4 mmol) to a Schlenk tube, then under nitrogen atmosphere, solvent dioxane (20 mL) and react at 50 °C using oil bath for 36 hours. After the mixture was cooled to room temperature, the solvent was removed under vacuum and further purification by flash column chromatography on silica gel (petroleum ether: EtOAc = 100:1) to provide **3a** (0.66 g, 73%, 91% ee) as a white solid.

HPLC conditions: Chiralpak IA-H, 5% ^{i}PrOH/Hx eluent, 0.5 mL/min, 254 nm

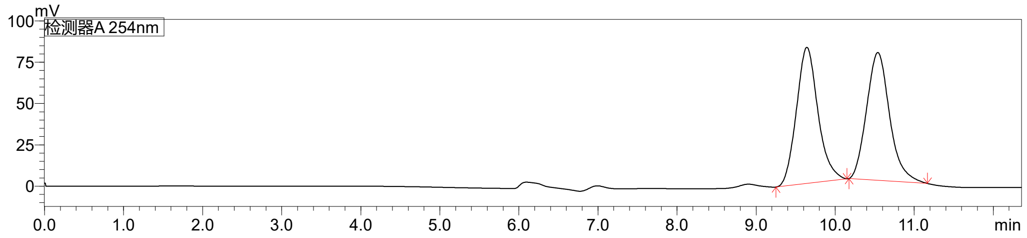

Peak#	Ret. Time	Height	Area%
1	9.642	82447	49.990
2	10.540	77330	50.010

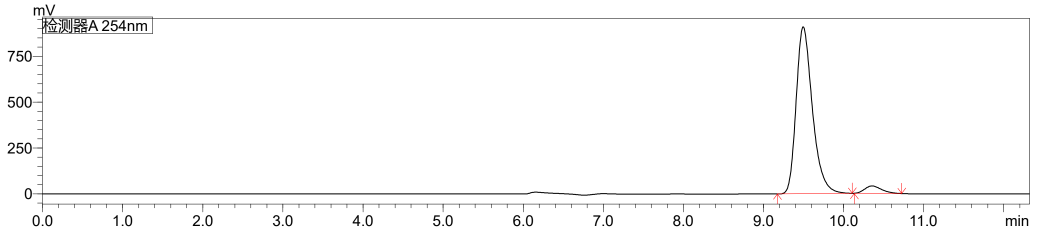

Peak#	Ret. Time	Height	Area%
1	9.496	908183	95.587
2	10.356	40373	4.413

10. Crystallographic data for 3w

Supplementary Table 3 Crystal data and structure refinement for 3w.

Identification code	**3w**
Empirical formula	$C_{26}H_{16}Cl_2$
Formula weight	399.29
Temperature/K	150.00(10)
Crystal system	orthorhombic
Space group	$P2_12_12_1$
a/Å	10.8510(3)
b/Å	16.2553(4)
c/Å	22.1782(4)
α/°	90
β/°	90
γ/°	90
Volume/Å^3	3911.93(16)
Z	8
ρ_{calc}g/cm^3	1.356
μ/mm^{-1}	3.032
F(000)	1648.0
Crystal size/mm^3	0.16 × 0.13 × 0.12
Radiation	Cu Kα (λ = 1.54184)
2Θ range for data collection/°	6.742 to 148.008
Index ranges	-13 ≤ h ≤ 12, -20 ≤ k ≤ 19, -17 ≤ l ≤ 27
Reflections collected	15140
Independent reflections	7723 [R_{int} = 0.0411, R_{sigma} = 0.0482]
Data/restraints/parameters	7723/0/505
Goodness-of-fit on F^2	1.039

Final R indexes [I>=2σ (I)]	R_1 = 0.0464, wR_2 = 0.1199
Final R indexes [all data]	R_1 = 0.0490, wR_2 = 0.1246
Largest diff. peak/hole / e Å^{-3}	0.26/-0.38
Flack/Hooft parameter	-0.001(7)/-0.001(8)

Crystal structure determination of [3w]

Crystal Data for $C_{26}H_{16}Cl_2$ (*M* =399.29 g/mol): orthorhombic, space group $P2_12_12_1$ (no. 19), *a* = 10.8510(3) Å, *b* = 16.2553(4) Å, *c* = 22.1782(4) Å, *V* = 3911.93(16) Å^3, *Z* = 8, *T* = 150.00(10) K, μ(Cu Kα) = 3.032 mm^{-1}, *Dcalc* = 1.356 g/cm^3, 15140 reflections measured (6.742° ≤ 2Θ ≤ 148.008°), 7723 unique (R_{int} = 0.0411, R_{sigma} = 0.0482) which were used in all calculations. The final R_1 was 0.0464 (I > 2σ(I)) and wR_2 was 0.1246 (all data).

Refinement model description

Supplementary Table 4 Fractional Atomic Coordinates (×10^4) and Equivalent Isotropic Displacement Parameters (Å^2×10^3) for 3w. U_{eq} is defined as 1/3 of the trace of the orthogonalised U_{IJ} tensor.

Atom	*x*	*y*	*z*	U(eq)
Cl1	5859.0(9)	5833.5(8)	3787.5(4)	53.5(3)
Cl2	8111.9(8)	6353.1(9)	2984.9(6)	62.7(3)
C1	922(3)	4809.9(19)	3006.4(12)	25.9(5)
C2	1170(3)	4217(2)	3448.5(14)	32.4(6)
C3	986(4)	3388(2)	3344.2(15)	37.8(7)
C4	565(3)	3128(2)	2787.2(16)	39.4(7)
C5	322(3)	3706(2)	2341.3(14)	34.2(7)
C6	488(3)	4553(2)	2438.4(13)	28.1(6)
C7	141(3)	5124(2)	1942.9(12)	29.3(6)
C8	-910(3)	4950(3)	1594.3(14)	39.6(8)
C9	-1251(4)	5442(3)	1117.9(17)	48.8(9)

Supplementary Table 4 Fractional Atomic Coordinates (×10⁴) and Equivalent Isotropic Displacement Parameters (Å²×10³) for 3w. U_{eq} is defined as 1/3 of the trace of the orthogonalised U_{IJ} tensor.

**Atom**	***x***	***y***	***z***	**U(eq)**
C10	-555(4)	6127(3)	972.8(16)	48.2(9)
C11	500(3)	6311(2)	1303.7(14)	37.4(7)
C12	848(3)	5815.4(19)	1789.6(12)	27.9(6)
C13	1963(3)	6022.4(18)	2150.4(12)	26.6(6)
C14	1661(3)	6250.8(18)	2782.0(12)	25.3(5)
C15	1106(2)	5685.3(19)	3173.2(12)	25.2(6)
C16	776(3)	5952(2)	3751.4(13)	31.3(6)
C17	982(3)	6750(2)	3936.9(14)	36.3(7)
C18	1537(3)	7306(2)	3546.7(14)	34.4(7)
C19	1872(3)	7055.7(19)	2973.5(14)	30.6(6)
C20	3078(3)	6041.0(19)	1896.2(12)	28.0(6)
C21	4278(3)	6160.0(18)	2181.0(13)	28.2(6)
C22	5291(3)	6398(2)	1831.2(15)	37.2(7)
C23	6459(4)	6473(2)	2077.5(17)	44.7(8)
C24	6640(3)	6307(2)	2677.2(17)	39.8(7)
C25	5652(3)	6069(2)	3030.8(15)	34.4(6)
C26	4493(3)	5996(2)	2790.2(14)	31.0(6)
Cl3	2538.9(12)	8719.6(9)	1225.7(4)	67.7(4)
Cl4	5420.8(12)	8399.8(7)	1033.0(4)	59.1(3)
C27	3586(4)	8808(2)	1815.2(15)	42.7(8)
C28	4822(4)	8661(2)	1735.4(14)	39.4(8)
C29	5635(3)	8732(2)	2213.6(14)	34.7(7)
C30	5212(3)	8949.0(19)	2788.5(13)	30.6(6)

Supplementary Table 4 Fractional Atomic Coordinates (×10^{4}) and Equivalent Isotropic Displacement Parameters (Å^{2}×10^{3}) for 3w. U_{eq} is defined as 1/3 of the trace of the orthogonalised U_{IJ} tensor.

**Atom**	***x***	***y***	***z***	**U(eq)**
C31	3155(4)	9038(3)	2374.8(16)	44.9(9)
C32	3955(3)	9108(2)	2859.7(14)	36.9(7)
C33	6149(3)	9042.6(19)	3262.0(13)	30.0(6)
C34	6058(3)	9047.8(19)	3866.2(13)	29.7(6)
C35	7196(3)	9161(2)	4224.0(13)	30.6(6)
C36	8200(4)	8635(3)	4121.6(15)	41.7(8)
C37	9294(4)	8738(3)	4430.4(17)	52.1(10)
C38	9391(4)	9347(3)	4857.4(17)	53.7(11)
C39	8406(4)	9856(3)	4975.9(15)	46.1(9)
C40	7294(3)	9781(2)	4656.5(13)	32.6(7)
C41	6314(3)	10405(2)	4747.7(12)	32.8(7)
C42	6702(4)	11220(2)	4831.5(14)	44.2(9)
C43	5873(5)	11849(2)	4936.9(16)	53.1(11)
C44	4626(5)	11670(2)	4969.3(16)	53.5(11)
C45	4221(4)	10872(2)	4883.1(13)	42.7(8)
C46	5034(3)	10228(2)	4764.3(12)	31.7(7)
C47	4534(3)	9389(2)	4687.8(12)	31.3(7)
C48	3567(3)	9124(3)	5060.7(14)	38.6(7)
C49	3081(3)	8342(3)	5014.8(15)	44.8(9)
C50	3534(4)	7802(3)	4582.6(16)	44.6(9)
C51	4460(3)	8057(2)	4197.3(14)	37.8(7)
C52	4968(3)	8844(2)	4243.1(12)	29.1(6)

Supplementary Table 5 Anisotropic Displacement Parameters ($Å^2 \times 10^3$) for 3w. The Anisotropic displacement factor exponent takes the form: $-2\pi^2[h^2a^{*2}U_{11}+2hka^*b^*U_{12}+\ldots]$.

**Atom**	**U_{11}**	**U_{22}**	**U_{33}**	**U_{23}**	**U_{13}**	**U_{12}**
Cl1	43.1(5)	87.1(7)	30.3(4)	3.9(4)	-6.4(3)	4.3(5)
Cl2	27.5(4)	87.4(8)	73.1(7)	10.8(6)	-6.1(4)	-8.8(5)
C1	20.9(12)	34.6(14)	22.1(12)	0.6(11)	2.0(11)	-2.1(11)
C2	31.9(15)	39.0(16)	26.3(13)	4.8(12)	-0.3(11)	-2.2(13)
C3	41.3(18)	36.9(17)	35.3(16)	7.3(13)	0.6(14)	-2.8(15)
C4	42.4(19)	34.1(16)	41.7(17)	-3.0(14)	3.0(15)	-8.8(14)
C5	32.0(15)	39.5(17)	31.1(14)	-3.7(12)	1.9(12)	-7.4(14)
C6	20.2(13)	35.6(15)	28.5(13)	0.4(12)	0.6(11)	-3.0(11)
C7	27.3(14)	38.8(15)	21.9(12)	-3.8(11)	-2.4(11)	1.7(13)
C8	30.2(16)	57(2)	31.8(15)	-3.1(14)	-5.9(13)	-3.5(16)
C9	38.9(19)	69(3)	38.4(18)	-2.8(18)	-18.1(15)	9.2(18)
C10	55(2)	57(2)	32.3(16)	6.7(15)	-14.2(16)	16.2(19)
C11	45.0(18)	41.2(17)	26.1(13)	5.1(13)	-2.6(13)	8.5(15)
C12	29.0(14)	33.6(14)	21.1(12)	-1.7(11)	1.4(11)	3.5(13)
C13	30.5(14)	28.9(13)	20.6(12)	5.1(10)	0.4(11)	0.8(11)
C14	23.6(12)	31.2(14)	21.0(12)	-0.4(11)	-0.4(10)	0.1(11)
C15	18.4(12)	34.3(15)	22.9(12)	0.7(11)	-1.6(10)	-0.3(11)
C16	27.5(14)	42.2(17)	24.1(13)	-1.1(12)	2.1(11)	-0.2(13)
C17	38.2(17)	46.4(18)	24.2(13)	-9.3(12)	0.6(12)	5.6(15)
C18	37.4(17)	34.3(15)	31.5(15)	-6.4(12)	-6.1(13)	5.4(14)
C19	31.8(14)	29.8(14)	30.2(14)	0.8(11)	-2.6(12)	1.9(12)
C20	36.0(15)	29.8(14)	18.2(11)	5.2(10)	2.5(11)	-1.1(12)
C21	29.2(14)	30.0(13)	25.5(13)	0.6(11)	5.3(12)	1.3(12)
C22	35.5(16)	43.7(17)	32.5(15)	6.8(13)	9.7(13)	-3.8(15)

Supplementary Table 5 Anisotropic Displacement Parameters (Å^2×10^3) for 3w. The Anisotropic displacement factor exponent takes the form: -2π^2[h^{2}a*^{2}U$_{11}$+2hka*b*U$_{12}$+…].

Atom	U11	U22	U33	U23	U13	U12
C23	35.6(17)	52(2)	46.7(19)	7.5(17)	13.9(15)	-3.6(16)
C24	26.3(15)	43.9(18)	49.2(19)	-0.9(15)	1.7(14)	-0.5(14)
C25	35.9(16)	36.7(16)	30.6(14)	-0.2(12)	-0.1(13)	3.9(13)
C26	26.7(14)	38.5(16)	27.7(13)	1.0(12)	7.7(11)	-0.7(12)
Cl3	67.5(7)	98.0(9)	37.4(5)	4.2(5)	-21.4(5)	-27.6(7)
Cl4	84.3(8)	71.1(6)	21.8(4)	-11.8(4)	6.5(4)	-9.7(6)
C27	53(2)	50.0(19)	25.5(14)	4.4(14)	-9.3(14)	-18.4(18)
C28	59(2)	36.8(16)	22.2(13)	-4.9(12)	2.4(14)	-12.1(17)
C29	43.6(18)	35.0(15)	25.3(13)	-2.7(12)	3.9(13)	-2.0(14)
C30	38.9(16)	32.1(14)	20.8(12)	-1.9(11)	2.3(12)	-2.9(13)
C31	37.9(18)	63(2)	34.0(16)	4.1(16)	-0.9(14)	-8.2(18)
C32	36.0(16)	50.4(19)	24.3(13)	-1.7(13)	3.5(12)	-4.0(15)
C33	34.7(16)	32.8(14)	22.6(13)	-4.5(11)	4.2(12)	-1.7(13)
C34	36.9(16)	30.1(14)	22.1(13)	-1.4(11)	2.3(11)	-3.4(13)
C35	34.6(16)	36.4(16)	21.0(12)	5.5(12)	1.5(11)	-1.0(13)
C36	41.7(18)	51(2)	32.1(15)	6.1(14)	5.4(14)	8.3(17)
C37	40.0(19)	75(3)	41.5(18)	14.5(19)	3.7(16)	17(2)
C38	33.5(18)	91(3)	37.0(18)	12.9(19)	-7.7(15)	-3(2)
C39	46(2)	66(2)	26.5(15)	1.5(15)	-5.7(15)	-10.0(19)
C40	36.9(17)	40.9(17)	20.1(12)	1.1(12)	-1.0(12)	-6.2(14)
C41	47.6(19)	36.9(16)	14.0(11)	-1.2(11)	-1.0(11)	-4.8(14)
C42	67(2)	41.7(19)	23.6(14)	-6.2(13)	1.3(15)	-10.9(19)
C43	91(3)	38.8(19)	29.1(16)	-7.6(14)	5.4(19)	-2(2)
C44	90(3)	42.6(19)	28.5(15)	-5.1(14)	4.3(18)	22(2)

Supplementary Table 5 Anisotropic Displacement Parameters ($Å^2 \times 10^3$) for 3w. The Anisotropic displacement factor exponent takes the form: -$2\pi^2[h^2a^{*2}U_{11}+2hka^*b^*U_{12}+\ldots]$.

Atom	U_{11}	U_{22}	U_{33}	U_{23}	U_{13}	U_{12}
C45	56(2)	51(2)	21.6(13)	-3.9(13)	1.2(14)	14.2(18)
C46	41.3(17)	39.5(17)	14.1(11)	-0.5(11)	0.9(11)	5.0(14)
C47	30.9(15)	44.4(18)	18.7(12)	3.2(12)	-3.3(11)	-1.7(14)
C48	31.0(15)	62(2)	23.3(13)	4.5(14)	1.0(12)	0.4(16)
C49	34.1(16)	71(3)	29.5(15)	14.6(16)	-1.8(13)	-10.9(18)
C50	44.9(19)	53(2)	36.3(16)	10.6(15)	-10.3(15)	-17.0(18)
C51	45.6(19)	39.7(17)	28.1(14)	3.3(13)	-6.0(14)	-9.0(15)
C52	32.3(15)	35.4(15)	19.7(12)	1.0(11)	-2.6(11)	-3.7(12)

Supplementary Table 6 Bond Lengths for 3w.

Atom	Atom	Length/Å	Atom	Atom	Length/Å
Cl1	C25	1.736(3)	Cl3	C27	1.738(4)
Cl2	C24	1.738(4)	Cl4	C28	1.740(3)
C1	C2	1.401(4)	C27	C28	1.374(6)
C1	C6	1.408(4)	C27	C31	1.378(5)
C1	C15	1.484(4)	C28	C29	1.384(5)
C2	C3	1.383(5)	C29	C30	1.400(4)
C3	C4	1.383(5)	C30	C32	1.397(5)
C4	C5	1.390(5)	C30	C33	1.470(4)
C5	C6	1.405(5)	C31	C32	1.386(5)
C6	C7	1.487(4)	C33	C34	1.344(4)
C7	C8	1.407(4)	C34	C35	1.479(4)
C7	C12	1.402(5)	C34	C52	1.486(4)

Supplementary Table 6 Bond Lengths for 3w.

Atom	Atom	Length/Å	Atom	Atom	Length/Å
C8	C9	1.376(5)	C35	C36	1.403(5)
C9	C10	1.382(6)	C35	C40	1.396(5)
C10	C11	1.392(5)	C36	C37	1.380(6)
C11	C12	1.397(4)	C37	C38	1.374(7)
C12	C13	1.489(4)	C38	C39	1.378(6)
C13	C14	1.486(4)	C39	C40	1.404(5)
C13	C20	1.336(4)	C40	C41	1.484(5)
C14	C15	1.401(4)	C41	C42	1.401(5)
C14	C19	1.394(4)	C41	C46	1.419(5)
C15	C16	1.400(4)	C42	C43	1.382(6)
C16	C17	1.380(5)	C43	C44	1.385(7)
C17	C18	1.389(5)	C44	C45	1.383(6)
C18	C19	1.384(5)	C45	C46	1.394(5)
C20	C21	1.460(4)	C46	C47	1.478(5)
C21	C22	1.400(4)	C47	C48	1.403(5)
C21	C26	1.397(4)	C47	C52	1.406(4)
C22	C23	1.385(5)	C48	C49	1.380(6)
C23	C24	1.371(6)	C49	C50	1.390(6)
C24	C25	1.384(5)	C50	C51	1.382(5)
C25	C26	1.372(5)	C51	C52	1.398(5)

Supplementary Table 7 Bond Angles for 3w.

Atom	Atom	Atom	Angle/°	Atom	Atom	Atom	Angle/°
C2	C1	C6	119.1(3)	C28	C27	Cl3	121.8(3)

Supplementary Table 7 Bond Angles for 3w.

Atom	Atom	Atom	Angle/°	Atom	Atom	Atom	Angle/°
C2	C1	C15	117.3(3)	C28	C27	C31	119.6(3)
C6	C1	C15	123.5(3)	C31	C27	Cl3	118.6(3)
C3	C2	C1	121.7(3)	C27	C28	Cl4	121.5(3)
C2	C3	C4	119.7(3)	C27	C28	C29	120.6(3)
C3	C4	C5	119.4(3)	C29	C28	Cl4	117.9(3)
C4	C5	C6	122.0(3)	C28	C29	C30	120.6(3)
C1	C6	C7	124.1(3)	C29	C30	C33	116.7(3)
C5	C6	C1	118.0(3)	C32	C30	C29	118.0(3)
C5	C6	C7	117.8(3)	C32	C30	C33	125.1(3)
C8	C7	C6	119.0(3)	C27	C31	C32	120.6(4)
C12	C7	C6	122.8(3)	C31	C32	C30	120.5(3)
C12	C7	C8	118.1(3)	C34	C33	C30	131.5(3)
C9	C8	C7	121.5(4)	C33	C34	C35	118.4(3)
C8	C9	C10	120.0(3)	C33	C34	C52	128.1(3)
C9	C10	C11	120.0(3)	C35	C34	C52	113.0(2)
C10	C11	C12	120.3(3)	C36	C35	C34	119.1(3)
C7	C12	C13	119.7(3)	C40	C35	C34	121.5(3)
C11	C12	C7	120.0(3)	C40	C35	C36	119.5(3)
C11	C12	C13	120.3(3)	C37	C36	C35	120.9(4)
C14	C13	C12	112.6(3)	C38	C37	C36	119.7(4)
C20	C13	C12	121.0(3)	C37	C38	C39	120.3(4)
C20	C13	C14	126.3(3)	C38	C39	C40	121.2(4)
C15	C14	C13	121.0(3)	C35	C40	C39	118.4(3)
C19	C14	C13	119.1(3)	C35	C40	C41	122.2(3)
C19	C14	C15	119.9(3)	C39	C40	C41	119.2(3)

Supplementary Table 7 Bond Angles for 3w.

Atom	Atom	Atom	Angle/°	Atom	Atom	Atom	Angle/°
C14	C15	C1	122.2(3)	C42	C41	C40	116.7(3)
C16	C15	C1	119.4(3)	C42	C41	C46	118.8(4)
C16	C15	C14	118.3(3)	C46	C41	C40	124.5(3)
C17	C16	C15	121.5(3)	C43	C42	C41	121.7(4)
C16	C17	C18	119.8(3)	C42	C43	C44	119.3(4)
C19	C18	C17	119.7(3)	C45	C44	C43	120.0(4)
C18	C19	C14	120.9(3)	C44	C45	C46	122.0(4)
C13	C20	C21	129.0(3)	C41	C46	C47	122.9(3)
C22	C21	C20	119.8(3)	C45	C46	C41	118.1(3)
C26	C21	C20	122.8(3)	C45	C46	C47	118.8(3)
C26	C21	C22	117.3(3)	C48	C47	C46	119.3(3)
C23	C22	C21	121.6(3)	C48	C47	C52	118.1(3)
C24	C23	C22	119.8(3)	C52	C47	C46	122.6(3)
C23	C24	Cl2	120.3(3)	C49	C48	C47	121.6(3)
C23	C24	C25	119.6(3)	C48	C49	C50	119.8(3)
C25	C24	Cl2	120.1(3)	C51	C50	C49	119.6(4)
C24	C25	Cl1	120.6(3)	C50	C51	C52	121.1(3)
C26	C25	Cl1	118.4(3)	C47	C52	C34	121.4(3)
C26	C25	C24	121.0(3)	C51	C52	C34	118.5(3)
C25	C26	C21	120.8(3)	C51	C52	C47	119.7(3)

Supplementary Table 8 Torsion Angles for 3w.

A	B	C	D	Angle/°	A	B	C	D	Angle/°
Cl1	C25	C26	C21	-178.6(2)	Cl3	C27	C28	Cl4	1.6(5)

Supplementary Table 8 Torsion Angles for 3w.

A	B	C	D	Angle/°	A	B	C	D	Angle/°
Cl2	C24	C25	Cl1	1.3(5)	Cl3	C27	C28	C29	-179.7(3)
Cl2	C24	C25	C26	-177.3(3)	Cl3	C27	C31	C32	179.3(3)
C1	C2	C3	C4	1.1(5)	Cl4	C28	C29	C30	179.3(3)
C1	C6	C7	C8	-139.5(3)	C27	C28	C29	C30	0.6(5)
C1	C6	C7	C12	43.3(4)	C27	C31	C32	C30	0.3(6)
C1	C15	C16	C17	176.9(3)	C28	C27	C31	C32	-1.2(6)
C2	C1	C6	C5	0.0(4)	C28	C29	C30	C32	-1.4(5)
C2	C1	C6	C7	177.2(3)	C28	C29	C30	C33	-177.6(3)
C2	C1	C15	C14	137.9(3)	C29	C30	C32	C31	1.0(5)
C2	C1	C15	C16	-38.6(4)	C29	C30	C33	C34	-161.8(3)
C2	C3	C4	C5	-0.6(6)	C30	C33	C34	C35	-179.7(3)
C3	C4	C5	C6	-0.2(5)	C30	C33	C34	C52	9.1(6)
C4	C5	C6	C1	0.5(5)	C31	C27	C28	Cl4	-178.0(3)
C4	C5	C6	C7	-176.9(3)	C31	C27	C28	C29	0.7(6)
C5	C6	C7	C8	37.7(4)	C32	C30	C33	C34	22.3(6)
C5	C6	C7	C12	-139.5(3)	C33	C30	C32	C31	176.8(3)
C6	C1	C2	C3	-0.8(5)	C33	C34	C35	C36	-53.9(4)
C6	C1	C15	C14	-43.7(4)	C33	C34	C35	C40	125.6(3)
C6	C1	C15	C16	139.8(3)	C33	C34	C52	C47	-127.2(4)
C6	C7	C8	C9	-177.8(3)	C33	C34	C52	C51	60.1(5)
C6	C7	C12	C11	177.5(3)	C34	C35	C36	C37	177.7(3)
C6	C7	C12	C13	-3.1(4)	C34	C35	C40	C39	-179.5(3)
C7	C8	C9	C10	-0.1(6)	C34	C35	C40	C41	-5.3(5)
C7	C12	C13	C14	-65.0(4)	C35	C34	C52	C47	61.2(4)
C7	C12	C13	C20	119.0(3)	C35	C34	C52	C51	-111.4(3)

Supplementary Table 8 Torsion Angles for 3w.

A	B	C	D	Angle/°	A	B	C	D	Angle/°
C8	C7	C12	C11	0.2(5)	C35	C36	C37	C38	2.0(6)
C8	C7	C12	C13	179.6(3)	C35	C40	C41	C42	-137.5(3)
C8	C9	C10	C11	0.9(6)	C35	C40	C41	C46	43.6(4)
C9	C10	C11	C12	-1.2(6)	C36	C35	C40	C39	0.0(5)
C10	C11	C12	C7	0.6(5)	C36	C35	C40	C41	174.2(3)
C10	C11	C12	C13	-178.8(3)	C36	C37	C38	C39	-0.4(6)
C11	C12	C13	C14	114.4(3)	C37	C38	C39	C40	-1.5(6)
C11	C12	C13	C20	-61.5(4)	C38	C39	C40	C35	1.7(5)
C12	C7	C8	C9	-0.5(5)	C38	C39	C40	C41	-172.7(3)
C12	C13	C14	C15	62.7(4)	C39	C40	C41	C42	36.7(4)
C12	C13	C14	C19	-113.5(3)	C39	C40	C41	C46	-142.3(3)
C12	C13	C20	C21	-174.3(3)	C40	C35	C36	C37	-1.9(5)
C13	C14	C15	C1	7.3(4)	C40	C41	C42	C43	-178.2(3)
C13	C14	C15	C16	-176.2(3)	C40	C41	C46	C45	176.9(3)
C13	C14	C19	C18	176.1(3)	C40	C41	C46	C47	-0.4(4)
C13	C20	C21	C22	-161.4(3)	C41	C42	C43	C44	0.9(5)
C13	C20	C21	C26	22.9(5)	C41	C46	C47	C48	137.9(3)
C14	C13	C20	C21	10.3(5)	C41	C46	C47	C52	-43.7(4)
C14	C15	C16	C17	0.3(4)	C42	C41	C46	C45	-2.0(4)
C15	C1	C2	C3	177.6(3)	C42	C41	C46	C47	-179.3(3)
C15	C1	C6	C5	-178.3(3)	C42	C43	C44	C45	-1.5(6)
C15	C1	C6	C7	-1.1(5)	C43	C44	C45	C46	0.2(5)
C15	C14	C19	C18	-0.1(5)	C44	C45	C46	C41	1.6(5)
C15	C16	C17	C18	-0.5(5)	C44	C45	C46	C47	178.9(3)
C16	C17	C18	C19	0.4(5)	C45	C46	C47	C48	-39.4(4)

Supplementary Table 8 Torsion Angles for 3w.

A	B	C	D	Angle/°	A	B	C	D	Angle/°
C17	C18	C19	C14	-0.1(5)	C45	C46	C47	C52	139.0(3)
C19	C14	C15	C1	-176.5(3)	C46	C41	C42	C43	0.8(5)
C19	C14	C15	C16	0.0(4)	C46	C47	C48	C49	-178.9(3)
C20	C13	C14	C15	-121.6(3)	C46	C47	C52	C34	7.0(5)
C20	C13	C14	C19	62.2(4)	C46	C47	C52	C51	179.6(3)
C20	C21	C22	C23	-176.4(3)	C47	C48	C49	C50	-1.3(5)
C20	C21	C26	C25	176.0(3)	C48	C47	C52	C34	-174.6(3)
C21	C22	C23	C24	0.4(6)	C48	C47	C52	C51	-2.0(5)
C22	C21	C26	C25	0.2(5)	C48	C49	C50	C51	-0.7(5)
C22	C23	C24	Cl2	177.1(3)	C49	C50	C51	C52	1.4(5)
C22	C23	C24	C25	-0.2(6)	C50	C51	C52	C34	172.8(3)
C23	C24	C25	Cl1	178.6(3)	C50	C51	C52	C47	0.0(5)
C23	C24	C25	C26	0.0(6)	C52	C34	C35	C36	118.5(3)
C24	C25	C26	C21	0.0(5)	C52	C34	C35	C40	-62.0(4)
C26	C21	C22	C23	-0.4(5)	C52	C47	C48	C49	2.6(5)

Supplementary Table 9 Hydrogen Atom Coordinates (Å×10^4) and Isotropic Displacement Parameters (Å^2×10^3) for 3w.

Atom	*x*	*y*	*z*	U(eq)
H2	1473.75	4389.78	3829.89	39
H3	1146.95	2998.1	3653.61	45
H4	442.46	2558.9	2710.08	47
H5	35.5	3523.57	1959.39	41
H8	-1396.47	4481.85	1690.11	47
H9	-1964.18	5311.76	888.57	59

Supplementary Table 9 Hydrogen Atom Coordinates (Å×10^4) and Isotropic Displacement Parameters (Å^2×10^3) for 3w.

Atom	*x*	*y*	*z*	U(eq)
H10	-796.42	6470.98	647.52	58
H11	985.01	6775.32	1198.64	45
H16	401.82	5574.31	4022.25	38
H17	744.65	6919.01	4330.25	44
H18	1686.6	7855.98	3672.78	41
H19	2251.08	7436.88	2706.85	37
H20	3093.02	5966.67	1471.42	34
H22	5174.96	6510.09	1414.71	45
H23	7131.28	6638.3	1831.73	54
H26	3826.94	5831.62	3041.13	37
H29	6488.34	8633.08	2151.14	42
H31	2303.31	9149.96	2428.88	54
H32	3645.63	9264.47	3243.56	44
H33	6964.25	9111.66	3114.48	36
H36	8126.37	8202.27	3835.91	50
H37	9976.01	8389.9	4348.01	63
H38	10141.55	9416.88	5072.01	64
H39	8479.71	10265.97	5279.19	55
H42	7557.55	11342.34	4815.58	53
H43	6154.52	12398.17	4986.5	64
H44	4049.73	12095.58	5050.78	64
H45	3362.96	10759.65	4905.53	51
H48	3239.46	9492.52	5352.12	46
H49	2437.93	8173.75	5277.95	54
H50	3209.02	7261.2	4552.15	54

Supplementary Table 9 Hydrogen Atom Coordinates (Å×10^4) and Isotropic Displacement Parameters (Å^2×10^3) for 3w.

Atom	*x*	*y*	*z*	U(eq)
H51	4755.05	7690.47	3896.46	45

Number of restraints - 0, number of constraints - unknown.

Details:

1. Fixed Uiso

At 1.2 times of:

All C(H) groups

2.a Aromatic/amide H refined with riding coordinates:

C2(H2), C3(H3), C4(H4), C5(H5), C8(H8), C9(H9), C10(H10), C11(H11), C16(H16),

C17(H17), C18(H18), C19(H19), C20(H20), C22(H22), C23(H23), C26(H26), C29(H29),

C31(H31), C32(H32), C33(H33), C36(H36), C37(H37), C38(H38), C39(H39),

C42(H42), C43(H43), C44(H44), C45(H45), C48(H48), C49(H49), C50(H50), C51(H51)

checkCIF/PLATON report

Structure factors have been supplied for datablock(s) 2-34-1

THIS REPORT IS FOR GUIDANCE ONLY. IF USED AS PART OF A REVIEW PROCEDURE FOR PUBLICATION, IT SHOULD NOT REPLACE THE EXPERTISE OF AN EXPERIENCED CRYSTALLOGRAPHIC REFEREE.

No syntax errors found. CIF dictionary Interpreting this report

Datablock: 2-34-1

```
Bond precision:    C-C = 0.0049 A          Wavelength=1.54184

Cell:               a=10.8510(3)      b=16.2553(4)      c=22.1782(4)
                    alpha=90          beta=90           gamma=90
Temperature:        150 K

                  Calculated                  Reported
Volume            3911.93(16)                 3911.93(16)
Space group       P 21 21 21                  P 21 21 21
Hall group        P 2ac 2ab                   P 2ac 2ab
Moiety formula    C26 H16 Cl2                 C26 H16 Cl2
Sum formula       C26 H16 Cl2                 C26 H16 Cl2
Mr                399.29                      399.29
Dx,g cm-3         1.356                       1.356
Z                 8                           8
Mu (mm-1)         3.032                       3.032
F000              1648.0                      1648.0
F000'             1657.47
h,k,lmax          13,20,27                    13,20,27
Nref              7945[ 4432]                 7723
Tmin,Tmax         0.654,0.695                 0.323,1.000
Tmin'             0.586

Correction method= # Reported T Limits: Tmin=0.323 Tmax=1.000
AbsCorr = MULTI-SCAN

Data completeness= 1.74/0.97        Theta(max)= 74.004

                                                  wR2(reflections)=
R(reflections)= 0.0464( 7264)                     0.1246( 7723)

S = 1.039                  Npar= 505
```

The following ALERTS were generated. Each ALERT has the format
test-name_ALERT_alert-type_alert-level.
Click on the hyperlinks for more details of the test.

Alert level C

PLAT250_ALERT_2_C Large U3/U1 Ratio for Average U(i,j) Tensor	2.1	Note
PLAT340_ALERT_3_C Low Bond Precision on C-C Bonds	0.00488	Ang.

Alert level G

PLAT912_ALERT_4_G Missing # of FCF Reflections Above STh/L= 0.600	67	Note
PLAT941_ALERT_3_G Average HKL Measurement Multiplicity	3.5	Low
PLAT978_ALERT_2_G Number C-C Bonds with Positive Residual Density.	0	Info

0 **ALERT level A** = Most likely a serious problem - resolve or explain
0 **ALERT level B** = A potentially serious problem, consider carefully
2 **ALERT level C** = Check. Ensure it is not caused by an omission or oversight
3 **ALERT level G** = General information/check it is not something unexpected

0 ALERT type 1 CIF construction/syntax error, inconsistent or missing data
2 ALERT type 2 Indicator that the structure model may be wrong or deficient
2 ALERT type 3 Indicator that the structure quality may be low
1 ALERT type 4 Improvement, methodology, query or suggestion
0 ALERT type 5 Informative message, check

Datablock 2-34-1 - ellipsoid plot

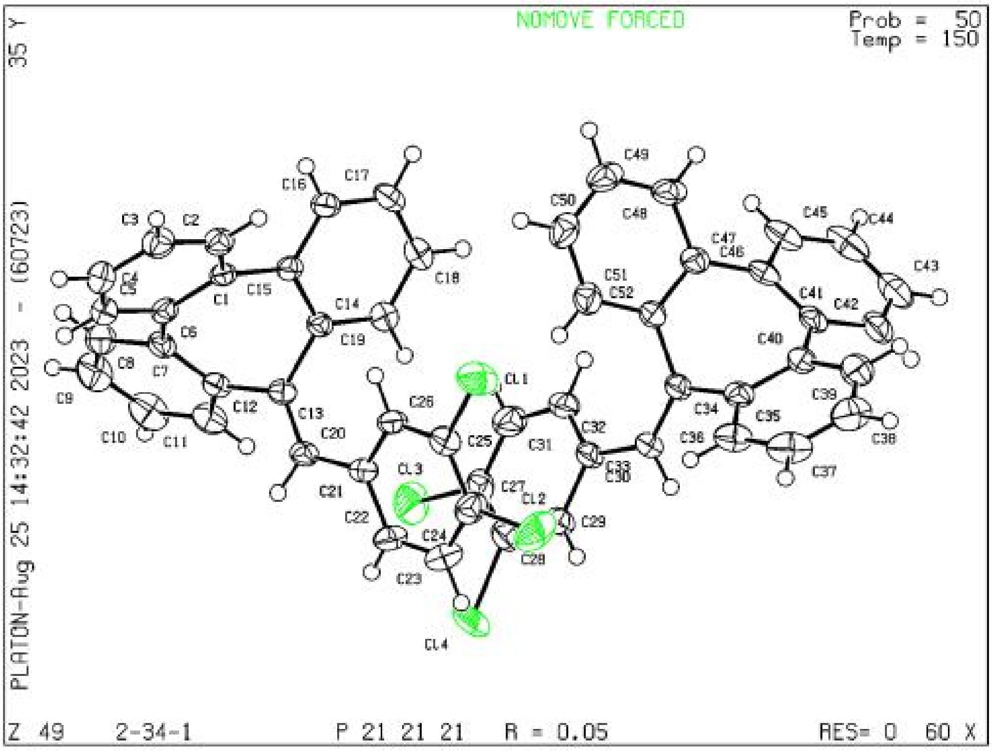

Supplementary Fig. 1 Single crystal structure of **3w** (CCDC 2302555)

11. Rotational barriers

The enantiomerisation barrier, corresponding to the barrier to rotation for the following atropisomers, was obtained by kinetic of racemisation of an enantiomer.The slope of the firstorder kinetic line gives the racemisation constant (kracemisation = 2×kenantiomerisation). Eyring equation gives the enantiomerisation barrier (ΔG‡enaniomerization) from enantiomerisation constant (kenantiomerisation), R = 8.31451 J.K^{-1} mol^{-1}, h =6.62608×10^{-34} Js and k_B =1.38066×10^{-23} J/K. Reactions were conducted at 5 mg/mL concentration. Enantiomeric excess values were determined by chiral HPLC.

$$\Delta G^{\ddagger}\text{enaniomerization} = RT \times \ln \frac{k_B \times T}{h \times k_{enantiomerisation}}$$

Racemization of **3a** in m-Xylene at 140 °C

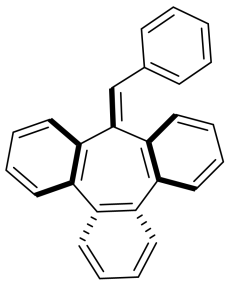

3a

ΔG‡= 31.70 kcal/mol

(m-Xylene, 140 °C)

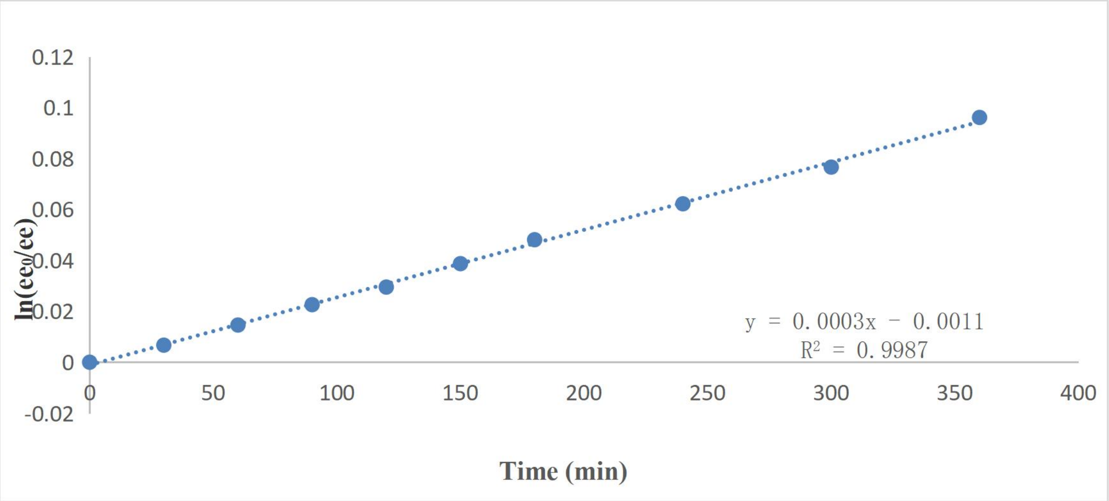

Kracemisation = 0.0003 min^{-1}

Kenantiomerisation = 0.00015 min^{-1}

ΔG‡enaniomerization= 31.70 kcal/mol

Time (min)	ee (%)	ln(ee0/ee)
0	89.5	0
30	88.9	0.0067
60	88.2	0.0146
90	87.5	0.0226
120	86.9	0.0295
150	86.1	0.0387
180	85.3	0.0481
240	84.1	0.0622
300	82.9	0.0766
360	81.3	0.0961
420	80.1	0.111
480	78.7	0.1286

Racemization of **3a** in m-Xylene at 150 °C

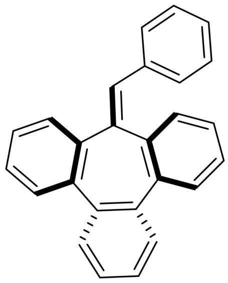

3a

ΔG‡= 31.1 kcal/mol

(m-Xylene, 150 °C)

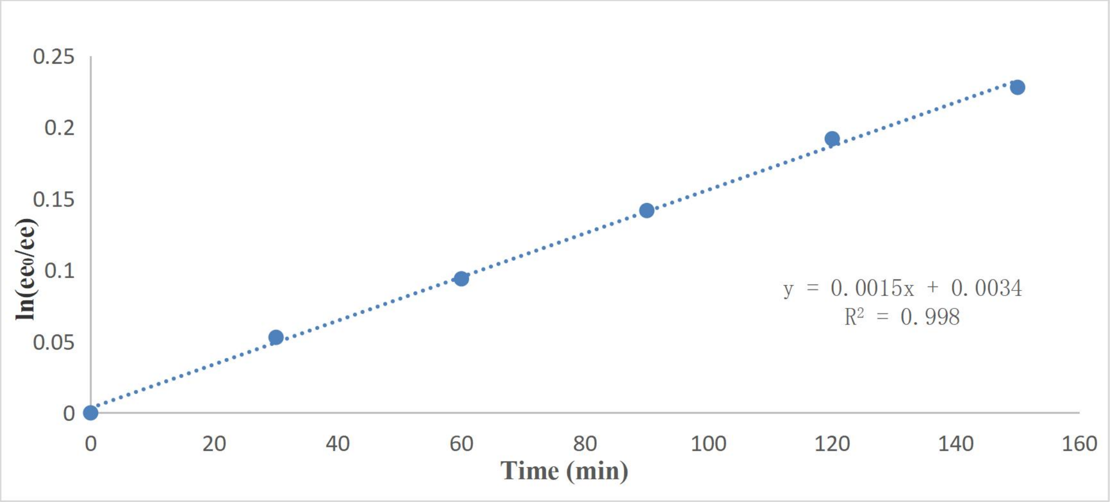

Kracemisation = 0.0015 min^{-1}

Kenantiomerisation = 0.00075 min^{-1}

ΔG‡enaniomerization= 31.1 kcal/mol

Time (min)	ee (%)	ln(ee0/ee)
0	89.5	0
30	84.7	0.0529
60	81.3	0.0939
90	77.5	0.1417
120	73.7	0.1920
150	71.1	0.2280

Racemization of **3a** in toluene at 120 °C

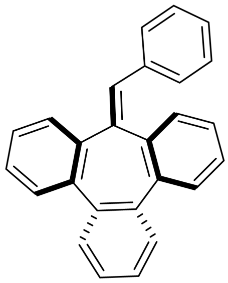

3a

ΔG‡ = 29.9 kcal/mol

(toluene, 120 °C)

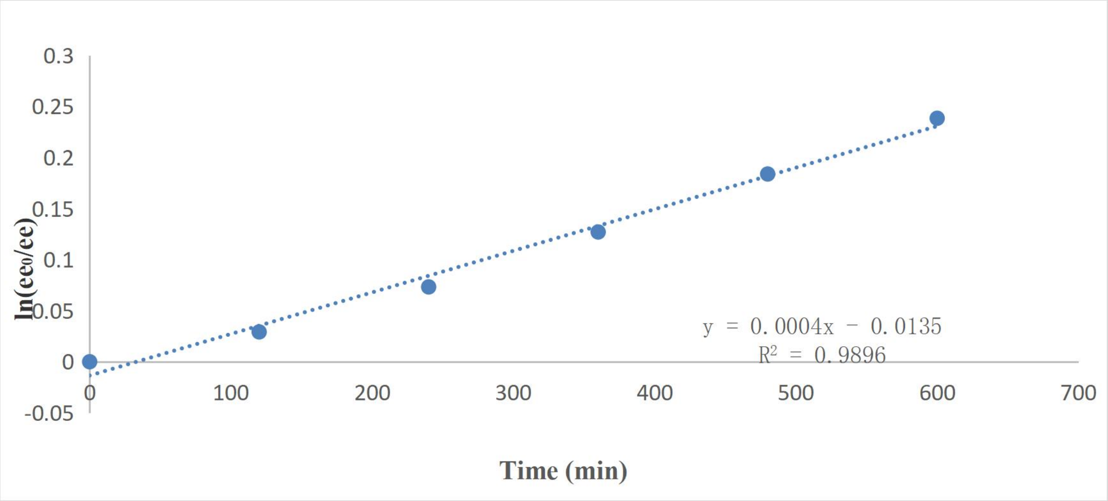

Kracemisation = 0.0004 min^{-1}

Kenantiomerisation = 0.0002 min^{-1}

ΔG‡enaniomerization= 29.9 kcal/mol

Time (min)	ee (%)	ln(ee0/ee)
0	90.5	0
120	90.5	0.0292
240	87.9	0.0733
360	84.1	0.1271
480	79.7	0.1839
600	75.3	0.2385

Racemization of **3al** in toluene at 120 °C

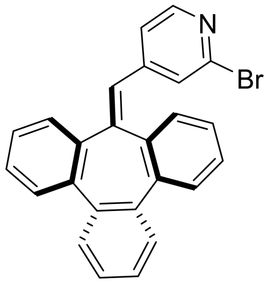

3al

ΔG‡= 30.10 kcal/mol

(toluene, 120 °C)

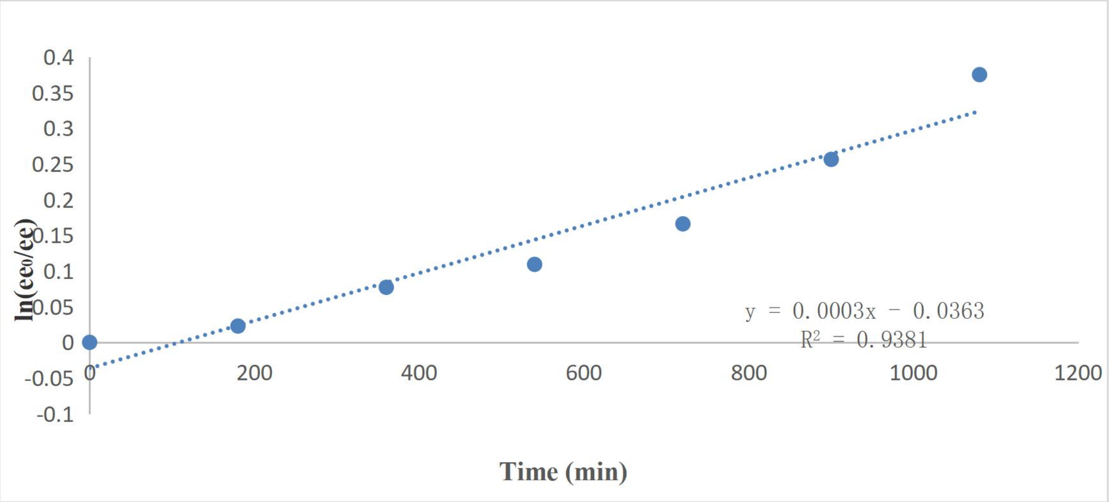

Kracemisation = 0.0003 min^{-1}

Kenantiomerisation = 0.00015 min^{-1}

ΔG‡enaniomerization= 30.10 kcal/mol

Time (min)	ee (%)	ln(ee0/ee)
0	88.9	0
180	85.9	0.0228
360	82.3	0.0771
540	79.7	0.1092
720	75.3	0.166
900	68.8	0.2563
1080	61.1	0.375

Enantiomeric conversion half-life calculation

The Eyring Equation relates the activation free energy and rate constant:

$$k = \kappa \frac{k_B T}{h} e^{-\frac{\Delta G^{\neq}}{RT}} \quad (1)$$

(1) In this equation, $\Delta G^{\ddagger}$ is the Gibbs energy of activation, κ is the transmission coefficient, k_B is Boltzmann's constant, and h is Planck's constant. The transmission coefficient is often assumed to be equal to one as it reflects what fraction of the flux through the transition state proceeds to the product without recrossing the transition state.

The epimerization of atropoisomer is a first order reaction, which makes the half-life only relates to the reaction rate constant:

$$t_{1/2} = \ln(2)/k \quad (2)$$

The $\Delta G^{\ddagger}$ of **3a** at 298 K was estimate 30.9 kcal/mol. Based on Equations 1 and 2, we calculated the half-life of **3a**, $t_{1/2}$ = 162 years.

Racemization of **5** in toluene at 120 °C

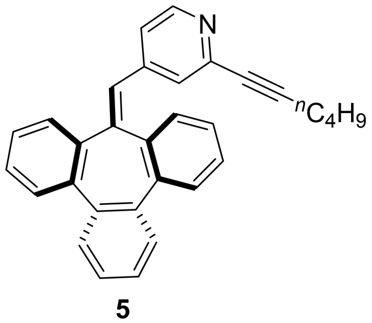

5

$\Delta G^{\ddagger}$= 31.00 kcal/mol

(toluene, 120 °C)

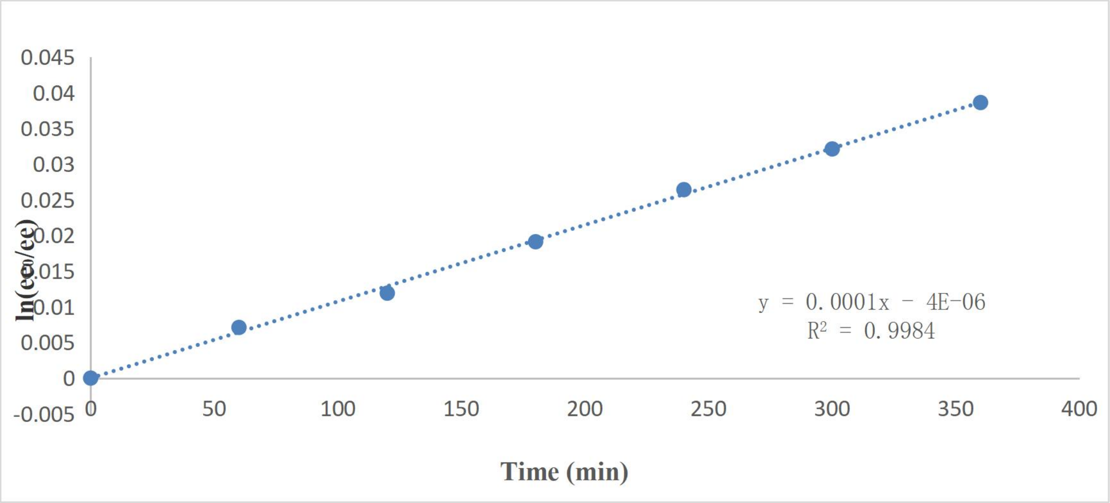

Kracemisation = 0.0001 min^{-1}

Kenantiomerisation = 0.00005 min^{-1}

ΔG‡enaniomerization= 31.00 kcal/mol

Time (min)	ee (%)	ln(ee0/ee)
0	84.5	0
60	83.9	0.0071
120	83.5	0.0119
180	82.9	0.0191
240	82.3	0.0264
300	81.8	0.0321
360	81.3	0.0386

Racemization of **3a** in CH_3CN at 25 °C (370 nm)

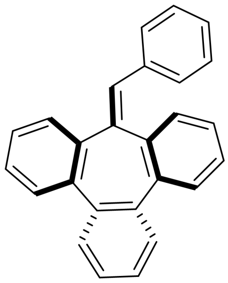

3a

Time (min)	ee (%)
0	89.5
30	86.1
60	82.1

90	78.3
120	74.5
180	67.5
240	60.7
300	54.1
360	48.0
420	41.9
480	36.3

Racemization of **3a** in m-Xylene at 140 °C (Trifluoroacetic acid)

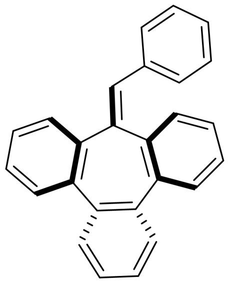

3a

ΔG‡= 30.1 kcal/mol

(m-Xylene, 140 °C)

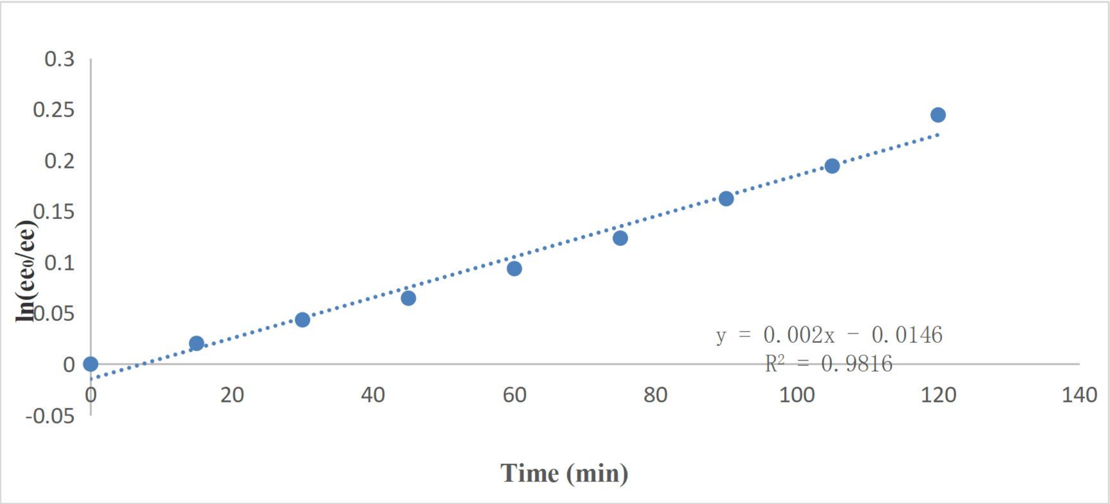

Kracemisation = 0.002 min^{-1}

Kenantiomerisation = 0.001 min^{-1}

ΔG‡enaniomerization= 30.10 kcal/mol

Time (min)	ee (%)	ln(ee0/ee)
0	89.5	0
15	87.7	0.0203
30	85.7	0.0434
45	83.9	0.0646
60	81.5	0.0936

75	79.1	0.1235
90	76.1	0.1622
105	73.7	0.1942
120	70.1	0.2443
180	57.7	
240	45.5	
300	38.7	

12. HRMS of all substrates and products

Ib HRMS: (ESI) m/z: $[M+H]^+$ Calcd for $C_{25}H_{17}O$ 333.1274; Found 333.1271.

Monoisotopic Mass, Even Electron Ions
895 formula(e) evaluated with 1 results within limits (up to 50 best isotopic matches for each mass)
Elements Used:
C: 25-25 H: 17-17 N: 0-200 O: 0-200 Na: 0-1 K: 0-1
9
231003-1-441-2-33 14 (0.145)

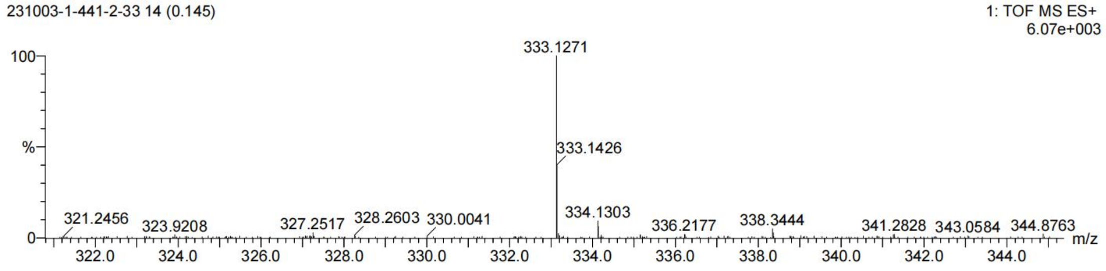

Minimum:				-1.5				
Maximum:		5.0	10.0	50.0				
Mass	Calc. Mass	mDa	PPM	DBE	i-FIT	Norm	Conf(%)	Formula
333.1271	333.1279	-0.8	-2.4	17.5	213.5	n/a	n/a	C25 H17 O

Ic HRMS: (ESI) m/z: $[M+H]^+$ Calcd for $C_{25}H_{16}OCl$ 367.0884; Found 367.0891.

Monoisotopic Mass, Even Electron Ions
1217 formula(e) evaluated with 1 results within limits (up to 50 best isotopic matches for each mass)
Elements Used:
C: 25-25 H: 16-16 N: 0-200 O: 0-200 Na: 0-1 Cl: 1-3
9
231003-1-441-2-34 11 (0.119)

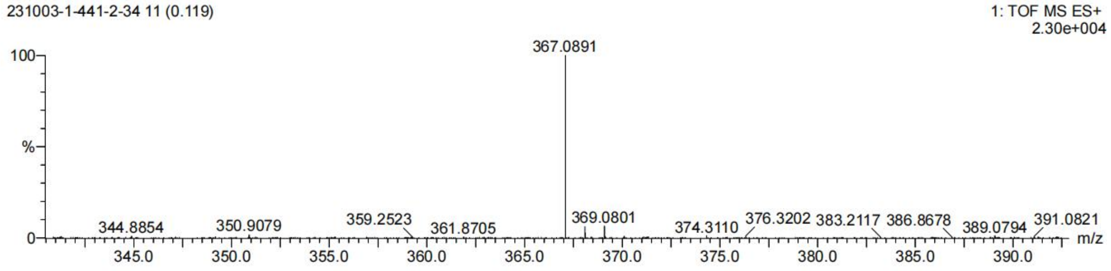

Minimum:				-1.5				
Maximum:		5.0	10.0	50.0				
Mass	Calc. Mass	mDa	PPM	DBE	i-FIT	Norm	Conf(%)	Formula
367.0891	367.0890	0.1	0.3	17.5	223.0	n/a	n/a	C25 H16 O Cl

Id HRMS: (ESI) m/z: $[M+H]^+$ Calcd for $C_{27}H_{21}O$ 361.1587 ; Found 361.1591 .

Monoisotopic Mass, Even Electron Ions
586 formula(e) evaluated with 1 results within limits (up to 50 best isotopic matches for each mass)
Elements Used:
C: 27-27 H: 21-21 N: 0-200 O: 0-200 Na: 0-1
9
231003-1-441-2-37 15 (0.154) 1: TOF MS ES+
2.20e+005

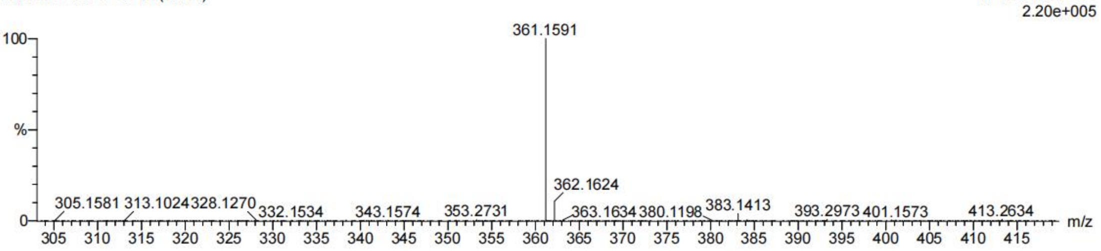

Minimum:				-1.5
Maximum:		5.0	10.0	50.0

Mass	Calc. Mass	mDa	PPM	DBE	i-FIT	Norm	Conf(%)	Formula
361.1591	361.1592	-0.1	-0.3	17.5	258.4	n/a	n/a	C27 H21 O

Ie HRMS: (ESI) m/z: $[M+Na]^+$ Calcd for $C_{26}H_{15}F_3O_2Na$ 439.0916; Found 439.0917.

Monoisotopic Mass, Even Electron Ions
121 formula(e) evaluated with 1 results within limits (up to 50 best isotopic matches for each mass)
Elements Used:
C: 26-26 H: 15-15 O: 0-100 Na: 0-1 F: 1-3

32
240308-14-535-2-5 14 (0.087) 1: TOF MS ES+
3.05e+004

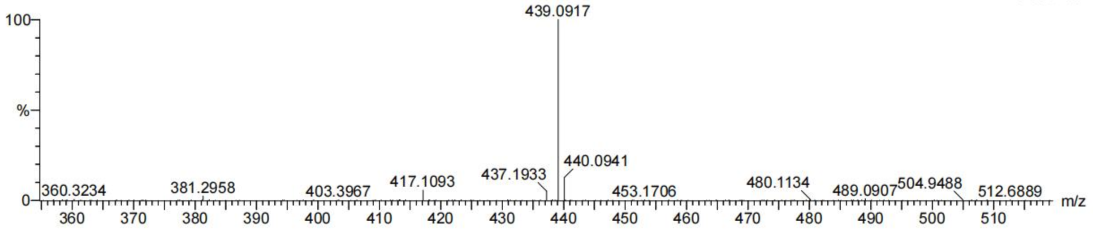

Minimum:				-1.5
Maximum:		5.0	10.0	50.0

Mass	Calc. Mass	mDa	PPM	DBE	i-FIT	Norm	Conf(%)	Formula
439.0917	439.0922	-0.5	-1.1	17.5	51.5	n/a	n/a	C26 H15 O2 Na F3

If HRMS: (ESI) m/z: $[M+H]^+$ Calcd for $C_{31}H_{21}O$ 409.1587; Found 409.1593.

Monoisotopic Mass, Even Electron Ions
47 formula(e) evaluated with 1 results within limits (up to 50 best isotopic matches for each mass)
Elements Used:
C: 31-31 H: 21-21 O: 0-100 Na: 0-1
32
240308-14-535-2-6 14 (0.087) 1: TOF MS ES+
3.39e+003

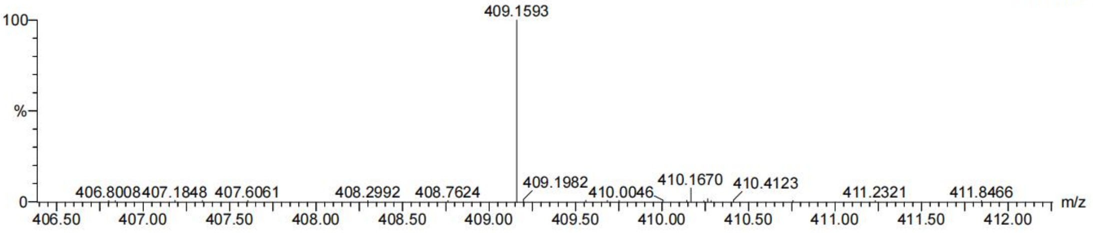

Minimum:				-1.5
Maximum:		5.0	10.0	50.0

Mass	Calc. Mass	mDa	PPM	DBE	i-FIT	Norm	Conf(%)	Formula
409.1593	409.1592	0.1	0.2	21.5	97.5	n/a	n/a	C31 H21 O

1a HRMS: (ESI) m/z: $[M+Na]^+$ Calcd for $C_{26}H_{20}N_2O_2SNa$ 447.1138; Found 447.1138.

Monoisotopic Mass, Even Electron Ions
1373 formula(e) evaluated with 1 results within limits (up to 50 best isotopic matches for each mass)
Elements Used:
C: 26-26 H: 20-20 N: 0-200 O: 0-200 Na: 0-1 S: 1-2

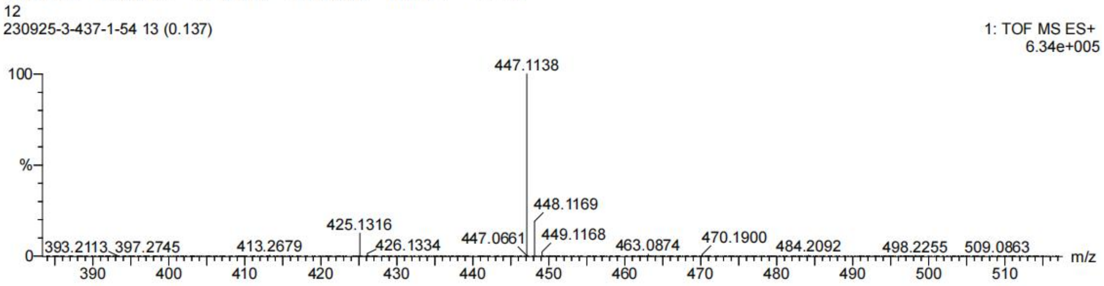

Minimum:				-1.5				
Maximum:		5.0	10.0	50.0				

Mass	Calc. Mass	mDa	PPM	DBE	i-FIT	Norm	Conf(%)	Formula
447.1138	447.1143	-0.5	-1.1	17.5	192.1	n/a	n/a	C26 H20 N2 O2 Na S

1b HRMS: (ESI) m/z: $[M+Na]^+$ Calcd for $C_{32}H_{24}N_2O_2SNa$ 523.1451; Found 523.1453.

Monoisotopic Mass, Even Electron Ions
1959 formula(e) evaluated with 1 results within limits (up to 50 best isotopic matches for each mass)
Elements Used:
C: 32-32 H: 24-24 N: 0-200 O: 0-200 Na: 0-1 S: 1-2

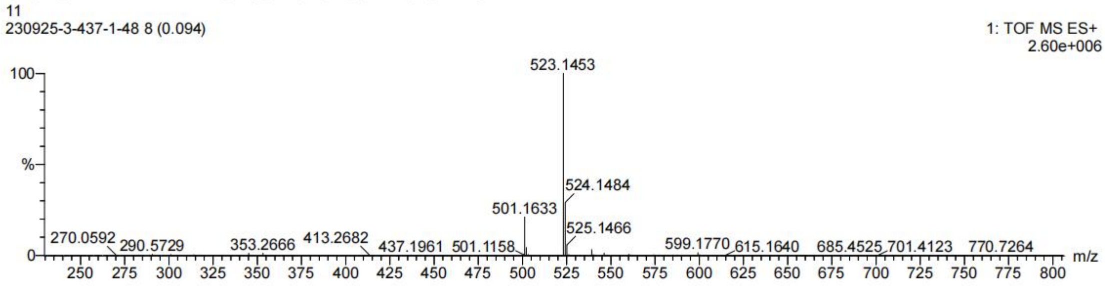

Minimum:				-1.5				
Maximum:		5.0	10.0	50.0				

Mass	Calc. Mass	mDa	PPM	DBE	i-FIT	Norm	Conf(%)	Formula
523.1453	523.1456	-0.3	-0.6	21.5	320.1	n/a	n/a	C32 H24 N2 O2 Na S

1c HRMS: (ESI) m/z: $[M+Na]^+$ Calcd for $C_{32}H_{23}ClN_2O_2SNa$ 557.1061; Found 557.1069.

Monoisotopic Mass, Even Electron Ions
3671 formula(e) evaluated with 1 results within limits (up to 50 best isotopic matches for each mass)
Elements Used:
C: 32-32 H: 23-23 N: 0-200 O: 0-200 Na: 0-1 S: 1-2 Cl: 1-2

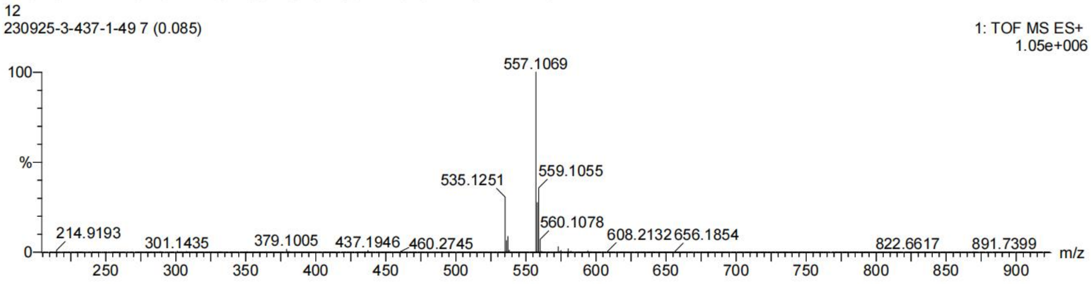

Minimum:				-1.5				
Maximum:		5.0	10.0	50.0				

Mass	Calc. Mass	mDa	PPM	DBE	i-FIT	Norm	Conf(%)	Formula
557.1069	557.1066	0.3	0.5	21.5	218.5	n/a	n/a	C32 H23 N2 O2 Na S Cl

1d HRMS: (ESI) m/z: $[M+Na]^+$ Calcd for $C_{34}H_{28}N_2O_2SNa$ 551.1764; Found 551.1765.

Monoisotopic Mass, Even Electron Ions
2200 formula(e) evaluated with 1 results within limits (up to 50 best isotopic matches for each mass)
Elements Used:
C: 34-34 H: 28-28 N: 0-200 O: 0-200 Na: 0-1 S: 1-2

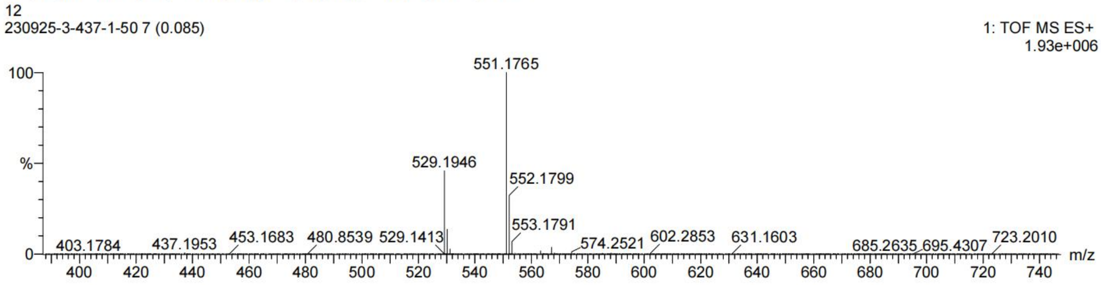

Minimum:				-1.5				
Maximum:		5.0	10.0	50.0				

Mass	Calc. Mass	mDa	PPM	DBE	i-FIT	Norm	Conf(%)	Formula
551.1765	551.1769	-0.4	-0.7	21.5	239.7	n/a	n/a	C34 H28 N2 O2 Na S

1e HRMS: (ESI) m/z: $[M+Na]^+$ Calcd for $C_{33}H_{23}F_3N_2O_3SNa$ 607.1274; Found 607.1276.

Monoisotopic Mass, Even Electron Ions
2171 formula(e) evaluated with 1 results within limits (up to 50 best isotopic matches for each mass)
Elements Used:
C: 33-33 H: 23-23 N: 0-200 O: 0-200 Na: 0-1 S: 1-2 F: 3-3

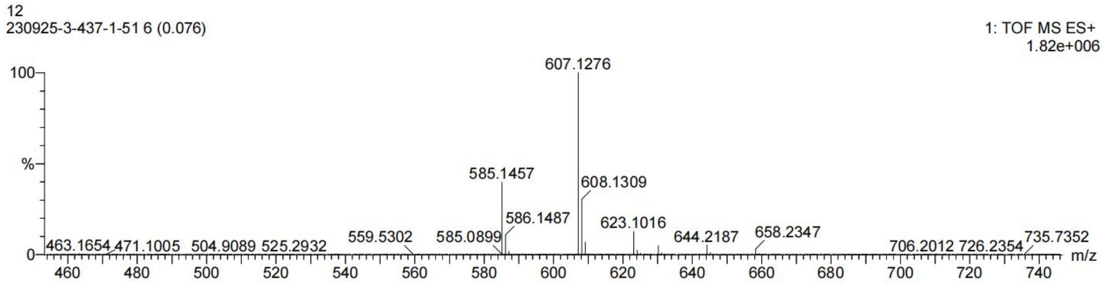

Minimum:				-1.5				
Maximum:		5.0	10.0	50.0				

Mass	Calc. Mass	mDa	PPM	DBE	i-FIT	Norm	Conf(%)	Formula
607.1276	607.1279	-0.3	-0.5	21.5	311.1	n/a	n/a	C33 H23 N2 O3 Na S F3

1f HRMS: (ESI) m/z: $[M+Na]^+$ Calcd for $C_{38}H_{28}N_2O_2SNa$ 599.1764; Found 599.1773.

Monoisotopic Mass, Even Electron Ions
2654 formula(e) evaluated with 1 results within limits (up to 50 best isotopic matches for each mass)
Elements Used:
C: 38-38 H: 28-28 N: 0-100 O: 0-100 Na: 0-1 S: 1-2

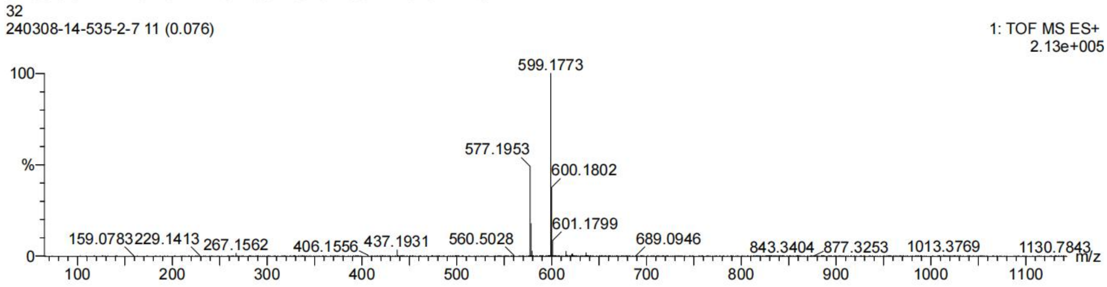

Minimum:				-1.5				
Maximum:		5.0	10.0	50.0				

Mass	Calc. Mass	mDa	PPM	DBE	i-FIT	Norm	Conf(%)	Formula
599.1773	599.1769	0.4	0.7	25.5	70.8	n/a	n/a	C38 H28 N2 O2 Na S

1g HRMS: (ESI) m/z: $[M+Na]^+$ Calcd for $C_{26}H_{19}ClN_2O_2SNa$ 481.0748; Found 481.0749.

Monoisotopic Mass, Even Electron Ions
4923 formula(e) evaluated with 1 results within limits (up to 50 best isotopic matches for each mass)
Elements Used:
C: 26-26 H: 19-19 N: 0-100 O: 0-100 Na: 0-1 S: 1-2 Cl: 1-5
32
240308-14-535-2-8 11 (0.076) 1: TOF MS ES+
1.28e+005

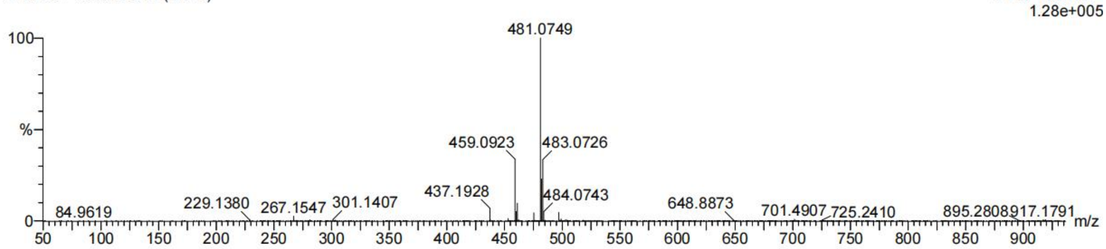

Minimum:				-1.5				
Maximum:		5.0	10.0	50.0				
Mass	Calc. Mass	mDa	PPM	DBE	i-FIT	Norm	Conf(%)	Formula
481.0749	481.0753	-0.4	-0.8	17.5	76.0	n/a	n/a	C26 H19 N2 O2 Na S Cl

1h HRMS: (ESI) m/z: $[M+Na]^+$ Calcd for $C_{28}H_{20}N_4O_2SNa$ 499.1199; Found 499.1204.

Monoisotopic Mass, Even Electron Ions
1726 formula(e) evaluated with 1 results within limits (up to 50 best isotopic matches for each mass)
Elements Used:
C: 28-28 H: 20-20 N: 0-200 O: 0-200 Na: 0-1 S: 1-2
12
230925-3-437-1-52 6 (0.076) 1: TOF MS ES+
4.04e+006

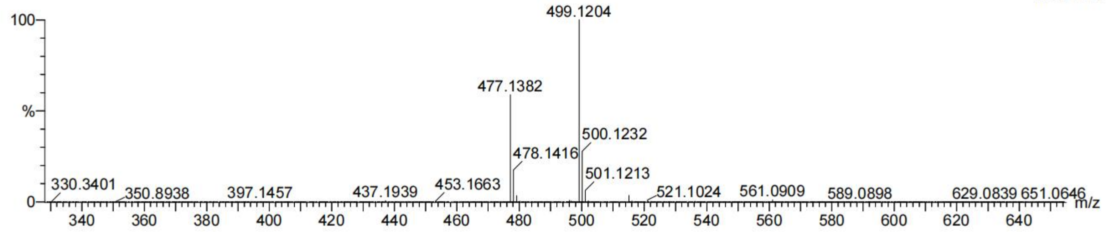

Minimum:				-1.5				
Maximum:		5.0	10.0	50.0				
Mass	Calc. Mass	mDa	PPM	DBE	i-FIT	Norm	Conf(%)	Formula
499.1204	499.1205	-0.1	-0.2	20.5	286.5	n/a	n/a	C28 H20 N4 O2 Na S

3a HRMS: (ESI) m/z: $[M+K]^+$ Calcd for $C_{26}H_{18}K$ 369.1040; Found 369.1048.

Monoisotopic Mass, Even Electron Ions
495 formula(e) evaluated with 1 results within limits (up to 50 best isotopic matches for each mass)
Elements Used:
C: 26-26 H: 18-18 N: 0-200 O: 0-200 Na: 0-1 K: 1-1
11
230925-3-437-1-1 7 (0.085) 1: TOF MS ES+
1.58e+003

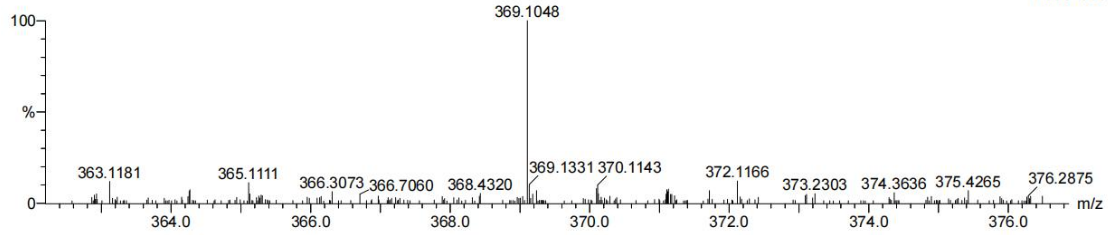

Minimum:				-1.5				
Maximum:		5.0	10.0	50.0				
Mass	Calc. Mass	mDa	PPM	DBE	i-FIT	Norm	Conf(%)	Formula
369.1048	369.1046	0.2	0.5	17.5	377.9	n/a	n/a	C26 H18 K

3b HRMS: (ESI) m/z: $[M+K]^+$ Calcd for $C_{27}H_{20}K$ 383.1197; Found 383.1200.

Monoisotopic Mass, Even Electron Ions
538 formula(e) evaluated with 1 results within limits (up to 50 best isotopic matches for each mass)
Elements Used:
C: 27-27 H: 20-20 N: 0-200 O: 0-200 Na: 0-1 K: 1-1

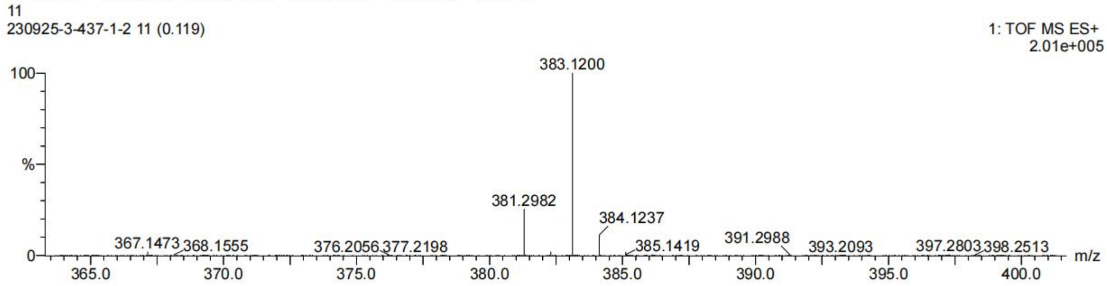

Minimum:				-1.5				
Maximum:		5.0	10.0	50.0				
Mass	Calc. Mass	mDa	PPM	DBE	i-FIT	Norm	Conf(%)	Formula
383.1200	383.1202	-0.2	-0.5	17.5	300.2	n/a	n/a	C27 H20 K

3c HRMS: (ESI) m/z: $[M+K]^+$ Calcd for $C_{32}H_{22}K$ 445.1353; Found 445.1357.

Monoisotopic Mass, Even Electron Ions
747 formula(e) evaluated with 1 results within limits (up to 50 best isotopic matches for each mass)
Elements Used:
C: 32-32 H: 22-22 N: 0-200 O: 0-200 Na: 0-1 K: 1-1

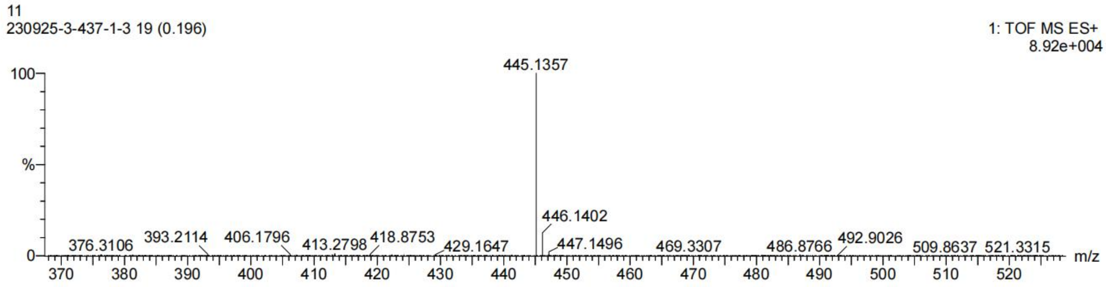

Minimum:				-1.5				
Maximum:		5.0	10.0	50.0				
Mass	Calc. Mass	mDa	PPM	DBE	i-FIT	Norm	Conf(%)	Formula
445.1357	445.1359	-0.2	-0.4	21.5	271.8	n/a	n/a	C32 H22 K

3d HRMS: (ESI) m/z: $[M+K]^+$ Calcd for $C_{26}H_{17}FK$ 387.0946; Found 387.0952.

Monoisotopic Mass, Even Electron Ions
492 formula(e) evaluated with 1 results within limits (up to 50 best isotopic matches for each mass)
Elements Used:
C: 26-26 H: 17-17 N: 0-200 O: 0-200 Na: 0-1 K: 1-1 F: 1-1

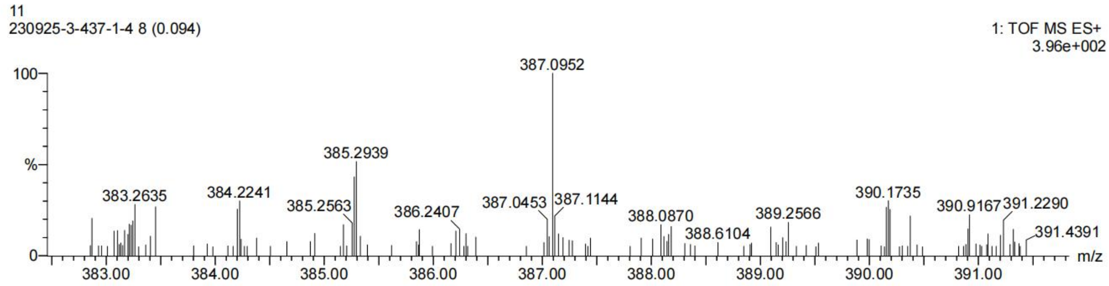

Minimum:				-1.5				
Maximum:		5.0	10.0	50.0				
Mass	Calc. Mass	mDa	PPM	DBE	i-FIT	Norm	Conf(%)	Formula
387.0952	387.0951	0.1	0.3	17.5	231.1	n/a	n/a	C26 H17 K F

3e HRMS: (ESI) m/z: $[M+K]^+$ Calcd for $C_{26}H_{17}ClK$ 403.0650; Found 403.0653.

Monoisotopic Mass, Even Electron Ions
889 formula(e) evaluated with 1 results within limits (up to 50 best isotopic matches for each mass)
Elements Used:
C: 26-26 H: 17-17 N: 0-200 O: 0-200 Na: 0-1 Cl: 1-2 K: 1-1
11
230925-3-437-1-5 9 (0.102) 1: TOF MS ES+ 7.77e+002

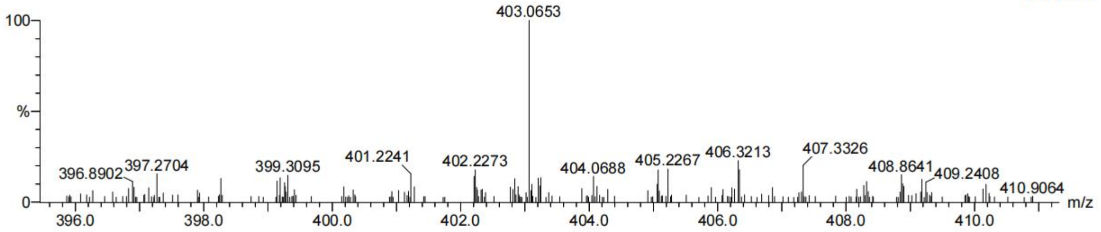

Minimum:			-1.5	
Maximum:	5.0	10.0	50.0	

Mass	Calc. Mass	mDa	PPM	DBE	i-FIT	Norm	Conf(%)	Formula
403.0653	403.0656	-0.3	-0.7	17.5	269.4	n/a	n/a	C26 H17 Cl K

3f HRMS: (ESI) m/z: $[M+K]^+$ Calcd for $C_{26}H_{17}BrK$ 447.0145; Found 447.0154.

Monoisotopic Mass, Even Electron Ions
780 formula(e) evaluated with 1 results within limits (up to 50 best isotopic matches for each mass)
Elements Used:
C: 26-26 H: 17-17 N: 0-200 O: 0-200 Na: 0-1 K: 1-1 Br: 1-2

11
230925-3-437-1-6 11 (0.119) 1: TOF MS ES+ 9.18e+002

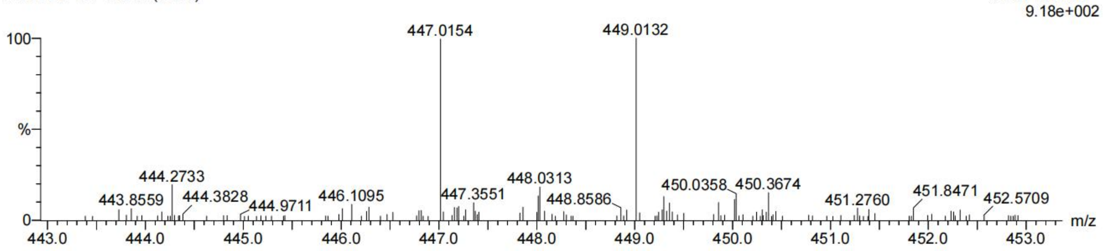

Minimum:			-1.5	
Maximum:	5.0	10.0	50.0	

Mass	Calc. Mass	mDa	PPM	DBE	i-FIT	Norm	Conf(%)	Formula
447.0154	447.0151	0.3	0.7	17.5	291.8	n/a	n/a	C26 H17 K Br

3g HRMS: (ESI) m/z: $[M+Na]^+$ Calcd for $C_{27}H_{17}NNa$ 378.1253; Found 378.1253.

Monoisotopic Mass, Even Electron Ions
634 formula(e) evaluated with 1 results within limits (up to 50 best isotopic matches for each mass)
Elements Used:
C: 27-27 H: 17-17 N: 0-200 O: 0-200 Na: 0-1
11
230925-3-437-1-7 7 (0.085) 1: TOF MS ES+ 1.32e+006

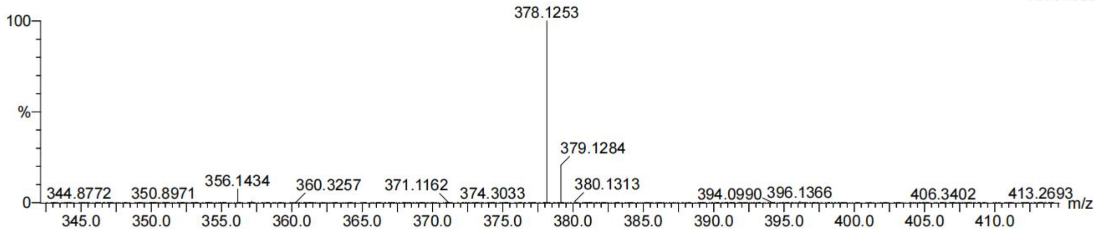

Minimum:			-1.5	
Maximum:	5.0	10.0	50.0	

Mass	Calc. Mass	mDa	PPM	DBE	i-FIT	Norm	Conf(%)	Formula
378.1253	378.1259	-0.6	-1.6	19.5	341.6	n/a	n/a	C27 H17 N Na

3h HRMS: (ESI) m/z: $[M+K]^+$ Calcd for $C_{27}H_{18}F_2OK$ 435.0957; Found 435.0966.

Monoisotopic Mass, Even Electron Ions
577 formula(e) evaluated with 1 results within limits (up to 50 best isotopic matches for each mass)
Elements Used:
C: 27-27 H: 18-18 N: 0-200 O: 0-200 Na: 0-1 F: 2-2 K: 1-1

11
230925-3-437-1-8 9 (0.102)

1: TOF MS ES+
5.91e+003

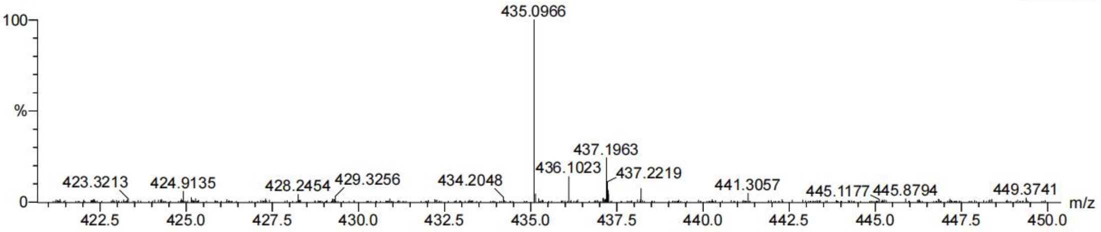

Minimum:				-1.5				
Maximum:		5.0	10.0	50.0				
Mass	Calc. Mass	mDa	PPM	DBE	i-FIT	Norm	Conf(%)	Formula
435.0966	435.0963	0.3	0.7	17.5	313.3	n/a	n/a	C27 H18 O F2 K

3i HRMS: (ESI) m/z: $[M+Na]^+$ Calcd for $C_{27}H_{20}O_2SNa$ 431.1076; Found 431.1080.

Monoisotopic Mass, Even Electron Ions
1296 formula(e) evaluated with 1 results within limits (up to 50 best isotopic matches for each mass)
Elements Used:
C: 27-27 H: 20-20 N: 0-200 O: 0-200 Na: 0-1 S: 1-2
11
230925-3-437-1-9 8 (0.094)

1: TOF MS ES+
2.09e+006

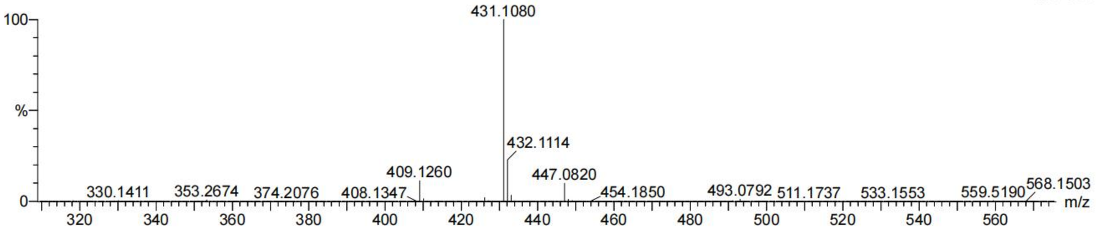

Minimum:				-1.5				
Maximum:		5.0	10.0	50.0				
Mass	Calc. Mass	mDa	PPM	DBE	i-FIT	Norm	Conf(%)	Formula
431.1080	431.1082	-0.2	-0.5	17.5	362.6	n/a	n/a	C27 H20 O2 Na S

3j HRMS: (ESI) m/z: $[M+Na]^+$ Calcd for $C_{26}H_{19}NO_2SNa$ 432.1029; Found 432.1033.

Monoisotopic Mass, Even Electron Ions
1288 formula(e) evaluated with 1 results within limits (up to 50 best isotopic matches for each mass)
Elements Used:
C: 26-26 H: 19-19 N: 0-200 O: 0-200 Na: 0-1 S: 1-2
11
230925-3-437-1-10 6 (0.076)

1: TOF MS ES+
7.32e+005

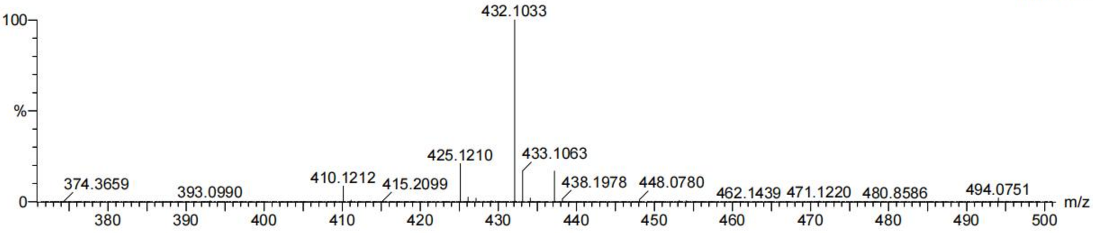

Minimum:				-1.5				
Maximum:		5.0	10.0	50.0				
Mass	Calc. Mass	mDa	PPM	DBE	i-FIT	Norm	Conf(%)	Formula
432.1033	432.1034	-0.1	-0.2	17.5	327.9	n/a	n/a	C26 H19 N O2 Na S

3k HRMS: (ESI) m/z: $[M+H]^+$ Calcd for $C_{27}H_{18}F_3$ 399.1355; Found 399.1353.

Monoisotopic Mass, Even Electron Ions
532 formula(e) evaluated with 1 results within limits (up to 50 best isotopic matches for each mass)
Elements Used:
C: 27-27 H: 18-18 N: 0-200 O: 0-200 Na: 0-1 F: 3-3

11
230925-3-437-1-11 10 (0.111) 1: TOF MS ES+ 4.21e+002

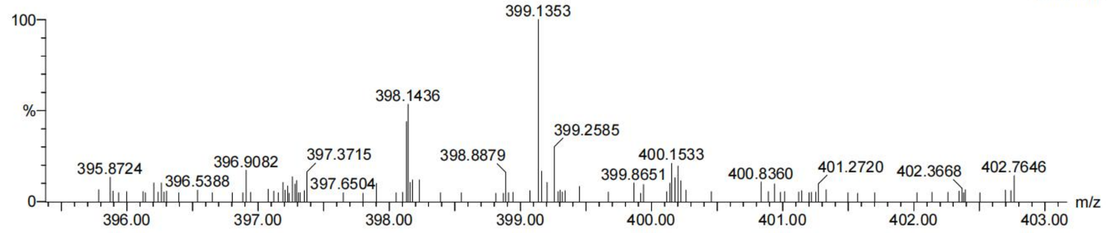

Minimum:				-1.5				
Maximum:		5.0	10.0	50.0				
Mass	Calc. Mass	mDa	PPM	DBE	i-FIT	Norm	Conf(%)	Formula
399.1353	399.1361	-0.8	-2.0	17.5	235.3	n/a	n/a	C27 H18 F3

3l HRMS: (ESI) m/z: $[M+H]^+$ Calcd for $C_{27}H_{18}F_3O$ 415.1304; Found 415.1305.

Monoisotopic Mass, Even Electron Ions
577 formula(e) evaluated with 1 results within limits (up to 50 best isotopic matches for each mass)
Elements Used:
C: 27-27 H: 18-18 N: 0-200 O: 0-200 F: 3-3 Na: 0-1
11
230925-3-437-1-12 9 (0.102) 1: TOF MS ES+ 6.61e+003

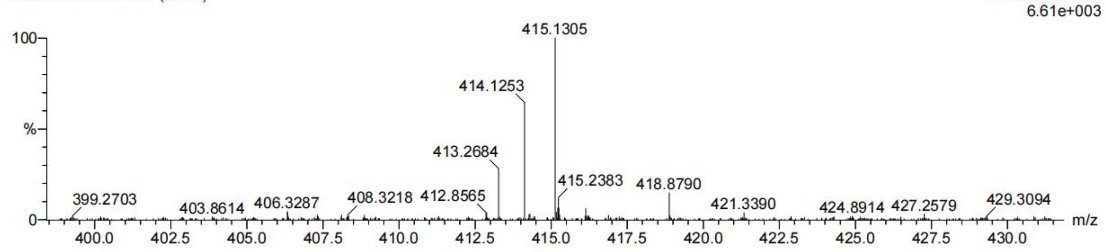

Minimum:				-1.5				
Maximum:		5.0	10.0	50.0				
Mass	Calc. Mass	mDa	PPM	DBE	i-FIT	Norm	Conf(%)	Formula
415.1305	415.1310	-0.5	-1.2	17.5	366.4	n/a	n/a	C27 H18 O F3

3m HRMS: (ESI) m/z: $[M+H]^+$ Calcd for $C_{27}H_{18}F_3S$ 431.1076; Found 431.1088.

Monoisotopic Mass, Even Electron Ions
973 formula(e) evaluated with 1 results within limits (up to 50 best isotopic matches for each mass)
Elements Used:
C: 27-27 H: 18-18 N: 0-200 O: 0-200 F: 3-3 Na: 0-1 S: 1-2
11
230925-3-437-1-13 13 (0.137) 1: TOF MS ES+ 4.17e+002

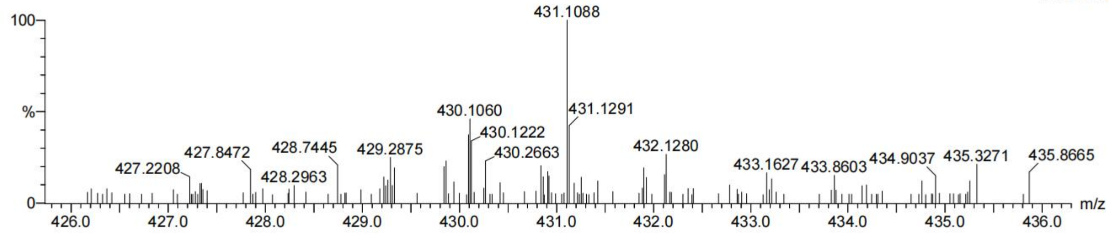

Minimum:				-1.5				
Maximum:		5.0	10.0	50.0				
Mass	Calc. Mass	mDa	PPM	DBE	i-FIT	Norm	Conf(%)	Formula
431.1088	431.1081	0.7	1.6	17.5	253.9	n/a	n/a	C27 H18 F3 S

3n HRMS: (ESI) m/z: $[M+Na]^+$ Calcd for $C_{27}H_{18}ONa$ 381.1250; Found 381.1254.

Monoisotopic Mass, Even Electron Ions
643 formula(e) evaluated with 1 results within limits (up to 50 best isotopic matches for each mass)
Elements Used:
C: 27-27 H: 18-18 N: 0-200 O: 0-200 Na: 0-1
11
230925-3-437-1-14 7 (0.085)
1: TOF MS ES+
9.87e+005

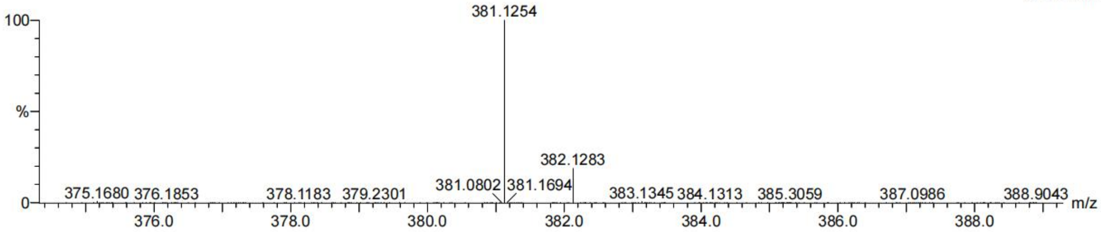

Minimum:				-1.5	
Maximum:		5.0	10.0	50.0	

Mass	Calc. Mass	mDa	PPM	DBE	i-FIT	Norm	Conf(%)	Formula
381.1254	381.1255	-0.1	-0.3	18.5	363.6	n/a	n/a	C27 H18 O Na

3o HRMS: (ESI) m/z: $[M+Na]^+$ Calcd for $C_{28}H_{20}O_2Na$ 411.1356; Found 411.1360.

Monoisotopic Mass, Even Electron Ions
737 formula(e) evaluated with 1 results within limits (up to 50 best isotopic matches for each mass)
Elements Used:
C: 28-28 H: 20-20 N: 0-200 O: 0-200 Na: 0-1
11
230925-3-437-1-15 9 (0.102)
1: TOF MS ES+
2.38e+006

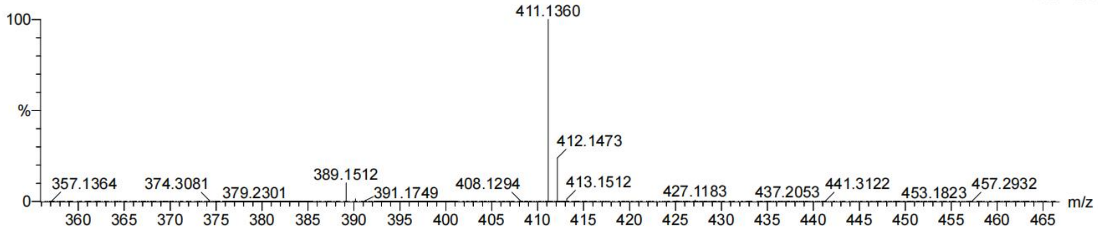

Minimum:				-1.5	
Maximum:		5.0	10.0	50.0	

Mass	Calc. Mass	mDa	PPM	DBE	i-FIT	Norm	Conf(%)	Formula
411.1360	411.1361	-0.1	-0.2	18.5	422.9	n/a	n/a	C28 H20 O2 Na

3p HRMS: (ESI) m/z: $[M+K]^+$ Calcd for $C_{26}H_{17}BrK$ 447.0145; Found 447.0154.

Monoisotopic Mass, Even Electron Ions
780 formula(e) evaluated with 1 results within limits (up to 50 best isotopic matches for each mass)
Elements Used:
C: 26-26 H: 17-17 N: 0-200 O: 0-200 Na: 0-1 Br: 1-2 K: 1-1
11
230925-3-437-1-16 11 (0.119)
1: TOF MS ES+
1.26e+003

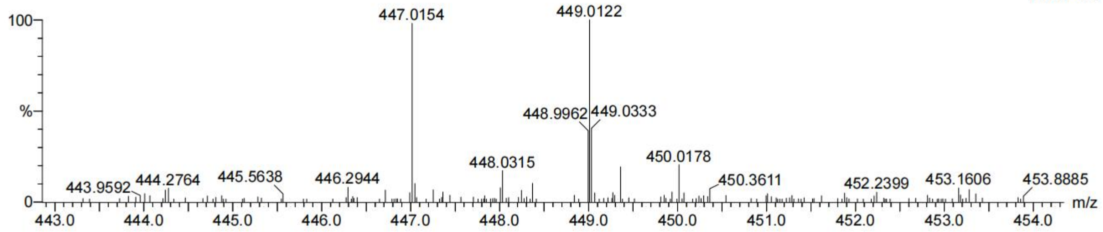

Minimum:				-1.5	
Maximum:		5.0	10.0	50.0	

Mass	Calc. Mass	mDa	PPM	DBE	i-FIT	Norm	Conf(%)	Formula
447.0154	447.0151	0.3	0.7	17.5	344.2	n/a	n/a	C26 H17 Br K

3q HRMS: (ESI) m/z: $[M+H]^+$ Calcd for $C_{26}H_{18}Cl$ 365.1092; Found 365.1097.

Monoisotopic Mass, Even Electron Ions
895 formula(e) evaluated with 1 results within limits (up to 50 best isotopic matches for each mass)
Elements Used:
C: 26-26 H: 18-18 N: 0-200 O: 0-200 Na: 0-1 Cl: 1-2
11
230925-3-437-1-17 11 (0.119) 1: TOF MS ES+ 8.84e+002

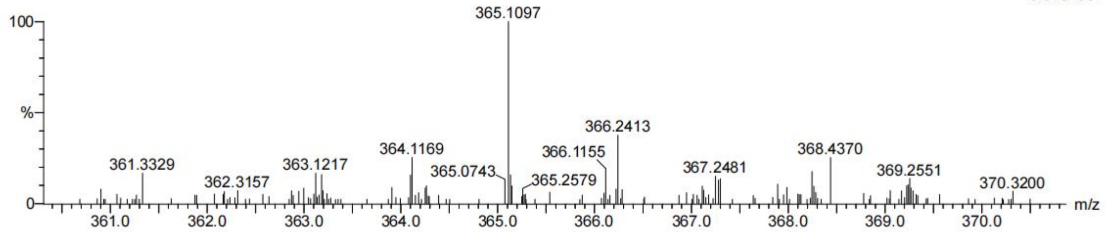

	Mass	Calc. Mass	mDa	PPM	DBE	i-FIT	Norm	Conf(%)	Formula
Minimum:					-1.5				
Maximum:			5.0	10.0	50.0				
	365.1097	365.1097	0.0	0.0	17.5	284.1	n/a	n/a	C26 H18 Cl

3r HRMS: (ESI) m/z: $[M+Na]^+$ Calcd for $C_{26}H_{17}FNa$ 371.1206; Found 371.1210.

Monoisotopic Mass, Even Electron Ions
1 formula(e) evaluated with 1 results within limits (up to 50 best isotopic matches for each mass)
Elements Used:
C: 26-26 H: 17-17 Na: 0-1 F: 1-1
11
230925-3-437-1-18 8 (0.094) 1: TOF MS ES+ 6.72e+002

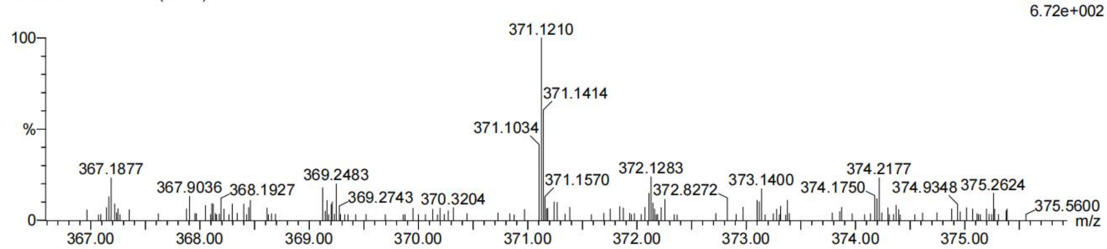

	Mass	Calc. Mass	mDa	PPM	DBE	i-FIT	Norm	Conf(%)	Formula
Minimum:					-1.5				
Maximum:			5.0	10.0	50.0				
	371.1210	371.1212	-0.2	-0.5	17.5	320.0	n/a	n/a	C26 H17 Na F

3s HRMS: (ESI) m/z: $[M+Na]^+$ Calcd for $C_{27}H_{17}NNa$ 378.1253; Found 378.1251.

Monoisotopic Mass, Even Electron Ions
634 formula(e) evaluated with 1 results within limits (up to 50 best isotopic matches for each mass)
Elements Used:
C: 27-27 H: 17-17 N: 0-200 O: 0-200 Na: 0-1
11
230925-3-437-1-19 7 (0.085) 1: TOF MS ES+ 2.71e+006

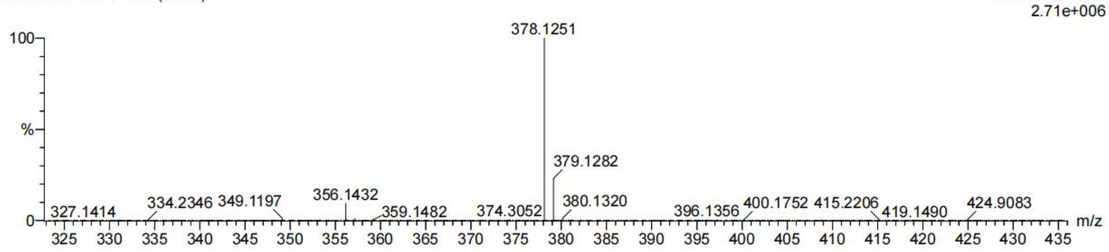

	Mass	Calc. Mass	mDa	PPM	DBE	i-FIT	Norm	Conf(%)	Formula
Minimum:					-1.5				
Maximum:			5.0	10.0	50.0				
	378.1251	378.1259	-0.8	-2.1	19.5	406.3	n/a	n/a	C27 H17 N Na

3t HRMS: (ESI) m/z: $[M+H]^+$ Calcd for $C_{27}H_{21}O$ 361.1587; Found 361.1588.

Monoisotopic Mass, Even Electron Ions
586 formula(e) evaluated with 1 results within limits (up to 50 best isotopic matches for each mass)
Elements Used:
C: 27-27 H: 21-21 N: 0-200 O: 0-200 Na: 0-1

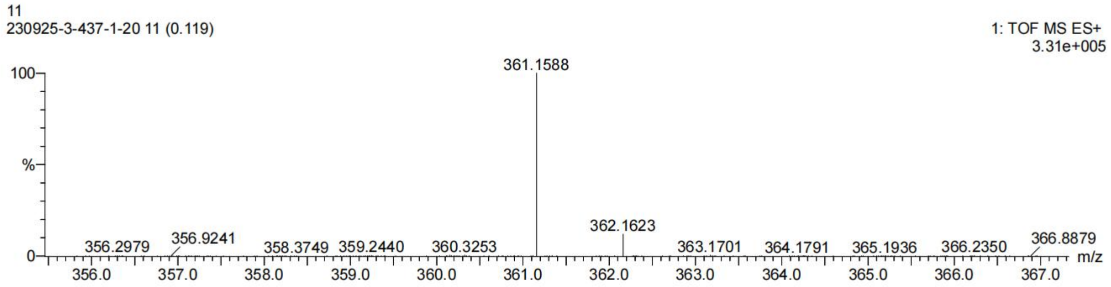

Minimum:			-1.5	
Maximum:	5.0	10.0	50.0	

Mass	Calc. Mass	mDa	PPM	DBE	i-FIT	Norm	Conf(%)	Formula
361.1588	361.1592	-0.4	-1.1	17.5	282.2	n/a	n/a	C27 H21 O

3u HRMS: (ESI) m/z: $[M+Na]^+$ Calcd for $C_{31}H_{27}NO_2Na$ 468.1934; Found 468.1932.

Monoisotopic Mass, Even Electron Ions
945 formula(e) evaluated with 1 results within limits (up to 50 best isotopic matches for each mass)
Elements Used:
C: 31-31 H: 27-27 N: 0-200 O: 0-200 Na: 0-1

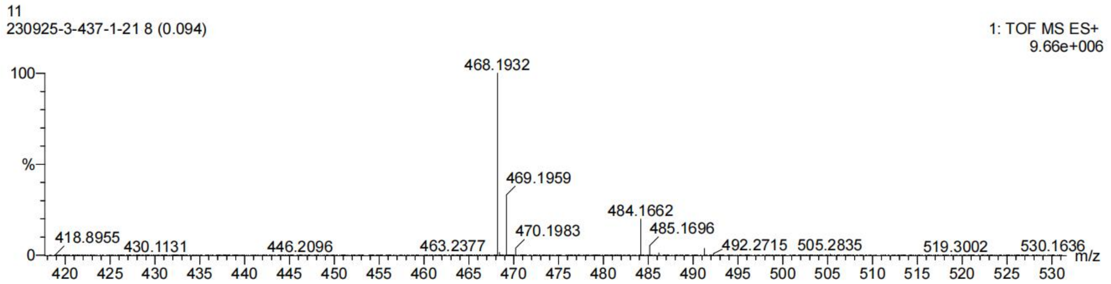

Minimum:			-1.5	
Maximum:	5.0	10.0	50.0	

Mass	Calc. Mass	mDa	PPM	DBE	i-FIT	Norm	Conf(%)	Formula
468.1932	468.1939	-0.7	-1.5	18.5	437.6	n/a	n/a	C31 H27 N O2 Na

3v HRMS: (ESI) m/z: $[M+K]^+$ Calcd for $C_{26}H_{16}ClFK$ 421.0556; Found 421.0552.

Monoisotopic Mass, Even Electron Ions
885 formula(e) evaluated with 1 results within limits (up to 50 best isotopic matches for each mass)
Elements Used:
C: 26-26 H: 16-16 N: 0-200 O: 0-200 Na: 0-1 Cl: 1-2 F: 1-1 K: 1-1

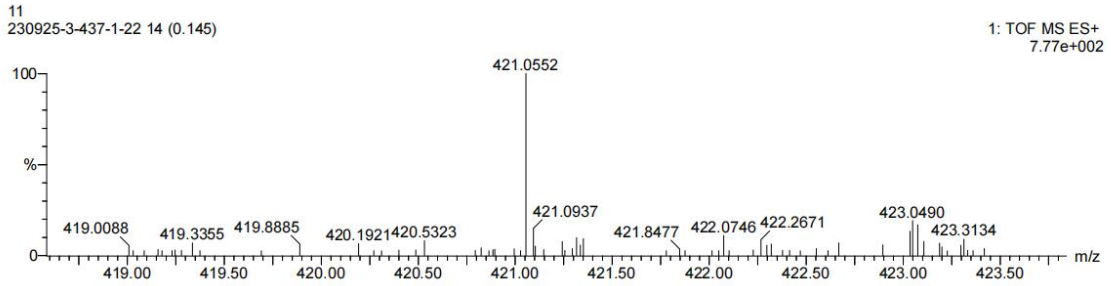

Minimum:			-1.5	
Maximum:	5.0	10.0	50.0	

Mass	Calc. Mass	mDa	PPM	DBE	i-FIT	Norm	Conf(%)	Formula
421.0552	421.0562	-1.0	-2.4	17.5	288.7	n/a	n/a	C26 H16 Cl F K

3w HRMS: (ESI) m/z: $[M+K]^+$ Calcd for $C_{27}H_{19}BrOK$ 477.0251; Found 477.0252.

Monoisotopic Mass, Even Electron Ions
1201 formula(e) evaluated with 1 results within limits (up to 50 best isotopic matches for each mass)
Elements Used:
C: 27-27 H: 19-19 N: 0-100 O: 0-100 Na: 0-1 Br: 1-4 K: 1-1

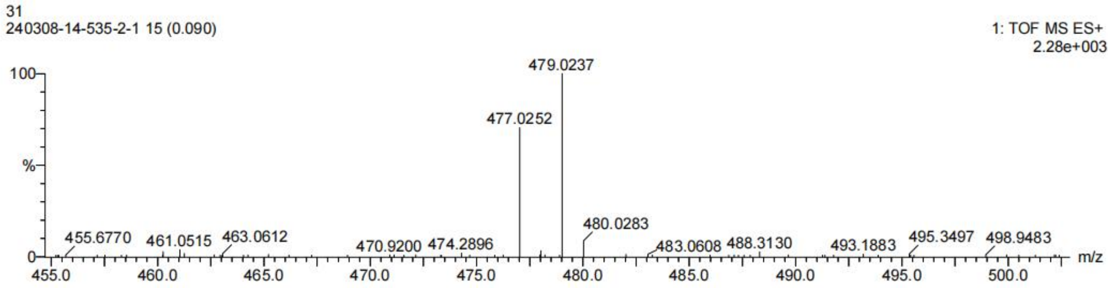

Minimum:				-1.5				
Maximum:		5.0	10.0	50.0				
Mass	Calc. Mass	mDa	PPM	DBE	i-FIT	Norm	Conf(%)	Formula
477.0252	477.0256	-0.4	-0.8	17.5	35.4	n/a	n/a	C27 H19 O Br K

3x HRMS: (ESI) m/z: $[M+K]^+$ Calcd for $C_{30}H_{20}K$ 419.1197; Found 419.1199.

Monoisotopic Mass, Even Electron Ions
656 formula(e) evaluated with 1 results within limits (up to 50 best isotopic matches for each mass)
Elements Used:
C: 30-30 H: 20-20 N: 0-100 O: 0-100 Na: 0-1 K: 1-1

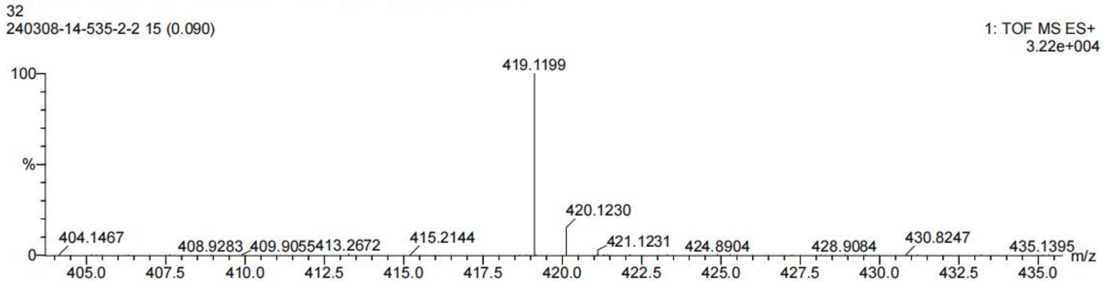

Minimum:				-1.5				
Maximum:		5.0	10.0	50.0				
Mass	Calc. Mass	mDa	PPM	DBE	i-FIT	Norm	Conf(%)	Formula
419.1199	419.1202	-0.3	-0.7	20.5	55.5	n/a	n/a	C30 H20 K

3y HRMS: (ESI) m/z: $[M+Na]^+$ Calcd for $C_{26}H_{16}Cl_2Na$ 421.0521; Found 421.0519.

Monoisotopic Mass, Even Electron Ions
1 formula(e) evaluated with 1 results within limits (up to 50 best isotopic matches for each mass)
Elements Used:
C: 26-26 H: 16-16 Na: 0-1 Cl: 1-2

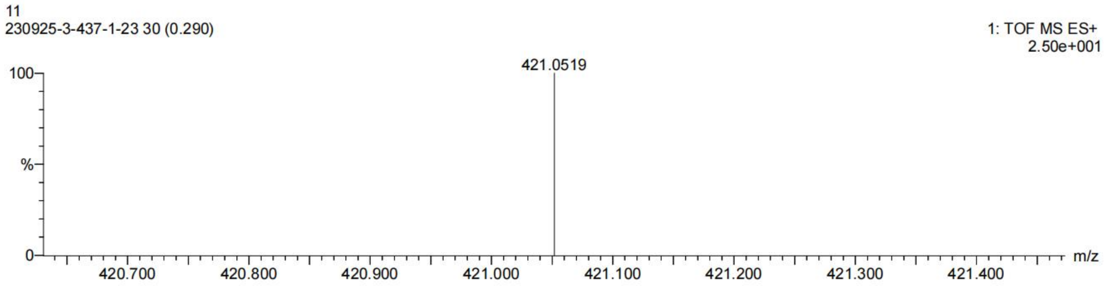

Minimum:				-1.5				
Maximum:		5.0	10.0	50.0				
Mass	Calc. Mass	mDa	PPM	DBE	i-FIT	Norm	Conf(%)	Formula
421.0519	421.0527	-0.8	-1.9	17.5	15.4	n/a	n/a	C26 H16 Na Cl2

3z HRMS: (ESI) m/z: $[M+K]^+$ Calcd for $C_{27}H_{19}FOK$ 417.1052; Found 417.1063.

Monoisotopic Mass, Even Electron Ions
41 formula(e) evaluated with 1 results within limits (up to 50 best isotopic matches for each mass)
Elements Used:
C: 27-27 H: 19-19 O: 0-200 Na: 0-1 F: 1-1 K: 1-1

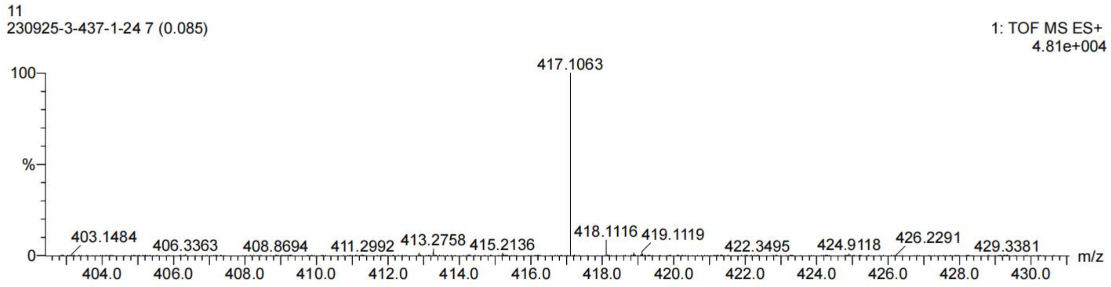

Minimum:				-1.5				
Maximum:		5.0	10.0	50.0				
Mass	Calc. Mass	mDa	PPM	DBE	i-FIT	Norm	Conf(%)	Formula
417.1063	417.1057	0.6	1.4	17.5	319.0	n/a	n/a	C27 H19 O F K

3aa HRMS: (ESI) m/z: $[M+Na]^+$ Calcd for $C_{26}H_{16}FNO_2Na$ 416.1057; Found 416.1060.

Monoisotopic Mass, Even Electron Ions
680 formula(e) evaluated with 1 results within limits (up to 50 best isotopic matches for each mass)
Elements Used:
C: 26-26 H: 16-16 N: 0-200 O: 0-200 F: 1-1 Na: 0-1

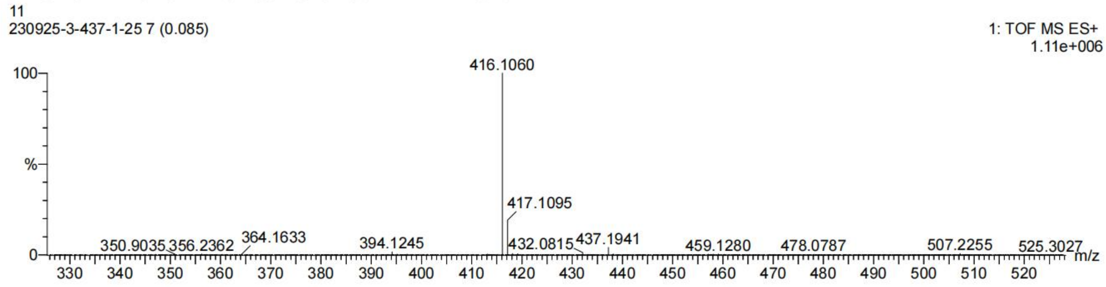

Minimum:				-1.5				
Maximum:		5.0	10.0	50.0				
Mass	Calc. Mass	mDa	PPM	DBE	i-FIT	Norm	Conf(%)	Formula
416.1060	416.1063	-0.3	-0.7	18.5	338.7	n/a	n/a	C26 H16 N O2 F Na

3ab HRMS: (ESI) m/z: $[M+K]^+$ Calcd for $C_{26}H_{16}ClFK$ 421.0556; Found 421.0564.

Monoisotopic Mass, Even Electron Ions
885 formula(e) evaluated with 1 results within limits (up to 50 best isotopic matches for each mass)
Elements Used:
C: 26-26 H: 16-16 N: 0-200 O: 0-200 F: 1-1 Na: 0-1 Cl: 1-2 K: 1-1

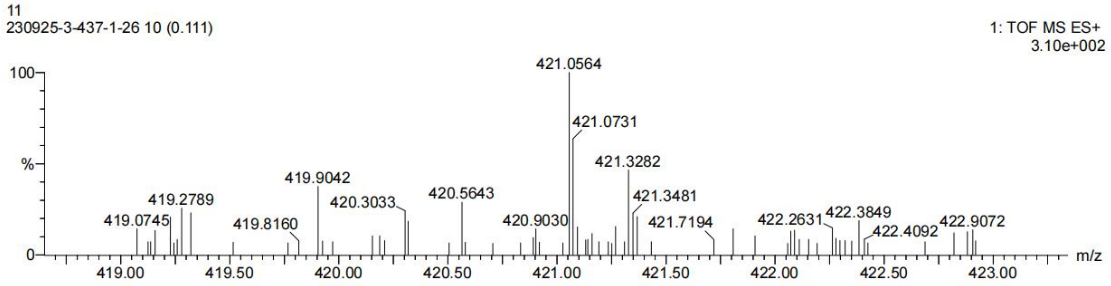

Minimum:				-1.5				
Maximum:		5.0	10.0	50.0				
Mass	Calc. Mass	mDa	PPM	DBE	i-FIT	Norm	Conf(%)	Formula
421.0564	421.0562	0.2	0.5	17.5	267.0	n/a	n/a	C26 H16 F Cl K

3ac HRMS: (ESI) m/z: $[M+H]^+$ Calcd for $C_{26}H_{17}F_2$ 367.1293; Found 367.1304.

Monoisotopic Mass, Even Electron Ions
492 formula(e) evaluated with 1 results within limits (up to 50 best isotopic matches for each mass)
Elements Used:
C: 26-26 H: 17-17 N: 0-200 O: 0-200 F: 2-2 Na: 0-1
11
230925-3-437-1-27 8 (0.094)

1: TOF MS ES+
1.50e+003

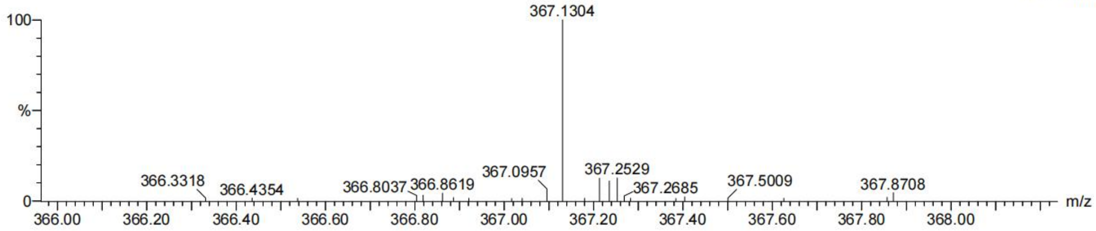

Minimum:				-1.5				
Maximum:		5.0	10.0	50.0				
Mass	Calc. Mass	mDa	PPM	DBE	i-FIT	Norm	Conf(%)	Formula
367.1304	367.1298	0.6	1.6	17.5	116.8	n/a	n/a	C26 H17 F2

3ad HRMS: (ESI) m/z: $[M+K]^+$ Calcd for $C_{27}H_{16}F_4K$ 455.0820; Found 455.0827.

Monoisotopic Mass, Even Electron Ions
527 formula(e) evaluated with 1 results within limits (up to 50 best isotopic matches for each mass)
Elements Used:
C: 27-27 H: 16-16 N: 0-200 O: 0-200 F: 4-4 Na: 0-1 K: 1-1

11
230925-3-437-1-28 51 (0.479)

1: TOF MS ES+
8.30e+001

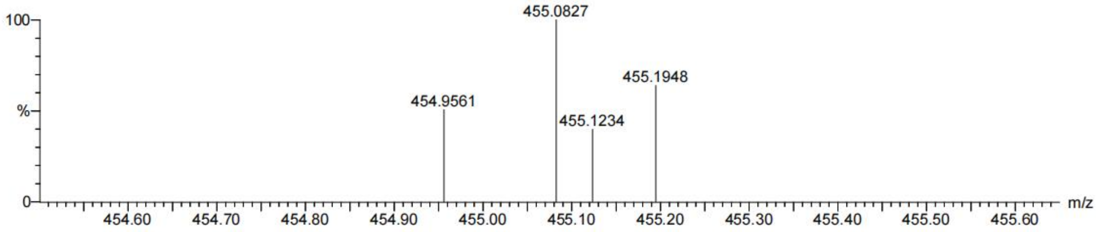

Minimum:				-1.5				
Maximum:		5.0	10.0	50.0				
Mass	Calc. Mass	mDa	PPM	DBE	i-FIT	Norm	Conf(%)	Formula
455.0827	455.0825	0.2	0.4	17.5	32.8	n/a	n/a	C27 H16 F4 K

3ae HRMS: (ESI) m/z: $[M+K]^+$ Calcd for $C_{26}H_{16}ClFK$ 421.0556; Found 421.0553.

Monoisotopic Mass, Even Electron Ions
885 formula(e) evaluated with 1 results within limits (up to 50 best isotopic matches for each mass)
Elements Used:
C: 26-26 H: 16-16 N: 0-200 O: 0-200 F: 1-1 Na: 0-1 Cl: 1-2 K: 1-1
11
230925-3-437-1-29 43 (0.410)

1: TOF MS ES+
4.40e+001

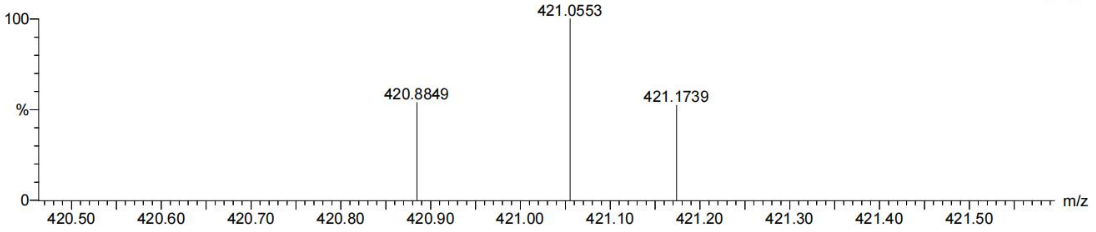

Minimum:				-1.5				
Maximum:		5.0	10.0	50.0				
Mass	Calc. Mass	mDa	PPM	DBE	i-FIT	Norm	Conf(%)	Formula
421.0553	421.0562	-0.9	-2.1	17.5	22.9	n/a	n/a	C26 H16 F Cl K

3af HRMS: (ESI) m/z: $[M+H]^+$ Calcd for $C_{26}H_{17}F_2$ 367.1293; Found 367.1291.

Monoisotopic Mass, Even Electron Ions
492 formula(e) evaluated with 1 results within limits (up to 50 best isotopic matches for each mass)
Elements Used:
C: 26-26 H: 17-17 N: 0-200 O: 0-200 F: 2-2 Na: 0-1

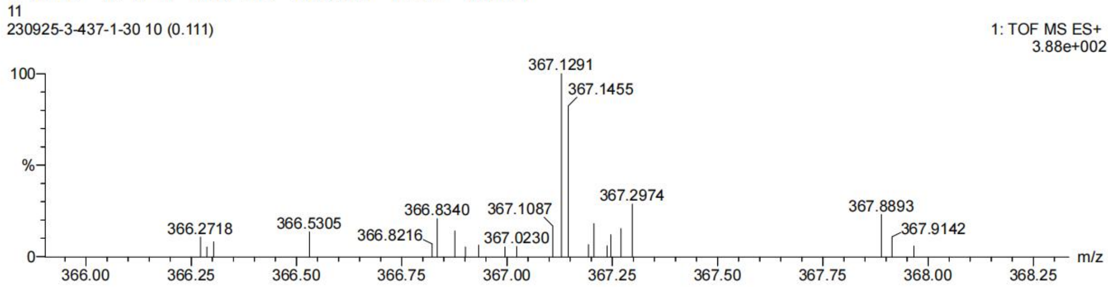

Minimum:				-1.5				
Maximum:		5.0	10.0	50.0				
Mass	Calc. Mass	mDa	PPM	DBE	i-FIT	Norm	Conf(%)	Formula
367.1291	367.1298	-0.7	-1.9	17.5	102.5	n/a	n/a	C26 H17 F2

3ag HRMS: (ESI) m/z: $[M+K]^+$ Calcd for $C_{26}H_{15}F_3K$ 423.0757; Found 423.0754.

Monoisotopic Mass, Even Electron Ions
488 formula(e) evaluated with 1 results within limits (up to 50 best isotopic matches for each mass)
Elements Used:
C: 26-26 H: 15-15 N: 0-200 O: 0-200 F: 3-3 Na: 0-1 K: 1-1

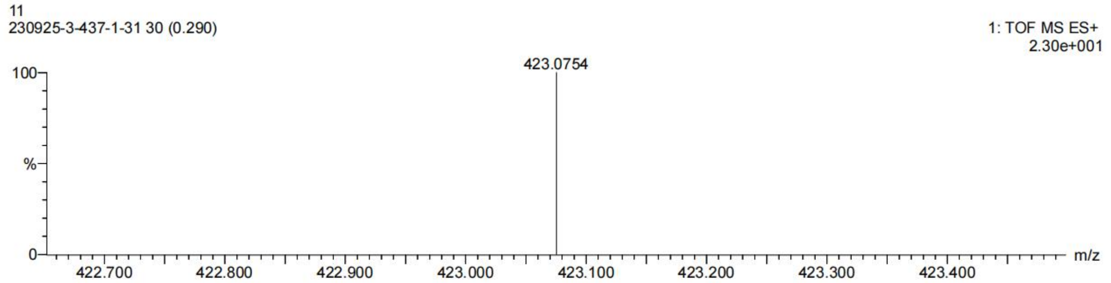

Minimum:				-1.5				
Maximum:		5.0	10.0	50.0				
Mass	Calc. Mass	mDa	PPM	DBE	i-FIT	Norm	Conf(%)	Formula
423.0754	423.0763	-0.9	-2.1	17.5	13.8	n/a	n/a	C26 H15 F3 K

3ah HRMS: (ESI) m/z: $[M+K]^+$ Calcd for $C_{30}H_{20}K$ 419.1197; Found 419.1204.

Monoisotopic Mass, Even Electron Ions
656 formula(e) evaluated with 1 results within limits (up to 50 best isotopic matches for each mass)
Elements Used:
C: 30-30 H: 20-20 N: 0-200 O: 0-200 Na: 0-1 K: 1-1

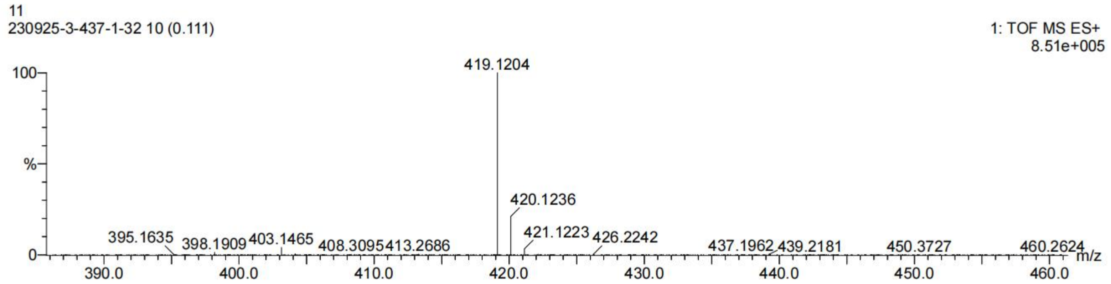

Minimum:				-1.5				
Maximum:		5.0	10.0	50.0				
Mass	Calc. Mass	mDa	PPM	DBE	i-FIT	Norm	Conf(%)	Formula
419.1204	419.1202	0.2	0.5	20.5	284.5	n/a	n/a	C30 H20 K

3ai HRMS: (ESI) m/z: $[M+Na]^+$ Calcd for $C_{33}H_{21}NNa$ 454.1566; Found 454.1572.

Monoisotopic Mass, Even Electron Ions
57 formula(e) evaluated with 1 results within limits (up to 50 best isotopic matches for each mass)
Elements Used:
C: 33-33   H: 21-21   N: 0-200   Na: 0-1

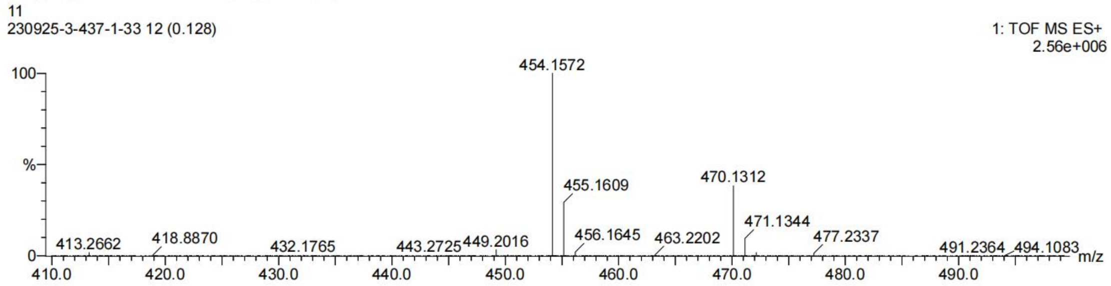

Minimum:				-1.5				
Maximum:		5.0	10.0	50.0				
Mass	Calc. Mass	mDa	PPM	DBE	i-FIT	Norm	Conf(%)	Formula
454.1572	454.1572	0.0	0.0	23.5	335.1	n/a	n/a	C33 H21 N Na

3aj HRMS: (ESI) m/z: $[M+K]^+$ Calcd for $C_{46}H_{32}K$ 623.2136; Found 623.2134.

Monoisotopic Mass, Even Electron Ions
79 formula(e) evaluated with 1 results within limits (up to 50 best isotopic matches for each mass)
Elements Used:
C: 46-46   H: 32-32   N: 0-200   Na: 0-1   K: 1-1

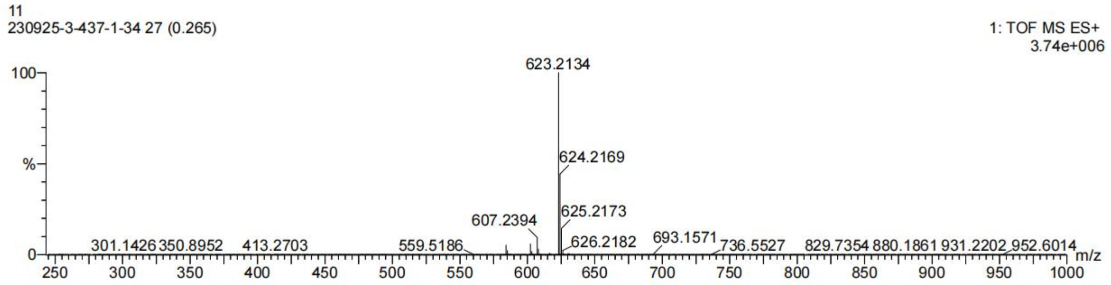

Minimum:				-1.5				
Maximum:		5.0	10.0	50.0				
Mass	Calc. Mass	mDa	PPM	DBE	i-FIT	Norm	Conf(%)	Formula
623.2134	623.2141	-0.7	-1.1	30.5	391.5	n/a	n/a	C46 H32 K

3ak HRMS: (ESI) m/z: $[M+Na]^+$ Calcd for $C_{37}H_{30}O_2Na$ 529.2138; Found 529.2137.

Monoisotopic Mass, Even Electron Ions
56 formula(e) evaluated with 1 results within limits (up to 50 best isotopic matches for each mass)
Elements Used:
C: 37-37   H: 30-30   O: 0-200   Na: 0-1

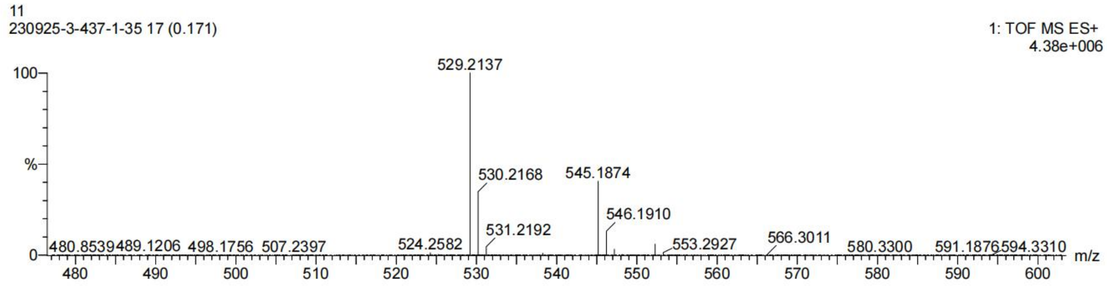

Minimum:				-1.5				
Maximum:		5.0	10.0	50.0				
Mass	Calc. Mass	mDa	PPM	DBE	i-FIT	Norm	Conf(%)	Formula
529.2137	529.2143	-0.6	-1.1	22.5	420.3	n/a	n/a	C37 H30 O2 Na

3al HRMS: (ESI) m/z: $[M+Na]^+$ Calcd for $C_{52}H_{36}N_4Na$ 739.2832; Found 739.2845.

Monoisotopic Mass, Even Electron Ions
2396 formula(e) evaluated with 1 results within limits (up to 50 best isotopic matches for each mass)
Elements Used:
C: 52-52 H: 36-36 N: 0-200 O: 0-200 Na: 0-1

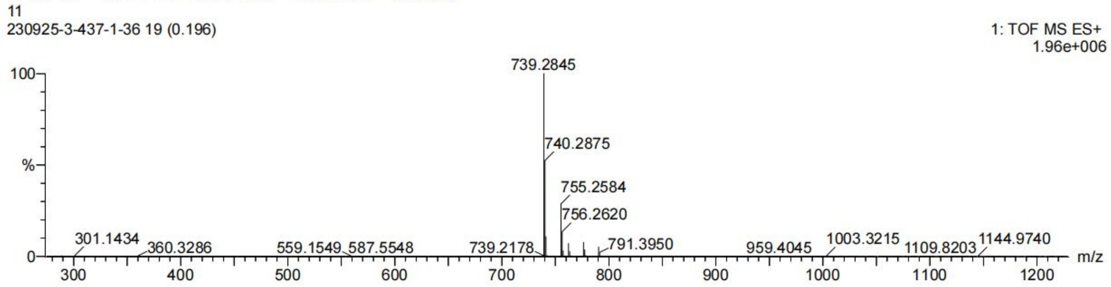

Minimum:				-1.5				
Maximum:		5.0	10.0	50.0				

Mass	Calc. Mass	mDa	PPM	DBE	i-FIT	Norm	Conf(%)	Formula
739.2845	739.2838	0.7	0.9	36.5	253.2	n/a	n/a	C52 H36 N4 Na

3am HRMS: (ESI) m/z: $[M+Na]^+$ Calcd for $C_{30}H_{22}O_2Na$ 437.1512; Found 437.1516.

Monoisotopic Mass, Even Electron Ions
836 formula(e) evaluated with 1 results within limits (up to 50 best isotopic matches for each mass)
Elements Used:
C: 30-30 H: 22-22 N: 0-200 O: 0-200 Na: 0-1

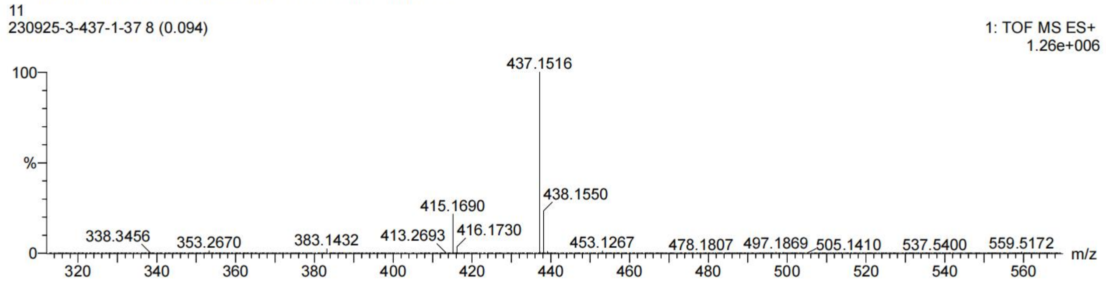

Minimum:				-1.5				
Maximum:		5.0	10.0	50.0				

Mass	Calc. Mass	mDa	PPM	DBE	i-FIT	Norm	Conf(%)	Formula
437.1516	437.1517	-0.1	-0.2	19.5	288.7	n/a	n/a	C30 H22 O2 Na

3an HRMS: (ESI) m/z: $[M+H]^+$ Calcd for $C_{25}H_{17}BrN$ 410.0539; Found 410.0546.

Monoisotopic Mass, Even Electron Ions
787 formula(e) evaluated with 1 results within limits (up to 50 best isotopic matches for each mass)
Elements Used:
C: 25-25 H: 17-17 N: 0-200 O: 0-200 Na: 0-1 Br: 1-2

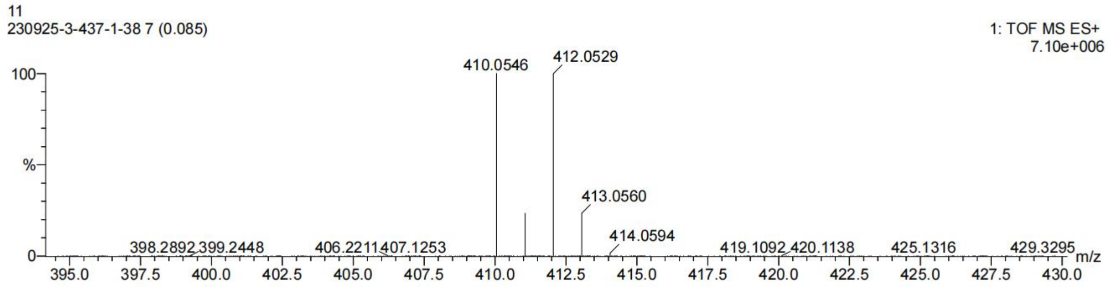

Minimum:				-1.5				
Maximum:		5.0	10.0	50.0				

Mass	Calc. Mass	mDa	PPM	DBE	i-FIT	Norm	Conf(%)	Formula
410.0546	410.0544	0.2	0.5	17.5	497.6	n/a	n/a	C25 H17 N Br

3ao HRMS: (ESI) m/z: $[M+H]^+$ Calcd for $C_{25}H_{17}BrN$ 410.0539; Found 410.0542.

Monoisotopic Mass, Even Electron Ions
787 formula(e) evaluated with 1 results within limits (up to 50 best isotopic matches for each mass)
Elements Used:
C: 25-25 H: 17-17 N: 0-200 O: 0-200 Na: 0-1 Br: 1-2

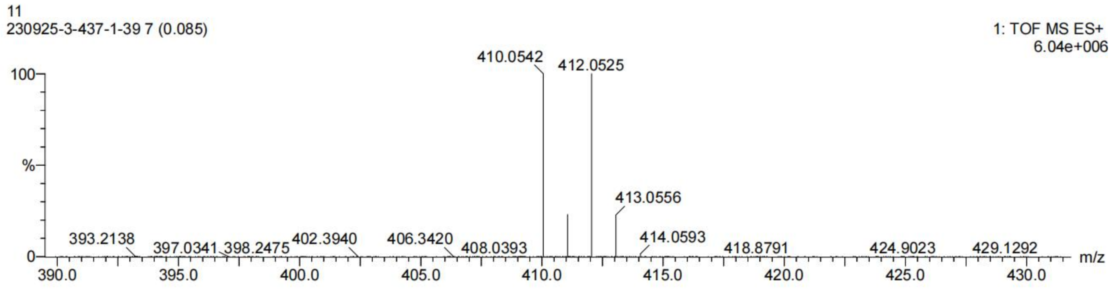

Minimum:				-1.5				
Maximum:		5.0	10.0	50.0				
Mass	Calc. Mass	mDa	PPM	DBE	i-FIT	Norm	Conf(%)	Formula
410.0542	410.0544	-0.2	-0.5	17.5	448.0	n/a	n/a	C25 H17 N Br

3ap HRMS: (ESI) m/z: $[M+H]^+$ Calcd for $C_{23}H_{15}BrNS$ 416.0103; Found 416.0107.

Monoisotopic Mass, Even Electron Ions
1175 formula(e) evaluated with 1 results within limits (up to 50 best isotopic matches for each mass)
Elements Used:
C: 23-23 H: 15-15 N: 0-200 O: 0-200 Na: 0-1 S: 1-2 Br: 1-2

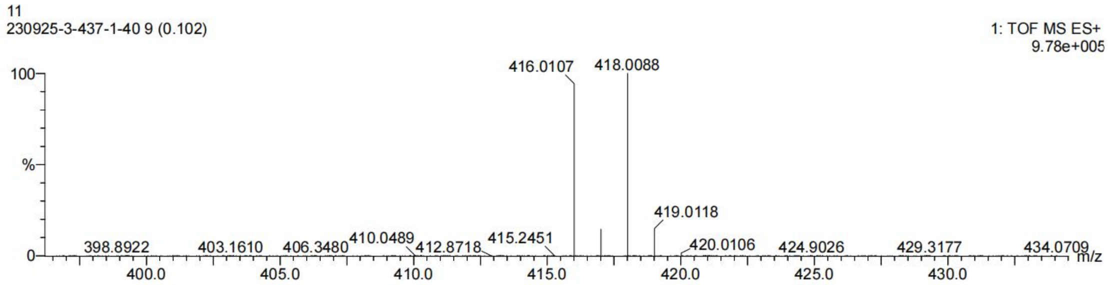

Minimum:				-1.5				
Maximum:		5.0	10.0	50.0				
Mass	Calc. Mass	mDa	PPM	DBE	i-FIT	Norm	Conf(%)	Formula
416.0107	416.0109	-0.2	-0.5	16.5	354.1	n/a	n/a	C23 H15 N S Br

3aq HRMS: (ESI) m/z: $[M+H]^+$ Calcd for $C_{25}H_{16}F_3O$ 389.1148; Found 389.1155.

Monoisotopic Mass, Even Electron Ions
497 formula(e) evaluated with 1 results within limits (up to 50 best isotopic matches for each mass)
Elements Used:
C: 25-25 H: 16-16 N: 0-200 O: 0-200 Na: 0-1 F: 3-3

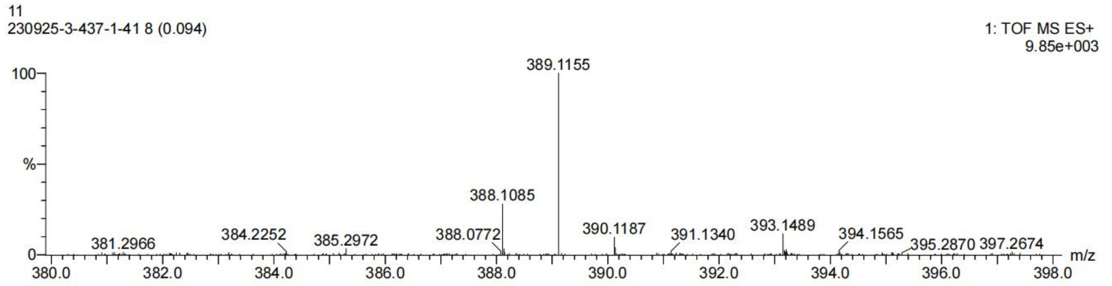

Minimum:				-1.5				
Maximum:		5.0	10.0	50.0				
Mass	Calc. Mass	mDa	PPM	DBE	i-FIT	Norm	Conf(%)	Formula
389.1155	389.1153	0.2	0.5	16.5	259.3	n/a	n/a	C25 H16 O F3

3ar HRMS: (ESI) m/z: $[M+K]^+$ Calcd for $C_{32}H_{22}K$ 445.1353; Found 445.1354.

Monoisotopic Mass, Even Electron Ions
747 formula(e) evaluated with 1 results within limits (up to 50 best isotopic matches for each mass)
Elements Used:
C: 32-32 H: 22-22 N: 0-200 O: 0-200 Na: 0-1 K: 1-1

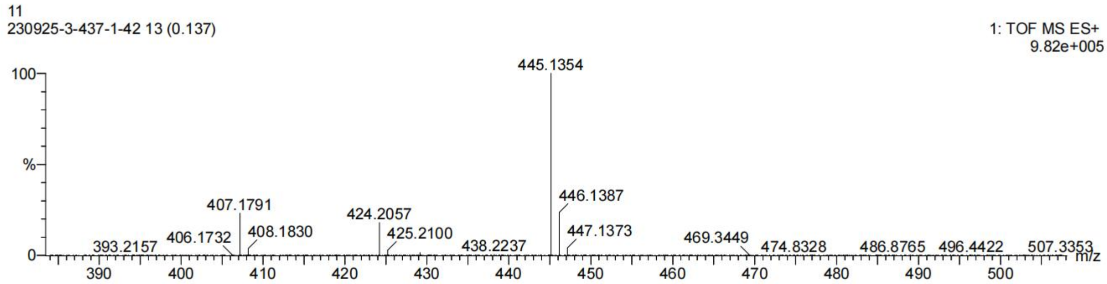

Minimum:				-1.5				
Maximum:		5.0	10.0	50.0				
Mass	Calc. Mass	mDa	PPM	DBE	i-FIT	Norm	Conf(%)	Formula
445.1354	445.1359	-0.5	-1.1	21.5	209.0	n/a	n/a	C32 H22 K

3as HRMS: (ESI) m/z: $[M+K]^+$ Calcd for $C_{32}H_{21}ClK$ 479.0963; Found 479.0968.

Monoisotopic Mass, Even Electron Ions
1369 formula(e) evaluated with 1 results within limits (up to 50 best isotopic matches for each mass)
Elements Used:
C: 32-32 H: 21-21 N: 0-200 O: 0-200 Na: 0-1 Cl: 1-2 K: 1-1

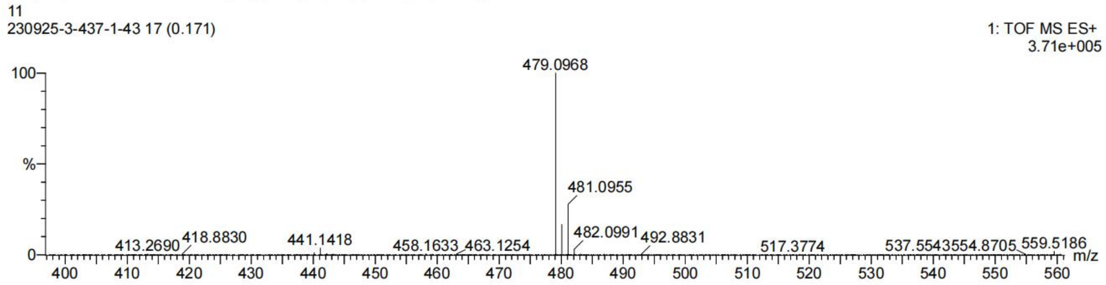

Minimum:				-1.5				
Maximum:		5.0	10.0	50.0				
Mass	Calc. Mass	mDa	PPM	DBE	i-FIT	Norm	Conf(%)	Formula
479.0968	479.0969	-0.1	-0.2	21.5	335.5	n/a	n/a	C32 H21 Cl K

3at HRMS: (ESI) m/z: $[M+K]^+$ Calcd for $C_{34}H_{26}K$ 473.1666; Found 473.1667.

Monoisotopic Mass, Even Electron Ions
851 formula(e) evaluated with 1 results within limits (up to 50 best isotopic matches for each mass)
Elements Used:
C: 34-34 H: 26-26 N: 0-200 O: 0-200 Na: 0-1 K: 1-1

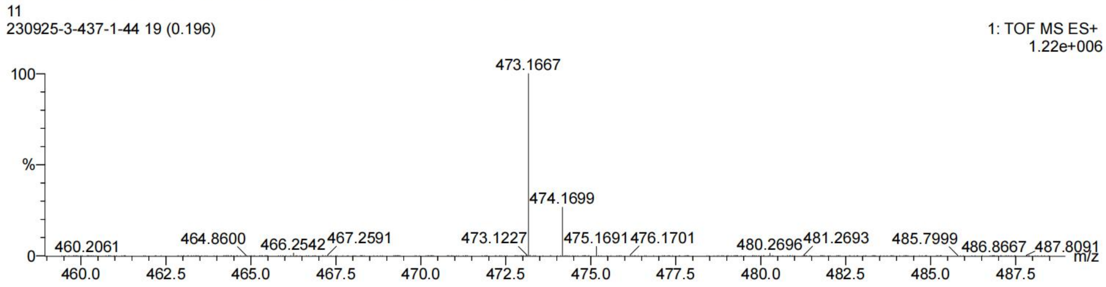

Minimum:				-1.5				
Maximum:		5.0	10.0	50.0				
Mass	Calc. Mass	mDa	PPM	DBE	i-FIT	Norm	Conf(%)	Formula
473.1667	473.1672	-0.5	-1.1	21.5	244.2	n/a	n/a	C34 H26 K

3au HRMS: (ESI) m/z: $[M+K]^+$ Calcd for $C_{33}H_{21}F_3OK$ 529.1176; Found 529.1180.

Monoisotopic Mass, Even Electron Ions
848 formula(e) evaluated with 1 results within limits (up to 50 best isotopic matches for each mass)
Elements Used:
C: 33-33 H: 1-21 N: 0-200 O: 0-200 Na: 0-1 K: 1-1 F: 3-3

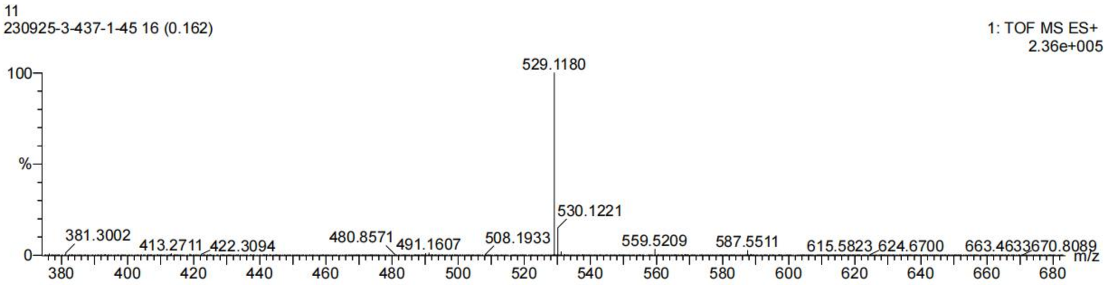

Minimum:				-1.5				
Maximum:		5.0	10.0	50.0				
Mass	Calc. Mass	mDa	PPM	DBE	i-FIT	Norm	Conf(%)	Formula
529.1180	529.1182	-0.2	-0.4	21.5	291.7	n/a	n/a	C33 H21 O K F3

3av HRMS: (ESI) m/z: $[M+K]^+$ Calcd for $C_{38}H_{26}K$ 521.1666; Found 521.1675.

Monoisotopic Mass, Even Electron Ions
1049 formula(e) evaluated with 1 results within limits (up to 50 best isotopic matches for each mass)
Elements Used:
C: 38-38 H: 26-26 N: 0-100 O: 0-100 Na: 0-1 K: 1-1

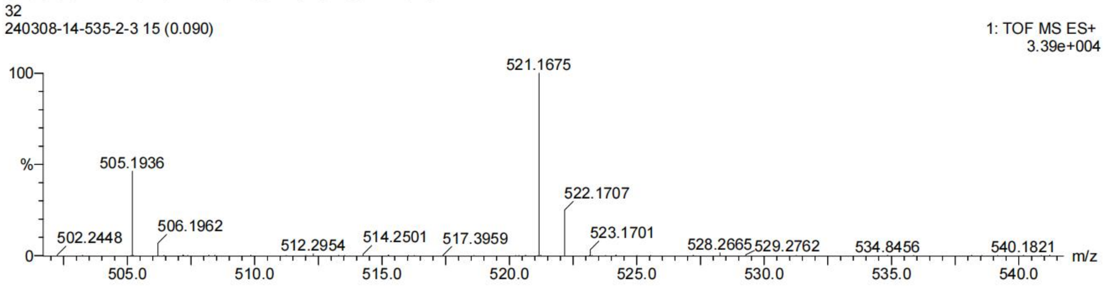

Minimum:				-1.5				
Maximum:		5.0	10.0	50.0				
Mass	Calc. Mass	mDa	PPM	DBE	i-FIT	Norm	Conf(%)	Formula
521.1675	521.1672	0.3	0.6	25.5	41.8	n/a	n/a	C38 H26 K

3aw HRMS: (ESI) m/z: $[M+K]^+$ Calcd for $C_{26}H_{17}ClK$ 403.0650; Found 403.0649.

Monoisotopic Mass, Even Electron Ions
889 formula(e) evaluated with 1 results within limits (up to 50 best isotopic matches for each mass)
Elements Used:
C: 26-26 H: 17-17 N: 0-100 O: 0-100 Cl: 1-2 Na: 0-1 K: 1-1

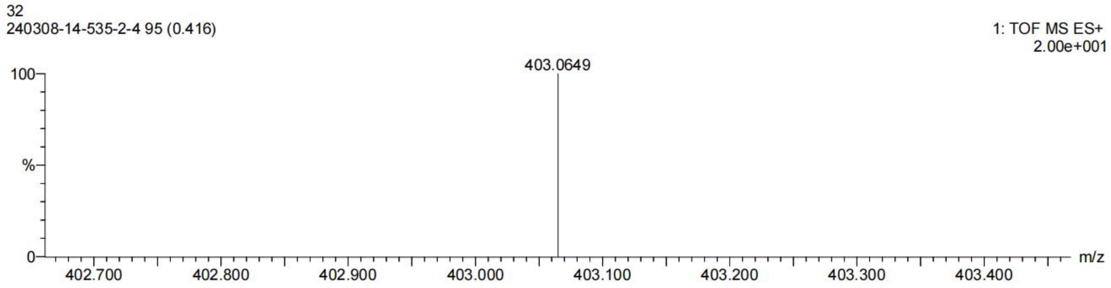

Minimum:				-1.5				
Maximum:		5.0	10.0	50.0				
Mass	Calc. Mass	mDa	PPM	DBE	i-FIT	Norm	Conf(%)	Formula
403.0649	403.0656	-0.7	-1.7	17.5	14.2	n/a	n/a	C26 H17 Cl K

3ax HRMS: (ESI) m/z: $[M+H]^+$ Calcd for $C_{28}H_{19}N_2$ 383.1543; Found 383.1551.

Monoisotopic Mass, Even Electron Ions
655 formula(e) evaluated with 1 results within limits (up to 50 best isotopic matches for each mass)
Elements Used:
C: 28-28 H: 19-19 N: 0-200 O: 0-200 Na: 0-1
11
230925-3-437-1-46 14 (0.145)

1: TOF MS ES+
2.25e+006

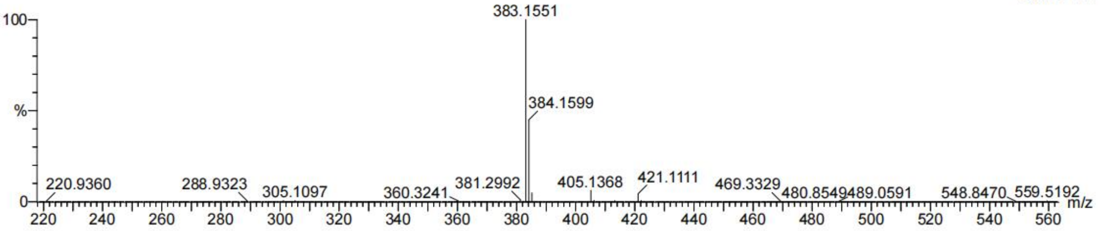

Minimum:				-1.5				
Maximum:		5.0	10.0	50.0				
Mass	Calc. Mass	mDa	PPM	DBE	i-FIT	Norm	Conf(%)	Formula
383.1551	383.1548	0.3	0.8	20.5	417.4	n/a	n/a	C28 H19 N2

3ay HRMS: (ESI) m/z: $[M+H]^+$ Calcd for $C_{22}H_{17}$ 281.1325; Found 281.1327.

Monoisotopic Mass, Even Electron Ions
360 formula(e) evaluated with 1 results within limits (up to 50 best isotopic matches for each mass)
Elements Used:
C: 22-22 H: 17-17 N: 0-200 O: 0-200 Na: 0-1
11
230925-3-437-1-47 8 (0.094)

1: TOF MS ES+
8.80e+003

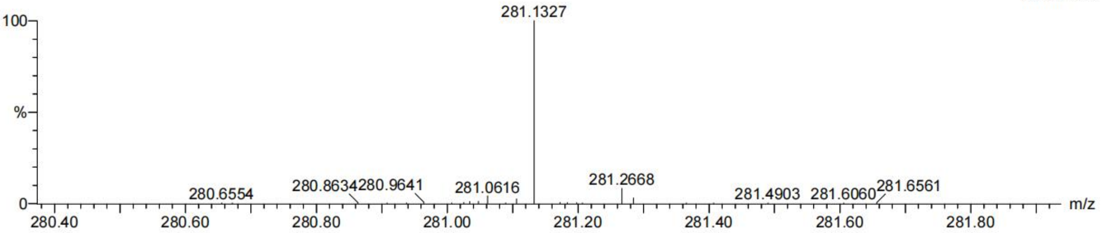

Minimum:				-1.5				
Maximum:		5.0	10.0	50.0				
Mass	Calc. Mass	mDa	PPM	DBE	i-FIT	Norm	Conf(%)	Formula
281.1327	281.1330	-0.3	-1.1	14.5	112.2	n/a	n/a	C22 H17

4 HRMS: (ESI) m/z: $[M+H]^+$ Calcd for $C_{29}H_{25}N_2O$ 417.1961; Found 417.1969.

Monoisotopic Mass, Even Electron Ions
400 formula(e) evaluated with 1 results within limits (up to 50 best isotopic matches for each mass)
Elements Used:
C: 29-29 H: 25-25 N: 0-200 O: 0-200
12
230925-3-437-1-56 8 (0.094)

1: TOF MS ES+
4.99e+006

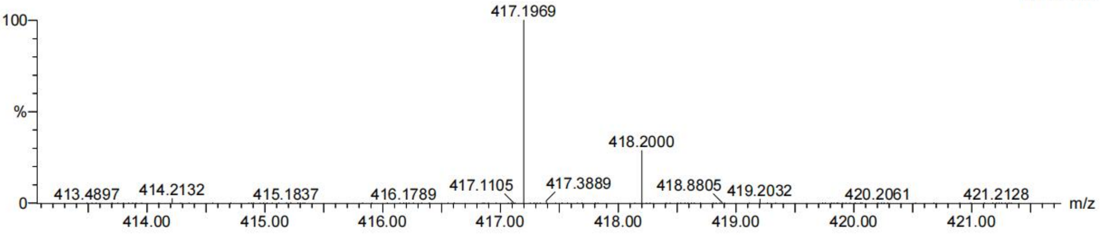

Minimum:				-1.5				
Maximum:		5.0	10.0	50.0				
Mass	Calc. Mass	mDa	PPM	DBE	i-FIT	Norm	Conf(%)	Formula
417.1969	417.1967	0.2	0.5	18.5	307.5	n/a	n/a	C29 H25 N2 O

5 HRMS: (ESI) m/z: $[M+Na]^+$ Calcd for $C_{31}H_{25}NNa$ 434.1879; Found 434.1890.

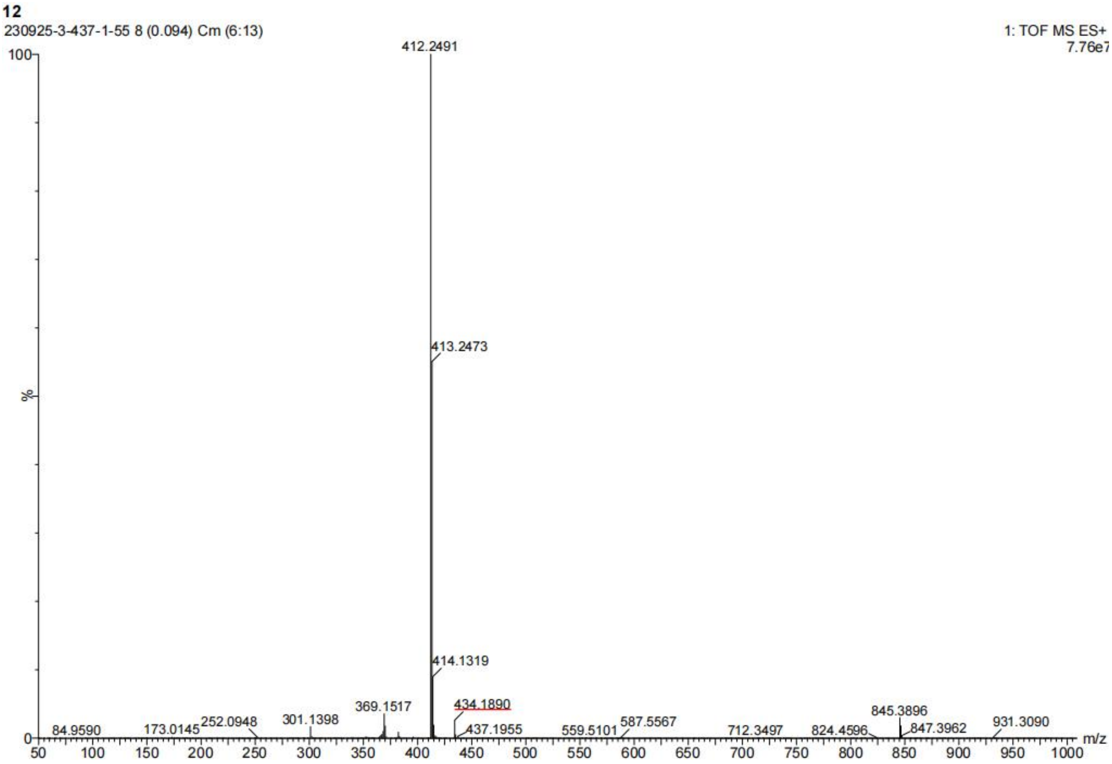

6 HRMS: (ESI) m/z: [M+H]$^+$ Calcd for $C_{29}H_{20}NS$ 414.1311; Found 414.1307.

Monoisotopic Mass, Even Electron Ions
349 formula(e) evaluated with 1 results within limits (up to 50 best isotopic matches for each mass)
Elements Used:
C: 29-29 H: 20-20 N: 0-200 O: 0-200 S: 1-1

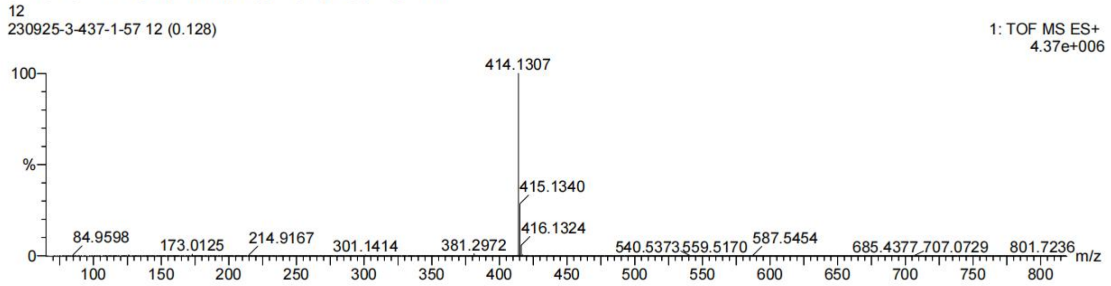

Minimum:				-1.5				
Maximum:		5.0	10.0	50.0				

Mass	Calc. Mass	mDa	PPM	DBE	i-FIT	Norm	Conf(%)	Formula
414.1307	414.1316	-0.9	-2.2	20.5	352.9	n/a	n/a	C29 H20 N S

13. Supplementary References

1. Tochtermann, W.; Oppenlaender, K. & Walter, U. Seven-membered ring systems. II. Synthesis and rearrangement of tribenzocycloheptatrienone derivatives. *Chem. Ber.* **975**, 1329−1336 (1964).

2. Pun, S. H.; Miao, Q. Introduction of Eight-Membered Rings to Polycyclic Arenes by Ring Expansion. *Chin. J. Org. Chem.* **40**, 3347−3353 (2020).

3. Zhou, L.; Sun, M.; Zhou, F.; Deng, Gu.; Yang, Y. & Liang, Y. Atmosphere-Controlled Palladium-Catalyzed Divergent Decarboxylative Cyclization of 2-Iodobiphenyls and α-Oxocarboxylic Acids. *Org. Lett.* **23**, 7150−7155 (2021).

4. Chang, M.-Y.; Tsai, C.-Y. & Chan, C.-K. *m*CPBA-mediated conjugation of dibenzosuberenone and amines or carboxylic acids. *Tetrahedron* **71**, 424−430 (2015).
